# Systematic Dissection of the Evolutionarily Conserved WetA Developmental Regulator across a Genus of Filamentous Fungi

**DOI:** 10.1128/mBio.01130-18

**Published:** 2018-08-21

**Authors:** Ming-Yueh Wu, Matthew E. Mead, Mi-Kyung Lee, Erin M. Ostrem Loss, Sun-Chang Kim, Antonis Rokas, Jae-Hyuk Yu

**Affiliations:** a Department of Bacteriology, University of Wisconsin—Madison, Madison, Wisconsin, USA; b Laboratory of Genetics, University of Wisconsin—Madison, Madison, Wisconsin, USA; c Department of Biological Sciences, Vanderbilt University, Nashville, Tennessee, USA; d Biological Resource Center, Korea Research Institute of Bioscience and Biotechnology (KRIBB), Jeongeup-si, Republic of Korea; e Molecular and Environmental Toxicology Center, University of Wisconsin—Madison, Madison, Wisconsin, USA; f Department of Biological Sciences, Korea Advanced Institute of Science and Technology, Dae-Jon, Republic of Korea; g Department of Systems Biotechnology, Konkuk University, Seoul, Republic of Korea; Universidade de Sao Paulo

**Keywords:** *Aspergillus*, WetA, asexual development, gene regulatory network, sporulation

## Abstract

Asexual sporulation is fundamental to the ecology and lifestyle of filamentous fungi and can facilitate both plant and human infection. In *Aspergillus*, the production of asexual spores is primarily governed by the BrlA→AbaA→WetA regulatory cascade. The final step in this cascade is controlled by the WetA protein and governs not only the morphological differentiation of spores but also the production and deposition of diverse metabolites into spores. While WetA is conserved across the genus *Aspergillus*, the structure and degree of conservation of the *wetA* gene regulatory network (GRN) remain largely unknown. We carried out comparative transcriptome analyses of comparisons between *wetA* null mutant and wild-type asexual spores in three representative species spanning the diversity of the genus *Aspergillus*: A. nidulans, A. flavus, and A. fumigatus. We discovered that WetA regulates asexual sporulation in all three species via a negative-feedback loop that represses BrlA, the cascade’s first step. Furthermore, data from chromatin immunoprecipitation sequencing (ChIP-seq) experiments in A. nidulans asexual spores suggest that WetA is a DNA-binding protein that interacts with a novel regulatory motif. Several global regulators known to bridge spore production and the production of secondary metabolites show species-specific regulatory patterns in our data. These results suggest that the BrlA→AbaA→WetA cascade’s regulatory role in cellular and chemical asexual spore development is functionally conserved but that the *wetA*-associated GRN has diverged during *Aspergillus* evolution.

## IMPORTANCE

The formation of resilient spores is a key factor contributing to the survival and fitness of many microorganisms, including fungi. In the fungal genus *Aspergillus*, spore formation is controlled by a complex gene regulatory network that also impacts a variety of other processes, including secondary metabolism. To gain mechanistic insights into how fungal spore formation is controlled across *Aspergillus*, we dissected the gene regulatory network downstream of a major regulator of spore maturation (WetA) in three species that span the diversity of the genus: the genetic model A. nidulans, the human pathogen A. fumigatus, and the aflatoxin producer A. flavus. Our data show that WetA regulates asexual sporulation in all three species via a negative-feedback loop and likely binds a novel regulatory element that we term the WetA response element (WRE). These results shed light on how gene regulatory networks in microorganisms control important biological processes and evolve across diverse species.

## INTRODUCTION

The ability to produce numerous asexual spores is one of the key factors contributing to the fecundity and fitness of filamentous fungi. Fungal asexual spores are highly efficient for genome protection, survival, and propagation. Spores are also the primary means of infecting host organisms for many pathogenic fungi (1). Importantly, in some filamentous fungi, morphological development is coordinated with the production of secondary metabolites with toxic and antibiotic properties (2–4).

Asexual development (conidiation) in the fungal class Eurotiomycetes results in the formation of mitotically derived asexual spores known as conidiospores or conidia. As asexual sporulation is widespread among fungi, it represents a simple, highly tractable system for understanding how gene regulatory networks (GRNs) evolve in microbial eukaryotes and how this evolution has influenced developmental and metabolic phenotypes.

Members of the genus *Aspergillus* are ubiquitous in most environments and include various beneficial, pathogenic, and/or toxigenic species (5). All aspergilli produce conidia as the main means of dispersion and infection. Importantly, the asexual development and the production of certain secondary metabolites, including mycotoxins, are intimately associated (2).

**FIG 1:**
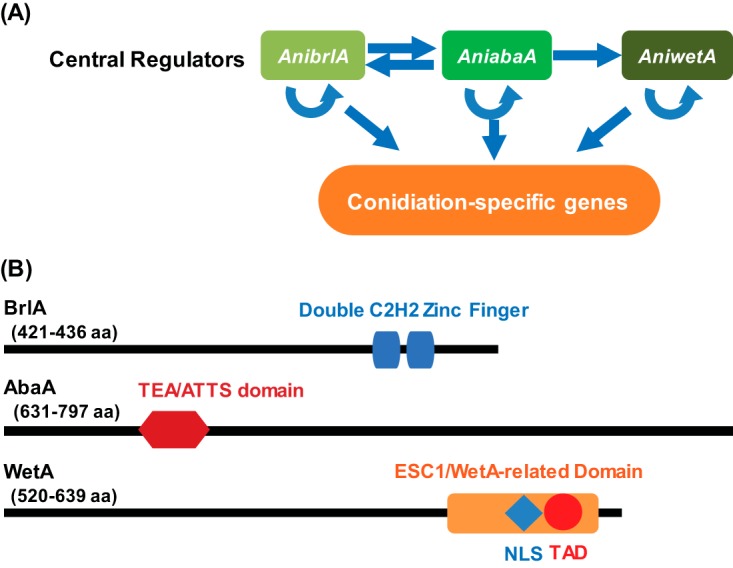
The central regulatory pathway of *Aspergillus* conidiation. (A) A cartoon depiction of genetic interactions of the central regulators in A. nidulans conidiogenesis. The central regulators cooperatively activate the conidiation-specific genes responsible for the morphogenesis of conidiophores. (B) The predicted protein architectures for the three conserved central regulators of conidiation in A. nidulans, A. fumigatus, and A. flavus. The blue box and the red hexagon represent the C2H2 zinc finger domain and TEA/ATTS domain in BrlA and AbaA, respectively, and were identified in a blastP (version 2.6.0) search (71). The red circle represents a putative transcription activation domain (TAD), which was predicted by 9aaTAD using the “Less stringent Pattern” setting (31), and in A. nidulans it has the amino acid sequence SEAALQAVR. The blue diamond represents the nuclear localization signal (NLS) predicted by NLStradamus using the 4 state HMM static model (32), and in A. nidulans it has the amino acid sequence KTKARREQEARDRRRK. The orange rectangle represents the ESC1/WetA-related domain (PTHR22934) predicted by the PANTHER classification system (72) and located at amino acids 497 to 547 in the A. nidulans protein.

The three distantly related species Aspergillus nidulans, Aspergillus flavus, and Aspergillus fumigatus, whose pairwise genome similarities are similar to the genomic similarities between the human and fish genomes (6), form distinct conidiophores with various sizes of conidia. The regulatory mechanisms of conidiation have been extensively studied in A. nidulans (7–23). The regulatory genes can be divided into central regulators, upstream activators, negative regulators, light-dependent regulators, and the *velvet* regulators (24, 25). The central genetic regulatory cascade BrlA→AbaA→WetA is present in *Aspergillus* and governs both conidiation-specific GRNs and the resulting morphological pathway of conidiation (Fig. 1A) (22, 24, 26). BrlA is a C_2_H_2_-zinc finger-type transcription factor (TF) which recognizes and interacts with BrlA response elements (BRE) (Fig. 1B) (27, 28). The *brlA* gene is expressed in the early phase of conidiation and mediates vesicle formation and budding-like cell growth (11). The *abaA* gene is activated by BrlA and regulates the formation of metulae and phialides. Similarly to BrlA, AbaA is a TF, containing a TEA/ATTS DNA binding motif and a potential leucine zipper that recognizes the AbaA response elements (AREs) (Fig. 1B) (29). The *wetA* (wet-white A) gene, activated by AbaA, functions in the late phase of conidiation that completes sporogenesis. The BrlA→AbaA→WetA central regulatory cascade acts in concert with other genes to control conidiation-specific gene expression and determine the order of gene activation during the cellular and chemical development of spores.

The WetA protein plays a pivotal role in the coordinated control of *Aspergillus* conidiogenesis; however, the precise molecular mechanisms of WetA function have been unknown. WetA is highly and broadly conserved in Ascomycetes (8, 10–13, 15–23, 26, 30), plays an essential role in the synthesis of crucial conidial wall components, and makes the conidia both impermeable and mature (20, 21, 30). The *Aspergillus* WetA proteins have a conserved ESC1/WetA-related domain that is annotated as a DNA-binding domain by the PANTHER classification system (PTHR22934: SF23) and contains both a predicted transcription activation domain (TAD) (31) and a nuclear localization signal (NLS) (32, 33) near the C terminus (16, 34). Taken together, the data suggest that WetA is likely a DNA-binding TF (30) (Fig. 1B). As summarized in Table 1, the deletion of *wetA* results in a plethora of conidial defects, including the formation of colorless conidia that undergo autolysis in A. nidulans (10–13, 20–22, 26), A. fumigatus (8, 15), A. oryzae (18), and A. flavus (30). The metabolism and expression of several conidial components are perturbed in the Δ*wetA* conidia, leading to reduced stress tolerance and spore viability (8, 30).

**TABLE 1:** Phenotypes of Δ*wetA* mutants in three *Aspergillus* species

Parameter (reference[s])	Result for the *Aspergillus* Δ*wetA* strains:		
	Δ*AniwetA*	Δ*AfuwetA*	Δ*AflwetA*
Conidia			
Colorless and autolyzed (8, 10–13, 20–22, 26, 30)	+	+	+
Reduced size (8)	+	+	+
Disrupted wall structure (8, 20, 21, 30, 35)	+	+	+
Disrupted C2 layer thickness (8, 30)	Thicker	Thicker	Thinner
Reduced viability and stress tolerance (8, 30)	+	+	+
Reduced trehalose amount (M.-Y. Wu and J.-H. Yu, unpublished data) (8, 30)	+	+	+
Increased β-(1,3)-glucan amount (30)	+	NA	+
Reduced light-dependent conidiation (30)	NA^a^	NA	+
Disrupted conidiation time (M.-Y. Wu and J.-H. Yu, unpublished data) (8, 30)	−	Postponed	Advanced
			
Hyphae			
Reduced growth rate (M.-Y. Wu and J.-H. Yu, unpublished data) (8, 30)	+	+	+
Higher branching rate (M.-Y. Wu and J.-H. Yu, unpublished data) (8, 30)	+	+	+
Reduced aflatoxin production (30)	NA	NA	+

a NA, not applicable.

In this study, we investigated the structure and degree of conservation of the BrlA→AbaA→WetA central regulatory cascade of *Aspergillus* conidiation and the broader *wetA* GRNs in three representative *Aspergillus* species: the genetic model A. nidulans, the mycotoxin producer A. flavus, and the human pathogen A. fumigatus. Specifically, we carried out comparative transcriptome analyses of comparisons between *wetA* null mutant and wild-type (WT) asexual spores in the three species. We also investigated the WetA-chromatin interaction in asexual spores via chromatin immunoprecipitation sequencing (ChIP-seq) in A. nidulans spores, which enabled us to identify the consensus WetA-associated DNA sequence. Further comparative genome-wide analyses revealed that the WetA-associated GRN has diverged during *Aspergillus* evolution, uncovering important and yet unexplored regulatory networks of asexual sporulation and metabolic remodeling in *Aspergillus*. Our findings provide the first clear and systematic dissection of the evolutionarily conserved WetA developmental regulator governing the diverged processes of cellular differentiation, chemical development, and cell survival across a genus of filamentous fungi.

## RESULTS

### Conserved and diverged roles for WetA in the control of gene expression in aspergilli.

To investigate the conserved and divergent regulatory roles that WetA plays in the three *Aspergillus* species, we carried out comprehensive analyses of gene expression differences between the WT and *wetA* null mutant conidia. We found that WetA plays a broad regulatory role in conidia in all three *Aspergillus* species; approximately 52%, 57%, and 43% of all genes showed differential accumulation of mRNAs in the Δ*wetA* conidia in comparison to WT conidia in A. nidulans, A. fumigatus, and A. flavus, respectively (Table 2). Among the differentially expressed genes (DEGs), 46%, 48%, and 50% were underexpressed and 54%, 52%, and 50% were overexpressed in the Δ*wetA* conidia compared to the WT conidia in A. nidulans, A. fumigatus, and A. flavus, respectively (Table 2).

**TABLE 2:** Summary of DEGs in the three *Aspergillus* Δ*wetA* conidial species

Gene category	No. (%) of genes		
	A. nidulans	A. fumigatus	A. flavus
Unaffected	5,246 (48)	4,374 (43)	7,730 (57)
Differentially expressed	5,742 (52)	5,756 (57)	5,755 (43)
Overexpressed in mutant Δ*wetA*	3,107 (28)	2,998 (30)	2,899 (21)
Underexpressed in mutant Δ*wetA*	2,635 (24)	2,758 (27)	2,856 (21)
			
Total	10,988	10,130	13,485

Functional category analysis was carried out by determining Gene Ontology (GO) terms that were enriched in DEGs. Specifically, the biological process GO categories that were enriched in the Δ*wetA* conidia included “asexual sporulation,” “secondary metabolic process,” and “toxin biosynthetic process.” Moreover, over 70% of all genes in the cellular component GO category “fungal-type cell wall” were also regulated in each species. These top enriched GO categories are consistent with the phenotypes of the Δ*wetA* mutants, suggesting that WetA plays a key role in carbohydrate metabolism, secondary metabolism, and conidial wall integrity (30).

To explore the conserved and diverged regulatory roles of WetA, we examined the mRNA expression profiles of orthologous groups of genes (orthogroups) in the three *Aspergillus* genomes. A total of 8,978 orthogroups were identified, and 6,466 of these contained orthologs in all three species. Of the 8,978 total orthogroups, 7,301 (81%) had at least one gene that showed differential expression in the Δ*wetA* conidia, but only 1,294 orthogroups showed consistent WetA regulation (i.e., all orthologs in the group were either overexpressed or underexpressed).

**FIG 2:**
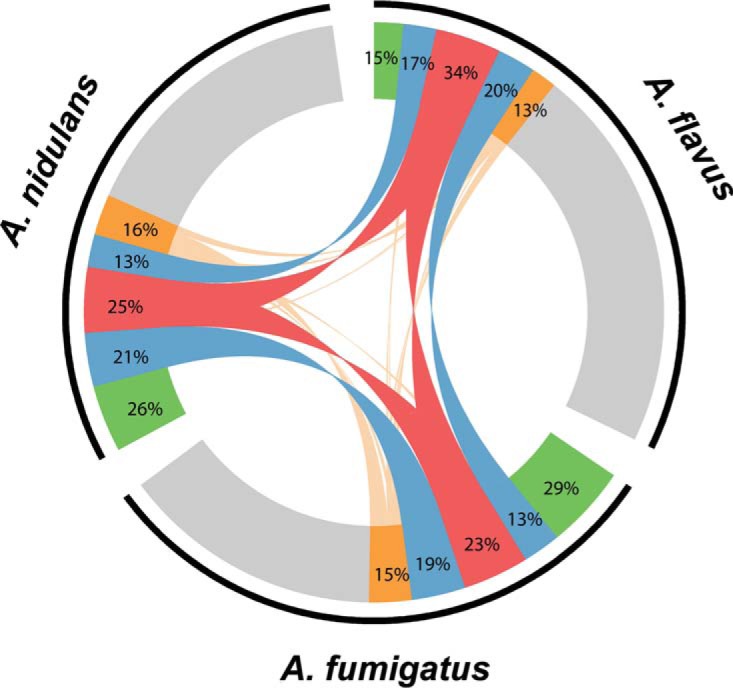
Overview of the WetA-regulated orthologs in A. nidulans, A. fumigatus, and A. flavus. The 20,288 genes belonging to 6,566 orthogroups that possessed at least one member from A. nidulans, A. fumigatus, and A. flavus are represented by the black arcs next to their respective species labels. Gray, orthologs whose expression did not change between Δ*wetA* and WT conidia. Green, orthologs that were differentially expressed in only one species. Blue, genes that showed the same differential expression pattern in two of the three species. Red, genes that showed the same differential expression pattern in all three species. Orange, genes that showed a divergent differential pattern in two or more species. Lines connect expressed genes from the same orthogroup. Percentages represent the fractions of regulated orthologs from that species that belong to each category.

The enriched GO categories of the 1,294 orthogroups whose genes showed the same differential expression pattern suggest that WetA is functionally conserved in controlling stress response, pigmentation, spore trehalose formation, cell wall organization, and cellular development, which are also consistent with the phenotypes observed in Δ*wetA* strains. In contrast, the remaining 6,007 WetA-regulated orthogroups showed divergent differential expression patterns, implying that a substantial portion of the WetA-controlled GRN has functionally diverged among the three species. Furthermore, of the 6,466 *Aspergillus* orthogroups that contain orthologs in all three species, only 788 exhibited a conserved pattern of differential expression (i.e., all genes were either overexpressed or underexpressed in the Δ*wetA* conidia in all three species) (Fig. 2; see also Table S2 in the supplemental material).

10.1128/mBio.01130-18.1 TABLE S1  *Aspergillus* strains and oligonucleotides used in this study. Download TABLE S1, DOCX file, 0.02 MB. Copyright © 2018 Wu et al. 2018 Wu et al. This content is distributed under the terms of the Creative Commons Attribution 4.0 International license.

10.1128/mBio.01130-18.2 TABLE S2  List of conserved WetA-regulated orthogroups. Download TABLE S2, DOCX file, 0.1 MB. Copyright © 2018 Wu et al. 2018 Wu et al. This content is distributed under the terms of the Creative Commons Attribution 4.0 International license.

### WetA-regulated genes involved in asexual development, signal transduction, and conidial integrity are divergently regulated among aspergilli.

**FIG 3:**
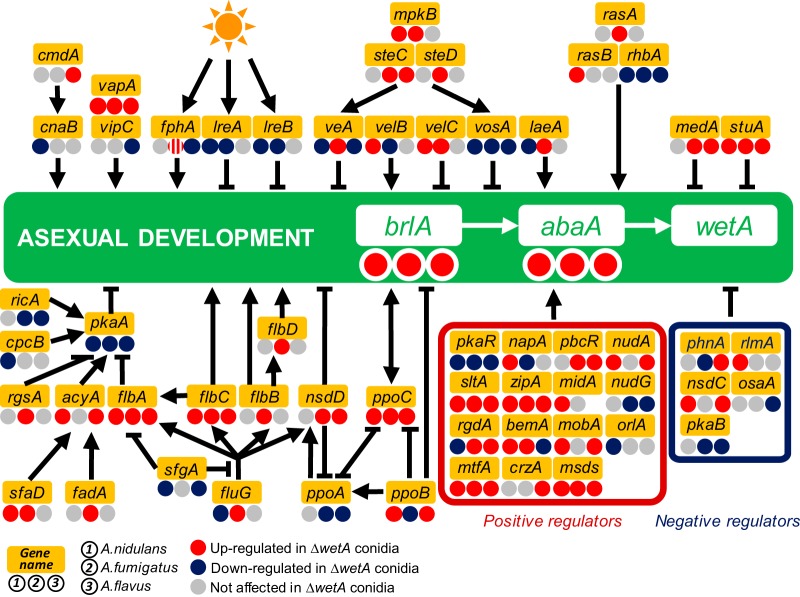
WetA-mediated regulation of asexual development in the three *Aspergillus* species. A schematic diagram of the WetA-mediated regulatory model of conidiation is shown. Genes with increased, decreased, and unaffected mRNA levels in the Δ*wetA* conidia are labeled with red (WetA-inhibited), blue (WetA-activated), and gray (not affected by WetA) circles, and the WetA-regulatory effects in the Δ*AniwetA*, Δ*AfuwetA*, and Δ*AflwetA* conidia are listed under the gene names at the left, middle, and right, respectively. There are two orthologs of *fphA* in A. fumigatus; one is WetA inhibited, and the other is not regulated by WetA.

To explore the conserved and diverged molecular roles of WetA in conidiation in the three species, we examined mRNA levels of genes related to asexual development, signal transduction, and conidial integrity (Fig. 3; see also Table S3), phenotypes previously implicated to be controlled by WetA (8, 10–13, 20–22, 26, 30, 35).

10.1128/mBio.01130-18.3 TABLE S3  DEGs related to asexual development in the Δ*wetA* conidia. Download TABLE S3, DOCX file, 0.04 MB. Copyright © 2018 Wu et al. 2018 Wu et al. This content is distributed under the terms of the Creative Commons Attribution 4.0 International license.

Our data show that WetA negatively regulates asexual development in conidia produced by species across the genus *Aspergillus* via a negative-feedback loop that represses the pathway’s upstream regulator, *brlA*. Specifically, both *brlA* expression and *abaA* expression are increased in the Δ*wetA* conidia relative to the WT in all three species (Fig. 3). However, to achieve the conserved repression of *brlA* and *abaA* mRNA accumulation, WetA regulates *brlA* upstream regulatory networks in a species-specific manner. For example, in the *velvet* protein family and complex, *vosA* was consistently underexpressed in the three Δ*wetA* conidia, but the WetA effects on *veA*, *velB*, *velC*, and *laeA* expression were not conserved in each species. Similarly, the light-dependent regulators were differentially regulated by WetA. The blue-light-dependent regulators *lreA* and *lreB* were unaffected in the A. flavus Δ*wetA* (Δ*AflwetA*) conidia but were repressed in both the A. nidulans Δ*wetA* (Δ*AniwetA*) and A. fumigatus Δ*wetA* (Δ*AfuwetA*) conidia. Taking the data together, the WetA-mediated feedback repression of asexual development is functionally conserved across the genus *Aspergillus* but the specific GRNs appear to have diverged during the evolution of the genus *Aspergillus*.

Our previous study showed that *Afl*WetA is involved in regulating G-protein regulatory pathways (30). Expanding this analysis in the three *Aspergillus* species showed that *gprC*, *gprF*, *gprG*, *nopA*, *flbA*, and *pkaA* were consistently differentially regulated in the Δ*wetA* conidia, while other members in the G-protein regulatory pathways either were not affected by WetA or showed species-specific regulatory patterns in the Δ*wetA* conidia (Table S4).

10.1128/mBio.01130-18.4 TABLE S4  G-protein pathway-related DEGs in the Δ*wetA* conidia in three *Aspergillus* species. Download TABLE S4, DOCX file, 0.02 MB. Copyright © 2018 Wu et al. 2018 Wu et al. This content is distributed under the terms of the Creative Commons Attribution 4.0 International license.

WetA is involved in other signal transduction pathways. A total of 110, 126, and 92 kinase-encoding genes were differentially expressed in the Δ*AniwetA*, Δ*AfuwetA*, and Δ*AflwetA* conidia, respectively; however, only 21 of them were consistently over- or underexpressed in the Δ*wetA* conidia of all three species (Table S5). Similarly, 132, 153, and 142 putative TF-encoding genes in each species were differentially expressed in Δ*AniwetA*, Δ*AfuwetA*, and Δ*AflwetA* conidia, respectively; however, only 32 were consistently over- or underexpressed in the Δ*wetA* conidia of all three species (Table S6).

10.1128/mBio.01130-18.5 TABLE S5  Kinases differentially expressed in the Δ*wetA* conidia. Download TABLE S5, DOCX file, 0.1 MB. Copyright © 2018 Wu et al. 2018 Wu et al. This content is distributed under the terms of the Creative Commons Attribution 4.0 International license.

10.1128/mBio.01130-18.6 TABLE S6  Transcription factors differentially expressed in the Δ*wetA* conidia. Download TABLE S6, DOCX file, 0.1 MB. Copyright © 2018 Wu et al. 2018 Wu et al. This content is distributed under the terms of the Creative Commons Attribution 4.0 International license.

We further investigated the mRNA levels of the genes in the secondary metabolite gene (SMG) clusters in each species (30, 36, 37) (Table S7). In total, 96% (64/67), 100% (33/33), and 92% (68/74) of SMG clusters in the Δ*AniwetA*, Δ*AfuwetA*, and Δ*AflwetA* conidia, respectively, had at least one gene that showed altered mRNA expression levels (Table 3). One of the SMG backbone genes, *wA*, is conserved in all three species, and it encodes a polyketide synthase (PKS) necessary for the formation of a key conidial pigment (38). Previous studies showed that *wA* is activated by WetA (20), consistent with the colorless conidia phenotype of the Δ*wetA* mutants. Although *wA* was underexpressed in the Δ*AniwetA* and Δ*AflwetA* conidia as expected, it was overexpressed in Δ*AfuwetA* conidia, suggesting that the regulation of the conidial pigmentation pathway in A. fumigatus differs from that in the other two species.

10.1128/mBio.01130-18.7 TABLE S7  Secondary metabolite cluster—gene list. Download TABLE S7, DOCX file, 0.03 MB. Copyright © 2018 Wu et al. 2018 Wu et al. This content is distributed under the terms of the Creative Commons Attribution 4.0 International license.

**TABLE 3:** WetA-mediated SMG regulation

Parameter	Result(s)		
	A. nidulans	A. fumigatus	A. flavus
Total cluster no.	67	33	74
No. (%) of clusters with at least one WetA-regulated gene	64 (96)	33 (100)	68 (92)
No. (%) of clusters not regulated by WetA	3 (4): cluster 41, cluster 56, cluster 63	0 (0)	6 (8): cluster 2, cluster 5, cluster 14, cluster 19, cluster 38, cluster 68
No. (%) of clusters where every gene was regulated by WetA	5 (7): emericellamide, terriquinone, cluster 26, cluster 37, cluster 60	8 (24): ferricrocin, DHN melanin, fumigaclavine, endocrocin, helvolic acid, fumisoquin, fumiquinazolines, cluster 31	8 (11): cluster 23, cluster 35, cluster 41, cluster 46, cluster 48, cluster 52, cluster 54, cluster 71
No. (%) of clusters where the whole cluster is up-regulated in Δ*wetA* conidia	2 (3): emericellamide, terriquinone, cluster 26	6 (18): ferricrocin, DHN melanin, endocrocin, helvolic acid, fumisoquin, fumiquinazolines	1 (1): cluster 71
No. (%) of clusters where the whole cluster is down-regulated in Δ*wetA* conidia	0 (0)	1 (3): cluster 31	2 (3): cluster 23, cluster 52

**FIG 4:**
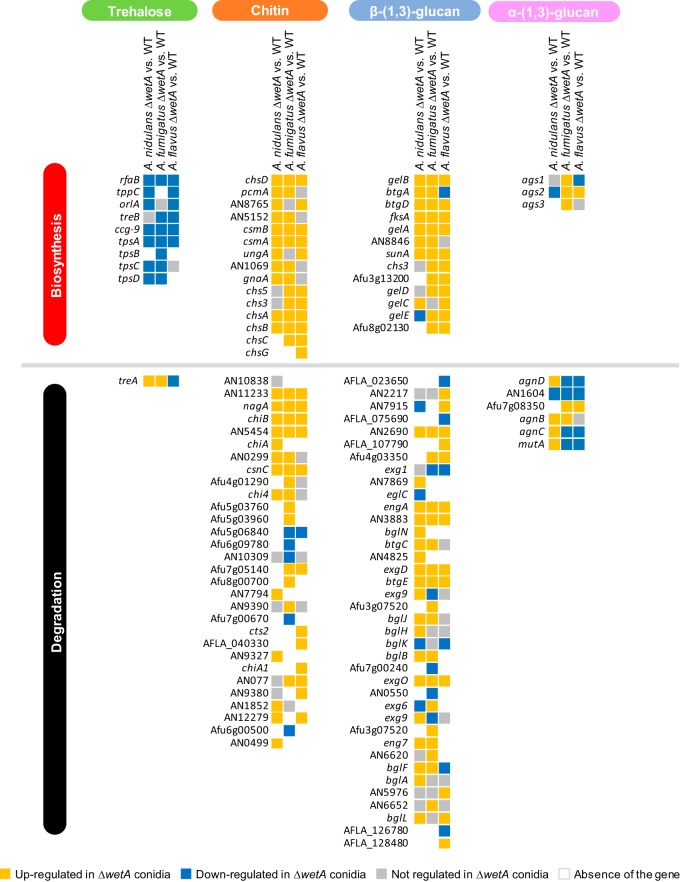
WetA-regulatory effects on trehalose, chitin, β-(1,3)-glucan, and α-(1,3)-glucan metabolism in *Aspergillus* species.

Finally, we examined the expression levels of genes involved in conidial content and conidial wall integrity. Most of the DEGs associated with trehalose biosynthesis were underexpressed in the Δ*wetA* conidia in all three species, while *treA*, involved in trehalose degradation, was overexpressed in the Δ*AniwetA* and Δ*AfuwetA* conidia but underexpressed in the Δ*AflwetA* conidia (Fig. 4). Loss of *wetA* resulted in overexpression of almost all genes involved in the biosynthesis of chitin and β-(1,3)-glucan, but genes involved in the biosynthesis and degradation of α-(1,3)-glucan were both overexpressed and underexpressed relative to the WT (Fig. 4).

**FIG 5:**
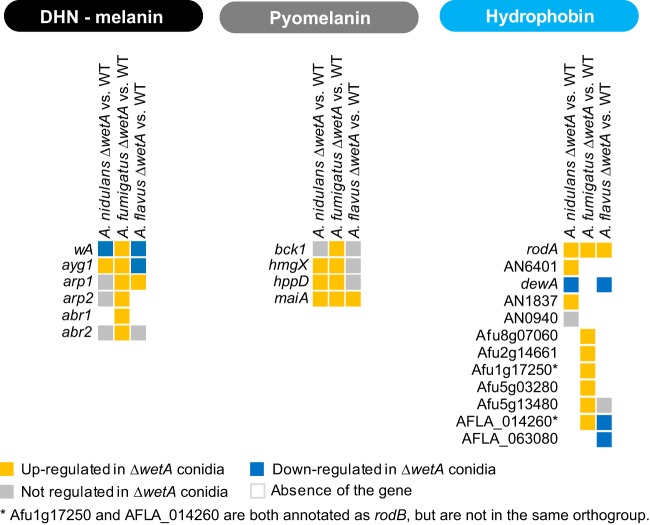
WetA-regulatory effects on DHN-melanin, pyomelanin, and hydrophobin biosynthesis in *Aspergillus* species.

Moreover, our results show that WetA is a key regulator of hydrophobins, 1,8-dihydroxynaphthalene (DHN)-melanin biosynthesis, and pyomelanin biosynthesis. Somewhat unexpectedly, although we observed the conserved “wet” and “white” phenotypes of the Δ*wetA* conidia in all three species, all of the genes proposed to be related to the “wet” (hydrophobin) and “white” (DHN-melanin and pyomelanin) phenotypes were overexpressed in the Δ*AfuwetA* conidia (Fig. 5).

### Identification of WetA response elements (WREs).

To better understand WetA regulatory mechanisms in conidia, we carried out chromatin immunoprecipitation (ChIP) experiments followed by high-throughput sequencing of the enriched DNA fragments (ChIP-seq) in the A. nidulans conidia. We identified 157 peaks from two independent ChIP-seq experiments, using a false-discovery-rate (FDR) (*q* value) cutoff of less than or equal to 0.001 and a fold change (FC; sample tag counts divided by input tag counts) cutoff of greater than or equal to 2. Of the 157 peaks, 135 were located in at least one of the following: a protein coding region, an intron, an upstream region, or a downstream region (Table S8). Upstream and downstream regions were defined as locations within 1.5 kb of the translation start or stop site, respectively. Many peaks were located within multiple features due to the condensed nature of the A. nidulans genome; therefore, 212 genes were considered “peak associated.” Only a few peaks were located within protein coding regions (18) or introns (5); however, 105 peaks were in upstream regions and 59 peaks were in downstream regions. Of the 212 peak-associated genes, 139 showed differential expression in the A. nidulans transcriptome sequencing (RNA-seq) data set. Multiple previously described genes are in the list of peak-associated genes, including *flbA*, *mtfA*, *nopA*, *velB*, *sfaD*, *wetA*, *vosA*, *hsp70*, *srbA*, and *tpsA* (Table S8).

10.1128/mBio.01130-18.8 TABLE S8  A. nidulans WetA ChIP-seq peak-associated genes. Download TABLE S8, DOCX file, 0.1 MB. Copyright © 2018 Wu et al. 2018 Wu et al. This content is distributed under the terms of the Creative Commons Attribution 4.0 International license.

**FIG 6:**
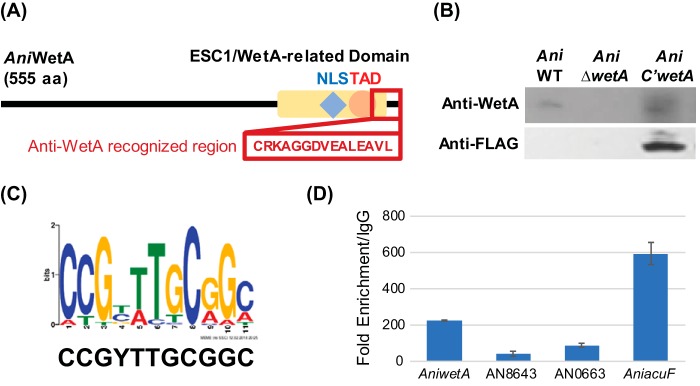
Identification of WetA regulatory element. (A) Diagram of the recognized region of the customized anti-WetA polyclonal antibodies. The recognized anti-WetA region overlaps part of the highly conserved Esc1/WetA-related domain near the WetA C-terminus. (B) Western blot analysis of the crude proteins of conidia from A. nidulans WT, Δ*wetA*, and TMY3 strains using anti-WetA and anti-FLAG polyclonal antibodies. The TMY3 strain expresses WetA::3xFLAG and can be recognized by both anti-WetA and anti-FLAG polyclonal antibodies. The results validated the specificity of the customized anti-WetA polyclonal antibodies. (C) The predicted WetA response element (WRE) and its WebLogo. (D) ChIP-qPCR analysis demonstrates the *Ani*WetA DNA-associating capability of the upstream regions of *AniwetA* (AN1937), AN8643, AN0663, and *AniacuF* (AN1918). Data are presented as fold change compared with rabbit IgG-enriched DNA fragments. Data presented are means ± standard deviations (SD), *n* = 3.

A putative WRE was predicted by MEME-ChIP (39). The 100 bp surrounding the summits of all peaks was used as the input for the MEME-ChIP analysis. The only statistically significant motif identified (E value = 8.8e−8) was 5′-CCGYTTGCGGC-3′, and it exists in the upstream region of *AniwetA* (Fig. 6C). Potential *Ani*WetA-recognized regions were identified by searching for the predicted motif in the upstream regions of open reading frames (ORFs) in the A. nidulans genome with FIMO (40). In total, 2,217 genes were predicted to contain the WRE within their upstream regions in A. nidulans (Table S9). We further carried out ChIP quantitative PCR (ChIP-qPCR) to validate the ability of *Ani*WetA to associate with the WRE and observed a significant enrichment of DNA in our WetA ChIP samples relative to control samples for WRE sequences found upstream of AN8643, AN0663, and *AniacuF* (AN1918) (Fig. 6D). Functional analysis shows that many biological processes were enriched in the large list of potential *Ani*WetA-targeted genes, including the “trehalose metabolic process” and “cell wall mannoprotein biosynthetic process,” consistent with what is known about WetA function in conidiation.

10.1128/mBio.01130-18.9 TABLE S9  Genes containing a WRE in their 1.5-kb upstream regions. Download TABLE S9, DOCX file, 0.4 MB. Copyright © 2018 Wu et al. 2018 Wu et al. This content is distributed under the terms of the Creative Commons Attribution 4.0 International license.

To investigate the expression profile of potential *Ani*WetA target genes in conidia, data from the transcriptomic analysis were utilized. In total, 1,176 WRE-containing genes, including 2 G-protein signaling pathway-associated genes, 22 conidial integrity-associated genes, 14 putative kinase-encoding genes, 22 putative transcription factor-encoding genes, 5 SMG backbone genes, and 11 asexual development-associated genes, were differentially expressed in A. nidulans (Table 4).

**TABLE 4:** WetA-targeted DEGs in Δ*AniwetA* conidia

DEG function or product	DEGs
G-protein pathway	*gprC*, *nopA*
Conidial integrity	*tppC*, *ccg-9*, *tpsC*, *treA*, *chsA*, *chiA*, *btgD*, *eglC*, *engA*, *btgC*, *eng7*, *bglA*, *ags2*, *agnD*, *agnC*, *hmgX*, *hppD*, *maiA*, *dewA*, AN0499, AN1069, AN1837
Kinase	*rio2*, *aromA*, *nimO*, *isr1*, *teaR*, *pho80*, *ffkA*, *panK*, *nimX*, AN3619, AN8213, AN8843, AN10188, AN10551
TF	*fcr1*, *aflR*, *dbaA*, *cpcA*, *vosA*, *mdpE*, *wetA*, *zapA*, AN6295, AN1217, AN0817, AN3502, AN3769, AN0094, AN4773, AN6790, AN8111, AN8355, AN8949, AN11169, AN0388, AN10550
SMG backbone(s)	*apdA*, *inpB*, AN0016, AN1242, AN9129
Asexual development	*ams1*, *chsB*, *cnaB*, *cpcB*, *dewA*, *gprC*, *odeA*, *tpsC*, *velB*, *wetA*, *wsc1*

**FIG 7:**
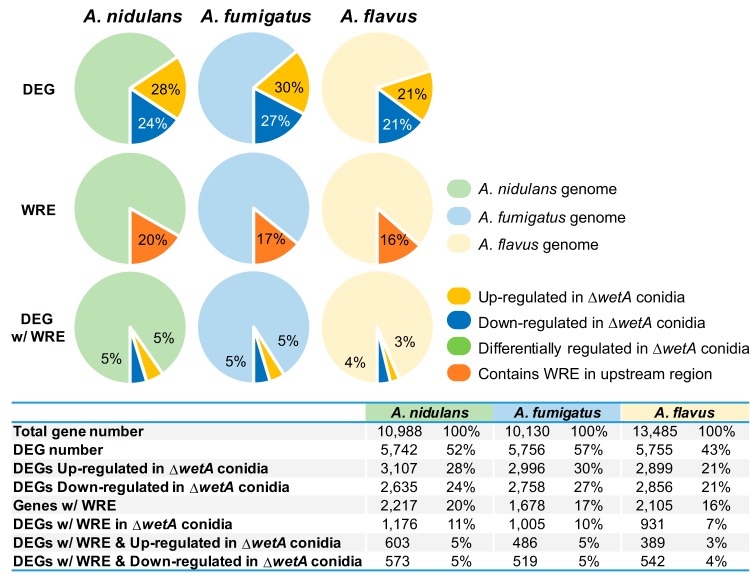
Overlap between DEGs and WRE-containing genes in three *Aspergillus* species. The percentages of genes differentially expressed in the Δ*wetA* conidia (DEG), the percentages of genes that contain predicted WRE sequences in their upstream 1.5-kb regions (WRE), and the DEGs with a WRE in their upstream 1.5-kb regions (DEG w/WRE) are shown. The A. nidulans, A. fumigatus, and A. flavus genes are shown in light green, light blue, and light orange, respectively.

**FIG 8:**
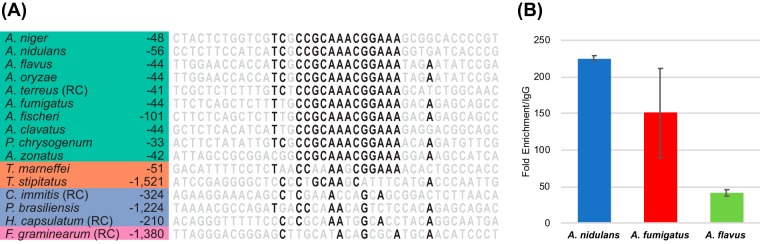
WRE occurrences in the upstream regions of *wetA* orthologs in representative fungi. WRE occurrences were identified in a series of regions located upstream of *wetA* orthologs. (A) Numbers to the left of the sequence indicate at what position relative to the translation start site the sequence shown begins. The sequences shown are from 15 bp upstream of the WRE occurrence that was identified by FIMO (40) with the lowest *P* value to 14 bp downstream of the WRE occurrence. Bases are colored black if they are conserved in at least 60% of the species. Green—Aspergillaceae. Orange—Trichocomaceae. Blue—Onygenales. Purple—Sodariomycete. (RC)—reverse complement. (B) ChIP-qPCR analysis demonstrated that WetA occupies *wetA* promoters in A. nidulans, A. fumigatus, and A. flavus. Data are presented as fold change compared with rabbit IgG-enriched DNA fragments. Data presented are means ± SD, *n* = 3.

Since the *Aspergillus* WetA proteins have a highly conserved potential DNA-binding domain and have conserved functions in the overall conidiation process, we hypothesized that *Afu*WetA and *Afl*WetA associate with a sequence similar to the *Ani*WetA WRE. We therefore searched for the *Ani*WetA WRE in the A. fumigatus and A. flavus genomes and summarized the results in Fig. 7. Only 15 genes, including *wetA*, that contained a WRE in their upstream 1.5-kb regions in all three species also exhibited consistent differential expression in our RNAseq data (Table 5). To test if WetA from species other than A. nidulans associated with the WRE, we carried out ChIP-qPCR experiments in A. fumigatus and A. flavus that examined the enrichment of WetA at the WRE found upstream of the *wetA* genes in those species. The ChIP-qPCR result demonstrated that WetA recognized the WRE in the *wetA* upstream regions in A. nidulans, A. fumigatus, and A. flavus (Fig. 8B), suggesting that the WRE is conserved and potentially functional in the three *Aspergillus* species. We further searched for WRE occurrences in the 1.5-kb sequence upstream of other *wetA* orthologs in different *Aspergillus* species and other fungal species and found that the WRE in the upstream region of *wetA* genes is completely conserved throughout the family Aspergillaceae (Fig. 8A).

**TABLE 5:** Conserved DEGs with a WRE in aspergilli

DEG category	DEG ID^a^		
	A. nidulans	A. flavus	A. fumigatus
Up-regulated in Δ*wetA* conidia	AN1156	AFLA_068310	Afu1g11450
	AN10598	AFLA_101220	Afu3g07020
	AN6088	AFLA_045760	Afu2g09282
	AN4836	AFLA_102180	Afu3g07290
	AN3752	AFLA_073850	Afu7g04580
			
Down-regulated in Δ*wetA* conidia	AN4464	AFLA_112150	Afu4g07690
	AN1524	AFLA_078640	Afu8g05330
	AN5715	AFLA_127800	Afu1g06770
	AN10265	AFLA_014960	Afu2g15910
	AN10551	AFLA_112710	Afu4g07140
	AN5215	AFLA_087840	Afu6g07490
	AN8763	AFLA_131750	Afu6g02960
	AN9037	AFLA_023560	Afu3g15190
	AN4716	AFLA_091920	Afu5g08580
	AN1937	AFLA_052030	Afu4g13230

a ID, identifier.

Taking the results together, we conclude that WetA regulates the *Aspergillus* conidial transcriptomes through both direct and indirect methods and controls species-specific GRNs to achieve conserved and diverged functions.

## DISCUSSION

While WetA is well known as the key regulator of multiple cellular and chemical developmental processes in Ascomycetes (7–23,30), the regulatory mechanisms that it employs are not known. In this study, we investigated the roles of WetA-mediated GRNs in the model organism A. nidulans, the human pathogen A. fumigatus, and the aflatoxin producer A. flavus and further identified a potential WetA binding motif in A. nidulans.

Previous studies suggested that *Ani*WetA is required for activating a set of genes whose products comprise, or direct the assembly of, the conidial wall layers and also ensure proper cytoplasmic metabolic remodeling, including massive trehalose biogenesis (20, 22, 41). We also reported that *Afl*WetA is involved in the regulation of conidial secondary metabolism and hypothesized that this was done by WetA controlling a group of TFs and signaling pathways (30). Our RNA-seq results reported here show that 52%, 57%, and 43% of A. nidulans, A. fumigatus, and A. flavus transcriptomes were differentially regulated in the Δ*wetA* conidia, respectively, suggesting a broad regulatory role for WetA in aspergilli (Table 2). While *Ani*WetA, *Afu*WetA, and *Afu*WetA are functionally conserved in many aspects of developmental processes in conidia, the specific genes regulated by WetA are divergent in each species. Although WetA regulates a large number of common orthogroups in aspergilli, only 9% of *Aspergillus* orthogroups were consistently all over- or underexpressed in Δ*wetA* conidia from the three species, suggesting that while the WetA-mediated regulation is functionally conserved, the WetA-mediated GRNs have been highly rewired (Fig. 2).

An example of a divergent WetA-mediated GRN whose output is the conserved regulation of a biological process is the GRN associated with *Aspergillus* asexual development (Fig. 3). In all three species, loss of *wetA* leads to increased levels of the central regulator *brlA* in conidia and shuts down asexual development. However, the set of regulatory events that result in WetA-mediated repression of *brlA* is unique to each species (Fig. 3). In addition, our ChIP-seq data did not show an enrichment of *wetA* near *brlA* in A. nidulans, nor does *brlA* have a WRE in its upstream region in A. nidulans, A. fumigatus, or A. flavus. We hypothesize that the conserved repression of *brlA* by WetA is carried out through diverged, indirect methods in each of the three species.

We further examined the WetA-mediated GRNs controlling other pathways on the basis of previously characterized, conserved WetA functions. First, we analyzed genes involved in conidial integrity for their WetA regulation. The genes associated with trehalose biosynthesis are almost all underexpressed in the Δ*wetA* conidia of all three species (Fig. 4). Similarly, almost all the genes associated with β-(1,3)-glucan biosynthesis were overexpressed in the Δ*wetA* conidia of all three species (Fig. 4). These results explain the dramatically reduced amount of trehalose and increased content of β-(1,3)-glucan in the Δ*wetA* conidia (8, 30) and suggest the presence of a conserved WetA-mediated GRN for activation of trehalose biogenesis and repression of β-(1,3)-glucan biosynthesis. WetA’s function is likely divergent in α-(1,3)-glucan metabolism. *Ani*WetA upregulates the α-(1,3)-glucan synthase *Aniags2* but downregulates all the genes associated with α-(1,3)-glucan degradation except AN1604 (Fig. 4). In contrast, *Afu*WetA downregulates all the α-(1,3)-glucan synthases (*Afuags1*, *Afuags2*, and *Afuags3*) but has mixed effects on the genes associated with α-(1,3)-glucan degradation in conidia (Fig. 4). In conidia, *Afl*WetA shows mixed effects on both the genes associated with α-(1,3)-glucan biosynthesis and those associated with degradation (Fig. 4).

WetA is involved in the regulation of hydrophobin expression. Only one of the five hydrophobin-encoding genes in A. nidulans was not differentially expressed in the Δ*wetA* conidia, and only *AnidewA* was underexpressed (Fig. 5). In A. fumigatus, all six hydrophobin-encoding genes were overexpressed in the Δ*wetA* conidia (Fig. 5). In A. flavus, three of five hydrophobin-encoding genes were underexpressed in the Δ*wetA* conidia, one of the five was not regulated, and only *AflrodA* was upregulated (Fig. 5). Since the loss of *wetA* causes lower hydrophobicity of conidia, there might be other unidentified hydrophobins controlled by *Afu*WetA. In addition, the hydrophobins in A. fumigatus could be regulated post-transcriptionally or could be expressed and translated earlier in development, at a time point before that at which the RNA was isolated from conidia for our experiments.

*Afu*WetA diverges from *Ani*WetA and *Afl*WetA in its regulation of melanin biosynthesis. A previous study showed that *wA*, the first regulator in the DHN-melanin synthesis pathway, is activated by WetA in A. nidulans conidia (20, 42). Our RNA-seq analyses have revealed that both *AniwA* and *AflwA* were underexpressed in the Δ*AniwetA* and Δ*AflwetA* conidia (Fig. 5). Moreover, *Aflayg1*, the second gene in the DHN-melanin pathway (43), was underexpressed in the Δ*AflwetA* conidia (Fig. 5). Surprisingly, although the Δ*AfuwetA* conidia are colorless, all the DEGs associated with both DHN-melanin and pyomelanin biosynthesis were overexpressed in the Δ*AflwetA* conidia (Fig. 5), suggesting the melanin biosynthesis pathway in A. fumigatus may have uniquely evolved.

Our ChIP-seq experiments performed with an antibody native to WetA identified a potential WetA response element (5′-CCGYTTGCGGC-3′) (Fig. 6C) which is recognized by WetA in A. nidulans, A. fumigatus, and A. flavus (Fig. 8B) and is highly similar to the Saccharomyces cerevisiae Ixr1, Dal81, and Leu3 DNA-binding motifs (44–46). The list of peak-associated genes in A. nidulans includes *wetA* and the important developmental regulators *vosA* and *velB*, suggesting that these genes may play crucial roles in conidiation and thus may be conserved during evolution. VosA and VelB are both members of the *velvet* family of proteins (47–49). Moreover, the VosA-VelB complex is a crucial functional unit controlling maturation of conidia (48–50). Loss of *vosA* causes some phenotypes similar to those caused by the loss of *wetA*, such as a reduction in the trehalose amount (51), suggesting that part of the WetA-mediated GRN may be controlled by regulating VosA. Previous studies showed that *Ani*WetA contains an *Ani*VosA binding motif in its upstream 2-kb region (47), implicating the cross feedback regulation of WetA by VosA. Taking the data together, the WetA-mediated regulatory pathway may cross talk with the *velvet* regulatory pathways via the cooperative activity of WetA/VosA/VelB.

Although 52% of genes in the A. nidulans genome were differentially regulated in the Δ*AniwetA* conidia, only 20% of those regulated genes contain a WRE in their upstream 1.5-kb regions (Fig. 7), suggesting a model where WetA-mediated regulation is carried out via both direct and indirect interactions to control a downstream cascade of genes. We also scanned the A. fumigatus and A. flavus genomes for instances of the WRE and found that, while the numbers of genes that contained the WRE were similar to the numbers seen with A. nidulans, those lists of genes were different in content (Fig. 7). Only 15 genes with the WRE in their upstream regions were consistently under- or overexpressed in the Δ*wetA* conidia from all three species. Our data suggest that WetA serves as a conserved regulatory hub that controls a diverse group of species-specific regulators and effectors in *Aspergillus* species.

In conclusion, our studies provided the first clear and systematic dissection of WetA, an evolutionarily and functionally conserved regulator of morphological and chemical development of filamentous fungal conidiation. Moreover, we have revealed the molecular mechanisms of WetA as a likely DNA-binding, multifunctional regulator governing the diverse processes of cellular differentiation, chemical development, and cell survival across a genus of filamentous fungi, advancing our knowledge of spore formation in pathogenic and toxigenic fungi.

## MATERIALS AND METHODS

### Strains, media, and culture conditions.

All strains used in this study are listed in Table S1 in the supplemental material. The fungal strains were grown on minimal medium (MM) with appropriate supplements as previously described (51, 52) and were incubated at 37°C (A. nidulans and A. fumigatus) or 30°C (A. flavus). For liquid cultures, conidia were inoculated in liquid MM and incubated at 37°C or 30°C and 220 rpm.

### Generation of *wetA* deletion and complementation strains.

We generated the deletion (Δ) and complementation (complement) strains of *wetA* in A. nidulans (*AniwetA*). The oligonucleotides used in this study are listed in Table S1. Briefly, the deletion construct containing the A. fumigatus *pyrG* marker with 5′ and 3′ flanking regions of *AniwetA* was introduced into recipient strain RJMP1.59 (53). To generate complemented strains, a WT *AniwetA* gene region, including its 2-kb upstream region, was cloned to pHS13 (48). The resulting pMY1 plasmid was then introduced into recipient Δ*AniwetA* strain TMY3, resulting in isolation of TMY4. Multiple Δ*AniwetA* strains were generated, and all behaved the same in every assay. Multiple complement *AniwetA* strains were generated, and all behaved identically to one another as well. The Δ*AfuwetA* (TSGw4), Δ*AflwetA* (TMY1), and complement *AflwetA* (TMY2) strains were generated in previous studies (8, 30).

### Nucleic acid manipulation.

The isolation of genomic DNA and total RNA for Northern blot analyses was performed as previously described (54–56). For RNA-seq and ChIP-seq, conidia of WT and Δ*wetA* strains were grown on solid culture at 37°C or 30°C for 2 days because the number of intact Δ*wetA* spores at 2 days was similar to the number of wild-type spores as we reported before (30), and on the third day the Δ*wetA* conidia began to autolyze. Fresh conidia were harvested from these 2-day-old cultures and filtrated by the use of four-layer Miracloth.

### RNA sequencing.

Total RNA from four A. nidulans biological replicates, three A. flavus biological replicates, and three A. fumigatus biological replicates was extracted and submitted to ProteinCT Biotechnologies (Madison, WI) and the University of Wisconsin Gene Expression Center (Madison, WI) for library preparation and sequencing. For each replicate, a strand-specific library was prepared from total RNA using a Illumina TruSeq strand-specific RNA sample preparation system. The libraries of all the replicates were sequenced (PE100bp for A. nidulans and SE100bp for A. fumigatus and A. flavus) using an Illumina HiSeq 2500 system.

The A. flavus expression data were analyzed as previously reported (30). The following analyses were carried out for the A. fumigatus and A. nidulans data. The overall quality of the raw sequence reads was verified using version 0.11.5 of FastQC (57). The genomes and annotations were downloaded from FungiDB and used for mapping (58). Mapping of the raw sequence reads to the genome was carried out with version 2.1.1 of Tophat2 (59), and the default settings were used except that the maximum intron length was set to 4,000 bases. The alignment files were compared to the gene annotation file, and raw counts for the number of reads mapping to each gene were generated using version 0.6.1p1 of HTSeq-count (60). Differential expression analysis of the raw counts was carried out using version 1.14.1 of DESeq2 (61). Genes were considered differentially expressed between the WT and Δ*wetA* conidia if their adjusted *P* value was less than 0.05 and their log2-fold change value was lower than −1 or higher than 1. All RNA-seq data files are available from the NCBI Gene Expression Omnibus database (A. nidulans and A. fumigatus, GSE114143; A. flavus, GSE95711).

### Functional enrichment and orthogroup identification.

Gene Ontology enrichment analyses were carried out using the tool available at FungiDB (58). Unless otherwise stated, default settings were used in FungiDB, and redundant terms were collapsed with the REVIGO tool (62) using the “Tiny” setting for allowed similarity.

Orthologs were identified using OrthoMCL with the following settings: *P* value cutoff of 6e−6, identity cutoff of 30%, match cutoff of 70%, Markov cluster algorithm (MCL) inflation value of 2, and allowed maximum weight of 180.

### Anti-WetA polyclonal antibody synthesis.

Multiple-sequence alignment using Clustal W/X (46) revealed that *Ani*WetA, *Afu*WetA, and *Afl*WetA contain a 15-amino-acid (aa)-length conserved region near their C-termini (Fig. 6A and B). The 15-aa CRKAGGDVEALEAVL peptide was selected, synthesized, and used for rabbit immunization and generation of an affinity-purified polyclonal antibody (GenScript Corp., Piscataway, NJ).

### Chromatin immunoprecipitation sequencing (ChIP-seq) and chromatin immunoprecipitation quantitative PCR (ChIP-qPCR).

ChIP analyses were performed using MAGnify ChIP assays (Invitrogen) according to the manufacturer’s instructions. Briefly, 10^9^ of A. nidulans WT conidia were cross-linked with 1% formaldehyde, lysed, and broken as previously described (63). Cell lysates were sonicated to shear DNA to 300 to 500 bp and were immunoprecipitated with customized rabbit anti-WetA polyclonal antibodies (GenScript, NJ). Two experiments were performed, each with biological triplicates. In the first experiment, 10% of the supernatants was kept as an input control (input represents PCR amplification of the total sample) and compared to the ChIP sample. In the second experiment, the ChIP sample from the WT strain was compared to the ChIP sample from the Δ*wetA* strain. ChIP DNA samples were sent for ChIP-seq service (ProteinCT, WI). Libraries were prepared using a TruSeq ChIP library preparation kit (Illumina, CA) and sequenced on a HiSeq 2500 system with single reads of 50 bp. Approximately 8 to 30 M reads were achieved per replicate.

ChIP-seq reads were first trimmed using version 0.36 of the Trimmomatic software (64), and then version 0.7.15 of the BWA-MEM software (65) was used to map reads to the A. nidulans (FGSC A4) genome. Reads with any of the following flags were removed: unmapped, secondary alignment, or supplementary mapped read. Reads with a mapping quality (MAPQ) score of 0 were also removed. Duplicate reads were removed and samples were pooled using version 1.3 of the SAMtools software (66). Version 2.1.1.20160309 of the MACS2 software (67) with the settings -g 2.93e7 –s 101—nomodel—extsize was used to call peaks. Extension sizes were calculated using SPP (68, 69). Peaks that exhibited a fold change value greater than 2 and a *q*-value of less than 0.001 were used in further analyses. Peak lists from each of the ChIP experiments were combined. The ChIP-seq data are available from the NCBI Gene Expression Omnibus database (GSE114141).

ChIP-qPCR was performed with iTaq universal Sybr green supermix (Bio-Rad, Hercules, CA) on a Bio-Rad CFX96 real-time PCR detection system.

### Motif discovery analyses.

To discover the WetA response element (WRE), 100 bp of sequence surrounding the summits of the 157 combined peaks were pulled from the A. nidulans genome using bedtools software, version 2.26.0 (70), and submitted to processing using MEME-ChIP software, version 4.12.0 (39).

MEME was instructed to search for 10 motifs, 5 to 21 bp in length; all other settings were left at the defaults. Instances of the WRE were identified in the upstream regions (1.5 kb upstream of the translation start) of all genes in the three *Aspergillus* genomes using FIMO software (40) with a *P* value cutoff of 5e−5.

### Data availability.

All RNA-seq and ChIP-seq data files are available from the NCBI Gene Expression Omnibus database (A. nidulans and A. fumigatus RNA-seq, GSE114143; A. flavus RNA-seq, GSE95711; A. nidulans ChIP-seq, GSE114141).
