# Supplementary material for: Systematic Dissection of the Evolutionarily Conserved WetA Developmental Regulator across a Genus of Filamentous Fungi
Source: mBio. 2018 Aug 21;9(4):e01130-18. doi: 10.1128/mBio.01130-18 (PMC6106085; doi:10.1128/mBio.01130-18)
Supplement: TABLE S1 [file mbo004184026st1.docx]

Table S1 *Aspergillus* strains and oligonucleotides used in this study.

| STRAINS | | | |  |
| --- | --- | --- | --- | --- |
| Name | Genotype | | Source |  |
| NRRL3357 | Wild-type | | Fungal Genetic Stock Center |  |
| 3357.5 | *pyrG^−^* | | (1) |  |
| TMY1 | Δ*AflwetA::AfupyrG^+^*; *pyrG^−^* | | (2) |  |
| TMY2 | Δ*AflwetA::AfupyrG^+^*; *pyrG^−^; wetA* | | (2) |  |
| Af293 | Wild-type | | (3) |  |
| TSGw4 | *AfupyrG1* Δ*AfuwetA*::*AnipyrG^+^* | | (4) |  |
| FGSC4 | Wild-type | | Fungal Genetic Stock Center |  |
| RJMP1.59 | *pyrG89*; *pyroA4* | | (5) |  |
| TMY3 | *pyrG89*; *pyroA4*; Δ*AniwetA::AfupyrG^+^* | | This study |  |
| TMY4 | *pyrG89*; *pyroA*::*AniwetA*(p)::*AniwetA*::FLAG_3X_::*pyroA^*^*; Δ*AniwetA::AfupyrG^+^* | | This study |  |
| ^*^The 3/4 *pyroA* marker selects for the targeted integration at the *pyroA* locus. | | | |  |
| OLIGONUCLEOTIDES | | | | |
| Name | **Sequence (5’🡪3’)** | **Purpose** | | |
| oMY-43 | tagcgcattgttgcttaggg | 5’ flanking of *AniwetA* | | |
| oMY-44 | gccgttaccgacggatactc | 3’ flanking of *AniwetA* | | |
| oMY-45 | gtaatagactcagtggaccgggc | 5’ nested of *AniwetA* | | |
| oMY-46 | ctcctcctagaacccattatggc | 3’ nested of *AniwetA* | | |
| oMY-47 | gtgaagagcattgtttgaggcaggaagaggctgccagaagacctg | 5' *AniwetA* with *AfupyrG* tail | | |
| oMY-48 | agtgcctcctctcagacagaataggaggaagcttagatctgtggc | 3' *AniwetA* with *AfupyrG* tail | | |
| oMY-25 | gaccactcgttcaacaacgatg | 5’ *AniwetA* | | |
| oMY-26 | cgtactgcattaagtgcgg | 3’ *AniwetA* | | |
| oMY-53 | ccgaattcttgaagtattgattatgtaattatgc | 5’ *AniwetA* with *Eco*RI | | |
| oMY-54 | tagcggccgcgcagaggacagcctctaggg | 3’ *AniwetA* with *Not*I | | |
| oJH-84 | gctgaagtcatgatacaggccaaa | 5’ *AfupyrG* marker | | |
| oJH-85 | atcgtcgggaggtattgtcgtcac | 3’ *AfupyrG* marker | | |
| omy-242 | ccgctggttcaggtcttctg | 5’ *AniwetA* upstream WRE (100 bp) | | |
| omy-243 | catcttgccagcgggtga | 3’ *AniwetA* upstream WRE (100 bp) | | |
| OMY-268 | aagcctagtgtacgcttacaagg | 5’ AN8643 upstream WRE (100 bp) | | |
| OMY-269 | ctgcatccgccaatcatgg | 3’ AN8643 upstream WRE (100 bp) | | |
| OMY-270 | ctgcggatctcgtttccgtc | 5’ AN0663 upstream WRE (100 bp) | | |
| OMY-271 | ctgccctctctacaccaccaatc | 3’ AN0663 upstream WRE (100 bp) | | |
| OMY-272 | cgaatttgcggggatagg | 5’ AN1918 upstream WRE (100 bp) | | |
| OMY-273 | gcaagtcttgtgggaatgtactcag | 3’ AN1918 upstream WRE (100 bp) | | |
| omy-260 | gccctgggagtagagaccag | 5’ *AniwetA* upstream WRE (200 bp) | | |
| omy-261 | tcggtactcgatgtgtccat | 3’ *AniwetA* upstream WRE (200 bp) | | |
| omy-262 | gacacccgaactctcttcgag | 5’ *AfuwetA* upstream WRE (200 bp) | | |
| omy-263 | tgagtccatgtcgacgtactg | 3’ *AfuwetA* upstream WRE (200 bp) | | |
| omy-264 | gatccgcagttcccttcgac | 5’ *AflwetA* upstream WRE (200 bp) | | |
| omy-265 | gacgtactgactgaataaatcatcg | 3’ *AflwetA* upstream WRE (200 bp) | | |

1. He Z-M, Price MS, Obrian GR, Georgianna DR, Payne GA. 2007. BMC Microbiol 7:104.

2. Wu M-Y, Mead ME, Kim S-C, Rokas A, Yu J-H. 2017. PLoS One 12:e0179571.

3. Brookman JL, Denning DW. 2000. Curr Opin Microbiol 3:468–474.

4. Tao L, Yu J-H. 2011. Microbiology 157:313–326.

5. Shaaban MI, Bok JW, Lauer C, Keller NP. 2010. Eukaryot Cell 9:1816–1824.
