# Supplementary material for: Systematic Dissection of the Evolutionarily Conserved WetA Developmental Regulator across a Genus of Filamentous Fungi
Source: mBio. 2018 Aug 21;9(4):e01130-18. doi: 10.1128/mBio.01130-18 (PMC6106085; doi:10.1128/mBio.01130-18)
Supplement: TABLE S2 [file mbo004184026st2.docx]

Table S2 List of conserved WetA-regulated orthogroups

| **Expression pattern in the Δ*wetA* conidia** | ***A. nidulans*** | ***A. fumigatus*** | ***A. flavus*** |
| --- | --- | --- | --- |
| All upregulated in the Δ*wetA* conidia | AN1427 | Afu1g00440 Afu8g04110 Afu8g06560 | AFLA_012030 |
|  | AN2018 AN3402 | Afu2g00710 Afu4g10130 | AFLA_026140 |
|  | AN10062 AN5559 | Afu4g00570 Afu4g11780 | AFLA_027800 |
|  | AN6470 AN8969 | Afu6g10130 Afu8g06980 | AFLA_104880 |
|  | AN12126 AN9187 | Afu4g09270 | AFLA_003770 |
|  | AN1428 | Afu1g00450 Afu8g04100 | AFLA_012050 |
|  | AN1418 | Afu1g00480 Afu8g04070 | AFLA_012080 |
|  | AN9205 | Afu5g00100 Afu7g06523 | AFLA_038870 |
|  | AN11043 | Afu3g01610 Afu4g09300 | AFLA_110180 |
|  | AN4385 | Afu4g06750 | AFLA_113190 AFLA_113200 |
|  | AN9305 | Afu3g14730 Afu7g00200 | AFLA_117500 |
|  | AN0233 | Afu6g14500 | AFLA_000820 |
|  | AN6472 | Afu2g00680 | AFLA_001640 |
|  | AN7230 | Afu2g17620 | AFLA_001890 |
|  | AN7334 | Afu2g16520 | AFLA_003220 |
|  | AN7352 | Afu2g16390 | AFLA_003460 |
|  | AN12489 | Afu2g16230 | AFLA_003630 |
|  | AN2133 | Afu2g16200 | AFLA_003650 |
|  | AN2483 | Afu4g03060 | AFLA_003660 |
|  | AN3883 | Afu5g02280 | AFLA_004480 |
|  | AN7823 | Afu5g02300 | AFLA_004510 |
|  | AN8003 | Afu5g02600 | AFLA_004830 |
|  | AN8004 | Afu5g02610 | AFLA_004870 |
|  | AN8163 | Afu5g02870 | AFLA_005570 |
|  | AN8164 | Afu5g02880 | AFLA_005580 |
|  | AN8167 | Afu5g02950 | AFLA_005660 |
|  | AN8175 | Afu5g03010 | AFLA_005720 |
|  | AN8181 | Afu5g03060 | AFLA_005790 |
|  | AN8182 | Afu5g03080 | AFLA_005810 |
|  | AN8233 | Afu5g03690 | AFLA_006520 |
|  | AN9521 | Afu5g03750 | AFLA_006580 |
|  | AN8242 | Afu5g03770 | AFLA_006610 |
|  | AN11070 | Afu5g03990 | AFLA_006750 |
|  | AN8278 | Afu5g04255 | AFLA_007050 |
|  | AN5330 | Afu6g03350 | AFLA_007620 |
|  | AN1818 | Afu4g09480 | AFLA_008110 |
|  | AN5343 | Afu6g14220 | AFLA_008290 |
|  | AN10512 | Afu6g14200 | AFLA_008310 |
|  | AN5442 | Afu6g13540 | AFLA_008990 |
|  | AN7378 | Afu4g14205 | AFLA_011370 |
|  | AN8546 | Afu3g01530 | AFLA_011560 |
|  | AN1437 | Afu8g04300 | AFLA_011790 |
|  | AN2145 | Afu2g16060 | AFLA_014740 |
|  | AN2161 | Afu2g15900 | AFLA_014980 |
|  | AN7583 | Afu2g15170 | AFLA_015680 |
|  | AN7563 | Afu2g14920 | AFLA_016700 |
|  | AN0736 | Afu1g14230 | AFLA_016980 |
|  | AN10113 | Afu1g13680 | AFLA_017740 |
|  | AN0698 | Afu1g13610 | AFLA_017800 |
|  | AN0674 | Afu1g13340 | AFLA_018080 |
|  | AN1014 | Afu1g12910 | AFLA_018560 |
|  | AN2498 | Afu3g14090 | AFLA_019010 |
|  | AN2502 | Afu3g14130 | AFLA_019040 |
|  | AN2505 | Afu3g14150 | AFLA_019100 |
|  | AN2503 | Afu3g14140 | AFLA_019130 |
|  | AN2493 | Afu3g14030 | AFLA_019800 |
|  | AN2492 | Afu3g14020 | AFLA_019810 |
|  | AN10356 | Afu3g13080 | AFLA_020720 |
|  | AN3132 | Afu3g13010 | AFLA_020780 |
|  | AN3125 | Afu3g12830 | AFLA_020860 |
|  | AN1292 | Afu1g09730 | AFLA_021190 |
|  | AN1298 | Afu1g09670 | AFLA_021240 |
|  | AN10181 | Afu1g09530 | AFLA_021630 |
|  | AN5167 | Afu6g06990 | AFLA_021950 |
|  | AN5152 | Afu1g07110 | AFLA_022110 |
|  | AN5187 | Afu1g07170 | AFLA_022160 |
|  | AN5145 | Afu1g07210 | AFLA_022210 |
|  | AN5131 | Afu1g07470 | AFLA_022400 |
|  | AN5104 | Afu1g07700 | AFLA_022650 |
|  | AN5099 | Afu1g07750 | AFLA_022700 |
|  | AN11680 | Afu4g00390 | AFLA_024260 |
|  | AN3222 | Afu4g00970 | AFLA_024490 |
|  | AN8074 | Afu5g01750 | AFLA_024860 |
|  | AN8927 | Afu5g01120 | AFLA_025370 |
|  | AN1828 | Afu4g09590 | AFLA_025500 |
|  | AN1846 | Afu4g09670 | AFLA_025630 |
|  | AN1847 | Afu4g09700 | AFLA_025650 |
|  | AN1855 | Afu4g09890 | AFLA_025880 |
|  | AN2045 | Afu4g10070 | AFLA_026040 |
|  | AN2044 | Afu4g10080 | AFLA_026050 |
|  | AN2043 | Afu4g10090 | AFLA_026060 |
|  | AN2013 | Afu4g10190 | AFLA_026200 |
|  | AN2009 | Afu4g10220 | AFLA_026250 |
|  | AN5646 | Afu4g10950 | AFLA_026950 |
|  | AN5619 | Afu4g11150 | AFLA_027140 |
|  | AN5611 | Afu4g11250 | AFLA_027240 |
|  | AN5583 | Afu4g11480 | AFLA_027460 |
|  | AN5586 | Afu4g11510 | AFLA_027500 |
|  | AN5558 | Afu4g11800 | AFLA_027810 |
|  | AN4049 | Afu1g03630 | AFLA_028340 |
|  | AN4019 | Afu1g03730 | AFLA_028460 |
|  | AN0472 | Afu1g04260 | AFLA_028950 |
|  | AN0453 | Afu1g04750 | AFLA_029150 |
|  | AN0435 | Afu1g04510 | AFLA_029470 |
|  | AN10078 | Afu1g04780 | AFLA_029540 |
|  | AN0422 | Afu1g04830 | AFLA_029620 |
|  | AN0421 | Afu1g04840 | AFLA_029630 |
|  | AN0416 | Afu1g04880 | AFLA_029680 |
|  | AN0409 | Afu1g04960 | AFLA_029770 |
|  | AN7836 | Afu7g01060 | AFLA_029850 |
|  | AN0245 | Afu1g05290 | AFLA_029950 |
|  | AN2739 | Afu1g05260 | AFLA_029980 |
|  | AN2750 | Afu1g05140 | AFLA_030090 |
|  | AN2749 | Afu1g05130 | AFLA_030100 |
|  | AN2738 | Afu1g05120 | AFLA_030110 |
|  | AN2862 | Afu3g11860 | AFLA_030680 |
|  | AN2895 | Afu3g11500 | AFLA_031140 |
|  | AN2899 | Afu3g11450 | AFLA_031180 |
|  | AN4871 | Afu3g11280 | AFLA_031380 |
|  | AN4881 | Afu3g11150 | AFLA_031480 |
|  | AN4921 | Afu3g10680 | AFLA_032010 |
|  | AN11770 | Afu3g10600 | AFLA_032100 |
|  | AN4978 | Afu3g10100 | AFLA_032760 |
|  | AN4989 | Afu3g09980 | AFLA_032920 |
|  | AN0787 | Afu1g14560 | AFLA_033400 |
|  | AN5453 | Afu6g13380 | AFLA_033540 |
|  | AN5494 | Afu6g13160 | AFLA_033770 |
|  | AN9054 | Afu2g00520 | AFLA_034090 |
|  | AN3129 | Afu5g07320 | AFLA_034670 |
|  | AN2101 | Afu2g05080 | AFLA_035050 |
|  | AN2100 | Afu2g05070 | AFLA_035060 |
|  | AN1869 | Afu2g04510 | AFLA_035770 |
|  | AN1870 | Afu2g04480 | AFLA_035800 |
|  | AN1895 | Afu2g04240 | AFLA_036070 |
|  | AN1910 | Afu6g07610 | AFLA_036240 |
|  | AN3945 | Afu6g08130 | AFLA_036930 |
|  | AN5775 | Afu6g06535 | AFLA_037760 |
|  | AN5776 | Afu6g06520 | AFLA_037770 |
|  | AN5781 | Afu6g06470 | AFLA_037820 |
|  | AN10709 | Afu6g06340 | AFLA_037960 |
|  | AN7183 | Afu4g03170 | AFLA_039300 |
|  | AN1723 | Afu4g03230 | AFLA_039400 |
|  | AN7173 | Afu4g03320 | AFLA_039470 |
|  | AN7148 | Afu4g03595 | AFLA_041010 |
|  | AN7048 | Afu4g03980 | AFLA_041520 |
|  | AN7101 | Afu4g03820 | AFLA_041770 |
|  | AN7131 | Afu4g03800 | AFLA_041790 |
|  | AN10903 | Afu4g03770 | AFLA_041830 |
|  | AN0780 | Afu1g14490 | AFLA_041940 |
|  | AN0778 | Afu1g14430 | AFLA_041970 |
|  | AN4659 | Afu5g08470 | AFLA_042760 |
|  | AN5567 | Afu1g01530 | AFLA_043100 |
|  | AN4601 | Afu2g02220 | AFLA_043230 |
|  | AN1788 | Afu2g14655 | AFLA_043500 |
|  | AN5920 | Afu2g10890 | AFLA_043650 |
|  | AN5973 | Afu2g10620 | AFLA_043950 |
|  | AN10763 | Afu2g10640 | AFLA_043960 |
|  | AN5968 | Afu2g10560 | AFLA_044040 |
|  | AN5985 | Afu2g10230 | AFLA_044400 |
|  | AN5988 | Afu2g10190 | AFLA_044430 |
|  | AN6012 | Afu2g09940 | AFLA_044630 |
|  | AN6015 | Afu2g09890 | AFLA_044680 |
|  | AN6031 | Afu2g09850 | AFLA_044730 |
|  | AN6046 | Afu2g09680 | AFLA_044940 |
|  | AN6048 | Afu2g09650 | AFLA_045020 |
|  | AN6050 | Afu2g09630 | AFLA_045100 |
|  | AN6095 | Afu2g09450 | AFLA_045560 |
|  | AN8604 | Afu7g00850 | AFLA_045680 |
|  | AN6088 | Afu2g09282 | AFLA_045760 |
|  | AN6071 | Afu2g09070 | AFLA_045960 |
|  | AN6107 | Afu2g08900 | AFLA_046090 |
|  | AN6125 | Afu2g08690 | AFLA_046350 |
|  | AN6132 | Afu2g08660 | AFLA_046370 |
|  | AN6177 | Afu2g08180 | AFLA_046830 |
|  | AN10751 | Afu2g08090 | AFLA_046880 |
|  | AN5848 | Afu2g08010 | AFLA_046950 |
|  | AN5836 | Afu2g07900 | AFLA_046990 |
|  | AN10747 | Afu2g07870 | AFLA_047060 |
|  | AN5822 | Afu2g07690 | AFLA_047180 |
|  | AN5821 | Afu2g07630 | AFLA_047230 |
|  | AN8836 | Afu5g05900 | AFLA_048350 |
|  | AN2277 | Afu5g06340 | AFLA_048790 |
|  | AN2267 | Afu5g06440 | AFLA_048900 |
|  | AN6650 | Afu6g03590 | AFLA_049290 |
|  | AN4201 | Afu1g05980 | AFLA_049990 |
|  | AN4206 | Afu1g06020 | AFLA_050040 |
|  | AN4214 | Afu1g06090 | AFLA_050110 |
|  | AN10277 | Afu1g06130 | AFLA_050140 |
|  | AN3675 | Afu4g12470 | AFLA_050250 |
|  | AN3664 | Afu4g12350 | AFLA_050410 |
|  | AN4062 | Afu1g05370 | AFLA_050680 |
|  | AN4190 | Afu1g05790 | AFLA_051250 |
|  | AN3589 | Afu4g12890 | AFLA_051510 |
|  | AN3590 | Afu4g12880 | AFLA_051520 |
|  | AN3591 | Afu4g12870 | AFLA_051530 |
|  | AN3619 | Afu4g12680 | AFLA_051700 |
|  | AN5659 | Afu4g13600 | AFLA_052490 |
|  | AN5660 | Afu4g13670 | AFLA_052510 |
|  | AN3727 | Afu6g12380 | AFLA_052780 |
|  | AN3729 | Afu6g12400 | AFLA_052800 |
|  | AN8461 | Afu3g00710 | AFLA_053080 |
|  | AN6528 | Afu6g04890 | AFLA_055090 |
|  | AN6551 | Afu6g04830 | AFLA_055150 |
|  | AN6535 | Afu6g04690 | AFLA_055290 |
|  | AN6565 | Afu6g04550 | AFLA_055470 |
|  | AN6588 | Afu6g04300 | AFLA_055770 |
|  | AN6625 | Afu6g03900 | AFLA_056160 |
|  | AN5408 | Afu3g03050 | AFLA_056830 |
|  | AN7622 | Afu5g06980 | AFLA_057430 |
|  | AN10975 | Afu5g06970 | AFLA_057460 |
|  | AN2424 | Afu2g00640 | AFLA_057680 |
|  | AN10994 | Afu2g01130 | AFLA_058400 |
|  | AN7657 | Afu2g01170 | AFLA_058480 |
|  | AN7661 | Afu2g01260 | AFLA_058610 |
|  | AN10993 | Afu2g01310 | AFLA_058650 |
|  | AN1702 | Afu2g01370 | AFLA_058820 |
|  | AN2532 | Afu3g14590 | AFLA_059590 |
|  | AN8981 | Afu7g00990 | AFLA_060770 |
|  | AN7762 | Afu5g07640 | AFLA_061340 |
|  | AN7760 | Afu5g07670 | AFLA_061360 |
|  | AN11018 | Afu5g07902 | AFLA_061660 |
|  | AN7739 | Afu5g07940 | AFLA_061700 |
|  | AN7690 | Afu2g01610 | AFLA_062430 |
|  | AN7670 | Afu2g01430 | AFLA_062710 |
|  | AN1037 | Afu1g12530 | AFLA_066330 |
|  | AN2607 | Afu5g02620 | AFLA_066700 |
|  | AN10165 | Afu1g11910 | AFLA_067760 |
|  | AN10166 | Afu1g11900 | AFLA_067770 |
|  | AN1124 | Afu1g11720 | AFLA_068020 |
|  | AN1137 | Afu1g11590 | AFLA_068130 |
|  | AN10156 | Afu1g11550 | AFLA_068180 |
|  | AN1156 | Afu1g11450 | AFLA_068310 |
|  | AN1187 | Afu1g10900 | AFLA_068660 |
|  | AN1186 | Afu1g10890 | AFLA_068670 |
|  | AN1217 | Afu1g10580 | AFLA_069100 |
|  | AN1237 | Afu1g10410 | AFLA_069300 |
|  | AN1253 | Afu1g10260 | AFLA_069450 |
|  | AN1251 | Afu1g10230 | AFLA_069460 |
|  | AN1258 | Afu1g10210 | AFLA_069480 |
|  | AN11981 | Afu7g01560 | AFLA_070660 |
|  | AN9137 | Afu7g01695 | AFLA_070790 |
|  | AN11178 | Afu7g01700 | AFLA_070800 |
|  | AN8562 | Afu4g01580 | AFLA_070830 |
|  | AN9121 | Afu7g01930 | AFLA_071090 |
|  | AN8099 | Afu2g14610 | AFLA_073210 |
|  | AN1801 | Afu2g14620 | AFLA_073240 |
|  | AN4255 | Afu7g04040 | AFLA_073260 |
|  | AN5687 | Afu7g04200 | AFLA_073470 |
|  | AN5686 | Afu7g04210 | AFLA_073480 |
|  | AN5683 | Afu7g04240 | AFLA_073510 |
|  | AN5677 | Afu7g04300 | AFLA_073570 |
|  | AN3743 | Afu7g04400 | AFLA_073700 |
|  | AN3752 | Afu7g04580 | AFLA_073850 |
|  | AN3760 | Afu7g04700 | AFLA_074050 |
|  | AN10457 | Afu7g04795 | AFLA_074130 |
|  | AN3765 | Afu7g04800 | AFLA_074150 |
|  | AN8785 | Afu7g05220 | AFLA_075120 |
|  | AN8625 | Afu6g00680 | AFLA_075200 |
|  | AN6685 | Afu7g05340 | AFLA_076050 |
|  | AN6688 | Afu7g05370 | AFLA_076070 |
|  | AN6697 | Afu7g05450 | AFLA_076430 |
|  | AN6718 | Afu7g05752 | AFLA_076720 |
|  | AN11810 | Afu3g01260 | AFLA_078140 |
|  | AN1555 | Afu8g05630 | AFLA_078290 |
|  | AN1551 | Afu8g05610 | AFLA_078320 |
|  | AN1543 | Afu8g05530 | AFLA_078420 |
|  | AN1532 | Afu8g05410 | AFLA_078590 |
|  | AN1502 | Afu8g05020 | AFLA_078900 |
|  | AN1500 | Afu8g05010 | AFLA_078920 |
|  | AN7392 | Afu8g04910 | AFLA_078990 |
|  | AN9448 | Afu1g03030 | AFLA_080470 |
|  | AN9450 | Afu1g03040 | AFLA_080530 |
|  | AN10063 | Afu1g03050 | AFLA_080540 |
|  | AN0302 | Afu1g02760 | AFLA_080700 |
|  | AN0340 | Afu1g02270 | AFLA_081210 |
|  | AN0367 | Afu1g01910 | AFLA_081540 |
|  | AN1832 | Afu4g09580 | AFLA_081910 |
|  | AN1833 | Afu4g09560 | AFLA_081920 |
|  | AN10234 | Afu4g09550 | AFLA_081930 |
|  | AN10238 | Afu4g09540 | AFLA_081940 |
|  | AN6923 | Afu3g14170 | AFLA_082160 |
|  | AN8153 | Afu7g06680 | AFLA_082200 |
|  | AN0638 | Afu1g16870 | AFLA_082410 |
|  | AN0986 | Afu1g16745 | AFLA_082600 |
|  | AN0973 | Afu1g16590 | AFLA_082850 |
|  | AN0969 | Afu2g01380 | AFLA_082870 |
|  | AN11897 | Afu1g16600 | AFLA_082890 |
|  | AN11891 | Afu1g16460 | AFLA_083100 |
|  | AN0942 | Afu1g16270 | AFLA_083240 |
|  | AN0934 | Afu1g16200 | AFLA_083350 |
|  | AN0902 | Afu1g15680 | AFLA_083820 |
|  | AN10128 | Afu1g15370 | AFLA_084200 |
|  | AN0867 | Afu1g15180 | AFLA_084370 |
|  | AN0854 | Afu1g15110 | AFLA_084520 |
|  | AN0837 | Afu1g14960 | AFLA_084700 |
|  | AN0824 | Afu1g14850 | AFLA_084820 |
|  | AN0820 | Afu1g14800 | AFLA_084860 |
|  | AN4998 | Afu3g09900 | AFLA_084900 |
|  | AN3080 | Afu3g09700 | AFLA_085130 |
|  | AN3027 | Afu3g08850 | AFLA_085920 |
|  | AN2995 | Afu3g08620 | AFLA_086440 |
|  | AN2989 | Afu3g08580 | AFLA_086540 |
|  | AN2982 | Afu3g08480 | AFLA_086610 |
|  | AN2954 | Afu3g07870 | AFLA_087170 |
|  | AN2953 | Afu3g07890 | AFLA_087180 |
|  | AN2919 | Afu3g08010 | AFLA_087350 |
|  | AN3808 | Afu2g03780 | AFLA_087390 |
|  | AN3804 | Afu2g03830 | AFLA_087430 |
|  | AN3791 | Afu2g03990 | AFLA_087600 |
|  | AN5213 | Afu6g07470 | AFLA_087880 |
|  | AN1359 | Afu1g09270 | AFLA_088530 |
|  | AN1394 | Afu1g08850 | AFLA_088760 |
|  | AN9397 | Afu3g04070 | AFLA_089270 |
|  | AN11051 | Afu3g14980 | AFLA_089380 |
|  | AN9377 | Afu3g02940 | AFLA_090070 |
|  | AN9339 | Afu3g02270 | AFLA_090690 |
|  | AN10515 | Afu1g05930 | AFLA_090810 |
|  | AN11101 | Afu6g02300 | AFLA_091080 |
|  | AN8741 | Afu6g02690 | AFLA_091490 |
|  | AN10514 | Afu6g02770 | AFLA_091610 |
|  | AN8751 | Afu6g02840 | AFLA_091740 |
|  | AN10566 | Afu2g03130 | AFLA_092600 |
|  | AN4515 | Afu2g03120 | AFLA_092630 |
|  | AN0113 | Afu5g11890 | AFLA_092830 |
|  | AN6827 | Afu5g12895 | AFLA_093820 |
|  | AN6859 | Afu5g13070 | AFLA_094150 |
|  | AN6875 | Afu5g13190 | AFLA_094340 |
|  | AN9451 | Afu5g13650 | AFLA_094780 |
|  | AN2692 | Afu5g14020 | AFLA_095870 |
|  | AN2690 | Afu5g14030 | AFLA_095890 |
|  | AN6410 | Afu4g00770 | AFLA_098170 |
|  | AN6409 | Afu4g00760 | AFLA_098180 |
|  | AN8800 | Afu5g09610 | AFLA_098350 |
|  | AN8801 | Afu5g09600 | AFLA_098360 |
|  | AN8803 | Afu5g09580 | AFLA_098380 |
|  | AN12435 | Afu5g09390 | AFLA_098710 |
|  | AN4691 | Afu5g08900 | AFLA_099240 |
|  | AN4690 | Afu5g08910 | AFLA_099250 |
|  | AN4688 | Afu5g08930 | AFLA_099270 |
|  | AN4674 | Afu5g09020 | AFLA_099380 |
|  | AN4680 | Afu5g08990 | AFLA_099460 |
|  | AN4699 | Afu5g08770 | AFLA_099790 |
|  | AN10581 | Afu5g08640 | AFLA_099900 |
|  | AN4866 | Afu3g07720 | AFLA_100020 |
|  | AN9485 | Afu3g06570 | AFLA_100720 |
|  | AN10598 | Afu3g07020 | AFLA_101220 |
|  | AN4809 | Afu3g07030 | AFLA_101230 |
|  | AN4833 | Afu3g07260 | AFLA_102120 |
|  | AN4834 | Afu3g07270 | AFLA_102130 |
|  | AN4836 | Afu3g07290 | AFLA_102180 |
|  | AN10603 | Afu3g07510 | AFLA_102630 |
|  | AN4854 | Afu3g07570 | AFLA_102690 |
|  | AN4743 | Afu3g06300 | AFLA_102810 |
|  | AN3446 | Afu3g05575 | AFLA_103800 |
|  | AN2360 | Afu7g00800 | AFLA_104760 |
|  | AN2395 | Afu2g14520 | AFLA_104890 |
|  | AN5362 | Afu3g13760 | AFLA_106120 |
|  | AN2467 | Afu6g10340 | AFLA_106760 |
|  | AN7206 | Afu6g10330 | AFLA_107010 |
|  | AN1475 | Afu8g04660 | AFLA_107290 |
|  | AN11036 | Afu4g04140 | AFLA_108090 |
|  | AN10099 | Afu6g11440 | AFLA_108810 |
|  | AN0558 | Afu6g11390 | AFLA_108860 |
|  | AN0572 | Afu6g11290 | AFLA_108990 |
|  | AN0575 | Afu6g11200 | AFLA_109110 |
|  | AN0585 | Afu6g11110 | AFLA_109220 |
|  | AN1633 | Afu4g08980 | AFLA_110490 |
|  | AN1671 | Afu4g08970 | AFLA_110500 |
|  | AN1685 | Afu4g08630 | AFLA_111040 |
|  | AN1701 | Afu4g08470 | AFLA_111220 |
|  | AN4410 | Afu4g07180 | AFLA_112670 |
|  | AN4418 | Afu4g07090 | AFLA_112760 |
|  | AN4390 | Afu4g06820 | AFLA_113120 |
|  | AN4375 | Afu4g06610 | AFLA_113350 |
|  | AN4364 | Afu4g06520 | AFLA_113420 |
|  | AN4371 | Afu4g06510 | AFLA_113430 |
|  | AN4332 | Afu4g06290 | AFLA_113690 |
|  | AN4324 | Afu4g06170 | AFLA_113790 |
|  | AN4312 | Afu4g06040 | AFLA_113940 |
|  | AN4303 | Afu4g05940 | AFLA_114050 |
|  | AN12070 | Afu4g04160 | AFLA_114750 |
|  | AN7026 | Afu4g04230 | AFLA_114860 |
|  | AN6818 | Afu8g00480 | AFLA_116000 |
|  | AN9159 | Afu8g00910 | AFLA_116460 |
|  | AN3377 | Afu7g01200 | AFLA_116740 |
|  | AN5850 | Afu2g08050 | AFLA_117490 |
|  | AN10395 | Afu7g01370 | AFLA_117870 |
|  | AN10404 | Afu7g01410 | AFLA_117930 |
|  | AN6096 | Afu3g03160 | AFLA_118730 |
|  | AN10285 | Afu5g10660 | AFLA_119550 |
|  | AN12124 | Afu5g10490 | AFLA_119730 |
|  | AN10286 | Afu5g10430 | AFLA_119830 |
|  | AN7905 | Afu5g01250 | AFLA_121990 |
|  | AN7204 | Afu6g03050 | AFLA_122040 |
|  | AN4224 | Afu1g06320 | AFLA_127240 |
|  | AN10714 | Afu1g06650 | AFLA_127680 |
|  | AN5714 | Afu1g06762 | AFLA_127790 |
|  | AN5716 | Afu1g06780 | AFLA_127810 |
|  | AN5727 | Afu1g06910 | AFLA_127930 |
|  | AN3925 | Afu6g08390 | AFLA_127950 |
|  | AN3917 | Afu6g08460 | AFLA_128090 |
|  | AN3914 | Afu6g08510 | AFLA_128120 |
|  | AN1726 | Afu6g08830 | AFLA_128600 |
|  | AN1763 | Afu6g09140 | AFLA_128960 |
|  | AN7533 | Afu6g09250 | AFLA_129100 |
|  | AN7269 | Afu3g12950 | AFLA_129230 |
|  | AN7510 | Afu2g05325 | AFLA_129430 |
|  | AN10958 | Afu2g05350 | AFLA_129450 |
|  | AN7436 | Afu2g06150 | AFLA_130310 |
|  | AN4278 | Afu7g03760 | AFLA_131170 |
|  | AN4271 | Afu7g03830 | AFLA_131250 |
|  | AN4267 | Afu7g03840 | AFLA_131260 |
|  | AN4265 | Afu7g03890 | AFLA_131300 |
|  | AN4260 | Afu7g03970 | AFLA_131400 |
|  | AN7553 | Afu2g14800 | AFLA_131640 |
|  | AN10043 | Afu5g11170 | AFLA_132330 |
|  | AN0186 | Afu5g11190 | AFLA_132340 |
|  | AN0168 | Afu5g11330 | AFLA_132550 |
|  | AN4539 | Afu2g02820 | AFLA_133270 |
|  | AN4542 | Afu2g02730 | AFLA_133330 |
|  | AN10572 | Afu2g02450 | AFLA_133800 |
|  | AN10575 | Afu2g02440 | AFLA_133810 |
|  | AN5893 | Afu2g11180 | AFLA_134030 |
|  | AN5882 | Afu2g11310 | AFLA_134140 |
|  | AN10752 | Afu2g11470 | AFLA_134330 |
|  | AN10779 | Afu2g11870 | AFLA_134770 |
|  | AN6295 | Afu2g12310 | AFLA_135110 |
|  | AN6280 | Afu2g12520 | AFLA_135250 |
|  | AN10805 | Afu2g12490 | AFLA_135330 |
|  | AN6273 | Afu2g12630 | AFLA_135700 |
|  | AN6318 | Afu2g13440 | AFLA_136030 |
|  | AN6317 | Afu2g13430 | AFLA_136040 |
|  | AN6241 | Afu2g13160 | AFLA_136510 |
|  | AN6243 | Afu2g13140 | AFLA_136540 |
|  | AN6247 | Afu2g13100 | AFLA_136580 |
|  | AN2421 | Afu2g13770 | AFLA_137320 |
|  | AN6380 | Afu2g13930 | AFLA_137530 |
|  | AN6372 | Afu2g13990 | AFLA_137730 |
|  | AN6370 | Afu2g14010 | AFLA_137750 |
|  | AN6369 | Afu2g14020 | AFLA_137780 |
| All downregulated in the Δ*wetA* conidia | AN4913 | Afu3g00370 Afu3g10760 | AFLA_019650 AFLA_031900 |
|  | AN7632 | Afu2g01040 | AFLA_058310 AFLA_059790 AFLA_072340 |
|  | AN11921 | Afu8g07200 | AFLA_062120 AFLA_072920 AFLA_120010 |
|  | AN8639 | Afu4g03190 Afu5g14300 | AFLA_002830 |
|  | AN0391 | Afu1g01490 | AFLA_003760 AFLA_013730 |
|  | AN2470 | Afu2g00170 Afu5g03930 | AFLA_006730 |
|  | AN8621 | Afu3g02780 | AFLA_008410 AFLA_015590 |
|  | AN5563 | Afu4g11730 | AFLA_027730 AFLA_049340 |
|  | AN8587 | Afu1g00500 Afu8g06000 | AFLA_028100 |
|  | AN0895 | Afu1g15610 | AFLA_061270 AFLA_083890 |
|  | AN11094 | Afu6g10120 | AFLA_077990 AFLA_110070 |
|  | AN0963 AN10629 | Afu3g01900 | AFLA_097450 |
|  | AN10421 AN3523 | Afu6g13470 | AFLA_097700 |
|  | AN6349 | Afu2g14180 | AFLA_105670 AFLA_105680 |
|  | AN3243 AN8549 | Afu5g01248 | AFLA_138550 |
|  | AN6450 | Afu5g01040 | AFLA_000380 |
|  | AN9235 | Afu7g06610 | AFLA_001220 |
|  | AN7293 | Afu2g16890 | AFLA_002550 |
|  | AN8638 | Afu4g00730 | AFLA_002840 |
|  | AN8642 | Afu8g04870 | AFLA_002860 |
|  | AN7331 | Afu2g16530 | AFLA_003110 |
|  | AN8058 | Afu5g02190 | AFLA_003930 |
|  | AN7992 | Afu5g02500 | AFLA_004690 |
|  | AN8121 | Afu5g02720 | AFLA_004920 |
|  | AN8118 | Afu5g02750 | AFLA_004950 |
|  | AN8193 | Afu5g03210 | AFLA_005930 |
|  | AN8216 | Afu5g03490 | AFLA_006300 |
|  | AN11058 | Afu5g03520 | AFLA_006330 |
|  | AN11060 | Afu5g03600 | AFLA_006410 |
|  | AN12206 | Afu5g04100 | AFLA_006880 |
|  | AN11067 | Afu5g04120 | AFLA_006890 |
|  | AN7702 | Afu6g03140 | AFLA_007600 |
|  | AN10507 | Afu5g10270 | AFLA_007740 |
|  | AN5338 | Afu6g14280 | AFLA_007760 |
|  | AN10506 | Afu5g14780 | AFLA_008180 |
|  | AN5390 | Afu6g13770 | AFLA_008530 |
|  | AN5401 | Afu6g13860 | AFLA_008690 |
|  | AN5404 | Afu6g13670 | AFLA_009110 |
|  | AN3499 | Afu4g14600 | AFLA_009590 |
|  | AN6421 | Afu1g10870 | AFLA_010940 |
|  | AN1423 | Afu8g04150 | AFLA_011970 |
|  | AN3679 | Afu4g05870 | AFLA_012400 |
|  | AN12093 | Afu6g11670 | AFLA_013470 |
|  | AN7989 | Afu1g16930 | AFLA_014230 |
|  | AN3522 | Afu8g06080 | AFLA_014530 |
|  | AN2488 | Afu8g01690 | AFLA_014570 |
|  | AN10265 | Afu2g15910 | AFLA_014960 |
|  | AN12198 | Afu2g15810 | AFLA_015180 |
|  | AN10273 | Afu2g15770 | AFLA_015220 |
|  | AN2177 | Afu2g15740 | AFLA_015260 |
|  | AN7590 | Afu2g15430 | AFLA_015580 |
|  | AN10951 | Afu2g15320 | AFLA_015830 |
|  | AN7558 | Afu2g14870 | AFLA_016610 |
|  | AN0757 | Afu1g14180 | AFLA_017090 |
|  | AN3535 | Afu2g00130 | AFLA_017260 |
|  | AN9437 | Afu1g13920 | AFLA_017510 |
|  | AN0694 | Afu1g13560 | AFLA_017850 |
|  | AN0693 | Afu1g13550 | AFLA_017860 |
|  | AN0691 | Afu1g13530 | AFLA_017880 |
|  | AN0690 | Afu1g13520 | AFLA_017890 |
|  | AN0687 | Afu1g13490 | AFLA_017920 |
|  | AN0675 | Afu1g13370 | AFLA_018050 |
|  | AN0671 | Afu1g13310 | AFLA_018110 |
|  | AN0664 | Afu1g13250 | AFLA_018170 |
|  | AN11278 | Afu1g13195 | AFLA_018260 |
|  | AN6796 | Afu5g01430 | AFLA_019490 |
|  | AN11377 | Afu3g13910 | AFLA_020140 |
|  | AN3183 | Afu3g13230 | AFLA_020600 |
|  | AN1625 | Afu3g12760 | AFLA_020940 |
|  | AN10499 | Afu1g09750 | AFLA_021160 |
|  | AN1345 | Afu1g09440 | AFLA_021730 |
|  | AN5179 | Afu6g07100 | AFLA_021840 |
|  | AN5178 | Afu6g07090 | AFLA_021850 |
|  | AN5134 | Afu1g07380 | AFLA_022340 |
|  | AN6398 | Afu7g00360 | AFLA_023510 |
|  | AN9037 | Afu3g15190 | AFLA_023560 |
|  | AN9038 | Afu3g15170 | AFLA_023570 |
|  | AN12130 | Afu3g15160 | AFLA_023580 |
|  | AN8449 | Afu5g10420 | AFLA_023710 |
|  | AN8628 | Afu6g00510 | AFLA_024610 |
|  | AN8066 | Afu5g01850 | AFLA_024940 |
|  | AN8036 | Afu5g01910 | AFLA_025040 |
|  | AN8049 | Afu5g02080 | AFLA_025210 |
|  | AN11039 | Afu5g02110 | AFLA_025250 |
|  | AN0400 | Afu4g09610 | AFLA_025520 |
|  | AN2057 | Afu4g09750 | AFLA_025760 |
|  | AN1965 | Afu4g10790 | AFLA_026820 |
|  | AN1959 | Afu4g10860 | AFLA_026900 |
|  | AN11487 | Afu4g10920 | AFLA_026920 |
|  | AN5633 | Afu4g10990 | AFLA_026980 |
|  | AN5624 | Afu4g11110 | AFLA_027110 |
|  | AN5580 | Afu4g11550 | AFLA_027540 |
|  | AN5576 | Afu4g11590 | AFLA_027580 |
|  | AN5564 | Afu4g11720 | AFLA_027720 |
|  | AN10450 | Afu4g12140 | AFLA_028210 |
|  | AN4023 | Afu1g03690 | AFLA_028390 |
|  | AN4039 | Afu1g03960 | AFLA_028650 |
|  | AN0465 | Afu1g04320 | AFLA_029020 |
|  | AN0460 | Afu1g04380 | AFLA_029080 |
|  | AN0427 | Afu1g04760 | AFLA_029510 |
|  | AN2741 | Afu1g05220 | AFLA_030010 |
|  | AN2734 | Afu1g05080 | AFLA_030140 |
|  | AN2733 | Afu1g05060 | AFLA_030160 |
|  | AN5049 | Afu6g00440 | AFLA_030380 |
|  | AN5021 | Afu3g12100 | AFLA_030450 |
|  | AN2853 | Afu3g11955 | AFLA_030610 |
|  | AN2867 | Afu3g11830 | AFLA_030710 |
|  | AN10348 | Afu3g11820 | AFLA_030720 |
|  | AN2875 | Afu3g11690 | AFLA_030930 |
|  | AN2894 | Afu3g11550 | AFLA_031090 |
|  | AN4883 | Afu3g11140 | AFLA_031490 |
|  | AN10619 | Afu3g11120 | AFLA_031510 |
|  | AN4888 | Afu3g11070 | AFLA_031570 |
|  | AN4889 | Afu3g11060 | AFLA_031580 |
|  | AN10614 | Afu3g10920 | AFLA_031780 |
|  | AN4905 | Afu3g10830 | AFLA_031820 |
|  | AN4940 | Afu3g10480 | AFLA_032230 |
|  | AN4946 | Afu3g10410 | AFLA_032290 |
|  | AN4979 | Afu3g10090 | AFLA_032770 |
|  | AN4987 | Afu3g10000 | AFLA_032870 |
|  | AN0817 | Afu1g14750 | AFLA_033160 |
|  | AN5449 | Afu6g13440 | AFLA_033500 |
|  | AN5480 | Afu6g13330 | AFLA_033620 |
|  | AN10440 | Afu6g12790 | AFLA_034190 |
|  | AN3714 | Afu6g12730 | AFLA_034250 |
|  | AN11411 | Afu6g12720 | AFLA_034280 |
|  | AN3708 | Afu6g12680 | AFLA_034320 |
|  | AN3706 | Afu6g12660 | AFLA_034340 |
|  | AN3691 | Afu6g12560 | AFLA_034500 |
|  | AN11083 | Afu3g00660 | AFLA_034630 |
|  | AN2103 | Afu2g05090 | AFLA_035030 |
|  | AN10260 | Afu2g04930 | AFLA_035220 |
|  | AN2084 | Afu2g04900 | AFLA_035250 |
|  | AN11862 | Afu2g04610 | AFLA_035610 |
|  | AN1932 | Afu6g07840 | AFLA_036510 |
|  | AN4174 | Afu6g07940 | AFLA_036660 |
|  | AN3954 | Afu6g08050 | AFLA_036840 |
|  | AN5753 | Afu6g06840 | AFLA_037400 |
|  | AN5746 | Afu6g06770 | AFLA_037480 |
|  | AN5764 | Afu6g06670 | AFLA_037590 |
|  | AN8446 | Afu7g06780 | AFLA_038390 |
|  | AN10902 | Afu4g03390 | AFLA_040140 |
|  | AN9338 | Afu4g03420 | AFLA_040280 |
|  | AN4185 | Afu4g03430 | AFLA_040300 |
|  | AN0776 | Afu1g14410 | AFLA_041990 |
|  | AN8982 | Afu2g02030 | AFLA_043020 |
|  | AN4593 | Afu2g02140 | AFLA_043140 |
|  | AN4615 | Afu2g02310 | AFLA_043380 |
|  | AN10762 | Afu2g10740 | AFLA_043830 |
|  | AN5935 | Afu2g10690 | AFLA_043930 |
|  | AN5960 | Afu2g10440 | AFLA_044170 |
|  | AN5994 | Afu2g10120 | AFLA_044500 |
|  | AN5996 | Afu2g10100 | AFLA_044520 |
|  | AN5997 | Afu2g10090 | AFLA_044530 |
|  | AN6005 | Afu2g10020 | AFLA_044580 |
|  | AN6009 | Afu2g09990 | AFLA_044610 |
|  | AN6032 | Afu2g09840 | AFLA_044740 |
|  | AN8640 | Afu4g03615 | AFLA_044800 |
|  | AN6037 | Afu2g09790 | AFLA_044820 |
|  | AN6049 | Afu2g09640 | AFLA_045090 |
|  | AN6077 | Afu2g09130 | AFLA_045860 |
|  | AN6114 | Afu2g08840 | AFLA_046180 |
|  | AN6169 | Afu2g08290 | AFLA_046730 |
|  | AN6792 | Afu2g08250 | AFLA_046760 |
|  | AN8868 | Afu5g05480 | AFLA_048000 |
|  | AN8829 | Afu2g14330 | AFLA_048440 |
|  | AN2284 | Afu5g06270 | AFLA_048720 |
|  | AN2275 | Afu5g06360 | AFLA_048810 |
|  | AN8664 | Afu6g01940 | AFLA_049920 |
|  | AN3680 | Afu4g12500 | AFLA_050210 |
|  | AN3665 | Afu4g12440 | AFLA_050280 |
|  | AN3663 | Afu4g12340 | AFLA_050420 |
|  | AN3658 | Afu4g12260 | AFLA_050510 |
|  | AN7960 | Afu1g03580 | AFLA_050590 |
|  | AN4061 | Afu1g05350 | AFLA_050660 |
|  | AN4073 | Afu1g05500 | AFLA_050800 |
|  | AN4104 | Afu1g05860 | AFLA_051160 |
|  | AN4105 | Afu1g05850 | AFLA_051180 |
|  | AN10525 | Afu1g05820 | AFLA_051220 |
|  | AN3605 | Afu4g12710 | AFLA_051670 |
|  | AN4164 | Afu4g13180 | AFLA_051990 |
|  | AN1937 | Afu4g13230 | AFLA_052030 |
|  | AN3687 | Afu6g12500 | AFLA_053060 |
|  | AN8962 | Afu8g01890 | AFLA_053410 |
|  | AN6487 | Afu6g05350 | AFLA_054660 |
|  | AN6537 | Afu6g04705 | AFLA_055270 |
|  | AN6604 | Afu6g04170 | AFLA_055950 |
|  | AN8637 | Afu6g03890 | AFLA_056170 |
|  | AN6630 | Afu6g03820 | AFLA_056260 |
|  | AN2253 | Afu5g06647 | AFLA_056610 |
|  | AN8443 | Afu7g00720 | AFLA_056990 |
|  | AN2223 | Afu5g07140 | AFLA_057220 |
|  | AN7629 | Afu5g07380 | AFLA_057630 |
|  | AN7630 | Afu5g07390 | AFLA_057640 |
|  | AN7395 | Afu5g00840 | AFLA_058000 |
|  | AN7636 | Afu2g00970 | AFLA_058070 |
|  | AN5488 | Afu8g04920 | AFLA_059070 |
|  | AN5938 | Afu5g01380 | AFLA_059450 |
|  | AN10322 | Afu3g14460 | AFLA_060540 |
|  | AN11347 | Afu3g14440 | AFLA_060570 |
|  | AN11001 | Afu5g08360 | AFLA_062170 |
|  | AN10983 | Afu2g01630 | AFLA_062400 |
|  | AN7683 | Afu2g01590 | AFLA_062460 |
|  | AN7060 | Afu1g17430 | AFLA_065280 |
|  | AN1063 | Afu1g12290 | AFLA_067060 |
|  | AN1075 | Afu1g12080 | AFLA_067440 |
|  | AN1096 | Afu1g11950 | AFLA_067680 |
|  | AN1166 | Afu1g11130 | AFLA_068420 |
|  | AN1193 | Afu1g10830 | AFLA_068760 |
|  | AN1228 | Afu1g10510 | AFLA_069180 |
|  | AN1243 | Afu1g10370 | AFLA_069340 |
|  | AN1246 | Afu1g10350 | AFLA_069370 |
|  | AN8932 | Afu1g10110 | AFLA_069660 |
|  | AN1269 | Afu1g09980 | AFLA_069760 |
|  | AN7639 | Afu8g07030 | AFLA_069870 |
|  | AN8339 | Afu7g06770 | AFLA_070060 |
|  | AN5273 | Afu7g01720 | AFLA_070820 |
|  | AN11161 | Afu7g01730 | AFLA_070850 |
|  | AN9116 | Afu7g01880 | AFLA_071040 |
|  | AN9103 | Afu7g02070 | AFLA_071230 |
|  | AN6933 | Afu7g02420 | AFLA_071570 |
|  | AN3751 | Afu7g04510 | AFLA_073770 |
|  | AN6274 | Afu7g04540 | AFLA_073800 |
|  | AN6682 | Afu7g05320 | AFLA_074990 |
|  | AN6710 | Afu7g05680 | AFLA_076620 |
|  | AN1604 | Afu8g06030 | AFLA_077910 |
|  | AN1603 | Afu8g05985 | AFLA_077930 |
|  | AN2404 | Afu8g05810 | AFLA_078210 |
|  | AN1528 | Afu8g05370 | AFLA_078600 |
|  | AN1524 | Afu8g05330 | AFLA_078640 |
|  | AN10196 | Afu8g04830 | AFLA_079110 |
|  | AN1480 | Afu8g04760 | AFLA_079450 |
|  | AN5014 | Afu3g12300 | AFLA_079880 |
|  | AN2847 | Afu3g12280 | AFLA_079900 |
|  | AN0278 | Afu1g03110 | AFLA_080230 |
|  | AN0285 | Afu1g02980 | AFLA_080390 |
|  | AN0297 | Afu1g02820 | AFLA_080630 |
|  | AN0315 | Afu1g02560 | AFLA_080910 |
|  | AN0319 | Afu1g02490 | AFLA_080950 |
|  | AN10061 | Afu1g02050 | AFLA_081410 |
|  | AN0633 | Afu1g16980 | AFLA_082310 |
|  | AN10130 | Afu1g16523 | AFLA_083050 |
|  | AN5015 | Afu6g03210 | AFLA_083110 |
|  | AN0930 | Afu1g15940 | AFLA_083400 |
|  | AN6632 | Afu7g04490 | AFLA_083470 |
|  | AN6420 | Afu2g01100 | AFLA_083910 |
|  | AN0887 | Afu1g15520 | AFLA_084000 |
|  | AN0886 | Afu1g15530 | AFLA_084010 |
|  | AN0843 | Afu1g15020 | AFLA_084620 |
|  | AN5004 | Afu3g09830 | AFLA_084950 |
|  | AN3060 | Afu3g09350 | AFLA_085390 |
|  | AN3022 | Afu3g08900 | AFLA_085840 |
|  | AN3034 | Afu3g09030 | AFLA_085980 |
|  | AN3011 | Afu3g08770 | AFLA_086280 |
|  | AN2981 | Afu3g08470 | AFLA_086620 |
|  | AN2980 | Afu3g08460 | AFLA_086630 |
|  | AN10351 | Afu3g08290 | AFLA_086780 |
|  | AN3807 | Afu2g03790 | AFLA_087400 |
|  | AN5217 | Afu6g07520 | AFLA_087820 |
|  | AN5215 | Afu6g07490 | AFLA_087840 |
|  | AN9465 | Afu1g09100 | AFLA_088370 |
|  | AN1383 | Afu1g08960 | AFLA_088650 |
|  | AN10189 | Afu1g08950 | AFLA_088660 |
|  | AN9409 | Afu3g04260 | AFLA_089130 |
|  | AN9401 | Afu3g04150 | AFLA_089230 |
|  | AN11630 | Afu6g02705 | AFLA_091540 |
|  | AN4662 | Afu5g08490 | AFLA_091830 |
|  | AN4663 | Afu5g08500 | AFLA_091840 |
|  | AN4716 | Afu5g08580 | AFLA_091920 |
|  | AN9002 | Afu2g03620 | AFLA_092090 |
|  | AN3823 | Afu2g03590 | AFLA_092120 |
|  | AN4510 | Afu2g03170 | AFLA_092540 |
|  | AN0126 | Afu5g11700 | AFLA_092710 |
|  | AN7640 | Afu5g13780 | AFLA_093550 |
|  | AN7959 | Afu5g12830 | AFLA_093920 |
|  | AN6856 | Afu5g13100 | AFLA_094180 |
|  | AN8605 | Afu3g07430 | AFLA_094560 |
|  | AN6900 | Afu5g13450 | AFLA_094630 |
|  | AN8553 | Afu2g00200 | AFLA_096210 |
|  | AN5292 | Afu5g12490 | AFLA_097670 |
|  | AN3973 | Afu8g07130 | AFLA_097940 |
|  | AN8815 | Afu5g09450 | AFLA_098640 |
|  | AN0241 | Afu5g09240 | AFLA_099000 |
|  | AN0240 | Afu5g09230 | AFLA_099010 |
|  | AN10040 | Afu5g09180 | AFLA_099050 |
|  | AN4682 | Afu5g08980 | AFLA_099480 |
|  | AN11766 | Afu3g07660 | AFLA_100090 |
|  | AN4739 | Afu3g06210 | AFLA_100180 |
|  | AN4794 | Afu3g06840 | AFLA_101020 |
|  | AN3305 | Afu5g14310 | AFLA_101250 |
|  | AN2471 | Afu3g12070 | AFLA_102410 |
|  | AN4762 | Afu3g06460 | AFLA_103050 |
|  | AN3433 | Afu3g05760 | AFLA_103640 |
|  | AN3464 | Afu3g05390 | AFLA_103990 |
|  | AN8143 | Afu8g02400 | AFLA_105050 |
|  | AN3558 | Afu2g14300 | AFLA_105480 |
|  | AN11180 | Afu3g01760 | AFLA_105890 |
|  | AN2446 | Afu6g10560 | AFLA_106460 |
|  | AN8641 | Afu6g10450 | AFLA_106710 |
|  | AN7193 | Afu6g10260 | AFLA_106880 |
|  | AN0597 | Afu6g10970 | AFLA_109370 |
|  | AN2351 | Afu7g06260 | AFLA_110030 |
|  | AN1608 | Afu4g09250 | AFLA_110230 |
|  | AN1614 | Afu4g09190 | AFLA_110370 |
|  | AN1670 | Afu4g08960 | AFLA_110510 |
|  | AN1662 | Afu4g08810 | AFLA_110770 |
|  | AN10222 | Afu4g08880 | AFLA_110810 |
|  | AN1682 | Afu4g08680 | AFLA_111000 |
|  | AN11889 | Afu4g08290 | AFLA_111390 |
|  | AN3873 | Afu4g08240 | AFLA_111530 |
|  | AN3831 | Afu4g08130 | AFLA_111660 |
|  | AN4474 | Afu4g07725 | AFLA_112100 |
|  | AN4464 | Afu4g07690 | AFLA_112150 |
|  | AN10551 | Afu4g07140 | AFLA_112710 |
|  | AN4421 | Afu4g07050 | AFLA_112800 |
|  | AN4299 | Afu4g05900 | AFLA_114100 |
|  | AN7034 | Afu4g04200 | AFLA_114790 |
|  | AN7003 | Afu4g04460 | AFLA_115110 |
|  | AN12060 | Afu4g04540 | AFLA_115190 |
|  | AN10873 | Afu4g04620 | AFLA_115300 |
|  | AN3361 | Afu7g01430 | AFLA_117970 |
|  | AN0237 | Afu5g10840 | AFLA_119400 |
|  | AN10290 | Afu5g10650 | AFLA_119560 |
|  | AN2316 | Afu5g10560 | AFLA_119650 |
|  | AN10287 | Afu5g09970 | AFLA_120190 |
|  | AN3616 | Afu3g00350 | AFLA_121090 |
|  | AN4177 | Afu8g00600 | AFLA_121840 |
|  | AN5109 | Afu1g07650 | AFLA_123230 |
|  | AN4222 | Afu1g06340 | AFLA_127220 |
|  | AN2554 | Afu1g06730 | AFLA_127770 |
|  | AN5715 | Afu1g06770 | AFLA_127800 |
|  | AN10233 | Afu6g08730 | AFLA_128510 |
|  | AN1734 | Afu6g08740 | AFLA_128520 |
|  | AN1782 | Afu6g09210 | AFLA_129060 |
|  | AN7497 | Afu2g05500 | AFLA_129650 |
|  | AN7484 | Afu2g05720 | AFLA_129860 |
|  | AN7488 | Afu2g05750 | AFLA_129920 |
|  | AN7461 | Afu2g05900 | AFLA_130060 |
|  | AN10967 | Afu2g06205 | AFLA_130360 |
|  | AN4276 | Afu7g03770 | AFLA_131180 |
|  | AN8763 | Afu6g02960 | AFLA_131750 |
|  | AN0212 | Afu5g10990 | AFLA_132070 |
|  | AN10025 | Afu5g11280 | AFLA_132480 |
|  | AN0174 | Afu5g11290 | AFLA_132490 |
|  | AN10039 | Afu5g11500 | AFLA_132730 |
|  | AN2801 | Afu2g02950 | AFLA_133100 |
|  | AN4553 | Afu2g02630 | AFLA_133440 |
|  | AN5884 | Afu2g11290 | AFLA_134120 |
|  | AN6209 | Afu2g11940 | AFLA_134810 |
|  | AN10782 | Afu2g12080 | AFLA_134930 |
|  | AN6305 | Afu2g12200 | AFLA_135040 |
|  | AN10803 | Afu2g13000 | AFLA_135910 |
|  | AN6255 | Afu2g13010 | AFLA_135920 |
|  | AN9471 | Afu2g13200 | AFLA_136460 |
|  | AN9297 | Afu3g03570 | AFLA_138300 |
|  | AN11985 | Afu3g01210 | AFLA_138590 |
|  | AN2822 | Afu4g00680 | AFLA_138670 |
|  | AN10021 | Afu4g14550 | AFLA_139340 |
