# Supplementary material for: Systematic Dissection of the Evolutionarily Conserved WetA Developmental Regulator across a Genus of Filamentous Fungi
Source: mBio. 2018 Aug 21;9(4):e01130-18. doi: 10.1128/mBio.01130-18 (PMC6106085; doi:10.1128/mBio.01130-18)
Supplement: TABLE S3 [file mbo004184026st3.docx]

Table S3 DEGs related with asexual development in the Δ*wetA* conidia

|  | ***A. nidulans*** | | ***A. fumigauts*** | | ***A. flavus*** | |
| --- | --- | --- | --- | --- | --- | --- |
|  | **ID** | **Log_2_ Fold Change** | **ID** | **Log_2_ Fold Change** | **ID** | **Log_2_ Fold Change** |
| *abaA* | AN0422 | 4.96 | Afu1g04830 | 3.72 | AFLA_029620 | 2.96 |
| *acyA* | AN2623 | 4.03 | Afu3g12620 |  | AFLA_070880 | 3.90 |
| *ams1* | AN2936 | 1.56 | Afu3g08200 |  | AFLA_086900 | 1.02 |
| *argB* | AN4409 |  | Afu4g07190 |  | AFLA_112660 | -1.53 |
| *atfB* | AN6849 | -1.79 | Afu5g12960 | 1.53 | AFLA_094010 | -6.01 |
| *atg1* | AN1632 | 1.42 | Afu4g09050 |  | AFLA_110620 | 1.39 |
| *atgH* | AN5131 | 3.34 | Afu1g07470 | 1.08 | AFLA_022400 | 1.23 |
| *bem1* | AN7030 | 1.16 | Afu4g04120 | 2.68 | AFLA_114720 | -1.12 |
| *brlA* | AN0973 | 6.65 | Afu1g16590 | 1.93 | AFLA_082850 | 3.31 |
| *cch1* | AN1168 |  | Afu1g11110 |  | AFLA_068440 | 1.53 |
| *chsA* | AN4566 |  | Afu2g01870 | 2.50 | AFLA_042780 | 1.95 |
| *chsB* | AN7032 | 2.19 | Afu4g04180 | 1.25 | AFLA_114760 | 1.72 |
| *chsE* | AN6318 | 4.97 | Afu2g13440 | 3.56 | AFLA_136030 | 3.37 |
| *chsF* | AN1555 | 3.05 | Afu8g05630 | 6.59 | AFLA_078290 | 2.43 |
| *chsG* | AN2523 | 2.55 | Afu3g14420 | 4.00 | AFLA_060590 | 2.59 |
| *cmdA* | AN2047 |  | Afu4g10050 |  | AFLA_026020 | 1.59 |
| *cnaB* | AN6566 | -1.59 | Afu6g04540 |  | AFLA_055480 | -0.06 |
| *cpcB* | AN4163 | -2.58 | Afu4g13170 |  | AFLA_051980 | -0.91 |
| *crzA* | AN5726 |  | Afu1g06900 |  | AFLA_127920 | 1.57 |
| *dewA* | AN8006 | -9.48 | [Afu8g07060](http://www.aspergillusgenome.org/cgi-bin/compute/blast_clade.pl#Afu8g07060) | 3.48 | AFLA_060780 | -4.32 |
| *esdC* | AN9121 | 1.02 | Afu7g01930 | 3.32 | AFLA_071090 | 2.51 |
| *fadA* | AN0651 |  | Afu1g13140 | 3.82 | AFLA_018340 | 0.25 |
| *fbx15* | AN2505 | 1.25 | Afu3g14150 | 1.93 | AFLA_019100 | 1.74 |
| *figA* | AN3036 | 4.02 | Afu3g09060 |  | AFLA_086010 | 2.40 |
| *flbA* | AN5893 | 4.08 | Afu2g11180 | 2.70 | AFLA_134030 | 1.71 |
| *flbB* | AN7542 |  | Afu2g14680 | 1.17 | AFLA_131490 | 0.95 |
| *flbC* | AN2421 | 1.91 | Afu2g13770 | 4.92 | AFLA_137320 | 3.04 |
| *flbD* | AN0279 |  | Afu1g03210 | 7.20 | AFLA_080170 | 0.43 |
| *fluG* | AN4819 | -2.37 | Afu3g07140 | 1.88 | AFLA_039530 | -2.59 |
| *fphA* | AN9008 |  | Afu4g02900 | 1.02 | AFLA_065850 | -1.09 |
| *gpaA* | AN0651 |  | Afu1g13140 | 3.82 | AFLA_018340 | 0.25 |
| *gprC* | AN3765 | 1.85 | Afu7g04800 | 4.40 | AFLA_074150 | 2.04 |
| *gprD* | AN3387 | -1.72 | Afu2g12640 | 4.12 | AFLA_135680 | 0.49 |
| *hsp90* | AN8269 | 3.67 | Afu5g04170 | -2.33 | AFLA_006960 | -0.70 |
| *ime2* | AN6243 | 2.24 | Afu2g13140 | 3.44 | AFLA_136540 | 1.71 |
| *kex1* | AN10184 |  | Afu1g08940 |  | AFLA_088670 | -1.05 |
| *laeA* | AN0807 | -2.93 | Afu1g14660 | 2.92 | AFLA_033290 | 0.15 |
| *llmB/vipC* | AN8945 |  | Afu8g01930 |  | AFLA_121330 | -4.53 |
| *llmF* | AN6749 |  | Afu8g01930 |  | AFLA_008970 | 2.89 |
| *lreA* | AN3435 | -3.04 | Afu3g05780 | -1.73 | AFLA_103610 | 0.35 |
| *lreB* | AN3607 | -1.06 | Afu4g12690 | -3.43 | AFLA_051690 | -0.49 |
| *medA* | AN6230 |  | Afu2g13260 | 5.50 | AFLA_136410 | 2.81 |
| *midA* | AN4897 | 3.21 | Afu3g10960 | 3.26 | AFLA_031700 | 2.89 |
| *mob1* | AN6288 | 2.26 | Afu2g12390 |  | AFLA_135550 | 2.38 |
| *mpkB* | AN3719 | 4.29 | Afu6g12820 | 1.98 | AFLA_034170 | 0.96 |
| *msdS* | AN0787 | 9.00 | Afu1g14560 | 4.99 | AFLA_033400 | 4.17 |
| *msnA* | AN1652 | -1.66 | Afu4g09080 | 3.52 | AFLA_110650 | 2.23 |
| *mtfA* | AN8741 | 1.51 | Afu6g02690 | 3.71 | AFLA_091490 | 2.27 |
| *napA* | AN8863 |  | Afu5g05540 | 1.50 | AFLA_048050 | 0.43 |
| *nce102* | AN7683 | -2.44 | Afu2g01590 | -4.35 | AFLA_062460 | -5.13 |
| *nsdC* | AN4263 | 1.60 | Afu7g03910 |  | AFLA_131330 | 2.72 |
| *nsdD* | AN3152 |  | Afu3g13870 | 2.78 | AFLA_020210 | 1.68 |
| *nudA* | AN0118 | 2.76 | Afu5g11810 |  | AFLA_092800 | 2.17 |
| *nudG* | AN0420 |  | Afu1g04850 | -1.22 | AFLA_029640 | -1.26 |
| *odeA* | AN1037 | 1.50 | Afu1g12530 | 3.08 | AFLA_066330 | 1.85 |
| *orlA* | AN3441 | -2.90 | Afu3g05650 |  | AFLA_103730 | -0.59 |
| *osaA* | AN6578 |  | Afu6g04490 |  | AFLA_055650 | -1.50 |
| *pac2/osaB* | AN3074 |  | Afu3g09640 | 2.06 | AFLA_085200 | 1.08 |
| *pbcR* | AN1599 |  | Afu7g01640 |  | AFLA_028410 | -1.02 |
| *pcl1* | AN0453 | 5.52 | Afu1g04750 | 4.14 | AFLA_029150 | 2.40 |
| *phnA* | AN0082 |  | Afu5g12200 | -1.39 | AFLA_093230 | 1.66 |
| *pkaA* | AN6305 | -2.36 | Afu2g12200 | -3.46 | AFLA_135040 | -2.63 |
| *pkaB* | AN4717 |  | Afu5g08570 | -1.16 | AFLA_091910 | -2.09 |
| *pkaR* | AN4987 | -1.69 | Afu3g10000 | -1.81 | AFLA_032870 | -1.36 |
| *pksP* | AN8209 | -4.97 | Afu2g17600 | 1.22 | AFLA_006170 | -5.70 |
| *ppoA* | AN1967 |  | Afu4g10770 | -2.51 | AFLA_026790 | -1.46 |
| *ppoB* | AN6320 | 4.75 | Afu4g00180 | -11.69 | AFLA_120760 | -11.01 |
| *ppoC* | AN5028 | 3.00 | Afu3g12120 | 5.41 | AFLA_030430 | 1.23 |
| *ppoD* | - |  | - |  | AFLA_021100 | 3.74 |
| *prpA* | AN3129 | 1.85 | Afu5g07320 | 2.50 | AFLA_034670 | 5.31 |
| *rasA* | AN0182 |  | Afu5g11230 | 2.06 | AFLA_132380 | 0.21 |
| *rasB* | AN5832 | 1.88 | Afu2g07770 |  | AFLA_047110 | 0.17 |
| *rgdA* | AN4745 | 2.26 | Afu3g06280 |  | AFLA_102850 | 2.22 |
| *rgsA* | AN5755 |  | Afu6g06860 | 1.01 | AFLA_037370 | 0.56 |
| *rhbA* | AN8868 | -1.30 | Afu5g05480 | -2.91 | AFLA_048000 | -1.15 |
| *rho1* | AN5740 | 1.09 | Afu6g06900 |  | AFLA_037320 | 1.58 |
| *ricA* | AN1661 |  | Afu4g08820 | -1.33 | AFLA_110790 | -1.07 |
| *rlmA* | AN2984 | 1.73 | Afu3g08520 |  | AFLA_086590 | 0.34 |
| *rodA* | AN8803 | 7.02 | Afu5g09580 |  | AFLA_098380 | 1.72 |
| *rodB* | - |  | Afu1g17250 | 6.54 | AFLA_014260 | -2.00 |
| *sfaD* | AN0081 | 1.59 | Afu5g12210 | 2.31 | AFLA_093240 | 0.47 |
| *sfgA* | AN8129 | -1.13 | Afu5g02800 |  | AFLA_005520 | -2.82 |
| *sidB* | AN8751 | 2.40 | Afu6g02840 | 2.09 | AFLA_091740 | 1.39 |
| *sltA* | AN2919 | 3.33 | Afu3g08010 | 3.36 | AFLA_087350 | 2.86 |
| *ssc1* | AN9467 |  | Afu5g02560 | 1.16 | AFLA_004760 | 1.40 |
| *steC* | AN2269 |  | Afu5g06420 | 2.37 | AFLA_048880 | 1.48 |
| *steD* | AN7252 |  | Afu2g17130 | 2.55 | AFLA_002340 | -0.45 |
| *stuA* | AN5836 | 4.03 | Afu2g07900 | 5.61 | AFLA_046990 | 2.32 |
| *swoM* | AN6037 | -1.22 | Afu2g09790 | -1.29 | AFLA_044820 | -1.43 |
| *tcpA* | AN0641 |  | Afu1g16840 |  | AFLA_082510 | -1.07 |
| *tmpA* | AN0055 | -2.08 | Afu5g12520 |  | AFLA_093580 | -2.48 |
| *tpsA* | AN5523 | -1.62 | Afu6g12950 | -2.09 | AFLA_034030 | 0.25 |
| *tpsB* | AN5523 | -1.62 | Afu2g04010 | -1.84 | AFLA_087630 | -2.65 |
| *tpsC* | AN10533 | -1.50 | Afu7g03940 | -2.63 | AFLA_131370 | -2.30 |
| *ugtA* | AN3113 |  | Afu3g12700 | 2.63 | AFLA_020990 | 1.34 |
| *vapA* | AN0186 | 2.98 | Afu5g11190 | 5.93 | AFLA_132340 | 1.48 |
| *veA* | AN1052 | -2.43 | Afu1g12490 | 2.44 | AFLA_066460 | -1.90 |
| *velB* | AN0363 | 1.07 | Afu1g01970 | -2.20 | AFLA_081490 | 0.20 |
| *velC* | AN2059 | 2.36 | Afu4g09770 | 1.07 | AFLA_025780 | 0.82 |
| *vosA* | AN1959 | -3.35 | Afu4g10860 | -3.28 | AFLA_026900 | -3.70 |
| *vosB* | - |  | - |  | AFLA_074470 | -6.25 |
| *wetA* | AN1937 | -10.99 | Afu4g13230 | -12.90 | AFLA_052030 | -10.74 |
| *wsc1* | AN4674 | 5.45 | Afu5g09020 | 3.89 | AFLA_099380 | 3.60 |
| *wsc3* | AN5660 | 3.81 | Afu4g13670 | 5.57 | AFLA_052510 | 3.01 |
| *zipA* | AN11891 | 1.74 | Afu1g16460 | 3.77 | AFLA_083100 | 1.81 |
