# Supplementary material for: Systematic Dissection of the Evolutionarily Conserved WetA Developmental Regulator across a Genus of Filamentous Fungi
Source: mBio. 2018 Aug 21;9(4):e01130-18. doi: 10.1128/mBio.01130-18 (PMC6106085; doi:10.1128/mBio.01130-18)
Supplement: TABLE S4 [file mbo004184026st4.docx]

Table S4 G-protein pathway related DEGs in the Δ*wetA* conidia in three *Aspergillus* species

| GENE ID | *A. nidulans* | | *A. fumigauts* | | *A. flavus* | |
| --- | --- | --- | --- | --- | --- | --- |
|  |  | **Log_2_ Fold Change** |  | **Log_2_ Fold Change** |  | **Log_2_ Fold Change** |
| *fadA*/*gpaA* | AN0651 | - | Afu1g13140 | 3.82 | AFLA_018340 | - |
| *flbA* | AN5893 | 4.08 | Afu2g11180 | 2.7 | AFLA_134030 | 1.71 |
| *ganA* | AN3090 | -1.72 | Afu3g12400 | 2.88 | AFLA_079780 | 1.20 |
| *gprA* | AN2520 | - | Afu3g14330 | 1.58 | AFLA_060740 | 2.03 |
| *gprB* | AN7743 | -3.67 | Afu5g07880 | -1.24 | AFLA_061620 | - |
| *gprC* | AN3765 | 1.85 | Afu7g04800 | 4.4 | AFLA_074150 | 2.04 |
| *gprD* | AN3387 | -1.72 | Afu2g12640 | 4.12 | AFLA_135680 | - |
| *gprE* | AN9199 | 3.01 | NA | NA | NA | NA |
| *gprF* | AN12206 | -3.59 | Afu5g04100 | -2.79 | AFLA_006880 | -2.68 |
| *gprG* | AN10166 | 2.71 | Afu1g11900 | 3.86 | AFLA_067770 | 3.09 |
| *gprH* | AN8262 | - | Afu5g04135 | 2.07 | AFLA_006920 | -3.09 |
| *gprK* | AN7795 | 4.29 | Afu4g01350 | 1.67 | AFLA_009790 | -1.41 |
| *gprM* | AN6680 | 2.01 | Afu7g05300 | 2.04 | AFLA_075000 | - |
| *gprO* | AN4932 | - | Afu3g10570 | 1.32 | AFLA_032130 | - |
| *gprR* | NA | NA | NA | NA | AFLA_023070 | -2.93 |
| *gprS* | NA | NA | NA | NA | AFLA_006320 | 1.16 |
| *nopA* | AN3361 | -1.16 | Afu7g01430 | -3.51 | AFLA_117970 | -3.04 |
| *pdeA* | AN0829 | 1.29 | Afu1g14890 | - | AFLA_084770 | -1.39 |
| *pkaA* | AN6305 | -2.35 | Afu2g12200 | -3.46 | AFLA_135040 | -2.63 |
| *pkaB* | AN4717 | - | Afu5g08570 | -1.16 | AFLA_091910 | -2.09 |

Note: “NA“: an ortholog does not exist in the genome. “-”: not differential expressed in Δ*wetA* conidia
