# Supplementary material for: Systematic Dissection of the Evolutionarily Conserved WetA Developmental Regulator across a Genus of Filamentous Fungi
Source: mBio. 2018 Aug 21;9(4):e01130-18. doi: 10.1128/mBio.01130-18 (PMC6106085; doi:10.1128/mBio.01130-18)
Supplement: TABLE S5 [file mbo004184026st5.docx]

Table S5 Kinases differentially expressed in the Δ*wetA* conidia

|  | *A. nidulans* | | *A. fumigatus* | | *A. flavus* | |
| --- | --- | --- | --- | --- | --- | --- |
|  | **ID** | **Log_2_ Fold Change** | **ID** | **Log_2_ Fold Change** | **ID** | **Log_2_ Fold Change** |
| *dakA* | **AN0034** | 1.18 | **Afu5g12690** | - | **AFLA_093790** | -3.14 |
| *atmA* | **AN0038** | -1.33 | **Afu5g12660** | 1.09 | **AFLA_093750** | - |
| *pkcA* | **AN0106** | 1.49 | **Afu5g11970** | - | **AFLA_092990** | - |
| *rio2* | **AN0124** | -2.13 | **Afu5g11730** | -1.90 |  |  |
|  | **AN0156** | -1.08 | **Afu5g11450** | - | **AFLA_132700** | - |
| *ireA* | **AN0235** | 3.74 | **Afu1g01720** | 2.32 | **AFLA_081860** | - |
|  | **AN0259** | - | **Afu1g03420** | 1.64 | **AFLA_080110** | - |
|  | **AN0699** | - | **Afu1g13600** | -1.72 | **AFLA_017810** | - |
| *aromA* | **AN0708** | -1.12 | **Afu1g13740** | 1.65 | **AFLA_017680** | - |
|  | **AN0929** | 1.66 | **Afu1g15930** | - | **AFLA_083430** | 1.32 |
| *pbsA* | **AN0931** | -1.67 | **Afu1g15950** | -2.63 | **AFLA_083380** | - |
| *oca2* | **AN10019** | 1.08 | **Afu5g11840** | -1.41 | **AFLA_092880** | -1.06 |
| *srpkF* | **AN10082** | 2.77 | **Afu6g02242** | 2.18 | **AFLA_090470** | - |
|  | **AN10156** | 2.34 | **Afu1g11550** | 2.41 | **AFLA_068180** | 3.36 |
| *null* | **AN10188** | -2.09 | **Afu1g08840** | - | **AFLA_088770** | - |
| *srpkG* | **AN10462** | 1.35 |  |  | **AFLA_090280** | -2.55 |
| *stk21* | **AN10485** | - | **Afu6g08590** | 1.68 | **AFLA_128210** | - |
| *prk1* | **AN10515** | 3.43 | **Afu1g05930** | 2.01 | **AFLA_090810** | 2.29 |
|  | **AN10551** | -2.81 | **Afu4g07140** | -1.97 | **AFLA_112710** | -1.10 |
|  | **AN10646** | - | **Afu1g07040** | -2.48 | **AFLA_022040** | -1.09 |
|  | **AN10682** | - | **Afu6g12890** | -1.76 | **AFLA_034120** | - |
|  | **AN10731** | -1.35 |  |  |  |  |
| *pclB* | **AN10741** | 1.61 | **Afu2g07660** | 2.33 | **AFLA_047210** | - |
| *pkpA* | **AN10800** | 1.66 | **Afu2g13600** | -1.91 | **AFLA_136730** | - |
| *snf4* | **AN10854** | - | **Afu5g12990** | -1.11 | **AFLA_094030** | -1.08 |
| *srpkB* | **AN10895** | -2.24 | **Afu6g02590** | 4.06 | **AFLA_106860** | - |
| *srpkE* | **AN10937** | 4.04 | **Afu3g02460** | -8.56 | **AFLA_009240** | - |
|  | **AN1096** | -1.08 | **Afu1g11950** | -1.77 | **AFLA_067680** | -2.15 |
|  | **AN11032** | - | **Afu5g02220** | - | **AFLA_004050** | 1.50 |
| *gin4* | **AN11101** | 3.95 | **Afu6g02300** | 2.79 | **AFLA_091080** | 2.64 |
| *kin1* | **AN1171** | - | **Afu1g11080** | 1.15 | **AFLA_068480** | 3.19 |
|  | **AN1176** | -1.11 | **Afu1g10980** | - | **AFLA_068540** | - |
|  | **AN11803** | -2.71 | **Afu4g10840** | - | **AFLA_026880** | -2.24 |
|  | **AN11814** | -6.30 |  |  | **AFLA_119130** | 1.67 |
|  | **AN4279** | - | **Afu7g03750** | 1.33 |  |  |
|  | **AN11932** | 3.11 | **Afu7g01620** | - | **AFLA_070720** | 1.66 |
| *sD* | **AN1194** | -2.00 | **Afu1g10820** | - | **AFLA_068790** | - |
| *pgkA* | **AN1246** | -2.45 | **Afu1g10350** | -3.19 | **AFLA_069370** | -2.11 |
| *srpkC* | **AN1315** | 2.18 |  |  | **AFLA_072390** | -1.69 |
|  | **AN1315** | 2.18 |  |  | **AFLA_072390** | -1.69 |
|  | **AN1370** | - | **Afu1g09170** | 3.58 | **AFLA_088430** | - |
| *plkA* | **AN1560** | - | **Afu8g05680** | 1.72 | **AFLA_078250** | - |
| *atg1* | **AN1632** | 1.42 | **Afu4g09050** | - | **AFLA_110620** | 1.39 |
|  | **AN1668** | - | **Afu4g08900** | -1.64 | **AFLA_110530** | - |
| *nimO* | **AN1779** | -1.76 | **Afu6g09180** | 1.22 | **AFLA_129020** | - |
| *sln1* | **AN1800** | - | **Afu2g00660** | - | **AFLA_039130** | 1.15 |
|  | **AN1854** | 1.47 | **Afu4g09910** | 2.05 | **AFLA_025890** | - |
| *phoB* | **AN1867** | 1.17 |  |  |  |  |
|  | **AN2211** | -1.42 | **Afu5g07030** | - | **AFLA_057380** | - |
| *gcn2* | **AN2246** | -1.05 | **Afu5g06750** | - | **AFLA_056720** | - |
| *ksp1* | **AN2265** | 1.35 | **Afu5g06470** | - |  |  |
|  | **AN2269** | - | **Afu5g06420** | 2.37 | **AFLA_048880** | 1.48 |
|  | **AN2272** | - | **Afu5g06390** | 1.02 | **AFLA_048840** | - |
|  | **AN2311** | - | **Afu5g10680** | - | **AFLA_119540** | 1.82 |
| *ffkC* | **AN2373** | 1.98 |  |  |  |  |
|  | **AN2412** | - | **Afu2g13680** | 3.37 | **AFLA_136990** | 1.89 |
| *ssn3* | **AN2489** | - | **Afu3g13990** | 1.14 | **AFLA_019840** | - |
|  | **AN2513** | - | **Afu3g14290** | - | **AFLA_098060** | -1.32 |
|  | **AN2766** | - | **Afu3g06080** | 1.06 | **AFLA_103280** | - |
| *mps1* | **AN2927** | 3.58 | **Afu3g08100** | 3.54 | **AFLA_087000** | - |
| *isr1* | **AN3001** | -2.33 | **Afu3g08710** | - |  |  |
|  | **AN3065** | - | **Afu3g09550** | 1.83 | **AFLA_085320** | - |
|  | **AN3110** | - | **Afu3g12670** | 2.58 | **AFLA_021030** | 2.35 |
|  | **AN3177** | - | **Afu3g13270** | 1.08 | **AFLA_020560** | - |
|  | **AN3181** | -3.01 | **Afu3g13210** | - | **AFLA_020620** | 1.18 |
|  | **AN3214** | -2.86 | **Afu4g01020** | -1.58 | **AFLA_024540** | -8.52 |
| *cdc7* | **AN3450** | 1.54 | **Afu3g05540** | - | **AFLA_103840** | 1.13 |
|  | **AN3619** | 1.20 | **Afu4g12680** | 1.08 | **AFLA_051700** | 2.46 |
|  | **AN3648** | 2.27 | **Afu4g12160** | 2.94 | **AFLA_028230** | - |
| *mpkB* | **AN3719** | 4.29 | **Afu6g12820** | 1.98 | **AFLA_034170** | - |
|  | **AN3755** | - | **Afu7g04640** | -1.19 | **AFLA_073980** | - |
| *erg12* | **AN3869** | 1.73 | **Afu4g07780** | 1.99 | **AFLA_112040** | - |
| *srrC* | **AN4134** | 3.46 |  |  |  |  |
| *teaR* | **AN4214** | 1.88 | **Afu1g06090** | 2.05 | **AFLA_050110** | 1.79 |
| *ura6* | **AN4258** | - | **Afu7g03990** | -1.37 | **AFLA_131420** | - |
| *stt4* | **AN4278** | 1.17 | **Afu7g03760** | 1.45 | **AFLA_131170** | 1.11 |
| *phoC* | **AN4310** | - | **Afu4g06020** | -1.06 | **AFLA_113980** | - |
|  | **AN4380** | 1.65 | **Afu4g06690** | 1.28 | **AFLA_113250** | - |
|  | **AN4382** | -2.43 | **Afu4g06710** | -2.39 | **AFLA_113230** | 2.69 |
|  | **AN4447** | 1.96 | **Afu4g07400** | 4.51 | **AFLA_112430** | - |
|  | **AN4483** | - | **Afu2g03490** | -1.68 | **AFLA_092240** | - |
| *psk1* | **AN4536** | 1.80 | **Afu2g02850** | -1.70 | **AFLA_133230** | 2.95 |
| *ckiA* | **AN4563** | 1.55 | **Afu2g02530** | - | **AFLA_133580** | - |
| *vps34* | **AN4709** | -1.08 | **Afu5g08670** | 1.08 | **AFLA_099880** | - |
| *pkaC2* | **AN4717** | - | **Afu5g08570** | -1.16 | **AFLA_091910** | -2.09 |
| *bck1* | **AN4887** | - | **Afu3g11080** | 1.63 | **AFLA_031560** | - |
|  | **AN4914** | - | **Afu3g10750** | -4.53 | **AFLA_031910** | -3.50 |
| *ran1* | **AN4935** | 1.23 | **Afu3g10530** | - | **AFLA_032170** | -1.54 |
|  | **AN4957** | 1.39 | **Afu3g10300** | -2.30 | **AFLA_032510** | -1.06 |
|  | **AN4984** | -1.23 | **Afu3g10040** | - | **AFLA_032830** | - |
| *pkaR* | **AN4987** | -1.69 | **Afu3g10000** | -1.81 | **AFLA_032870** | -1.36 |
|  | **AN5122** | -1.40 | **Afu1g07530** | - | **AFLA_022470** | - |
|  | **AN5144** | - | **Afu1g07220** | - | **AFLA_022220** | -1.35 |
| *pho80* | **AN5156** | 1.18 | **Afu1g07070** | - | **AFLA_022060** | - |
|  | **AN5167** | 1.07 | **Afu6g06990** | 5.82 | **AFLA_021950** | 2.31 |
| *pkiA* | **AN5210** | 1.44 | **Afu6g07430** | - | **AFLA_087900** | - |
| *fos-1* | **AN5296** | - | **Afu6g10240** | -1.32 | **AFLA_106830** | - |
| *chkA* | **AN5494** | 2.04 | **Afu6g13160** | 1.95 | **AFLA_033770** | 1.54 |
|  | **AN5529** | - | **Afu4g11890** | 4.79 | **AFLA_027910** | 1.72 |
| *mpkA* | **AN5666** | -1.15 | **Afu4g13720** | 1.48 | **AFLA_052570** | - |
| *mst1* | **AN5674** | 2.44 | **Afu7g04330** | 2.23 | **AFLA_073630** | - |
|  | **AN5719** | -2.16 |  |  |  |  |
| *stk22* | **AN5728** | -1.71 | **Afu1g06920** | - |  |  |
|  | **AN5757** | - | **Afu6g06870** | 1.66 | **AFLA_037350** | 1.55 |
| *ark1* | **AN5815** | - | **Afu2g07550** | - | **AFLA_047300** | -1.22 |
|  | **AN5817** | - | **Afu2g07570** | - | **AFLA_047280** | - |
| *ankA* | **AN5822** | 3.54 | **Afu2g07690** | 3.22 | **AFLA_047180** | 3.31 |
| *pdkA* | **AN5843** | 2.44 | **Afu5g14790** | -7.47 |  |  |
| *pkcB* | **AN5973** | 2.02 | **Afu2g10620** | 1.62 | **AFLA_043950** | 2.63 |
| *tor* | **AN5982** | - | **Afu2g10270** | 1.02 | **AFLA_044350** | - |
| *npkA* | **AN6044** | - | **Afu2g09710** | -1.32 | **AFLA_044920** | - |
|  | **AN6053** | - | **Afu2g09570** | 1.53 | **AFLA_045240** | 2.68 |
| *ffkG* | **AN6192** | 2.36 | **Afu2g11730** | 4.71 | **AFLA_134590** | - |
| *imeB* | **AN6243** | 2.24 | **Afu2g13140** | 3.44 | **AFLA_136540** | 1.71 |
| *mob1* | **AN6288** | 2.26 | **Afu2g12390** | - | **AFLA_135550** | 2.38 |
| *pkaA* | **AN6305** | -2.36 | **Afu2g12200** | -3.46 | **AFLA_135040** | -2.63 |
| *pod6* | **AN6339** | -1.94 | **Afu2g13640** | - | **AFLA_136840** | - |
|  | **AN6347** | - | **Afu2g14200** | 2.58 | **AFLA_105620** | - |
| *sudD* | **AN6363** | -2.49 | **Afu2g14090** | -1.04 | **AFLA_138010** | - |
|  | **AN6367** | - | **Afu2g14040** | -1.49 | **AFLA_137970** | -1.68 |
| *gsk3* | **AN6508** | 1.36 | **Afu6g05120** | 1.05 | **AFLA_054840** | - |
|  | **AN6570** | - | **Afu6g04500** | -1.20 | **AFLA_055520** | -1.44 |
|  | **AN6572** | -2.19 | **Afu6g04380** | - | **AFLA_055540** | - |
| *ffkJ* | **AN6758** | 2.70 |  |  |  |  |
|  | **AN6824** | - | **Afu5g12870** | -2.24 | **AFLA_093870** | -2.75 |
|  | **AN6943** | 3.48 |  |  |  |  |
| *uvsb* | **AN6975** | - | **Afu4g04760** | - | **AFLA_115480** | 1.24 |
| *bem1* | **AN7030** | 1.16 | **Afu4g04120** | 2.68 | **AFLA_114720** | -1.12 |
|  | **AN7185** | - | **Afu4g03140** | -1.21 | **AFLA_039070** | - |
|  | **AN7321** | - | **Afu2g16620** | - | **AFLA_002970** | -1.07 |
|  | **AN7469** | - | **Afu2g05820** | - | **AFLA_129990** | -1.62 |
|  | **AN7502** | -1.14 | **Afu2g05430** | - | **AFLA_129590** | - |
| *ppk33* | **AN7537** | - | **Afu6g09240** | - | **AFLA_129090** | - |
| *chkC* | **AN7563** | 5.65 | **Afu2g14920** | 4.04 | **AFLA_016700** | 1.55 |
| *snf1* | **AN7695** | -2.05 | **Afu2g01700** | -1.80 | **AFLA_062250** | - |
|  | **AN7737** | - | **Afu5g07950** | - | **AFLA_061710** | 6.15 |
| *ku70* | **AN7753** | - | **Afu5g07740** | -1.12 | **AFLA_061470** | 1.69 |
|  | **AN7787** | - |  |  |  |  |
|  | **AN7945** | - |  |  | **AFLA_104660** | -1.55 |
| *ffkA* | **AN7986** | 2.87 |  |  |  |  |
|  | **AN7995** | - | **Afu5g02530** | -2.32 | **AFLA_004730** | -1.10 |
|  | **AN8213** | -3.22 | **Afu5g03460** | -1.15 | **AFLA_006260** | - |
| *swoH* | **AN8216** | -2.09 | **Afu5g03490** | -3.32 | **AFLA_006300** | -1.35 |
|  | **AN8751** | 2.40 | **Afu6g02840** | 2.09 | **AFLA_091740** | 1.39 |
| *iki3* | **AN8790** | - | **Afu5g09840** | -1.29 | **AFLA_098240** | -1.49 |
| *cmkC* | **AN8827** | 2.16 | **Afu5g05980** | - | **AFLA_048460** | 1.48 |
| *cla4* | **AN8836** | 2.24 | **Afu5g05900** | 4.30 | **AFLA_048350** | 1.07 |
|  | **AN8837** | 2.16 | **Afu5g05890** | -1.71 | **AFLA_048340** | - |
|  | **AN8843** | -2.74 | **Afu5g05820** | -1.05 | **AFLA_048290** | - |
|  | **AN8859** | -1.69 | **Afu5g05590** | - | **AFLA_048100** | - |
| *ptkA* | **AN8865** | -1.10 |  |  | **AFLA_048030** | - |
|  | **AN9022** | -1.74 |  |  | **AFLA_040580** | 1.03 |
|  | **AN9024** | 2.30 |  |  |  |  |
| *tinC* | **AN9067** | 1.21 | **Afu7g02570** | 2.68 | **AFLA_071680** | -1.55 |
| *ffkK* | **AN9302** | - |  |  |  |  |
| *panK* | **AN9446** | -1.35 | **Afu3g07180** | - | **AFLA_102040** | -1.51 |
|  | **AN9461** | - | **Afu5g03240** | -1.35 | **AFLA_005960** | - |
|  | **AN9500** | 3.58 | **Afu6g02020** | - | **AFLA_049940** | - |
| *nimA* | **AN9504** | - | **Afu6g02670** | -1.43 | **AFLA_091450** | - |
|  |  |  | **Afu1g00530** | -2.02 | **AFLA_028130** | -2.85 |
|  |  |  | **Afu1g00640** | 2.66 | **AFLA_003780** | 3.03 |
|  |  |  | **Afu2g00670** | -1.09 |  |  |
| *tcsC* |  |  | **Afu2g03560** | 3.16 |  |  |
|  |  |  | **Afu4g04680** | 2.25 |  |  |
|  |  |  | **Afu4g14735** | 2.24 |  |  |
|  |  |  | **Afu4g14740** | 3.85 |  |  |
|  |  |  | **Afu5g03950** | - |  |  |
|  |  |  | **Afu5g06730** | 1.79 | **AFLA_138880** | - |
|  |  |  | **Afu5g08480** | -3.44 | **AFLA_091820** | - |
|  |  |  | **Afu5g13420** | 3.67 |  |  |
|  |  |  | **Afu6g03252** | 3.48 |  |  |
|  |  |  | **Afu7g00530** | 1.36 | **AFLA_005410** | -1.05 |
|  |  |  | **Afu7g04550** | -1.84 | **AFLA_073810** | 2.42 |
|  |  |  | **Afu7g04735** | 1.50 |  |  |
|  |  |  | **Afu8g06150** | -3.14 |  |  |
|  |  |  | **Afu8g06180** | 2.46 |  |  |
|  |  |  |  |  | **AFLA_008230** | -2.78 |
|  |  |  |  |  | **AFLA_009250** | -3.25 |
|  |  |  |  |  | **AFLA_010150** | -1.50 |
|  |  |  |  |  | **AFLA_040840** | -2.10 |
|  |  |  |  |  | **AFLA_043510** | -2.09 |
| *cdc2l1* |  |  |  |  | **AFLA_069750** | 2.37 |
| *bos1* |  |  |  |  | **AFLA_092160** | 1.10 |
| *phoA* | **AN8261** | - | **Afu5g04130** | 1.05 | **AFLA_006900** | - |
| The following genes are grouped by their orthogroup | | | | | | |
| *fphA* | **AN9008** | - | **Afu4g02900** | 1.02 | **AFLA_065850** | -1.09 |
|  |  |  |  |  | **AFLA_095440** | 5.45 |
|  |  |  |  |  | **AFLA_101910** | 2.09 |
| *sepH* | **AN4385** | 2.06 | **Afu4g06750** | 2.38 | **AFLA_113190** | 1.19 |
|  |  |  |  |  | **AFLA_113200** | 1.62 |
| *nimX* | **AN4182** | -1.23 | **Afu6g07980** | 4.43 | **AFLA_036730** | - |
|  |  |  |  |  | **AFLA_125910** | - |
|  | **AN6985** | 2.31 | **Afu4g04680** | 2.25 | **AFLA_097830** | 3.44 |
|  | **AN1568** | - |  | - | **AFLA_115360** | - |
|  | **AN4479** | 2.42 | **Afu2g03560** | 3.16 | **AFLA_092150** | 1.28 |
|  |  |  |  |  | **AFLA_092160** | 1.10 |
|  |  |  |  |  | **AFLA_119810** | - |
|  | **AN1568** | - | **Afu4g04680** | 2.25 | **AFLA_097830** | 3.44 |
|  | **AN6985** | 2.31 |  | - | **AFLA_115360** | - |
|  | **AN1017** | -1.20 | **Afu1g12940** | -1.92 | **AFLA_099500** | - |
|  | **AN4668** | - | **Afu5g09100** | -3.10 |  |  |
|  | **AN8886** | -1.22 | **Afu1g11630** | 1.87 | **AFLA_068090** | - |
|  | **AN1132** | - | **Afu8g02700** | - | **AFLA_122560** | 1.18 |
|  | **AN5589** | -1.54 | **Afu4g11540** | -2.20 | **AFLA_027530** | - |
|  | **AN3916** | - | **Afu6g08470** | 2.68 | **AFLA_128100** | - |
