# Supplementary material for: Systematic Dissection of the Evolutionarily Conserved WetA Developmental Regulator across a Genus of Filamentous Fungi
Source: mBio. 2018 Aug 21;9(4):e01130-18. doi: 10.1128/mBio.01130-18 (PMC6106085; doi:10.1128/mBio.01130-18)
Supplement: TABLE S6 [file mbo004184026st6.docx]

Table S6 Transcription factors differentially expressed in the Δ*wetA* conidia

|  | *A. nidulans* | | *A. fumigatus* | | *A. flavus* | |
| --- | --- | --- | --- | --- | --- | --- |
|  | **ID** | **Log_2_ Fold Change** | **ID** | **Log_2_ Fold Change** | **ID** | **Log_2_ Fold Change** |
| *sfgA* | **AN8129** | -1.13 | **Afu5g02800** | - | **AFLA_005520** | -2.82 |
|  | **AN12489** | 3.57 | **Afu2g16230** | 4.00 | **AFLA_003630** | 1.67 |
| *mdpE* | **AN0148** | -2.04 | **Afu4g14540** | -1.33 |  | - |
|  | **AN0333** | - | **Afu1g02360** | 1.22 | **AFLA_081130** | - |
| *rsrA* | **AN0273** | 1.38 |  |  |  |  |
|  |  |  | **Afu5g09170** | -1.45 | **AFLA_099570** | -2.18 |
|  | **AN10809** | 1.58 | **Afu2g14250** | -1.31 | **AFLA_105360** | - |
|  | **AN5806** | - | **Afu2g07460** | -3.33 |  |  |
|  | **AN11962** | - | **Afu3g14600** | 2.27 | **AFLA_059510** | - |
|  | **AN7434** | -1.09 | **Afu2g06130** | 1.84 | **AFLA_013090** | -2.16 |
|  | **AN7434** | -1.09 | **Afu2g05360** | 2.77 | **AFLA_013090** | -2.16 |
|  |  |  | **Afu3g01155** | -1.21 |  |  |
|  | **AN0585** | 1.11 | **Afu6g11110** | 4.36 | **AFLA_109220** | 1.73 |
| *cnjB* | **AN4133** | -3.17 | **Afu4g13060** | - | **AFLA_051900** | 1.35 |
|  | **AN0658** | -1.02 | **Afu1g13190** | - |  |  |
|  | **AN0671** | -1.48 | **Afu1g13310** | -1.73 | **AFLA_018110** | -1.18 |
|  |  |  | **Afu3g14750** | -1.86 |  |  |
|  | **AN0644** | 3.62 | **Afu1g13050** | - | **AFLA_018410** | 1.21 |
|  | **AN0718** | - | **Afu1g14460** | 2.22 | **AFLA_017290** | 1.36 |
| *zfpB* | **AN1251** | 3.37 | **Afu1g10230** | 4.64 | **AFLA_069460** | 3.11 |
|  | **AN5929** | 2.03 | **Afu2g10770** | 2.04 | **AFLA_043760** | - |
| *uaY* | **AN0891** | - | **Afu1g15470** | 1.62 | **AFLA_083940** | - |
|  | **AN0902** | 5.76 | **Afu1g15680** | 4.41 | **AFLA_083820** | 1.13 |
|  |  |  | **Afu1g16620** | 2.82 | **AFLA_082910** | - |
|  | **AN4175** | - | **Afu6g07950** | 2.91 | **AFLA_036680** | - |
|  | **AN1298** | 5.64 | **Afu1g09670** | 5.38 | **AFLA_021240** | 4.26 |
| *brlA* | **AN0973** | 6.65 | **Afu1g16590** | 1.93 | **AFLA_082850** | 3.31 |
|  | **AN6295** | 1.65 | **Afu2g12310** | 3.56 | **AFLA_135110** | 1.44 |
|  | **AN10059** | 4.56 |  |  | **AFLA_081600** | - |
|  | **AN10083** | 1.69 | **Afu6g03700** | - | **AFLA_108220** | - |
| *rosA* | **AN5170** | - | **Afu4g09710** | 1.52 |  |  |
|  | **AN10120** | - | **Afu1g14360** | 1.63 | **AFLA_042030** | -1.29 |
|  | **AN10128** | 1.12 | **Afu1g15370** | 1.28 | **AFLA_084200** | 1.59 |
|  | **AN10294** | - | **Afu5g09890** | - | **AFLA_120290** | -1.80 |
| *zipC* | **AN10378** | 1.79 | **Afu4g02940** | -1.64 | **AFLA_066000** | - |
|  | **AN10384** | - | **Afu5g14530** | - |  |  |
| *rfeG* | **AN4680** | 3.88 | **Afu5g08990** | 5.57 | **AFLA_099460** | 1.76 |
|  | **AN10491** | 1.08 | **Afu6g08550** | - | **AFLA_128160** | - |
|  | **AN3667** | - | **Afu4g12410** | 1.09 | **AFLA_050300** | -3.01 |
| *galX* | **AN10543** | -2.46 | **Afu1g11000** | - | **AFLA_049270** | -1.42 |
|  | **AN10548** | -1.57 | **Afu4g06420** | -2.04 | **AFLA_113510** | - |
|  | **AN10600** | 2.73 |  |  | **AFLA_000770** | - |
|  | **AN11165** | 1.29 | **Afu7g01960** | 1.79 |  |  |
|  | **AN11222** | 2.59 | **Afu2g17880** | - | **AFLA_124220** | 2.51 |
| *qutA* | **AN1134** | -1.06 | **Afu1g11620** | 2.05 |  |  |
| *wetA* | **AN1937** | -10.99 | **Afu4g13230** | -12.90 | **AFLA_052030** | -10.74 |
| *zipA* | **AN11891** | 1.74 | **Afu1g16460** | 3.77 | **AFLA_083100** | 1.81 |
|  | **AN1217** | 2.10 | **Afu1g10580** | 3.97 | **AFLA_069100** | 3.18 |
|  | **AN3761** | 2.31 | **Afu7g04710** | - | **AFLA_074060** | 1.28 |
|  | **AN3063** | - | **Afu3g09380** | -2.13 | **AFLA_085360** | -1.13 |
| *srbA* | **AN7661** | 1.81 | **Afu2g01260** | 1.17 | **AFLA_058610** | 1.54 |
| *zapA* | **AN1265** | -1.71 | **Afu1g10080** | -1.38 | **AFLA_069700** | - |
|  | **AN1536** | 1.67 | **Afu8g05460** | 3.26 | **AFLA_078500** | - |
|  | **AN8753** | 2.80 | **Afu6g02850** | 2.25 | **AFLA_091710** | -1.14 |
|  | **AN0568** | -1.46 | **Afu6g11230** | -1.53 | **AFLA_109030** | - |
|  | **AN1569** | - |  |  | **AFLA_097720** | -2.91 |
| *rfeB* | **AN2009** | 2.14 | **Afu4g10220** | 4.22 | **AFLA_026250** | 2.16 |
| *msnA* | **AN1652** | -1.66 | **Afu4g09080** | 3.52 | **AFLA_110650** | 2.23 |
|  | **AN1678** | 2.18 |  |  | **AFLA_054310** | -3.24 |
|  | **AN1736** | -1.45 | **Afu2g00880** | 1.64 | **AFLA_104780** | 1.57 |
| *jlbA* | **AN1812** | 1.48 |  |  |  |  |
|  | **AN0817** | -1.59 | **Afu1g14750** | -1.97 | **AFLA_033160** | -1.27 |
|  | **AN1924** | 1.52 | **Afu6g07780** | 4.63 | **AFLA_036460** | - |
|  | **AN8596** | -2.00 | **Afu1g17150** | - | **AFLA_120780** | -3.10 |
|  | **AN2025** | 2.89 |  |  |  |  |
| *dopA* | **AN2094** | -1.03 | **Afu2g05020** | - | **AFLA_035130** | - |
|  | **AN1212** | -2.04 | **Afu1g10660** | 1.68 | **AFLA_068970** | - |
|  | **AN2367** | - |  |  | **AFLA_024570** | -1.01 |
| *flbC* | **AN2421** | 1.91 | **Afu2g13770** | 4.92 | **AFLA_137320** | 3.04 |
|  | **AN2615** | -1.82 | **Afu7g00210** | -4.32 |  |  |
| *metR* | **AN4361** | -1.08 | **Afu4g06530** | -2.94 | **AFLA_113450** | - |
| *hexA* | **AN4695** | 4.36 | **Afu5g03920** | -1.83 | **AFLA_099650** | - |
| *pacC* | **AN2855** | 2.08 | **Afu3g11970** | - | **AFLA_030580** | 1.17 |
| *nscR* |  |  | **Afu7g00130** | 1.44 |  |  |
| *sltA* | **AN2919** | 3.33 | **Afu3g08010** | 3.36 | **AFLA_087350** | 2.86 |
|  | **AN7170** | - | **Afu4g03460** | -2.31 | **AFLA_040260** | 2.00 |
| *rlmA* | **AN2984** | 1.73 | **Afu3g08520** | - | **AFLA_086590** | - |
| *mbf1* | **AN2996** | 2.67 | **Afu3g08630** | - | **AFLA_086430** | - |
| *abaA* | **AN0422** | 4.96 | **Afu1g04830** | 3.72 | **AFLA_029620** | 2.96 |
| *tbp* | **AN4976** | - | **Afu3g10120** | -2.03 | **AFLA_032740** | - |
|  | **AN3216** | 1.54 |  |  |  |  |
| *oefC* | **AN3075** | 5.13 | **Afu3g09670** | 3.05 | **AFLA_085170** | - |
|  | **AN0026** | - | **Afu5g09990** | -3.30 | **AFLA_138930** | -1.72 |
|  | **AN3385** | - |  |  | **AFLA_116880** | - |
| *prtT* |  |  | **Afu4g10120** | 2.40 | **AFLA_026130** | - |
|  | **AN0096** | - | **Afu5g12060** | - | **AFLA_093070** | -2.15 |
|  | **AN3433** | -4.20 | **Afu3g05760** | -4.30 | **AFLA_103640** | -4.14 |
| *lreA* | **AN3435** | -3.04 | **Afu3g05780** | -1.73 | **AFLA_103610** | - |
|  | **AN3501** | -1.72 | **Afu4g14590** | - | **AFLA_009580** | -2.63 |
|  | **AN3502** | -1.75 | **Afu1g06540** | - | **AFLA_107030** | -1.06 |
|  | **AN3506** | -2.63 |  |  | **AFLA_123840** | -2.87 |
| *lreB* | **AN3607** | -1.06 | **Afu4g12690** | -3.43 | **AFLA_051690** | - |
|  | **AN7610** | 1.08 | **Afu2g15620** | 1.28 | **AFLA_015390** | - |
| *cpcA* | **AN3675** | 1.92 | **Afu4g12470** | 3.74 | **AFLA_050250** | 1.75 |
| *flbD* | **AN0279** | - | **Afu1g03210** | 7.20 | **AFLA_080170** | - |
| *vosA* | **AN1959** | -3.35 | **Afu4g10860** | -3.28 | **AFLA_026900** | -3.70 |
|  | **AN3769** | 1.90 | **Afu7g04820** | -1.98 | **AFLA_074200** | 1.41 |
| *fhpA* | **AN4521** | 1.46 |  |  | **AFLA_132980** | - |
|  | **AN0486** | - | **Afu3g02070** | - | **AFLA_013890** | -1.17 |
| *facB* | **AN0689** | - | **Afu1g13510** | -2.72 | **AFLA_017900** | - |
| *rpn4* | **AN0709** | 1.17 | **Afu1g13750** | -1.15 | **AFLA_017640** | 2.87 |
|  | **AN0094** | -1.10 | **Afu5g12080** | - | **AFLA_093110** | - |
|  | **AN10432** | - |  |  | **AFLA_040220** | -2.62 |
|  | **AN3290** | 5.63 |  |  | **AFLA_101420** | -4.05 |
|  | **AN4001** | -1.65 | **Afu1g04170** | - |  |  |
|  | **AN10192** | - | **Afu8g04540** | - | **AFLA_139560** | -3.60 |
| *sln1* | **AN1028** | - | **Afu3g02700** | 2.90 | **AFLA_038210** | -2.39 |
|  | **AN4185** | -1.02 | **Afu4g03430** | -2.82 | **AFLA_040300** | -7.03 |
| *nsdC* | **AN4263** | 1.60 | **Afu7g03910** | - | **AFLA_131330** | 2.72 |
|  | **AN4324** | 1.94 | **Afu4g06170** | 6.27 | **AFLA_113790** | 5.05 |
|  | **AN10295** | - | **Afu2g14350** | 1.84 | **AFLA_105310** | - |
|  | **AN10334** | - | **Afu2g00360** | -1.09 | **AFLA_083510** | -1.14 |
|  | **AN4773** | 1.29 |  |  |  |  |
|  | **AN4821** | - | **Afu8g01150** | -1.02 | **AFLA_101970** | - |
| *thiA* | **AN10492** | - | **Afu6g08350** | 1.34 | **AFLA_037170** | -1.50 |
|  | **AN7971** | -1.09 |  |  |  |  |
|  | **AN11003** | - | **Afu8g07360** | - | **AFLA_062330** | -1.93 |
|  | **AN11073** | - |  |  | **AFLA_136880** | -2.19 |
|  | **AN4788** | -1.17 | **Afu3g06770** | - | **AFLA_100950** | - |
|  | **AN11793** | - | **Afu8g07000** | -3.21 | **AFLA_009490** | -1.01 |
| *fcr1* | **AN4861** | 1.23 | **Afu3g07670** | -1.12 | **AFLA_100080** | - |
|  | **AN4878** | 1.36 | **Afu3g11170** | 1.39 | **AFLA_031450** | - |
| *btf3* | **AN12485** | - | **Afu1g09130** | 1.47 | **AFLA_088390** | 2.07 |
| *metZ* | **AN5218** | -1.16 | **Afu6g07530** | - | **AFLA_087810** | -1.07 |
| *zfpA* | **AN1500** | 1.09 | **Afu8g05010** | 2.32 | **AFLA_078920** | 4.44 |
|  | **AN1824** | - | **Afu5g14390** | 1.10 | **AFLA_097680** | -1.26 |
|  | **AN1906** | - | **Afu6g07560** | 1.80 | **AFLA_036190** | 1.10 |
| *aslA* | **AN5583** | 1.87 | **Afu4g11480** | 6.58 | **AFLA_027460** | 1.78 |
| *rfeF* | **AN2012** | 2.72 | **Afu4g10200** | 2.84 |  |  |
| *htfA* | **AN2020** | - | **Afu4g10110** | 1.16 | **AFLA_026100** | - |
|  | **AN5775** | 1.90 | **Afu6g06530** | 1.83 | **AFLA_037760** | 2.87 |
| *amdA* | **AN2270** | - | **Afu5g06410** | 1.14 | **AFLA_048870** | -1.29 |
| *stuA* | **AN5836** | 4.03 | **Afu2g07900** | 5.61 | **AFLA_046990** | 2.32 |
|  | **AN5849** | 5.89 | **Afu2g08040** | 5.90 |  |  |
|  | **AN5870** | -1.05 | **Afu2g11460** | - | **AFLA_134320** | 1.07 |
| *steA* | **AN2290** | - | **Afu5g06190** | 3.22 | **AFLA_048650** | 1.14 |
|  | **AN2667** | - | **Afu8g01990** | 2.14 | **AFLA_097920** | -1.59 |
| *fkh1/2* | **AN2854** | - | **Afu3g11960** | -1.64 | **AFLA_030600** | -1.45 |
|  | **AN2957** | -2.16 |  |  | **AFLA_017040** | -1.03 |
| *areB* | **AN6221** | -1.69 | **Afu2g13380** | - | **AFLA_136100** | - |
|  | **AN3024** | - | **Afu3g08880** | -2.96 | **AFLA_085880** | -4.19 |
| *nsdD* | **AN3152** | - | **Afu3g13870** | 2.78 | **AFLA_020210** | 1.68 |
|  | **AN3154** | -1.06 | **Afu3g13920** | 1.45 | **AFLA_020130** | 1.21 |
|  | **AN3217** | - | **Afu4g01010** | - | **AFLA_024580** | -1.19 |
|  | **AN3224** | - | **Afu4g00950** | -1.05 | **AFLA_024470** | - |
| *regA* | **AN3391** | 2.99 | **Afu1g17640** | -3.64 | **AFLA_073870** | -1.45 |
|  | **AN6715** | -1.75 | **Afu7g05620** | - | **AFLA_076560** | - |
|  | **AN3900** | - | **Afu5g06460** | - | **AFLA_048920** | -1.24 |
|  | **AN6790** | -1.15 |  |  | **AFLA_013200** | 1.02 |
|  | **AN4013** | - | **Afu1g04110** | -1.07 | **AFLA_028760** | -1.30 |
| *amdR* | **AN4035** | - | **Afu1g03860** | - | **AFLA_028560** | -1.06 |
|  | **AN7061** | -3.95 |  |  |  |  |
| *ace2* | **AN4873** | - | **Afu3g11250** | -1.85 | **AFLA_031400** | - |
|  | **AN7073** | 5.16 |  |  | **AFLA_059960** | 4.33 |
|  | **AN7343** | 3.41 |  |  |  |  |
|  | **AN5048** | 1.48 | **Afu3g12160** | 2.25 | **AFLA_030390** | - |
| *napA* | **AN7513** | 1.81 | **Afu6g09930** | -1.10 | **AFLA_129340** | - |
|  | **AN5405** | - | **Afu6g13680** | 2.03 | **AFLA_008860** | - |
| *devR* | **AN7553** | 1.77 | **Afu2g14800** | 3.25 | **AFLA_131640** | 1.47 |
| *aflR* | **AN8645** | -1.94 |  |  | **AFLA_139360** | -2.99 |
| *crz1* | **AN5726** | - | **Afu1g06900** | - | **AFLA_127920** | 1.57 |
| *anbH1* | **AN7734** | 3.52 | **Afu5g08020** | - | **AFLA_061790** | 1.92 |
| *dbaA* | **AN7896** | 3.80 |  |  |  |  |
|  | **AN7919** | 2.46 | **Afu8g05840** | -2.39 | **AFLA_024040** | - |
|  | **AN5924** | - | **Afu2g10850** | - | **AFLA_043710** | -1.17 |
|  | **AN8111** | -1.55 |  |  | **AFLA_058750** | - |
|  | **AN6396** | - | **Afu1g17240** | - | **AFLA_014270** | -1.81 |
| *azf1* | **AN6503** | - | **Afu6g05160** | 2.32 | **AFLA_054800** | - |
|  | **AN8164** | 1.53 | **Afu5g02880** | 2.23 | **AFLA_005580** | 1.20 |
|  | **AN8177** | -1.04 | **Afu5g03030** | 1.34 | **AFLA_005740** | - |
|  | **AN6747** | - | **Afu7g00652** | -6.11 | **AFLA_096330** | -2.40 |
| *palcA* | **AN8271** | -1.57 | **Afu5g04190** | - | **AFLA_006980** | - |
|  | **AN8355** | -3.25 | **Afu6g00220** | - | **AFLA_050970** | 1.61 |
| *apdR* | **AN8414** | 1.67 |  |  |  |  |
|  | **AN8506** | -1.65 |  |  | **AFLA_049680** | - |
|  | **AN8535** | 1.24 | **Afu5g09740** | - | **AFLA_118300** | 1.00 |
|  | **AN6828** | - | **Afu5g12900** | -2.05 | **AFLA_093810** | - |
|  | **AN6846** | - | **Afu5g12930** | - | **AFLA_093980** | -1.45 |
| *farA* | **AN7050** | - | **Afu4g03960** | 1.02 | **AFLA_041540** | - |
|  | **AN8655** | 2.64 | **Afu8g06460** | 2.25 | **AFLA_123500** | -1.19 |
| *mcmA* | **AN8676** | 1.08 | **Afu6g02110** | - | **AFLA_090910** | - |
| *mtfA* | **AN8741** | 1.51 | **Afu6g02690** | 3.71 | **AFLA_091490** | 2.27 |
| *flbB* | **AN7542** | - | **Afu2g14680** | 1.17 | **AFLA_131490** | - |
|  | **AN7592** | - | **Afu2g15340** | -2.26 | **AFLA_015850** | -1.62 |
| *mcnB* | **AN8858** | 1.30 | **Afu5g05600** | - | **AFLA_048110** | - |
|  | **AN8918** | -3.38 |  |  | **AFLA_129400** | -2.83 |
|  | **AN8949** | 5.67 |  |  |  |  |
| *alcR* | **AN8978** | 4.15 | **Afu5g07510** | - | **AFLA_013240** | - |
|  | **AN9013** | 1.77 | **Afu8g02280** | -2.48 | **AFLA_132200** | - |
| *hsf1* | **AN8035** | - | **Afu5g01900** | -1.14 | **AFLA_025030** | - |
|  | **AN9240** | 3.35 |  |  |  |  |
|  | **AN8894** | - | **Afu8g02640** | 3.76 | **AFLA_122500** | -2.37 |
|  | **AN9328** | 1.03 |  |  |  |  |
| *hacA* | **AN9397** | 4.00 | **Afu3g04070** | 3.12 | **AFLA_089270** | 1.11 |
|  | **AN9117** | - | **Afu7g01890** | 2.03 | **AFLA_071050** | - |
| *amdX* | **AN9492** | 1.77 | **Afu2g17220** | - | **AFLA_002290** | 1.58 |
| *atfB* |  |  | **Afu5g12960** | 1.53 | **AFLA_094010** | -6.01 |
|  |  |  |  |  | **AFLA_080270** | 1.17 |
|  |  |  |  |  | **AFLA_096320** | -2.84 |
|  |  |  | **Afu7g04811** | 3.98 | **AFLA_074180** | -1.49 |
|  |  |  | **Afu3g00490** | 1.56 | **AFLA_059360** | 2.05 |
|  |  |  | **Afu5g01460** | -2.04 | **AFLA_038900** | -3.28 |
|  |  |  | **Afu1g04140** | -1.01 | **AFLA_028840** | 1.05 |
|  |  |  | **Afu1g03775** | - | **AFLA_028410** | -1.02 |
| *aro90* |  |  |  |  | **AFLA_015920** | -1.25 |
|  |  |  | **Afu1g01240** | 1.24 | **AFLA_124010** | -2.44 |
|  |  |  | **Afu3g01510** | -1.75 | **AFLA_105530** | -2.27 |
|  |  |  | **Afu1g15850** | -1.93 | **AFLA_083560** | 2.61 |
|  |  |  | **Afu4g03670** | 2.41 | **AFLA_041330** | -2.22 |
|  |  |  | **Afu3g03315** | -3.05 | **AFLA_119890** | -2.01 |
|  |  |  | **Afu3g00690** | 4.82 | **AFLA_034610** | -2.28 |
|  |  |  | **Afu3g13600** | -1.27 | **AFLA_110020** | -1.57 |
|  |  |  | **Afu7g06370** | 2.09 | **AFLA_126910** | -2.72 |
|  |  |  |  |  | **AFLA_033480** | -2.26 |
|  |  |  |  |  | **AFLA_036490** | -1.36 |
|  |  |  |  |  | **AFLA_086110** | 3.54 |
|  |  |  |  |  | **AFLA_100300** | -1.44 |
| *acu-15* |  |  |  |  | **AFLA_134920** | -2.36 |
|  |  |  | **Afu3g02750** | - | **AFLA_064370** | -1.08 |
|  |  |  | **Afu5g00950** | -3.18 | **AFLA_049410** | -4.68 |
|  |  |  | **Afu2g04600** | - | **AFLA_035590** | 2.12 |
|  |  |  | **Afu1g17350** | - | **AFLA_097380** | 1.21 |
| *acuK* | **AN7468** | 1.08 | **Afu2g05830** | - |  | - |
|  |  |  |  |  | **AFLA_023420** | 1.01 |
|  |  |  | **Afu7g06500** | - | **AFLA_123770** | -1.81 |
| *nirA* | **AN0098** | -1.21 | **Afu5g12020** | -1.02 | **AFLA_093040** | - |
|  |  |  | **Afu5g10250** | -2.50 | **AFLA_120090** | 2.62 |
| *creA* | **AN6195** | 1.09 | **Afu2g11780** | - | **AFLA_134680** | - |
|  |  |  |  |  | **AFLA_096370** | 2.41 |
| *hasA* |  |  | **Afu3g12890** | 4.91 |  |  |
| *fumR* |  |  | **Afu8g00420** | 1.12 |  |  |
|  |  |  | **Afu2g01190** | 2.97 |  |  |
|  | **AN1962** | - | **Afu4g10820** | - | **AFLA_026850** | -2.34 |
|  |  |  | **Afu2g03050** | -1.13 |  |  |
|  |  |  | **Afu3g03230** | 1.68 |  |  |
|  |  |  | **Afu5g02655** | 1.65 |  |  |
|  |  |  | **Afu7g08320** | -4.53 |  |  |
|  |  |  | **Afu8g07180** | -1.37 |  |  |
| *atfC* |  |  | **Afu1g17360** | -1.66 |  |  |
| *fmpR* |  |  | **Afu6g03430** | 6.87 | **AFLA_064330** | - |
|  |  |  | **Afu1g17460** | -1.12 |  |  |
|  |  |  | **Afu3g02000** | -1.32 |  |  |
|  |  |  | **Afu7g04340** | 2.21 | **AFLA_073640** | - |
|  |  |  | **Afu1g02860** | 4.84 |  |  |
| *gliZ* |  |  | **Afu6g09630** | -2.12 |  |  |
|  | **AN11098** | - | **Afu4g14712** | 1.65 | **AFLA_076320** | -1.18 |
| *nosA* | **AN1848** | 1.57 | **Afu6g07010** | -1.06 | **AFLA_025720** | -1.69 |
|  |  |  |  |  | **AFLA_066630** | -1.30 |
|  |  |  |  |  | **AFLA_093080** | -2.33 |
| The following genes are grouped by their orthogroup | | | | | | |
|  | **AN10906** | - | **Afu5g01662** | -1.49 | **AFLA_023260** | -1.88 |
|  | **AN11169** | -1.98 | **Afu7g01820** | -1.88 | **AFLA_070980** | 1.48 |
|  | **AN10579** | - | **Afu8g06630** | - |  |  |
|  |  |  | **Afu1g16115** | -1.82 |  |  |
|  | **AN0388** | 2.27 | **Afu1g01590** | - | **AFLA_025430** | - |
|  | **AN10550** | -2.10 |  |  |  |  |
| *xprG* | **AN1414** | -1.18 | **Afu1g00580** | 3.58 | **AFLA_012100** | -2.16 |
|  |  |  | **Afu8g04050** | 2.16 |  | - |
|  | **AN2422** | -1.41 | **Afu4g09860** | - | **AFLA_025860** | -1.65 |
|  | **AN2785** | -1.69 |  |  |  |  |
| *farB2* | **AN1425** | - | **Afu1g00410** | - | **AFLA_012010** | -1.49 |
| *farB1* |  |  | **Afu8g04130** | 1.75 |  |  |
