# Supplementary material for: Systematic Dissection of the Evolutionarily Conserved WetA Developmental Regulator across a Genus of Filamentous Fungi
Source: mBio. 2018 Aug 21;9(4):e01130-18. doi: 10.1128/mBio.01130-18 (PMC6106085; doi:10.1128/mBio.01130-18)
Supplement: TABLE S7 [file mbo004184026st7.docx]

Table S7 Secondary Metabolite Cluster – Gene list

| **Cluster** | **Name or Product if known** | **Gene** |
| --- | --- | --- |
| ***A. nidulans*** | |  |
| 1 | Asperfuranone (afo) | AN1029-AN1036, AN11287 |
| 2 | Asperthecin (apt) | AN6000-AN6002 |
| 3 | Aspyridone (asp) | AN8408-AN8715, AN11872 |
| 4 | Austinol (aus) cluster 1 | AN9244-AN9253 |
| 5 | Austinol (aus) cluster 2 | AN8379-AN8384, AN11871 |
| 6 | Derivative of Benzaldehyde1 (dba) and F9775 hybrid cluster 1 | AN7896-AN7903, AN11584 |
| 7 | Derivative of Benzaldehyde1 (dba) and F9775 hybrid cluster 2 | AN7907-AN7916, AN12004 |
| 8 | Emericellamide (eas) cluster | AN2545-AN2549 |
| 9 | inp cluster | AN3490-AN3496 |
| 10 | ivo cluster | AN10573, AN10576 |
| 11 | Microperfuranone (mic) cluster | AN3394-AN3396 |
| 12 | Monodictyphenone (mdp) cluster | AN0146-AN0150, AN10021-AN10023, AN10035, AN10038, AN10044, AN10049 |
| 13 | Penicillin cluster | AN2621-AN2623 |
| 14 | Nidulanin A (nptA) cluster | AN11080, AN11082 |
| 15 | pkb cluster | AN6444-AN6451, AN11922 |
| 16 | pkdA cluster | AN0523-AN0533 |
| 17 | pkf cluster | AN3225-AN3230 |
| 18 | pkg cluster | AN7070-AN7075, AN10884, AN10889 |
| 19 | pkh cluster | AN2030-AN2038 |
| 20 | pki cluster | AN3379-AN3386 |
| 21 | Sterigmatocystin (stc) cluster | AN7804-AN7824, AN12089-AN12090, AN11013, AN11017, AN11021 |
| 22 | Terriquinone (tdi) cluster | AN8513-AN8514, AN8516, AN8518, AN8520 |
| 23 | xptA-containing cluster | AN6784-AN6791, AN11527, AN11907 |
| 24 | xptB-containing cluster | AN7999, AN9467, AN12402, AN12431 |
| 25 | . | AN0015-AN0029, AN10005 |
| 26 | sidC cluster | AN0607-AN0609 |
| 27 | . | AN2346-AN2347, AN10289, AN10295, AN12125 |
| 28 | . | AN2396-AN2400, AN2402, AN2405-AN2407, , AN10297, AN10300, AN11337, AN12115-AN12116, AN12119 |
| 29 | . | AN3375, AN10396, AN10401 |
| 30 | . | AN3911, AN10486, AN10491 |
| 31 | . | AN8256-AN8259, AN8261-AN8263, AN11064, AN11067, AN11071, AN12204-AN12206 |
| 32 | . | AN9218-ANAN9220, AN11191, AN11198-AN11199 |
| 33 | . | AN8229, AN8231-AN8235, AN11055-AN11056, AN11060-AN11061, AN123440AN12345 |
| 34 | . | AN1242-AN1247 |
| 35 | . | AN1592-AN1599 |
| 36 | . | AN1678-AN1681 |
| 37 | . | AN1784-AN1787 |
| 38 | . | AN1792 |
| 38 | . | AN1793-AN1796 |
| 39 | . | AN2057-2060, AN2062-AN2068, AN11861-AN11863 |
| 40 | . | AN2921-AN2924 |
| 41 | . | AN3252-AN3257 |
| 42 | . | AN3269-AN3273, AN3275-AN3287, AN10388-AN10389, AN10402, AN12188 |
| 43 | . | AN3605-AN3609, AN3611-AN3612, AN10430, AN10447 |
| 44 | . | AN4823-AN4827, AN9445-AN9446, AN10600, AN11232 |
| 45 | . | AN5314-AN5318 |
| 46 | . | AN5608-AN5610, AN9473-AN9474, AN10720 |
| 47 | . | AN6234-AN6236 |
| 48 | . | AN6431-AN6437, AN10816 |
| 49 | . | AN7080-AN7081, AN7083-AN7084, AN7086, AN10886-AN10887, AN12290-AN11291 |
| 50 | mirC | AN7485-AN7493 |
| 51 | . | AN7836, AN7839, AN11024, AN12330-AN12331 |
| 52 | . | AN7872-AN7881, AN7883-AN7884, AN11028, AN11031, AN11582 |
| 53 | . | AN8105-AN8106, AN8108, AN8110-AN8112, AN11037, AN11049, AN11941-AN11942 |
| 54 | . | AN8139-AN8144, AN12440 |
| 55 | wA | AN8209, AN12403-AN12404 |
| 56 | . | AN11063, AN11069 |
| 57 | . | AN8495-AN8504 |
| 58 | . | AN8905-AN8910, AN12240 |
| 59 | . | AN9002-AN9007 |
| 60 | . | AN9129-AN9130 |
| 61 | . | AN9223-AN9227, AN9230-AN9234, AN11193-AN11194, AN11201-AN11202 |
| 62 | . | AN9292-AN9294, AN11820-AN11821 |
| 63 | . | AN9313-AN9314 |
| 64 | . | AN9179-AN9181, AN9183-AN9185, AN11190, AN11195 |
| 65 | . | AN2553-AN2557, AN10319-AN10320, AN10325-AN10326, AN11348 |
| 66 | . | AN0653-AN0660, AN11278 |
| 67 | . | AN0042-AN0043 |
| ***A. fumigatus*** | |  |
| 1 | . | Afu1g00970, Afu1g00980, Afu1g00990, Afu1g01000, Afu1g01010 |
| 2 | Nidulanin-like | Afu1g10270, Afu1g10280, Afu1g10295, Afu1g10319, Afu1g10320, Afu1g10330, Afu1g10340, Afu1g10350, Afu1g10360, Afu1g10370, Afu1g10380, Afu1g10390 |
| 3 | Ferricrocin | Afu1g17190, Afu1g17200 |
| 4 | Fusarielin-like | Afu1g17710, Afu1g17720, Afu1g17723, Afu1g17725, Afu1g17730, Afu1g17740 |
| 5 | . | Afu2g01260, Afu2g01270, Afu2g01280, Afu2g01290, Afu2g01300, Afu2g01310, Afu2g01320, Afu2g01330, Afu2g01340, Afu2g01350, Afu2g01360, Afu2g01370, Afu2g01380, Afu2g01390, Afu2g01400, Afu2g01410 |
| 6 | . | Afu2g05700, Afu2g05710, Afu2g05720, Afu2g05730, Afu2g05740, Afu2g05750, Afu2g05760, Afu2g05770, Afu2g05780, Afu2g05790, Afu2g05800, Afu2g05810, Afu2g05820, Afu2g05830, Afu2g05840, Afu2g05850 |
| 7 | DHN melanin spore pigment | Afu2g17530, Afu2g17540, Afu2g17550, Afu2g17560, Afu2g17580, Afu2g17600 |
| 8 | Fumigaclavine | Afu2g17960, Afu2g17970, Afu2g17980, Afu2g17990, Afu2g18000, Afu2g18010, Afu2g18020, Afu2g18030, Afu2g18040, Afu2g18050, Afu2g18060 |
| 9 | . | Afu3g01400, Afu3g01410, Afu3g01420, Afu3g01430, Afu3g01440, Afu3g01450, Afu3g01460, Afu3g01470, Afu3g01480 |
| 10 | . | Afu3g02450, Afu3g02460, Afu3g02470, Afu3g02480, Afu3g02500, Afu3g02520, Afu3g02530, Afu3g02540, Afu3g02550, Afu3g02560, Afu3g02570, Afu3g02580, Afu3g02590, Afu3g02600, Afu3g02610, Afu3g02620, Afu3g02630, Afu3g02640, Afu3g02650, Afu3g02670, Afu3g02680, Afu3g02700, Afu3g02710, Afu3g02720, Afu3g02740, Afu3g02750, Afu3g02760 |
| 11 | Fusarine C | Afu3g03390, Afu3g03400, Afu3g03410, Afu3g03420, Afu3g03430, Afu3g03440 |
| 12 | . | Afu3g03520, Afu3g03530, Afu3g03540, Afu3g03550, Afu3g03560, Afu3g03570, Afu3g03580, Afu3g03590, Afu3g03600, Afu3g03610, Afu3g03620, Afu3g03630 |
| 13 | HAS | Afu3g12890, Afu3g12900, Afu3g12910, Afu3g12920, Afu3g12930, Afu3g12940, Afu3g12950, Afu3g12960 |
| 14 | . | Afu3g13600, Afu3g13610, Afu3g13620, Afu3g13630, Afu3g13640, Afu3g13650, Afu3g13660, Afu3g13670, Afu3g13680, Afu3g13690, Afu3g13700, Afu3g13710, Afu3g13720, Afu3g13730 |
| 15 | . | Afu3g14690, Afu3g14700, Afu3g14710, Afu3g14720, Afu3g14730, Afu3g14740, Afu3g14750, Afu3g14760, Afu3g14770 |
| 16 | . | Afu3g15250, Afu3g15260, Afu3g15270, Afu3g15280, Afu3g15290, Afu3g15300 |
| 17 | Endocrocin | Afu4g00210, Afu4g00220, Afu4g00225, Afu4g00230 |
| 18 | Trypacidin | Afu4g14460, Afu4g14470, Afu4g14480, Afu4g14490, Afu4g14500, Afu4g14510, Afu4g14520, Afu4g14530, Afu4g14540, Afu4g14550, Afu4g14560, Afu4g14570, Afu4g14580 |
| 19 | helvolic acid | Afu4g14770, Afu4g14780, Afu4g14790, Afu4g14800, Afu4g14810, Afu4g14820, Afu4g14830, Afu4g14840, Afu4g14850 |
| 20 | . | Afu5g10120, Afu5g10130, Afu5g10140, Afu5g10150, Afu5g10160, Afu5g10170, Afu5g10180, Afu5g10190, Afu5g10200, Afu5g10210, Afu5g10220 |
| 21 | . | Afu5g12700, Afu5g12710, Afu5g12720, Afu5g12730, Afu5g12740, Afu5g12750, Afu5g12760, Afu5g12770, Afu5g12780, Afu5g12790 |
| 22 | fumisoquin | Afu6g03430, Afu6g03440, Afu6g03450, Afu6g03460, Afu6g03470, Afu6g03480, Afu6g03490 |
| 23 | . | Afu6g08540, Afu6g08550, Afu6g08560, Afu6g08570, Afu6g08580 |
| 24 | . | Afu6g09580, Afu6g09590, Afu6g09600, Afu6g09610, Afu6g09620 |
| 25 | gliotoxin | Afu6g09630, Afu6g09640, Afu6g09650, Afu6g09660, Afu6g09670, Afu6g09680, Afu6g09690, Afu6g09700, Afu6g09710, Afu6g09720, Afu6g09730, Afu6g09740 |
| 26 | Fumiquinozalines | Afu6g12040, Afu6g12050, Afu6g12060, Afu6g12070, Afu6g12080 |
| 27 | Pyripyropene A | Afu6g13920, Afu6g13930, Afu6g13940, Afu6g13945, Afu6g13950, Afu6g13970, Afu6g13980, Afu6g13990, Afu6g14000 |
| 28 | neosartoricin | Afu7g00120, Afu7g00130, Afu7g00150, Afu7g00160, Afu7g00170, Afu7g00180 |
| 29 | Fumitremorgin | Afu8g00170, Afu8g00190, Afu8g00200, Afu8g00210, Afu8g00220, Afu8g00230, Afu8g00240, Afu8g00250, Afu8g00260 |
| 30 | Fumagillin and pseurotin | Afu8g00370, Afu8g00380, Afu8g00390, Afu8g00400, Afu8g00410, Afu8g00420, Afu8g00430, Afu8g00440, Afu8g00460, Afu8g00470, Afu8g00480, Afu8g00490, Afu8g00500, Afu8g00510, Afu8g00520, Afu8g00530, Afu8g00540, Afu8g00550, Afu8g00560, Afu8g00570, Afu8g00580 |
| 31 | . | Afu8g01630, Afu8g01640 |
| 32 | . | Afu8g02350, Afu8g02360, Afu8g02380, Afu8g02390, Afu8g02400, Afu8g02410, Afu8g02420, Afu8g02430, Afu8g02440, Afu8g02450, Afu8g02460 |
| 33 | . | Afu5g00100, Afu5g00110, Afu5g00120, Afu5g00130, Afu5g00135 |
| ***A. flavus*** |  |  |
| 1 | . | AFLA_125760, AFLA_125770, AFLA_125780 |
| 2 | . | AFLA_126710, AFLA_126720, AFLA_126730, AFLA_126740 |
| 3 | . | AFLA_126970, AFLA_126980, AFLA_126990, AFLA_127000, AFLA_127010, AFLA_127020, AFLA_127030, AFLA_127040, AFLA_127050, AFLA_127060, AFLA_127070, AFLA_127080, AFLA_127100, AFLA_127110, AFLA_127120, AFLA_127130, AFLA_127140, AFLA_127150, AFLA_127160, AFLA_127170, AFLA_127190 |
| 4 | . | AFLA_128040, AFLA_128050, AFLA_108060 |
| 5 | . | AFLA_128160, AFLA128170 |
| 6 | . | AFLA_053870, AFLA_053880, AFLA_053890, AFLA_053900, AFLA_053910, AFLA_053920, AFLA_053930, AFLA_053940, AFLA_053950 |
| 7 | . | AFLA_054040, AFLA_054050, AFLA_054060, AFLA_054070, AFLA_054080, AFLA_054090, AFLA_054100 |
| 8 | . | AFLA_054180, AFLA_054190, AFLA_054200, AFLA_054210, AFLA_054220, AFLA_054230, AFLA_054240, AFLA_054250, AFLA_054260, AFLA_054270, AFLA_054280, AFLA_054290, AFLA_054300, AFLA_054310, AFLA_054320, AFLA_054330, AFLA_054340, AFLA_054350, AFLA_054360, AFLA_054370 |
| 9 | . | AFLA_008700, AFLA_008710, AFLA_008720, AFLA_008730, AFLA_008740, AFLA_008750, AFLA_008760, AFLA_008770, AFLA_008780, AFLA_008790 |
| 10 | . | AFLA_009980, AFLA_009990, AFLA_010010, AFLA_010020, AFLA_010030, AFLA_010040, AFLA_010050 |
| 11 | . | AFLA_010590, AFLA_010600, AFLA_010610, AFLA_010620, AFLA_010630, AFLA_010640 |
| 12 | . | AFLA_079360, AFLA_079370, AFLA_079380, AFLA_079390, AFLA_079400, AFLA_079410, AFLA_079420, AFLA_079430, AFLA_079440, AFLA_079450, AFLA_07946 |
| 13 | . | AFLA_080470, AFLA_080480, AFLA_080490, AFLA_080500, AFLA_080510, AFLA_080520, AFLA_080530, AFLA_080540, AFLA_080550 |
| 14 | . | AFLA_082050, AFLA_082060, AFLA_082070, AFLA_082080, AFLA_082090, AFLA_082100 |
| 15 | . | AFLA_082150, AFLA_082160, AFLA_082170, AFLA_082180, AFLA_082190, AFLA_082200, AFLA_082210, AFLA_082220, AFLA_082230 |
| 16 | . | AFLA_082430, AFLA_082440, AFLA_082450, AFLA_082460, AFLA_082470, AFLA_082480 |
| 17 | . | AFLA_083210, AFLA_083220, AFLA_083230, AFLA_083240, AFLA_083250, AFLA_083260, AFLA_083270 |
| 18 | . | AFLA_087810, AFLA_087820, AFLA_087830, AFLA_087840, AFLA_087850, AFLA_087860, AFLA_087870, AFLA_087880 |
| 19 | . | AFLA_114820, AFLA_114830, AFLA_114840 |
| 20 | . | AFLA_116140, AFLA_116150, AFLA_116160, AFLA_116170, AFLA_116180, AFLA_116190, AFLA_116200, AFLA_116210, AFLA_116220, AFLA_116230, AFLA_116240, AFLA_116250, AFLA_116260, AFLA_116270, AFLA_116280, AFLA_116290, AFLA_116300, AFLA_116310, AFLA_116320, AFLA_116330 |
| 21 | . | AFLA_116830, AFLA_116840, AFLA_116850, AFLA_116860, AFLA_116870, AFLA_116880, AFLA_116890, AFLA_116900 |
| 22 | . | AFLA_117760, AFLA_117770, AFLA_117780 |
| 23 | . | AFLA_118430, AFLA_118440 |
| 24 | . | AFLA_118820, AFLA_118830, AFLA_118840, AFLA_118850, AFLA_118860, AFLA_118870, AFLA_118880, AFLA_118890, AFLA_118900, AFLA_118910, AFLA_118920, AFLA_118930, AFLA_118940, AFLA_118950, AFLA_118960, AFLA_118970, AFLA_118980, AFLA_118990 |
| 25 | . | AFLA_119080, AFLA_119090, AFLA_119100, AFLA_119110, AFLA_119120 |
| 26 | . | AFLA_119750, AFLA_119760, AFLA_119770, AFLA_119780, AFLA_119790, AFLA_119800, AFLA_119810, AFLA_119820, AFLA_119830, AFLA_119840, AFLA_119850, AFLA_119860, AFLA_119870, AFLA_119880 |
| 27 | . | AFLA_121400, AFLA_121410, AFLA_121420, AFLA_121430, AFLA_121440, AFLA_121450, AFLA_121460, AFLA_121470, AFLA_121480, AFLA_121490, AFLA_121500, AFLA_121510, AFLA_121520, AFLA_121530, AFLA_121540, AFLA_121550, AFLA_121560, AFLA_121570, AFLA_121580, AFLA_121590, AFLA_121600 |
| 28 | . | AFLA_089620, AFLA_089630, AFLA_089640, AFLA_089650, AFLA_089660, AFLA_089670 |
| 29 | . | AFLA_090180, AFLA_090190, AFLA_090200 |
| 30 | . | AFLA_090590, AFLA_090600, AFLA_090610, AFLA_090620, AFLA_090630, AFLA_090640, AFLA_090650, AFLA_090660, AFLA_090670, AFLA_090680, AFLA_090690 |
| 31 | . | AFLA_096330, AFLA_096340, AFLA_096350, AFLA_096360, AFLA_096370, AFLA_096380, AFLA_096390, AFLA_096400, AFLA_096410, AFLA_096420, AFLA_096430 |
| 32 | . | AFLA_096700, AFLA_096710, AFLA_096720, AFLA_096730, AFLA_096740, AFLA_096750, AFLA_096760, AFLA_096770 |
| 33 | . | AFLA_017840, AFLA_017850, AFLA_017860 |
| 34 | . | AFLA_018250, AFLA_018260, AFLA_018270, AFLA_018280, AFLA_018290, AFLA_018300, AFLA_018310, AFLA_018320, AFLA_018330, AFLA_018340, AFLA_018350 |
| 35 | . | AFLA_038570, AFLA_038580, AFLA_038590, AFLA_038600 |
| 36 | . | AFLA_039200, AFLA_039210, AFLA_039220, AFLA_039230, AFLA_039240, AFLA_039250, AFLA_039260, AFLA_039270 |
| 37 | . | AFLA_041590, AFLA_041600, AFLA_041610, AFLA_041620 |
| 38 | . | AFLA_042330, AFLA_042340, AFLA_042350, AFLA_042360, AFLA_042370 |
| 39 | . | AFLA_045490, AFLA_045500, AFLA_045510 |
| 40 | . | AFLA_100270, AFLA_100280, AFLA_100290, AFLA_100300, AFLA_100310, AFLA_100320, AFLA_100330, AFLA_100340, AFLA_100350, AFLA_100360, AFLA_100370, AFLA_100380, AFLA_100390, AFLA_100400, AFLA_100410, AFLA_100420 |
| 41 | . | AFLA_101690, AFLA_101700, AFLA_101710, AFLA_101720, AFLA_101730, AFLA_101740, AFLA_101750, AFLA_101760 |
| 42 | . | AFLA_102110, AFLA_102120, AFLA_102130, AFLA_102140, AFLA_102150, AFLA_102160 |
| 43 | . | AFLA_102480, AFLA_102490, AFLA_102500, AFLA_102510, AFLA_102520, AFLA_102530, AFLA_102540, AFLA_102550 |
| 44 | . | AFLA_064240, AFLA_064250, AFLA_064260, AFLA_064270, AFLA_064280, AFLA_064290, AFLA_064300, AFLA_064310, AFLA_064320, AFLA_064330, AFLA_064340 |
| 45 | . | AFLA_064440, AFLA_064450, AFLA_064460, AFLA_064470, AFLA_064480, AFLA_064490, AFLA_064500, AFLA_064510, AFLA_064520, AFLA_064530, AFLA_064540, AFLA_064550, AFLA_064560, AFLA_064570, AFLA_064580, AFLA_064590, AFLA_064600, AFLA_064610, AFLA_064620, AFLA_064630, AFLA_064640 |
| 46 | . | AFLA_066700, AFLA_066710, AFLA_066720, AFLA_066730 |
| 47 | . | AFLA_066840, AFLA_066850, AFLA_066860, AFLA_066870, AFLA_066880, AFLA_066890, AFLA_066900, AFLA_066910, AFLA_066920, AFLA_066930, AFLA_066940, AFLA_066950, AFLA_066960, AFLA_066970 |
| 48 | . | AFLA_069330, AFLA_069340, AFLA_069350 |
| 49 | . | AFLA_070860, AFLA_070870, AFLA_070880, AFLA_070890, AFLA_070900, AFLA_070910, AFLA_070920 |
| 50 | . | AFLA_002890, AFLA_002900, AFLA_002910, AFLA_002920, AFLA_002930 |
| 51 | . | AFLA_004280, AFLA_004290, AFLA_004300 |
| 52 | . | AFLA_004430, AFLA_004440, AFLA_004450 |
| 53 | . | AFLA_005270, AFLA_005280, AFLA_005290, AFLA_005300, AFLA_005310, AFLA_005320, AFLA_005330, AFLA_005340, AFLA_005350, AFLA_005360, AFLA_005370, AFLA_005380, AFLA_005390, AFLA_005400, AFLA_005410 |
| 54 | . | AFLA_005440, AFLA_005450 |
| 55 | . | AFLA_006100, AFLA_006110, AFLA_006120, AFLA_006130, AFLA_006140, AFLA_006150, AFLA_006160, AFLA_006170, AFLA_006180, AFLA_006190, AFLA_006200, AFLA_006210, AFLA_006220, AFLA_006230, AFLA_006240 |
| 56 | . | AFLA_006800, AFLA_006810, AFLA_006820, AFLA_006830, AFLA_006840, AFLA_006850, AFLA_006860, AFLA_006870, AFLA_006880, AFLA_006890, AFLA_006900, AFLA_006910, AFLA_006920 |
| 57 | . | AFLA_135430, AFLA_135440, AFLA_135450, AFLA_135460, AFLA_135470, AFLA_135480, AFLA_135490 |
| 58 | . | AFLA_137780, AFLA_137790, AFLA_137800, AFLA_137810, AFLA_137820, AFLA_137830, AFLA_137840, AFLA_137850, AFLA_137860, AFLA_137870, AFLA_137880, AFLA_137890 |
| 59 | . | AFLA_139150, AFLA_139160, AFLA_139170, AFLA_139180, AFLA_139190, AFLA_139200, AFLA_139210, AFLA_139220, AFLA_139230, AFLA_139250, AFLA_139260, AFLA_139280, AFLA_139300, AFLA_139310, AFLA_139320, AFLA_139330, AFLA_139340, AFLA_139360, AFLA_139370, AFLA_139380, AFLA_139390, AFLA_139400, AFLA_139410, AFLA_139420, AFLA_139430, AFLA_139440 |
| 60 | . | AFLA_139590, AFLA_139600, AFLA_139610, AFLA_139620, AFLA_139630, AFLA_139640, AFLA_139650, AFLA_139660, AFLA_139670 |
| 61 | . | AFLA_022840, AFLA_022850, AFLA_022860, AFLA_022870, AFLA_022880, AFLA_022990, AFLA_023000, AFLA_023010, AFLA_023020, AFLA_023030, AFLA_023040, AFLA_023050, AFLA_023060, AFLA_023070, AFLA_023080 |
| 62 | . | AFLA_027200, AFLA_027210, AFLA_027220, AFLA_027230, AFLA_027240, AFLA_027250, AFLA_027260 |
| 63 | . | AFLA_028710, AFLA_028720, AFLA_028730, AFLA_028740, AFLA_028750, AFLA_028760 |
| 64 | . | AFLA_105000, AFLA_105010, AFLA_105020, AFLA_105030, AFLA_105040, AFLA_105050, AFLA_105060, AFLA_105070, AFLA_105080 |
| 65 | . | AFLA_105120, AFLA_105130, AFLA_105140, AFLA_105150, AFLA_105160, AFLA_105170, AFLA_105180, AFLA_105190 |
| 66 | . | AFLA_105370, AFLA_105380, AFLA_105390, AFLA_105400, AFLA_105410, AFLA_105420, AFLA_105430, AFLA_105440, AFLA_105450, AFLA_105460, AFLA_105470, AFLA_105480, AFLA_105490, AFLA_105500, AFLA_105510, AFLA_105520, AFLA_105530, AFLA_105540 |
| 67 | . | AFLA_107100, AFLA_107110, AFLA_107120, AFLA_107130, AFLA_107140, AFLA_107150 |
| 68 | . | AFLA_108540, AFLA_108550, AFLA_108560, AFLA_108570, AFLA_108580 |
| 69 | . | AFLA_109380, AFLA_109390, AFLA_109400, AFLA_109410, AFLA_109420, AFLA_109430, AFLA_109440 |
| 70 | . | AFLA_112780, AFLA_112790, AFLA_112800, AFLA_112810, AFLA_112820, AFLA_112830, AFLA_112840, AFLA_112850, AFLA_112860, AFLA_112870, AFLA_112880, AFLA_112890, AFLA_112900, AFLA_112910, AFLA_112920 |
| 71 | . | AFLA_059950, AFLA_059960, AFLA_059970, AFLA_059980, AFLA_059990, AFLA_060000, AFLA_060010, AFLA_060020 |
| 72 | . | AFLA_060650, AFLA_060660, AFLA_060670, AFLA_060680 |
| 73 | . | AFLA_062430, AFLA_062440, AFLA_062450, AFLA_062460, AFLA_062470, AFLA_062480, AFLA_062490, AFLA_062500, AFLA_062510, AFLA_062520 |
| 74 | . | AFLA_062800, AFLA_062810, AFLA_062820, AFLA_062830, AFLA_062840, AFLA_062850, AFLA_062860, AFLA_062870, AFLA_062880, AFLA_062890, AFLA_062900, AFLA_062910, AFLA_062920, AFLA_062930 |
