# Supplementary material for: Systematic Dissection of the Evolutionarily Conserved WetA Developmental Regulator across a Genus of Filamentous Fungi
Source: mBio. 2018 Aug 21;9(4):e01130-18. doi: 10.1128/mBio.01130-18 (PMC6106085; doi:10.1128/mBio.01130-18)
Supplement: TABLE S8 [file mbo004184026st8.docx]

Table S8 *A. nidulans* WetA ChIP-seq peak-associated genes

| **Peak ID** | **Gene ID** | **Location** | **Annotation** |
| --- | --- | --- | --- |
| 1 | AN4999 | downstream | protein of unknown function |
| 2 | AN12458 | upstream | Has domain(s) with predicted catalytic activity and role in metabolic process |
| 3 | AN7131 | CDS | Putative cytochrome P450 |
| 4 | AN7661 | downstream | Ortholog(s) have RNA polymerase II core promoter proximal region sequence-specific DNA binding, sterol response element binding and transcriptional activator activity, more |
| 4 | AN7662 | upstream | Putative metalloreductase with a predicted role in iron homeostasis |
| 5 | AN7738 | upstream | Has domain(s) with predicted nutrient reservoir activity |
| 5 | AN7739 | upstream | Has domain(s) with predicted chromatin binding activity |
| 6 | AN5990 | upstream | Putative long-chain-fatty-acid-CoA ligase with a predicted role in fatty acid metabolism |
| 6 | AN5991 | downstream | Putative peroxisomal protein (peroxin) with a role in fatty acid utilization |
| 7 | AN6857 | upstream | Ortholog(s) have alpha-1,2-mannosyltransferase activity, role in protein glycosylation and Golgi apparatus localization |
| 7 | AN6858 | upstream | Has domain(s) with predicted RNA polymerase II transcription factor activity, sequence-specific DNA binding, zinc ion binding activity, role in regulation of transcription, DNA-templated and nucleus localization |
| 8 | AN10235 | upstream | Ortholog of A. fumigatus Af293 : Afu2g04400, A. niger CBS 513.88 : An11g03330, A. oryzae RIB40 : AO090003001565, Aspergillus wentii : Aspwe1_0120274 and Aspergillus terreus NIH2624 : ATET_06071 |
| 8 | AN1877 | downstream | Ortholog of A. nidulans FGSC A4 : AN7228, A. fumigatus Af293 : Afu2g17490, A. niger CBS 513.88 : An04g09550, A. oryzae RIB40 : AO090102000061 and Neosartorya fischeri NRRL 181 : NFIA_092910 |
| 9 | AN2342 | downstream | Has domain(s) with predicted role in response to stress and integral component of membrane localization |
| 9 | AN2343 | CDS | Putative nitroreductase |
| 10 | AN3079 | upstream | Secreted thaumatin-like protein |
| 10 | AN3080 | downstream | Ortholog(s) have cytosol localization |
| 11 | AN3042 | upstream | Ortholog of A. fumigatus Af293 : Afu7g00320, A. niger CBS 513.88 : An13g02670, A. oryzae RIB40 : AO090005001314 and Neosartorya fischeri NRRL 181 : NFIA_112860 |
| 12 | AN2989 | upstream | Ortholog(s) have intracellular localization |
| 13 | AN10626 | CDS | Ortholog(s) have intracellular localization |
| 13 | AN10631 | downstream | Ortholog(s) have cytosol, nucleus localization |
| 14 | AN5068 | downstream | Has domain(s) with predicted arylformamidase activity and role in tryptophan catabolic process to kynurenine |
| 14 | AN5069 | upstream | Ortholog of A. nidulans FGSC A4 : AN2683, A. fumigatus Af293 : Afu5g02860, Afu5g14320, A. niger CBS 513.88 : An09g04830, An14g05730 and A. oryzae RIB40 : AO090102000482, AO090001000197 |
| 15 | AN5017 | upstream | Has domain(s) with predicted oxidoreductase activity and role in metabolic process |
| 15 | AN5018 | downstream | protein of unknown function |
| 16 | AN5015 | CDS | Putative conidiation gene |
| 16 | AN5016 | downstream | Ortholog of Aspergillus sydowii : Aspsy1_0147753 |
| 17 | AN5014 | upstream | Ortholog(s) have cytosol, nucleolus localization |
| 18 | AN4936 | downstream | Protein kinase with a role in mRNA cis splicing, via spliceosome |
| 19 | AN8828 | downstream | Component of the TRAPII complex that mediates Rab guanyl-nucleotide exchange factor activity, involved in Golgi vesicle-mediated transport |
| 19 | AN8829 | downstream | Ortholog of A. oryzae RIB40 : AO090009000665, Aspergillus wentii : Aspwe1_0054722, Aspergillus sydowii : Aspsy1_0117087 and Aspergillus terreus NIH2624 : ATET_02810, ATET_09435 |
| 20 | AN8740 | CDS | protein of unknown function |
| 20 | AN8741 | upstream | Putative C2H2 transcription factor involved in regulation of secondary metabolism and morphogenesis |
| 21 | AN8669 | downstream | Dynein light chain |
| 22 | AN8648 | downstream | Has domain(s) with predicted RNA binding, RNA-DNA hybrid ribonuclease activity, RNA-directed DNA polymerase activity, nucleic acid binding activity and role in RNA-dependent DNA replication |
| 22 | AN8649 | downstream | Ortholog of A. fumigatus Af293 : Afu1g04100, A. niger CBS 513.88 : An01g02930, A. oryzae RIB40 : AO090009000519 and Aspergillus wentii : Aspwe1_0037325, Aspwe1_0104267 |
| 23 | AN8643 | upstream | Ortholog(s) have role in cellular response to heat, cellular response to hydrogen peroxide |
| 24 | AN8639 | upstream | Putative alpha,alpha-trehalose-phosphate synthase (UDP-forming) with a predicted role in trehalose biosynthesis |
| 24 | AN8640 | downstream | Ortholog of N. crassa conF, light-induced transcript expressed during conidiation in N. crassa |
| 24 | AN8641 | CDS | Transcript induced by light in in developmentally competent mycelia |
| 24 | AN8642 | upstream | Has domain(s) with predicted oxidoreductase activity |
| 25 | AN7894 | downstream | protein of unknown function |
| 25 | AN7895 | upstream | putative oxidoreductase |
| 26 | AN7950 | upstream | Putative glucan endo-1,3-beta-D-glucosidase with predicted role in degradation of glucans |
| 26 | AN7951 | upstream | Has domain(s) with predicted DNA binding, RNA polymerase II transcription factor activity, sequence-specific DNA binding, zinc ion binding activity and role in regulation of transcription, DNA-templated, transcription, DNA-templated |
| 27 | AN11938 | CDS | protein of unknown function |
| 27 | AN8017 | upstream | Ortholog(s) have cytosol, mitotic spindle pole body, nucleus localization |
| 27 | AN8018 | downstream | Transcript induced by light in in developmentally competent mycelia |
| 28 | AN8028 | downstream | Has domain(s) with predicted catalytic activity |
| 28 | AN8029 | upstream | Putative plasma membrane high affinity K+ transporter |
| 29 | AN8278 | downstream | Ortholog of Neosartorya fischeri NRRL 181 : NFIA_037840, Aspergillus wentii : Aspwe1_0605622, Aspergillus versicolor : Aspve1_0047287 and Aspergillus clavatus NRRL 1 : ACLA_000900 |
| 29 | AN8279 | upstream | Ortholog of S. cerevisiae Can1p which has arginine transmembrane transporter activity |
| 30 | AN8279 | downstream | Ortholog of S. cerevisiae Can1p which has arginine transmembrane transporter activity |
| 30 | AN8280 | upstream | Putative long-chain-fatty-acid-CoA ligase with a predicted role in fatty acid metabolism |
| 31 | AN4287 | downstream | Ortholog(s) have phosphoenolpyruvate transmembrane transporter activity, role in phosphoenolpyruvate transmembrane import into Golgi lumen and Golgi apparatus localization |
| 31 | AN4288 | upstream | Protein expressed at decreased levels in a hapX mutant versus wild-type |
| 32 | AN4160 | downstream | Has domain(s) with predicted RNA polymerase II transcription cofactor activity, role in regulation of transcription from RNA polymerase II promoter and mediator complex localization |
| 33 | AN4159 | upstream | Putative glutamate-ammonia ligase with a predicted role in glutamate and glutamine metabolism |
| 34 | AN4153 | upstream | Has domain(s) with predicted catalytic activity, pyridoxal phosphate binding activity and role in biosynthetic process |
| 34 | AN4154 | upstream | Has domain(s) with predicted 1-alkyl-2-acetylglycerophosphocholine esterase activity and role in lipid catabolic process |
| 35 | AN4071 | upstream | Ortholog(s) have role in dolichol biosynthetic process, dolichol-linked oligosaccharide biosynthetic process, polyprenol catabolic process, pseudohyphal growth |
| 35 | AN4072 | upstream | Ortholog of A. fumigatus Af293 : Afu1g05490, A. niger CBS 513.88 : An18g04300, A. oryzae RIB40 : AO090009000396, Aspergillus wentii : Aspwe1_0169302 and Aspergillus sydowii : Aspsy1_0057624 |
| 36 | AN10494 | upstream | Has domain(s) with predicted oxidoreductase activity and role in metabolic process |
| 36 | AN10502 | downstream | Has domain(s) with predicted catalytic activity, hydrolase activity, hydrolyzing O-glycosyl compounds activity and role in carbohydrate metabolic process |
| 37 | AN3835 | upstream | Has domain(s) with predicted DNA binding, RNA polymerase II transcription factor activity, sequence-specific DNA binding, zinc ion binding activity and role in regulation of transcription, DNA-templated, transcription, DNA-templated |
| 38 | AN3782 | upstream | Has domain(s) with predicted FAD binding, oleate hydratase activity and role in fatty acid metabolic process |
| 38 | AN3783 | CDS | protein of unknown function |
| 39 | AN3678 | downstream | Ortholog of A. fumigatus Af293 : Afu4g12490, A. niger CBS 513.88 : An01g07920, A. oryzae RIB40 : AO090009000462, Aspergillus wentii : Aspwe1_0033898 and Aspergillus sydowii : Aspsy1_0046608 |
| 39 | AN3679 | upstream | Ortholog(s) have cytosol, nucleus localization |
| 40 | AN10458 | downstream | protein of unknown function |
| 40 | AN3674 | upstream | Has domain(s) with predicted phospholipid binding activity |
| 41 | AN3598 | downstream | Putative peptidyl-prolyl cis-trans isomerase |
| 42 | AN7335 | upstream | Ortholog of A. fumigatus Af293 : Afu2g16510, A. niger CBS 513.88 : An15g06990, A. oryzae RIB40 : AO090102000205, Aspergillus wentii : Aspwe1_0460265 and Aspergillus sydowii : Aspsy1_0169307 |
| 42 | AN7336 | upstream | Ortholog of A. fumigatus Af293 : Afu2g16500, A. niger CBS 513.88 : An15g06925, Aspergillus wentii : Aspwe1_0026944, Aspergillus sydowii : Aspsy1_0054451 and Aspergillus terreus NIH2624 : ATET_02290 |
| 43 | AN7286 | upstream | Ortholog(s) have role in calcium ion import and plasma membrane localization |
| 43 | AN7287 | upstream | Mitochondrial succinate/fumarate antiporter involved in utilization of acetate and other carbon sources |
| 44 | AN7101 | upstream | Ortholog of A. fumigatus Af293 : Afu4g03820, A. niger CBS 513.88 : An14g01070, A. oryzae RIB40 : AO090011000344, Aspergillus wentii : Aspwe1_0115647 and Aspergillus sydowii : Aspsy1_0048914 |
| 44 | AN7102 | downstream | Ortholog of A. fumigatus Af293 : Afu4g03830, A. niger CBS 513.88 : An14g01068, A. oryzae RIB40 : AO090011000343, Aspergillus wentii : Aspwe1_0030332 and Aspergillus sydowii : Aspsy1_0060599 |
| 45 | AN7076 | upstream | protein of unknown function |
| 46 | AN7029 | upstream | Ortholog(s) have role in cellular response to oxidative stress, misfolded or incompletely synthesized protein catabolic process, protein import into peroxisome matrix and mitochondrial inner membrane localization |
| 46 | AN7030 | downstream | Polarisome component required for conidiation and vacuolar fusion |
| 47 | AN10945 | upstream | Ortholog of A. fumigatus Af293 : Afu6g09950, A. niger CBS 513.88 : An11g08010, A. oryzae RIB40 : AO090001000628, Aspergillus wentii : Aspwe1_0121304 and Aspergillus sydowii : Aspsy1_0058487 |
| 47 | AN7513 | upstream | Homologous to bZIP family transcription factor human AP-1 and Aspergillus nidulans AtfA |
| 48 | AN7689 | downstream | protein of unknown function |
| 48 | AN7690 | upstream | Ortholog of A. fumigatus Af293 : Afu2g01610, A. niger CBS 513.88 : An03g04840, A. oryzae RIB40 : AO090701000787, Aspergillus wentii : Aspwe1_0112969 and Aspergillus sydowii : Aspsy1_0058707 |
| 49 | AN10984 | upstream | protein of unknown function |
| 49 | AN11000 | upstream | Ortholog of Aspergillus flavus NRRL 3357 : AFL2T_11759, Neosartorya fischeri NRRL 181 : NFIA_094100, Aspergillus versicolor : Aspve1_0085617 and Aspergillus clavatus NRRL 1 : ACLA_044880 |
| 50 | AN7700 | upstream | Ortholog(s) have role in regulation of fungal-type cell wall biogenesis and cytoplasm localization |
| 51 | AN7734 | upstream | Basic-region helix-loop-helix (bHLH) transcription factor |
| 52 | AN11917 | upstream | Ortholog(s) have fungal-type vacuole localization |
| 52 | AN6403 | upstream | Ortholog of Aspergillus clavatus NRRL 1 : ACLA_055850 and Aspergillus aculeatus ATCC16872 : Aacu16872_061969 |
| 53 | AN12052 | upstream | protein of unknown function |
| 54 | AN6105 | upstream | Has domain(s) with predicted carbon-sulfur lyase activity and role in metabolic process |
| 55 | AN12261 | CDS | protein of unknown function |
| 56 | AN5956 | intron | Has domain(s) with predicted DNA binding, transposase activity and role in DNA integration, transposition, DNA-mediated |
| 57 | AN5893 | upstream | RGS (regulator of G-protein signaling) family member |
| 58 | AN6509 | CDS | protein of unknown function |
| 58 | AN6510 | downstream | Translocase of outer mitochondrial membrane complex |
| 59 | AN11907 | CDS | protein of unknown function |
| 59 | AN6790 | upstream | putative transcription factor |
| 59 | AN6791 | upstream | Putative polyketide synthase |
| 60 | AN11980 | intron | protein of unknown function |
| 60 | AN6804 | upstream | Predicted transporter of the major facilitator superfamily (MFS) |
| 61 | AN11980 | downstream | protein of unknown function |
| 62 | AN9284 | downstream | Ortholog of Aspergillus tubingensis : Asptu1_0059406, Aspergillus versicolor : Aspve1_0129968 and Aspergillus sydowii : Aspsy1_0068838 |
| 62 | AN9285 | CDS | Ortholog of A. fumigatus grg1 |
| 62 | AN9286 | downstream | Alpha-glucuronidase, involved in degradation of xylans |
| 63 | AN1378 | upstream | Ortholog of A. fumigatus Af293 : Afu1g09030, A. niger CBS 513.88 : An08g00540, A. oryzae RIB40 : AO090005001622, Aspergillus wentii : Aspwe1_0024518 and Aspergillus sydowii : Aspsy1_0138364 |
| 63 | AN1379 | upstream | Putative nuclear pore complex protein |
| 64 | AN1319 | upstream | Ortholog(s) have U3 snoRNA binding, U4 snRNA binding activity and role in mRNA splicing, via spliceosome, maturation of SSU-rRNA from tricistronic rRNA transcript (SSU-rRNA, 5.8S rRNA, LSU-rRNA) |
| 64 | AN1320 | upstream | Has domain(s) with predicted serine-type peptidase activity and role in proteolysis |
| 65 | AN1058 | downstream | Ortholog of A. fumigatus Af293 : Afu1g12350, A. oryzae RIB40 : AO090001000294, Aspergillus wentii : Aspwe1_0172072, Aspergillus sydowii : Aspsy1_0053315 and Aspergillus terreus NIH2624 : ATET_00433 |
| 65 | AN1059 | upstream | Carnitine acetyltransferase, required for utilization of acetate as carbon source |
| 66 | AN0933 | upstream | Putative transglycosidase with a predicted role in glucan processing |
| 67 | AN0928 | upstream | Ortholog(s) have role in conidiophore development |
| 68 | AN0709 | upstream | Putative zinc-finger protein |
| 69 | AN0663 | upstream | protein of unknown function |
| 70 | AN0537 | downstream | protein of unknown function |
| 70 | AN0538 | CDS | protein of unknown function |
| 71 | AN0471 | upstream | Putative vacuolar H+/Ca2+ exchanger |
| 71 | AN0472 | downstream | Putative 1,3-beta-glucosidase with a role in carbon starvation-induced autolytic cell wall degradation |
| 72 | AN0363 | upstream | Component of the velvet complex composed of VelB, VeA, and LaeA that coordinates development and secondary metabolism in response to light |
| 73 | AN0286 | downstream | Ortholog of A. fumigatus Af293 : Afu1g02985, A. niger CBS 513.88 : An01g05160, A. oryzae RIB40 : AO090005000790, Aspergillus wentii : Aspwe1_0103349 and Aspergillus sydowii : Aspsy1_0626563 |
| 73 | AN0287 | upstream | Ortholog(s) have cytosol localization |
| 74 | AN0254 | upstream | Has domain(s) with predicted catalytic activity, coenzyme binding activity and role in cellular metabolic process |
| 75 | AN10030 | downstream | Putative alkaline serine protease |
| 75 | AN10040 | upstream | Ortholog of A. fumigatus Af293 : Afu5g09180, A. niger CBS 513.88 : An07g03930, A. oryzae RIB40 : AO090020000514, Aspergillus wentii : Aspwe1_0035291 and Aspergillus terreus NIH2624 : ATET_06551 |
| 76 | AN10045 | downstream | Has domain(s) with predicted nucleic acid binding activity |
| 77 | AN0200 | downstream | Ortholog(s) have cytosol, nucleus localization |
| 77 | AN10034 | upstream | Ortholog(s) have role in inositol metabolic process and cytosol, nuclear envelope localization |
| 78 | AN0168 | downstream | Ortholog of A. fumigatus Af293 : Afu5g11330, A. niger CBS 513.88 : An01g02510, A. oryzae RIB40 : AO090026000707, Aspergillus wentii : Aspwe1_0075145 and Aspergillus sydowii : Aspsy1_0027709 |
| 78 | AN0169 | upstream | protein of unknown function |
| 78 | AN0170 | CDS | thioredoxin |
| 79 | AN0162 | upstream | Has domain(s) with predicted DNA binding, transcription factor activity, sequence-specific DNA binding activity and role in regulation of transcription, DNA-templated |
| 80 | AN0128 | downstream | Ortholog(s) have tRNA methyltransferase activity, role in tRNA methylation, wybutosine biosynthetic process and cytosol, mitochondrion, nucleus localization |
| 80 | AN0129 | upstream | Putative dual-specificity protein tyrosine/serine/threonine phosphatase |
| 81 | AN0098 | upstream | Putative Gal4-type zinc finger protein that regulates expression of genes involved in nitrate assimilation |
| 81 | AN9523 | upstream | Ortholog of A. fumigatus Af293 : Afu5g12040, A. niger CBS 513.88 : An18g02305, A. oryzae RIB40 : AO090120000321, Aspergillus wentii : Aspwe1_0445614 and Aspergillus sydowii : Aspsy1_0054923 |
| 82 | AN0080 | downstream | Putative aldolase with a predicted role in naphthalene degradation |
| 82 | AN0081 | upstream | Beta subunit of a heterotrimeric G protein composed of FadA, SfaD, GpgA and involved in regulation of proliferation and conidiophore development |
| 83 | AN11156 | CDS | Ortholog of Aspergillus glaucus : Aspgl1_0125887 |
| 83 | AN11157 | upstream | Ortholog of A. oryzae RIB40 : AO090026000764, Aspergillus flavus NRRL 3357 : AFL2T_06550, Aspergillus wentii : Aspwe1_0062270 and Aspergillus clavatus NRRL 1 : ACLA_043160 |
| 84 | AN1414 | upstream | p53-like transcription factor that contains a Ndt80-like DNA-binding domain |
| 84 | AN1415 | upstream | protein of unknown function |
| 85 | AN1415 | intron | protein of unknown function |
| 85 | AN1416 | downstream | Has domain(s) with predicted N-acetyltransferase activity, hydrolase activity, hydrolyzing O-glycosyl compounds activity and role in carbohydrate metabolic process |
| 86 | AN1425 | upstream | Putative transcription factor containing a Zn2-Cys6 binuclear cluster domain |
| 87 | AN1427 | upstream | Ortholog(s) have N-acetylglucosamine transmembrane transporter activity, role in N-acetylglucosamine transport and cytoplasm, plasma membrane localization |
| 87 | AN1428 | upstream | Ortholog(s) have N-acetylglucosamine-6-phosphate deacetylase activity |
| 88 | AN1603 | intron | Ortholog of A. fumigatus Af293 : Afu8g05985, Neosartorya fischeri NRRL 181 : NFIA_098760, NFIA_099140, Aspergillus wentii : Aspwe1_0689083 and Aspergillus versicolor : Aspve1_0037404 |
| 89 | AN10220 | upstream | Putatice cytochrome c peroxidase |
| 90 | AN11887 | upstream | Ortholog of A. fumigatus Af293 : Afu4g08780, A. niger CBS 513.88 : An04g03650, A. oryzae RIB40 : AO090023000677, Neosartorya fischeri NRRL 181 : NFIA_107380 and Aspergillus wentii : Aspwe1_0078968 |
| 91 | AN1664 | upstream | Has domain(s) with predicted hydrolase activity |
| 92 | AN1677 | upstream | Short-chain dehydrogenase |
| 93 | AN1918 | upstream | Putative phosphoenolpyruvate carboxykinase with a predicted role in gluconeogenesis and glycolysis |
| 94 | AN1937 | upstream | Regulatory protein involved in conidial development |
| 95 | AN1951 | downstream | Has domain(s) with predicted nucleic acid binding, zinc ion binding activity |
| 96 | AN1952 | upstream | Ortholog of A. fumigatus Af293 : Afu4g13380, A. niger CBS 513.88 : An01g09090, Aspergillus wentii : Aspwe1_0022581, Aspergillus sydowii : Aspsy1_0142528 and Aspergillus terreus NIH2624 : ATET_03221 |
| 97 | AN1958 | downstream | protein of unknown function |
| 97 | AN1959 | upstream | Nuclear protein involved in spore formation and trehalose accumulation |
| 98 | AN10265 | upstream | Ortholog(s) have alpha-1,6-mannosyltransferase activity, role in protein N-linked glycosylation and alpha-1,6-mannosyltransferase complex, endoplasmic reticulum localization |
| 98 | AN10269 | downstream | Has domain(s) with predicted oxidoreductase activity and role in metabolic process |
| 99 | AN2163 | upstream | Nonessential protein with similarity to yeast karyopherin Kap114p |
| 99 | AN2164 | upstream | Ortholog(s) have cytosol, nuclear envelope localization |
| 100 | AN2171 | upstream | Ortholog(s) have cytosol, nucleus localization |
| 101 | AN2300 | upstream | Putative ATP-binding cassette (ABC) transporter of the P-glycoprotein cluster |
| 102 | AN2385 | upstream | Protein with licheninase activity, involved in degradation of glucans: predicted glycosyl phosphatidylinositol (GPI)-anchor |
| 103 | AN2466 | upstream | Has domain(s) with predicted substrate-specific transmembrane transporter activity, transmembrane transporter activity, role in transmembrane transport and integral component of membrane, membrane localization |
| 103 | AN2467 | downstream | Ortholog of A. fumigatus Af293 : Afu6g10340, A. niger CBS 513.88 : An11g01080, A. oryzae RIB40 : AO090023000250, Aspergillus wentii : Aspwe1_0059095 and Aspergillus sydowii : Aspsy1_0125009 |
| 104 | AN2500 | upstream | Putative nicotinamide N-methyltransferase |
| 104 | AN2501 | upstream | Ortholog of A. oryzae RIB40 : AO090012000652, Aspergillus sydowii : Aspsy1_0087107, Aspergillus terreus NIH2624 : ATET_04017 and Aspergillus carbonarius ITEM 5010 : Acar5010_208778 |
| 105 | AN9067 | upstream | Protein identified by two-hybrid interaction with NimA kinase |
| 105 | AN9068 | upstream | Ortholog of A. niger CBS 513.88 : An12g00010, Aspergillus wentii : Aspwe1_0114304, Aspergillus versicolor : Aspve1_0047905 and Aspergillus clavatus NRRL 1 : ACLA_064970 |
| 106 | AN3433 | downstream | Has domain(s) with predicted RNA polymerase II transcription factor activity, sequence-specific DNA binding, zinc ion binding activity, role in regulation of transcription, DNA-templated and nucleus localization |
| 107 | AN3432 | upstream | Aldose 1-epimerase with a predicted role in carbohydrate metabolism |
| 108 | AN10393 | downstream | Ortholog(s) have cytosol localization |
| 108 | AN3361 | upstream | Bacterial rhodopsin family G-protein coupled receptor-like protein |
| 109 | AN3203 | upstream | Putative F-box protein |
| 110 | AN3180 | downstream | Ortholog of Aspergillus versicolor : Aspve1_0051207 and Aspergillus sydowii : Aspsy1_0056131 |
| 111 | AN0378 | CDS | Ortholog of A. niger CBS 513.88 : An01g06440, A. oryzae RIB40 : AO090005001321, Aspergillus versicolor : Aspve1_0085305, Aspve1_0120457 and Aspergillus niger ATCC 1015 : 36035-mRNA |
| 111 | AN3077 | upstream | Has domain(s) with predicted catalytic activity and role in nucleoside metabolic process |
| 112 | AN3074 | upstream | Ortholog of A. nidulans FGSC A4 : AN6578, A. fumigatus Af293 : Afu3g09640, Afu6g04490, A. niger CBS 513.88 : An15g01110 and A. oryzae RIB40 : AO090701000110 |
| 113 | AN2983 | upstream | Putative Rho GTPase-activating protein (Rho-GAP) |
| 114 | AN2980 | upstream | Ortholog(s) have structural constituent of ribosome activity, role in cytoplasmic translation and cytosolic large ribosomal subunit, nucleolus localization |
| 115 | AN2911 | upstream | Basic-region leucine zipper transcription factor |
| 116 | AN2910 | downstream | 5-hydroxyisourate hydrolase |
| 117 | AN5099 | upstream | Ortholog(s) have role in chromatin remodeling and Clr6 histone deacetylase complex I'', Rpd3L-Expanded complex, Rpd3S complex localization |
| 118 | AN5103 | downstream | Ortholog of A. fumigatus Af293 : Afu1g07710, A. niger CBS 513.88 : An07g10393, A. oryzae RIB40 : AO090012001023, Neosartorya fischeri NRRL 181 : NFIA_017020 and Aspergillus versicolor : Aspve1_0139230 |
| 118 | AN5104 | upstream | Has domain(s) with predicted substrate-specific transmembrane transporter activity, transmembrane transporter activity, transporter activity, role in transmembrane transport and integral component of membrane, membrane localization |
| 119 | AN5128 | upstream | Ortholog of A. fumigatus Af293 : Afu1g07430, A. niger CBS 513.88 : An07g09970, A. oryzae RIB40 : AO090012000994, Neosartorya fischeri NRRL 181 : NFIA_017320 and Aspergillus wentii : Aspwe1_0069020 |
| 119 | AN5129 | upstream | 70 kilodalton heat shock protein |
| 120 | AN12180 | CDS | Ortholog of Aspergillus tubingensis : Asptu1_0045895, Aspergillus brasiliensis : Aspbr1_0124629, Aspergillus glaucus : Aspgl1_0031148 and Aspergillus flavus NRRL 3357 : AFL2T_03856 |
| 120 | AN5129 | downstream | 70 kilodalton heat shock protein |
| 120 | AN5130 | downstream | Ortholog(s) have coproporphyrinogen oxidase activity, role in cellular response to hypoxia, heme biosynthetic process and cell surface, cytosol, mitochondrion, nucleus, yeast-form cell wall localization |
| 121 | AN5130 | upstream | Ortholog(s) have coproporphyrinogen oxidase activity, role in cellular response to hypoxia, heme biosynthetic process and cell surface, cytosol, mitochondrion, nucleus, yeast-form cell wall localization |
| 121 | AN5131 | upstream | Ortholog(s) have protein tag activity |
| 122 | AN10651 | upstream | Ortholog(s) have cytosol localization |
| 122 | AN5210 | upstream | Putative pyruvate kinase with a predicted role in gluconeogenesis and glycolysis |
| 123 | AN5756 | intron | protein of unknown function |
| 124 | AN5749 | upstream | Has domain(s) with predicted hydrolase activity and role in metabolic process |
| 125 | AN5709 | downstream | Ortholog of A. fumigatus Af293 : Afu1g06670, A. niger CBS 513.88 : An18g05730, A. oryzae RIB40 : AO090001000467, Aspergillus wentii : Aspwe1_0038290 and Aspergillus sydowii : Aspsy1_0056984 |
| 126 | AN5681 | upstream | Ortholog(s) have ubiquitin-protein transferase activity, role in protein import into peroxisome matrix, protein polyubiquitination and peroxisomal importomer complex, peroxisomal membrane localization |
| 126 | AN5682 | upstream | Ortholog(s) have role in protein glycosylation, protein retention in ER lumen and fungal-type vacuole membrane localization |
| 127 | AN5634 | upstream | Isocitrate lyase, required for utilization of acetate and fatty acids as carbon sources |
| 128 | AN9473 | upstream | Ortholog of A. fumigatus Af293 : Afu4g11270, A. niger CBS 513.88 : An04g05360, A. oryzae RIB40 : AO090003001094, Aspergillus wentii : Aspwe1_0102365 and Aspergillus sydowii : Aspsy1_0148568 |
| 129 | AN5567 | downstream | Ortholog of A. fumigatus Af293 : Afu1g01530, A. oryzae RIB40 : AO090011000484, Neosartorya fischeri NRRL 181 : NFIA_023130, Aspergillus versicolor : Aspve1_0084207 and Aspergillus zonatus : Aspzo1_0155350 |
| 130 | AN5523 | upstream | Putative alpha,alpha-trehalose-phosphate synthase (UDP-forming) with a role in trehalose biosynthesis |
| 130 | AN5524 | downstream | Has domain(s) with predicted hydrolase activity |
| 131 | AN5492 | upstream | Has domain(s) with predicted deaminase activity and role in purine ribonucleoside monophosphate biosynthetic process |
| 131 | AN5493 | upstream | Putative nucleoside transporter |
| 132 | AN5450 | downstream | Ortholog(s) have role in cellular response to drug |
| 133 | AN10671 | downstream | Has domain(s) with predicted oxidoreductase activity, zinc ion binding activity and role in oxidation-reduction process |
| 133 | AN10688 | downstream | protein of unknown function |
| 133 | AN11477 | upstream | Ortholog of Aspergillus sydowii : Aspsy1_0137382 and Aspergillus aculeatus ATCC16872 : Aacu16872_040719 |
| 133 | AN5387 | downstream | Ortholog of A. fumigatus Af293 : Afu6g00360, A. niger CBS 513.88 : An13g01480, An02g11060, An18g00050 and A. oryzae RIB40 : AO090023000455, AO090003001393, AO090001000640 |
| 134 | AN10671 | downstream | Has domain(s) with predicted oxidoreductase activity, zinc ion binding activity and role in oxidation-reduction process |
| 134 | AN10688 | downstream | protein of unknown function |
| 134 | AN11477 | CDS | Ortholog of Aspergillus sydowii : Aspsy1_0137382 and Aspergillus aculeatus ATCC16872 : Aacu16872_040719 |
| 135 | AN5349 | upstream | Has domain(s) with predicted RNA polymerase II transcription factor activity, sequence-specific DNA binding, zinc ion binding activity, role in regulation of transcription, DNA-templated and nucleus localization |
| 135 | AN5350 | upstream | Has domain(s) with predicted role in response to stress and integral component of membrane localization |
