# Supplementary material for: Systematic Dissection of the Evolutionarily Conserved WetA Developmental Regulator across a Genus of Filamentous Fungi
Source: mBio. 2018 Aug 21;9(4):e01130-18. doi: 10.1128/mBio.01130-18 (PMC6106085; doi:10.1128/mBio.01130-18)
Supplement: TABLE S9 [file mbo004184026st9.docx]

Table S9 Genes containing a WRE in their 1.5 kb upstream regions

***A. nidulans***

| Gene ID | Annotation |
| --- | --- |
| AN0009 | Has domain(s) with predicted catalytic activity, hydrolase activity |
| AN0010 | Possible pseudogene, similar to amino acid transporter |
| AN0015 | Has domain(s) with predicted ATP binding, ATPase activity, ATPase activity, coupled to transmembrane movement of substances, nucleoside-triphosphatase activity, nucleotide binding activity and role in transmembrane transport |
| AN0016 | Putative nonribosomal peptide synthase |
| AN0017 | Has domain(s) with predicted copper ion binding activity and role in copper ion homeostasis |
| AN0031 | protein of unknown function |
| AN0033 | Ortholog of A. nidulans FGSC A4 : AN10483, AN2809, AN2115, AN12460, A. fumigatus Af293 : Afu2g00940, Afu2g16560, Afu3g15340, Afu7g00460, Afu7g06360 and A. niger CBS 513.88 : An14g02830, An16g00830 |
| AN0039 | Ortholog of S. cerevisiae : YNL011C, A. fumigatus Af293 : Afu5g12650, A. niger CBS 513.88 : An14g06550, A. oryzae RIB40 : AO090120000392 and Aspergillus wentii : Aspwe1_0131485 |
| AN0044 | Ortholog(s) have role in nucleotide-excision repair, postreplication repair and nucleus, site of double-strand break localization |
| AN0059 | Ortholog(s) have nucleolus localization |
| AN0064 | Ortholog of A. fumigatus Af293 : Afu5g12380, A. niger CBS 513.88 : An14g06840, A. oryzae RIB40 : AO090120000356, Aspergillus wentii : Aspwe1_0447672 and Aspergillus sydowii : Aspsy1_0101454 |
| AN0065 | Ortholog(s) have histone methyltransferase activity (H3-K4 specific) activity |
| AN0066 | Ortholog(s) have isopropylmalate transmembrane transporter activity, malonate(1-) transmembrane transporter activity, oxaloacetate transmembrane transporter activity, sulfate transmembrane transporter activity |
| AN0073 | Ortholog of A. fumigatus Af293 : Afu5g12302, A. niger CBS 513.88 : An01g02110, A. oryzae RIB40 : AO090120000347, Aspergillus wentii : Aspwe1_0170763 and Aspergillus sydowii : Aspsy1_0195064 |
| AN0089 | Putative small GTPase involved in endosomal maturation and vacuolar biogenesis |
| AN0091 | Ortholog(s) have histone methyltransferase activity (H3-K79 specific), nucleosomal histone binding activity |
| AN0092 | Putative methyltransferase with a predicted role in histidine metabolism |
| AN0094 | Has domain(s) with predicted DNA binding, zinc ion binding activity, role in transcription, DNA-templated and nucleus localization |
| AN0096 | Has domain(s) with predicted DNA binding, RNA polymerase II transcription factor activity, sequence-specific DNA binding, nucleic acid binding, zinc ion binding activity |
| AN0101 | Has domain(s) with predicted oxidoreductase activity, acting on the aldehyde or oxo group of donors, NAD or NADP as acceptor activity and role in oxidation-reduction process |
| AN0108 | Ortholog of A. fumigatus Af293 : Afu5g11940, A. niger CBS 513.88 : An18g02440, Aspergillus wentii : Aspwe1_0131348 and Aspergillus sydowii : Aspsy1_0195151 |
| AN0116 | Ortholog(s) have structural constituent of ribosome activity and mitochondrial large ribosomal subunit localization |
| AN0117 | Ortholog(s) have role in cellular response to oxidative stress and cytosol, nucleus localization |
| AN0122 | Has domain(s) with predicted ATP binding, ATP-dependent peptidase activity, nucleoside-triphosphatase activity, nucleotide binding, serine-type endopeptidase activity and role in proteolysis |
| AN0124 | Essential protein kinase |
| AN0127 | Ortholog(s) have phosphatidylinositol-3,5-bisphosphate binding, ubiquitin binding activity |
| AN0131 | Ortholog(s) have Golgi apparatus, endoplasmic reticulum localization |
| AN0132 | Ortholog of A. fumigatus Af293 : Afu5g11630, A. niger CBS 513.88 : An18g03110, A. oryzae RIB40 : AO090026000663, Aspergillus wentii : Aspwe1_0170695 and Aspergillus sydowii : Aspsy1_0195219 |
| AN0135 | Ortholog(s) have role in cellular protein localization, mitotic actomyosin contractile ring assembly and actomyosin contractile ring, cytosol, nucleus, polysome localization |
| AN0136 | protein of unknown function |
| AN0141 | Ortholog(s) have double-stranded DNA 5'-3' exodeoxyribonuclease activity, single-stranded DNA endodeoxyribonuclease activity, tRNA (m5U54) methyltransferase activity and role in double-strand break repair, tRNA modification |
| AN0147 | Flavin-containing monooxygenase |
| AN0148 | C6 zinc finger transcription factor similar to AflR |
| AN0159 | Ortholog of A. fumigatus Af293 : Afu5g11420, A. niger CBS 513.88 : An18g04550, A. oryzae RIB40 : AO090026000696, Aspergillus wentii : Aspwe1_0442980 and Aspergillus sydowii : Aspsy1_0054986 |
| AN0164 | Protein phosphatase |
| AN0172 | Ortholog of A. fumigatus Af293 : Afu5g11310, A. niger CBS 513.88 : An01g02460, A. oryzae RIB40 : AO090026000711, Aspergillus wentii : Aspwe1_0442534 and Aspergillus sydowii : Aspsy1_0027712 |
| AN0179 | putative oxidoreductase |
| AN0184 | Ortholog(s) have Arp2/3 complex binding activity, role in actin cortical patch assembly, actin filament debranching, negative regulation of Arp2/3 complex-mediated actin nucleation and actin cortical patch, cytosol, nucleus localization |
| AN0191 | Ortholog(s) have ATPase activity, tRNA binding activity, role in regulation of transcription from RNA polymerase II promoter, tRNA wobble uridine modification and Elongator holoenzyme complex, cytosol, nucleus localization |
| AN0192 | Putative RNA polymerase subunit 8 |
| AN0196 | Ortholog of A. fumigatus Af293 : Afu5g14200, Afu6g13810, Afu7g05110, Afu8g01720, A. niger CBS 513.88 : An13g02610, An15g02090, An04g09640 and A. oryzae RIB40 : AO090003001444 |
| AN0198 | Ortholog of A. fumigatus Af293 : Afu5g14750, A. niger CBS 513.88 : An14g07220, Aspergillus wentii : Aspwe1_0043650, Aspwe1_0044769 and Aspergillus sydowii : Aspsy1_0040680 |
| AN0201 | Has domain(s) with predicted role in transmembrane transport and integral component of membrane localization |
| AN0202 | Putative zinc-finger transcription factor of the Zn(II)2Cys6 type involved in regulation of arginine catabolism |
| AN0204 | Ortholog(s) have nucleolus localization |
| AN0205 | Putative pantoate-beta-alanine ligase with a predicted role in Coenzyme A and pantothenate biosynthesis |
| AN0206 | Has domain(s) with predicted transferase activity, transferring acyl groups activity and role in metabolic process |
| AN0208 | Has domain(s) with predicted role in transmembrane transport and integral component of membrane localization |
| AN0212 | Ortholog(s) have extracellular region localization |
| AN0226 | Ortholog(s) have protein homodimerization activity, ubiquitin protein ligase activity, ubiquitin-protein transferase activity |
| AN0228 | Ortholog(s) have ATP-dependent 3'-5' DNA helicase activity, ATP-dependent four-way junction helicase activity, DNA replication origin binding and single-stranded DNA binding, more |
| AN0237 | Ortholog(s) have mRNA binding activity and role in maturation of 5.8S rRNA from tricistronic rRNA transcript (SSU-rRNA, 5.8S rRNA, LSU-rRNA), maturation of LSU-rRNA from tricistronic rRNA transcript (SSU-rRNA, 5.8S rRNA, LSU-rRNA) |
| AN0241 | Cu/Zn-superoxide dismutase |
| AN0242 | Ortholog(s) have cytosol localization |
| AN0244 | Putative vacuolar assembly protein with a predicted role in late endosome to vacuole transport via the multivesicular body sorting pathway |
| AN0244-uORF | Conserved upstream open reading frame (uORF) that mediates repression of an AN0244 (vps8) protein reporter in vitro |
| AN0251 | Ortholog of A. fumigatus Af293 : Afu1g03520, A. niger CBS 513.88 : An01g04620, A. oryzae RIB40 : AO090005000747, Neosartorya fischeri NRRL 181 : NFIA_021140 and Aspergillus wentii : Aspwe1_0181100 |
| AN0252 | Putative F1F0-ATPase complex gamma subunit with a predicted role in energy metabolism |
| AN0253 | Putative DNA topoisomerase I |
| AN0254 | Has domain(s) with predicted catalytic activity, coenzyme binding activity and role in cellular metabolic process |
| AN0255 | Ortholog of A. fumigatus Af293 : Afu1g03460, A. niger CBS 513.88 : An01g04670, A. oryzae RIB40 : AO090005000754, Aspergillus wentii : Aspwe1_0105945 and Aspergillus sydowii : Aspsy1_0055077 |
| AN0257 | Putative peroxisomal ATP carrier protein with a predicted role in fatty acid beta-oxidation |
| AN0263 | Predicted mariner transposon-related ORF |
| AN0271 | Putative dUTP pyrophosphatase with a predicted role in pyrimidine metabolism |
| AN0276 | Ortholog of A. fumigatus Af293 : Afu1g02840, A. niger CBS 513.88 : An01g04950, A. oryzae RIB40 : AO090005000773, Aspergillus wentii : Aspwe1_0169585 and Aspergillus sydowii : Aspsy1_0085871 |
| AN0280 | Putative alpha-1,4-glucosidase |
| AN0281 | Putative component of the Septation Initiation Network (SIN) |
| AN0282 | Has domain(s) with predicted nucleic acid binding, nucleotide binding activity |
| AN0286 | Ortholog of A. fumigatus Af293 : Afu1g02985, A. niger CBS 513.88 : An01g05160, A. oryzae RIB40 : AO090005000790, Aspergillus wentii : Aspwe1_0103349 and Aspergillus sydowii : Aspsy1_0626563 |
| AN0288 | Ortholog of A. fumigatus Af293 : Afu1g03000, Neosartorya fischeri NRRL 181 : NFIA_021630, Aspergillus wentii : Aspwe1_0169599 and Aspergillus versicolor : Aspve1_0231328 |
| AN0289 | Ortholog(s) have U2-type spliceosomal complex localization |
| AN0290 | Ortholog(s) have actin filament binding activity and role in barbed-end actin filament capping, filamentous growth, mitotic actomyosin contractile ring contraction |
| AN0292 | Subunit of the SAGA transcriptional regulatory complex |
| AN0309 | Ortholog of A. niger CBS 513.88 : An01g05570, Neosartorya fischeri NRRL 181 : NFIA_022000, Aspergillus versicolor : Aspve1_0048560 and Aspergillus clavatus NRRL 1 : ACLA_031790 |
| AN0317 | Ortholog(s) have ubiquitin binding activity and role in actin cortical patch organization, endocytosis, endoplasmic reticulum unfolded protein response, positive regulation of cytokinesis |
| AN0319 | Ortholog of A. fumigatus Af293 : Afu1g02490, A. niger CBS 513.88 : An01g05680, A. oryzae RIB40 : AO090005000843, Aspergillus wentii : Aspwe1_0049702 and Aspergillus sydowii : Aspsy1_0144752 |
| AN0322 | Has domain(s) with predicted oxidoreductase activity and role in metabolic process |
| AN0334 | Ortholog of A. fumigatus Af293 : Afu1g02340, A. oryzae RIB40 : AO090005000866, Aspergillus wentii : Aspwe1_0169667 and Aspergillus sydowii : Aspsy1_0145773 |
| AN0357 | Putative ubiquinol-cytochrome-c reductase subunit with a predicted role in energy metabolism |
| AN0359 | Putative eIF3b subunit of translation initiation factor 3 (eIF3) |
| AN0360 | Ortholog(s) have cytoplasm, nucleolus localization |
| AN0363 | Component of the velvet complex composed of VelB, VeA, and LaeA that coordinates development and secondary metabolism in response to light |
| AN0366 | Has domain(s) with predicted integral component of membrane localization |
| AN0368 | Has domain(s) with predicted RNA binding, RNA-DNA hybrid ribonuclease activity, RNA-directed DNA polymerase activity, nucleic acid binding activity and role in RNA-dependent DNA replication |
| AN0378 | Ortholog of A. niger CBS 513.88 : An01g06440, A. oryzae RIB40 : AO090005001321, Aspergillus versicolor : Aspve1_0085305, Aspve1_0120457 and Aspergillus niger ATCC 1015 : 36035-mRNA |
| AN0379 | Ortholog of A. fumigatus Af293 : Afu1g01760, A. oryzae RIB40 : AO090005000918, Aspergillus wentii : Aspwe1_0025697 and Aspergillus sydowii : Aspsy1_0115500 |
| AN0381 | Ortholog(s) have unfolded protein binding activity, role in protein folding and chaperonin-containing T-complex, nucleus localization |
| AN0383 | Putative endo-mannanase GH76 family protein |
| AN0384 | Ortholog of A. nidulans FGSC A4 : AN5649, AN1674, AN1268, AN7262, A. fumigatus Af293 : Afu1g01700, Afu2g17000, Afu4g08730, Afu4g13630, Afu1g10012 and A. niger CBS 513.88 : An01g09380, An01g06520, An04g09080, An04g03600 |
| AN0388 | Ortholog(s) have transcription regulatory region DNA binding activity |
| AN0389 | Ortholog of A. fumigatus Af293 : Afu1g01570, A. niger CBS 513.88 : An04g08610, Neosartorya fischeri NRRL 181 : NFIA_023080, Aspergillus wentii : Aspwe1_0169743 and Aspergillus versicolor : Aspve1_0119805 |
| AN0393 | Putative endo-mannanase GH76 family protein |
| AN0396 | Has domain(s) with predicted protein dimerization activity |
| AN0405 | Ortholog of A. fumigatus Af293 : Afu1g05000, Aspergillus wentii : Aspwe1_0037521, Aspergillus sydowii : Aspsy1_0055250 and Aspergillus terreus NIH2624 : ATET_02569 |
| AN0414 | Ortholog of A. fumigatus Af293 : Afu1g04920/sha1, A. niger CBS 513.88 : An01g03970, A. oryzae RIB40 : AO090003000844, Aspergillus wentii : Aspwe1_0037503 and Aspergillus sydowii : Aspsy1_0055258 |
| AN0434 | Ortholog(s) have cytosol, nucleus localization |
| AN0440 | Subunit of the SAGA transcriptional regulatory complex |
| AN0441 | Has domain(s) with predicted zinc ion binding activity |
| AN0451 | Putative C-8 sterol isomerase with a predicted role in sterol metabolism |
| AN0452 | Protein with xyloglucan-specific endoglucanase activity, involved in degradation of xyloglucans |
| AN0454 | Ortholog of A. fumigatus Af293 : Afu1g04440, A. niger CBS 513.88 : An01g03310, A. oryzae RIB40 : AO090003000907, Aspergillus wentii : Aspwe1_0150842 and Aspergillus sydowii : Aspsy1_0086117 |
| AN0457 | protein of unknown function |
| AN0459 | Putative cytochrome P450 |
| AN0460 | Putative F-box protein |
| AN0464 | Has domain(s) with predicted hydrolase activity |
| AN0465 | Ortholog of S. cerevisiae RPS8A and RPS8B |
| AN0472 | Putative 1,3-beta-glucosidase with a role in carbon starvation-induced autolytic cell wall degradation |
| AN0473 | Has domain(s) with predicted role in transmembrane transport and integral component of membrane localization |
| AN0479 | Putative beta-glucosidase with a predicted role in polysaccharide degradation |
| AN0480 | protein of unknown function |
| AN0481 | protein of unknown function |
| AN0483 | Ortholog of Neosartorya fischeri NRRL 181 : NFIA_058180, Aspergillus versicolor : Aspve1_0158177, Aspergillus clavatus NRRL 1 : ACLA_085420 and Aspergillus zonatus : Aspzo1_0018005 |
| AN0487 | Has domain(s) with predicted transferase activity, transferring hexosyl groups activity and role in lipid glycosylation |
| AN0494 | Putative cellobiohydrolase |
| AN0495 | Has domain(s) with predicted amino acid binding, formyltetrahydrofolate deformylase activity, hydroxymethyl-, formyl- and related transferase activity and role in 'de novo' IMP biosynthetic process, biosynthetic process, metabolic process |
| AN0498 | Has domain(s) with predicted role in transmembrane transport and integral component of membrane localization |
| AN0499 | Has domain(s) with predicted chitin binding activity, role in chitin metabolic process and extracellular region localization |
| AN0519 | protein of unknown function |
| AN0528 | Has domain(s) with predicted role in transmembrane transport and integral component of membrane localization |
| AN0530 | Has domain(s) with predicted FAD binding, oxidoreductase activity and role in metabolic process |
| AN0531 | protein of unknown function |
| AN0548 | Ortholog of Aspergillus brasiliensis : Aspbr1_0045481 and Aspergillus terreus NIH2624 : ATET_08579 |
| AN0561 | Ortholog(s) have mitochondrion localization |
| AN0562 | Has domain(s) with predicted catalytic activity and role in metabolic process |
| AN0567 | Putative alcohol oxidase with a predicted role in glycerol metabolism |
| AN0569 | Ortholog of A. niger CBS 513.88 : An08g07480, Aspergillus tubingensis : Asptu1_0026628, Aspergillus brasiliensis : Aspbr1_0504530 and Aspergillus kawachii : Aspka1_0173250 |
| AN0573 | Has domain(s) with predicted intracellular localization |
| AN0574 | Ortholog(s) have glyoxysome localization |
| AN0580 | Ortholog of A. fumigatus Af293 : Afu6g11150, A. niger CBS 513.88 : An08g07580, A. oryzae RIB40 : AO090023000501, Aspergillus wentii : Aspwe1_0027096 and Aspergillus sydowii : Aspsy1_0055387 |
| AN0581 | Ortholog(s) have role in S-adenosylmethionine biosynthetic process and fungal-type vacuole membrane localization |
| AN0592 | Has domain(s) with predicted nucleic acid binding, nucleotide binding activity |
| AN0596 | Putative DNA damage binding protein involved in the DNA damage response |
| AN0611 | protein of unknown function |
| AN0622 | Possible pseudogene |
| AN0623 | Has domain(s) with predicted flavin adenine dinucleotide binding, oxidoreductase activity, acting on CH-OH group of donors activity and role in oxidation-reduction process |
| AN0624 | Ortholog(s) have mitochondrion, nucleus localization |
| AN0633 | Ortholog of A. fumigatus Af293 : Afu1g16980, A. niger CBS 513.88 : An01g09880, A. oryzae RIB40 : AO090005000977, Aspergillus wentii : Aspwe1_0109484 and Aspergillus sydowii : Aspsy1_0055497 |
| AN0634 | Predicted ADP ribosylation factor GTPase |
| AN0649 | Putative long-chain-fatty-acid-CoA ligase with a predicted role in fatty acid metabolism |
| AN0660 | Putative allantoin transporter |
| AN0663 | protein of unknown function |
| AN0664 | Putative 1-phosphatidylinositol-4,5-bisphosphate phosphodiesterase with a predicted role in phospholipid metabolism |
| AN0667 | Putative mannose-6-phosphate isomerase with a predicted role in mannose/mannitol, fructose, and sorbose/sorbitol metabolism |
| AN0673 | Protein with a predicted role in actin assembly |
| AN0677 | Has domain(s) with predicted zinc ion binding activity |
| AN0684 | Ortholog of A. fumigatus Af293 : Afu1g13450, A. niger CBS 513.88 : An08g06520, A. oryzae RIB40 : AO090012000535, Aspergillus wentii : Aspwe1_0171971 and Aspergillus sydowii : Aspsy1_0028405 |
| AN0685 | Ortholog(s) have rRNA (uridine-N3-)-methyltransferase activity, role in rRNA base methylation and cytoplasm localization |
| AN0689 | Transcription factor containing a Zn(II)2-Cys6 binuclear DNA-binding cluster domain |
| AN0695 | Putative snoRNP component with a predicted role in ribosomal RNA pseudouridinylation |
| AN0698 | Ortholog of A. nidulans FGSC A4 : AN9272, A. fumigatus Af293 : Afu1g13610, Afu4g09370, A. niger CBS 513.88 : An08g06690, An07g08670 and A. oryzae RIB40 : AO090012000513 |
| AN0700 | Ortholog(s) have fungal-type vacuole membrane localization |
| AN0703 | Ortholog(s) have protein complex binding activity and role in cellular response to calcium ion, cellular response to zinc ion, early endosome to late endosome transport, regulation of protein complex assembly, vacuolar acidification |
| AN0706 | Ortholog(s) have role in ER to Golgi vesicle-mediated transport, Golgi vesicle docking, SNARE complex assembly and ER to Golgi transport vesicle membrane, Golgi membrane localization |
| AN0708 | Putative pentafunctional AROM polypeptide with 3-dehydroquinate synthase, 3-dehydroquinate dehydratase, shikimate 5-dehydrogenase, shikimate kinase, and EPSP synthase activities |
| AN0710 | Has domain(s) with predicted oxidoreductase activity, zinc ion binding activity and role in oxidation-reduction process |
| AN0715 | Ortholog of A. fumigatus Af293 : Afu1g14070, A. niger CBS 513.88 : An01g11910, A. oryzae RIB40 : AO090012000454, Aspergillus wentii : Aspwe1_0110525 and Aspergillus sydowii : Aspsy1_0028467 |
| AN0716 | Ortholog(s) have phosphatidate cytidylyltransferase activity, role in cardiolipin biosynthetic process and extrinsic component of mitochondrial inner membrane, hyphal cell wall, mitochondrial matrix localization |
| AN0717 | Putative histidinol-phosphate aminotransferase with a predicted role in histidine metabolism |
| AN0727 | Ortholog(s) have role in mRNA cis splicing, via spliceosome and U4/U6 x U5 tri-snRNP complex, mitotic spindle pole body localization |
| AN0732 | Putative transporter of the major facilitator superfamily (MFS) |
| AN0736 | Ortholog of A. nidulans FGSC A4 : AN2423, A. fumigatus Af293 : Afu1g14230, Afu2g13800, A. niger CBS 513.88 : An01g12240, An02g05360 and A. oryzae RIB40 : AO090026000195 |
| AN0765 | Has domain(s) with predicted catalytic activity, coenzyme binding activity and role in cellular metabolic process |
| AN0770 | Ortholog(s) have role in histone H2B ubiquitination, protein processing and Dsc E3 ubiquitin ligase complex, endoplasmic reticulum localization |
| AN0771 | Putative ABC multidrug transporter |
| AN0776 | Ortholog(s) have structural constituent of ribosome activity, role in cellular response to drug, cytoplasmic translation and cell surface, cytosolic large ribosomal subunit localization |
| AN0778 | Has domain(s) with predicted viral capsid localization |
| AN0780 | Ortholog(s) have 2-aminoadipate transaminase activity and cytoplasm, nucleus localization |
| AN0781 | Ortholog of A. fumigatus Af293 : Afu1g14500, A. niger CBS 513.88 : An01g12470, A. oryzae RIB40 : AO090011000360, Aspergillus wentii : Aspwe1_0112044 and Aspergillus sydowii : Aspsy1_0145580 |
| AN0785 | Putative manganese superoxide dismutase |
| AN0786 | Ortholog of Neosartorya fischeri NRRL 181 : NFIA_010870, Aspergillus wentii : Aspwe1_0171813, Aspergillus versicolor : Aspve1_0049042 and Aspergillus clavatus NRRL 1 : ACLA_020880 |
| AN0789 | Ortholog of Aspergillus sydowii : Aspsy1_0053160 |
| AN0791 | protein of unknown function |
| AN0792 | Has domain(s) with predicted metal ion binding, zinc ion binding activity |
| AN0799 | Has domain(s) with predicted chitin synthase activity and role in chitin biosynthetic process |
| AN0800 | protein of unknown function |
| AN0817 | Ortholog(s) have RNA polymerase II transcription factor activity, sequence-specific DNA binding activity |
| AN0818 | Ortholog(s) have mRNA binding, tRNA dihydrouridine synthase activity, role in tRNA modification and cytoplasm, nuclear periphery localization |
| AN0819 | Ortholog(s) have endoplasmic reticulum, plasma membrane localization |
| AN0825 | Ortholog of A. fumigatus Af293 : Afu1g14860, A. niger CBS 513.88 : An06g02310, An01g12970, A. oryzae RIB40 : AO090005001247, Aspergillus wentii : Aspwe1_0485796 and Aspergillus sydowii : Aspsy1_0083958 |
| AN0827 | Ortholog(s) have glucosaminyl-phosphotidylinositol O-acyltransferase activity and role in GPI anchor biosynthetic process, cellular response to drug |
| AN0837 | Protein with similarity to mammalian fragmin |
| AN0841 | protein of unknown function |
| AN0852 | Has domain(s) with predicted integral component of membrane localization |
| AN0859 | protein of unknown function |
| AN0860 | Ortholog of A. fumigatus Af293 : Afu1g15260, A. niger CBS 513.88 : An01g13370, A. oryzae RIB40 : AO090103000449, AO090005001207 and Aspergillus wentii : Aspwe1_0111296 |
| AN0861 | Ortholog of A. fumigatus Af293 : Afu1g15250, A. oryzae RIB40 : AO090005001206, Aspergillus wentii : Aspwe1_0051149, Aspergillus sydowii : Aspsy1_0122516 and Aspergillus terreus NIH2624 : ATET_00827 |
| AN0865 | Ortholog(s) have cytosol, nucleus localization |
| AN0867 | Ortholog of A. nidulans FGSC A4 : AN4122, AN2881, AN10123, A. fumigatus Af293 : Afu1g13860, Afu1g15180, Afu3g11650 and A. niger CBS 513.88 : An08g07010, An02g07440, An01g13480 |
| AN0879 | Ortholog(s) have cytosol, nucleus localization |
| AN0888 | Ortholog of A. fumigatus Af293 : Afu1g15510, A. niger CBS 513.88 : An01g13820, A. oryzae RIB40 : AO090005001158, Neosartorya fischeri NRRL 181 : NFIA_009900 and Aspergillus wentii : Aspwe1_0039986 |
| AN0893 | Putative adenylosuccinate synthase with a predicted role in purine metabolism |
| AN0894 | Putative ATP-dependent RNA helicase |
| AN0895 | Predicted NAD-dependent oxidoreductase |
| AN0901 | Hyphal tip laccase with predicted role in degradation of phenolic substrates |
| AN0907 | Ortholog(s) have structural constituent of ribosome activity and cytosolic small ribosomal subunit, nucleus localization |
| AN0914 | Putative serine/threonine phosphatase |
| AN0918 | Putative ceramide hydroxylase with a predicted role in sphingoglycolipid metabolism |
| AN0919 | Has domain(s) with predicted catalytic activity, orotidine-5'-phosphate decarboxylase activity and role in 'de novo' pyrimidine nucleobase biosynthetic process |
| AN0922 | Ortholog(s) have role in ER to Golgi vesicle-mediated transport, Golgi inheritance, Golgi localization, retrograde vesicle-mediated transport, Golgi to ER and COPI vesicle coat localization |
| AN0924 | Has domain(s) with predicted magnesium ion transmembrane transporter activity, role in magnesium ion transport and integral component of membrane localization |
| AN0925 | Has domain(s) with predicted phosphoric ester hydrolase activity |
| AN0927 | Ortholog(s) have ubiquitin-specific protease activity, role in cellular response to drug, protein deubiquitination and nuclear periphery localization |
| AN0928 | Ortholog(s) have role in conidiophore development |
| AN0933 | Putative transglycosidase with a predicted role in glucan processing |
| AN0936 | Ortholog(s) have threonine-tRNA ligase activity and mitochondrion localization |
| AN0939 | Has domain(s) with predicted catalytic activity and role in metabolic process |
| AN0941 | Protein with alpha-glucosidase activity, predicted role in maltose metabolism |
| AN0950 | Ortholog of Aspergillus versicolor : Aspve1_0036555 and Aspergillus sydowii : Aspsy1_0038433 |
| AN0969 | Putative GNAT-type acetyltransferase |
| AN0982 | Ortholog(s) have role in receptor-mediated endocytosis and actin cortical patch localization |
| AN0984 | Ortholog(s) have nucleus localization |
| AN0989 | Has domain(s) with predicted role in DNA-templated transcription, initiation and transcription factor TFIID complex localization |
| AN0990 | Ortholog(s) have cytosol, mRNA cleavage and polyadenylation specificity factor complex localization |
| AN1001 | Ortholog of A. fumigatus Af293 : Afu1g12770, A. niger CBS 513.88 : An08g05540, A. oryzae RIB40 : AO090012000633, Aspergillus wentii : Aspwe1_0027882 and Aspergillus sydowii : Aspsy1_0191418 |
| AN10011 | Has domain(s) with predicted oxidoreductase activity, oxidoreductase activity, acting on the aldehyde or oxo group of donors, NAD or NADP as acceptor activity and role in oxidation-reduction process |
| AN10014 | protein of unknown function |
| AN10019 | Ortholog(s) have protein serine/threonine kinase activity |
| AN1002 | Ortholog(s) have role in Golgi to plasma membrane transport, endoplasmic reticulum inheritance, establishment or maintenance of cell polarity, exocyst assembly |
| AN10023 | Member of the monodictyphenone (mdp) secondary metabolite biosynthesis gene cluster |
| AN10029 | Ortholog(s) have cytosol, nucleus localization |
| AN10038 | Putative glutathione S transferase |
| AN10040 | Ortholog of A. fumigatus Af293 : Afu5g09180, A. niger CBS 513.88 : An07g03930, A. oryzae RIB40 : AO090020000514, Aspergillus wentii : Aspwe1_0035291 and Aspergillus terreus NIH2624 : ATET_06551 |
| AN10041 | Ortholog of A. niger CBS 513.88 : An07g01780, Neosartorya fischeri NRRL 181 : NFIA_113800, Aspergillus wentii : Aspwe1_0281884 and Aspergillus versicolor : Aspve1_0082092 |
| AN10052 | Ortholog(s) have nucleolus localization |
| AN10061 | F-box protein involved in control of the turnover of kinesin motor KipA |
| AN10074 | Has domain(s) with predicted catalytic activity and role in metabolic process |
| AN10076 | Has domain(s) with predicted myosin binding activity |
| AN10081 | Alpha-ketoglutarate-dependent xanthine dioxygenase involved in the oxidation of xanthine to uric acid |
| AN10098 | Putative zinc-binding oxidoreductase |
| AN10100 | protein of unknown function |
| AN10109 | Ortholog of A. fumigatus Af293 : Afu1g13670, Neosartorya fischeri NRRL 181 : NFIA_011830, Aspergillus wentii : Aspwe1_0040281 and Aspergillus clavatus NRRL 1 : ACLA_021770 |
| AN1011 | Ortholog(s) have Mis6-Sim4 complex, condensed nuclear chromosome kinetochore, cytoplasm localization |
| AN10116 | Ortholog of A. nidulans FGSC A4 : AN2186, A. fumigatus Af293 : Afu1g13980, A. niger CBS 513.88 : An08g07120, An12g09760, An05g02480, An12g10180 and A. oryzae RIB40 : AO090010000355, AO090010000554 |
| AN10117 | Ortholog of A. fumigatus Af293 : Afu1g14050/fbpA, A. oryzae RIB40 : AO090012000463, Aspergillus wentii : Aspwe1_0040217, Aspergillus sydowii : Aspsy1_0086500 and Aspergillus terreus NIH2624 : ATET_00606 |
| AN10119 | Ortholog(s) have poly(A) RNA binding, poly(A)-specific ribonuclease activity, role in nuclear-transcribed mRNA poly(A) tail shortening, postreplication repair and PAN complex, cytosol, nucleus localization |
| AN10120 | Has domain(s) with predicted DNA binding, RNA polymerase II transcription factor activity, sequence-specific DNA binding, zinc ion binding activity and role in regulation of transcription, DNA-templated, transcription, DNA-templated |
| AN10121 | Has domain(s) with predicted nucleic acid binding, zinc ion binding activity |
| AN10123 | Ortholog of A. nidulans FGSC A4 : AN4122, AN2881, AN0867, A. fumigatus Af293 : Afu1g13860, Afu1g15180, Afu3g11650 and A. niger CBS 513.88 : An08g07010, An02g07440, An01g13480 |
| AN1013 | Putative 60S Ribosomal protein L5 |
| AN10136 | Has domain(s) with predicted cation transmembrane transporter activity and role in cation transport, transmembrane transport |
| AN10138 | Predicted dual-specificity protein tyrosine/serine/threonine phosphatase |
| AN10139 | Protein expressed at increased levels in a hapX mutant versus wild-type |
| AN10142 | protein of unknown function |
| AN10147 | Putative pectin lyase with a predicted role in the degradation of pectin |
| AN1015 | Putative phosphorylase with a predicted role in glycogen degradation |
| AN10150 | Putative 1,3-beta-transglucosylase with a predicted role in glucan processing |
| AN10151 | Possible pseudogene, similar to Zn(II)2Cys6 transcription factor |
| AN10152 | Has domain(s) with predicted role in transmembrane transport and integral component of membrane localization |
| AN10156 | Has domain(s) with predicted kinase activity, transferase activity, transferring phosphorus-containing groups activity and role in glycerophospholipid biosynthetic process |
| AN10158 | Ortholog of A. nidulans FGSC A4 : AN7874, A. fumigatus Af293 : Afu1g12200, Afu3g00570, Afu8g02315 and A. niger CBS 513.88 : An08g04520, An06g00280, An16g02300 |
| AN10169 | Short-chain dehydrogenase |
| AN10173 | Ortholog(s) have nucleolus localization |
| AN10183 | Ortholog of A. fumigatus Af293 : Afu1g09520, A. niger CBS 513.88 : An08g01080, A. oryzae RIB40 : AO090012000920, Neosartorya fischeri NRRL 181 : NFIA_016090 and Aspergillus wentii : Aspwe1_0103125 |
| AN10186 | Ortholog(s) have GTPase binding activity, role in ER to Golgi vesicle-mediated transport and Golgi membrane localization |
| AN10188 | Putative guanylate kinase |
| AN10201 | Ortholog of A. fumigatus Af293 : Afu8g04826, A. niger CBS 513.88 : An16g07990, A. oryzae RIB40 : AO090005000664, Neosartorya fischeri NRRL 181 : NFIA_097640 and Aspergillus wentii : Aspwe1_0645178 |
| AN10204 | Ortholog of A. oryzae RIB40 : AO090026000169 and Aspergillus flavus NRRL 3357 : AFL2T_07074 |
| AN10206 | Has domain(s) with predicted role in anaphase-promoting complex-dependent proteasomal ubiquitin-dependent protein catabolic process, regulation of mitotic metaphase/anaphase transition and anaphase-promoting complex localization |
| AN10208 | Subunit 8 of the COP9 signalosome |
| AN10216 | Ortholog of A. fumigatus Af293 : Afu8g05640, A. niger CBS 513.88 : An15g04470, A. oryzae RIB40 : AO090005000578, Aspergillus wentii : Aspwe1_0118846 and Aspergillus sydowii : Aspsy1_0055743 |
| AN10218 | protein of unknown function |
| AN1022 | Ortholog(s) have role in potassium ion transport, proton transport and integral component of mitochondrial inner membrane localization |
| AN10222 | Ortholog(s) have glucose-6-phosphate 1-epimerase activity and cytoplasm, nucleus localization |
| AN10223 | Putative 1-Cys peroxiredoxin |
| AN10224 | Possible role in diphthamide biosynthesis |
| AN10243 | Ortholog(s) have cytoplasm, nucleus localization |
| AN10247 | Ortholog of A. fumigatus Af293 : Afu2g04390, A. oryzae RIB40 : AO090003000225, Aspergillus wentii : Aspwe1_0046123 and Aspergillus terreus NIH2624 : ATET_06072 |
| AN10249 | Ortholog(s) have aminoacyl-tRNA hydrolase activity, role in negative regulation of proteasomal ubiquitin-dependent protein catabolic process and mitochondrial outer membrane localization |
| AN10256 | Has domain(s) with predicted RNA binding, RNA-directed DNA polymerase activity, nucleic acid binding activity and role in DNA integration, RNA-dependent DNA replication |
| AN10265 | Ortholog(s) have alpha-1,6-mannosyltransferase activity, role in protein N-linked glycosylation and alpha-1,6-mannosyltransferase complex, endoplasmic reticulum localization |
| AN10270 | Ortholog of A. fumigatus Af293 : Afu2g15720, A. niger CBS 513.88 : An15g05970, A. oryzae RIB40 : AO090012000253, Neosartorya fischeri NRRL 181 : NFIA_090950 and Aspergillus wentii : Aspwe1_0182342 |
| AN10271 | Ortholog of A. fumigatus Af293 : Afu2g15710, A. niger CBS 513.88 : An15g05960, A. oryzae RIB40 : AO090012000254, Aspergillus clavatus NRRL 1 : ACLA_074660 and Aspergillus niger ATCC 1015 : 200760-mRNA |
| AN10272 | Ortholog of A. fumigatus Af293 : Afu2g15690, Aspergillus wentii : Aspwe1_0170945, Aspergillus clavatus NRRL 1 : ACLA_074640 and Aspergillus zonatus : Aspzo1_0138260 |
| AN1028 | Has domain(s) with predicted RNA polymerase II transcription factor activity, sequence-specific DNA binding, zinc ion binding activity, role in regulation of transcription, DNA-templated and nucleus localization |
| AN10283 | Has domain(s) with predicted FMN binding, iron ion binding, oxidoreductase activity and role in oxidation-reduction process |
| AN10288 | protein of unknown function |
| AN10292 | Ortholog of A. fumigatus Af293 : Afu5g10455, A. niger CBS 513.88 : An14g04320, A. oryzae RIB40 : AO090010000491, Aspergillus wentii : Aspwe1_0038732 and Aspergillus sydowii : Aspsy1_0054224 |
| AN10293 | Ortholog of A. fumigatus Af293 : Afu5g09950, Aspergillus glaucus : Aspgl1_0181943, Aspergillus flavus NRRL 3357 : AFL2T_11694 and Neosartorya fischeri NRRL 181 : NFIA_077320 |
| AN10307 | Has domain(s) with predicted zinc ion binding activity |
| AN10309 | Putative chitin deacetylase |
| AN1031 | Putative efflux pump |
| AN10310 | protein of unknown function |
| AN10316 | Putative RNA polymerase III, large subunit |
| AN1032 | putative oxidoreductase |
| AN10321 | Has domain(s) with predicted role in transmembrane transport and integral component of membrane localization |
| AN1033 | Putative salicylate hydroxylase |
| AN10331 | Has domain(s) with predicted DNA binding, RNA polymerase II transcription factor activity, sequence-specific DNA binding, zinc ion binding activity and role in regulation of transcription, DNA-templated, transcription, DNA-templated |
| AN10336 | Ortholog(s) have acetyltransferase activity, chromatin binding activity |
| AN10337 | Has domain(s) with predicted enzyme regulator activity, role in regulation of protein catabolic process and proteasome complex localization |
| AN10343 | Ortholog(s) have sodium:inorganic phosphate symporter activity, role in phosphate ion transmembrane transport and plasma membrane localization |
| AN10344 | CobW domain-containing protein |
| AN10346 | Putative cutinase with a predicted role in the hydrolysis of cutin |
| AN10360 | Ortholog of A. niger CBS 513.88 : An02g11450, A. oryzae RIB40 : AO090005001479, Neosartorya fischeri NRRL 181 : NFIA_069110 and Aspergillus wentii : Aspwe1_0173457 |
| AN10368 | Has domain(s) with predicted carbon-sulfur lyase activity and role in metabolic process |
| AN1037 | Oleate delta-12 desaturase |
| AN10380 | Putative Dicer protein |
| AN10386 | Ortholog of A. nidulans FGSC A4 : AN5039, A. niger CBS 513.88 : An09g05600, An13g01270 and A. oryzae RIB40 : AO090026000484, AO090005000917, AO090103000243, AO090005001067 |
| AN10387 | Possible pseudogene, similar to alternative sulfate transporter |
| AN10394 | Ortholog(s) have ubiquitin-protein transferase activity, role in anaphase-promoting complex-dependent proteasomal ubiquitin-dependent protein catabolic process, protein ubiquitination and anaphase-promoting complex localization |
| AN10400 | protein of unknown function |
| AN10404 | Ortholog of A. fumigatus Af293 : Afu7g01410, A. niger CBS 513.88 : An12g01490, A. oryzae RIB40 : AO090010000217, Neosartorya fischeri NRRL 181 : NFIA_114100 and Aspergillus clavatus NRRL 1 : ACLA_066160 |
| AN10408 | Ortholog of A. fumigatus Af293 : Afu3g05850, A. niger CBS 513.88 : An11g10750, A. oryzae RIB40 : AO090020000054, Neosartorya fischeri NRRL 181 : NFIA_071380 and Aspergillus clavatus NRRL 1 : ACLA_033820 |
| AN10410 | Has domain(s) with predicted role in transmembrane transport and integral component of membrane localization |
| AN10411 | Ortholog of A. fumigatus Af293 : Afu3g05670, A. niger CBS 513.88 : An11g10960, A. oryzae RIB40 : AO090020000037, Neosartorya fischeri NRRL 181 : NFIA_071520 and Aspergillus wentii : Aspwe1_0173697 |
| AN10415 | Ortholog(s) have double-stranded DNA-dependent ATPase activity, role in double-strand break repair via homologous recombination and Smc5-Smc6 complex, nucleus localization |
| AN1042 | Ortholog of Aspergillus tubingensis : Asptu1_0038623, Aspergillus brasiliensis : Aspbr1_0047614, Aspergillus glaucus : Aspgl1_0033936 and Aspergillus acidus : Aspfo1_0037723 |
| AN10423 | Ortholog(s) have role in hyphal growth, maltose metabolic process, regulation of transcription, DNA-templated and nucleus localization |
| AN10425 | Possible pseudogene |
| AN10428 | Has domain(s) with predicted role in transmembrane transport and integral component of membrane localization |
| AN1043 | Has domain(s) with predicted hydrolase activity, hydrolyzing O-glycosyl compounds activity and role in carbohydrate metabolic process |
| AN10431 | protein of unknown function |
| AN10440 | Ortholog(s) have membrane, mitochondrion localization |
| AN10444 | Gamma-glutamyl transpeptidase, involved in sexual development |
| AN10453 | Ortholog(s) have protein channel activity, role in protein import into mitochondrial inner membrane and mitochondrial inner membrane protein insertion complex localization |
| AN10454 | Ortholog of A. fumigatus Af293 : Afu6g12650, A. niger CBS 513.88 : An06g01860, A. oryzae RIB40 : AO090003000382, Neosartorya fischeri NRRL 181 : NFIA_058610 and Aspergillus wentii : Aspwe1_0033674 |
| AN10456 | NEDD8-specific protease |
| AN10457 | Ortholog of A. fumigatus Af293 : Afu7g04795, A. niger CBS 513.88 : An13g01320, A. oryzae RIB40 : AO090005000171, Neosartorya fischeri NRRL 181 : NFIA_025730 and Aspergillus clavatus NRRL 1 : ACLA_006450 |
| AN10458 | protein of unknown function |
| AN10461 | protein of unknown function |
| AN10464 | protein of unknown function |
| AN10468 | Ortholog(s) have role in cadmium ion transport, zinc II ion transport |
| AN10471 | Has domain(s) with predicted 3-hydroxyacyl-CoA dehydrogenase activity, coenzyme binding, oxidoreductase activity, acting on the CH-OH group of donors, NAD or NADP as acceptor activity |
| AN10486 | Putative nonribosomal peptide synthetase (NRPS)-like enzyme |
| AN10489 | FKBP-type peptidyl-prolyl cis-trans isomerase |
| AN10496 | Putative regulator of mannosylphosphorylation |
| AN10499 | Ortholog(s) have cytosol, nucleus localization |
| AN10502 | Has domain(s) with predicted catalytic activity, hydrolase activity, hydrolyzing O-glycosyl compounds activity and role in carbohydrate metabolic process |
| AN10508 | Ortholog(s) have Golgi apparatus, endoplasmic reticulum localization |
| AN10510 | Ortholog of A. fumigatus Af293 : Afu4g13140, A. niger CBS 513.88 : An01g08830, A. oryzae RIB40 : AO090009000267, Aspergillus wentii : Aspwe1_0100948 and Aspergillus sydowii : Aspsy1_0059125 |
| AN10514 | Has domain(s) with predicted oxidoreductase activity and role in metabolic process |
| AN10519 | Ortholog(s) have cytosol, nucleus localization |
| AN10529 | Has domain(s) with predicted catalytic activity, pyridoxal phosphate binding activity and role in biosynthetic process |
| AN10530 | Ortholog of A. fumigatus Af293 : Afu2g01690, A. niger CBS 513.88 : An14g03330, A. oryzae RIB40 : AO090701000773, Neosartorya fischeri NRRL 181 : NFIA_034010 and Aspergillus wentii : Aspwe1_0157118 |
| AN10531 | Ortholog of A. fumigatus Af293 : Afu6g07960, A. niger CBS 513.88 : An11g02900, Neosartorya fischeri NRRL 181 : NFIA_053660 and Aspergillus wentii : Aspwe1_0046026 |
| AN10533 | Putative trehalose-6-phosphate synthase |
| AN10535 | Has domain(s) with predicted GTP binding, GTPase activity, translation initiation factor activity, zinc ion binding activity, role in translational initiation and intracellular localization |
| AN10550 | Putative transcriptional regulator with homology to XlnR and AraR |
| AN10551 | Ortholog(s) have tRNA binding activity, role in regulation of transcription from RNA polymerase II promoter, tRNA wobble uridine modification and Elongator holoenzyme complex, cytosol, nucleus localization |
| AN10558 | Ortholog of A. fumigatus Af293 : Afu4g07280/sok1, A. niger CBS 513.88 : An04g01670, A. oryzae RIB40 : AO090023000845, Aspergillus wentii : Aspwe1_0023238 and Aspergillus terreus NIH2624 : ATET_05483 |
| AN10564 | Ortholog(s) have role in positive regulation of TOR signaling and Seh1-associated complex, cytosol, extrinsic component of fungal-type vacuolar membrane localization |
| AN10567 | Ortholog(s) have tRNA 2'-phosphotransferase activity, role in tRNA splicing, via endonucleolytic cleavage and ligation and cytoplasm, nucleus localization |
| AN10570 | Putative protein tyrosine phosphatase |
| AN10571 | Has domain(s) with predicted oxidoreductase activity |
| AN10574 | Ortholog(s) have nucleus localization |
| AN10579 | Ortholog of A. nidulans FGSC A4 : AN4100, A. fumigatus Af293 : Afu8g06630, A. niger CBS 513.88 : An05g01150, Aspergillus wentii : Aspwe1_0169274 and Aspergillus sydowii : Aspsy1_0065818, Aspsy1_0089096 |
| AN10589 | Ortholog(s) have fungal-type vacuole localization |
| AN1059 | Carnitine acetyltransferase, required for utilization of acetate as carbon source |
| AN10592 | Ortholog of A. fumigatus Af293 : Afu5g08730, A. oryzae RIB40 : AO090020000434, Neosartorya fischeri NRRL 181 : NFIA_078560 and Aspergillus versicolor : Aspve1_0134596 |
| AN10593 | Has domain(s) with predicted phosphatase activity, phosphoserine phosphatase activity and role in L-serine biosynthetic process, metabolic process |
| AN10598 | Has domain(s) with predicted calcium ion binding, calcium-dependent phospholipid binding activity |
| AN1060 | Histone H3 demethylase, involved in transcriptional regulation |
| AN10601 | Ortholog of A. nidulans FGSC A4 : AN2923, A. fumigatus Af293 : Afu2g17770, Afu8g00650, A. niger CBS 513.88 : An11g02090, An01g01000 and A. oryzae RIB40 : AO090020000174, AO090103000301 |
| AN10602 | Has domain(s) with predicted oxidoreductase activity, oxidoreductase activity, acting on the aldehyde or oxo group of donors, NAD or NADP as acceptor activity and role in oxidation-reduction process |
| AN10604 | Ortholog of A. fumigatus Af293 : Afu2g17760, A. niger CBS 513.88 : An03g02870, Neosartorya fischeri NRRL 181 : NFIA_093160, Aspergillus wentii : Aspwe1_0141692 and Aspergillus versicolor : Aspve1_0070749 |
| AN10606 | Has domain(s) with predicted RNA polymerase II transcription factor activity, sequence-specific DNA binding, zinc ion binding activity, role in regulation of transcription, DNA-templated and nucleus localization |
| AN10608 | Has domain(s) with predicted hydrolase activity |
| AN10609 | Ortholog(s) have role in mitochondrial respiratory chain complex III assembly and integral component of mitochondrial inner membrane localization |
| AN10621 | Ortholog(s) have ATP binding, ATPase activity, Y-form DNA binding, double-strand/single-strand DNA junction binding, four-way junction DNA binding and guanine/thymine mispair binding, more |
| AN10635 | Essential protein involved in septation and chromosome segregation |
| AN10636 | Ortholog(s) have Golgi apparatus, endoplasmic reticulum localization |
| AN10637 | Ortholog(s) have role in microautophagy, polyphosphate metabolic process, protein localization, vacuolar transport, vacuole fusion, non-autophagic |
| AN10638 | Ortholog(s) have role in hyphal growth |
| AN1064 | Putative nuclear pore complex protein with homology to Saccharomyces cerevisiae Nup57p |
| AN10643 | Has domain(s) with predicted protein heterodimerization activity, sequence-specific DNA binding activity and intracellular localization |
| AN10645 | Ortholog of A. fumigatus Af293 : Afu1g07310, A. niger CBS 513.88 : An07g09850, A. oryzae RIB40 : AO090012000984, Aspergillus wentii : Aspwe1_0040933 and Aspergillus sydowii : Aspsy1_0155965 |
| AN10650 | Ortholog of A. nidulans FGSC A4 : AN11203, A. fumigatus Af293 : Afu4g00520, A. niger CBS 513.88 : An12g05740 and Aspergillus wentii : Aspwe1_0072865, Aspwe1_0156452 |
| AN10657 | Has domain(s) with predicted catalytic activity and role in metabolic process |
| AN10661 | Has domain(s) with predicted hydrolase activity and role in nucleotide catabolic process |
| AN10666 | protein of unknown function |
| AN10675 | Ortholog(s) have mitochondrion localization |
| AN10681 | Ortholog(s) have structural constituent of ribosome activity, role in cytoplasmic translation, regulation of translational fidelity and cytosolic large ribosomal subunit localization |
| AN10686 | Has domain(s) with predicted role in transmembrane transport |
| AN1069 | Ortholog(s) have unfolded protein binding activity |
| AN10690 | Has domain(s) with predicted oxidoreductase activity and role in metabolic process |
| AN10710 | Ortholog(s) have phosphomannomutase activity, role in establishment or maintenance of cell polarity, protein targeting to ER and cytosol, nucleus localization |
| AN10711 | Ortholog(s) have role in mitochondrial respiratory chain complex IV assembly and mitochondrion localization |
| AN10713 | Has domain(s) with predicted mitochondrion localization |
| AN10718 | 6,7-mimethyl-8-ribityl-lumazine synthase |
| AN10721 | Ortholog(s) have protein methyltransferase activity, rRNA (guanine) methyltransferase activity, tRNA (guanine-N2-)-methyltransferase activity |
| AN10726 | protein of unknown function |
| AN10728 | Ortholog of Aspergillus versicolor : Aspve1_0084438, Aspergillus zonatus : Aspzo1_1674623, Aspergillus sydowii : Aspsy1_0056919 and Aspergillus carbonarius ITEM 5010 : Acar5010_128637, Acar5010_128669 |
| AN10730 | protein of unknown function |
| AN10734 | Ortholog(s) have role in regulation of translational elongation and cytosolic ribosome, mitochondrion localization |
| AN10736 | protein of unknown function |
| AN10740 | Ortholog(s) have cell surface, cytosolic large ribosomal subunit, preribosome, large subunit precursor localization |
| AN10743 | Ortholog(s) have role in endocytosis, establishment or maintenance of actin cytoskeleton polarity, vacuole organization and integral component of Golgi membrane localization |
| AN10746 | MOSC-domain containing protein |
| AN10760 | Has domain(s) with predicted zinc ion binding activity |
| AN10761 | Predicted PIN domain-containing RNA-binding protein |
| AN10763 | Subunit of the SAGA transcriptional regulatory complex with a role in nucleosome positioning |
| AN10764 | Ortholog(s) have role in N',N'',N'''-triacetylfusarinine C biosynthetic process, cellular response to hydrogen peroxide, cellular response to iron ion starvation, ergosterol biosynthetic process, pathogenesis and peroxisome localization |
| AN10768 | Has domain(s) with predicted oxidoreductase activity and role in oxidation-reduction process |
| AN10776 | Putative cytochrome P450 |
| AN10781 | Ortholog(s) have cytosol, nucleus localization |
| AN10784 | Has domain(s) with predicted role in RNA processing |
| AN10788 | Ortholog(s) have deubiquitinase activator activity |
| AN10793 | Has domain(s) with predicted oxidoreductase activity, zinc ion binding activity and role in oxidation-reduction process |
| AN10797 | Ortholog(s) have mitochondrion localization |
| AN10799 | Ortholog of A. fumigatus Af293 : Afu2g13480, A. niger CBS 513.88 : An02g02390, A. oryzae RIB40 : AO090026000326, Aspergillus wentii : Aspwe1_0045443 and Aspergillus sydowii : Aspsy1_0151451 |
| AN10806 | Ortholog(s) have cytoplasm localization |
| AN10811 | Putative cytochrome P450 |
| AN10814 | Has domain(s) with predicted FAD binding, oxidoreductase activity and role in metabolic process |
| AN10816 | Ortholog of A. fumigatus Af293 : Afu8g05030, A. oryzae RIB40 : AO090003001446, AO090009000107, Aspergillus versicolor : Aspve1_0130291 and Aspergillus clavatus NRRL 1 : ACLA_058430 |
| AN10823 | Ortholog(s) have FAD transmembrane transporter activity, role in FAD transport and mitochondrion localization |
| AN1083 | Ortholog(s) have mitochondrion localization |
| AN10838 | Has domain(s) with predicted catalytic activity, chitin binding, chitinase activity, hydrolase activity, hydrolyzing O-glycosyl compounds activity |
| AN10841 | Ortholog(s) have dodecenoyl-CoA delta-isomerase activity, role in fatty acid beta-oxidation, filamentous growth and peroxisome localization |
| AN10845 | Ortholog(s) have plasma membrane localization |
| AN10854 | Ortholog(s) have AMP binding, AMP-activated protein kinase activity, ATP binding, protein serine/threonine kinase activator activity |
| AN10856 | Possible pseudogene |
| AN10857 | Ortholog of A. fumigatus Af293 : Afu5g13110, A. niger CBS 513.88 : An14g06040, A. oryzae RIB40 : AO090120000436, Aspergillus wentii : Aspwe1_0039047 and Aspergillus sydowii : Aspsy1_0040418 |
| AN10861 | Has domain(s) with predicted role in transmembrane transport and integral component of membrane localization |
| AN10862 | Has domain(s) with predicted role in transmembrane transport and integral component of membrane localization |
| AN10867 | Putative alpha-L-rhamnosidase |
| AN10870 | protein of unknown function |
| AN10873 | Ortholog(s) have role in protein import into mitochondrial outer membrane and mitochondrial outer membrane localization |
| AN10878 | Ortholog(s) have role in ER to Golgi vesicle-mediated transport, cellular response to drug, vesicle fusion with Golgi apparatus |
| AN10879 | Ortholog(s) have holocytochrome-c synthase activity and role in aerobic respiration, cytochrome c-heme linkage, filamentous growth of a population of unicellular organisms, sporocarp development involved in sexual reproduction |
| AN10882 | Ortholog of A. fumigatus Af293 : Afu4g04560, A. niger CBS 513.88 : An14g00260, A. oryzae RIB40 : AO090206000132, Neosartorya fischeri NRRL 181 : NFIA_028790 and Aspergillus wentii : Aspwe1_0115725 |
| AN10905 | Ortholog(s) have gamma-aminobutyric acid:proton symporter activity, putrescine transmembrane transporter activity and role in gamma-aminobutyric acid transport, putrescine transport, transmembrane transport |
| AN10908 | Ortholog of A. niger CBS 513.88 : An06g00460, An16g08070, An07g04470, A. oryzae RIB40 : AO090038000122 and Aspergillus wentii : Aspwe1_0030051, Aspwe1_0034330, Aspwe1_0052115, Aspwe1_0144037, Aspwe1_0734215 |
| AN10911 | Has domain(s) with predicted RNA polymerase II transcription factor activity, sequence-specific DNA binding, zinc ion binding activity, role in regulation of transcription, DNA-templated and nucleus localization |
| AN10913 | Ortholog(s) have mitochondrion localization |
| AN10915 | Ortholog of A. niger CBS 513.88 : An04g09630, An03g01920, Aspergillus wentii : Aspwe1_0160312, Aspwe1_0167997, Aspergillus versicolor : Aspve1_0513563 and Aspergillus niger ATCC 1015 : 195107-mRNA, 45712-mRNA |
| AN10917 | Has domain(s) with predicted ATP binding, nucleoside-triphosphatase activity, nucleotide binding activity |
| AN10924 | Ortholog(s) have tRNA adenylyltransferase activity, role in tRNA 3'-terminal CCA addition and mitochondrial matrix localization |
| AN10932 | Has domain(s) with predicted hydrolase activity, hydrolase activity, acting on carbon-nitrogen (but not peptide) bonds, in linear amidines activity and role in metabolic process |
| AN10936 | Ortholog(s) have 2,5-diamino-6-ribitylamino-4(3H)-pyrimidinone 5'-phosphate deaminase activity, pseudouridine synthase activity, role in riboflavin biosynthetic process, tRNA pseudouridine synthesis and cytosol localization |
| AN1094 | Putative mitochondrial NADH dehydrogenase (ubiquinone) with a predicted role in energy metabolism |
| AN10941 | Has domain(s) with predicted nucleic acid binding, nucleotide binding activity |
| AN1095 | Ortholog(s) have structural constituent of ribosome activity and mitochondrial large ribosomal subunit, nucleolus localization |
| AN10952 | Has domain(s) with predicted FAD binding, oxidoreductase activity and role in metabolic process |
| AN10966 | Ortholog(s) have role in mRNA splicing, via spliceosome and U1 snRNP, U2 snRNP, U4/U6 x U5 tri-snRNP complex, U5 snRNP, cytosol, spliceosomal complex localization |
| AN10969 | Ortholog of A. nidulans FGSC A4 : AN10998, A. fumigatus Af293 : Afu2g01150, Afu3g01930, A. niger CBS 513.88 : An09g03620, An05g01520 and A. oryzae RIB40 : AO090012000323, AO090701000385 |
| AN1097 | Ortholog(s) have protein kinase activity, role in protein phosphorylation, proteolysis and cytoplasm localization |
| AN10973 | Putative citrate synthase |
| AN1098 | protein of unknown function |
| AN10981 | Putative bifunctional GTP cyclohydrolase II |
| AN10984 | protein of unknown function |
| AN1100 | Has domain(s) with predicted catalytic activity, hydrolase activity |
| AN11000 | Ortholog of Aspergillus flavus NRRL 3357 : AFL2T_11759, Neosartorya fischeri NRRL 181 : NFIA_094100, Aspergillus versicolor : Aspve1_0085617 and Aspergillus clavatus NRRL 1 : ACLA_044880 |
| AN11009 | Putative mitochondrial pyruvate carboxylase |
| AN1101 | Ortholog of A. nidulans FGSC A4 : AN8026, AN0010, A. fumigatus Af293 : Afu3g00120, Afu5g09120 and A. niger CBS 513.88 : An12g05610, An07g03970 |
| AN11016 | Has domain(s) with predicted role in transmembrane transport and integral component of membrane localization |
| AN11019 | Ortholog(s) have protein-cysteine S-palmitoyltransferase activity, role in protein palmitoylation and endoplasmic reticulum localization |
| AN11021 | Ortholog of A. oryzae RIB40 : AO090001000219, Aspergillus brasiliensis : Aspbr1_0192818, N. fischeri NRRL 181 : NFIA_012970, Aspergillus flavus NRRL 3357 : AFL2T_07452 and A. clavatus NRRL 1 : ACLA_022880 |
| AN11022 | Ortholog of A. fumigatus Af293 : Afu5g07770, A. oryzae RIB40 : AO090701000684, Neosartorya fischeri NRRL 181 : NFIA_079500 and Aspergillus clavatus NRRL 1 : ACLA_012740 |
| AN11033 | Ortholog of A. fumigatus Af293 : Afu5g02050, A. niger CBS 513.88 : An16g01890, A. oryzae RIB40 : AO090003001318, Aspergillus wentii : Aspwe1_0116157 and Aspergillus sydowii : Aspsy1_0207101 |
| AN11034 | Has domain(s) with predicted catalytic activity and role in metabolic process |
| AN11038 | Has domain(s) with predicted carbohydrate binding, carbon-oxygen lyase activity and role in amino sugar catabolic process, carbohydrate metabolic process |
| AN11041 | Has domain(s) with predicted zinc ion binding activity |
| AN11046 | protein of unknown function |
| AN11054 | Putative alpha-1,4-glucosidase |
| AN11056 | Has domain(s) with predicted protein heterodimerization activity, role in transcription initiation from RNA polymerase II promoter and nucleus localization |
| AN11057 | protein of unknown function |
| AN11064 | Putative alpha-1,3-glucanase |
| AN11069 | Ortholog of A. fumigatus Af293 : Afu5g03860, A. niger CBS 513.88 : An09g06330, Neosartorya fischeri NRRL 181 : NFIA_038250 and Aspergillus wentii : Aspwe1_0115239 |
| AN11075 | protein of unknown function |
| AN11076 | Ortholog of A. fumigatus Af293 : Afu3g02220, A. niger CBS 513.88 : An01g02020, Neosartorya fischeri NRRL 181 : NFIA_003350, Aspergillus wentii : Aspwe1_0171305 and Aspergillus versicolor : Aspve1_0056749 |
| AN11078 | Putative unsaturated glucuronyl hydrolase |
| AN11080 | Putative dimethyl-allyl-tryptophan synthase (DMATS)-type aromatic prenyltransferase |
| AN11081 | Has domain(s) with predicted 3-beta-hydroxy-delta5-steroid dehydrogenase activity and role in oxidation-reduction process, steroid biosynthetic process |
| AN11086 | protein of unknown function |
| AN11091 | Predicted DDE1 transposon-related ORF |
| AN11105 | Has domain(s) with predicted oxidoreductase activity and role in metabolic process |
| AN11109 | ORF that was absent from the original release of version 4 of the A. nidulans annotation, but present in a previous version |
| AN11124 | Has domain(s) with predicted hydrolase activity and role in dUTP metabolic process |
| AN11131 | Ortholog(s) have RNA polymerase II core binding activity, role in chromatin-mediated maintenance of transcription, transcription elongation from RNA polymerase II promoter and transcription elongation factor complex localization |
| AN11136 | Ortholog(s) have protein transporter activity, role in protein import into mitochondrial inner membrane and cytosol, mitochondrial intermembrane space protein transporter complex localization |
| AN11138 | Ortholog(s) have cytoplasm localization |
| AN11142 | Putative cytochrome P450 |
| AN11145 | Functional ortholog of S. cerevisiae CGR1 |
| AN11146 | Ortholog(s) have cytidine deaminase activity, role in cytidine catabolic process, deoxycytidine catabolic process, pyrimidine-containing compound salvage and cytosol, nucleus localization |
| AN11154 | Ortholog of A. niger CBS 513.88 : An05g01490, A. oryzae RIB40 : AO090009000214, Neosartorya fischeri NRRL 181 : NFIA_084670, Aspergillus wentii : Aspwe1_0119954 and Aspergillus versicolor : Aspve1_0144019 |
| AN11156 | Ortholog of Aspergillus glaucus : Aspgl1_0125887 |
| AN11161 | Has domain(s) with predicted phosphatidylserine decarboxylase activity and role in phospholipid biosynthetic process |
| AN11162 | Ortholog(s) have nucleolus localization |
| AN11169 | Has domain(s) with predicted DNA binding, RNA polymerase II transcription factor activity, sequence-specific DNA binding, zinc ion binding activity and role in regulation of transcription, DNA-templated, transcription, DNA-templated |
| AN1117 | Ortholog(s) have COPII adaptor activity, role in ER to Golgi vesicle-mediated transport and ER to Golgi transport vesicle, Golgi apparatus, endoplasmic reticulum, plasma membrane localization |
| AN11171 | Ortholog of A. nidulans FGSC A4 : AN7148, A. fumigatus Af293 : Afu4g03595, A. niger CBS 513.88 : An02g01320, An14g01685, An03g06340 and A. oryzae RIB40 : AO090026000213, AO090026000142, AO090023000130 |
| AN11174 | Ortholog of A. fumigatus Af293 : Afu7g02120, A. niger CBS 513.88 : An12g00530, A. oryzae RIB40 : AO090038000590, Neosartorya fischeri NRRL 181 : NFIA_114850 and Aspergillus wentii : Aspwe1_0042354 |
| AN11177 | Has domain(s) with predicted oxidoreductase activity, zinc ion binding activity and role in oxidation-reduction process |
| AN11180 | Ortholog of A. fumigatus Af293 : Afu3g01760, A. niger CBS 513.88 : An05g01480, A. oryzae RIB40 : AO090023000156, Aspergillus wentii : Aspwe1_0120430 and Aspergillus sydowii : Aspsy1_0061462 |
| AN11182 | Ortholog of Aspergillus sydowii : Aspsy1_0061393 |
| AN11183 | Ortholog(s) have RNA polymerase I activity, RNA polymerase II activity, RNA polymerase III activity, RNA-directed RNA polymerase activity, zinc ion binding activity |
| AN11189 | Has domain(s) with predicted role in transmembrane transport and integral component of membrane localization |
| AN11190 | Ortholog(s) have role in cellular response to drug |
| AN11195 | Has domain(s) with predicted DNA binding, RNA polymerase II transcription factor activity, sequence-specific DNA binding, nucleic acid binding, zinc ion binding activity |
| AN11196 | Has domain(s) with predicted role in transmembrane transport and integral component of membrane localization |
| AN11197 | Has domain(s) with predicted DNA binding, nucleic acid binding, zinc ion binding activity, role in transcription, DNA-templated and nucleus localization |
| AN11202 | Putative dimethyl-allyl-tryptophan synthase (DMATS) type aromatic prenyltransferase |
| AN11203 | Ortholog of A. nidulans FGSC A4 : AN10650, A. fumigatus Af293 : Afu4g00520, A. niger CBS 513.88 : An12g05740 and Aspergillus wentii : Aspwe1_0072865, Aspwe1_0156452 |
| AN11221 | Has domain(s) with predicted proline dehydrogenase activity and role in glutamate biosynthetic process, oxidation-reduction process, proline catabolic process |
| AN11234 | Ortholog(s) have endoplasmic reticulum localization |
| AN11251 | Has domain(s) with predicted 4 iron, 4 sulfur cluster binding, NADH dehydrogenase (ubiquinone) activity, oxidoreductase activity, acting on NAD(P)H, quinone binding activity and role in oxidation-reduction process |
| AN11260 | protein of unknown function |
| AN11288 | protein of unknown function |
| AN11292 | protein of unknown function |
| AN11303 | Ortholog(s) have proton-transporting ATP synthase activity, rotational mechanism, structural molecule activity and role in ATP synthesis coupled proton transport, cristae formation, protein complex oligomerization |
| AN11313 | protein of unknown function |
| AN11314 | Ortholog of Aspergillus glaucus : Aspgl1_0053511, Aspergillus versicolor : Aspve1_0037663 and Aspergillus sydowii : Aspsy1_0038825 |
| AN1132 | Negative regulator of qut gene expression |
| AN11321 | protein of unknown function |
| AN1133 | Ortholog(s) have Golgi apparatus, fungal-type vacuole membrane localization |
| AN11375 | protein of unknown function |
| AN11378 | Ortholog of A. oryzae RIB40 : AO090001000134, Aspergillus glaucus : Aspgl1_0071332, Aspergillus wentii : Aspwe1_0171393 and Aspergillus versicolor : Aspve1_0087914 |
| AN1140 | Dehydroshikimate dehydratase, involved in quinic acid utilization |
| AN11419 | Ortholog(s) have cytosolic large ribosomal subunit, hyphal cell wall, yeast-form cell wall localization |
| AN11425 | Ortholog of Neosartorya fischeri NRRL 181 : NFIA_021020, Aspergillus versicolor : Aspve1_0043603, Aspergillus clavatus NRRL 1 : ACLA_030960 and Aspergillus zonatus : Aspzo1_0131242 |
| AN11432 | protein of unknown function |
| AN11438 | protein of unknown function |
| AN11461 | Possible pseudogene |
| AN1147 | Ortholog of A. fumigatus Af293 : Afu1g11500/erg27, A. niger CBS 513.88 : An08g03620, Aspergillus wentii : Aspwe1_0172221, Aspergillus sydowii : Aspsy1_0053214 and Aspergillus terreus NIH2624 : ATET_00340 |
| AN11477 | Ortholog of Aspergillus sydowii : Aspsy1_0137382 and Aspergillus aculeatus ATCC16872 : Aacu16872_040719 |
| AN1152 | Protein expressed at decreased levels in a hapX mutant versus wild-type |
| AN1154 | Ortholog(s) have role in ER to Golgi vesicle-mediated transport, protein retention in ER lumen, vesicle organization and ER to Golgi transport vesicle, endoplasmic reticulum, plasma membrane localization |
| AN11543 | Ortholog of A. niger CBS 513.88 : An14g00820, Aspergillus wentii : Aspwe1_0116393, Aspergillus versicolor : Aspve1_0046161 and Aspergillus clavatus NRRL 1 : ACLA_054360 |
| AN11544 | protein of unknown function |
| AN11552 | protein of unknown function |
| AN1156 | Ortholog of A. fumigatus Af293 : Afu1g11450, A. niger CBS 513.88 : An08g03570, A. oryzae RIB40 : AO090038000280, Aspergillus wentii : Aspwe1_0068767 and Aspergillus sydowii : Aspsy1_0191007 |
| AN11563 | Ortholog of Aspergillus fumigatus A1163 : AFUB_023040, Aspergillus clavatus NRRL 1 : ACLA_068330 and Aspergillus aculeatus ATCC16872 : Aacu16872_043662 |
| AN11574 | protein of unknown function |
| AN11581 | protein of unknown function |
| AN11582 | protein of unknown function |
| AN11585 | protein of unknown function |
| AN11597 | Ortholog of A. fumigatus Af293 : Afu5g03300, A. niger CBS 513.88 : An09g05625, Neosartorya fischeri NRRL 181 : NFIA_038810, Aspergillus wentii : Aspwe1_0114897 and Aspergillus versicolor : Aspve1_0203178 |
| AN11600 | protein of unknown function |
| AN11616 | protein of unknown function |
| AN1166 | Ortholog(s) have RNA binding, structural constituent of ribosome activity |
| AN11670 | protein of unknown function |
| AN1168 | Putative voltage-gated calcium channel |
| AN1168-uORF | Conserved upstream open reading frame (uORF) of AN1168/cch1 |
| AN1169 | Ortholog of A. fumigatus Af293 : Afu1g11100, A. niger CBS 513.88 : An08g03390, A. oryzae RIB40 : AO090038000299, Neosartorya fischeri NRRL 181 : NFIA_014530 and Aspergillus wentii : Aspwe1_0172243 |
| AN11691 | Ortholog(s) have structural constituent of ribosome activity and mitochondrial large ribosomal subunit localization |
| AN11696 | Ortholog(s) have RecQ helicase-Topo III complex localization |
| AN11699 | Ortholog(s) have nucleus, ribosome localization |
| AN11701 | protein of unknown function |
| AN11703 | protein of unknown function |
| AN11704 | Has domain(s) with predicted RNA polymerase II transcription cofactor activity, role in regulation of transcription from RNA polymerase II promoter and mediator complex localization |
| AN11707 | Ortholog of A. fumigatus Af293 : Afu3g12460, A. niger CBS 513.88 : An02g08400, A. oryzae RIB40 : AO090005000723, Aspergillus wentii : Aspwe1_0371733 and Aspergillus glaucus : Aspgl1_0043974 |
| AN11711 | protein of unknown function |
| AN11717 | protein of unknown function |
| AN11718 | Ortholog(s) have mitochondrial intermembrane space localization |
| AN11721 | Ortholog(s) have 5' overhang single-stranded DNA endodeoxyribonuclease activity, 5'-flap endonuclease activity, ATP-dependent DNA helicase activity and chromatin binding, more |
| AN11725 | protein of unknown function |
| AN1173 | Ortholog of A. fumigatus Af293 : Afu1g11060, A. niger CBS 513.88 : An08g03320, Aspergillus wentii : Aspwe1_0024838, Aspergillus sydowii : Aspsy1_0038077 and Aspergillus terreus NIH2624 : ATET_00305 |
| AN11730 | Ortholog of A. fumigatus Af293 : Afu6g12430, A. niger CBS 513.88 : An06g01590, A. oryzae RIB40 : AO090009000169, Aspergillus wentii : Aspwe1_0166417 and Aspergillus carbonarius ITEM 5010 : Acar5010_502743 |
| AN11733 | protein of unknown function |
| AN11739 | protein of unknown function |
| AN1174 | Has domain(s) with predicted ATP binding, ATPase activity, ATPase activity, coupled to transmembrane movement of substances, nucleoside-triphosphatase activity, nucleotide binding activity and role in transport |
| AN11745 | protein of unknown function |
| AN11752 | protein of unknown function |
| AN11758 | protein of unknown function |
| AN11764 | protein of unknown function |
| AN11768 | protein of unknown function |
| AN1177 | Ortholog(s) have cytosol, plasma membrane localization |
| AN11771 | protein of unknown function |
| AN11775 | Ortholog(s) have role in Golgi to endosome transport, Golgi to plasma membrane protein transport, protein targeting to Golgi, vesicle organization and integral component of Golgi membrane, trans-Golgi network localization |
| AN11779 | protein of unknown function |
| AN1178 | Ortholog(s) have cytosol, nucleus localization |
| AN11796 | protein of unknown function |
| AN11804 | Has domain(s) with predicted ATP binding, DNA binding, helicase activity, nucleic acid binding activity |
| AN11808 | Ortholog of A. niger CBS 513.88 : An11g03390, An16g00760, Aspergillus wentii : Aspwe1_0119499, Aspergillus versicolor : Aspve1_0125345, Aspve1_0138797 and Aspergillus sydowii : Aspsy1_0139733, Aspsy1_0154359 |
| AN1182 | Beta-tubulin, highly conserved component of microtubules |
| AN11823 | Ortholog(s) have role in secondary metabolite biosynthetic process |
| AN11842 | protein of unknown function |
| AN11844 | protein of unknown function |
| AN11845 | protein of unknown function |
| AN11846 | protein of unknown function |
| AN11852 | protein of unknown function |
| AN11858 | Ortholog of A. fumigatus Af293 : Afu2g13970, A. oryzae RIB40 : AO090026000164, Neosartorya fischeri NRRL 181 : NFIA_089160 and Aspergillus wentii : Aspwe1_0119587 |
| AN11862 | Ortholog(s) have role in posttranslational protein targeting to membrane and TRC complex, nucleus localization |
| AN11869 | protein of unknown function |
| AN11872 | protein of unknown function |
| AN11873 | Has domain(s) with predicted NAD+ binding activity |
| AN11885 | Ortholog(s) have cytosol, nucleus localization |
| AN11888 | Ortholog of A. fumigatus Af293 : Afu4g08270, Aspergillus wentii : Aspwe1_0102345, Aspergillus terreus NIH2624 : ATET_05370 and Aspergillus carbonarius ITEM 5010 : Acar5010_504985 |
| AN11898 | Ortholog of A. fumigatus Af293 : Afu7g05420, Aspergillus wentii : Aspwe1_0101341, Aspergillus sydowii : Aspsy1_0159682 and Aspergillus terreus NIH2624 : ATET_06393 |
| AN1190 | Putative nuclear pore complex protein with homology to Saccharomyces cerevisiae Nup84p |
| AN11901 | Putative component of the EKC/KEOPS complex |
| AN11907 | protein of unknown function |
| AN1191 | Small ubiquitin-like modifier (SUMO) protein |
| AN11911 | protein of unknown function |
| AN11912 | Ortholog of A. fumigatus Af293 : Afu2g02600, A. oryzae RIB40 : AO090026000604, Neosartorya fischeri NRRL 181 : NFIA_035000 and Aspergillus clavatus NRRL 1 : ACLA_092190 |
| AN11917 | Ortholog(s) have fungal-type vacuole localization |
| AN11920 | Has domain(s) with predicted oxidoreductase activity and role in metabolic process |
| AN11921 | Ortholog(s) have role in response to amino acid |
| AN11924 | Has domain(s) with predicted electron carrier activity, heme binding, iron ion binding, oxidoreductase activity, acting on paired donors, with incorporation or reduction of molecular oxygen activity and role in oxidation-reduction process |
| AN11925 | protein of unknown function |
| AN11930 | Ortholog(s) have ATP binding, DNA-dependent ATPase activity, double-stranded DNA binding, recombinase activity, single-stranded DNA binding activity |
| AN11934 | Has domain(s) with predicted ATP binding, ATPase activity, ATPase activity, coupled to transmembrane movement of substances, nucleoside-triphosphatase activity, nucleotide binding activity and role in transmembrane transport |
| AN11935 | Ortholog of A. fumigatus Af293 : Afu1g10060, A. oryzae RIB40 : AO090038000429, Aspergillus wentii : Aspwe1_0168733, Aspergillus sydowii : Aspsy1_0126887 and Aspergillus terreus NIH2624 : ATET_00179 |
| AN11937 | protein of unknown function |
| AN11938 | protein of unknown function |
| AN11946 | protein of unknown function |
| AN11968 | Ortholog(s) have 5-amino-6-(5-phosphoribosylamino)uracil reductase activity, role in riboflavin biosynthetic process and cytosol, nucleus localization |
| AN11974 | protein of unknown function |
| AN11979 | Ortholog(s) have IgE binding activity and fungal-type cell wall localization |
| AN1198 | Putative aminomethyltransferase with a predicted role in glycine, serine, and threonine metabolism |
| AN11980 | protein of unknown function |
| AN11983 | protein of unknown function |
| AN11991 | protein of unknown function |
| AN12003 | protein of unknown function |
| AN12013 | protein of unknown function |
| AN12021 | protein of unknown function |
| AN12033 | Has domain(s) with predicted catalytic activity |
| AN12035 | Has domain(s) with predicted carbonate dehydratase activity, zinc ion binding activity |
| AN12044 | Ortholog(s) have endoplasmic reticulum localization |
| AN12048 | protein of unknown function |
| AN1205 | Ortholog(s) have tubulin binding activity and role in positive regulation of transcription elongation from RNA polymerase II promoter, regulation of conjugation with cellular fusion, tubulin complex assembly |
| AN12051 | protein of unknown function |
| AN12052 | protein of unknown function |
| AN12056 | Ortholog of A. fumigatus Af293 : Afu8g04230, A. oryzae RIB40 : AO090103000038, Aspergillus wentii : Aspwe1_0175151, Aspergillus sydowii : Aspsy1_0086843 and Aspergillus terreus NIH2624 : ATET_00036 |
| AN12062 | Ortholog(s) have cytosol, nucleus localization |
| AN12065 | Putative polketide synthase |
| AN12066 | Has domain(s) with predicted catalytic activity and role in metabolic process |
| AN12072 | Has domain(s) with predicted role in cell wall macromolecule catabolic process |
| AN12078 | protein of unknown function |
| AN12085 | Ortholog of Aspergillus versicolor : Aspve1_0150796 and Aspergillus sydowii : Aspsy1_0090491 |
| AN12086 | Has domain(s) with predicted carbon-sulfur lyase activity and role in metabolic process |
| AN12095 | protein of unknown function |
| AN12099 | Ortholog of A. nidulans FGSC A4 : AN11017, A. fumigatus Af293 : Afu4g14480/tpcL, A. oryzae RIB40 : AO090026000010 and Neosartorya fischeri NRRL 181 : NFIA_101640, NFIA_101900 |
| AN12100 | Has domain(s) with predicted oxidoreductase activity and role in metabolic process |
| AN12102 | Has domain(s) with predicted identical protein binding, serine-type endopeptidase activity and role in negative regulation of catalytic activity |
| AN12109 | protein of unknown function |
| AN12114 | protein of unknown function |
| AN12127 | Ortholog of A. fumigatus Af293 : Afu3g07310, A. niger CBS 513.88 : An02g13400, A. oryzae RIB40 : AO090020000191 and Aspergillus wentii : Aspwe1_0173528 |
| AN12129 | Has domain(s) with predicted role in transmembrane transport and integral component of membrane localization |
| AN1213 | Ortholog of A. fumigatus Af293 : Afu1g10650, A. niger CBS 513.88 : An08g02730, A. oryzae RIB40 : AO090038000354, Neosartorya fischeri NRRL 181 : NFIA_014990 and Aspergillus versicolor : Aspve1_0119055 |
| AN12131 | Has domain(s) with predicted aldehyde-lyase activity, catalytic activity, zinc ion binding activity and role in carbohydrate metabolic process |
| AN12132 | protein of unknown function |
| AN12133 | Ortholog of A. niger CBS 513.88 : An16g07310, Neosartorya fischeri NRRL 181 : NFIA_098160, Aspergillus wentii : Aspwe1_0685698 and Aspergillus versicolor : Aspve1_0037327 |
| AN12149 | Ortholog of Aspergillus glaucus : Aspgl1_0039633, Aspergillus wentii : Aspwe1_0187295, Aspergillus versicolor : Aspve1_0046062 and Aspergillus sydowii : Aspsy1_0048834 |
| AN12155 | protein of unknown function |
| AN12159 | Ortholog of A. fumigatus Af293 : Afu2g10870, A. niger CBS 513.88 : An02g02720, A. oryzae RIB40 : AO090011000543, Neosartorya fischeri NRRL 181 : NFIA_086190 and Aspergillus wentii : Aspwe1_0044183 |
| AN12163 | Ortholog of A. fumigatus Af293 : Afu2g02240, A. niger CBS 513.88 : An07g06590, A. oryzae RIB40 : AO090011000503, Aspergillus wentii : Aspwe1_0353485 and Aspergillus sydowii : Aspsy1_0155092 |
| AN1217 | Putative LIM/homeobox transcription factor |
| AN12173 | Ortholog(s) have cytosol, nucleus localization |
| AN12183 | protein of unknown function |
| AN12185 | Has domain(s) with predicted protein C-terminal S-isoprenylcysteine carboxyl O-methyltransferase activity, role in C-terminal protein methylation and integral component of membrane localization |
| AN12195 | protein of unknown function |
| AN12204 | Ortholog of A. fumigatus Af293 : Afu5g04020, A. niger CBS 513.88 : An09g06150, A. oryzae RIB40 : AO090102000605, Neosartorya fischeri NRRL 181 : NFIA_038090 and Aspergillus wentii : Aspwe1_0043117 |
| AN12213 | Ortholog(s) have ubiquinol-cytochrome-c reductase activity, role in aerobic respiration, mitochondrial electron transport, ubiquinol to cytochrome c and mitochondrial respiratory chain complex III, plasma membrane localization |
| AN12222 | Has domain(s) with predicted role in transmembrane transport and integral component of membrane localization |
| AN12229 | Ortholog(s) have cytosol, nucleus localization |
| AN12234 | Ortholog of A. oryzae RIB40 : AO090023000229, Aspergillus wentii : Aspwe1_0171134, Aspergillus versicolor : Aspve1_0480771, Aspergillus zonatus : Aspzo1_0653150 and Aspergillus sydowii : Aspsy1_0084619 |
| AN12239 | protein of unknown function |
| AN12241 | Ortholog of A. niger CBS 513.88 : An12g08040, Aspergillus wentii : Aspwe1_0314624, Aspergillus versicolor : Aspve1_0153222 and Aspergillus niger ATCC 1015 : 42139-mRNA |
| AN12245 | Ortholog of Aspergillus tubingensis : Asptu1_0179003, Aspergillus brasiliensis : Aspbr1_0067218 and Aspergillus kawachii : Aspka1_0176756 |
| AN12250 | protein of unknown function |
| AN12255 | Ortholog(s) have hydrolase activity, acting on ester bonds activity, role in iron assimilation and cytoplasm localization |
| AN12261 | protein of unknown function |
| AN12264 | protein of unknown function |
| AN12270 | protein of unknown function |
| AN12280 | Has domain(s) with predicted DNA binding, chromatin binding activity |
| AN12281 | protein of unknown function |
| AN12286 | protein of unknown function |
| AN12290 | Has domain(s) with predicted oxidoreductase activity and role in metabolic process |
| AN12306 | Has domain(s) with predicted oxidoreductase activity and role in metabolic process |
| AN12314 | Ortholog(s) have role in hyphal growth |
| AN12318 | protein of unknown function |
| AN1232 | Binuclear Zn cluster, chromatin-associated protein involved in the DNA damage response |
| AN12334 | protein of unknown function |
| AN12337 | Ortholog(s) have role in protein folding in endoplasmic reticulum and integral component of endoplasmic reticulum membrane localization |
| AN12339 | Ortholog of A. fumigatus Af293 : Afu8g01800, A. niger CBS 513.88 : An07g00940, An03g05910, A. oryzae RIB40 : AO090010000636 and Aspergillus wentii : Aspwe1_0121910 |
| AN12343 | Ortholog of Aspergillus wentii : Aspwe1_0053067, Aspergillus versicolor : Aspve1_0057213, Aspergillus clavatus NRRL 1 : ACLA_001690 and Aspergillus niger ATCC 1015 : 188904-mRNA |
| AN12348 | Ortholog of A. niger CBS 513.88 : An08g00240, Neosartorya fischeri NRRL 181 : NFIA_016850, Aspergillus versicolor : Aspve1_0024638 and Aspergillus clavatus NRRL 1 : ACLA_026690 |
| AN12351 | protein of unknown function |
| AN12358 | Ortholog(s) have mitochondrion localization |
| AN12360 | Has domain(s) with predicted role in response to stress |
| AN1237 | Protein with homology to Saccharomyces cerevisiae Rad51p and to bacterial RecA |
| AN12377 | protein of unknown function |
| AN12378 | protein of unknown function |
| AN12379 | protein of unknown function |
| AN12388 | Ortholog of A. fumigatus Af293 : Afu6g08650, Aspergillus flavus NRRL 3357 : AFL2T_09170, Neosartorya fischeri NRRL 181 : NFIA_054300 and Aspergillus versicolor : Aspve1_0083813 |
| AN1239 | Ortholog of A. fumigatus Af293 : Afu7g01750, A. niger CBS 513.88 : An12g00960, A. oryzae RIB40 : AO090120000009, Aspergillus wentii : Aspwe1_0045721 and Aspergillus sydowii : Aspsy1_0085157 |
| AN12411 | protein of unknown function |
| AN12413 | Ortholog of A. nidulans FGSC A4 : AN9185, A. oryzae RIB40 : AO090011000007, Aspergillus versicolor : Aspve1_0078310, Aspve1_0402361 and Aspergillus niger ATCC 1015 : 55148-mRNA |
| AN12419 | Ortholog(s) have role in mRNA export from nucleus and cytosol, nucleolus localization |
| AN1242 | Putative nonribosomal peptide synthase (NRPS) |
| AN12423 | protein of unknown function |
| AN12424 | protein of unknown function |
| AN1244 | Ortholog(s) have cytosol, nucleus localization |
| AN12446 | protein of unknown function |
| AN1245 | Ortholog of A. fumigatus Af293 : Afu1g10355, A. niger CBS 513.88 : An08g02270, Aspergillus wentii : Aspwe1_0036569, Aspergillus sydowii : Aspsy1_0053120 and Aspergillus terreus NIH2624 : ATET_00225 |
| AN12452 | Has domain(s) with predicted phosphotransferase activity, for other substituted phosphate groups activity, role in phospholipid biosynthetic process and membrane localization |
| AN12457 | Ortholog(s) have phospholipid transporter activity and role in drug export, phospholipid translocation |
| AN12465 | Ortholog(s) have role in cellular response to biotic stimulus, filamentous growth of a population of unicellular organisms in response to biotic stimulus and mitochondrion localization |
| AN12472 | Has domain(s) with predicted hydrolase activity, hydrolase activity, acting on acid halide bonds, in C-halide compounds activity and role in metabolic process |
| AN12473 | Putative heat shock protein |
| AN12477 | Has domain(s) with predicted GTP binding, GTPase activity |
| AN12478 | IMP dehydrogenase/GMP reductase |
| AN12480 | Ortholog(s) have role in response to wounding and porous cell septum localization |
| AN12487 | protein of unknown function |
| AN1257 | Has domain(s) with predicted transferase activity, transferring glycosyl groups activity |
| AN1265 | Putative transcription factor, similar to S.cerevisiae ZAP1 and A. fumigatus zafA, which are regulators of zinc homeostasis |
| AN1266 | Ortholog(s) have ATPase activity, U1 snRNP binding, U2 snRNP binding activity, role in U2-type prespliceosome assembly and U2-type prespliceosome, cytosol, mitotic spindle pole body localization |
| AN1267 | High-mobility group (HMG) protein with a predicted role in sequence-specific DNA binding, RNA polymerase III transcriptional preinitiation complex assembly and chromatin remodeling |
| AN1276 | Has domain(s) with predicted substrate-specific transmembrane transporter activity, transmembrane transporter activity, role in transmembrane transport and integral component of membrane, membrane localization |
| AN1277 | Protein with alpha-arabinofuranosidase activity, involved in degradation of pectin |
| AN1285 | Endo-beta-1,4-glucanase A |
| AN1292 | Putative diacylglycerol pyrophosphate phosphatase with a predicted role in phospholipid metabolism |
| AN1293 | Ortholog of A. fumigatus Af293 : Afu1g09720, A. niger CBS 513.88 : An08g01500, A. oryzae RIB40 : AO090012000872, Aspergillus wentii : Aspwe1_0103011 and Aspergillus sydowii : Aspsy1_0025452 |
| AN1294 | protein of unknown function |
| AN1296 | Ortholog(s) have 2',3'-cyclic-nucleotide 3'-phosphodiesterase activity, GTP-dependent polyribonucleotide 5'-hydroxyl-kinase activity, RNA ligase (ATP) activity |
| AN1321 | Ortholog of Aspergillus versicolor : Aspve1_0068355 |
| AN1336 | Ortholog of A. fumigatus Af293 : Afu1g09570, A. niger CBS 513.88 : An08g01160, A. oryzae RIB40 : AO090012000913, Aspergillus wentii : Aspwe1_0024569 and Aspergillus sydowii : Aspsy1_0037865 |
| AN1346 | Ortholog of A. fumigatus Af293 : Afu1g09430, A. oryzae RIB40 : AO090012000930, Aspergillus wentii : Aspwe1_0103922, Aspergillus sydowii : Aspsy1_0025400 and Aspergillus terreus NIH2624 : ATET_08566 |
| AN1353 | Ortholog(s) have role in RNA polymerase II transcriptional preinitiation complex assembly and transcription factor TFIID complex localization |
| AN1357 | Putative cholinephosphate cytidylyltransferase with a predicted role in phospholipid metabolism |
| AN1360 | Putative GNAT-type acetyltransferase |
| AN1375 | Ortholog of A. fumigatus Af293 : Afu1g09060, A. oryzae RIB40 : AO090005001619, Aspergillus wentii : Aspwe1_0106093 and Aspergillus sydowii : Aspsy1_0052990 |
| AN1376 | Putative methylene-fatty-acyl-phospholipid synthase with a predicted role in phospholipid metabolism |
| AN1377 | Member of the RGS (regulator of G-protein signaling) family |
| AN1378 | Ortholog of A. fumigatus Af293 : Afu1g09030, A. niger CBS 513.88 : An08g00540, A. oryzae RIB40 : AO090005001622, Aspergillus wentii : Aspwe1_0024518 and Aspergillus sydowii : Aspsy1_0138364 |
| AN1379 | Putative nuclear pore complex protein |
| AN1380 | Ortholog(s) have cytosol localization |
| AN1382 | Ortholog(s) have role in cellular zinc ion homeostasis, endoplasmic reticulum inheritance, protein targeting to nuclear inner membrane, septin ring assembly |
| AN1386 | Ortholog of A. fumigatus Af293 : Afu1g08920, A. niger CBS 513.88 : An08g00410, A. oryzae RIB40 : AO090005001634, Aspergillus wentii : Aspwe1_0036270 and Aspergillus sydowii : Aspsy1_0138746 |
| AN1389 | Ortholog of A. niger CBS 513.88 : An08g00380, A. oryzae RIB40 : AO090005001637, Neosartorya fischeri NRRL 181 : NFIA_016750 and Aspergillus versicolor : Aspve1_0059354 |
| AN1390 | Ortholog(s) have copper chaperone activity and role in cellular copper ion homeostasis, cellular iron ion homeostasis, cellular response to oxidative stress, copper ion transport, regulation of iron ion transmembrane transport |
| AN1391 | Ortholog of A. fumigatus Af293 : Afu1g08870, A. niger CBS 513.88 : An08g00360, A. oryzae RIB40 : AO090005001639, Aspergillus wentii : Aspwe1_0106441 and Aspergillus sydowii : Aspsy1_0141722 |
| AN1392 | Ortholog(s) have NMS complex, Ndc80 complex, condensed nuclear chromosome kinetochore, mitotic spindle pole body localization |
| AN1406 | Has domain(s) with predicted DNA binding, RNA polymerase II transcription factor activity, sequence-specific DNA binding, zinc ion binding activity and role in regulation of transcription, DNA-templated, transcription, DNA-templated |
| AN1417 | protein of unknown function |
| AN1425 | Putative transcription factor containing a Zn2-Cys6 binuclear cluster domain |
| AN1426 | Ortholog(s) have serine-type carboxypeptidase activity |
| AN1427 | Ortholog(s) have N-acetylglucosamine transmembrane transporter activity, role in N-acetylglucosamine transport and cytoplasm, plasma membrane localization |
| AN1428 | Ortholog(s) have N-acetylglucosamine-6-phosphate deacetylase activity |
| AN1430 | Ortholog(s) have betaine-aldehyde dehydrogenase activity |
| AN1435 | Ortholog(s) have DNA replication origin binding, chromatin binding activity |
| AN1456 | Ortholog(s) have mRNA cleavage and polyadenylation specificity factor complex localization |
| AN1463 | Predicted glycosylphosphatidylinositol (GPI)-anchored protein |
| AN1483 | Ortholog of A. fumigatus Af293 : Afu8g04780, A. niger CBS 513.88 : An16g08040, A. oryzae RIB40 : AO090005000669, Aspergillus wentii : Aspwe1_0643990 and Aspergillus sydowii : Aspsy1_0055812 |
| AN1485 | protein kinase |
| AN1486 | Has domain(s) with predicted ribonuclease activity and role in tRNA processing |
| AN1488 | Putative zinc-finger protein with a predicted role in ER unfolded protein degradation |
| AN1494 | Putative TATA binding protein associated factor |
| AN1494-uORF | Conserved upstream open reading frame (uORF) of AN1494 |
| AN1497 | Ortholog of A. fumigatus Af293 : Afu8g04990, A. niger CBS 513.88 : An16g07810, Neosartorya fischeri NRRL 181 : NFIA_097800 and Aspergillus versicolor : Aspve1_0125683 |
| AN1499 | protein of unknown function |
| AN1505 | Ortholog of A. nidulans FGSC A4 : AN3207, A. fumigatus Af293 : Afu2g15470, Afu8g00720, Afu8g05070 and A. niger CBS 513.88 : An07g04980, An16g07680, An14g02720, An14g07130, An15g05540 |
| AN1511 | Putative peroxisomal protein (peroxin) involved in peroxisomal protein import |
| AN1518 | Ortholog(s) have transcription factor activity, RNA polymerase II core promoter proximal region sequence-specific binding activity and role in cellular response to drug, positive regulation of transcription from RNA polymerase II promoter |
| AN1520 | Ortholog of A. fumigatus Af293 : Afu8g05290, A. niger CBS 513.88 : An16g07440, A. oryzae RIB40 : AO090005000620, Aspergillus wentii : Aspwe1_0045135 and Aspergillus sydowii : Aspsy1_0145694 |
| AN1521 | DNA-directed RNA polymerase subunit E' |
| AN1521-uORF | Conserved upstream open reading frame (uORF) of AN1521 |
| AN1522 | Ortholog(s) have role in sphingoid catabolic process and endoplasmic reticulum localization |
| AN1523 | Putative F1F0-ATPase complex subunit with a predicted role in energy metabolism |
| AN1524 | Putative methylenetetrahydrofolate dehydrogenase (NAD+) with a predicted role in one-carbon metabolism |
| AN1532 | Ortholog of A. fumigatus Af293 : Afu8g05410, A. niger CBS 513.88 : An16g07330, A. oryzae RIB40 : AO090005000610, Aspergillus wentii : Aspwe1_0045147 and Aspergillus sydowii : Aspsy1_0041815 |
| AN1533 | Ortholog(s) have role in cellular response to drug, endonucleolytic cleavage in 5'-ETS of tricistronic rRNA transcript (SSU-rRNA, 5.8S rRNA and LSU-rRNA), more |
| AN1535 | Ortholog of A. fumigatus Af293 : Afu8g05450, Neosartorya fischeri NRRL 181 : NFIA_098190, Aspergillus wentii : Aspwe1_0176287 and Aspergillus versicolor : Aspve1_0435110 |
| AN1544 | Ortholog of A. fumigatus Af293 : Afu8g05540, A. niger CBS 513.88 : An16g07140, A. oryzae RIB40 : AO090005000591, Aspergillus wentii : Aspwe1_0045161 and Aspergillus sydowii : Aspsy1_0041791 |
| AN1545 | Putative regulatory subunit of protein phosphatase 2A (PP2A) |
| AN1561 | Predicted glycosylphosphatidylinositol (GPI)-anchored protein |
| AN1563 | Has domain(s) with predicted NAD binding, oxidoreductase activity, acting on the CH-OH group of donors, NAD or NADP as acceptor activity and role in oxidation-reduction process |
| AN1574 | protein of unknown function |
| AN1577 | Has domain(s) with predicted substrate-specific transmembrane transporter activity, transmembrane transporter activity, role in transmembrane transport and integral component of membrane, membrane localization |
| AN1578 | Ortholog of Aspergillus wentii : Aspwe1_0177575, Aspergillus niger ATCC 1015 : 42227-mRNA, Aspergillus sydowii : Aspsy1_0046412 and Aspergillus brasiliensis : Aspbr1_0045436 |
| AN1579 | Ortholog of A. nidulans FGSC A4 : AN12486, A. oryzae RIB40 : AO090003001382 and Neosartorya fischeri NRRL 181 : NFIA_081910 |
| AN1588 | Ortholog of A. fumigatus Af293 : Afu3g13740, Neosartorya fischeri NRRL 181 : NFIA_049880, NFIA_063630, Aspergillus wentii : Aspwe1_0042965 and Aspergillus fumigatus A1163 : AFUB_035450 |
| AN1597 | protein of unknown function |
| AN1609 | protein of unknown function |
| AN1610 | Ortholog of A. fumigatus Af293 : Afu4g09230, A. oryzae RIB40 : AO090023000613, Neosartorya fischeri NRRL 181 : NFIA_106900, Aspergillus wentii : Aspwe1_0166893 and Aspergillus versicolor : Aspve1_0440039 |
| AN1611 | Has domain(s) with predicted FAD binding activity |
| AN1617 | protein of unknown function |
| AN1623 | Has domain(s) with predicted FAD binding, oxidoreductase activity and role in metabolic process |
| AN1625 | Ortholog of A. nidulans FGSC A4 : AN8147, A. fumigatus Af293 : Afu3g01080, Afu3g12760, A. niger CBS 513.88 : An02g08740 and A. oryzae RIB40 : AO090012000846, AO090023000643 |
| AN1628 | Putative membrane ATPase with a predicted role in energy metabolism |
| AN1641 | Ortholog(s) have role in sporocarp development involved in sexual reproduction |
| AN1643 | Ortholog of A. nidulans FGSC A4 : AN1704, AN7062, A. fumigatus Af293 : Afu2g14410, Afu4g01500 and A. niger CBS 513.88 : An18g01050, An04g02710, An09g01370 |
| AN1645 | Has domain(s) with predicted role in blood coagulation, proteolysis and extracellular region localization |
| AN1662 | Ortholog(s) have cytosol, nucleus, ribosome localization |
| AN1666 | Putative nucleolar GTPase |
| AN1683 | Putative oligosaccharyltransferase delta subunit |
| AN1684 | Ortholog of A. fumigatus Af293 : Afu4g08650, A. niger CBS 513.88 : An04g03490, A. oryzae RIB40 : AO090023000691, Aspergillus wentii : Aspwe1_0167016 and Aspergillus sydowii : Aspsy1_0026068 |
| AN1686 | Ortholog of A. fumigatus Af293 : Afu4g08620, A. niger CBS 513.88 : An04g03440, Neosartorya fischeri NRRL 181 : NFIA_107560, Aspergillus wentii : Aspwe1_0079295 and Aspergillus versicolor : Aspve1_0124141 |
| AN1688 | Ortholog of S. cerevisiae : YBR053C, A. fumigatus Af293 : Afu4g08610, A. niger CBS 513.88 : An04g03420, A. oryzae RIB40 : AO090023000696 and Aspergillus wentii : Aspwe1_0034438 |
| AN1689 | putative oxidoreductase |
| AN1693 | Putative F-box protein |
| AN1696 | Has domain(s) with predicted transferase activity, transferring acyl groups other than amino-acyl groups activity |
| AN1697 | Ortholog(s) have role in cellular response to drug |
| AN1705 | Has domain(s) with predicted RNA polymerase II transcription factor activity, sequence-specific DNA binding, nucleic acid binding, zinc ion binding activity, role in regulation of transcription, DNA-templated and nucleus localization |
| AN1719 | Ortholog of A. fumigatus Af293 : Afu4g08370, A. niger CBS 513.88 : An04g03180, A. oryzae RIB40 : AO090701000366 and Aspergillus wentii : Aspwe1_0102057, Aspwe1_0436878 |
| AN1724 | Has domain(s) with predicted role in transmembrane transport and integral component of membrane localization |
| AN1725 | Ortholog of S. cerevisiae : YEL023C, A. nidulans FGSC A4 : AN5027, A. fumigatus Af293 : Afu2g14400, Afu3g12130 and A. niger CBS 513.88 : An11g08400, An02g07940, An12g01310, An02g00780 |
| AN1729 | Transcriptional activator from the zinc binuclear cluster family, regulates the four gene cluster (prn) involved in proline utilization |
| AN1733 | Putative delta-1-pyrroline-5-carboxylate dehydrogenase with a predicted role in glutamate and glutamine metabolism |
| AN1738 | Ortholog of A. nidulans FGSC A4 : AN6413, AN6419, AN6946, AN5312, AN5664, AN8328, AN3241, AN10369, AN1930, AN2386, AN11159, AN8984, AN8951, AN1540, AN8971, AN9387, AN9266, AN9306, AN1317, AN10886, AN7232, AN7774, AN12202 |
| AN1739 | protein of unknown function |
| AN1745 | Ortholog(s) have cytosol, nucleus localization |
| AN1747 | Ortholog(s) have mitochondrial inner membrane localization |
| AN1750 | Ortholog(s) have nucleolus localization |
| AN1752 | Putative sulfite reductase with a predicted role in sulfur metabolism |
| AN1753 | Ortholog(s) have endoplasmic reticulum, mitochondrial outer membrane localization |
| AN1757 | Predicted alpha 3 subunit of the 20S core proteasome |
| AN1760 | Ortholog(s) have role in macroautophagy and cytosol, nucleus localization |
| AN1761 | Ortholog(s) have ubiquitin-protein transferase activity |
| AN1769 | Putative 3'-5' bisphosphate nucleotidase with a predicted role in cysteine metabolism |
| AN1770 | Ortholog(s) have structural constituent of ribosome activity and mitochondrial large ribosomal subunit, nucleus localization |
| AN1773 | Ortholog of A. fumigatus Af293 : Afu6g09030, A. niger CBS 513.88 : An11g06790, A. oryzae RIB40 : AO090001000581, Aspergillus wentii : Aspwe1_0121600 and Aspergillus sydowii : Aspsy1_0038822 |
| AN1774 | Ortholog of Aspergillus versicolor : Aspve1_0090039 and Aspergillus sydowii : Aspsy1_1165308 |
| AN1777 | Ortholog(s) have nucleus localization |
| AN1779 | Essential protein kinase required for DNA synthesis and mitotic checkpoint control |
| AN1780 | Ortholog(s) have aminopeptidase activity, role in protein processing, protein stabilization and extrinsic component of mitochondrial inner membrane, nucleus localization |
| AN1781 | Has domain(s) with predicted FMN binding, oxidoreductase activity and role in oxidation-reduction process |
| AN1786 | Predicted monooxygenase |
| AN1788 | Has domain(s) with predicted metal ion transmembrane transporter activity, role in metal ion transport, transmembrane transport and membrane localization |
| AN1790 | protein of unknown function |
| AN1797 | Ortholog(s) have fructose transmembrane transporter activity, glucose transmembrane transporter activity, mannose transmembrane transporter activity |
| AN1800 | Transmembrane histidine kinase, part of a two-component signal transducer involved in the HOG signaling pathway that regulates osmotic stress response' transcript upregulated by growth in glycerol |
| AN1805 | Putative carbonic anhydrase |
| AN1807 | Ortholog of A. fumigatus Af293 : Afu1g17540, A. oryzae RIB40 : AO090009000305, Neosartorya fischeri NRRL 181 : NFIA_003200, Aspergillus wentii : Aspwe1_0108466 and Aspergillus versicolor : Aspve1_0123605 |
| AN1808 | Has domain(s) with predicted oxidoreductase activity and role in oxidation-reduction process |
| AN1829 | Has domain(s) with predicted RNA polymerase II transcription factor activity, sequence-specific DNA binding, zinc ion binding activity, role in regulation of transcription, DNA-templated and nucleus localization |
| AN1837 | Putative hydrophobin |
| AN1860 | Ortholog(s) have role in ubiquinone-6 biosynthetic process and mitochondrion localization |
| AN1861 | Ortholog(s) have Prp19 complex, spliceosomal complex localization |
| AN1868 | Putative glycerol dehydrogenase with a predicted role in glycerol metabolism |
| AN1892 | Ortholog(s) have protein channel activity, role in mitochondrial genome maintenance, protein import into mitochondrial matrix and mitochondrial inner membrane presequence translocase complex localization |
| AN1895 | Maleyl-acetoacetate isomerase, enzyme involved in phenylalanine catabolism |
| AN1896 | Fumarylacetoacetate hydrolase, catalyzes the last step in the phenylalanine catabolic pathway |
| AN1898 | Ortholog(s) have role in melanin biosynthetic process from tyrosine, tyrosine catabolic process and cytoplasm localization |
| AN1899 | Putative 4-hydroxyphenylpyruvate dioxygenase with a predicted role in aromatic amino acid biosynthesis |
| AN1902 | Ortholog of A. fumigatus Af293 : Afu2g04170, A. niger CBS 513.88 : An11g02270, A. oryzae RIB40 : AO090003000203, Aspergillus wentii : Aspwe1_0032670 and Aspergillus sydowii : Aspsy1_0026333 |
| AN1903 | protein of unknown function |
| AN1918 | Putative phosphoenolpyruvate carboxykinase with a predicted role in gluconeogenesis and glycolysis |
| AN1925 | protein of unknown function |
| AN1929 | Ortholog(s) have DNA translocase activity, chromatin binding activity |
| AN1937 | Regulatory protein involved in conidial development |
| AN1945 | Ortholog(s) have cytoplasm, nucleus localization |
| AN1946 | Ortholog of A. fumigatus Af293 : Afu4g13300, A. niger CBS 513.88 : An01g09010, A. oryzae RIB40 : AO090009000247, Aspergillus wentii : Aspwe1_0208317 and Aspergillus sydowii : Aspsy1_0138807 |
| AN1948 | Ortholog(s) have role in DNA methylation |
| AN1950 | Ortholog(s) have FAD transmembrane transporter activity, calcium channel activity |
| AN1951 | Has domain(s) with predicted nucleic acid binding, zinc ion binding activity |
| AN1956 | Ortholog(s) have ATP binding, ATPase activity |
| AN1959 | Nuclear protein involved in spore formation and trehalose accumulation |
| AN1962 | Ortholog of A. fumigatus Af293 : Afu4g10820, A. niger CBS 513.88 : An04g05820, Aspergillus wentii : Aspwe1_0145558, Aspergillus sydowii : Aspsy1_0142548 and Aspergillus terreus NIH2624 : ATET_03987 |
| AN1963 | Has domain(s) with predicted hydrolase activity, hydrolase activity, acting on ester bonds activity and role in lipid metabolic process |
| AN1964 | Ortholog of S. cerevisiae RPS6B and RPS6A |
| AN1969 | Ortholog(s) have alpha-1,6-mannosyltransferase activity, role in protein N-linked glycosylation and alpha-1,6-mannosyltransferase complex, endoplasmic reticulum localization |
| AN1971 | Ortholog(s) have ATP-dependent 3'-5' DNA helicase activity, ATP-dependent 5'-3' DNA helicase activity, sequence-specific DNA binding activity |
| AN1987 | Ortholog of A. fumigatus Af293 : Afu4g10500, A. niger CBS 513.88 : An04g06170, A. oryzae RIB40 : AO090003001161, Aspergillus wentii : Aspwe1_0047460 and Aspergillus sydowii : Aspsy1_0316623 |
| AN1992 | Ortholog(s) have role in mRNA cis splicing, via spliceosome and U4/U6 x U5 tri-snRNP complex localization |
| AN2000 | Polyubiquitin, contains four head to tail repeats of ubiqiutin |
| AN2002 | Protein predicted to have a role in pheromone precursor processing |
| AN20053 | protein of unknown function |
| AN20055 | protein of unknown function |
| AN2006 | Ortholog of A. fumigatus Af293 : Afu4g10260, A. niger CBS 513.88 : An04g06660, A. oryzae RIB40 : AO090003001196, Aspergillus wentii : Aspwe1_0034162 and Aspergillus sydowii : Aspsy1_0142465 |
| AN2008 | Ortholog(s) have phosphatidylglycerophosphatase activity, role in cardiolipin biosynthetic process, phosphorylated carbohydrate dephosphorylation and mitochondrial matrix localization |
| AN2011 | Ortholog(s) have phospholipid-translocating ATPase activity and role in intracellular protein transport, phospholipid translocation, response to pheromone involved in conjugation with cellular fusion |
| AN2034 | protein of unknown function |
| AN2036 | putative transcription factor |
| AN2040 | Putative cytochrome P450 |
| AN2041 | Ortholog of A. niger CBS 513.88 : An16g01460, An07g04410, An16g06600, An11g04970, An03g01890, A. oryzae RIB40 : AO090026000564 and Neosartorya fischeri NRRL 181 : NFIA_066540, NFIA_083780 |
| AN2042 | Ortholog(s) have cytosol localization |
| AN2047 | Calmodulin |
| AN2048 | Protein similar to S. cerevisiae Tlg2p |
| AN2053 | Putative nucleoside transporter |
| AN2057 | Ortholog(s) have structural constituent of ribosome activity and mitochondrial large ribosomal subunit, mitochondrial nucleoid localization |
| AN2068 | Ortholog(s) have cytosol, endoplasmic reticulum, nuclear outer membrane-endoplasmic reticulum membrane network localization |
| AN2077 | Ortholog(s) have cytosol, nucleus localization |
| AN2079 | Ortholog(s) have role in maturation of SSU-rRNA from tricistronic rRNA transcript (SSU-rRNA, 5.8S rRNA, LSU-rRNA) and cytosol, nucleus localization |
| AN2093 | Ortholog(s) have Rab guanyl-nucleotide exchange factor activity, phosphatidylinositol-3-phosphate binding, phosphatidylinositol-5-phosphate binding, phosphatidylserine binding activity |
| AN2102 | Putative GNAT-type acetyltransferase |
| AN2103 | Ortholog(s) have role in carbon utilization, mitochondrial respiratory chain complex II assembly, regulation of gluconeogenesis and mitochondrial intermembrane space localization |
| AN2115 | Ortholog of A. nidulans FGSC A4 : AN10483, AN2809, AN0033, AN12460, A. fumigatus Af293 : Afu2g00940, Afu2g16560, Afu3g15340, Afu7g00460, Afu7g06360 and A. niger CBS 513.88 : An14g02830, An16g00830 |
| AN2118 | Has domain(s) with predicted catalytic activity and role in metabolic process |
| AN2119 | Ortholog(s) have sphingolipid transporter activity, role in sphingolipid metabolic process and fungal-type vacuole membrane localization |
| AN2121 | Has domain(s) with predicted proline racemase activity |
| AN2126 | Predicted actin capping protein |
| AN2135 | Has domain(s) with predicted DNA binding, DNA-directed RNA polymerase activity, role in transcription from RNA polymerase III promoter and DNA-directed RNA polymerase III complex localization |
| AN2144 | Ortholog(s) have cytosol, nucleus localization |
| AN2156 | protein of unknown function |
| AN2161 | Putative GNAT-type acetyltransferase |
| AN2162 | Has domain(s) with predicted zinc ion binding activity |
| AN2177 | Has domain(s) with predicted oxidoreductase activity and role in metabolic process |
| AN2182 | Ortholog of A. fumigatus Af293 : Afu2g15670, A. niger CBS 513.88 : An15g05910, Aspergillus wentii : Aspwe1_0039157, Aspergillus sydowii : Aspsy1_0139789 and Aspergillus terreus NIH2624 : ATET_02443 |
| AN2187 | protein of unknown function |
| AN2188 | Ortholog of A. fumigatus Af293 : Afu7g03930, A. niger CBS 513.88 : An13g00390, A. oryzae RIB40 : AO090026000821, Aspergillus wentii : Aspwe1_0050251 and Aspergillus sydowii : Aspsy1_0026945 |
| AN2209 | Ortholog of A. fumigatus Af293 : Afu5g07010, A. niger CBS 513.88 : An17g00230, A. oryzae RIB40 : AO090701000283, Aspergillus wentii : Aspwe1_0269169 and Aspergillus sydowii : Aspsy1_0054348 |
| AN2214 | Ortholog of A. fumigatus Af293 : Afu5g07060, A. niger CBS 513.88 : An17g00280, A. oryzae RIB40 : AO090701000278, Neosartorya fischeri NRRL 181 : NFIA_080200 and Aspergillus versicolor : Aspve1_0080700 |
| AN2220 | protein of unknown function |
| AN2226 | Ortholog(s) have nucleus localization |
| AN2230 | Has domain(s) with predicted oxidoreductase activity and role in oxidation-reduction process |
| AN2231 | Has domain(s) with predicted nucleic acid binding activity |
| AN2236 | Ortholog of A. fumigatus Af293 : Afu5g07310, A. niger CBS 513.88 : An17g00750, A. oryzae RIB40 : AO090701000224, Aspergillus wentii : Aspwe1_0048016 and Aspergillus sydowii : Aspsy1_0141770 |
| AN2237 | Putative carboxypeptidase C |
| AN2240 | Ortholog(s) have mannosyltransferase activity and role in ER-associated ubiquitin-dependent protein catabolic process, GPI anchor biosynthetic process, protein processing |
| AN2250 | Has domain(s) with predicted oxidoreductase activity and role in metabolic process |
| AN2259 | Ortholog of A. fumigatus Af293 : Afu5g06550, A. niger CBS 513.88 : An17g01100, A. oryzae RIB40 : AO090701000188, Aspergillus wentii : Aspwe1_0023497 and Aspergillus sydowii : Aspsy1_0054289 |
| AN2268 | Ortholog(s) have structural constituent of ribosome activity and mitochondrial large ribosomal subunit localization |
| AN2270 | C2H2 zinc-finger transcription factor involved in regulation of structural genes for acetamidase (amdS), formate dehydrogenase (aciA), and alcohol dehydrogenase II (alcB) |
| AN2274 | Ortholog of A. fumigatus Af293 : Afu5g06370, A. niger CBS 513.88 : An17g01350, A. oryzae RIB40 : AO090009000619, Aspergillus wentii : Aspwe1_0056514 and Aspergillus sydowii : Aspsy1_1156792 |
| AN2278 | Ortholog(s) have role in cellular response to biotic stimulus, cellular response to neutral pH and filamentous growth of a population of unicellular organisms in response to biotic stimulus, more |
| AN2282 | Putative MFS transporter |
| AN2288 | Ortholog(s) have potassium:proton antiporter activity, sodium:proton antiporter activity and role in potassium ion transmembrane transport, sodium ion transmembrane transport, vacuolar acidification |
| AN2290 | STE-like transcription factor with homeobox and zinc finger domains |
| AN2291 | Predicted DDE1 transposon-related ORF |
| AN2298 | SumO activating (E1) enzyme |
| AN2302 | Skp1-related protein with a predicted role in sulfur metabolism |
| AN2303 | Ortholog(s) have alpha-1,2-mannosyltransferase activity, dolichyl-phosphate-mannose-glycolipid alpha-mannosyltransferase activity and role in GPI anchor biosynthetic process, fungal-type cell wall biogenesis, plasmid maintenance |
| AN2318 | Ortholog of A. fumigatus Af293 : Afu5g10580, A. niger CBS 513.88 : An14g04140, A. oryzae RIB40 : AO090010000479, Aspergillus wentii : Aspwe1_0106828 and Aspergillus sydowii : Aspsy1_0026806 |
| AN2335 | Has domain(s) with predicted coenzyme binding, oxidoreductase activity, acting on the CH-OH group of donors, NAD or NADP as acceptor, phosphogluconate dehydrogenase (decarboxylating) activity |
| AN2337 | Ortholog of Neosartorya fischeri NRRL 181 : NFIA_077020, Aspergillus wentii : Aspwe1_0170563, Aspergillus clavatus NRRL 1 : ACLA_013280 and Aspergillus niger ATCC 1015 : 53845-mRNA |
| AN2338 | Ortholog of A. fumigatus Af293 : Afu5g10240, A. oryzae RIB40 : AO090020000142, AO090010000531, Neosartorya fischeri NRRL 181 : NFIA_077030 and Aspergillus wentii : Aspwe1_0033922, Aspwe1_0038721 |
| AN2346 | protein of unknown function |
| AN2349 | Putative ATP-binding cassette (ABC) transporter of the P-glycoprotein cluster |
| AN2351 | Putative zinc-containing alcohol dehydrogenase |
| AN2359 | Beta-xylosidase, involved in degradation of xylans |
| AN2367 | Putative Zn(II)2Cys6 transcription factor |
| AN2368 | Has domain(s) with predicted role in transmembrane transport and integral component of membrane localization |
| AN2381 | Ortholog of A. nidulans FGSC A4 : AN8456 and Neosartorya fischeri NRRL 181 : NFIA_099730 |
| AN2387 | Has domain(s) with predicted UDP-N-acetylmuramate dehydrogenase activity, flavin adenine dinucleotide binding activity and role in oxidation-reduction process |
| AN2388 | Putative beta-1,4-endoglucanase |
| AN2390 | Has domain(s) with predicted oxidoreductase activity, acting on paired donors, with incorporation or reduction of molecular oxygen, 2-oxoglutarate as one donor, and incorporation of one atom each of oxygen into both donors activity |
| AN2391 | Ortholog of A. fumigatus Af293 : Afu2g14510, A. niger CBS 513.88 : An02g00660, An07g03040, A. oryzae RIB40 : AO090023000055, AO090026000077, AO090020000644 and Aspergillus wentii : Aspwe1_0163838 |
| AN2393 | Ortholog(s) have cytosol localization |
| AN2395 | Putative beta-glucuronidase with a predicted role in polysaccharide degradation |
| AN2397 | Ortholog of A. fumigatus Af293 : Afu8g06960, Aspergillus flavus NRRL 3357 : AFL2T_05473, Neosartorya fischeri NRRL 181 : NFIA_099650 and Aspergillus versicolor : Aspve1_0026050 |
| AN2399 | protein of unknown function |
| AN2400 | protein of unknown function |
| AN2404 | Ortholog of A. fumigatus Af293 : Afu8g05810, A. niger CBS 513.88 : An15g04670, A. oryzae RIB40 : AO090005000570, Aspergillus wentii : Aspwe1_0118486 and Aspergillus sydowii : Aspsy1_0039439 |
| AN2452 | Has domain(s) with predicted DNA binding activity, role in DNA replication and nuclear origin of replication recognition complex localization |
| AN2453 | Putative peptidyl-prolyl cis-trans isomerase (PPIase) |
| AN2463 | Putative beta-galactosidase with a predicted role in lactose metabolism |
| AN2466 | Has domain(s) with predicted substrate-specific transmembrane transporter activity, transmembrane transporter activity, role in transmembrane transport and integral component of membrane, membrane localization |
| AN2468 | Has domain(s) with predicted amino acid transmembrane transporter activity, role in amino acid transmembrane transport, amino acid transport, transmembrane transport and integral component of membrane, membrane localization |
| AN2471 | Ortholog of A. niger CBS 513.88 : An02g07790, Neosartorya fischeri NRRL 181 : NFIA_065240, Aspergillus clavatus NRRL 1 : ACLA_039940, ACLA_078340 and Aspergillus niger ATCC 1015 : 144091-mRNA |
| AN2474 | Has domain(s) with predicted GTP binding, GTPase activity and role in intracellular protein transport, nucleocytoplasmic transport, protein transport, signal transduction, small GTPase mediated signal transduction |
| AN2475 | putative sugar transporter |
| AN2476 | Ortholog of A. fumigatus Af293 : Afu4g03130, A. niger CBS 513.88 : An14g02240, A. oryzae RIB40 : AO090102000251, Aspergillus wentii : Aspwe1_0116252 and Aspergillus sydowii : Aspsy1_0158425 |
| AN2479 | Putative GNAT-type acetyltransferase |
| AN2481 | Ortholog(s) have lysophospholipase activity, role in phosphatidylcholine catabolic process, regulation of phospholipid biosynthetic process and endoplasmic reticulum localization |
| AN2488 | Ortholog of A. fumigatus Af293 : Afu3g00500, Afu4g03010, A. niger CBS 513.88 : An14g03080, A. oryzae RIB40 : AO090012000174 and Aspergillus wentii : Aspwe1_0176515, Aspwe1_0622364 |
| AN2490 | Has domain(s) with predicted ubiquitin-protein transferase activity, zinc ion binding activity |
| AN2491 | Ortholog(s) have DNA replication origin binding, chromatin binding, single-stranded DNA binding, single-stranded DNA-dependent ATP-dependent DNA helicase activity |
| AN2510 | Ortholog(s) have cytosol, nucleus localization |
| AN2511 | Putative spindle assembly checkpoint protein that localizes to the nuclear pore complex |
| AN2526 | Putative ketol-acid reductoisomerase with a predicted role in Coenzyme A and pantothenate biosynthesis or amino acid metabolism |
| AN2540 | Ortholog of Aspergillus versicolor : Aspve1_0051083 and Aspergillus sydowii : Aspsy1_0087014 |
| AN2543 | Putative rhamnogalacturonan lyase |
| AN2546 | Predicted glycosylphosphatidylinositol (GPI)-anchored protein |
| AN2568 | Ortholog of Aspergillus tubingensis : Asptu1_0047536, Aspergillus brasiliensis : Aspbr1_0129643, Aspergillus kawachii : Aspka1_0176839 and Aspergillus acidus : Aspfo1_0137686 |
| AN2574 | Has domain(s) with predicted UDP-N-acetylmuramate dehydrogenase activity, flavin adenine dinucleotide binding activity and role in oxidation-reduction process |
| AN2586 | Has domain(s) with predicted catalytic activity and role in metabolic process |
| AN2587 | Ortholog of A. nidulans FGSC A4 : AN5639, AN9444, AN7395, A. fumigatus Af293 : Afu5g00840, A. niger CBS 513.88 : An03g01000 and A. oryzae RIB40 : AO090102000018 |
| AN2588 | Ortholog of Aspergillus glaucus : Aspgl1_0026529, Aspergillus versicolor : Aspve1_0047339 and Aspergillus sydowii : Aspsy1_0049555 |
| AN2590 | Has domain(s) with predicted catalytic activity, heme binding, oxidoreductase activity |
| AN2595 | protein of unknown function |
| AN2605 | protein of unknown function |
| AN2611 | Has domain(s) with predicted role in isoprenoid biosynthetic process |
| AN2634 | Has domain(s) with predicted catalytic activity and role in metabolic process |
| AN2636 | Putative F-box protein |
| AN2642 | protein of unknown function |
| AN2644 | Has domain(s) with predicted DNA binding, RNA polymerase II transcription factor activity, sequence-specific DNA binding, zinc ion binding activity and role in regulation of transcription, DNA-templated, transcription, DNA-templated |
| AN2648 | Has domain(s) with predicted UDP-N-acetylmuramate dehydrogenase activity, flavin adenine dinucleotide binding, oxidoreductase activity and role in oxidation-reduction process |
| AN2649 | Ortholog of A. fumigatus Af293 : Afu1g01190, A. niger CBS 513.88 : An11g03730, A. oryzae RIB40 : AO090020000652, Aspergillus wentii : Aspwe1_0741100 and Aspergillus sydowii : Aspsy1_0134175 |
| AN2651 | Ortholog(s) have phospholipid-translocating ATPase activity, role in fatty acid transport, phospholipid translocation and endoplasmic reticulum, integral component of membrane, plasma membrane localization |
| AN2655 | Has domain(s) with predicted iron ion binding, oxidoreductase activity, oxidoreductase activity, acting on paired donors, with incorporation or reduction of molecular oxygen and 2-oxoglutarate as one donor, more |
| AN2656 | Has domain(s) with predicted role in transmembrane transport and integral component of membrane localization |
| AN2669 | Has domain(s) with predicted role in response to stress and integral component of membrane localization |
| AN2672 | Has domain(s) with predicted DNA binding, RNA polymerase II transcription factor activity, sequence-specific DNA binding, zinc ion binding activity and role in regulation of transcription, DNA-templated, transcription, DNA-templated |
| AN2674 | Has domain(s) with predicted catalytic activity and role in metabolic process |
| AN2675 | Has domain(s) with predicted role in transmembrane transport and integral component of membrane localization |
| AN2689 | SUMO-specific isopeptidase |
| AN2692 | Phosphoprotein regulated by proline-directed phosphorylation |
| AN2698 | Has domain(s) with predicted oxidoreductase activity and role in oxidation-reduction process |
| AN2709 | Putative beta-1,4-endoglucanase |
| AN2715 | Has domain(s) with predicted oxidoreductase activity and role in metabolic process |
| AN2728 | Has domain(s) with predicted catalytic activity, lyase activity and role in metabolic process |
| AN2729 | Has domain(s) with predicted role in transmembrane transport and integral component of membrane localization |
| AN2730 | Putative transporter with a predicted role in small molecule transport |
| AN2742 | Gamma subunit of a heterotrimeric G protein composed of FadA, SfaD, GpgA and involved in regulation of proliferation and development |
| AN2743 | Ortholog(s) have role in regulation of translation in response to nitrogen starvation |
| AN2746 | Has domain(s) with predicted role in transmembrane transport and integral component of membrane localization |
| AN2748 | Ortholog(s) have cytosol, nucleus localization |
| AN2750 | Ortholog of A. fumigatus Af293 : Afu1g05140, A. niger CBS 513.88 : An01g04360, A. oryzae RIB40 : AO090003000810, Aspergillus wentii : Aspwe1_0181081 and Aspergillus sydowii : Aspsy1_0059496 |
| AN2759 | Ortholog(s) have rRNA (pseudouridine) methyltransferase activity |
| AN2760 | Ortholog of A. fumigatus Af293 : Afu3g06020, A. niger CBS 513.88 : An11g10520, A. oryzae RIB40 : AO090020000072, Aspergillus wentii : Aspwe1_0052504 and Aspergillus sydowii : Aspsy1_0056680 |
| AN2769 | Subunit of the SAGA transcriptional regulatory complex |
| AN2774 | Has domain(s) with predicted transferase activity, transferring glycosyl groups activity |
| AN2782 | Has domain(s) with predicted RNA polymerase II transcription factor activity, sequence-specific DNA binding, transcription factor activity, sequence-specific DNA binding, zinc ion binding activity |
| AN2786 | Ortholog(s) have L-pipecolate oxidase activity, flavin adenine dinucleotide binding, proline oxidase activity and cytosol, nucleus localization |
| AN2802 | Ortholog of Aspergillus versicolor : Aspve1_0030904 and Aspergillus sydowii : Aspsy1_0090141 |
| AN2803 | protein of unknown function |
| AN2815 | Putative mannitol 2-dehydrogenase with a predicted role in mannose/mannitol, fructose, and sorbose/sorbitol metabolism |
| AN2818 | Ortholog of A. niger CBS 513.88 : An12g03370, An18g00460, An07g00580, A. oryzae RIB40 : AO090103000478, Aspergillus wentii : Aspwe1_0058229, Aspwe1_0111514 and Aspergillus versicolor : Aspve1_0051884 |
| AN2823 | Has domain(s) with predicted ion channel activity, role in transport and integral component of membrane localization |
| AN2831 | Has domain(s) with predicted role in transmembrane transport and integral component of membrane localization |
| AN2832 | Ortholog of Aspergillus sydowii : Aspsy1_0060442 |
| AN2839 | Has domain(s) with predicted DNA binding, RNA polymerase II transcription factor activity, sequence-specific DNA binding, zinc ion binding activity and role in regulation of transcription, DNA-templated, transcription, DNA-templated |
| AN2845 | Putative component of the EKC/KEOPS complex |
| AN2853 | Ortholog(s) have cytosol, nucleus localization |
| AN2854 | Ortholog(s) have DNA replication origin binding, RNA polymerase II core promoter proximal region sequence-specific DNA binding, centromeric DNA binding and transcriptional activator activity, more |
| AN2858 | Has domain(s) with predicted oxidoreductase activity |
| AN2859 | Has domain(s) with predicted catalytic activity, lyase activity and role in metabolic process |
| AN2867 | Putative phosphoglucomutase with a predicted role in carbohydrate metabolism |
| AN2871 | CUE-domain containing phosphoprotein that enters the nucleus during mitosis |
| AN2872 | protein of unknown function |
| AN2873 | Putative saccharopine dehydrogenase (NAD+, L-lysine-forming) with a predicted role in lysine metabolism |
| AN2877 | Ortholog(s) have oxysterol binding, phosphatidic acid binding, phosphatidylinositol-4,5-bisphosphate binding, phosphatidylinositol-4-phosphate binding, sterol transporter activity |
| AN2886 | Essential protein interacting with kinesin KipA, localized to kinetochore |
| AN2895 | Has domain(s) with predicted ADP binding activity |
| AN2896 | Has domain(s) with predicted catalytic activity and role in metabolic process |
| AN2901 | arginase, putative |
| AN2903 | Aspartic protease |
| AN2912 | Ortholog of A. niger CBS 513.88 : An02g12880, Neosartorya fischeri NRRL 181 : NFIA_069420, Aspergillus versicolor : Aspve1_0081767 and Aspergillus clavatus NRRL 1 : ACLA_035690 |
| AN2922 | Ortholog of A. nidulans FGSC A4 : AN1515, AN7536, A. niger CBS 513.88 : An12g10030, A. oryzae RIB40 : AO090701000305, AO090102000038, AO090124000043 and Aspergillus wentii : Aspwe1_0030521 |
| AN2923 | Ortholog of A. nidulans FGSC A4 : AN10601, A. fumigatus Af293 : Afu2g17770, Afu8g00650, A. niger CBS 513.88 : An11g02090, An01g01000 and A. oryzae RIB40 : AO090020000174, AO090103000301 |
| AN2924 | Putative nonribosomal peptide synthetase (NRPS)-like enzyme |
| AN2925 | Putative peroxisomal import protein (peroxin) with a role in fatty acid utilization |
| AN2926 | Putative 60S ribosomal protein |
| AN2928 | Ortholog of A. fumigatus Af293 : Afu3g08110, A. niger CBS 513.88 : An02g11620, A. oryzae RIB40 : AO090005001467, Aspergillus wentii : Aspwe1_0078084 and Aspergillus terreus NIH2624 : ATET_01658 |
| AN2936 | Putative Class 2C alpha-mannosidase with a predicted role in mannose polymer metabolism |
| AN2938 | Ortholog(s) have 8-oxo-7,8-dihydroguanosine triphosphate pyrophosphatase activity, role in DNA repair and peroxisome localization |
| AN2939 | Ortholog(s) have mitochondrion, plasma membrane localization |
| AN2954 | Ortholog of A. fumigatus Af293 : Afu3g07870, A. niger CBS 513.88 : An02g11390, A. oryzae RIB40 : AO090005001484, Aspergillus wentii : Aspwe1_0029219 and Aspergillus sydowii : Aspsy1_0042610 |
| AN2955 | Has domain(s) with predicted role in biosynthetic process |
| AN2969 | Ortholog(s) have DNA clamp loader activity and role in UV-damage excision repair, leading strand elongation, mitotic DNA replication checkpoint, sister chromatid cohesion |
| AN2977 | Putative transporter with a predicted role in small molecule transport |
| AN2982 | Ortholog of A. niger CBS 513.88 : An02g12160, A. oryzae RIB40 : AO090005001426, Aspergillus wentii : Aspwe1_0135673, Aspergillus sydowii : Aspsy1_0042531 and Aspergillus terreus NIH2624 : ATET_01622 |
| AN2987 | Has domain(s) with predicted oxidoreductase activity and role in metabolic process |
| AN2988 | Ortholog(s) have gamma-glutamylcyclotransferase activity, role in glutathione catabolic process and cytosol, nucleus localization |
| AN2994 | Ortholog of A. niger CBS 513.88 : An02g12350, Aspergillus tubingensis : Asptu1_0118593, Aspergillus glaucus : Aspgl1_0925168 and Aspergillus flavus NRRL 3357 : AFL2T_01318 |
| AN2999 | isocitrate dehydrogenase (NADP+), putative |
| AN3001 | Has domain(s) with predicted ATP binding, protein kinase activity, protein tyrosine kinase activity and role in protein phosphorylation |
| AN3002 | Ortholog of A. fumigatus Af293 : Afu3g08720, A. niger CBS 513.88 : An02g12480, A. oryzae RIB40 : AO090005001396, Aspergillus wentii : Aspwe1_0113467 and Aspergillus sydowii : Aspsy1_0077171 |
| AN3004 | Ortholog(s) have role in cellular response to oxidative stress and cytosol, mitochondrion, nucleus localization |
| AN3007 | protein of unknown function |
| AN3010 | Ortholog(s) have tRNA (guanine(9)-N(1))-methyltransferase activity, role in tRNA N1-guanine methylation and cytoplasm, nucleolus localization |
| AN3022 | Ortholog of A. fumigatus Af293 : Afu3g08900, A. niger CBS 513.88 : An16g02430, A. oryzae RIB40 : AO090005001346, Aspergillus wentii : Aspwe1_0041802 and Aspergillus sydowii : Aspsy1_0042503 |
| AN3027 | Ortholog(s) have flavin-linked sulfhydryl oxidase activity and role in cellular iron ion homeostasis, cellular response to oxidative stress, protein import into mitochondrial intermembrane space |
| AN3028 | Ortholog of A. fumigatus Af293 : Afu3g08860, A. niger CBS 513.88 : An16g02480, A. oryzae RIB40 : AO090005001356, Aspergillus wentii : Aspwe1_0041794 and Aspergillus sydowii : Aspsy1_0077129 |
| AN3031 | Putative threonine synthase with a predicted role in glycine, serine, and threonine metabolism |
| AN3032 | Predicted glycosylphosphatidylinositol (GPI)-anchored protein |
| AN3033 | Ortholog(s) have cytoplasm, nuclear chromatin localization |
| AN3034 | Suppressor of prgA1 mutation |
| AN3035 | Has domain(s) with predicted DNA binding, catalytic activity, nuclease activity and role in DNA repair |
| AN3041 | Has domain(s) with predicted role in transmembrane transport and integral component of membrane localization |
| AN3050 | Has domain(s) with predicted DNA binding, RNA polymerase II transcription factor activity, sequence-specific DNA binding, zinc ion binding activity and role in regulation of transcription, DNA-templated, transcription, DNA-templated |
| AN3058 | Putative glycine hydroxymethyltransferase with a predicted role in glycine, serine, and threonine metabolism |
| AN3059 | Putative phosphoglycerate mutase with a predicted role in gluconeogenesis and glycolysis |
| AN3062 | Putative pericentrin-related protein involved in microtubule organization and hyphal polarity |
| AN3073 | Putative asparaginyl-tRNA synthetase with a predicted role in tRNA charging for translation |
| AN3079 | Secreted thaumatin-like protein |
| AN3081 | Ortholog(s) have Golgi apparatus, cell division site, cell tip localization |
| AN3082 | Ortholog(s) have mRNA cleavage and polyadenylation specificity factor complex localization |
| AN3083 | Has domain(s) with predicted UDP-N-acetylmuramate dehydrogenase activity, flavin adenine dinucleotide binding activity and role in oxidation-reduction process |
| AN3085 | Ortholog(s) have role in cellular response to biotic stimulus, cellular response to starvation and filamentous growth of a population of unicellular organisms in response to biotic stimulus, more |
| AN3098 | Putative secretory component |
| AN3108 | Has domain(s) with predicted RNA polymerase II transcription factor activity, sequence-specific DNA binding, zinc ion binding activity, role in regulation of transcription, DNA-templated and nucleus localization |
| AN3113 | UDP-galactofuranose transporter |
| AN3116 | Ortholog(s) have transcription factor TFIIIB complex localization |
| AN3117 | Ortholog(s) have copper-exporting ATPase activity, role in cadmium ion transport, cellular copper ion homeostasis, copper ion transport, silver ion transport and plasma membrane localization |
| AN3119 | Has domain(s) with predicted catalytic activity, coenzyme binding activity and role in cellular metabolic process |
| AN3120 | Has domain(s) with predicted DNA binding, RNA polymerase II transcription factor activity, sequence-specific DNA binding, zinc ion binding activity and role in regulation of transcription, DNA-templated, transcription, DNA-templated |
| AN3124 | Kinesin-family protein |
| AN3127 | Predicted glycosylphosphatidylinositol (GPI)-anchored protein |
| AN3143 | Ortholog of A. nidulans FGSC A4 : AN3479, AN8495, A. fumigatus Af293 : Afu3g01440, Afu3g13940, Afu4g03340, Afu5g07620 and A. niger CBS 513.88 : An13g02880, An09g04780 |
| AN3149 | Ortholog(s) have endopeptidase activity, role in protein processing involved in protein targeting to mitochondrion and mitochondrial inner membrane peptidase complex localization |
| AN3150 | Putative gamma-glutamylcysteine synthetase with a predicted role in glutathione biosynthesis |
| AN3159 | Has domain(s) with predicted guanyl-nucleotide exchange factor activity, role in regulation of small GTPase mediated signal transduction, small GTPase mediated signal transduction and intracellular localization |
| AN3166 | Ortholog of A. fumigatus Af293 : Afu3g13410, A. oryzae RIB40 : AO090012001041, Aspergillus wentii : Aspwe1_0170055, Aspergillus sydowii : Aspsy1_0087150 and Aspergillus terreus NIH2624 : ATET_04062 |
| AN3167 | Putative ribosome biogenesis protein |
| AN3180 | Ortholog of Aspergillus versicolor : Aspve1_0051207 and Aspergillus sydowii : Aspsy1_0056131 |
| AN3207 | Ortholog of A. nidulans FGSC A4 : AN1505, A. fumigatus Af293 : Afu2g15470, Afu8g00720, Afu8g05070 and A. niger CBS 513.88 : An07g04980, An16g07680, An14g02720, An14g07130, An15g05540 |
| AN3210 | Has domain(s) with predicted substrate-specific transmembrane transporter activity, transmembrane transporter activity, transporter activity, role in transmembrane transport and integral component of membrane, membrane localization |
| AN3212 | Ortholog(s) have role in pathogenesis and cell surface, cell wall-bounded periplasmic space, extracellular region localization |
| AN3226 | Has domain protein |
| AN3228 | Predicted prenyltransferase |
| AN3231 | Has domain(s) with predicted metal ion transmembrane transporter activity, role in metal ion transport, transmembrane transport and membrane localization |
| AN3236 | Has domain(s) with predicted O-methyltransferase activity |
| AN3242 | protein of unknown function |
| AN3244 | Ortholog of A. nidulans FGSC A4 : AN5689, AN1550, A. niger CBS 513.88 : An18g00740, A. oryzae RIB40 : AO090038000176 and Aspergillus wentii : Aspwe1_0042514, Aspwe1_0046700, Aspwe1_0176395 |
| AN3248 | Has domain(s) with predicted role in oxidation-reduction process |
| AN3249 | Has domain(s) with predicted oxidoreductase activity and role in metabolic process |
| AN3256 | Putative cytochrome P450 |
| AN3265 | Arrestin domains and PY motif-containing protein with homology to Saccharomyces cerevisiae Rod1p and Rog3p proteins |
| AN3279 | Has domain(s) with predicted oxidoreductase activity and role in metabolic process |
| AN3280 | Has domain(s) with predicted RNA polymerase II transcription factor activity, sequence-specific DNA binding, zinc ion binding activity, role in regulation of transcription, DNA-templated and nucleus localization |
| AN3282 | protein of unknown function |
| AN3283 | Has domain(s) with predicted catalytic activity and role in nucleoside metabolic process |
| AN3285 | Ortholog of A. fumigatus Af293 : Afu4g01270, Neosartorya fischeri NRRL 181 : NFIA_044020, Aspergillus versicolor : Aspve1_0066429 and Aspergillus fumigatus A1163 : AFUB_101760 |
| AN3286 | Ortholog of A. nidulans FGSC A4 : AN10390, A. fumigatus Af293 : Afu4g01280, Afu5g00830, A. niger CBS 513.88 : An12g00980, An11g04090, An06g00150 and A. oryzae RIB40 : AO090138000050, AO090010000179 |
| AN3291 | Has domain(s) with predicted oxidoreductase activity and role in oxidation-reduction process |
| AN3295 | Ortholog of A. fumigatus Af293 : Afu8g00470, A. oryzae RIB40 : AO090010000638, Neosartorya fischeri NRRL 181 : NFIA_093950 and Aspergillus versicolor : Aspve1_0044578 |
| AN3297 | Protein with endo-beta-(1,4)-mannanase activity, involved in degradation of mannans |
| AN3307 | Catalytic subunit of the major alpha-1,3 glucan synthase complex |
| AN3312 | Putative L-xylulose reductase |
| AN3313 | Putative methyltransferase with a predicted role in histidine metabolism |
| AN3328 | Has domain(s) with predicted GTP binding activity |
| AN3333 | Has domain(s) with predicted oxidoreductase activity |
| AN3342 | Ortholog of A. nidulans FGSC A4 : AN10302, AN4609, A. fumigatus Af293 : Afu7g06670, A. niger CBS 513.88 : An04g04320, An16g05670, An04g03250 and A. oryzae RIB40 : AO090011000012 |
| AN3349 | Putative cytochrome P450 |
| AN3354 | Has domain(s) with predicted hydrolase activity, acting on carbon-nitrogen (but not peptide) bonds activity and role in carbohydrate metabolic process |
| AN3357 | Has domain(s) with predicted substrate-specific transmembrane transporter activity, transmembrane transporter activity, role in transmembrane transport and integral component of membrane, membrane localization |
| AN3361 | Bacterial rhodopsin family G-protein coupled receptor-like protein |
| AN3386 | Putative polyketide synthase |
| AN3393 | Protein with similarity to neutral metalloprotease II |
| AN3394 | Putative cytochrome P450 |
| AN3403 | Has domain(s) with predicted metallopeptidase activity |
| AN3404 | Predicted glycosylphosphatidylinositol (GPI)-anchored protein |
| AN3408 | Ortholog(s) have dipeptide transmembrane transporter activity, tripeptide transporter activity and role in dipeptide transmembrane transport, tripeptide transport |
| AN3409 | protein of unknown function |
| AN3425 | Ortholog(s) have cytosol localization |
| AN3432 | Aldose 1-epimerase with a predicted role in carbohydrate metabolism |
| AN3437 | Coiled-coil protein of the spindle pole body involved in distribution of nuclei in hyphae and conidiophores |
| AN3459 | Ortholog(s) have metallodipeptidase activity, omega peptidase activity, role in glutathione catabolic process and cytosol, mitochondrion, nucleus, ribosome localization |
| AN3462 | Ortholog(s) have translation release factor activity, role in mitochondrial translational termination and mitochondrial inner membrane localization |
| AN3465 | Ortholog of Aspergillus versicolor : Aspve1_0052031 |
| AN3484 | Ortholog of Aspergillus acidus : Aspfo1_0160947 |
| AN3485 | Ortholog of Aspergillus acidus : Aspfo1_0060189 |
| AN3493 | Predicted threonine-type endopeptidase |
| AN3496 | Putative nonribosomal peptide synthetase with a role in asperfuranone biosynthesis |
| AN3502 | putative transcription factor |
| AN3512 | Ortholog of Aspergillus brasiliensis : Aspbr1_0049104, Aspergillus glaucus : Aspgl1_0060098, Aspergillus flavus NRRL 3357 : AFL2T_08754 and Neosartorya fischeri NRRL 181 : NFIA_073230 |
| AN3514 | Ortholog of Aspergillus versicolor : Aspve1_0071799 and Aspergillus sydowii : Aspsy1_0045675 |
| AN3515 | Ortholog(s) have alpha-glucoside:proton symporter activity, maltose:proton symporter activity, trehalose transmembrane transporter activity |
| AN3520 | Ortholog of A. niger CBS 513.88 : An04g10140, Aspergillus brasiliensis : Aspbr1_0153268, Aspergillus kawachii : Aspka1_0181306 and Aspergillus acidus : Aspfo1_0032523 |
| AN3525 | protein of unknown function |
| AN3528 | Ortholog of A. nidulans FGSC A4 : AN7149, A. fumigatus Af293 : Afu4g03620, Afu6g02580, A. niger CBS 513.88 : An14g01630, An04g09610 and A. oryzae RIB40 : AO090113000188, AO090011000275, AO090023000922 |
| AN3545 | Has domain(s) with predicted oxidoreductase activity and role in metabolic process |
| AN3550 | Ortholog of A. oryzae RIB40 : AO090102000426, AO090138000144, Aspergillus wentii : Aspwe1_0173124, Aspergillus fumigatus A1163 : AFUB_044240 and Aspergillus zonatus : Aspzo1_0105859 |
| AN3559 | Ortholog of Aspergillus brasiliensis : Aspbr1_0209810, Aspergillus wentii : Aspwe1_0174914, Aspergillus versicolor : Aspve1_0043449 and Aspergillus sydowii : Aspsy1_0176892 |
| AN3570 | Has domain(s) with predicted oxidoreductase activity and role in oxidation-reduction process |
| AN3573 | Has domain(s) with predicted oxidoreductase activity, oxidoreductase activity, acting on the aldehyde or oxo group of donors, NAD or NADP as acceptor activity and role in oxidation-reduction process |
| AN3585 | Transcript induced in response to calcium dichloride in a CrzA-dependent manner |
| AN3586 | Ortholog(s) have mitochondrion localization |
| AN3590 | Ortholog of A. nidulans FGSC A4 : AN7906, A. fumigatus Af293 : Afu4g12880, A. niger CBS 513.88 : An01g08440, A. oryzae RIB40 : AO090009000315 and Aspergillus wentii : Aspwe1_0033843 |
| AN3592 | Putative calnexin with a predicted role in protein folding and protein quality control on the endoplasmic reticulum (ER) membrane |
| AN3593 | Ortholog(s) have methylthioribulose 1-phosphate dehydratase activity, role in L-methionine biosynthetic process from methylthioadenosine and cytosol, nucleus localization |
| AN3597 | Protein involved in carbon source utilization |
| AN3606 | Ortholog of A. fumigatus Af293 : Afu4g12700, A. niger CBS 513.88 : An03g02800, A. oryzae RIB40 : AO090009000298, Aspergillus wentii : Aspwe1_0047287 and Aspergillus sydowii : Aspsy1_0090817 |
| AN3613 | Protein with endo-1,4-beta-xylanase activity, involved in degradation of xylans |
| AN3619 | Protein with similarity to Rad50 |
| AN3625 | Ortholog of A. fumigatus Af293 : Afu4g12610, A. niger CBS 513.88 : An01g08100, A. oryzae RIB40 : AO090009000332, Aspergillus wentii : Aspwe1_0216634 and Aspergillus sydowii : Aspsy1_0046675 |
| AN3626 | Putative phosphoribosylamino-imidazole-carboxylase with a predicted role in purine metabolism |
| AN3629 | Ortholog(s) have oxidized purine nucleobase lesion DNA N-glycosylase activity |
| AN3630 | Ortholog of A. fumigatus Af293 : Afu5g01370, Neosartorya fischeri NRRL 181 : NFIA_040730, Aspergillus versicolor : Aspve1_0032027 and Aspergillus fumigatus A1163 : AFUB_049880 |
| AN3642 | Ortholog(s) have role in cellular response to biotic stimulus, cellular response to starvation and filamentous growth of a population of unicellular organisms in response to biotic stimulus, more |
| AN3643 | Ortholog(s) have guanyl-nucleotide exchange factor activity and role in Golgi to endosome transport, endocytosis, protein targeting to vacuole |
| AN3645 | Ortholog of A. fumigatus Af293 : Afu4g12090, Aspergillus wentii : Aspwe1_0102093, Aspergillus sydowii : Aspsy1_0154146, Aspergillus terreus NIH2624 : ATET_03856 and Aspergillus glaucus : Aspgl1_0068126 |
| AN3655 | Ortholog of A. fumigatus Af293 : Afu4g12220, A. niger CBS 513.88 : An01g07500, A. oryzae RIB40 : AO090009000424, Aspergillus wentii : Aspwe1_0166578 and Aspergillus sydowii : Aspsy1_0204923 |
| AN3656 | Putative nitrilase with a predicted role in nitrogen or cyanoamino acid metabolism |
| AN3657 | Ortholog(s) have 3'-5'-exoribonuclease activity, Ran GTPase binding, endoribonuclease activity, tRNA binding activity |
| AN3658 | Has domain(s) with predicted role in transmembrane transport and integral component of membrane localization |
| AN3659 | Predicted scaffold protein with similarity to mammalian paxillin |
| AN3665 | Ortholog(s) have Golgi apparatus, endoplasmic reticulum, fungal-type vacuole membrane localization |
| AN3670 | Ortholog of A. fumigatus Af293 : Afu4g12380, A. niger CBS 513.88 : An01g07790, A. oryzae RIB40 : AO090009000446, Aspergillus wentii : Aspwe1_0166560 and Aspergillus sydowii : Aspsy1_0032623 |
| AN3675 | Transcription factor of the Gcn4p c-Jun-like transcriptional activator family |
| AN3681 | Ortholog(s) have role in cellular iron ion homeostasis and fungal-type vacuole membrane localization |
| AN3690 | Ortholog(s) have iron ion transmembrane transporter activity, role in RNA splicing, iron ion homeostasis, mitochondrial iron ion transport and mitochondrion, plasma membrane localization |
| AN3692 | Ortholog(s) have COPI-coated vesicle, Golgi apparatus localization |
| AN3693 | Ortholog(s) have Rpd3L-Expanded complex, Set3 complex, cytosol localization |
| AN3696 | Ortholog(s) have role in actin cortical patch assembly, actin filament organization, cellular response to drug, endocytosis and mating projection tip localization |
| AN3710 | Ortholog(s) have role in attachment of mitotic spindle microtubules to kinetochore and DASH complex, cytosol, mitotic spindle polar microtubule, mitotic spindle pole body localization |
| AN3716 | Ortholog of A. fumigatus Af293 : Afu6g12770, A. niger CBS 513.88 : An08g10710, A. oryzae RIB40 : AO090003000397, Aspergillus wentii : Aspwe1_0102826 and Aspergillus sydowii : Aspsy1_0032599 |
| AN3722 | Ortholog(s) have role in secondary metabolite biosynthetic process |
| AN3727 | Putative 1,3-beta-transglucosylase with a predicted role in glucan processing |
| AN3731 | Ortholog of A. fumigatus Af293 : Afu6g12420, A. niger CBS 513.88 : An06g01580, A. oryzae RIB40 : AO090009000171, Aspergillus wentii : Aspwe1_0099460 and Aspergillus sydowii : Aspsy1_0044130 |
| AN3734 | Possible pseudogene, similar to autophagy-related protein |
| AN3741 | Alcohol dehydrogenase II, has a predicted role in two-carbon compound metabolism |
| AN3745 | Ribosome assembly factor |
| AN3748 | Putative ATP phosphoribosyltransferase with a predicted role in histidine metabolism |
| AN3752 | Has domain(s) with predicted intracellular localization |
| AN3765 | Putative G-protein coupled receptor |
| AN3769 | Has domain(s) with predicted DNA binding, RNA polymerase II transcription factor activity, sequence-specific DNA binding, zinc ion binding activity and role in regulation of transcription, DNA-templated, transcription, DNA-templated |
| AN3770 | Ortholog of A. niger CBS 513.88 : An11g08200, A. oryzae RIB40 : AO090701000165, Aspergillus wentii : Aspwe1_0185750, Aspergillus versicolor : Aspve1_0053166 and Aspergillus niger ATCC 1015 : 178171-mRNA |
| AN3771 | Ortholog(s) have cytoplasm localization |
| AN3781 | Has domain(s) with predicted inorganic phosphate transmembrane transporter activity, role in phosphate ion transport and membrane localization |
| AN3782 | Has domain(s) with predicted FAD binding, oleate hydratase activity and role in fatty acid metabolic process |
| AN3785 | Ortholog(s) have role in heterochromatin maintenance, homologous recombination-dependent replication fork processing, nucleosome assembly and CAF-1 complex, cytosol, nuclear replication fork localization |
| AN3829 | Putative succinate-semialdehyde dehydrogenase [NAD(P)+] with a predicted role in 4-aminobutyrate (GABA) shunt |
| AN3834 | Ortholog of A. fumigatus Af293 : Afu4g08090, A. niger CBS 513.88 : An04g02530, A. oryzae RIB40 : AO090023000760, Aspergillus wentii : Aspwe1_0064360 and Aspergillus sydowii : Aspsy1_0057405 |
| AN3838 | Ortholog(s) have cytoplasm localization |
| AN3854 | Has domain(s) with predicted syntaxin binding activity |
| AN3856 | protein of unknown function |
| AN3862 | Has domain(s) with predicted heme binding, oxidoreductase activity and role in oxidation-reduction process |
| AN3863 | Has domain(s) with predicted DNA binding, RNA polymerase II transcription factor activity, sequence-specific DNA binding, zinc ion binding activity and role in regulation of transcription, DNA-templated, transcription, DNA-templated |
| AN3865 | Ortholog(s) have mitochondrion localization |
| AN3871 | Ortholog of A. niger CBS 513.88 : An04g04400, A. oryzae RIB40 : AO090001000062, Neosartorya fischeri NRRL 181 : NFIA_045750 and Aspergillus wentii : Aspwe1_0024862, Aspwe1_0046487 |
| AN3872 | Transcript induced by light in in developmentally competent mycelia |
| AN3873 | Putative zinc-binding oxidoreductase |
| AN3874 | Putative alpha-1,4-galactosidase |
| AN3876 | Has domain(s) with predicted role in transmembrane transport and membrane localization |
| AN3890 | Ortholog(s) have role in mismatch repair, nucleotide-excision repair and mitotic spindle, nucleus localization |
| AN3900 | Has domain(s) with predicted DNA binding, RNA polymerase II transcription factor activity, sequence-specific DNA binding, zinc ion binding activity and role in regulation of transcription, DNA-templated, transcription, DNA-templated |
| AN3901 | Putative lactic acid dehydrogenase with a predicted role in energy metabolism |
| AN3902 | Has domain(s) with predicted UDP-N-acetylmuramate dehydrogenase activity, flavin adenine dinucleotide binding, oxidoreductase activity, oxidoreductase activity, acting on CH-OH group of donors activity |
| AN3924 | Has domain(s) with predicted nucleus localization |
| AN3928 | Putative thiazole synthase, enzyme of the thiamine biosynthesis pathway |
| AN3937 | Ortholog(s) have nucleus localization |
| AN3938 | Ortholog(s) have nucleolus localization |
| AN3970 | Kinesin-family protein |
| AN3973 | Putative peroxiredoxin |
| AN3977 | Has domain(s) with predicted role in cell wall macromolecule catabolic process |
| AN3994 | Ortholog of A. nidulans FGSC A4 : AN5182, AN2782, A. fumigatus Af293 : Afu1g01650, Afu2g00470, Afu6g07130, Afu7g00770, A. niger CBS 513.88 : An15g02620 and A. oryzae RIB40 : AO090005000527, AO090012000936 |
| AN3996 | Has domain(s) with predicted methyltransferase activity and role in metabolic process |
| AN4000 | Protein with similarity to poly(A)-binding proteins |
| AN4005 | Ortholog of A. fumigatus Af293 : Afu3g05800 and Aspergillus fumigatus A1163 : AFUB_043230 |
| AN4013 | Has domain(s) with predicted DNA binding, nucleic acid binding, zinc ion binding activity, role in transcription, DNA-templated and nucleus localization |
| AN4015 | Ortholog(s) have ribosome binding, translation elongation factor activity, translation initiation factor activity |
| AN4018 | Ortholog(s) have role in protein targeting to vacuole |
| AN4022 | Ortholog of A. fumigatus Af293 : Afu1g03700, A. oryzae RIB40 : AO090003000976, Aspergillus wentii : Aspwe1_0103267, Aspergillus sydowii : Aspsy1_0057572 and Aspergillus terreus NIH2624 : ATET_03840 |
| AN4026 | Ortholog of A. fumigatus Af293 : Afu1g03650, A. niger CBS 513.88 : An18g04060, A. oryzae RIB40 : AO090003000980, Aspergillus wentii : Aspwe1_0169329 and Aspergillus sydowii : Aspsy1_0149715 |
| AN4028 | Ortholog(s) have Golgi apparatus localization |
| AN4029 | Protein expressed at increased levels during osmoadaptation |
| AN4038 | Putative translation initiation factor eIF5B |
| AN4045 | Ortholog of A. fumigatus Af293 : Afu1g03910, A. niger CBS 513.88 : An01g02700, A. oryzae RIB40 : AO090003000959, Aspergillus wentii : Aspwe1_0169355 and Aspergillus sydowii : Aspsy1_0148783 |
| AN4052 | Putative glucan 1,3-beta-glucosidase with a predicted role in glucan metabolism |
| AN4069 | Protein with similarity to human parvulin |
| AN4071 | Ortholog(s) have role in dolichol biosynthetic process, dolichol-linked oligosaccharide biosynthetic process, polyprenol catabolic process, pseudohyphal growth |
| AN4072 | Ortholog of A. fumigatus Af293 : Afu1g05490, A. niger CBS 513.88 : An18g04300, A. oryzae RIB40 : AO090009000396, Aspergillus wentii : Aspwe1_0169302 and Aspergillus sydowii : Aspsy1_0057624 |
| AN4075 | protein of unknown function |
| AN4079 | protein of unknown function |
| AN4088 | Ortholog of A. fumigatus Af293 : Afu1g05680, A. oryzae RIB40 : AO090009000366, Neosartorya fischeri NRRL 181 : NFIA_019060, Aspergillus wentii : Aspwe1_0169283 and Aspergillus versicolor : Aspve1_0085484 |
| AN4089 | Ortholog of A. fumigatus Af293 : Afu1g05690, A. niger CBS 513.88 : An18g03450, A. oryzae RIB40 : AO090009000365, Aspergillus wentii : Aspwe1_0104096 and Aspergillus sydowii : Aspsy1_0057643 |
| AN4091 | Has domain(s) with predicted ATP binding, ATPase activity, ATPase activity, coupled to transmembrane movement of substances, nucleoside-triphosphatase activity, nucleotide binding activity and role in transmembrane transport |
| AN4092 | Ortholog of A. nidulans FGSC A4 : AN7483, A. fumigatus Af293 : Afu2g05710, A. oryzae RIB40 : AO090001000688, Neosartorya fischeri NRRL 181 : NFIA_082430 and Aspergillus versicolor : Aspve1_0043503 |
| AN4096 | Ortholog(s) have role in conidiophore development, hyphal growth and cytoplasm, nucleus localization |
| AN4097 | Has domain(s) with predicted role in transmembrane transport and integral component of membrane localization |
| AN4101 | Ortholog(s) have antimonite transmembrane transporter activity, antiporter activity, arsenite transmembrane transporter activity, role in antimonite transport, arsenite transport and plasma membrane localization |
| AN4102 | Putative beta-glucosidase |
| AN4104 | Ortholog of A. fumigatus Af293 : Afu1g05860, A. niger CBS 513.88 : An18g03590, A. oryzae RIB40 : AO090009000354, Aspergillus wentii : Aspwe1_0026045 and Aspergillus sydowii : Aspsy1_0089091 |
| AN4105 | Putative erythromycin esterase family protein |
| AN4118 | Ortholog(s) have sequence-specific DNA binding, transcription factor activity, sequence-specific DNA binding activity |
| AN4121 | protein of unknown function |
| AN4122 | Ortholog of A. nidulans FGSC A4 : AN2881, AN10123, AN0867, A. fumigatus Af293 : Afu1g13860, Afu1g15180, Afu3g11650 and A. niger CBS 513.88 : An08g07010, An02g07440, An01g13480 |
| AN4123 | Has domain(s) with predicted RNA polymerase II transcription factor activity, sequence-specific DNA binding, zinc ion binding activity, role in regulation of transcription, DNA-templated and nucleus localization |
| AN4125 | Ortholog of Aspergillus brasiliensis : Aspbr1_0028583, Aspergillus glaucus : Aspgl1_0050631, Aspergillus versicolor : Aspve1_0040328, Aspve1_0732669 and Aspergillus sydowii : Aspsy1_0029968, Aspsy1_0033388 |
| AN4126 | Putative dehydrogenase with a predicted role in metabolism or penicillin biosynthesis |
| AN4150 | Ortholog(s) have biotin transmembrane transporter activity, dethiobiotin transmembrane transporter activity, solute:proton symporter activity |
| AN4151 | Ortholog of A. nidulans FGSC A4 : AN5421, A. fumigatus Af293 : Afu5g07490, A. niger CBS 513.88 : An14g01660, An08g09760, An11g00210 and A. oryzae RIB40 : AO090012000034, AO090009000581, AO090012000374 |
| AN4159 | Putative glutamate-ammonia ligase with a predicted role in glutamate and glutamine metabolism |
| AN4160 | Has domain(s) with predicted RNA polymerase II transcription cofactor activity, role in regulation of transcription from RNA polymerase II promoter and mediator complex localization |
| AN4163 | Protein with seven WD repeats, involved in cross-pathway control of the response to amino acid starvation |
| AN4172 | Ortholog of A. fumigatus Af293 : Afu6g07930, A. niger CBS 513.88 : An11g02850, A. oryzae RIB40 : AO090003000141, Aspergillus wentii : Aspwe1_0748330 and Aspergillus sydowii : Aspsy1_0059114 |
| AN4176 | Ortholog of A. niger CBS 513.88 : An15g01870, An19g00110, An12g05110, A. oryzae RIB40 : AO090009000510 and Aspergillus wentii : Aspwe1_0033361, Aspwe1_0034402 |
| AN4178 | UPF0160 domain-containing protein |
| AN4182 | Cyclin-dependent protein kinase involved in cell cycle control |
| AN4197 | Ortholog of Aspergillus versicolor : Aspve1_0138462 and Aspergillus sydowii : Aspsy1_0154012 |
| AN4206 | Ortholog of A. fumigatus Af293 : Afu1g06020, A. niger CBS 513.88 : An18g03870, Aspergillus wentii : Aspwe1_0026071, Aspergillus sydowii : Aspsy1_0059171 and Aspergillus terreus NIH2624 : ATET_03025 |
| AN4207 | Ortholog(s) have role in endosomal transport, vesicle-mediated transport and AP-1 adaptor complex, endosome localization |
| AN4212 | Putative aryl-alcohol oxidase-related protein |
| AN4213 | protein of unknown function |
| AN4214 | Cell-end marker protein kinase |
| AN4216 | Ortholog(s) have mitochondrion localization |
| AN4226 | Ortholog(s) have mRNA binding activity, role in maturation of SSU-rRNA from tricistronic rRNA transcript (SSU-rRNA, 5.8S rRNA, LSU-rRNA) and 90S preribosome, mitotic spindle, nucleolus, small-subunit processome localization |
| AN4230 | Has domain(s) with predicted catalytic activity and role in metabolic process |
| AN4239 | Ortholog(s) have RSC complex localization |
| AN4241 | Has domain(s) with predicted role in transmembrane transport and integral component of membrane localization |
| AN4243 | Has domain(s) with predicted 3-beta-hydroxy-delta5-steroid dehydrogenase activity and role in oxidation-reduction process, steroid biosynthetic process |
| AN4247 | Has domain(s) with predicted RNA polymerase II transcription factor activity, sequence-specific DNA binding, zinc ion binding activity, role in regulation of transcription, DNA-templated and nucleus localization |
| AN4251 | Ortholog(s) have structural constituent of ribosome activity and mitochondrial small ribosomal subunit localization |
| AN4260 | Predicted glycosylphosphatidylinositol (GPI)-anchored protein |
| AN4269 | Ortholog(s) have DNA binding, RNA polymerase II activity, RNA-directed RNA polymerase activity, role in termination of RNA polymerase II transcription and DNA-directed RNA polymerase II, core complex, cytosol localization |
| AN4270 | Ortholog(s) have protein binding, bridging activity and role in actin cortical patch assembly, axial cellular bud site selection, bipolar cellular bud site selection, endocytosis |
| AN4280 | Putative mitochondrial carrier protein |
| AN4284 | Ortholog of A. fumigatus Af293 : Afu5g04336, A. niger CBS 513.88 : An09g06820, Neosartorya fischeri NRRL 181 : NFIA_037740 and Aspergillus versicolor : Aspve1_0047298 |
| AN4294 | Ortholog(s) have mitochondrion localization |
| AN4311 | Ortholog of A. fumigatus Af293 : Afu4g06030, A. niger CBS 513.88 : An04g00250, A. oryzae RIB40 : AO090023000993, Aspergillus wentii : Aspwe1_0167289 and Aspergillus sydowii : Aspsy1_0051121 |
| AN4312 | Ortholog of A. fumigatus Af293 : Afu4g06040, A. niger CBS 513.88 : An04g00270, A. oryzae RIB40 : AO090023000991, Aspergillus wentii : Aspwe1_0101375 and Aspergillus sydowii : Aspsy1_0036593 |
| AN4313 | Ortholog(s) have drug transmembrane transporter activity, spermidine transmembrane transporter activity, spermine transmembrane transporter activity |
| AN4322 | Ortholog(s) have clathrin-coated vesicle, cytoplasm localization |
| AN4323 | Putative branched chain amino acid aminotransferase with a predicted role in valine, leucine, and isoleucine metabolism |
| AN4352 | Ortholog(s) have mitochondrion, plasma membrane localization |
| AN4353 | Ortholog(s) have glyoxysome localization |
| AN4355 | Ortholog(s) have cytosol localization |
| AN4359 | Has domain(s) with predicted zinc ion binding activity |
| AN4363 | Has domain(s) with predicted UDP-N-acetylmuramate dehydrogenase activity, flavin adenine dinucleotide binding activity and role in oxidation-reduction process |
| AN4375 | Ortholog of A. nidulans FGSC A4 : AN2913, A. fumigatus Af293 : Afu3g07740, Afu4g06610, A. niger CBS 513.88 : An04g00930 and A. oryzae RIB40 : AO090023000927, AO090020000420 |
| AN4376 | Putative NADP-linked glutamate dehydrogenase |
| AN4377 | Has domain(s) with predicted RNA polymerase II transcription cofactor activity, role in regulation of transcription from RNA polymerase II promoter and mediator complex localization |
| AN4381 | Covalently-bound cell wall protein |
| AN4389 | Putative CMP glycosylase with a predicted role in nucleotide salvage pathways |
| AN4392 | protein of unknown function |
| AN4397 | Ortholog of S. cerevisiae Pcs60p |
| AN4406 | Has domain(s) with predicted structural molecule activity |
| AN4407 | Putative protein with a predicted role in homologous recombination and DNA damage repair |
| AN4409 | Ornithine carbamoyltransferase of the arginine biosynthesis pathway |
| AN4411 | Ortholog(s) have polyubiquitin binding, sterol binding activity and role in anaphase-promoting complex-dependent proteasomal ubiquitin-dependent protein catabolic process, mitotic sister chromatid segregation, proteasome localization |
| AN4412 | Putative nuclear exosomal DEAD-box family RNA helicase |
| AN4419 | Ortholog(s) have protein tyrosine phosphatase activity |
| AN4422 | Putative aspartic-type endopeptidase |
| AN4438 | Ortholog of A. nidulans FGSC A4 : AN4246, AN8484, A. fumigatus Af293 : Afu1g06480, Afu2g05150/mp2, A. niger CBS 513.88 : An18g05510 and A. oryzae RIB40 : AO090001000450 |
| AN4439 | Ortholog(s) have ATPase activity, nicotinamide-nucleotide adenylyltransferase activity |
| AN4441 | Ortholog(s) have endoplasmic reticulum localization |
| AN4443 | Putative methionine synthase with a predicted role in methionine metabolism |
| AN4460 | Putative 18S rRNA processing complex subunit |
| AN4460-uORF | Conserved upstream open reading frame (uORF) of AN4460 |
| AN4464 | Putative bifunctional enzyme with a predicted role in purine metabolism |
| AN4471 | Ortholog(s) have role in maturation of 5.8S rRNA from tricistronic rRNA transcript (SSU-rRNA, 5.8S rRNA, LSU-rRNA), maturation of LSU-rRNA from tricistronic rRNA transcript (SSU-rRNA, 5.8S rRNA, LSU-rRNA), ribosomal large subunit assembly |
| AN4473 | Ortholog of A. nidulans FGSC A4 : AN5608, A. fumigatus Af293 : Afu4g07720, A. niger CBS 513.88 : An14g07030, An04g02100 and A. oryzae RIB40 : AO090023000800, AO090009000070 |
| AN4494 | Ortholog(s) have cytosolic large ribosomal subunit, hyphal cell wall, mitochondrion, nucleus localization |
| AN4515 | Putative transglycosidase with a predicted role in glucan processing |
| AN4517 | Ortholog(s) have glutathione transmembrane transporter activity, phytochelatin transmembrane transporter ATPase activity |
| AN4524 | Has domain(s) with predicted DNA binding, chromatin binding activity |
| AN4538 | Ortholog of A. fumigatus Af293 : Afu2g02830, A. niger CBS 513.88 : An07g07180, A. oryzae RIB40 : AO090026000628, Aspergillus wentii : Aspwe1_0036706 and Aspergillus sydowii : Aspsy1_0091097 |
| AN4540 | Has domain(s) with predicted oxidoreductase activity, zinc ion binding activity and role in oxidation-reduction process |
| AN4548 | Ortholog(s) have mitochondrion localization |
| AN4550 | Putative asparaginyl-tRNA synthetase with a predicted role in tRNA charging for translation |
| AN4552 | Ortholog of Ku80 |
| AN4554 | protein of unknown function |
| AN4555 | Ortholog(s) have role in maintenance of rDNA, mitotic DNA replication, mitotic sister chromatid segregation, postreplication repair and RecQ helicase-Topo III complex, mitochondrion, nucleus, site of double-strand break localization |
| AN4559 | Ortholog(s) have mitochondrion localization |
| AN4561 | Phosducin-like protein |
| AN4567 | Ortholog of Aspergillus versicolor : Aspve1_0053822, Aspergillus sydowii : Aspsy1_0154895 and Aspergillus aculeatus ATCC16872 : Aacu16872_040769 |
| AN4575 | Ortholog of A. oryzae RIB40 : AO090011000461, Aspergillus flavus NRRL 3357 : AFL2T_05228, Aspergillus versicolor : Aspve1_0042983 and Aspergillus sydowii : Aspsy1_0091136 |
| AN4577 | Putative kynurenine formamidase with a predicted role in aromatic amino acid biosynthesis |
| AN4579 | Has domain(s) with predicted role in peroxisome inheritance and extrinsic component of intraperoxisomal membrane localization |
| AN4582 | Ortholog of A. fumigatus Af293 : Afu2g01970, A. niger CBS 513.88 : An07g05780, A. oryzae RIB40 : AO090011000467, Aspergillus wentii : Aspwe1_0029040 and Aspergillus sydowii : Aspsy1_1095743 |
| AN4586 | Has domain(s) with predicted nucleic acid binding, zinc ion binding activity |
| AN4587 | Ortholog(s) have methylated-DNA-[protein]-cysteine S-methyltransferase activity and role in DNA dealkylation involved in DNA repair |
| AN4589 | Putative translocon, beta subunit |
| AN4590 | Sugar transporter |
| AN4603 | Putative allantoinase with a predicted role in purine metabolism |
| AN4614 | Ortholog(s) have role in cellular response to calcium ion and RAVE complex, cytosol, nucleus localization |
| AN4615 | Transmembrane domain-containing protein with homology to S. cerevisiae Sur7p |
| AN4628 | protein of unknown function |
| AN4642 | Ortholog of A. nidulans FGSC A4 : AN6924, AN5943, AN8512, AN8548, AN0857, AN8661 and A. fumigatus Af293 : Afu1g15290, Afu3g00850, Afu4g08850, Afu7g00920 |
| AN4646 | Ortholog of A. niger CBS 513.88 : An07g05420, Aspergillus versicolor : Aspve1_0042823, Aspergillus niger ATCC 1015 : 39924-mRNA and Aspergillus sydowii : Aspsy1_0155641 |
| AN4652 | Ortholog(s) have structural constituent of ribosome activity and mitochondrial large ribosomal subunit, mitochondrial membrane localization |
| AN4656 | Possible pseudogene |
| AN4659 | Putative acyl-CoA synthetase/AMP-binding domain protein |
| AN4660 | protein of unknown function |
| AN4662 | Ortholog of A. fumigatus Af293 : Afu5g08490, Aspergillus wentii : Aspwe1_0048145, Aspergillus sydowii : Aspsy1_0155692 and Aspergillus terreus NIH2624 : ATET_05768 |
| AN4664 | Dynein light intermediate chain |
| AN4668 | Putative mitogen activated protein kinase (MAPK) |
| AN4669 | Ortholog(s) have role in chromatin silencing at centromere, chromatin silencing by small RNA and cytosol, nuclear RNA-directed RNA polymerase complex localization |
| AN4670 | Subunit of the SAGA transcriptional regulatory complex |
| AN4670-uORF | Conserved upstream open reading frame (uORF) of AN4670 |
| AN4674 | Ortholog(s) have role in asexual sporulation resulting in formation of a cellular spore, cellular response to drug and cell septum, cell surface localization |
| AN4677 | protein of unknown function |
| AN4697 | Ortholog(s) have palmitoyltransferase activity, role in protein palmitoylation, vacuole fusion, non-autophagic and Golgi apparatus, fungal-type vacuole membrane localization |
| AN4699 | Ortholog of A. niger CBS 513.88 : An07g04660, A. oryzae RIB40 : AO090020000435, Aspergillus wentii : Aspwe1_0278331, Aspergillus versicolor : Aspve1_0053730 and Aspergillus clavatus NRRL 1 : ACLA_011760 |
| AN4700 | Protein with endo-beta-(1,3)-glucanase activity, involved in degradation of glucans |
| AN4716 | Ortholog(s) have alpha-1,6-mannosyltransferase activity |
| AN4727 | UDP-glucose 4-epimerase, involved in galactose metabolism |
| AN4728 | Ortholog(s) have endoplasmic reticulum localization |
| AN4732 | Ortholog of A. fumigatus Af293 : Afu5g10730, A. niger CBS 513.88 : An14g03950, A. oryzae RIB40 : AO090010000468, Aspergillus wentii : Aspwe1_0439775 and Aspergillus terreus NIH2624 : ATET_07636 |
| AN4739 | Putative phosphoribosyl amino imidazolesuccinocarbozamide synthetase with a predicted role in purine metabolism |
| AN4747 | Has domain(s) with predicted role in cellular response to oxidative stress and endoplasmic reticulum membrane localization |
| AN4751 | Has domain(s) with predicted ATP binding, ATP-dependent helicase activity, nucleic acid binding activity |
| AN4760 | Ortholog(s) have U2 snRNP localization |
| AN4761 | Subfamily 1 protein O-mannosyltransferase |
| AN4762 | Ortholog(s) have mitochondrion localization |
| AN4768 | Ortholog of A. fumigatus Af293 : Afu3g06520, A. niger CBS 513.88 : An11g09800, A. oryzae RIB40 : AO090020000350, Aspergillus wentii : Aspwe1_0029356 and Aspergillus sydowii : Aspsy1_0043240 |
| AN4773 | Has domain(s) with predicted DNA binding, zinc ion binding activity, role in transcription, DNA-templated and nucleus localization |
| AN4785 | Ortholog(s) have RNA polymerase II core promoter proximal region sequence-specific DNA binding and RNA polymerase II transcription factor activity, more |
| AN4801 | protein of unknown function |
| AN4814 | Ortholog(s) have role in cellular calcium ion homeostasis and Golgi medial cisterna, cis-Golgi network, endoplasmic reticulum, fungal-type vacuole membrane localization |
| AN4818 | Putative histidine-containing phosphotransfer protein |
| AN4820 | Putative succinate-semialdehyde dehydrogenase [NAD(P)+] with a predicted role in 4-aminobutyrate (GABA) shunt |
| AN4821 | Has domain(s) with predicted DNA binding, zinc ion binding activity, role in transcription, DNA-templated and nucleus localization |
| AN4834 | Ortholog of A. fumigatus Af293 : Afu3g07270, A. niger CBS 513.88 : An02g13430, A. oryzae RIB40 : AO090020000197, Aspergillus wentii : Aspwe1_0052406 and Aspergillus sydowii : Aspsy1_0087772 |
| AN4836 | Ortholog(s) have Golgi apparatus localization |
| AN4842 | Ortholog(s) have copper ion binding activity, role in copper ion transport, protein complex assembly and mitochondrial inner membrane, plasma membrane localization |
| AN4843 | Putative alpha-glucosidase with a predicted role in maltose metabolism |
| AN4850 | Ortholog(s) have nucleus localization |
| AN4853 | Putative membrane protein involved in a signaling pathway that activates PacC transcription factor in response to alkaline ambient pH |
| AN4857 | Has domain(s) with predicted transferase activity, transferring phosphorus-containing groups activity |
| AN4859 | Plasma membrane ATPase with a predicted role in energy metabolism |
| AN4861 | Ortholog(s) have RNA polymerase II core binding, RNA polymerase II regulatory region DNA binding, transcription factor activity, core RNA polymerase II recruiting activity |
| AN4862 | Putative Ran GTPase activating protein (GAP) with homology to Saccharomyces cerevisiae Rna1p |
| AN4863 | Ortholog(s) have copper chaperone activity, role in intracellular copper ion transport, mitochondrial respiratory chain complex IV assembly and cytosol, mitochondrial intermembrane space, nucleus localization |
| AN4880 | Ortholog(s) have cell division site, cytosol, nucleus localization |
| AN4886 | Ortholog(s) have role in CVT pathway, ER to Golgi vesicle-mediated transport, pexophagy, retrograde transport, vesicle recycling within Golgi and Golgi transport complex, cell division site localization |
| AN4892 | Ortholog(s) have nucleus localization |
| AN4906 | Ortholog(s) have endoplasmic reticulum localization |
| AN4907 | Ortholog of A. fumigatus Af293 : Afu3g10810, A. niger CBS 513.88 : An03g02970, Aspergillus wentii : Aspwe1_0362245, Aspergillus sydowii : Aspsy1_0043016 and Aspergillus terreus NIH2624 : ATET_04610 |
| AN4917 | p62 subunit of dynactin, component of the dynactin complex involved in nuclear distribution |
| AN4918 | Ortholog(s) have intracellular localization |
| AN4920 | Putative calcium-transporting mitochondrial ATPase involved in calcium homeostasis |
| AN4923 | Putative 3-hydroxy-3-methylglutaryl coenzyme A synthase with a predicted role in sterol metabolism |
| AN4927 | Putative F1F0-ATPase complex subunit with a predicted role in energy metabolism |
| AN4939 | Ortholog(s) have enzyme activator activity, heme binding activity, role in ergosterol biosynthetic process, regulation of ergosterol biosynthetic process and endoplasmic reticulum, endosome localization |
| AN4959 | Has domain(s) with predicted role in DNA replication and nucleus localization |
| AN4973 | Ortholog of A. fumigatus Af293 : Afu3g10150, A. niger CBS 513.88 : An16g03980, A. oryzae RIB40 : AO090003000550, Aspergillus wentii : Aspwe1_0049197 and Aspergillus sydowii : Aspsy1_0056460 |
| AN4979 | Predicted dihydroneopterin aldolase |
| AN4980 | Ortholog(s) have cytosol localization |
| AN4986 | Has domain(s) with predicted L-ascorbic acid binding, iron ion binding, oxidoreductase activity, acting on paired donors, with incorporation or reduction of molecular oxygen and oxidoreductase activity, more |
| AN4989 | Ortholog of A. fumigatus Af293 : Afu3g09980, A. niger CBS 513.88 : An16g03710, Aspergillus sydowii : Aspsy1_0042878 and Aspergillus terreus NIH2624 : ATET_04521 |
| AN4993 | Putative GNAT-type acetyltransferase |
| AN4995 | Has domain(s) with predicted integral component of membrane localization |
| AN5005 | Ortholog of A. fumigatus Af293 : Afu3g09840, A. niger CBS 513.88 : An16g03510, A. oryzae RIB40 : AO090005001262, Aspergillus wentii : Aspwe1_0036826 and Aspergillus sydowii : Aspsy1_0042841 |
| AN5017 | Has domain(s) with predicted oxidoreductase activity and role in metabolic process |
| AN5020 | Putative ADP ribosylation factor (Arf) family protein |
| AN5021 | Ortholog(s) have role in conidium formation, regulation of growth rate |
| AN5030 | Has domain(s) with predicted NAD binding, oxidoreductase activity, acting on the CH-OH group of donors, NAD or NADP as acceptor activity and role in oxidation-reduction process |
| AN5033 | Predicted glycosylphosphatidylinositol (GPI)-anchored protein |
| AN5035 | Has domain(s) with predicted solute:proton antiporter activity, role in cation transport, transmembrane transport and integral component of membrane localization |
| AN5037 | Has domain(s) with predicted flavin adenine dinucleotide binding, oxidoreductase activity, acting on CH-OH group of donors activity and role in oxidation-reduction process |
| AN5072 | protein of unknown function |
| AN5074 | protein of unknown function |
| AN5076 | Has domain(s) with predicted role in cell wall macromolecule catabolic process |
| AN5087 | Ortholog of Aspergillus tubingensis : Asptu1_0123225, Aspergillus brasiliensis : Aspbr1_0049601 and Aspergillus fumigatus A1163 : AFUB_080030 |
| AN5111 | Ortholog(s) have cytosol, nucleolus localization |
| AN5112 | Has domain(s) with predicted single-stranded DNA endodeoxyribonuclease activity and role in DNA damage checkpoint, DNA repair |
| AN5120 | protein of unknown function |
| AN5123 | Has domain(s) with predicted oxidoreductase activity |
| AN5134 | Glutamate synthase, NAD(+)-dependent (GOGAT) with a predicted role in glutamate and glutamine metabolism |
| AN5148 | Ortholog(s) have structural constituent of ribosome activity, role in cellular response to drug and mitochondrial small ribosomal subunit localization |
| AN5149 | Ortholog of A. fumigatus Af293 : Afu6g07200, A. niger CBS 513.88 : An07g09260, A. oryzae RIB40 : AO090005001581, Aspergillus wentii : Aspwe1_0112093 and Aspergillus sydowii : Aspsy1_0059680 |
| AN5155 | Ortholog(s) have tRNA-specific adenosine-34 deaminase activity, role in adenosine to inosine editing and cytosol, nucleus, tRNA-specific adenosine-34 deaminase complex localization |
| AN5156 | Pho80-like cyclin involved in regulation of development and phosphate homeostasis, interacts with the cyclin-dependent kinase PhoA |
| AN5157 | Ortholog of A. fumigatus Af293 : Afu1g07050, A. niger CBS 513.88 : An07g09610, A. oryzae RIB40 : AO090012000957, Aspergillus wentii : Aspwe1_0051655 and Aspergillus sydowii : Aspsy1_0091619 |
| AN5170 | Putative Zn(II)2Cys6 transcription factor |
| AN5177 | Ortholog(s) have role in ribosomal large subunit biogenesis and nucleolus localization |
| AN5178 | Putative glycerol dehydrogenase with a predicted role in glycerol metabolism |
| AN5179 | Ortholog(s) have role in peptidyl-diphthamide biosynthetic process from peptidyl-histidine and cytosol localization |
| AN5181 | Protein involved in nuclear migration |
| AN5188 | Ortholog(s) have nuclear chromatin, nucleolus localization |
| AN5189 | protein of unknown function |
| AN5197 | Has domain(s) with predicted calcium ion binding, zinc ion binding activity |
| AN5203 | Ortholog(s) have structural constituent of ribosome activity and mitochondrial small ribosomal subunit localization |
| AN5204 | Ortholog of A. fumigatus Af293 : Afu6g07370, A. niger CBS 513.88 : An07g09060, A. oryzae RIB40 : AO090005001564, Aspergillus wentii : Aspwe1_0040832 and Aspergillus sydowii : Aspsy1_0156764 |
| AN5206 | Putative homoisocitrate dehydrogenase with a predicted role in lysine metabolism |
| AN5211 | Ortholog(s) have 1-phosphatidylinositol-3-phosphate 5-kinase activity, phosphatidylinositol-3-phosphate binding activity |
| AN5212 | Ortholog of A. fumigatus Af293 : Afu6g07460, A. niger CBS 513.88 : An07g08970, Aspergillus wentii : Aspwe1_0028223, Aspergillus sydowii : Aspsy1_0033560 and Aspergillus terreus NIH2624 : ATET_03679 |
| AN5215 | Ortholog(s) have role in endocytic recycling and cytosol, endosome, nucleus localization |
| AN5217 | Protein with similarity to sphingolipid long chain base-responsive protein Pil1 |
| AN5220 | Ortholog(s) have role in filamentous growth of a population of unicellular organisms in response to biotic stimulus and growth of unicellular organism as a thread of attached cells, more |
| AN5225 | Ortholog of A. fumigatus Af293 : Afu4g04605, Afu2g04095, A. niger CBS 513.88 : An13g04060, An07g08820, A. oryzae RIB40 : AO090206000029 and Aspergillus wentii : Aspwe1_0109774, Aspwe1_0159657 |
| AN5234 | Has domain(s) with predicted metal ion binding, oxidoreductase activity and role in oxidation-reduction process |
| AN5236 | Ortholog(s) have Golgi apparatus localization |
| AN5244 | Ortholog of A. nidulans FGSC A4 : AN5251, A. niger CBS 513.88 : An11g07550, An08g12150, A. oryzae RIB40 : AO090011000256, Aspergillus wentii : Aspwe1_0113272 and Aspergillus versicolor : Aspve1_0062915 |
| AN5251 | Ortholog of A. nidulans FGSC A4 : AN5244, A. niger CBS 513.88 : An11g07550, An08g12150, A. oryzae RIB40 : AO090011000256, Aspergillus wentii : Aspwe1_0113272 and Aspergillus versicolor : Aspve1_0062915 |
| AN5270 | Putative acyl-CoA dehydrogenase |
| AN5281 | Putative pyranose oxidase |
| AN5305 | Has domain(s) with predicted catalytic activity |
| AN5321 | Putative triacylglycerol lipase with a predicted role in glycerolipid metabolism |
| AN5334 | Predicted DDE1 transposon-related ORF |
| AN5342 | Protein expressed at increased levels during osmoadaptation |
| AN5347 | Has domain(s) with predicted cation transmembrane transporter activity, role in cation transport, transmembrane transport and integral component of membrane localization |
| AN5349 | Has domain(s) with predicted RNA polymerase II transcription factor activity, sequence-specific DNA binding, zinc ion binding activity, role in regulation of transcription, DNA-templated and nucleus localization |
| AN5362 | Ortholog of Aspergillus niger ATCC 1015 : 41393-mRNA |
| AN5373 | Has domain(s) with predicted oxidoreductase activity and role in metabolic process |
| AN5374 | protein of unknown function |
| AN5375 | Has domain(s) with predicted RNA polymerase II transcription cofactor activity, role in regulation of transcription from RNA polymerase II promoter and mediator complex localization |
| AN5434 | Has domain(s) with predicted oxidoreductase activity, transferase activity, transferring acyl groups other than amino-acyl groups, zinc ion binding activity and role in oxidation-reduction process |
| AN5437 | Ortholog of A. nidulans FGSC A4 : AN2941, A. fumigatus Af293 : Afu3g08250, Afu6g13630, A. niger CBS 513.88 : An02g11860 and A. oryzae RIB40 : AO090005001453, AO090103000325 |
| AN5442 | Putative carboxypeptidase Y |
| AN5444 | Putative tryptophan synthase with a predicted role in aromatic amino acid biosynthesis |
| AN5445 | Ortholog of A. fumigatus Af293 : Afu6g13510, A. niger CBS 513.88 : An08g08780, A. oryzae RIB40 : AO090103000339, Aspergillus wentii : Aspwe1_0033546 and Aspergillus sydowii : Aspsy1_0057232 |
| AN5446 | Ortholog of A. fumigatus Af293 : Afu6g13500, A. niger CBS 513.88 : An08g08820, A. oryzae RIB40 : AO090103000340, Neosartorya fischeri NRRL 181 : NFIA_059450 and Aspergillus versicolor : Aspve1_0084095 |
| AN5449 | Has domain(s) with predicted catalytic activity, sulfuric ester hydrolase activity and role in metabolic process |
| AN5455 | Ortholog(s) have snoRNA binding activity |
| AN5461 | Ortholog of A. fumigatus Af293 : Afu5g00130, A. niger CBS 513.88 : An01g00570, Aspergillus wentii : Aspwe1_0022994, Aspergillus versicolor : Aspve1_0039792 and Aspergillus clavatus NRRL 1 : ACLA_024300 |
| AN5462 | protein of unknown function |
| AN5476 | Has domain(s) with predicted DNA binding, chromatin binding activity |
| AN5481 | Has domain(s) with predicted Rho guanyl-nucleotide exchange factor activity, phospholipid binding activity, role in regulation of Rho protein signal transduction and intracellular localization |
| AN5482 | Putative Ran GTPase, GTP binding protein |
| AN5487 | Ortholog of A. nidulans FGSC A4 : AN2619, A. niger CBS 513.88 : An11g05410, An12g09960, An12g02190 and Aspergillus wentii : Aspwe1_0167938 |
| AN5501 | Ortholog(s) have lipase activity |
| AN5524 | Has domain(s) with predicted hydrolase activity |
| AN5530 | Ortholog(s) have U2 snRNP, cytosol localization |
| AN5532 | Has domain(s) with predicted nucleic acid binding, zinc ion binding activity |
| AN5533 | Has domain(s) with predicted RNA polymerase II transcription factor activity, sequence-specific DNA binding, zinc ion binding activity, role in regulation of transcription, DNA-templated and nucleus localization |
| AN5536 | Ortholog(s) have cytosol localization |
| AN5538 | protein of unknown function |
| AN5553 | Putative cytochrome P450 |
| AN5563 | Putative dehydrogenase with a predicted role in carbohydrate metabolism |
| AN5564 | Putative phosphatidyl synthase |
| AN5570 | Ortholog(s) have methylated histone binding activity |
| AN5571 | Putative oxoglutarate dehydrogenase (lipoamide) with a predicted role in the TCA cycle |
| AN5572 | Ortholog of A. fumigatus Af293 : Afu4g11640, A. niger CBS 513.88 : An04g04740, Aspergillus wentii : Aspwe1_0047362, Aspergillus sydowii : Aspsy1_0057118 and Aspergillus terreus NIH2624 : ATET_03912 |
| AN5573 | Ortholog of A. fumigatus Af293 : Afu4g11630, A. niger CBS 513.88 : An04g04730, A. oryzae RIB40 : AO090003001056, Aspergillus wentii : Aspwe1_0166629 and Aspergillus sydowii : Aspsy1_0088453 |
| AN5574 | Ortholog(s) have role in GINS complex assembly, double-strand break repair via break-induced replication, mitotic DNA replication initiation, mitotic DNA replication preinitiation complex assembly |
| AN5576 | Ortholog of A. fumigatus Af293 : Afu4g11590, A. niger CBS 513.88 : An04g04860, A. oryzae RIB40 : AO090003001061, Aspergillus wentii : Aspwe1_0166635 and Aspergillus sydowii : Aspsy1_0149581 |
| AN5578 | Ortholog(s) have mitotic spindle pole body, nucleus localization |
| AN5579 | Ortholog(s) have role in ascospore-type prospore membrane assembly, late endosome to vacuole transport, protein retention in Golgi apparatus, protein targeting to vacuole |
| AN5580 | Ortholog of A. fumigatus Af293 : Afu4g11550, A. niger CBS 513.88 : An04g04940, Aspergillus wentii : Aspwe1_0022740, Aspergillus sydowii : Aspsy1_0057110 and Aspergillus terreus NIH2624 : ATET_03924 |
| AN5581 | Ortholog of A. nidulans FGSC A4 : AN6527, A. fumigatus Af293 : Afu6g04900, Afu4g11477, A. niger CBS 513.88 : An14g03410, An04g05110 and A. oryzae RIB40 : AO090701000049, AO090003001075 |
| AN5586 | Putative mannose-1-phosphate guanylyltransferase with a predicted role in mannose/mannitol, fructose, and sorbose/sorbitol metabolism |
| AN5597 | Putative ubiquinol-cytochrome-c reductase subunit with a predicted role in energy metabolism |
| AN5599 | Putative glycerol-3-phosphate acyltransferase with a predicted role in phospholipid metabolism |
| AN5600 | Ortholog(s) have dehydrodolichyl diphosphate synthase activity, prenyltransferase activity, role in dolichol biosynthetic process and intrinsic component of membrane localization |
| AN5606 | Ortholog(s) have proton-transporting ATPase activity, rotational mechanism activity and role in endocytosis, polyphosphate metabolic process, protein complex assembly, proton transport, vacuolar acidification, vacuole organization |
| AN5612 | Ortholog of A. fumigatus Af293 : Afu4g11230, A. niger CBS 513.88 : An04g05430, A. oryzae RIB40 : AO090003001098, Aspergillus wentii : Aspwe1_0022777 and Aspergillus sydowii : Aspsy1_0148394 |
| AN5613 | Xanthine dehydrogenase (purine hydroxylase I) involved in purine catabolism |
| AN5616 | Ortholog(s) have cytosol, nucleus localization |
| AN5617 | Has domain(s) with predicted ATP binding, DNA binding, nucleoside-triphosphatase activity, nucleotide binding activity and role in DNA repair |
| AN5618 | Putative spindle pole body protein |
| AN5623 | Ortholog(s) have mitochondrion localization |
| AN5626 | Acetyl-CoA synthase, required for utilization of acetate as a carbon source |
| AN5627 | Nuclear pore complex protein with homology to human NUP98/NUP96 |
| AN5631 | Ortholog(s) have role in G1/S transition of mitotic cell cycle, regulation of mitotic sister chromatid segregation, tRNA wobble uridine modification and cytosol, mitochondrion, nuclear pericentric heterochromatin localization |
| AN5632 | Ortholog(s) have role in cellular response to drug, filamentous growth, late endosome to vacuole transport via multivesicular body sorting pathway and fungal-type vacuole membrane localization |
| AN5638 | Ortholog(s) have ubiquitin-specific protease activity, role in protein deubiquitination and cytosol, ribosome localization |
| AN5642 | Ortholog of A. fumigatus Af293 : Afu4g13470, A. niger CBS 513.88 : An01g09190, A. oryzae RIB40 : AO090009000225, Aspergillus wentii : Aspwe1_0206891 and Aspergillus sydowii : Aspsy1_0030327 |
| AN5652 | Putative hydantoinase/oxoprolinase |
| AN5657 | Ortholog of A. fumigatus Af293 : Afu4g13590, A. niger CBS 513.88 : An01g09400, A. oryzae RIB40 : AO090009000210, Aspergillus wentii : Aspwe1_0047218 and Aspergillus sydowii : Aspsy1_0199958 |
| AN5663 | Ortholog of A. fumigatus Af293 : Afu4g13710, A. niger CBS 513.88 : An01g09510, A. oryzae RIB40 : AO090010000145, AO090009000200 and Aspergillus wentii : Aspwe1_0106727 |
| AN5669 | Putative succinyl-CoA:3-ketoacid-coenzyme A transferase |
| AN5670 | Ortholog(s) have role in mitochondrial respiratory chain complex I assembly and mitochondrial membrane localization |
| AN5673 | Putative transcription factor involved in regulation of rhamnose utilization |
| AN5675 | Ortholog(s) have ubiquitin binding activity, role in proteasome-mediated ubiquitin-dependent protein catabolic process and cytoplasm, nucleus localization |
| AN5676 | Putative pre-initiation complex, subunit |
| AN5681 | Ortholog(s) have ubiquitin-protein transferase activity, role in protein import into peroxisome matrix, protein polyubiquitination and peroxisomal importomer complex, peroxisomal membrane localization |
| AN5682 | Ortholog(s) have role in protein glycosylation, protein retention in ER lumen and fungal-type vacuole membrane localization |
| AN5687 | Ortholog of A. fumigatus Af293 : Afu7g04200, A. niger CBS 513.88 : An13g00750, A. oryzae RIB40 : AO090005001653, Aspergillus wentii : Aspwe1_0038498 and Aspergillus sydowii : Aspsy1_0030277 |
| AN5715 | Putative 40s ribosomal protein S26 |
| AN5721 | Ortholog(s) have role in proteasome-mediated ubiquitin-dependent protein catabolic process and cytosol localization |
| AN5722 | Putative protein serine/threonine phosphatase |
| AN5724 | Ortholog(s) have role in respiratory chain complex IV assembly and mitochondrial inner membrane localization |
| AN5725 | Protein with a predicted role in asparagine-linked glycosylation |
| AN5730 | Ortholog of A. fumigatus Af293 : Afu1g06930, A. oryzae RIB40 : AO090003000083, Aspergillus wentii : Aspwe1_0109001, Aspergillus sydowii : Aspsy1_0043701 and Aspergillus terreus NIH2624 : ATET_02925 |
| AN5731 | Putative chorismate synthase with a predicted role in aromatic amino acid biosynthesis |
| AN5733 | Ortholog of A. fumigatus Af293 : Afu6g06970, A. oryzae RIB40 : AO090003000079, Neosartorya fischeri NRRL 181 : NFIA_052620, Aspergillus wentii : Aspwe1_0050160 and Aspergillus versicolor : Aspve1_0132614 |
| AN5738 | Ortholog(s) have methionyl-tRNA formyltransferase activity, role in conversion of methionyl-tRNA to N-formyl-methionyl-tRNA and mitochondrion localization |
| AN5748 | Putative mannosyl-oligosaccharide 1,2-alpha-mannosidase with a predicted role in mannose polymer metabolism |
| AN5762 | Predicted glycosylphosphatidylinositol (GPI)-anchored protein |
| AN5763 | Has domain(s) with predicted role in transmembrane transport and integral component of membrane localization |
| AN5765 | Ortholog of A. fumigatus Af293 : Afu2g09480, A. oryzae RIB40 : AO090011000718, Aspergillus flavus NRRL 3357 : AFL2T_05450 and Neosartorya fischeri NRRL 181 : NFIA_084970 |
| AN5767 | Has domain(s) with predicted phosphatase activity, protein tyrosine phosphatase activity and role in dephosphorylation |
| AN5770 | Ortholog(s) have endoplasmic reticulum localization |
| AN5780 | Ortholog of A. fumigatus Af293 : Afu6g06480, A. niger CBS 513.88 : An18g06640, Aspergillus wentii : Aspwe1_0177889, Aspergillus versicolor : Aspve1_0053552 and Aspergillus clavatus NRRL 1 : ACLA_088250 |
| AN5785 | Ortholog(s) have unfolded protein binding activity |
| AN5786 | Ortholog(s) have role in cellular ion homeostasis, establishment or maintenance of cell polarity, mitochondrion inheritance, regulation of cardiolipin metabolic process and mitochondrial inner membrane, nucleus localization |
| AN5788 | Ortholog(s) have COPII adaptor activity and role in ER to Golgi vesicle-mediated transport, fungal-type cell wall organization, protein glycosylation, protein retention in Golgi apparatus |
| AN5790 | Putative isocitrate dehydrogenase (NAD+) with a predicted role in the TCA cycle |
| AN5791 | Predicted pheromone precursor |
| AN5803 | Predicted fimbrin protein |
| AN5804 | Ortholog(s) have mitochondrion localization |
| AN5826 | PASA transcript |
| AN5828 | Has domain(s) with predicted heme binding activity |
| AN5833 | Putative acetyl-CoA synthase with a predicted role in two-carbon metabolism or the methylcitrate pathway |
| AN5838 | Has domain(s) with predicted FMN binding, iron ion binding, oxidoreductase activity and role in oxidation-reduction process |
| AN5839 | Ortholog(s) have cytosol localization |
| AN5841 | Ortholog of A. nidulans FGSC A4 : AN8634, A. fumigatus Af293 : Afu8g07320, Aspergillus wentii : Aspwe1_0062778, Aspergillus sydowii : Aspsy1_0048400 and Aspergillus terreus NIH2624 : ATET_08649 |
| AN5847 | Ortholog of A. fumigatus Af293 : Afu2g08000, A. niger CBS 513.88 : An05g00550, Neosartorya fischeri NRRL 181 : NFIA_083670 and Aspergillus wentii : Aspwe1_0175059, Aspwe1_0670697 |
| AN5853 | Ortholog of Aspergillus glaucus : Aspgl1_0040039, Aspergillus flavus NRRL 3357 : AFL2T_00523, Aspergillus versicolor : Aspve1_0131121 and Aspergillus sydowii : Aspsy1_0089204 |
| AN5861 | Ortholog(s) have ketoreductase activity, role in fatty acid elongation, sphingolipid biosynthetic process, very long-chain fatty acid biosynthetic process and endoplasmic reticulum membrane localization |
| AN5862 | Putative ortholog of S. cerevisiae Erg28p |
| AN5863 | Predicted DDE1 transposon-related ORF |
| AN5867 | Ortholog(s) have acetyl-CoA:L-glutamate N-acetyltransferase activity, role in ornithine biosynthetic process and mitochondrial matrix, mitochondrial membrane localization |
| AN5876 | Ortholog of A. nidulans FGSC A4 : AN2876, A. fumigatus Af293 : Afu2g11370, Afu3g11770, Aspergillus wentii : Aspwe1_0653753 and Aspergillus sydowii : Aspsy1_0146596, Aspsy1_1176317 |
| AN5897 | Ortholog(s) have structural constituent of ribosome activity and mitochondrial large ribosomal subunit localization |
| AN5899 | Putative condensin |
| AN5901 | Ortholog(s) have role in DNA-dependent DNA replication, double-strand break repair via break-induced replication and GINS complex, replication fork protection complex localization |
| AN5902 | Ortholog(s) have dolichyl-phosphate-glucose-glycolipid alpha-glucosyltransferase activity, role in protein N-linked glycosylation and endoplasmic reticulum membrane localization |
| AN5910 | Ortholog(s) have RNA polymerase II C-terminal domain phosphoserine binding, RNA polymerase II core binding, chromatin binding and transcription factor activity, more |
| AN5914 | Has domain(s) with predicted methyltransferase activity |
| AN5917 | Ortholog(s) have alpha-glucoside:proton symporter activity, maltose:proton symporter activity, trehalose transmembrane transporter activity and role in disaccharide catabolic process, maltose transport, trehalose transport |
| AN5918 | Putative catalase with a predicted role in gluconic acid and gluconate metabolism |
| AN5919 | Ortholog(s) have role in macroautophagy, multivesicular body membrane disassembly, neutral lipid catabolic process, piecemeal microautophagy of nucleus, vacuolar protein processing |
| AN5923 | protein of unknown function |
| AN5926 | Ortholog(s) have endoplasmic reticulum localization |
| AN5930 | Has domain(s) with predicted hydrolase activity |
| AN5939 | Putative 5'-nucleotidase with a predicted role in nucleotide salvage pathways |
| AN5944 | Ortholog of A. niger CBS 513.88 : An11g00100, A. oryzae RIB40 : AO090009000720, Aspergillus wentii : Aspwe1_0173103, Aspergillus versicolor : Aspve1_0147448 and Aspergillus niger ATCC 1015 : 179912-mRNA |
| AN5945 | Ortholog of A. fumigatus Af293 : Afu6g14630, Neosartorya fischeri NRRL 181 : NFIA_060670 and Aspergillus terreus NIH2624 : ATET_10017 |
| AN5947 | Ortholog of A. niger CBS 513.88 : An11g05770, Neosartorya fischeri NRRL 181 : NFIA_007650, Aspergillus versicolor : Aspve1_0047552 and Aspergillus zonatus : Aspzo1_0020862 |
| AN5962 | Has domain(s) with predicted nucleic acid binding activity |
| AN5966 | Putative zinc-finger DNA binding protein |
| AN5966-uORF | Conserved upstream open reading frame (uORF) of AN5966 |
| AN5970 | Ortholog(s) have protein disulfide isomerase activity, role in protein folding and Golgi apparatus, endoplasmic reticulum localization |
| AN5972 | Ortholog(s) have ubiquitin binding activity |
| AN5986 | Putative reductase with a predicted role in carbohydrate metabolism |
| AN5990 | Putative long-chain-fatty-acid-CoA ligase with a predicted role in fatty acid metabolism |
| AN5992 | Ortholog(s) have ATP-dependent 3'-5' DNA helicase activity, single-stranded DNA binding, single-stranded DNA-dependent ATP-dependent DNA helicase activity and MCM complex, MCM core complex, nuclear chromatin localization |
| AN6004 | Protein with an RNA recognition motif |
| AN6022 | Has domain(s) with predicted oxidoreductase activity, oxidoreductase activity, acting on the aldehyde or oxo group of donors, NAD or NADP as acceptor activity and role in oxidation-reduction process |
| AN6038 | Ortholog of A. fumigatus Af293 : Afu2g09780, A. niger CBS 513.88 : An16g05430, A. oryzae RIB40 : AO090011000660, Aspergillus wentii : Aspwe1_0045086 and Aspergillus sydowii : Aspsy1_0132339 |
| AN6041 | Ortholog(s) have mitochondrion localization |
| AN6042 | Has domain(s) with predicted nucleic acid binding, nucleotide binding activity |
| AN6043 | protein of unknown function |
| AN6052 | Ortholog of A. oryzae RIB40 : AO090011000694, Aspergillus flavus NRRL 3357 : AFL2T_05430 and Aspergillus sydowii : Aspsy1_0031385 |
| AN6058 | DUF833 domain-containing protein |
| AN6059 | Ortholog of A. fumigatus Af293 : Afu2g09470, A. niger CBS 513.88 : An16g06820, A. oryzae RIB40 : AO090011000717, Aspergillus wentii : Aspwe1_0054380 and Aspergillus sydowii : Aspsy1_0045140 |
| AN6064 | Ortholog(s) have hydrolase activity, acting on ester bonds activity |
| AN6066 | Has domain(s) with predicted catalytic activity and role in metabolic process |
| AN6067 | Ortholog(s) have GDP-dissociation inhibitor activity, GTPase activator activity, translation initiation factor activity, translation initiation factor binding activity |
| AN6072 | Ortholog(s) have protein-arginine N5-methyltransferase activity, role in peptidyl-arginine methylation and cytosol, nucleus localization |
| AN6074 | Ortholog(s) have mRNA binding, protein heterodimerization activity, role in mRNA polyadenylation, pre-mRNA cleavage required for polyadenylation, response to DNA damage checkpoint signaling and mRNA cleavage factor complex localization |
| AN6080 | Ortholog(s) have role in retrograde vesicle-mediated transport, Golgi to ER and COPI vesicle coat, cytosol, nucleus localization |
| AN6085 | Ortholog(s) have nucleolus localization |
| AN6086 | Putative F-box protein |
| AN6088 | Predicted metal ion transmembrane transporter |
| AN6090 | Ortholog(s) have peptide alpha-N-acetyltransferase activity, role in N-terminal protein amino acid acetylation and NatC complex, cytosol, nucleus localization |
| AN6101 | Putative cytochrome P450 |
| AN6119 | Ortholog(s) have endoplasmic reticulum, fungal-type vacuole localization |
| AN6120 | Predicted ADP ribosylation factor guanine nucleotide exchange factor (Arf GEF) |
| AN6126 | Putative acetyl-CoA carboxylase with a predicted role in cytosolic fatty acid formation |
| AN6133 | Putative succinate dehydrogenase |
| AN6138 | Component of the Anaphase-Promoting Complex/Cyclosome (APC/C), which is a ubiquitin ligase required for cell cycle progression |
| AN6154 | Ortholog of A. nidulans FGSC A4 : AN5342, A. fumigatus Af293 : Afu2g04510, Afu3g02680, Afu3g12240, Afu6g14230, Afu8g01830, Afu8g01940 and A. niger CBS 513.88 : An06g01180, An01g03400, An12g05490, An11g05900 |
| AN6177 | Putative flotillin ortholog, involved in maintenance of sterol-rich plasma membrane domains |
| AN6183 | Putative F-box protein |
| AN6189 | Putative nucleoside triphosphatase with a predicted role in nucleotide salvage pathways |
| AN6191 | Ortholog(s) have cytoplasm localization |
| AN6193 | Ortholog(s) have ATP-dependent peptidase activity, role in chaperone-mediated protein complex assembly, misfolded or incompletely synthesized protein catabolic process and mitochondrial matrix localization |
| AN6197 | Nuclear migration protein |
| AN6198 | Ortholog of A. fumigatus Af293 : Afu2g11790, A. niger CBS 513.88 : An02g03840, A. oryzae RIB40 : AO090026000463, Aspergillus wentii : Aspwe1_0175598 and Aspergillus sydowii : Aspsy1_0152269 |
| AN6203 | Ortholog of A. fumigatus Af293 : Afu2g11860, A. oryzae RIB40 : AO090026000457, Aspergillus tubingensis : Asptu1_0039119 and Aspergillus brasiliensis : Aspbr1_0038813 |
| AN6209 | Putative 5'-phosphoribosyl-4-(N-succinocarboxamide)-5-aminoimidazole lyase with a predicted role in purine metabolism |
| AN6210 | Ortholog(s) have GTP-Rho binding, phosphatidylinositol-4,5-bisphosphate binding activity and role in Golgi to plasma membrane transport, Rho protein signal transduction, exocyst assembly, exocyst localization |
| AN6214 | Putative Hat1 acetyltransferase |
| AN6215 | Has domain(s) with predicted 3-hydroxyacyl-CoA dehydrogenase activity, coenzyme binding, oxidoreductase activity, acting on the CH-OH group of donors, NAD or NADP as acceptor activity |
| AN6216 | Has domain(s) with predicted metal ion transmembrane transporter activity, role in metal ion transport, transmembrane transport and membrane localization |
| AN6220 | Ortholog of S. cerevisiae : YMR155W, A. fumigatus Af293 : Afu2g13390, A. niger CBS 513.88 : An02g02260, A. oryzae RIB40 : AO090026000316 and Aspergillus wentii : Aspwe1_0118199 |
| AN6228 | Ortholog(s) have cytosol localization |
| AN6232 | Putative F1F0-ATPase complex subunit with a predicted role in energy metabolism |
| AN6249 | Putative calcineurin binding protein, calcipressin |
| AN6251 | Predicted hydrolase |
| AN6262 | Ortholog of A. fumigatus Af293 : Afu2g12930, A. oryzae RIB40 : AO090026000341, Aspergillus wentii : Aspwe1_0031317, Aspergillus sydowii : Aspsy1_0058174 and Aspergillus terreus NIH2624 : ATET_01256 |
| AN6275 | Ortholog of A. fumigatus Af293 : Afu2g12570, A. niger CBS 513.88 : An02g01500, A. oryzae RIB40 : AO090026000362, Aspergillus wentii : Aspwe1_0116943 and Aspergillus sydowii : Aspsy1_0151369 |
| AN6276 | Has domain(s) with predicted 4-alpha-hydroxytetrahydrobiopterin dehydratase activity and role in tetrahydrobiopterin biosynthetic process |
| AN6278 | Ortholog of A. fumigatus Af293 : Afu2g12540, Neosartorya fischeri NRRL 181 : NFIA_087680, Aspergillus wentii : Aspwe1_0071673 and Aspergillus versicolor : Aspve1_0041153 |
| AN6279 | Carnitine acetyltransferase, required for utilization of acetate and fatty acids |
| AN6281 | Ortholog of Aspergillus versicolor : Aspve1_0028217 and Aspergillus sydowii : Aspsy1_0151548 |
| AN6283 | Ortholog(s) have role in peptidyl-diphthamide biosynthetic process from peptidyl-histidine and cytosol, nucleus localization |
| AN6295 | Has domain(s) with predicted protein dimerization activity |
| AN6296 | Ortholog of A. fumigatus Af293 : Afu2g12300, A. niger CBS 513.88 : An02g04340, A. oryzae RIB40 : AO090026000420, Aspergillus wentii : Aspwe1_0118064 and Aspergillus sydowii : Aspsy1_0152200 |
| AN6299 | Has domain(s) with predicted magnesium ion transmembrane transporter activity, role in magnesium ion transport and integral component of membrane localization |
| AN6300 | Ortholog(s) have role in DNA strand elongation involved in DNA replication, UV-damage excision repair and Elg1 RFC-like complex, cytosol, nucleus localization |
| AN6304 | Ortholog(s) have role in establishment of cell polarity, hyphal growth, positive regulation of mitotic cell cycle, regulation of actin cytoskeleton organization and TORC2 complex, cytosol localization |
| AN6312 | Ortholog(s) have cytosol, trimeric positive transcription elongation factor complex b localization |
| AN6315 | Ortholog of A. nidulans FGSC A4 : AN9346, A. niger CBS 513.88 : An13g01700, An08g05310, A. oryzae RIB40 : AO090020000178 and Aspergillus sydowii : Aspsy1_0072048, Aspsy1_0132512, Aspsy1_1153861 |
| AN6319 | Ortholog of A. nidulans FGSC A4 : AN0638, A. fumigatus Af293 : Afu1g16870, A. oryzae RIB40 : AO090005000988, Aspergillus wentii : Aspwe1_0038696 and Aspergillus sydowii : Aspsy1_0129541, Aspsy1_0995740 |
| AN6322 | Has domain(s) with predicted RNA polymerase II transcription factor activity, sequence-specific DNA binding, zinc ion binding activity, role in regulation of transcription, DNA-templated and nucleus localization |
| AN6334 | Ortholog(s) have mitotic spindle, nucleolus localization |
| AN6347 | Has domain(s) with predicted ATP binding, protein kinase activity, protein tyrosine kinase activity and role in protein phosphorylation |
| AN6348 | Ortholog(s) have mitochondrion targeting sequence binding, protein channel activity, role in protein import into mitochondrial matrix and mitochondrial inner membrane presequence translocase complex, plasma membrane localization |
| AN6349 | Ortholog of A. fumigatus Af293 : Afu2g14180, A. niger CBS 513.88 : An02g01370, A. oryzae RIB40 : AO090023000134, Aspergillus wentii : Aspwe1_0697288 and Aspergillus sydowii : Aspsy1_0178307 |
| AN6352 | Protein with arabinan endo-1,5-alpha-L-arabinosidase activity, involved in degradation of pectin |
| AN6356 | protein of unknown function |
| AN6376 | Ortholog(s) have structural constituent of ribosome activity and mitochondrial large ribosomal subunit localization |
| AN6378 | Ortholog of A. niger CBS 513.88 : An02g05060, A. oryzae RIB40 : AO090026000168, Aspergillus wentii : Aspwe1_0054681, Aspergillus sydowii : Aspsy1_0045559 and Aspergillus terreus NIH2624 : ATET_01381 |
| AN6391 | Protein phosphatase |
| AN6395 | Protein with rhamnogalacturonan lyase activity, involved in degradation of pectin |
| AN6399 | Putative bleomycin hydrolase |
| AN6403 | Ortholog of Aspergillus clavatus NRRL 1 : ACLA_055850 and Aspergillus aculeatus ATCC16872 : Aacu16872_061969 |
| AN6426 | Has domain(s) with predicted metallopeptidase activity |
| AN6433 | Has domain(s) with predicted transferase activity, transferring hexosyl groups activity and role in metabolic process |
| AN6434 | Putative cytochrome P450 |
| AN6443 | Predicted ABC transporter, encoded within the cichorine gene cluster |
| AN6446 | Predicted transcription factor |
| AN6464 | Has domain(s) with predicted hydrolase activity, hydrolase activity, acting on ester bonds activity and role in lipid metabolic process |
| AN6476 | protein of unknown function |
| AN6478 | protein of unknown function |
| AN6479 | protein of unknown function |
| AN6481 | Has domain(s) with predicted metal ion transmembrane transporter activity, role in metal ion transport, transmembrane transport and membrane localization |
| AN6485 | Putative cytochrome P450 |
| AN6501 | Ortholog(s) have U2 snRNP, cytosol localization |
| AN6514 | Ortholog(s) have GDP binding, RNA pyrophosphohydrolase activity, enzyme regulator activity, phosphodiesterase decapping endonuclease activity |
| AN6527 | Ortholog of A. nidulans FGSC A4 : AN5581, A. fumigatus Af293 : Afu6g04900, Afu4g11477, A. niger CBS 513.88 : An14g03410, An04g05110 and A. oryzae RIB40 : AO090701000049, AO090003001075 |
| AN6528 | Protein predicted to have a role in pheromone precursor processing |
| AN6538 | Has domain(s) with predicted phospholipid binding activity |
| AN6544 | Ortholog(s) have role in late endosome to vacuole transport via multivesicular body sorting pathway, protein targeting to vacuole and Golgi apparatus, Vps55/Vps68 complex, fungal-type vacuole membrane, mitochondrion localization |
| AN6549 | Ortholog(s) have RNA polymerase II transcription coactivator activity involved in preinitiation complex assembly activity |
| AN6551 | Has domain(s) with predicted ATP binding activity |
| AN6552 | Putative F-box protein |
| AN6553 | Ortholog(s) have role in cohesin localization to chromatin, establishment of mitotic sister chromatid cohesion, positive regulation of maintenance of mitotic sister chromatid cohesion |
| AN6562 | Ortholog of A. fumigatus Af293 : Afu6g04580, A. niger CBS 513.88 : An15g00740, A. oryzae RIB40 : AO090701000088, Aspergillus wentii : Aspwe1_0036108 and Aspergillus sydowii : Aspsy1_0051771 |
| AN6566 | Calcium-dependent protein serine/threonine phosphatase |
| AN6567 | Ortholog(s) have role in histone acetylation and NuA4 histone acetyltransferase complex localization |
| AN6581 | Has domain(s) with predicted ATP binding, ATPase activity, ATPase activity, coupled to transmembrane movement of substances, nucleoside-triphosphatase activity, nucleotide binding activity and role in transport |
| AN6596 | Ortholog of A. fumigatus Af293 : Afu6g04060, A. niger CBS 513.88 : An15g01300, A. oryzae RIB40 : AO090701000130, Aspergillus wentii : Aspwe1_0065449 and Aspergillus sydowii : Aspsy1_0051825 |
| AN6602 | Has domain(s) with predicted oxidoreductase activity and role in metabolic process |
| AN6604 | Ortholog(s) have ATPase activity, tRNA binding activity, role in tRNA modification and Elongator holoenzyme complex, cytosol, nucleus localization |
| AN6611 | Ortholog(s) have role in ascospore formation, asymmetric protein localization to old mitotic spindle pole body, intra-S DNA damage checkpoint and negative regulation of septation initiation signaling, more |
| AN6616 | Protein with homology to PCNA-like DNA damage sensor |
| AN6617 | Has domain(s) with predicted role in cell cycle arrest |
| AN6621 | Ortholog of A. nidulans FGSC A4 : AN7403, A. fumigatus Af293 : Afu8g01810, A. niger CBS 513.88 : An05g02280, A. oryzae RIB40 : AO090026000198 and Aspergillus wentii : Aspwe1_0151553, Aspwe1_0177691 |
| AN6622 | Ortholog of Aspergillus versicolor : Aspve1_0197528, Aspergillus sydowii : Aspsy1_0095517, Aspergillus terreus NIH2624 : ATET_01376 and Aspergillus carbonarius ITEM 5010 : Acar5010_508676 |
| AN6626 | Has domain(s) with predicted integral component of membrane localization |
| AN6628 | Ortholog(s) have role in establishment or maintenance of cell polarity, negative regulation of G0 to G1 transition |
| AN6630 | Putative nascent polypeptide-associated complex subunit alpha |
| AN6631 | Putative F1F0-ATPase complex subunit with a predicted role in energy metabolism |
| AN6643 | Putative biotin synthase with a predicted role in Coenzyme A and pantothenate biosynthesis |
| AN6644 | Putative bifunctional dethiobiotin synthetase/adenosylmethionine-8-amino-7-oxononanoate aminotransferase, enzyme of the biotin biosynthesis pathway |
| AN6645 | Putative 8-amino-7-oxononanoate synthase with a predicted role in Coenzyme A and pantothenate biosynthesis |
| AN6646 | protein of unknown function |
| AN6653 | Malate synthase, required for utilization of acetate as carbon source |
| AN6663 | protein of unknown function |
| AN6666 | protein of unknown function |
| AN6669 | High-affinity glucose transporter active in germinating conidia |
| AN6677 | Ortholog(s) have histone methyltransferase activity (H3-K4 specific) activity, role in chromatin silencing at telomere, histone H3-K4 methylation and Set1C/COMPASS complex localization |
| AN6678 | Ortholog(s) have role in interstrand cross-link repair and nucleus localization |
| AN6689 | Ortholog(s) have structural constituent of nuclear pore activity |
| AN6693 | protein of unknown function |
| AN6694 | Ortholog(s) have role in attachment of spindle microtubules to kinetochore involved in homologous chromosome segregation and cytosol, nucleus localization |
| AN6698 | Ortholog(s) have nucleolus localization |
| AN6723 | Putative 2,3-dihydroxybenzoate carboxylyase |
| AN6724 | protein of unknown function |
| AN6738 | Putative nuclear pore complex protein |
| AN6740 | protein of unknown function |
| AN6744 | Ortholog of A. niger CBS 513.88 : An07g01410, Neosartorya fischeri NRRL 181 : NFIA_113290, Aspergillus wentii : Aspwe1_0023957 and Aspergillus versicolor : Aspve1_0056607 |
| AN6750 | Has domain(s) with predicted FAD binding, oxidoreductase activity and role in metabolic process |
| AN6753 | Putative NADH-dependent flavin oxidoreductase |
| AN6774 | Has domain(s) with predicted role in transmembrane transport and integral component of membrane localization |
| AN6781 | protein of unknown function |
| AN6787 | Putative cytochrome P450 |
| AN6790 | putative transcription factor |
| AN6791 | Putative polyketide synthase |
| AN6792 | Putative NAD+ dependent glycerol 3-phosphate dehydrogenase with a predicted role in glycerol metabolism |
| AN6798 | Has domain(s) with predicted catalytic activity and role in metabolic process |
| AN6804 | Predicted transporter of the major facilitator superfamily (MFS) |
| AN6824 | Ortholog(s) have cytosol, nucleus localization |
| AN6825 | Component of the TRAPII complex that mediates Rab guanyl-nucleotide exchange factor activity, involved in Golgi vesicle-mediated transport |
| AN6835 | Putative cytochrome P450 |
| AN6841 | Ortholog(s) have endopeptidase activity, role in protein processing involved in protein targeting to mitochondrion and mitochondrial inner membrane peptidase complex localization |
| AN6843 | Mitochondrial ribosomal protein L4 |
| AN6853 | Ortholog(s) have phosphatidylinositol transporter activity |
| AN6856 | Ortholog of A. fumigatus Af293 : Afu5g13100, A. niger CBS 513.88 : An14g06050, A. oryzae RIB40 : AO090120000435, Aspergillus wentii : Aspwe1_0107169 and Aspergillus sydowii : Aspsy1_0040420 |
| AN6858 | Has domain(s) with predicted RNA polymerase II transcription factor activity, sequence-specific DNA binding, zinc ion binding activity, role in regulation of transcription, DNA-templated and nucleus localization |
| AN6863 | Member of kinesin-3 motor protein family |
| AN6869 | Ortholog(s) have endoplasmic reticulum localization |
| AN6872 | Ortholog of Aspergillus flavus NRRL 3357 : AFL2T_11606 |
| AN6881 | Ortholog of A. fumigatus Af293 : Afu8g07160, A. niger CBS 513.88 : An03g01630, Neosartorya fischeri NRRL 181 : NFIA_099950 and Aspergillus versicolor : Aspve1_0035247 |
| AN6882 | Ortholog(s) have oxaloacetase activity and role in oxalate metabolic process, oxaloacetate metabolic process |
| AN6883 | Has domain(s) with predicted role in transmembrane transport and integral component of membrane localization |
| AN6884 | Has domain(s) with predicted RNA polymerase II transcription factor activity, sequence-specific DNA binding, zinc ion binding activity, role in regulation of transcription, DNA-templated and nucleus localization |
| AN6886 | Protein involved in a signaling pathway that activates PacC transcription factor in response to alkaline ambient pH |
| AN6889 | Ortholog(s) have RNA polymerase II transcription factor activity, sequence-specific DNA binding, sequence-specific DNA binding activity |
| AN6894 | Putative peptidyl-prolyl cis-trans isomerase (PPIase) |
| AN6903 | Ortholog(s) have U1 snRNP, cytosol localization |
| AN6906 | Ortholog(s) have Prp19 complex, cytosol, nuclear envelope, spliceosomal complex localization |
| AN6917 | Ortholog of A. nidulans FGSC A4 : AN7927, A. niger CBS 513.88 : An12g02480, Aspergillus wentii : Aspwe1_0167675 and Aspergillus versicolor : Aspve1_0045429, Aspve1_0208268 |
| AN6918 | Has domain(s) with predicted oxidoreductase activity and role in metabolic process |
| AN6920 | Subunit of the endosomal sorting complex required for transport III (ESCRT-III) |
| AN6921 | Ortholog(s) have chaperone binding activity, role in negative regulation of DNA binding, positive regulation of telomere maintenance via telomerase, protein folding, regulation of telomerase activity and cytosol, nucleus localization |
| AN6936 | Putative 2-hydroxychromene-2-carboxylate isomerase |
| AN6937 | Ortholog of Aspergillus versicolor : Aspve1_0035147 and Aspergillus sydowii : Aspsy1_0585169 |
| AN6940 | Has domain(s) with predicted metal ion transmembrane transporter activity, role in metal ion transport, transmembrane transport and membrane localization |
| AN6955 | Has domain(s) with predicted catalytic activity, nucleoside-triphosphatase activity, nucleotide binding activity and role in nucleoside metabolic process |
| AN6967 | Predicted gypsy transposon-related ORF |
| AN6986 | Ortholog(s) have calcium:proton antiporter activity, potassium:proton antiporter activity, sodium:proton antiporter activity and role in hydrogen transport, potassium ion transport, sodium ion transport |
| AN6988 | Ortholog(s) have protein domain specific binding activity |
| AN6992 | Ortholog of Neosartorya fischeri NRRL 181 : NFIA_028800, Aspergillus wentii : Aspwe1_0043211, Aspergillus versicolor : Aspve1_0154813 and Aspergillus clavatus NRRL 1 : ACLA_053800 |
| AN7004 | Ortholog of A. fumigatus Af293 : Afu4g04450, A. niger CBS 513.88 : An14g00380, A. oryzae RIB40 : AO090206000048, Aspergillus wentii : Aspwe1_0043225 and Aspergillus sydowii : Aspsy1_0034669 |
| AN7005 | Ortholog of A. fumigatus Af293 : Afu4g04440, A. niger CBS 513.88 : An14g00390, A. oryzae RIB40 : AO090206000049, Aspergillus wentii : Aspwe1_0116246 and Aspergillus sydowii : Aspsy1_0158059 |
| AN7007 | Ortholog(s) have DNA binding activity |
| AN7010 | Ortholog(s) have role in chromatin silencing by small RNA and cytosol, nucleus localization |
| AN7011 | Ortholog of A. fumigatus Af293 : Afu4g04318, A. niger CBS 513.88 : An14g00530, Aspergillus wentii : Aspwe1_0053203, Aspergillus sydowii : Aspsy1_0184132 and Aspergillus terreus NIH2624 : ATET_02726 |
| AN7024 | protein of unknown function |
| AN7025 | Ortholog of A. fumigatus Af293 : Afu4g04220, A. oryzae RIB40 : AO090206000072, Aspergillus wentii : Aspwe1_0114911, Aspergillus sydowii : Aspsy1_0060635 and Aspergillus terreus NIH2624 : ATET_10114 |
| AN7032 | Class II chitin synthase with a role in chitin biosynthesis |
| AN7037 | Putative ESCRT II complex required for trafficking from the membrane to the vacuoles |
| AN7039 | Ortholog of A. fumigatus Af293 : Afu4g04080, A. niger CBS 513.88 : An14g00770, Neosartorya fischeri NRRL 181 : NFIA_029300, Aspergillus wentii : Aspwe1_0115799 and Aspergillus versicolor : Aspve1_0056350 |
| AN7041 | Signaling mucin involved in regulation of starvation response, adhesion and secretion of cellulase |
| AN7042 | Ortholog of A. fumigatus Af293 : Afu4g04060, A. niger CBS 513.88 : An14g00810, A. oryzae RIB40 : AO090206000108, Aspergillus wentii : Aspwe1_0043276 and Aspergillus sydowii : Aspsy1_0048943 |
| AN7043 | Ortholog(s) have holo-[acyl-carrier-protein] synthase activity and mitochondrion localization |
| AN7048 | Ortholog of A. fumigatus Af293 : Afu4g03980, A. niger CBS 513.88 : An14g00890, A. oryzae RIB40 : AO090011000319, Aspergillus wentii : Aspwe1_0115559 and Aspergillus sydowii : Aspsy1_0184047 |
| AN7060 | Ortholog(s) have monophenol monooxygenase activity |
| AN7062 | Ortholog of A. nidulans FGSC A4 : AN1643, AN1704, A. fumigatus Af293 : Afu2g14410, Afu4g01500 and A. niger CBS 513.88 : An18g01050, An04g02710, An09g01370 |
| AN7068 | Putative vanillyl alcohol oxidase |
| AN7070 | Beta-lactamase-type thioesterase |
| AN7098 | protein of unknown function |
| AN7099 | Ortholog of Aspergillus versicolor : Aspve1_0650099 and Aspergillus sydowii : Aspsy1_0089874 |
| AN7102 | Ortholog of A. fumigatus Af293 : Afu4g03830, A. niger CBS 513.88 : An14g01068, A. oryzae RIB40 : AO090011000343, Aspergillus wentii : Aspwe1_0030332 and Aspergillus sydowii : Aspsy1_0060599 |
| AN7103 | Protein with homology to Cockayne's syndrome nucleotide excision repair protein |
| AN7107 | Ortholog(s) have cell surface, nucleolus localization |
| AN7109 | protein of unknown function |
| AN7110 | Ortholog of Aspergillus versicolor : Aspve1_0139279, Aspve1_0834531 and Aspergillus sydowii : Aspsy1_0091735 |
| AN7119 | Has domain(s) with predicted substrate-specific transmembrane transporter activity, transmembrane transporter activity, role in transmembrane transport and integral component of membrane, membrane localization |
| AN7133 | protein of unknown function |
| AN7142 | Putative secreted acid phosphatase with a predicted role in gluconic acid and gluconate metabolism |
| AN7149 | Ortholog(s) have role in nucleobase-containing compound transport and plasma membrane localization |
| AN7157 | protein of unknown function |
| AN7165 | Ortholog of A. fumigatus Af293 : Afu4g03330, A. niger CBS 513.88 : An14g01960, A. oryzae RIB40 : AO090011000165, Aspergillus wentii : Aspwe1_0053298 and Aspergillus sydowii : Aspsy1_0060563 |
| AN7166 | Ortholog of A. nidulans FGSC A4 : AN3215, AN8609, A. fumigatus Af293 : Afu3g14210, Afu4g03360, Afu4g12510 and A. niger CBS 513.88 : An09g04200, An14g01840 |
| AN7167 | Has domain(s) with predicted role in transmembrane transport and integral component of membrane localization |
| AN7169 | NirA-dependent flavohemoprotein |
| AN7170 | protein of unknown function |
| AN7172 | Ortholog of A. niger CBS 513.88 : An14g02030, A. oryzae RIB40 : AO090011000125, Aspergillus wentii : Aspwe1_0030391, Aspergillus clavatus NRRL 1 : ACLA_055220 and Aspergillus niger ATCC 1015 : 41626-mRNA |
| AN7173 | Has domain(s) with predicted role in transmembrane transport and integral component of membrane localization |
| AN7174 | Has domain(s) with predicted DNA binding, chromatin binding activity |
| AN7180 | Protein with cutinase activity, involved in carbohydrate catabolism |
| AN7181 | Ortholog of A. fumigatus Af293 : Afu4g03200, A. oryzae RIB40 : AO090011000112, Neosartorya fischeri NRRL 181 : NFIA_030260 and Aspergillus versicolor : Aspve1_0140969 |
| AN7183 | protein of unknown function |
| AN7185 | Putative protein serine/threonine kinase |
| AN7186 | Ortholog of A. fumigatus Af293 : Afu5g14180, A. niger CBS 513.88 : An14g05580, A. oryzae RIB40 : AO090001000217, Aspergillus wentii : Aspwe1_0179974 and Aspergillus sydowii : Aspsy1_0026595 |
| AN7194 | Has domain(s) with predicted oxidoreductase activity, zinc ion binding activity and role in oxidation-reduction process |
| AN7201 | Ortholog(s) have role in proteolysis |
| AN7202 | Has domain(s) with predicted catalytic activity and role in metabolic process |
| AN7204 | Putative oleoyl-delta12 desaturase |
| AN7205 | Ortholog(s) have unfolded protein binding activity, role in ribosome biogenesis and cytosol, nucleolus localization |
| AN7206 | Ortholog(s) have GTPase activity, role in cellular protein localization, exit from mitosis, mitotic M phase, positive regulation of barrier septum assembly, septation initiation signaling and mitotic spindle pole body localization |
| AN7208 | SET domain protein |
| AN7219 | protein of unknown function |
| AN7231 | Ortholog(s) have serine-type endopeptidase activity and role in proteolysis |
| AN7232 | Ortholog of A. nidulans FGSC A4 : AN6413, AN6419, AN6946, AN5312, AN5664, AN8328, AN3241, AN10369, AN1930, AN2386, AN1738, AN11159, AN8984, AN8951, AN1540, AN8971, AN9387, AN9266, AN9306, AN1317, AN10886, AN7774, AN12202 |
| AN7233 | Putative epoxide hydrolase |
| AN7237 | Has domain(s) with predicted catalytic activity and role in metabolic process |
| AN7238 | Has domain(s) with predicted 3-hydroxyacyl-CoA dehydrogenase activity, coenzyme binding, oxidoreductase activity, acting on the CH-OH group of donors, NAD or NADP as acceptor activity |
| AN7242 | Has domain(s) with predicted role in transmembrane transport and membrane localization |
| AN7245 | Ortholog of A. niger CBS 513.88 : An01g06570, An06g00030, A. oryzae RIB40 : AO090023000010, AO090026000177, Neosartorya fischeri NRRL 181 : NFIA_055760 and Aspergillus wentii : Aspwe1_0032389, Aspwe1_0041672 |
| AN7271 | protein of unknown function |
| AN7273 | Ortholog of A. fumigatus Af293 : Afu2g16990, A. oryzae RIB40 : AO090102000119, Aspergillus wentii : Aspwe1_0182487, Aspergillus sydowii : Aspsy1_0027170 and Aspergillus terreus NIH2624 : ATET_10043 |
| AN7296 | Essential protein involved in progression through mitosis |
| AN7300 | Has domain(s) with predicted zinc ion binding activity |
| AN7315 | Putative succinate-semialdehyde dehydrogenase [NAD(P)+] with a predicted role in the 4-aminobutyrate (GABA) shunt |
| AN7319 | protein of unknown function |
| AN7322 | Ortholog of A. niger CBS 513.88 : An01g14820, Aspergillus wentii : Aspwe1_0093592, Aspergillus versicolor : Aspve1_0038743 and Aspergillus niger ATCC 1015 : 172038-mRNA |
| AN7323 | Covalently-bound cell wall protein |
| AN7327 | Predicted glycosylphosphatidylinositol (GPI)-anchored protein |
| AN7328 | Ortholog of A. fumigatus Af293 : Afu2g16550, A. niger CBS 513.88 : An03g03580, A. oryzae RIB40 : AO090102000183, Aspergillus wentii : Aspwe1_0039308 and Aspergillus sydowii : Aspsy1_0140287 |
| AN7335 | Ortholog of A. fumigatus Af293 : Afu2g16510, A. niger CBS 513.88 : An15g06990, A. oryzae RIB40 : AO090102000205, Aspergillus wentii : Aspwe1_0460265 and Aspergillus sydowii : Aspsy1_0169307 |
| AN7336 | Ortholog of A. fumigatus Af293 : Afu2g16500, A. niger CBS 513.88 : An15g06925, Aspergillus wentii : Aspwe1_0026944, Aspergillus sydowii : Aspsy1_0054451 and Aspergillus terreus NIH2624 : ATET_02290 |
| AN7347 | Ortholog of A. fumigatus Af293 : Afu2g16410, Aspergillus wentii : Aspwe1_0039276, Aspergillus versicolor : Aspve1_0038665 and Aspergillus clavatus NRRL 1 : ACLA_075330 |
| AN7348 | Ortholog of A. fumigatus Af293 : Afu2g16420, A. niger CBS 513.88 : An15g06820, A. oryzae RIB40 : AO090102000221, Aspergillus wentii : Aspwe1_0108539 and Aspergillus sydowii : Aspsy1_0027034 |
| AN7350 | Has domain(s) with predicted nucleic acid binding, nucleotide binding activity |
| AN7370 | Ortholog of A. nidulans FGSC A4 : AN3519, AN2646, AN0587, AN0866 and A. fumigatus Af293 : Afu1g15200, Afu3g03790, Afu4g14040, Afu5g06900, Afu7g08575 |
| AN7373 | Has domain(s) with predicted transporter activity, role in transmembrane transport, transport and membrane localization |
| AN7384 | Ortholog of Aspergillus versicolor : Aspve1_0148542 |
| AN7387 | Putative pyrroline-5-carboxylate reductase with a predicted role in proline metabolism |
| AN7395 | Ortholog of A. nidulans FGSC A4 : AN5639, AN2587, AN9444, A. fumigatus Af293 : Afu5g00840, A. niger CBS 513.88 : An03g01000 and A. oryzae RIB40 : AO090102000018 |
| AN7400 | Ortholog of Aspergillus versicolor : Aspve1_0037710 and Aspergillus sydowii : Aspsy1_0038876 |
| AN7403 | Ortholog of A. nidulans FGSC A4 : AN6621, A. fumigatus Af293 : Afu8g01810, A. niger CBS 513.88 : An05g02280, A. oryzae RIB40 : AO090026000198 and Aspergillus wentii : Aspwe1_0151553, Aspwe1_0177691 |
| AN7404 | Ortholog of A. fumigatus Af293 : Afu3g01280, A. niger CBS 513.88 : An02g12500, An16g05960, An09g03800, A. oryzae RIB40 : AO090011000171 and Aspergillus wentii : Aspwe1_0036364, Aspwe1_0041299, Aspwe1_0112391 |
| AN7410 | protein of unknown function |
| AN7413 | Putative endo-beta-(1,4)-mannanase with predicted role in degradation of mannans |
| AN7422 | putative ubiquitin carboxyl-terminal hydrolase |
| AN7423 | Putative replication protein |
| AN7430 | Putative glutamine amidotransferase with a predicted role in histidine metabolism |
| AN7431 | Ortholog(s) have nucleus localization |
| AN7432 | Has domain(s) with predicted ornithine decarboxylase inhibitor activity |
| AN7442 | protein of unknown function |
| AN7443 | Ortholog of A. fumigatus Af293 : Afu2g06100, A. niger CBS 513.88 : An02g14710, A. oryzae RIB40 : AO090001000726, Aspergillus wentii : Aspwe1_0110234 and Aspergillus sydowii : Aspsy1_0152786 |
| AN7444 | Ortholog(s) have cytosol, nucleus localization |
| AN7447 | Ortholog(s) have role in mRNA cis splicing, via spliceosome and U4/U6 x U5 tri-snRNP complex localization |
| AN7455 | Has domain(s) with predicted zinc ion binding activity |
| AN7456 | Ortholog(s) have U2 snRNP localization |
| AN7464 | Putative calcium-transporting ATPase with a predicted role in energy metabolism |
| AN7469 | Ortholog(s) have riboflavin kinase activity, zinc ion binding activity, role in FMN biosynthetic process, riboflavin metabolic process and cytosol, mitochondrial inner membrane, nucleus localization |
| AN7470 | Ortholog of A. fumigatus Af293 : Afu2g05810, A. niger CBS 513.88 : An02g14540, A. oryzae RIB40 : AO090001000700, Neosartorya fischeri NRRL 181 : NFIA_082530 and Aspergillus wentii : Aspwe1_0120676 |
| AN7471 | Ortholog(s) have cytoplasm localization |
| AN7477 | Ortholog of A. niger CBS 513.88 : An02g14040, Aspergillus versicolor : Aspve1_0044036, Aspergillus carbonarius ITEM 5010 : Acar5010_211768 and Aspergillus brasiliensis : Aspbr1_0191709 |
| AN7481 | Ortholog of A. fumigatus Af293 : Afu2g05680, A. niger CBS 513.88 : An02g14110, A. oryzae RIB40 : AO090001000682, Aspergillus wentii : Aspwe1_0055195 and Aspergillus sydowii : Aspsy1_0153107 |
| AN7482 | Ortholog of A. fumigatus Af293 : Afu2g05690, A. oryzae RIB40 : AO090001000684, Aspergillus wentii : Aspwe1_0055196, Aspergillus sydowii : Aspsy1_0152978 and Aspergillus terreus NIH2624 : ATET_06759 |
| AN7500 | Putative NADH dehydrogenase (ubiquinone) with a predicted role in energy metabolism |
| AN7516 | Predicted DDE1 transposon-related ORF |
| AN7517 | Ortholog of A. fumigatus Af293 : Afu5g03330, A. niger CBS 513.88 : An09g05650, A. oryzae RIB40 : AO090102000538, Aspergillus wentii : Aspwe1_0043027 and Aspergillus sydowii : Aspsy1_0146077 |
| AN7521 | Ortholog of A. fumigatus Af293 : Afu3g09230, A. niger CBS 513.88 : An12g05590, A. oryzae RIB40 : AO090023000559, Aspergillus wentii : Aspwe1_0110954 and Aspergillus sydowii : Aspsy1_1178405 |
| AN7524 | Ortholog(s) have mitochondrion localization |
| AN7543 | Ortholog of A. fumigatus Af293 : Afu2g14690, A. oryzae RIB40 : AO090026000808, Aspergillus wentii : Aspwe1_0119824 and Aspergillus sydowii : Aspsy1_0045967 |
| AN7545 | Component of AnCP/AnCF CCAAT-binding complex |
| AN7547 | Member of kinesin-3 motor protein family |
| AN7555 | protein of unknown function |
| AN7559 | Has domain(s) with predicted role in transcription initiation from RNA polymerase II promoter and transcription factor TFIID complex localization |
| AN7560 | Protein involved in a signaling pathway that activates PacC transcription factor in response to alkaline ambient pH |
| AN7562 | Ortholog(s) have alpha-1,6-mannosyltransferase activity and role in ascospore formation, barrier septum assembly, cell wall mannoprotein biosynthetic process, protein N-linked glycosylation |
| AN7564 | Putative threonine aldolase with a predicted role in glycine, serine, and threonine metabolism |
| AN7565 | Ortholog of A. fumigatus Af293 : Afu2g14940, A. niger CBS 513.88 : An15g03240, A. oryzae RIB40 : AO090012000409, Aspergillus wentii : Aspwe1_0693532 and Aspergillus sydowii : Aspsy1_1021602 |
| AN7569 | Ortholog(s) have thiamine transmembrane transporter activity, role in thiamine pyrophosphate transport and integral component of mitochondrial inner membrane localization |
| AN7575 | Putative C-3 sterol dehydrogenase with a predicted role in sterol metabolism |
| AN7584 | Has domain(s) with predicted DNA binding, zinc ion binding activity, role in transcription, DNA-templated and nucleus localization |
| AN7585 | Ortholog of Aspergillus carbonarius ITEM 5010 : Acar5010_049247, Acar5010_049992 |
| AN7587 | Ortholog of A. fumigatus Af293 : Afu2g15180, A. niger CBS 513.88 : An09g03440, A. oryzae RIB40 : AO090012000301, Aspergillus wentii : Aspwe1_0054076 and Aspergillus sydowii : Aspsy1_0058595 |
| AN7594 | DUF636 domain-containing protein |
| AN7600 | Ortholog(s) have role in hydrogen sulfide biosynthetic process, sulfate assimilation, sulfur amino acid biosynthetic process and cytosol localization |
| AN7605 | Ortholog of A. fumigatus Af293 : Afu2g15530, A. oryzae RIB40 : AO090012000278, Aspergillus wentii : Aspwe1_0026836 and Aspergillus sydowii : Aspsy1_0032147 |
| AN7617 | Ortholog of A. fumigatus Af293 : Afu4g00235, A. niger CBS 513.88 : An16g01350, Neosartorya fischeri NRRL 181 : NFIA_045390, Aspergillus wentii : Aspwe1_0160305 and Aspergillus versicolor : Aspve1_0054503 |
| AN7628 | Putative deadenylase with a predicted role in RNA processing |
| AN7633 | Putative malate dehydrogenase |
| AN7634 | Has domain(s) with predicted catalytic activity, sulfuric ester hydrolase activity and role in metabolic process |
| AN7635 | Transcript induced by exposure of A. nidulans to bafilomycin B1 |
| AN7640 | Ortholog of A. nidulans FGSC A4 : AN5292, A. fumigatus Af293 : Afu5g12490, Afu5g13780, A. niger CBS 513.88 : An14g06730 and A. oryzae RIB40 : AO090120000374, AO090020000661 |
| AN7641 | Putative copper amine oxidase |
| AN7651 | Ortholog of A. fumigatus Af293 : Afu2g01080, A. niger CBS 513.88 : An10g00470, A. oryzae RIB40 : AO090701000377, Aspergillus wentii : Aspwe1_0113772 and Aspergillus sydowii : Aspsy1_1029216 |
| AN7662 | Putative metalloreductase with a predicted role in iron homeostasis |
| AN7671 | Ortholog of A. fumigatus Af293 : Afu2g01440, A. niger CBS 513.88 : An03g05020, A. oryzae RIB40 : AO090701000815, Neosartorya fischeri NRRL 181 : NFIA_033740 and Aspergillus clavatus NRRL 1 : ACLA_093470 |
| AN7672 | Ortholog(s) have alpha-1,6-mannosyltransferase activity, mannosyltransferase activity |
| AN7676 | Has domain(s) with predicted ATP binding, ATPase activity, ATPase activity, coupled to transmembrane movement of substances, nucleoside-triphosphatase activity, nucleotide binding activity and role in transmembrane transport |
| AN7681 | Ortholog(s) have role in negative regulation of transcription from RNA polymerase III promoter, transcription factor TFIIIB complex assembly |
| AN7699 | Ortholog(s) have mitochondrial ribosome localization |
| AN7700 | Ortholog(s) have role in regulation of fungal-type cell wall biogenesis and cytoplasm localization |
| AN7709 | Ortholog(s) have transferase activity, transferring pentosyl groups activity, role in charged-tRNA amino acid modification and cytosol, nucleus localization |
| AN7710 | Ortholog(s) have intracellular localization |
| AN7717 | Ortholog of A. fumigatus Af293 : Afu5g08190, A. oryzae RIB40 : AO090701000736, Aspergillus wentii : Aspwe1_0101496, Aspergillus sydowii : Aspsy1_0046260 and Aspergillus terreus NIH2624 : ATET_08307 |
| AN7719 | Ortholog(s) have cytosol, nucleus localization |
| AN7723 | Ortholog(s) have DNA replication origin binding, chromatin binding activity |
| AN7726 | Putative nuclear pore-associated protein with homology to Saccharomyces cerevisiae Sac3p |
| AN7729 | Ortholog(s) have bilirubin transmembrane transporter activity, glutathione S-conjugate-exporting ATPase activity, phytochelatin transmembrane transporter activity |
| AN7739 | Has domain(s) with predicted chromatin binding activity |
| AN7741 | Ortholog(s) have cell division site, cytosol, mitotic spindle pole body, nucleus localization |
| AN7753 | ATP-dependent DNA helicase II |
| AN7758 | Ortholog of A. niger CBS 513.88 : An13g01890, A. oryzae RIB40 : AO090701000676, Neosartorya fischeri NRRL 181 : NFIA_079570 and Aspergillus wentii : Aspwe1_0035088 |
| AN7762 | Ortholog of A. fumigatus Af293 : Afu5g07640, A. oryzae RIB40 : AO090701000669, Neosartorya fischeri NRRL 181 : NFIA_079620, Aspergillus wentii : Aspwe1_0101081 and Aspergillus versicolor : Aspve1_0131565 |
| AN7764 | Has domain(s) with predicted ligase activity |
| AN7766 | Ortholog(s) have role in cellular response to biotic stimulus, cellular response to starvation and filamentous growth of a population of unicellular organisms in response to biotic stimulus, more |
| AN7767 | protein of unknown function |
| AN7773 | Putative cytochrome P450 |
| AN7775 | Has domain(s) with predicted role in response to stress and integral component of membrane localization |
| AN7776 | Has domain(s) with predicted RNA polymerase II transcription factor activity, sequence-specific DNA binding, zinc ion binding activity, role in regulation of transcription, DNA-templated and nucleus localization |
| AN7785 | Ortholog of A. nidulans FGSC A4 : AN5372, A. fumigatus Af293 : Afu5g07450, Afu6g14060 and A. niger CBS 513.88 : An08g08280, An15g02880, An12g10360 |
| AN7786 | Ortholog of A. fumigatus Af293 : Afu6g00600, A. oryzae RIB40 : AO090663000003, AO090113000133 and Aspergillus wentii : Aspwe1_0041313, Aspwe1_0117236 |
| AN7788 | Has domain(s) with predicted DNA binding, zinc ion binding activity, role in transcription, DNA-templated and nucleus localization |
| AN7789 | Has domain(s) with predicted aminopeptidase activity, dipeptidyl-peptidase activity, hydrolase activity and role in metabolic process, proteolysis |
| AN7792 | Putative lysophosphoplipase A |
| AN7812 | Putative versicolorin B synthase with a predicted role in sterigmatocystin/aflatoxin biosynthesis |
| AN7848 | protein of unknown function |
| AN7858 | Has domain(s) with predicted aspartic-type endopeptidase activity and role in proteolysis |
| AN7863 | protein of unknown function |
| AN7866 | Ortholog(s) have role in fumiquinazoline C biosynthetic process |
| AN7875 | protein of unknown function |
| AN7887 | protein of unknown function |
| AN7889 | Ortholog of A. nidulans FGSC A4 : AN4657, A. fumigatus Af293 : Afu1g00920, Afu2g02370, Afu2g05115, Afu3g06425 and A. niger CBS 513.88 : An07g04250, An05g02120, An01g11840, An12g06960, An03g03340 |
| AN7892 | Small heat-shock protein |
| AN7893 | Has domain(s) with predicted oxidoreductase activity, acting on paired donors, with incorporation or reduction of molecular oxygen, 2-oxoglutarate as one donor, and incorporation of one atom each of oxygen into both donors activity |
| AN7896 | Zn(II)2Cys6 transcription factor with a role in secondary metabolite biosynthesis |
| AN7898 | Major facilitator superfamily (MFS) transporter with a role in secondary metabolism |
| AN7902 | FAD-binding monooxygenase with a role in secondary metabolism |
| AN7903 | Polyketide synthase |
| AN7926 | Has domain(s) with predicted oxidoreductase activity and role in metabolic process |
| AN7950 | Putative glucan endo-1,3-beta-D-glucosidase with predicted role in degradation of glucans |
| AN7951 | Has domain(s) with predicted DNA binding, RNA polymerase II transcription factor activity, sequence-specific DNA binding, zinc ion binding activity and role in regulation of transcription, DNA-templated, transcription, DNA-templated |
| AN7968 | Predicted mariner transposon-related ORF |
| AN7981 | Has domain(s) with predicted oxidoreductase activity and role in oxidation-reduction process |
| AN7986 | Has domain(s) with predicted ATP binding, protein kinase activity, protein tyrosine kinase activity and role in protein phosphorylation |
| AN7995 | Putative ribokinase with a predicted role in ribose metabolism |
| AN8000 | Subunit of the SAGA transcriptional regulatory complex, possible pseudogene |
| AN8006 | Hydrophobin, protein of the conidium wall responsible for hydrophobicity of conidium surface |
| AN8008 | Ortholog of Aspergillus versicolor : Aspve1_0092890 and Aspergillus sydowii : Aspsy1_0059936 |
| AN8017 | Ortholog(s) have cytosol, mitotic spindle pole body, nucleus localization |
| AN8020 | Putative protein of unknown function |
| AN8021 | Putative vacuolar ATPase (V-ATPase), subunit A |
| AN8029 | Putative plasma membrane high affinity K+ transporter |
| AN8030 | Has domain(s) with predicted NAD binding, oxidoreductase activity, acting on the CH-OH group of donors, NAD or NADP as acceptor activity and role in oxidation-reduction process |
| AN8031 | Ortholog of A. fumigatus Af293 : Afu5g02250, A. niger CBS 513.88 : An02g10580, A. oryzae RIB40 : AO090102000288, Aspergillus wentii : Aspwe1_0042830 and Aspergillus sydowii : Aspsy1_0156829 |
| AN8039 | Histone variant H2A.Z |
| AN8044 | Ortholog(s) have metalloendopeptidase activity |
| AN8053 | Ortholog(s) have cell division site, cell tip, cytosol localization |
| AN8056 | Ortholog(s) have ATPase activity, DNA/DNA annealing activity, role in mitotic chromosome condensation and cytosol, nuclear condensin complex localization |
| AN8058 | Has domain(s) with predicted electron carrier activity, metal ion binding, molybdenum ion binding, oxidoreductase activity and role in nitrate assimilation, oxidation-reduction process |
| AN8060 | Has domain(s) with predicted NAD binding, oxidoreductase activity, acting on the aldehyde or oxo group of donors, NAD or NADP as acceptor activity and role in cellular amino acid metabolic process, oxidation-reduction process |
| AN8063 | Putative acid phosphatase |
| AN8065 | Ortholog(s) have role in Arp2/3 complex-mediated actin nucleation, actin cortical patch localization, cellular response to drug, endocytosis and establishment of mitochondrion localization, more |
| AN8067 | Ortholog of A. fumigatus Af293 : Afu5g01840, A. niger CBS 513.88 : An16g01550, A. oryzae RIB40 : AO090003001340, Aspergillus wentii : Aspwe1_0042768 and Aspergillus sydowii : Aspsy1_0059852 |
| AN8069 | Has domain(s) with predicted membrane localization |
| AN8073 | Ortholog(s) have nucleus localization |
| AN8078 | Phenylacetate 2-hydroxylase |
| AN8095 | Has domain(s) with predicted role in transmembrane transport and integral component of membrane localization |
| AN8099 | Putative oxalate decarboxylase with a predicted role in oxalic acid metabolism |
| AN8103 | putative transcription factor |
| AN8104 | Has domain(s) with predicted role in response to stress and integral component of membrane localization |
| AN8108 | Predicted fumarylacetoacetase |
| AN8111 | Putative Zn(II)2Cys6 transcription factor |
| AN8112 | Predicted ransmembrane transporter |
| AN8113 | Putative oxidoreductase with a predicted role in carbohydrate metabolism |
| AN8121 | Putative 5'-phosphoribosylformyl glycinamidine synthetase with a predicted role in purine metabolism |
| AN8122 | Ortholog(s) have role in cellular response to drug, hexose transport, pathogenesis |
| AN8137 | Has domain(s) with predicted alcohol O-acetyltransferase activity and role in alcohol metabolic process |
| AN8138 | Alpha-galactosidase, involved in degradation of mannans |
| AN8152 | Has domain(s) with predicted UDP-N-acetylmuramate dehydrogenase activity, flavin adenine dinucleotide binding, oxidoreductase activity and role in oxidation-reduction process |
| AN8153 | Has domain(s) with predicted ATP binding, nucleoside-triphosphatase activity, nucleotide binding activity |
| AN8159 | Predicted DDE1 transposon-related ORF |
| AN8162 | Ortholog of A. nidulans FGSC A4 : AN7214, A. fumigatus Af293 : Afu5g01210, A. niger CBS 513.88 : An16g00660 and A. oryzae RIB40 : AO090102000383, AO090120000014, AO090001000406, AO090103000238 |
| AN8163 | Putative short-chain dehydrogenase/reductase |
| AN8168 | Regulatory protein involved in nitrogen metabolite repression |
| AN8173 | Ortholog(s) have actin monomer binding, protein kinase inhibitor activity, ribosome binding activity, role in negative regulation of protein phosphorylation and cytoplasm, nucleus, polysome, ribosome localization |
| AN8180 | Ortholog(s) have role in mRNA cis splicing, via spliceosome and Prp19 complex, mitotic spindle pole body, spliceosomal complex localization |
| AN8183 | Putative ribosomal RNA processing complex subunit |
| AN8190 | Predicted protein serine/threonine kinase |
| AN8205 | Has domain(s) with predicted DNA binding activity, role in telomere maintenance and nuclear chromosome, telomeric region localization |
| AN8206 | Ortholog(s) have mitochondrion localization |
| AN8211 | Ortholog(s) have histone demethylase activity (H3-trimethyl-K4 specific) activity |
| AN8213 | Putative dTMP kinase with a predicted role in pyrimidine metabolism |
| AN8215 | Putative methylenetetrahydrofolate reductase (NADPH) with a predicted role in one-carbon metabolism |
| AN8234 | Has domain(s) with predicted protein phosphatase inhibitor activity and role in regulation of phosphoprotein phosphatase activity, regulation of signal transduction |
| AN8240 | Ortholog of Aspergillus glaucus : Aspgl1_0050060, Aspergillus versicolor : Aspve1_0142948, Aspergillus sydowii : Aspsy1_0512447 and Aspergillus aculeatus ATCC16872 : Aacu16872_044627 |
| AN8241 | Endochitinase with a predicted role in chitin hydrolysis |
| AN8244 | Coiled-coil protein associated with spindle pole body, involved in septation |
| AN8246 | Putative signal recognition particle protein |
| AN8247 | Ortholog(s) have role in maturation of 5.8S rRNA from tricistronic rRNA transcript (SSU-rRNA, 5.8S rRNA, LSU-rRNA), maturation of LSU-rRNA from tricistronic rRNA transcript (SSU-rRNA, 5.8S rRNA, LSU-rRNA) |
| AN8254 | Putative snoRNP complex protein |
| AN8268 | Putative RasGAP SH3 binding protein, contains NTF2 and RRM domains |
| AN8279 | Ortholog of S. cerevisiae Can1p which has arginine transmembrane transporter activity |
| AN8298 | Has domain(s) with predicted DNA binding, RNA polymerase II transcription factor activity, sequence-specific DNA binding, zinc ion binding activity and role in regulation of transcription, DNA-templated, transcription, DNA-templated |
| AN8301 | protein of unknown function |
| AN8309 | Putative cytochrome P450 |
| AN8323 | protein of unknown function |
| AN8329 | Putative glucose oxidase-related protein |
| AN8330 | Has domain(s) with predicted oxidoreductase activity, transferase activity, transferring acyl groups other than amino-acyl groups, zinc ion binding activity and role in oxidation-reduction process |
| AN8332 | protein of unknown function |
| AN8346 | Ortholog(s) have FAD binding, sulfide:quinone oxidoreductase activity |
| AN8347 | Ortholog(s) have role in cellobiose transport, cellulose catabolic process |
| AN8355 | Has domain(s) with predicted DNA binding, RNA polymerase II transcription factor activity, sequence-specific DNA binding, zinc ion binding activity and role in regulation of transcription, DNA-templated, transcription, DNA-templated |
| AN8358 | Putative cytochrome P450 |
| AN8360 | Has domain(s) with predicted oxidoreductase activity |
| AN8366 | Has domain(s) with predicted role in transmembrane transport and integral component of membrane localization |
| AN8382 | protein of unknown function |
| AN8388 | Has domain(s) with predicted catalytic activity and role in nucleoside metabolic process |
| AN8397 | Has domain(s) with predicted oxidoreductase activity, acting on CH-OH group of donors activity and role in oxidation-reduction process |
| AN8406 | Putative alcohol dehydrogenase |
| AN8407 | Ortholog of Aspergillus brasiliensis : Aspbr1_0177798, Aspergillus glaucus : Aspgl1_0030152, Aspergillus wentii : Aspwe1_0181982 and Aspergillus versicolor : Aspve1_0046756 |
| AN8412 | Putative hybrid polyketide synthase-nonribosomal peptide synthase (PKS-NRPS) |
| AN8416 | Protein with homology to the Saccharomyces cerevisiae uracil transporter Fur4p |
| AN8421 | Putative endo-mannanase GH76 family protein |
| AN8424 | Ortholog of A. nidulans FGSC A4 : AN3992, AN7089, A. fumigatus Af293 : Afu7g05085, A. oryzae RIB40 : AO090005000321 and Neosartorya fischeri NRRL 181 : NFIA_026200 |
| AN8430 | Ortholog of A. fumigatus Af293 : Afu5g12780, A. niger CBS 513.88 : An18g00330, A. oryzae RIB40 : AO090010000134, Neosartorya fischeri NRRL 181 : NFIA_074390 and Aspergillus wentii : Aspwe1_0449405 |
| AN8433 | Has domain(s) with predicted catalytic activity and role in metabolic process |
| AN8446 | Has domain(s) with predicted oxidoreductase activity and role in oxidation-reduction process |
| AN8448 | Has domain(s) with predicted substrate-specific transmembrane transporter activity, transmembrane transporter activity, role in transmembrane transport and integral component of membrane, membrane localization |
| AN8456 | Ortholog of A. nidulans FGSC A4 : AN2381 and Neosartorya fischeri NRRL 181 : NFIA_099730 |
| AN8459 | Ortholog(s) have role in arginine transport, gliotoxin biosynthetic process and plasma membrane localization |
| AN8460 | Has domain(s) with predicted DNA binding, RNA polymerase II transcription factor activity, sequence-specific DNA binding, zinc ion binding activity and role in regulation of transcription, DNA-templated, transcription, DNA-templated |
| AN8475 | Ortholog of A. niger CBS 513.88 : An13g02250, An04g03090, A. oryzae RIB40 : AO090023000363, AO090012000682, Aspergillus wentii : Aspwe1_0053209 and Aspergillus versicolor : Aspve1_0155359 |
| AN8496 | Has domain(s) with predicted metal ion binding activity |
| AN8497 | Has domain(s) with predicted carbon-sulfur lyase activity and role in metabolic process |
| AN8499 | Ortholog(s) have cytoplasm localization |
| AN8503 | Ortholog of A. niger CBS 513.88 : An13g03080, Aspergillus wentii : Aspwe1_0177501, Aspergillus versicolor : Aspve1_0046851 and Aspergillus sydowii : Aspsy1_0159981 |
| AN8505 | Ortholog of Aspergillus brasiliensis : Aspbr1_0111846, Aspergillus fumigatus A1163 : AFUB_075260, Aspergillus niger ATCC 1015 : 199457-mRNA and Aspergillus aculeatus ATCC16872 : Aacu16872_050877 |
| AN8507 | Ortholog(s) have role in secondary metabolite biosynthetic process |
| AN8511 | Putative branched chain amino acid aminotransferase with a predicted role in branched chain amino acid biosynthesis |
| AN8512 | protein of unknown function |
| AN8516 | Putative aminotransferase |
| AN8520 | Protein required for terrequinone A biosynthesis |
| AN8521 | Gene adjacent to tdi (terrequinone biosynthesis) gene cluster |
| AN8525 | Has domain(s) with predicted oxidoreductase activity and role in oxidation-reduction process |
| AN8532 | Predicted glycosylphosphatidylinositol (GPI)-anchored protein |
| AN8538 | Ortholog of A. niger CBS 513.88 : An12g09900, A. oryzae RIB40 : AO090003001519, Aspergillus versicolor : Aspve1_0178418 and Aspergillus niger ATCC 1015 : 181097-mRNA, 194913-mRNA |
| AN8544 | Ortholog of Aspergillus versicolor : Aspve1_0033877 and Aspergillus sydowii : Aspsy1_0466900 |
| AN8548 | Ortholog of A. nidulans FGSC A4 : AN6924, AN5943, AN8512, AN0857, AN8661, AN4642 and A. fumigatus Af293 : Afu1g15290, Afu3g00850, Afu4g08850, Afu7g00920 |
| AN8549 | protein of unknown function |
| AN8551 | Putative transferase with a predicted role in the pentose-phosphate shunt or xylulose metabolism |
| AN8555 | protein of unknown function |
| AN8557 | protein of unknown function |
| AN8558 | protein of unknown function |
| AN8565 | Putative serine O-acetyltransferase with a predicted role in cysteine metabolism |
| AN8566 | Has domain(s) with predicted catalytic activity, catechol 1,2-dioxygenase activity, ferric iron binding, iron ion binding and oxidoreductase activity, more |
| AN8569 | Has domain(s) with predicted O-methyltransferase activity |
| AN8570 | protein of unknown function |
| AN8586 | protein of unknown function |
| AN8590 | Has domain(s) with predicted DNA binding, RNA polymerase II transcription factor activity, sequence-specific DNA binding, zinc ion binding activity and role in regulation of transcription, DNA-templated, transcription, DNA-templated |
| AN8593 | Ortholog of A. fumigatus Af293 : Afu3g02790, Afu4g00460, A. niger CBS 513.88 : An11g01410, A. oryzae RIB40 : AO090009000455, AO090138000169 and Neosartorya fischeri NRRL 181 : NFIA_045160 |
| AN8607 | Ortholog of A. nidulans FGSC A4 : AN1754, A. fumigatus Af293 : Afu3g02050, A. niger CBS 513.88 : An11g06690, An18g01930 and A. oryzae RIB40 : AO090001000144 |
| AN8616 | Component of the plasma membrane-associated VapA-VipC-VapB methyltransferase complex that controls differentiation |
| AN8623 | Has domain(s) with predicted role in transmembrane transport and integral component of membrane localization |
| AN8624 | protein of unknown function |
| AN8625 | Ortholog of A. nidulans FGSC A4 : AN8376, AN7265 and A. fumigatus Af293 : Afu6g00680, Afu8g06420 |
| AN8627 | Has domain(s) with predicted transferase activity, transferring glycosyl groups activity and membrane localization |
| AN8628 | Has domain(s) with predicted oxidoreductase activity, transferase activity, transferring acyl groups other than amino-acyl groups, zinc ion binding activity and role in oxidation-reduction process |
| AN8639 | Putative alpha,alpha-trehalose-phosphate synthase (UDP-forming) with a predicted role in trehalose biosynthesis |
| AN8642 | Has domain(s) with predicted oxidoreductase activity |
| AN8643 | Ortholog(s) have role in cellular response to heat, cellular response to hydrogen peroxide |
| AN8645 | Ortholog(s) have role in positive regulation of secondary metabolite biosynthetic process, secondary metabolite biosynthetic process |
| AN8649 | Ortholog of A. fumigatus Af293 : Afu1g04100, A. niger CBS 513.88 : An01g02930, A. oryzae RIB40 : AO090009000519 and Aspergillus wentii : Aspwe1_0037325, Aspwe1_0104267 |
| AN8652 | protein of unknown function |
| AN8654 | Putative aminomethyltransferase with a predicted role in glycine, serine, and threonine metabolism |
| AN8657 | Has domain(s) with predicted oxidoreductase activity and role in oxidation-reduction process |
| AN8671 | Ortholog(s) have mRNA binding, protein domain specific binding activity |
| AN8677 | Galactofuranosyltransferase involved in biosynthesis of galactofuranose antigen of cell wall O-glycan |
| AN8687 | Ortholog(s) have cytosol localization |
| AN8688 | ORF that was absent from the original release of version 4 of the A. nidulans annotation, but present in a previous version |
| AN8689 | Putative glucokinase |
| AN8690 | Protein expressed at increased levels during osmoadaptation |
| AN8692 | Thioredoxin-dependent peroxidase |
| AN8695 | Ortholog of A. fumigatus Af293 : Afu6g02340, Neosartorya fischeri NRRL 181 : NFIA_048600, Aspergillus wentii : Aspwe1_0035776 and Aspergillus versicolor : Aspve1_0055004 |
| AN8696 | Ortholog(s) have tRNA methyltransferase activity, role in tRNA methylation, wybutosine biosynthetic process and cytosol, nucleus localization |
| AN8697 | Ortholog of A. fumigatus Af293 : Afu6g02360, A. niger CBS 513.88 : An12g08390, Aspergillus wentii : Aspwe1_0179779, Aspergillus sydowii : Aspsy1_0092191 and Aspergillus terreus NIH2624 : ATET_07214 |
| AN8701 | Has domain(s) with predicted zinc ion binding activity |
| AN8702 | Ortholog(s) have ubiquitin conjugating enzyme activity, ubiquitin-protein transferase activity and role in UV-damage excision repair, free ubiquitin chain polymerization, postreplication repair, protein K63-linked ubiquitination |
| AN8705 | Ortholog(s) have cytoplasmic stress granule, cytosol, nucleus localization |
| AN8709 | Putative aspartate transaminase with a predicted role in alanine and aspartate metabolism |
| AN8718 | Has domain(s) with predicted 2-dehydropantoate 2-reductase activity, NADP binding, coenzyme binding, oxidoreductase activity, oxidoreductase activity, acting on the CH-OH group of donors, NAD or NADP as acceptor activity |
| AN8723 | protein of unknown function |
| AN8723-uORF | Conserved upstream open reading frame (uORF) of AN8723 |
| AN8724 | Ortholog(s) have role in chromatin silencing at centromere outer repeat region, mRNA cis splicing, via spliceosome and U2-type spliceosomal complex, nucleolus localization |
| AN8738 | protein of unknown function |
| AN8754 | Putative asparagine synthase with a predicted role in asparagine metabolism |
| AN8760 | Has domain(s) with predicted role in transmembrane transport and integral component of membrane localization |
| AN8761 | Protein with polygalacturonase activity, involved in degradation of pectin |
| AN8763 | Ortholog(s) have mitochondrion localization |
| AN8774 | Transcript induced in response to calcium dichloride in a CrzA-dependent manner |
| AN8782 | Putative S-formylglutathione hydrolase with a predicted role in pyruvate metabolism |
| AN8792 | Has domain(s) with predicted catalytic activity and role in carbohydrate metabolic process |
| AN8793 | Putative succinate dehydrogenase (ubiquinone) with a predicted role in the TCA cycle |
| AN8794 | Ortholog(s) have structural molecule activity and mitochondrial large ribosomal subunit localization |
| AN8801 | Ortholog(s) have glyoxysome localization |
| AN8802 | Ortholog of Aspergillus versicolor : Aspve1_0088238 and Aspergillus sydowii : Aspsy1_0035041 |
| AN8813 | Has domain(s) with predicted ATP binding, ATPase activity, ATPase activity, coupled to transmembrane movement of substances, nucleoside-triphosphatase activity, nucleotide binding activity and role in transport |
| AN8818 | protein of unknown function |
| AN8824 | Ortholog(s) have ribosomal large subunit binding activity |
| AN8829 | Ortholog of A. oryzae RIB40 : AO090009000665, Aspergillus wentii : Aspwe1_0054722, Aspergillus sydowii : Aspsy1_0117087 and Aspergillus terreus NIH2624 : ATET_02810, ATET_09435 |
| AN8830 | Predicted protein kinase involved in halotolerance |
| AN8843 | Putative homoserine kinase with a predicted role in glycine, serine, and threonine metabolism |
| AN8844 | Putative E3 ubiquitin-protein ligase |
| AN8870 | Expression increased in salt-adapted strains |
| AN8881 | Predicted siderophore transporter |
| AN8882 | Ortholog of A. fumigatus Af293 : Afu8g02740, A. niger CBS 513.88 : An03g06840, Aspergillus wentii : Aspwe1_0055533, Aspergillus sydowii : Aspsy1_0522885 and Aspergillus terreus NIH2624 : ATET_10387 |
| AN8885 | Has domain(s) with predicted DNA binding, RNA polymerase II transcription factor activity, sequence-specific DNA binding, zinc ion binding activity and role in regulation of transcription, DNA-templated, transcription, DNA-templated |
| AN8896 | Has domain(s) with predicted oxidoreductase activity and role in metabolic process |
| AN8897 | Has domain(s) with predicted hydrolase activity and role in metabolic process |
| AN8898 | Putative flavin-containing monooxygenase |
| AN8903 | Putative peptide transporter |
| AN8911 | Has domain(s) with predicted role in transmembrane transport and integral component of membrane localization |
| AN8916 | putative transcription factor |
| AN8923 | Ortholog of A. fumigatus Af293 : Afu3g01872, Neosartorya fischeri NRRL 181 : NFIA_002950 and Aspergillus fumigatus A1163 : AFUB_046550 |
| AN8925 | protein of unknown function |
| AN8928 | Putative plasma membrane ATP-binding cassette (ABC) transporter with a predicted role in multidrug resistance |
| AN8934 | Has domain(s) with predicted role in transmembrane transport and integral component of membrane localization |
| AN8935 | Putative transferase with a predicted role in the pentose-phosphate shunt or xylulose metabolism |
| AN8938 | Has domain(s) with predicted RNA polymerase II transcription factor activity, sequence-specific DNA binding, zinc ion binding activity, role in regulation of transcription, DNA-templated and nucleus localization |
| AN8941 | Putative potassium-transporting ATPase (alpha 1 isoform) with a predicted role in energy metabolism |
| AN8947 | Putative exo-1,3-beta-glucanase with a predicted role in glucan processing |
| AN8949 | Has domain(s) with predicted sequence-specific DNA binding, transcription factor activity, sequence-specific DNA binding activity and role in regulation of transcription, DNA-templated |
| AN8950 | protein of unknown function |
| AN8962 | Has domain(s) with predicted solute:proton antiporter activity, role in cation transport, transmembrane transport and integral component of membrane localization |
| AN8966 | Has domain(s) with predicted neurotransmitter:sodium symporter activity, role in neurotransmitter transport and integral component of membrane localization |
| AN8969 | Has domain(s) with predicted catalytic activity, lysozyme activity and role in carbohydrate metabolic process, cell wall macromolecule catabolic process, peptidoglycan catabolic process |
| AN8973 | Has domain(s) with predicted RNA polymerase II transcription factor activity, sequence-specific DNA binding, transcription factor activity, sequence-specific DNA binding, zinc ion binding activity |
| AN8986 | Ortholog of A. oryzae RIB40 : AO090005001017, Aspergillus versicolor : Aspve1_0047420, Aspergillus clavatus NRRL 1 : ACLA_018770 and Aspergillus sydowii : Aspsy1_0051004 |
| AN8990 | Ortholog of A. fumigatus Af293 : Afu8g00840, A. oryzae RIB40 : AO090023000465, Neosartorya fischeri NRRL 181 : NFIA_094290 and Aspergillus wentii : Aspwe1_0046447, Aspwe1_0100493 |
| AN8994 | Ortholog of A. oryzae RIB40 : AO090009000566, Aspergillus zonatus : Aspzo1_0127856, Aspergillus sydowii : Aspsy1_0062118 and Aspergillus carbonarius ITEM 5010 : Acar5010_398349 |
| AN9000 | Has domain(s) with predicted role in transmembrane transport and integral component of membrane localization |
| AN9011 | Putative aryl-alcohol oxidase-related protein |
| AN9012 | Has domain(s) with predicted catalytic activity, lyase activity and role in metabolic process |
| AN9019 | protein of unknown function |
| AN9035 | Putative alpha-galactosidase with a predicted role in galactose and galactitol metabolism and in degradation of mannans |
| AN9037 | Has domain(s) with predicted molybdenum ion binding, oxidoreductase activity and role in oxidation-reduction process |
| AN9040 | protein of unknown function |
| AN9041 | Putative 3-hydroxyacyl-CoA dehydrogenase with a predicted role in fatty acid metabolism |
| AN9042 | Putative alpha-1,3-glucanase |
| AN9043 | Has domain(s) with predicted DNA binding, RNA polymerase II transcription factor activity, sequence-specific DNA binding, zinc ion binding activity and role in regulation of transcription, DNA-templated, transcription, DNA-templated |
| AN9044 | Has domain(s) with predicted FMN binding, catalytic activity, oxidoreductase activity and role in oxidation-reduction process |
| AN9048 | Putative histidine-containing phosphotransfer protein |
| AN9053 | Putative malate dehydrogenase |
| AN9080 | Ortholog(s) have cytosol, mitochondrial intermembrane space, nucleus localization |
| AN9086 | Component of the TRAPII complex that mediates Rab guanyl-nucleotide exchange factor activity, involved in Golgi vesicle-mediated transport |
| AN9093 | Ortholog(s) have mRNA binding, single-stranded DNA binding, translation regulator activity and role in cellular response to cold, cellular response to drug, cellular response to osmotic stress, positive regulation of translation |
| AN9096 | Has domain(s) with predicted RNA polymerase II transcription factor activity, sequence-specific DNA binding, zinc ion binding activity, role in regulation of transcription, DNA-templated and nucleus localization |
| AN9100 | Ortholog of A. fumigatus Af293 : Afu7g02100, A. niger CBS 513.88 : An12g00570, A. oryzae RIB40 : AO090038000587, Aspergillus wentii : Aspwe1_0112810 and Aspergillus sydowii : Aspsy1_0035583 |
| AN9115 | Ortholog of A. niger CBS 513.88 : An10g00220, A. oryzae RIB40 : AO090010000382, Aspergillus wentii : Aspwe1_0182832, Aspergillus versicolor : Aspve1_0180171 and Aspergillus niger ATCC 1015 : 192862-mRNA |
| AN9117 | Has domain(s) with predicted DNA binding, zinc ion binding activity, role in transcription, DNA-templated and nucleus localization |
| AN9118 | Ortholog of A. fumigatus Af293 : Afu7g01900, A. niger CBS 513.88 : An12g00740, Neosartorya fischeri NRRL 181 : NFIA_114630 and Aspergillus versicolor : Aspve1_0047801 |
| AN9119 | Ortholog(s) have nucleus localization |
| AN9120 | Ortholog(s) have DNA binding, DNA-directed RNA polymerase activity, RNA binding activity and role in chromatin silencing by small RNA, transcription from RNA polymerase II promoter |
| AN9122 | Has domain(s) with predicted hydrolase activity and role in metabolic process |
| AN9128 | Has domain(s) with predicted catalytic activity and role in metabolic process |
| AN9129 | Ortholog(s) have role in pathogenesis |
| AN9137 | Ortholog of A. fumigatus Af293 : Afu7g01695, Aspergillus clavatus NRRL 1 : ACLA_065860, Aspergillus zonatus : Aspzo1_0012532 and Aspergillus sydowii : Aspsy1_0093862 |
| AN9140 | Putative MFS-type amino acid transporter |
| AN9141 | Has domain(s) with predicted RNA polymerase II transcription factor activity, sequence-specific DNA binding, zinc ion binding activity, role in regulation of transcription, DNA-templated and nucleus localization |
| AN9142 | Ortholog of A. fumigatus Af293 : Afu7g01570, Aspergillus wentii : Aspwe1_0029597, Aspergillus sydowii : Aspsy1_0093879 and Aspergillus terreus NIH2624 : ATET_01773 |
| AN9143 | Putative molybdopterin synthase small subunit involved in molybdenum cofactor biosynthesis |
| AN9159 | Ortholog of A. nidulans FGSC A4 : AN7899/dbaE, AN2031, AN0529, AN1032/afoC, A. fumigatus Af293 : Afu8g00910 and A. niger CBS 513.88 : An03g01180, An09g01790, An03g01810 |
| AN9168 | Ortholog(s) have solute:proton symporter activity, role in glycerol transport, transmembrane transport and plasma membrane localization |
| AN9173 | Sugar transporter |
| AN9180 | Putative transketolase |
| AN9181 | Ortholog of A. nidulans FGSC A4 : AN8354, AN8970, A. fumigatus Af293 : Afu1g12440, Afu1g12460, Afu6g00230 and A. niger CBS 513.88 : An12g03270, An10g01010, An01g11070, An11g04060, An16g00990 |
| AN9184 | Ortholog(s) have plasma membrane localization |
| AN9191 | Ortholog(s) have intracellular localization |
| AN9192 | Ortholog of A. fumigatus Af293 : Afu3g03910, A. niger CBS 513.88 : An13g01790, A. oryzae RIB40 : AO090038000138, Neosartorya fischeri NRRL 181 : NFIA_006190 and Aspergillus kawachii : Aspka1_0177086 |
| AN9193 | Putative LaeA-like methyltransferase |
| AN9200 | Ortholog of A. nidulans FGSC A4 : AN7637, A. fumigatus Af293 : Afu4g00530, A. niger CBS 513.88 : An08g11390, An08g08230, An08g12160 and Aspergillus wentii : Aspwe1_0060194, Aspwe1_0120922 |
| AN9210 | Putative cytochrome P450 |
| AN9239 | protein of unknown function |
| AN9251 | Putative cytochrome P450 |
| AN9276 | Putative beta-1,4-endomannanase |
| AN9284 | Ortholog of Aspergillus tubingensis : Asptu1_0059406, Aspergillus versicolor : Aspve1_0129968 and Aspergillus sydowii : Aspsy1_0068838 |
| AN9294 | Has domain(s) with predicted role in transmembrane transport and integral component of membrane localization |
| AN9307 | Has domain(s) with predicted catalytic activity, molybdenum ion binding, pyridoxal phosphate binding activity |
| AN9308 | Has domain(s) with predicted UDP-N-acetylmuramate dehydrogenase activity, flavin adenine dinucleotide binding, oxidoreductase activity and role in oxidation-reduction process |
| AN9313 | Putative cytochrome P450 |
| AN9314 | Protein with homology to entkaurene synthases |
| AN9340 | Alpha,alpha-trehalase with a role in trehalose hydrolysis |
| AN9349 | protein of unknown function |
| AN9350 | protein of unknown function |
| AN9351 | Ortholog of Aspergillus versicolor : Aspve1_0048024 and Aspergillus sydowii : Aspsy1_0163543 |
| AN9355 | Ortholog of A. nidulans FGSC A4 : AN10864, A. oryzae RIB40 : AO090023000948, Neosartorya fischeri NRRL 181 : NFIA_073340, NFIA_096190 and Aspergillus wentii : Aspwe1_0022222, Aspwe1_0173992 |
| AN9356 | Has domain(s) with predicted oxidoreductase activity and role in oxidation-reduction process |
| AN9357 | Ortholog(s) have cytosol, nucleus localization |
| AN9360 | Ortholog(s) have nucleolus localization |
| AN9367 | Putative pectate lyase, involved in degradation of pectin |
| AN9380 | Putative chitin deacetylase |
| AN9402 | Ortholog(s) have endoplasmic reticulum localization |
| AN9403 | Putative pyruvate dehydrogenase (lipoamide) with a predicted role in pyruvate metabolism |
| AN9405 | Ortholog(s) have role in lipid homeostasis, mitochondrion organization and integral component of mitochondrial membrane, mitochondrial outer membrane localization |
| AN9422 | Ortholog of A. fumigatus Af293 : Afu8g03905, A. oryzae RIB40 : AO090103000004, Neosartorya fischeri NRRL 181 : NFIA_096610, Aspergillus wentii : Aspwe1_0030770 and Aspergillus versicolor : Aspve1_0049677 |
| AN9424 | Ortholog(s) have cyclin-dependent protein serine/threonine kinase activator activity, protein complex binding, zinc ion binding activity |
| AN9426 | Has domain(s) with predicted ADP binding, catalytic activity, microtubule motor activity, nucleoside-triphosphatase activity, nucleotide binding activity, role in nucleoside metabolic process and kinesin complex localization |
| AN9434 | Ortholog(s) have cytosol, nucleus localization |
| AN9438 | Ortholog(s) have mitochondrion localization |
| AN9440 | protein of unknown function |
| AN9445 | Has domain(s) with predicted ATP binding, ATP-dependent helicase activity, nucleic acid binding activity |
| AN9446 | Putative pantothenate kinase with a predicted role in Coenzyme A and pantothenate biosynthesis |
| AN9448 | Ortholog of A. fumigatus Af293 : Afu1g03030, A. oryzae RIB40 : AO090005000796, Aspergillus wentii : Aspwe1_0169602, Aspergillus sydowii : Aspsy1_0628822 and Aspergillus terreus NIH2624 : ATET_01955 |
| AN9463 | Ortholog(s) have actin binding, calmodulin binding activity |
| AN9482 | Ortholog of A. fumigatus Af293 : Afu5g09370, A. niger CBS 513.88 : An07g03610, A. oryzae RIB40 : AO090020000554, Aspergillus wentii : Aspwe1_0048231 and Aspergillus sydowii : Aspsy1_0035062 |
| AN9486 | Ortholog(s) have role in protein lipoylation and mitochondrion localization |
| AN9508 | protein of unknown function |
| AN9522 | Has domain(s) with predicted catalytic activity and role in metabolic process |
| AN9532 | protein of unknown function |

***A. fumigatus***

| Gene ID | Annotation |
| --- | --- |
| Afu1g00160 | Ortholog of A. oryzae RIB40 : AO090102000436, AO090138000137 and Aspergillus fumigatus A1163 : AFUB_097610 |
| Afu1g00170 | Ortholog of Aspergillus wentii : Aspwe1_0166268, Aspergillus fumigatus A1163 : AFUB_097600 and Aspergillus sydowii : Aspsy1_0165358, Aspsy1_0711744 |
| Afu1g00200 | Ortholog of Aspergillus fumigatus A1163 : AFUB_097480 and Aspergillus sydowii : Aspsy1_0037491 |
| Afu1g00230 | Has domain(s) with predicted ATP binding, phosphotransferase activity, alcohol group as acceptor activity, role in lipopolysaccharide biosynthetic process and membrane localization |
| Afu1g00440 | DUF895 domain membrane protein |
| Afu1g00540 | Has domain(s) with predicted hydrolase activity, hydrolyzing O-glycosyl compounds activity and role in carbohydrate metabolic process |
| Afu1g00570 | Predicted mariner Ant1 transposon-related ORF |
| Afu1g00850 | Has domain(s) with predicted catalytic activity and role in nucleoside metabolic process |
| Afu1g00950 | protein of unknown function |
| Afu1g01140 | Ortholog of A. niger CBS 513.88 : An03g00980, A. oryzae RIB40 : AO090102000017, Neosartorya fischeri NRRL 181 : NFIA_023450 and Aspergillus versicolor : Aspve1_0142180 |
| Afu1g01210 | Ortholog of Aspergillus fumigatus A1163 : AFUB_079590 and Aspergillus niger ATCC 1015 : 171940-mRNA |
| Afu1g01230 | Has domain(s) with predicted hydrolase activity, hydrolyzing O-glycosyl compounds activity and role in carbohydrate metabolic process |
| Afu1g01290 | Has domain(s) with predicted electron carrier activity, heme binding, iron ion binding, monooxygenase activity, oxidoreductase activity, acting on paired donors, with incorporation or reduction of molecular oxygen activity |
| Afu1g01360 | Ortholog of A. nidulans FGSC A4 : AN3872, AN5090, A. niger CBS 513.88 : An18g00390, Aspergillus wentii : Aspwe1_0037446, Aspergillus sydowii : Aspsy1_0121091 and Aspergillus terreus NIH2624 : ATET_06529 |
| Afu1g01440 | Ortholog of Aspergillus fumigatus A1163 : AFUB_079340 |
| Afu1g01600 | Putative deoxyribodipyrimidine photolyase |
| Afu1g01610 | Transcript up-regulated in conidia exposed to neutrophils |
| Afu1g01640 | Has domain(s) with predicted ATP binding, microtubule binding, microtubule motor activity, role in microtubule-based movement and microtubule associated complex localization |
| Afu1g01660 | Ortholog(s) have rhamnogalacturonan acetylesterase activity and role in pectin catabolic process |
| Afu1g01680 | Branched-chain amino acid aminotransferase |
| Afu1g01730 | Has domain(s) with predicted catalytic activity |
| Afu1g01760 | Ortholog of A. nidulans FGSC A4 : AN0379, A. oryzae RIB40 : AO090005000918, Aspergillus wentii : Aspwe1_0025697 and Aspergillus sydowii : Aspsy1_0115500 |
| Afu1g01770 | Ortholog(s) have cytosol localization |
| Afu1g01780 | Has domain(s) with predicted oxidoreductase activity, transferase activity, transferring acyl groups other than amino-acyl groups, zinc ion binding activity and role in oxidation-reduction process |
| Afu1g01790 | Ortholog of A. nidulans FGSC A4 : AN10070, A. niger CBS 513.88 : An01g06420, A. oryzae RIB40 : AO090005000908, Aspergillus wentii : Aspwe1_0049751 and Aspergillus sydowii : Aspsy1_0144393 |
| Afu1g01812 | Has domain(s) with predicted role in transmembrane transport and integral component of membrane localization |
| Afu1g01870 | Ortholog(s) have role in protein import into mitochondrial matrix and plasma membrane, presequence translocase-associated import motor localization |
| Afu1g01940 | Ortholog of A. nidulans FGSC A4 : AN8644, A. niger CBS 513.88 : An04g08240 and A. oryzae RIB40 : AO090026000236, AO090010000588, AO090011000050, AO090009000101 |
| Afu1g01960 | Has domain(s) with predicted RNA polymerase II transcription factor activity, sequence-specific DNA binding, transcription factor activity, sequence-specific DNA binding, zinc ion binding activity |
| Afu1g02110 | 3-deoxy-7-phosphoheptulonate synthase |
| Afu1g02140 | Putative glycogen debranching enzyme |
| Afu1g02160 | Ortholog of Aspergillus fumigatus A1163 : AFUB_002540 |
| Afu1g02200 | Ortholog of A. nidulans FGSC A4 : AN10073, A. niger CBS 513.88 : An01g06050, A. oryzae RIB40 : AO090005000879, Neosartorya fischeri NRRL 181 : NFIA_022450 and Aspergillus clavatus NRRL 1 : ACLA_032230 |
| Afu1g02340 | Ortholog of A. nidulans FGSC A4 : AN0334, A. oryzae RIB40 : AO090005000866, Aspergillus wentii : Aspwe1_0169667 and Aspergillus sydowii : Aspsy1_0145773 |
| Afu1g02520 | Ortholog of Aspergillus fumigatus A1163 : AFUB_002900 |
| Afu1g02630 | Ortholog(s) have cytosol, nucleolus localization |
| Afu1g02840 | Ortholog of A. nidulans FGSC A4 : AN0276, A. niger CBS 513.88 : An01g04950, A. oryzae RIB40 : AO090005000773, Aspergillus wentii : Aspwe1_0169585 and Aspergillus sydowii : Aspsy1_0085871 |
| Afu1g02850 | Ortholog of A. nidulans FGSC A4 : AN0275, A. fumigatus Af293 : Afu6g14530, A. niger CBS 513.88 : An01g04970, A. oryzae RIB40 : AO090005000775 and Aspergillus wentii : Aspwe1_0106139 |
| Afu1g02890 | Ortholog(s) have dUTP diphosphatase activity and cytosol, nucleus localization |
| Afu1g02980 | Putative 6-phosphogluconolactonase |
| Afu1g03070 | Ortholog(s) have SAGA complex, cytosol, transcription factor TFIID complex localization |
| Afu1g03120 | Ortholog of A. niger CBS 513.88 : An01g04910, A. oryzae RIB40 : AO090005000771, Aspergillus wentii : Aspwe1_0169581 and Aspergillus terreus NIH2624 : ATET_02526 |
| Afu1g03180 | Has domain(s) with predicted carbon-sulfur lyase activity and role in metabolic process |
| Afu1g03195 | Has domain(s) with predicted RNA polymerase II transcription factor activity, sequence-specific DNA binding, zinc ion binding activity, role in regulation of transcription, DNA-templated and nucleus localization |
| Afu1g03200 | Putative major facilitator superfamily (MFS) transporter |
| Afu1g03215 | rRNA |
| Afu1g03230 | Has domain(s) with predicted ATP binding, ATPase activity |
| Afu1g03310 | Ortholog of Aspergillus fumigatus A1163 : AFUB_003730 |
| Afu1g03320 | Ortholog of Aspergillus fumigatus A1163 : AFUB_003740 |
| Afu1g03352 | Has domain(s) with predicted hydrolase activity |
| Afu1g03370 | Ortholog of A. niger CBS 513.88 : An08g07440, An07g01270, An06g00780, A. oryzae RIB40 : AO090120000190 and Neosartorya fischeri NRRL 181 : NFIA_021280, NFIA_024060, NFIA_049510 |
| Afu1g03400 | Ortholog(s) have cytoplasm localization |
| Afu1g03440 | Ortholog(s) have adenine nucleotide transmembrane transporter activity, role in ATP transport, fatty acid beta-oxidation, peroxisome organization and glyoxysome, integral component of peroxisomal membrane localization |
| Afu1g03450 | Ortholog(s) have calcium-dependent cysteine-type endopeptidase activity |
| Afu1g03600 | Ortholog(s) have cell adhesion molecule binding, glucan endo-1,6-beta-glucosidase activity, glucan exo-1,3-beta-glucosidase activity |
| Afu1g03830 | Ortholog(s) have tubulin binding activity, role in tubulin complex assembly and cytoplasm, prefoldin complex localization |
| Afu1g03860 | Ortholog(s) have transcription regulatory region DNA binding activity and role in regulation of cellular amide catabolic process, regulation of transcription, DNA-templated |
| Afu1g04040 | Ubiquitin |
| Afu1g04070 | Eukaryotic initiation factor 5A |
| Afu1g04100 | Ortholog of A. nidulans FGSC A4 : AN8649, A. niger CBS 513.88 : An01g02930, A. oryzae RIB40 : AO090009000519, Aspergillus wentii : Aspwe1_0037325, Aspwe1_0104267 and Aspergillus sydowii : Aspsy1_0048337 |
| Afu1g04440 | Ortholog of A. nidulans FGSC A4 : AN0454, A. niger CBS 513.88 : An01g03310, A. oryzae RIB40 : AO090003000907, Aspergillus wentii : Aspwe1_0150842 and Aspergillus sydowii : Aspsy1_0086117 |
| Afu1g04450 | GNAT-type acetyltransferase |
| Afu1g04480 | Ortholog(s) have Sin3-type complex localization |
| Afu1g04485 | unspecified product |
| Afu1g04490 | Ortholog(s) have cytosol, nucleus localization |
| Afu1g04580 | Ortholog(s) have cytosol, nucleus localization |
| Afu1g04590 | Ortholog(s) have histone acetyltransferase activity and role in histone acetylation, regulation of transcription from RNA polymerase II promoter |
| Afu1g04600 | Has domain(s) with predicted zinc ion binding activity |
| Afu1g04770 | Ortholog of A. nidulans FGSC A4 : AN10089, A. niger CBS 513.88 : An01g03670, Neosartorya fischeri NRRL 181 : NFIA_019990, Aspergillus clavatus NRRL 1 : ACLA_029910 and Aspergillus zonatus : Aspzo1_0131370 |
| Afu1g04810 | Ortholog of A. nidulans FGSC A4 : AN0424, A. niger CBS 513.88 : An01g03730, Aspergillus wentii : Aspwe1_0169505 and Aspergillus sydowii : Aspsy1_0641478 |
| Afu1g04845 | unspecified product |
| Afu1g04850 | Ortholog(s) have ATP-dependent microtubule motor activity, plus-end-directed activity |
| Afu1g05000 | Ortholog(s) have endoplasmic reticulum localization |
| Afu1g05120 | Ortholog(s) have endoplasmic reticulum localization |
| Afu1g05220 | Has domain(s) with predicted selenium binding activity and role in cell redox homeostasis |
| Afu1g05300 | Ortholog of A. nidulans FGSC A4 : AN0246, A. niger CBS 513.88 : An01g04570, A. oryzae RIB40 : AO090003000826, Aspergillus wentii : Aspwe1_0049630 and Aspergillus sydowii : Aspsy1_0142918 |
| Afu1g05310 | Ortholog(s) have role in negative regulation of G0 to G1 transition, ribosomal small subunit biogenesis and 90S preribosome, nucleolus localization |
| Afu1g05370 | Ortholog of A. nidulans FGSC A4 : AN4062, A. niger CBS 513.88 : An18g04210, A. oryzae RIB40 : AO090009000407, Aspergillus wentii : Aspwe1_0104515 and Aspergillus sydowii : Aspsy1_0057614 |
| Afu1g05450 | Parvulin-like peptidyl-prolyl cis-trans isomerase |
| Afu1g05540 | Ortholog of A. nidulans FGSC A4 : AN4076, Aspergillus wentii : Aspwe1_0037239, Aspergillus versicolor : Aspve1_0054320, Aspergillus zonatus : Aspzo1_0131207 and Aspergillus terreus NIH2624 : ATET_03076 |
| Afu1g05550 | Has domain(s) with predicted DNA binding activity |
| Afu1g05570 | Cysteine dioxygenase |
| Afu1g05590 | Ortholog of A. nidulans FGSC A4 : AN4083, A. niger CBS 513.88 : An18g04430, A. oryzae RIB40 : AO090009000382, Aspergillus wentii : Aspwe1_0169293 and Aspergillus sydowii : Aspsy1_0201498 |
| Afu1g05595 | Ortholog of A. nidulans FGSC A4 : AN4084, A. niger CBS 513.88 : An18g04440, A. oryzae RIB40 : AO090009000381, Aspergillus wentii : Aspwe1_0025280 and Aspergillus sydowii : Aspsy1_0149732 |
| Afu1g05640 | Ortholog(s) have cytosol localization |
| Afu1g05650 | Ortholog of A. nidulans FGSC A4 : AN12323, A. niger CBS 513.88 : An18g03335, A. oryzae RIB40 : AO090009000372, Neosartorya fischeri NRRL 181 : NFIA_019100 and Aspergillus wentii : Aspwe1_0037223 |
| Afu1g05655 | Ortholog(s) have cell cortex localization |
| Afu1g05690 | Ortholog of A. nidulans FGSC A4 : AN4089, A. niger CBS 513.88 : An18g03450, A. oryzae RIB40 : AO090009000365, Aspergillus wentii : Aspwe1_0104096 and Aspergillus sydowii : Aspsy1_0057643 |
| Afu1g05700 | Ortholog(s) have U2 snRNP, cytosol localization |
| Afu1g05710 | Ortholog(s) have tRNA-specific adenosine-37 deaminase activity, role in tRNA modification and cytosol, nucleus localization |
| Afu1g05840 | Ortholog of A. nidulans FGSC A4 : AN4112, A. niger CBS 513.88 : An18g03610, A. oryzae RIB40 : AO090009000350, Aspergillus wentii : Aspwe1_0038192 and Aspergillus sydowii : Aspsy1_0089097 |
| Afu1g05850 | Has domain(s) with predicted role in response to antibiotic |
| Afu1g05860 | Ortholog of A. nidulans FGSC A4 : AN4104, A. niger CBS 513.88 : An18g03590, A. oryzae RIB40 : AO090009000354, Aspergillus wentii : Aspwe1_0026045 and Aspergillus sydowii : Aspsy1_0089091 |
| Afu1g05870 | Putative phospholipid biosynthesis protein |
| Afu1g05890 | Ortholog of Aspergillus fumigatus A1163 : AFUB_006280 |
| Afu1g05910 | Ortholog of Neosartorya fischeri NRRL 181 : NFIA_018810, Aspergillus fumigatus A1163 : AFUB_006300 and Aspergillus clavatus NRRL 1 : ACLA_028690 |
| Afu1g05920 | Ortholog of A. nidulans FGSC A4 : AN4193, A. niger CBS 513.88 : An18g03670, A. oryzae RIB40 : AO090120000081, Aspergillus wentii : Aspwe1_0038204 and Aspergillus sydowii : Aspsy1_0059184 |
| Afu1g05960 | Peptidase family M28 family protein |
| Afu1g05980 | Putative AMP-binding domain protein |
| Afu1g06050 | Has domain(s) with predicted sugar:proton symporter activity, role in carbohydrate transport and Golgi membrane, integral component of membrane localization |
| Afu1g06150 | Ortholog of S. cerevisiae : SDL1, A. nidulans FGSC A4 : AN3866, AN4217, AN2525, A. fumigatus Af293 : Afu3g14470, Afu4g07810 and A. niger CBS 513.88 : An09g03960, An04g02220 |
| Afu1g06250 | Has domain(s) with predicted catalytic activity and role in metabolic process |
| Afu1g06280 | Ortholog(s) have endoplasmic reticulum localization |
| Afu1g06310 | Ortholog(s) have RSC complex, SWI/SNF complex, cytosol localization |
| Afu1g06350 | Ortholog of A. nidulans FGSC A4 : AN10518, A. niger CBS 513.88 : An18g05000, A. oryzae RIB40 : AO090001000412, Neosartorya fischeri NRRL 181 : NFIA_018360 and Aspergillus wentii : Aspwe1_0038230 |
| Afu1g06520 | Ortholog(s) have GTPase activity, tRNA binding, translation initiation factor activity, role in mitochondrial translation and mitochondrion localization |
| Afu1g06540 | Has domain(s) with predicted DNA binding, RNA polymerase II transcription factor activity, sequence-specific DNA binding, zinc ion binding activity and role in regulation of transcription, DNA-templated, transcription, DNA-templated |
| Afu1g06570 | Ortholog(s) have structural constituent of ribosome activity and mitochondrial small ribosomal subunit localization |
| Afu1g06630 | Ortholog(s) have cytosol, nucleus localization |
| Afu1g06690 | Ortholog(s) have role in maturation of SSU-rRNA and cytoplasm, nucleus localization |
| Afu1g06770 | 40S ribosomal protein S26 |
| Afu1g06960 | Putative pyruvate dehydrogenase complex subunit alpha |
| Afu1g07000 | Ortholog(s) have role in DNA repair, G2 DNA damage checkpoint |
| Afu1g07010 | Putative phosphatidate cytidylyltransferase |
| Afu1g07030 | Ortholog of Aspergillus fumigatus A1163 : AFUB_007400 |
| Afu1g07040 | Ortholog of A. nidulans FGSC A4 : AN10646, A. niger CBS 513.88 : An07g09600, A. oryzae RIB40 : AO090012000956, Aspergillus wentii : Aspwe1_0111941 and Aspergillus terreus NIH2624 : ATET_02639 |
| Afu1g07110 | Ortholog of A. nidulans FGSC A4 : AN5152, A. niger CBS 513.88 : An07g09650, A. oryzae RIB40 : AO090012000963, Aspergillus wentii : Aspwe1_0028295 and Aspergillus sydowii : Aspsy1_0091614 |
| Afu1g07130 | Ortholog(s) have mitochondrion localization |
| Afu1g07260 | Ortholog(s) have Golgi apparatus, endoplasmic reticulum localization |
| Afu1g07270 | Has domain(s) with predicted protein heterodimerization activity |
| Afu1g07310 | Ortholog of A. nidulans FGSC A4 : AN10645, A. niger CBS 513.88 : An07g09850, A. oryzae RIB40 : AO090012000984, Aspergillus wentii : Aspwe1_0040933 and Aspergillus sydowii : Aspsy1_0155965 |
| Afu1g07360 | Ortholog of A. nidulans FGSC A4 : AN5136, A. niger CBS 513.88 : An07g09900, Aspergillus wentii : Aspwe1_0040941 and Aspergillus sydowii : Aspsy1_0047597 |
| Afu1g07420 | Ortholog(s) have SNAP receptor activity and role in ER to Golgi vesicle-mediated transport, retrograde vesicle-mediated transport, Golgi to ER, vesicle fusion |
| Afu1g07540 | Ortholog(s) have role in cellular response to drug and nuclear envelope, proteasome regulatory particle, lid subcomplex, proteasome storage granule localization |
| Afu1g08800 | Ortholog(s) have 3-hydroxyphenylacetate 6-hydroxylase activity |
| Afu1g08920 | Ortholog of A. nidulans FGSC A4 : AN1386, A. niger CBS 513.88 : An08g00410, A. oryzae RIB40 : AO090005001634, Aspergillus wentii : Aspwe1_0036270 and Aspergillus sydowii : Aspsy1_0138746 |
| Afu1g09020 | Ortholog(s) have role in poly(A)+ mRNA export from nucleus and cytosol, nuclear periphery, nuclear pore localization |
| Afu1g09180 | Has domain(s) with predicted role in histone modification, transcription elongation from RNA polymerase II promoter and Cdc73/Paf1 complex localization |
| Afu1g09290 | Has domain(s) with predicted catalytic activity and role in biosynthetic process |
| Afu1g09330 | Ortholog(s) have sequence-specific DNA binding activity and role in cytoplasmic translational initiation |
| Afu1g09350 | Has domain(s) with predicted protein heterodimerization activity, role in DNA-templated transcription, initiation, transcription from RNA polymerase II promoter and nucleus localization |
| Afu1g09390 | Has domain(s) with predicted nucleic acid binding, zinc ion binding activity |
| Afu1g09400 | Has domain(s) with predicted nucleic acid binding activity |
| Afu1g09430 | Ortholog of A. nidulans FGSC A4 : AN1346, A. oryzae RIB40 : AO090012000930, Aspergillus wentii : Aspwe1_0103922, Aspergillus sydowii : Aspsy1_0025400 and Aspergillus terreus NIH2624 : ATET_08566 |
| Afu1g09490 | Ortholog(s) have poly(A) binding activity, role in nuclear-transcribed mRNA poly(A) tail shortening and Mmi1 nuclear focus complex, cytoplasm, nuclear chromatin, nucleolus, nucleoplasm, polysome localization |
| Afu1g09540 | Ortholog(s) have GTPase regulator activity, mRNA binding activity |
| Afu1g09550 | Ortholog(s) have role in dynein-driven meiotic oscillatory nuclear movement, karyogamy involved in conjugation with cellular fusion, regulation of mitotic attachment of spindle microtubules to kinetochore |
| Afu1g09560 | Ortholog(s) have role in microtubule cytoskeleton organization, mitotic sister chromatid segregation and TORC1 complex, TORC2 complex, cytosol, nucleus localization |
| Afu1g09570 | Ortholog of A. nidulans FGSC A4 : AN1336, A. niger CBS 513.88 : An08g01160, A. oryzae RIB40 : AO090012000913, Aspergillus wentii : Aspwe1_0024569 and Aspergillus sydowii : Aspsy1_0037865 |
| Afu1g09650 | Putative GPI anchored protein |
| Afu1g09680 | Ortholog(s) have role in retrograde transport, endosome to Golgi and Golgi membrane, Golgi trans cisterna, fungal-type vacuole membrane localization |
| Afu1g09710 | Ortholog of Aspergillus fumigatus A1163 : AFUB_009160 |
| Afu1g09730 | Has domain(s) with predicted catalytic activity and membrane localization |
| Afu1g09790 | Ortholog(s) have role in cellular response to nitrogen starvation, chromatin silencing at centromere outer repeat region and chromatin silencing at silent mating-type cassette, more |
| Afu1g09810 | Has domain(s) with predicted RNA binding activity |
| Afu1g09920 | Ortholog of A. nidulans FGSC A4 : AN1275, A. niger CBS 513.88 : An08g01730, A. oryzae RIB40 : AO090038000461, Aspergillus wentii : Aspwe1_0343642 and Aspergillus sydowii : Aspsy1_0228584 |
| Afu1g09930 | Putative glycerol dehydrogenase |
| Afu1g10040 | Ortholog(s) have role in ascospore formation, asexual sporulation resulting in formation of a cellular spore and mitochondrion, nucleus localization |
| Afu1g10100 | Short-chain dehydrogenase |
| Afu1g10120 | Ortholog of A. nidulans FGSC A4 : AN8931, A. niger CBS 513.88 : An08g01940, A. oryzae RIB40 : AO090038000421, Aspergillus wentii : Aspwe1_0048983 and Aspergillus sydowii : Aspsy1_0044402 |
| Afu1g10130 | Putative adenosylhomocysteinase |
| Afu1g10190 | Ortholog(s) have role in mRNA cis splicing, via spliceosome and U4/U6 x U5 tri-snRNP complex localization |
| Afu1g10240 | Ortholog of Aspergillus fumigatus A1163 : AFUB_009650 |
| Afu1g10330 | Ortholog of A. niger CBS 513.88 : An08g02230, A. oryzae RIB40 : AO090038000397, Aspergillus wentii : Aspwe1_0036562 and Aspergillus sydowii : Aspsy1_0141143 |
| Afu1g10370 | Putative MFS multidrug transporter |
| Afu1g10460 | Ortholog of Neosartorya fischeri NRRL 181 : NFIA_015190 and Aspergillus clavatus NRRL 1 : ACLA_025070 |
| Afu1g10550 | Ortholog(s) have role in filamentous growth |
| Afu1g10590 | Putative GPI anchored protein |
| Afu1g10610 | Ortholog of Aspergillus fumigatus A1163 : AFUB_010030 |
| Afu1g10645 | Ortholog of A. nidulans FGSC A4 : AN1224, A. niger CBS 513.88 : An08g02720, A. oryzae RIB40 : AO090038000355, Neosartorya fischeri NRRL 181 : NFIA_015000 and Aspergillus wentii : Aspwe1_0168793 |
| Afu1g10720 | Ortholog(s) have DNA replication origin binding activity, role in mitotic DNA replication, mitotic DNA replication checkpoint and nuclear chromatin, nuclear origin of replication recognition complex localization |
| Afu1g10780 | Glycine cleavage system T protein |
| Afu1g10880 | Putative P-type calcium ATPase |
| Afu1g11060 | Ortholog of A. nidulans FGSC A4 : AN1173, A. niger CBS 513.88 : An08g03320, Aspergillus wentii : Aspwe1_0024838, Aspergillus sydowii : Aspsy1_0038077 and Aspergillus terreus NIH2624 : ATET_00305 |
| Afu1g11100 | Ortholog of A. nidulans FGSC A4 : AN1169, A. niger CBS 513.88 : An08g03390, A. oryzae RIB40 : AO090038000299, Neosartorya fischeri NRRL 181 : NFIA_014530 and Aspergillus wentii : Aspwe1_0172243 |
| Afu1g11140 | Has domain(s) with predicted iron ion binding, oxidoreductase activity and role in fatty acid biosynthetic process, oxidation-reduction process |
| Afu1g11160 | Ortholog(s) have nucleolus localization |
| Afu1g11170 | Ortholog(s) have role in reciprocal meiotic recombination and nucleus localization |
| Afu1g11230 | Hydroxymethylglutaryl-CoA (HMG-CoA) reductase |
| Afu1g11280 | Has domain(s) with predicted iron ion binding, oxidoreductase activity, oxidoreductase activity, acting on paired donors, with incorporation or reduction of molecular oxygen and 2-oxoglutarate as one donor, more |
| Afu1g11330 | Ortholog(s) have extracellular region localization |
| Afu1g11410 | Has domain(s) with predicted acid-amino acid ligase activity |
| Afu1g11450 | Ortholog of A. nidulans FGSC A4 : AN1156, A. niger CBS 513.88 : An08g03570, A. oryzae RIB40 : AO090038000280, Aspergillus wentii : Aspwe1_0068767 and Aspergillus sydowii : Aspsy1_0191007 |
| Afu1g11535 | Ortholog of S. cerevisiae : YDR286C, A. nidulans FGSC A4 : AN11843, Aspergillus wentii : Aspwe1_0040556, Aspergillus sydowii : Aspsy1_0083601 and Aspergillus carbonarius ITEM 5010 : Acar5010_202812 |
| Afu1g11720 | Ortholog(s) have extracellular region localization |
| Afu1g11780 | Ortholog of A. nidulans FGSC A4 : AN1116, A. niger CBS 513.88 : An08g03970, A. oryzae RIB40 : AO090038000243, Aspergillus wentii : Aspwe1_0109813 and Aspergillus sydowii : Aspsy1_0244584 |
| Afu1g11790 | Ortholog of A. nidulans FGSC A4 : AN1115, A. niger CBS 513.88 : An08g03980, A. oryzae RIB40 : AO090038000241, Aspergillus wentii : Aspwe1_0040527 and Aspergillus sydowii : Aspsy1_0038153 |
| Afu1g11860 | Ortholog(s) have mitochondrion targeting sequence binding, protein channel activity |
| Afu1g11900 | Putative G-protein coupled receptor |
| Afu1g12000 | Ortholog(s) have mRNA binding, poly(U) RNA binding activity and role in nuclear-transcribed mRNA catabolic process, nonsense-mediated decay, regulation of mRNA stability, stress granule assembly |
| Afu1g12190 | Has domain(s) with predicted arylformamidase activity and role in tryptophan catabolic process to kynurenine |
| Afu1g12250 | Mitochondrial hypoxia response domain protein |
| Afu1g12400 | Has domain(s) with predicted arylformamidase activity and role in tryptophan catabolic process to kynurenine |
| Afu1g12440 | Has domain(s) with predicted NAD binding, oxidoreductase activity, acting on the aldehyde or oxo group of donors, NAD or NADP as acceptor activity and role in cellular amino acid metabolic process, oxidation-reduction process |
| Afu1g12520 | Has domain(s) with predicted nucleoside-triphosphatase activity, nucleotide binding activity |
| Afu1g12640 | Ortholog of A. nidulans FGSC A4 : AN1049, A. niger CBS 513.88 : An08g05390, A. oryzae RIB40 : AO090012000714, Aspergillus wentii : Aspwe1_0501033 and Aspergillus sydowii : Aspsy1_0140149 |
| Afu1g12650 | Putative 3-ketoacyl-CoA ketothiolase with a predicted role in fatty acid beta oxidation |
| Afu1g12660 | Ortholog of A. nidulans FGSC A4 : AN1027, A. niger CBS 513.88 : An08g05410, A. oryzae RIB40 : AO090012000716, Aspergillus wentii : Aspwe1_0040406 and Aspergillus sydowii : Aspsy1_0038316 |
| Afu1g12670 | Ortholog(s) have DASH complex, cytosol, mitotic spindle pole body localization |
| Afu1g12690 | ABC multidrug transporter |
| Afu1g12710 | Ortholog of Aspergillus fumigatus A1163 : AFUB_012180 |
| Afu1g12800 | Putative NADPH isocitrate dehydrogenase |
| Afu1g12875 | Ortholog(s) have Mis6-Sim4 complex, condensed nuclear chromosome kinetochore, cytoplasm localization |
| Afu1g12890 | Putative 60S Ribosomal protein L5 |
| Afu1g12910 | Putative ABC fatty acid transporter |
| Afu1g13270 | Has domain(s) with predicted hydrolase activity and role in metabolic process |
| Afu1g13290 | Ortholog(s) have SAGA complex localization |
| Afu1g13620 | Putative oligopeptide transporter |
| Afu1g13640 | Ortholog of Aspergillus fumigatus A1163 : AFUB_013130 |
| Afu1g13800 | MFS multidrug transporter |
| Afu1g13810 | Ortholog of Aspergillus fumigatus A1163 : AFUB_013290 |
| Afu1g14050 | Putative F-box protein |
| Afu1g14080 | Putative integral membrane protein |
| Afu1g14150 | Ortholog of A. nidulans FGSC A4 : AN0754, A. niger CBS 513.88 : An01g11990, A. oryzae RIB40 : AO090012000447, Neosartorya fischeri NRRL 181 : NFIA_011270 and Aspergillus wentii : Aspwe1_0040208 |
| Afu1g14160 | Ortholog of Aspergillus fumigatus A1163 : AFUB_013700 |
| Afu1g14170 | Ortholog(s) have beta-galactosidase activity and role in carbohydrate metabolic process |
| Afu1g14200 | Putative mitochondrial processing peptidase beta subunit |
| Afu1g14240 | Ortholog(s) have role in Golgi vesicle transport and Golgi membrane localization |
| Afu1g14250 | Ortholog(s) have voltage-gated potassium channel activity, role in cellular potassium ion homeostasis, potassium ion transport and plasma membrane localization |
| Afu1g14380 | Has domain(s) with predicted oxidoreductase activity and role in metabolic process |
| Afu1g14520 | Ortholog(s) have NADPH dehydrogenase activity, role in spore germination and mitochondrial inner membrane localization |
| Afu1g14530 | General amidase |
| Afu1g14670 | Ortholog(s) have role in G2/M transition of mitotic cell cycle, histone H3-K4 methylation, positive regulation of mitotic nuclear division and Set1C/COMPASS complex, cytosol localization |
| Afu1g14800 | protein of unknown function |
| Afu1g14895 | protein of unknown function |
| Afu1g14910 | Ortholog(s) have cytoplasm localization |
| Afu1g14920 | Putative peptidase D |
| Afu1g15040 | Ortholog of A. nidulans FGSC A4 : AN0846, A. niger CBS 513.88 : An01g13210, A. oryzae RIB40 : AO090005001223, Aspergillus wentii : Aspwe1_0040051 and Aspergillus sydowii : Aspsy1_0038559 |
| Afu1g15080 | Ortholog of A. nidulans FGSC A4 : AN6081, AN0849, A. fumigatus Af293 : Afu2g09190, A. niger CBS 513.88 : An01g13240, An12g04840 and A. oryzae RIB40 : AO090005001220, AO090011000776 |
| Afu1g15240 | Ortholog(s) have tubulin binding activity, role in positive regulation of transcription elongation from RNA polymerase II promoter, tubulin complex assembly and cytosol, nucleus, prefoldin complex localization |
| Afu1g15250 | Ortholog of A. nidulans FGSC A4 : AN0861, A. oryzae RIB40 : AO090005001206, Aspergillus wentii : Aspwe1_0051149, Aspergillus sydowii : Aspsy1_0122516 and Aspergillus terreus NIH2624 : ATET_00827 |
| Afu1g15270 | Ortholog(s) have ATPase activity, misfolded protein binding activity, role in cellular response to misfolded protein, protein refolding, protein unfolding and cytosol, nuclear envelope localization |
| Afu1g15400 | Ortholog(s) have peroxisome matrix targeting signal-2 binding activity, role in fatty acid metabolic process, protein import into peroxisome matrix, docking and cytosol, nucleus, peroxisome localization |
| Afu1g15410 | Has domain(s) with predicted zinc ion binding activity |
| Afu1g15420 | Has domain(s) with predicted role in cell wall macromolecule catabolic process |
| Afu1g15450 | Ortholog(s) have adenylosuccinate synthase activity, sulfinylpropanyl adenylate synthase activity, role in 'de novo' AMP biosynthetic process, cellular response to cadmium ion, fumarate metabolic process and cytosol, nucleus localization |
| Afu1g15500 | Ortholog(s) have ribosome binding activity, role in cytoplasmic translation and extrinsic component of membrane, mitochondrial inner membrane localization |
| Afu1g15570 | Has domain(s) with predicted acid-amino acid ligase activity |
| Afu1g15590 | Putative succinate dehydrogenase membrane anchor subunit with a predicted role in the citric acid cycle |
| Afu1g15670 | Putative laccase |
| Afu1g16080 | protein of unknown function |
| Afu1g16160 | Has domain(s) with predicted RNA polymerase II transcription factor activity, sequence-specific DNA binding, zinc ion binding activity, role in regulation of transcription, DNA-templated and nucleus localization |
| Afu1g16170 | Ortholog of A. nidulans FGSC A4 : AN3546, A. fumigatus Af293 : Afu4g14400, Afu5g15040, Afu6g09300, A. niger CBS 513.88 : An08g11160, An04g08000 and A. oryzae RIB40 : AO090166000001 |
| Afu1g16200 | Ortholog of A. nidulans FGSC A4 : AN0934, A. niger CBS 513.88 : An01g11000, A. oryzae RIB40 : AO090005001089, Aspergillus wentii : Aspwe1_0171659 and Aspergillus sydowii : Aspsy1_0053430 |
| Afu1g16240 | Has domain(s) with predicted catalytic activity and role in metabolic process |
| Afu1g16290 | Ortholog(s) have cytosol localization |
| Afu1g16320 | Ortholog(s) have protein complex binding activity |
| Afu1g16480 | Ortholog(s) have mitochondrion localization |
| Afu1g16500 | Ortholog(s) have cytosol, nucleus localization |
| Afu1g16540 | Ortholog(s) have role in microtubule anchoring at spindle pole body, mitotic sister chromatid segregation, protein localization to mitotic spindle pole body |
| Afu1g16550 | Dihydrouridine synthase family protein |
| Afu1g16610 | Ortholog(s) have role in histone exchange and Swr1 complex, cytosol localization |
| Afu1g16730 | Ortholog(s) have pseudouridine synthase activity, role in enzyme-directed rRNA pseudouridine synthesis, mRNA pseudouridine synthesis, snRNA pseudouridine synthesis, tRNA pseudouridine synthesis and cytoplasm, nucleus localization |
| Afu1g16740 | Ortholog of Neosartorya fischeri NRRL 181 : NFIA_008640, Aspergillus fumigatus A1163 : AFUB_016120 and Aspergillus clavatus NRRL 1 : ACLA_018760 |
| Afu1g16880 | Ortholog(s) have glutathione S-conjugate-transporting ATPase activity and role in cellular response to cadmium ion, glutathione transmembrane import into vacuole, secondary metabolite biosynthetic process |
| Afu1g16970 | Ortholog(s) have GTPase activity, role in Golgi to plasma membrane protein transport, protein localization to organelle and Golgi apparatus, nuclear outer membrane-endoplasmic reticulum membrane network localization |
| Afu1g16980 | Ortholog of A. nidulans FGSC A4 : AN0633, A. niger CBS 513.88 : An01g09880, A. oryzae RIB40 : AO090005000977, Aspergillus wentii : Aspwe1_0109484 and Aspergillus sydowii : Aspsy1_0055497 |
| Afu1g17250 | Conidial cell wall hydrophobin involved in conidial cell wall composition |
| Afu1g17280 | Ortholog of A. niger CBS 513.88 : An09g04690, An04g08290, An09g01490, A. oryzae RIB40 : AO090701000484, AO090009000441 and Neosartorya fischeri NRRL 181 : NFIA_008090 |
| Afu1g17350 | Ortholog of A. oryzae RIB40 : AO090020000692, Neosartorya fischeri NRRL 181 : NFIA_008000, Aspergillus fumigatus A1163 : AFUB_016720 and Aspergillus clavatus NRRL 1 : ACLA_018150 |
| Afu1g17360 | Putative bZip-type transcription factor with similarity to atfA |
| Afu1g17370 | Putative heat shock protein |
| Afu1g17380 | Has domain(s) with predicted oxidoreductase activity and role in metabolic process |
| Afu1g17490 | Has domain(s) with predicted carbohydrate binding, carbon-oxygen lyase activity and role in amino sugar catabolic process, carbohydrate metabolic process |
| Afu1g17520 | Has domain(s) with predicted UDP-N-acetylmuramate dehydrogenase activity, catalytic activity, flavin adenine dinucleotide binding activity and role in oxidation-reduction process |
| Afu1g17530 | Has domain(s) with predicted role in transmembrane transport and integral component of membrane localization |
| Afu1g17590 | Putative phospholipase C, phoB-regulated |
| Afu1g17620 | Ortholog of A. oryzae RIB40 : AO090011000197, Aspergillus flavus NRRL 3357 : AFL2T_04974, Neosartorya fischeri NRRL 181 : NFIA_007750 and Aspergillus versicolor : Aspve1_0087209 |
| Afu1g17640 | Has domain(s) with predicted RNA polymerase II transcription factor activity, sequence-specific DNA binding, nucleic acid binding, zinc ion binding activity, role in regulation of transcription, DNA-templated and nucleus localization |
| Afu2g00100 | Has domain(s) with predicted RNA polymerase II transcription factor activity, sequence-specific DNA binding, zinc ion binding activity, role in regulation of transcription, DNA-templated and nucleus localization |
| Afu2g00200 | Ortholog(s) have catalase activity |
| Afu2g00220 | Putative aminopeptidase |
| Afu2g00300 | Ortholog of Aspergillus fumigatus A1163 : AFUB_017360 |
| Afu2g00310 | Transmembrane amino acid transporter family protein |
| Afu2g00320 | Putative sterol delta 5,6-desaturase with a predicted role in ergosterol biosynthesis |
| Afu2g00460 | Has domain(s) with predicted oxidoreductase activity and role in metabolic process |
| Afu2g00480 | Ortholog of Aspergillus fumigatus A1163 : AFUB_017540 |
| Afu2g00490 | Has domain(s) with predicted hydrolase activity, hydrolyzing O-glycosyl compounds activity and role in carbohydrate metabolic process |
| Afu2g00530 | Ortholog of Aspergillus fumigatus A1163 : AFUB_017590 |
| Afu2g00540 | Ortholog(s) have oxaloacetase activity and role in oxalate metabolic process, oxaloacetate metabolic process |
| Afu2g00580 | Ortholog of A. fumigatus Af293 : Afu7g05490, Neosartorya fischeri NRRL 181 : NFIA_032770, Aspergillus wentii : Aspwe1_0035458 and Aspergillus clavatus NRRL 1 : ACLA_007180, ACLA_093940 |
| Afu2g00590 | Predicted glutathione S transferase |
| Afu2g00730 | Has domain(s) with predicted UDP-N-acetylmuramate dehydrogenase activity, flavin adenine dinucleotide binding activity and role in oxidation-reduction process |
| Afu2g00800 | PelA protein |
| Afu2g00820 | Putative lipolytic enzyme with a predicted role in lipid metabolism |
| Afu2g00940 | Ortholog of A. nidulans FGSC A4 : AN10483, AN2809, AN2115, AN0033, AN12460, A. fumigatus Af293 : Afu2g16560, Afu3g15340, Afu7g00460, Afu7g06360 and A. niger CBS 513.88 : An14g02830, An16g00830 |
| Afu2g01270 | Has domain(s) with predicted oxidoreductase activity and role in oxidation-reduction process |
| Afu2g01340 | Ortholog of Aspergillus fumigatus A1163 : AFUB_018420 |
| Afu2g01405 | Ortholog of A. nidulans FGSC A4 : AN3481, A. fumigatus Af293 : Afu1g02290, A. niger CBS 513.88 : An01g05960 and A. oryzae RIB40 : AO090026000175, AO090005000871, AO090026000055 |
| Afu2g01410 | Ortholog of A. oryzae RIB40 : AO090701000824, Neosartorya fischeri NRRL 181 : NFIA_033720, Aspergillus wentii : Aspwe1_0041628 and Aspergillus versicolor : Aspve1_0090015 |
| Afu2g01450 | Alpha-1,6 mannosyltransferase subunit with a predicted role in N-linked protein glycosylation |
| Afu2g01600 | Putative sphingomyelin phosphodiesterase |
| Afu2g01770 | Has domain(s) with predicted flavin adenine dinucleotide binding, oxidoreductase activity, acting on CH-OH group of donors activity and role in oxidation-reduction process |
| Afu2g01850 | Ortholog(s) have role in cellular response to drug |
| Afu2g01860 | Ortholog of A. nidulans FGSC A4 : AN4565, A. niger CBS 513.88 : An07g05550, A. oryzae RIB40 : AO090120000188, Aspergillus wentii : Aspwe1_0041692 and Aspergillus sydowii : Aspsy1_0091128 |
| Afu2g01870 | Putative class I chitin synthase |
| Afu2g01880 | Has domain(s) with predicted protein tyrosine phosphatase activity and role in protein dephosphorylation |
| Afu2g01930 | Ortholog(s) have cytosol, nucleus localization |
| Afu2g01950 | Has domain(s) with predicted FAD binding, oxidoreductase activity and role in metabolic process |
| Afu2g02050 | Putative peptidyl-prolyl cis-trans isomerase |
| Afu2g02060 | Ortholog(s) have D-leucyl-tRNA(Leu) deacylase activity, D-tyrosyl-tRNA(Tyr) deacylase activity, role in D-leucine catabolic process, D-tyrosine catabolic process, tRNA metabolic process and cytosol, nucleus localization |
| Afu2g02070 | Ortholog(s) have cytosol localization |
| Afu2g02090 | O6-alkylguanine DNA alkyltransferase |
| Afu2g02110 | Sugar transporter |
| Afu2g02220 | Ortholog(s) have sterol 3-beta-glucosyltransferase activity, role in ascospore-type prospore membrane assembly, sterol metabolic process and cytoplasm localization |
| Afu2g02250 | Ortholog(s) have allantoinase activity and role in allantoin assimilation pathway, purine nucleobase catabolic process |
| Afu2g02270 | Ortholog(s) have structural constituent of ribosome activity |
| Afu2g02275 | Ortholog(s) have proton-transporting ATP synthase activity, rotational mechanism activity |
| Afu2g02280 | Ortholog(s) have DASH complex localization |
| Afu2g02290 | Has domain(s) with predicted methyltransferase activity and role in translation |
| Afu2g02330 | Ortholog of Aspergillus fumigatus A1163 : AFUB_019420 |
| Afu2g02450 | Ortholog of A. nidulans FGSC A4 : AN10572, A. niger CBS 513.88 : An07g06200, A. oryzae RIB40 : AO090026000558, Aspergillus wentii : Aspwe1_0353125 and Aspergillus sydowii : Aspsy1_0091260 |
| Afu2g02490 | Ortholog of A. nidulans FGSC A4 : AN0401, Aspergillus flavus NRRL 3357 : AFL2T_08730, Aspergillus versicolor : Aspve1_0035846 and Aspergillus fumigatus A1163 : AFUB_019590 |
| Afu2g02500 | Ortholog of Neosartorya fischeri NRRL 181 : NFIA_034900, Aspergillus wentii : Aspwe1_0040593, Aspergillus fumigatus A1163 : AFUB_019600 and Aspergillus clavatus NRRL 1 : ACLA_092300 |
| Afu2g02550 | Ortholog(s) have G-protein beta/gamma-subunit complex binding, actin binding activity, role in positive regulation of transcription from RNA polymerase II promoter by pheromones, protein folding and cytoplasm localization |
| Afu2g02560 | Ortholog(s) have role in establishment or maintenance of cell polarity and cytosol localization |
| Afu2g02570 | Ortholog(s) have mitochondrion localization |
| Afu2g02590 | Aspartyl-tRNA synthetase |
| Afu2g02680 | Putative matrix AAA protease |
| Afu2g02830 | Ortholog of A. nidulans FGSC A4 : AN4538, A. niger CBS 513.88 : An07g07180, A. oryzae RIB40 : AO090026000628, Aspergillus wentii : Aspwe1_0036706 and Aspergillus sydowii : Aspsy1_0091097 |
| Afu2g02880 | Ortholog(s) have cytosol localization |
| Afu2g02890 | Ortholog(s) have tRNA 2'-phosphotransferase activity, role in tRNA splicing, via endonucleolytic cleavage and ligation and cytoplasm, nucleus localization |
| Afu2g02950 | Has domain(s) with predicted oxidoreductase activity and role in oxidation-reduction process |
| Afu2g02980 | Has domain(s) with predicted ER retention sequence binding activity, role in protein retention in ER lumen and integral component of membrane localization |
| Afu2g03030 | Putative pre-mRNA splicing factor |
| Afu2g03110 | Putative alkaline phosphatase, phoB-regulated |
| Afu2g03115 | rRNA |
| Afu2g03120 | Putative cell wall glucanase |
| Afu2g03180 | Has domain(s) with predicted RNA polymerase II transcription factor activity, sequence-specific DNA binding, zinc ion binding activity, role in regulation of transcription, DNA-templated and nucleus localization |
| Afu2g03270 | Has domain(s) with predicted catalytic activity |
| Afu2g03280 | protein of unknown function |
| Afu2g03390 | Putative histone deacetylase |
| Afu2g03600 | Has domain(s) with predicted hydrolase activity |
| Afu2g03620 | Has domain(s) with predicted oxidoreductase activity and role in metabolic process |
| Afu2g03630 | Ortholog of A. nidulans FGSC A4 : AN9462, A. niger CBS 513.88 : An07g08200, Neosartorya fischeri NRRL 181 : NFIA_035990, Aspergillus wentii : Aspwe1_0110313 and Aspergillus clavatus NRRL 1 : ACLA_090870 |
| Afu2g03640 | Ortholog(s) have actin binding, calmodulin binding activity |
| Afu2g03700 | Hydroxymethylglutaryl-CoA (HMG-CoA) reductase |
| Afu2g03740 | Ortholog(s) have role in U1 snRNA 3'-end processing, U4 snRNA 3'-end processing and U5 snRNA 3'-end processing, more |
| Afu2g03810 | Putative histone deacetylase |
| Afu2g03880 | Has domain(s) with predicted heme binding activity |
| Afu2g03940 | Ortholog of Aspergillus fumigatus A1163 : AFUB_021010 |
| Afu2g04040 | Ortholog(s) have mitochondrion localization |
| Afu2g04200 | 4-hydroxyphenylpyruvate dioxygenase involved in the L-tyrosine degradation pathway |
| Afu2g04270 | Ortholog(s) have protein channel activity, role in mitochondrial genome maintenance, protein import into mitochondrial matrix and mitochondrial inner membrane presequence translocase complex localization |
| Afu2g04290 | Ortholog(s) have role in steroid metabolic process |
| Afu2g04310 | Argininosuccinate synthase |
| Afu2g04390 | Ortholog of A. nidulans FGSC A4 : AN10247, A. oryzae RIB40 : AO090003000225, Aspergillus wentii : Aspwe1_0046123 and Aspergillus terreus NIH2624 : ATET_06072 |
| Afu2g04420 | Ortholog of A. nidulans FGSC A4 : AN5126, A. fumigatus Af293 : Afu1g07490, A. niger CBS 513.88 : An11g03310, An07g10050, A. oryzae RIB40 : AO090012000999 and Aspergillus wentii : Aspwe1_0046371 |
| Afu2g04430 | Ortholog(s) have role in cellular manganese ion homeostasis, mitochondrion organization and fungal-type vacuole membrane localization |
| Afu2g04470 | Ortholog of A. nidulans FGSC A4 : AN1871, A. niger CBS 513.88 : An11g03160, A. oryzae RIB40 : AO090003000240, Aspergillus wentii : Aspwe1_0177180 and Aspergillus sydowii : Aspsy1_0140729 |
| Afu2g04533 | Ortholog of A. niger CBS 513.88 : An11g03770, A. oryzae RIB40 : AO090003000245, Aspergillus versicolor : Aspve1_0125145 and Aspergillus clavatus NRRL 1 : ACLA_090070 |
| Afu2g04680 | Protein serine/threonine kinase |
| Afu2g04800 | Ortholog(s) have role in maturation of SSU-rRNA from tricistronic rRNA transcript (SSU-rRNA, 5.8S rRNA, LSU-rRNA) and cytoplasm, nucleus localization |
| Afu2g04810 | protein of unknown function |
| Afu2g04820 | Putative translation release factor eRF3 |
| Afu2g04840 | Ortholog of A. nidulans FGSC A4 : AN2082, A. niger CBS 513.88 : An11g04530, A. oryzae RIB40 : AO090003000287, Aspergillus wentii : Aspwe1_0120888 and Aspergillus sydowii : Aspsy1_0127836 |
| Afu2g04850 | Ortholog of A. nidulans FGSC A4 : AN2083, A. niger CBS 513.88 : An11g04540, A. oryzae RIB40 : AO090003000288, Aspergillus wentii : Aspwe1_0046199 and Aspergillus sydowii : Aspsy1_0039236 |
| Afu2g04880 | Ortholog of A. nidulans FGSC A4 : AN10243, A. fumigatus Af293 : Afu4g09900, A. oryzae RIB40 : AO090003001243, Aspergillus wentii : Aspwe1_0072976, Aspwe1_0166814 and Aspergillus sydowii : Aspsy1_0141212 |
| Afu2g04930 | Protein of unknown function identified by mass spectrometry |
| Afu2g04940 | Ortholog(s) have mRNA binding activity, role in telomere maintenance and cytoplasm, nucleus localization |
| Afu2g05040 | Protein involved in glycosylphosphatidylinositol (GPI)-anchor biosynthesis and remodeling |
| Afu2g05060 | Alternative oxidase, mediates the cyanide-insensitive respiratory pathway |
| Afu2g05070 | Ortholog of A. nidulans FGSC A4 : AN2100, A. niger CBS 513.88 : An11g04820, Aspergillus wentii : Aspwe1_0120231, Aspergillus sydowii : Aspsy1_0054017 and Aspergillus terreus NIH2624 : ATET_05993 |
| Afu2g05290 | Ortholog of A. nidulans FGSC A4 : AN3976, A. niger CBS 513.88 : An02g11140, Neosartorya fischeri NRRL 181 : NFIA_082050, Aspergillus wentii : Aspwe1_0592927 and Aspergillus versicolor : Aspve1_0080485 |
| Afu2g05340 | Essential 1,3-beta-glucanosyltransferase, GPI-anchored to the plasma membrane |
| Afu2g05450 | Mitochondrial NADH dehydrogenase involved in oxidative phosphorylation |
| Afu2g05470 | Ortholog(s) have role in attachment of mitotic spindle microtubules to kinetochore, cohesin localization to chromatin, mitotic sister chromatid cohesion and negative regulation of G0 to G1 transition, more |
| Afu2g05480 | Ortholog(s) have RNA polymerase I activity and role in termination of RNA polymerase I transcription, transcription of nuclear large rRNA transcript from RNA polymerase I promoter |
| Afu2g05540 | Ortholog(s) have DNA binding activity, role in mRNA cis splicing, via spliceosome and Prp19 complex, cytosol, spliceosomal complex localization |
| Afu2g05560 | Putative exonuclease |
| Afu2g05590 | Ortholog(s) have ubiquitin-specific protease activity, role in protein deubiquitination and cytosol, nucleus localization |
| Afu2g05600 | Ortholog(s) have cytosol localization |
| Afu2g05630 | Ortholog of A. nidulans FGSC A4 : AN7476, A. niger CBS 513.88 : An02g14010, A. oryzae RIB40 : AO090001000675, Aspergillus wentii : Aspwe1_0120551 and Aspergillus sydowii : Aspsy1_0058449 |
| Afu2g05680 | Ortholog of A. nidulans FGSC A4 : AN7481, A. niger CBS 513.88 : An02g14110, A. oryzae RIB40 : AO090001000682, Aspergillus wentii : Aspwe1_0055195 and Aspergillus sydowii : Aspsy1_0153107 |
| Afu2g05700 | Ortholog of A. oryzae RIB40 : AO090001000687, Neosartorya fischeri NRRL 181 : NFIA_082420, Aspergillus clavatus NRRL 1 : ACLA_068640 and Aspergillus sydowii : Aspsy1_0090059 |
| Afu2g05730 | Putative siderophore transporter |
| Afu2g05830 | Putative transcription factor involved in regulation of gluconeogenesis and acquisition of iron |
| Afu2g05850 | Ortholog(s) have role in double-strand break repair via homologous recombination, mitotic sister chromatid cohesion and horsetail nucleus leading edge, nuclear mitotic cohesin complex localization |
| Afu2g06060 | Ortholog(s) have transcription coactivator activity, role in histone acetylation, transcription from RNA polymerase II promoter and Ada2/Gcn5/Ada3 transcription activator complex, SAGA complex, SLIK (SAGA-like) complex localization |
| Afu2g06280 | Oligosaccharyl transferase gamma subunit with a predicted role in N-linked protein glycosylation |
| Afu2g07510 | Ortholog of A. nidulans FGSC A4 : AN5811, A. niger CBS 513.88 : An05g00060, A. oryzae RIB40 : AO090011000941, Neosartorya fischeri NRRL 181 : NFIA_083250 and Aspergillus wentii : Aspwe1_0043750 |
| Afu2g07520 | Ortholog(s) have aminopeptidase activity, epoxide hydrolase activity, role in cellular lipid metabolic process, protein catabolic process and cytosol, extracellular region, nucleus localization |
| Afu2g07730 | Has domain(s) with predicted phospholipid binding activity, role in cortical protein anchoring and cell cortex localization |
| Afu2g07780 | Has domain(s) with predicted catalytic activity and role in metabolic process |
| Afu2g07830 | Ortholog of Neosartorya fischeri NRRL 181 : NFIA_083540, Aspergillus fumigatus A1163 : AFUB_023860 and Aspergillus clavatus NRRL 1 : ACLA_079800 |
| Afu2g07860 | Ortholog of Aspergillus fumigatus A1163 : AFUB_023880 |
| Afu2g07870 | Patatin-like serine hydrolase |
| Afu2g07950 | Ortholog(s) have cytosol localization |
| Afu2g08040 | C6 finger domain protein |
| Afu2g08050 | Has domain(s) with predicted oxidoreductase activity and role in metabolic process |
| Afu2g08100 | Ortholog of Aspergillus fumigatus A1163 : AFUB_024119 |
| Afu2g08110 | Has domain(s) with predicted ATP binding, glucose binding, hexokinase activity, phosphotransferase activity, alcohol group as acceptor activity |
| Afu2g08240 | Ortholog(s) have sequence-specific DNA binding activity and nucleus localization |
| Afu2g08350 | Ortholog(s) have Golgi apparatus, cell division site, cell tip, endoplasmic reticulum localization |
| Afu2g08360 | Orotidine 5'-monophosphate decarboxylase |
| Afu2g08370 | Predicted glutathione S transferase |
| Afu2g08400 | Ortholog of S. cerevisiae : YPR153W, A. niger CBS 513.88 : An12g03630, Aspergillus wentii : Aspwe1_0119895, Aspergillus versicolor : Aspve1_0634371 and Aspergillus clavatus NRRL 1 : ACLA_080230 |
| Afu2g08410 | Ortholog of A. nidulans FGSC A4 : AN6161, A. niger CBS 513.88 : An12g03650, A. oryzae RIB40 : AO090011000861, Aspergillus wentii : Aspwe1_0031690 and Aspergillus sydowii : Aspsy1_0089578 |
| Afu2g08450 | Ortholog(s) have transcription factor TFIIE complex localization |
| Afu2g08460 | Ortholog(s) have mRNA binding, pseudouridine synthase activity, role in mRNA pseudouridine synthesis, snRNA pseudouridine synthesis, tRNA pseudouridine synthesis and nucleus localization |
| Afu2g08630 | Ortholog(s) have endoplasmic reticulum localization |
| Afu2g08670 | Ortholog(s) have acetyl-CoA carboxylase activity, biotin carboxylase activity and role in long-chain fatty acid biosynthetic process, protein import into nucleus |
| Afu2g08720 | Ortholog(s) have role in ER to Golgi vesicle-mediated transport, protein complex assembly and TRAPPI protein complex, TRAPPII protein complex, TRAPPIII protein complex, cytosol, nucleus localization |
| Afu2g08730 | Ortholog of A. nidulans FGSC A4 : AN6123, A. niger CBS 513.88 : An12g04060, A. oryzae RIB40 : AO090011000832, Aspergillus wentii : Aspwe1_0677357 and Aspergillus sydowii : Aspsy1_0058030 |
| Afu2g08775 | Ortholog of Neosartorya fischeri NRRL 181 : NFIA_084360, Aspergillus wentii : Aspwe1_0176138, Aspergillus fumigatus A1163 : AFUB_024680 and Aspergillus clavatus NRRL 1 : ACLA_080620 |
| Afu2g08850 | Ortholog(s) have phospholipid-translocating ATPase activity |
| Afu2g08870 | Ortholog(s) have RNA binding activity, role in mRNA cis splicing, via spliceosome and Prp19 complex, spliceosomal complex localization |
| Afu2g08930 | Predicted DDE1 transposon-related ORF |
| Afu2g09090 | Ortholog(s) have role in mitochondrion inheritance, negative regulation of proteolysis, protein folding, replicative cell aging and mitochondrial inner membrane, peroxisomal importomer complex, plasma membrane localization |
| Afu2g09100 | Ortholog(s) have role in nuclear-transcribed mRNA catabolic process, meiosis-specific transcripts and nucleus localization |
| Afu2g09150 | Ortholog(s) have adenine deaminase activity, role in adenine catabolic process, hypoxanthine salvage and cytosol, nucleus localization |
| Afu2g09210 | 60S ribosomal protein L10 |
| Afu2g09282 | Has domain(s) with predicted metal ion transmembrane transporter activity, role in metal ion transport, transmembrane transport and membrane localization |
| Afu2g09340 | Ortholog of A. oryzae RIB40 : AO090011000758 and Aspergillus fumigatus A1163 : AFUB_025200 |
| Afu2g09500 | Has domain(s) with predicted role in response to stress |
| Afu2g09550 | Ortholog(s) have biotin-[acetyl-CoA-carboxylase] ligase activity, biotin-[pyruvate-carboxylase] ligase activity, role in protein biotinylation and cytoplasm, nucleus localization |
| Afu2g09590 | UDP-N-acetylglucosamine 1-carboxyvinyltransferase family protein |
| Afu2g09700 | Ortholog(s) have superoxide dismutase copper chaperone activity, role in cellular copper ion homeostasis, cellular response to metal ion, intracellular copper ion transport and cytosol, mitochondrial inner membrane, nucleus localization |
| Afu2g09820 | Ortholog(s) have role in protein import into mitochondrial matrix and mitochondrial inner membrane presequence translocase complex, plasma membrane localization |
| Afu2g09870 | Eukaryotic translation initiation factor 3 subunit G |
| Afu2g10030 | Immunoreactive protein |
| Afu2g10090 | 40S ribosomal protein S15 |
| Afu2g10270 | Tor kinase, involved in regulation cell wall biosynthesis and nutrient sensing |
| Afu2g10280 | Ortholog(s) have carbonyl reductase (NADPH) activity and cytosol, nucleus localization |
| Afu2g10290 | Ortholog(s) have mitochondrial ribosome localization |
| Afu2g10310 | Ortholog of A. nidulans FGSC A4 : AN5980, A. niger CBS 513.88 : An16g04680, A. oryzae RIB40 : AO090011000607, Aspergillus wentii : Aspwe1_0116525 and Aspergillus sydowii : Aspsy1_0045000 |
| Afu2g10340 | Ortholog(s) have role in filamentous growth and cytosol, mitotic spindle pole body, nucleus localization |
| Afu2g10370 | Has domain(s) with predicted iron-sulfur cluster binding, structural molecule activity and role in iron-sulfur cluster assembly |
| Afu2g10375 | rRNA |
| Afu2g10420 | Has domain(s) with predicted catalytic activity and role in metabolic process |
| Afu2g10600 | Putative NADH dehydrogenase |
| Afu2g10690 | Putative MFS phosphate transporter |
| Afu2g10720 | Ortholog of A. nidulans FGSC A4 : AN5933, A. niger CBS 513.88 : An02g02500, Aspergillus wentii : Aspwe1_0061884, Aspergillus sydowii : Aspsy1_0044922 and Aspergillus terreus NIH2624 : ATET_01504 |
| Afu2g10770 | putative transcription factor |
| Afu2g10800 | Ortholog(s) have endoplasmic reticulum localization |
| Afu2g10880 | Ortholog of A. nidulans FGSC A4 : AN12158, A. niger CBS 513.88 : An02g02730, A. oryzae RIB40 : AO090011000542, Neosartorya fischeri NRRL 181 : NFIA_086200 and Aspergillus wentii : Aspwe1_0044185 |
| Afu2g10890 | Ortholog(s) have cytosol localization |
| Afu2g11080 | Ortholog(s) have endoplasmic reticulum localization |
| Afu2g11090 | Ortholog of A. nidulans FGSC A4 : AN5901, A. niger CBS 513.88 : An02g02990, A. oryzae RIB40 : AO090026000549, Aspergillus wentii : Aspwe1_0044210 and Aspergillus sydowii : Aspsy1_0152482 |
| Afu2g11130 | Has domain(s) with predicted role in regulation of transcription, DNA-templated and nucleus localization |
| Afu2g11140 | Ortholog(s) have structural constituent of ribosome activity and mitochondrial large ribosomal subunit localization |
| Afu2g11290 | Orotate phosphoribosyltransferase 1 |
| Afu2g11330 | Ortholog(s) have role in cellular metal ion homeostasis, cellular response to biotic stimulus and cellular response to starvation, more |
| Afu2g11350 | Has domain(s) with predicted catalytic activity, transferase activity, transferring acyl groups other than amino-acyl groups activity and role in metabolic process |
| Afu2g11360 | Has domain(s) with predicted catalytic activity and role in metabolic process |
| Afu2g11440 | Putative proteasome component |
| Afu2g11460 | C6 finger domain protein |
| Afu2g11470 | Ortholog of A. nidulans FGSC A4 : AN10752, A. niger CBS 513.88 : An02g03450, Neosartorya fischeri NRRL 181 : NFIA_086710, Aspergillus wentii : Aspwe1_0044288 and Aspergillus versicolor : Aspve1_0040617 |
| Afu2g11475 | Ortholog(s) have role in endocytosis, establishment or maintenance of actin cytoskeleton polarity, vacuole organization and integral component of Golgi membrane localization |
| Afu2g11540 | Ortholog(s) have ketoreductase activity, role in fatty acid elongation, sphingolipid biosynthetic process, very long-chain fatty acid biosynthetic process and endoplasmic reticulum membrane localization |
| Afu2g11550 | Putative ergosterol biosynthesis protein |
| Afu2g11610 | Has domain(s) with predicted catalytic activity, cation binding activity and role in carbohydrate metabolic process |
| Afu2g11640 | Ortholog of A. nidulans FGSC A4 : AN12049, A. niger CBS 513.88 : An02g03700, Aspergillus wentii : Aspwe1_0044384, Aspergillus sydowii : Aspsy1_0058083 and Aspergillus terreus NIH2624 : ATET_01091 |
| Afu2g11660 | Ortholog(s) have role in mismatch repair, nucleotide-excision repair and nucleolus localization |
| Afu2g11740 | Ortholog(s) have ATP-dependent peptidase activity, role in chaperone-mediated protein complex assembly, misfolded or incompletely synthesized protein catabolic process and mitochondrial matrix localization |
| Afu2g11780 | Ortholog(s) have sequence-specific DNA binding, transcription factor activity, sequence-specific DNA binding, transcription regulatory region DNA binding activity |
| Afu2g11850 | Allergenic ribosomal L3 protein |
| Afu2g11910 | Ortholog of Aspergillus fumigatus A1163 : AFUB_027650 |
| Afu2g11920 | Ortholog of A. nidulans FGSC A4 : AN6208, A. niger CBS 513.88 : An02g04010, A. oryzae RIB40 : AO090026000451, Aspergillus wentii : Aspwe1_0658676 and Aspergillus sydowii : Aspsy1_0202684 |
| Afu2g11990 | Putative pten 3-phosphoinositide phosphatase alpha with a predicted role in lipid metabolism |
| Afu2g12110 | Ortholog(s) have cytoplasm localization |
| Afu2g12130 | Ortholog(s) have cytosol, trimeric positive transcription elongation factor complex b localization |
| Afu2g12140 | Has domain(s) with predicted ion channel activity, voltage-gated chloride channel activity, role in chloride transport, transmembrane transport and membrane localization |
| Afu2g12200 | cAMP-dependent protein kinase catalytic subunit |
| Afu2g12380 | Ortholog of A. nidulans FGSC A4 : AN6289, A. niger CBS 513.88 : An02g04500, A. oryzae RIB40 : AO090026000370, Aspergillus wentii : Aspwe1_0044464 and Aspergillus sydowii : Aspsy1_0151634 |
| Afu2g12460 | Ortholog(s) have role in mitochondrial respiratory chain complex II assembly and mitochondrial matrix localization |
| Afu2g12480 | Ortholog(s) have role in actin cortical patch localization, arginine transport, cellular protein localization, endocytosis, late endosome to vacuole transport and lysine transport, more |
| Afu2g12540 | Ortholog of A. nidulans FGSC A4 : AN6278, Neosartorya fischeri NRRL 181 : NFIA_087680, Aspergillus wentii : Aspwe1_0071673 and Aspergillus versicolor : Aspve1_0041153 |
| Afu2g12550 | Has domain(s) with predicted role in transmembrane transport and integral component of membrane localization |
| Afu2g12560 | Has domain(s) with predicted 4-alpha-hydroxytetrahydrobiopterin dehydratase activity and role in tetrahydrobiopterin biosynthetic process |
| Afu2g12590 | Ortholog of S. cerevisiae : MTC6, A. nidulans FGSC A4 : AN6270, A. niger CBS 513.88 : An02g01520, Aspergillus wentii : Aspwe1_0031301 and Aspergillus sydowii : Aspsy1_0089695 |
| Afu2g12600 | Ortholog of A. nidulans FGSC A4 : AN6271, A. niger CBS 513.88 : An02g01530, A. oryzae RIB40 : AO090026000355, Aspergillus wentii : Aspwe1_0175672 and Aspergillus sydowii : Aspsy1_0152443 |
| Afu2g12640 | Putative G-protein coupled receptor (GPCR)-like protein |
| Afu2g12660 | Has domain(s) with predicted role in isoprenoid biosynthetic process |
| Afu2g12820 | Has domain(s) with predicted phosphopantetheine binding activity |
| Afu2g13030 | Ortholog(s) have phenylalanine-tRNA ligase activity, role in mitochondrial phenylalanyl-tRNA aminoacylation and mitochondrion localization |
| Afu2g13090 | Ortholog(s) have DNA binding, RNA polymerase II activity, RNA-directed RNA polymerase activity, role in response to drug, termination of RNA polymerase II transcription and DNA-directed RNA polymerase II, core complex, cytosol localization |
| Afu2g13190 | Has domain(s) with predicted hydrolase activity, hydrolyzing O-glycosyl compounds activity and role in carbohydrate metabolic process |
| Afu2g13230 | Has domain(s) with predicted role in response to stress |
| Afu2g13245 | unspecified product |
| Afu2g13260 | Putative regulator of adherence, host cell interactions and virulence |
| Afu2g13265 | Ortholog of A. niger CBS 513.88 : An02g02070, A. oryzae RIB40 : AO090026000287, Neosartorya fischeri NRRL 181 : NFIA_088440 and Aspergillus wentii : Aspwe1_0044574 |
| Afu2g13280 | Ortholog of Aspergillus wentii : Aspwe1_0044577 and Aspergillus fumigatus A1163 : AFUB_028920 |
| Afu2g13290 | GYF domain protein |
| Afu2g13360 | Has domain(s) with predicted ATP binding, DNA binding, helicase activity, nucleic acid binding activity |
| Afu2g13380 | Ortholog(s) have RNA polymerase II transcription factor activity, sequence-specific DNA binding, sequence-specific DNA binding activity |
| Afu2g13512 | Ortholog of A. nidulans FGSC A4 : AN6328, A. niger CBS 513.88 : An02g05750, A. oryzae RIB40 : AO090026000262, Aspergillus wentii : Aspwe1_0163563 and Aspergillus sydowii : Aspsy1_0997059 |
| Afu2g13520 | Ortholog(s) have role in fermentation, protein maturation and mitochondrion localization |
| Afu2g13530 | Putative translation elongation factor EF-2 subunit |
| Afu2g13560 | Ortholog of A. nidulans FGSC A4 : AN6333, A. niger CBS 513.88 : An02g05660, Neosartorya fischeri NRRL 181 : NFIA_088760, Aspergillus wentii : Aspwe1_0062647 and Aspergillus versicolor : Aspve1_0131011 |
| Afu2g13640 | Has domain(s) with predicted ATP binding, protein kinase activity, protein tyrosine kinase activity and role in protein phosphorylation |
| Afu2g13660 | Ortholog of A. nidulans FGSC A4 : AN2411, A. niger CBS 513.88 : An02g05520, A. oryzae RIB40 : AO090026000242, Aspergillus wentii : Aspwe1_0045469 and Aspergillus sydowii : Aspsy1_0341196 |
| Afu2g13730 | Ortholog(s) have cytosol, nucleus localization |
| Afu2g13920 | Ortholog of A. nidulans FGSC A4 : AN4291, Aspergillus tubingensis : Asptu1_0084419, Aspergillus brasiliensis : Aspbr1_0043823 and Aspergillus acidus : Aspfo1_0097914 |
| Afu2g13950 | Ortholog of Aspergillus fumigatus A1163 : AFUB_029580 |
| Afu2g14040 | Has domain(s) with predicted phosphatidylinositol phosphate kinase activity and role in phosphatidylinositol metabolic process |
| Afu2g14060 | Ortholog(s) have endoplasmic reticulum, plasma membrane localization |
| Afu2g14080 | Ortholog(s) have role in mitotic chromosome condensation, mitotic sister chromatid cohesion, positive regulation of maintenance of mitotic sister chromatid cohesion, centromeric and cytosol, nuclear mitotic cohesin complex localization |
| Afu2g14090 | Ortholog(s) have cytosol localization |
| Afu2g14140 | Ortholog of Aspergillus fumigatus A1163 : AFUB_029760 |
| Afu2g14180 | Ortholog(s) have role in tRNA methylation and cytosol, nucleus localization |
| Afu2g14370 | Protein of unknown function identified by mass spectrometry |
| Afu2g14380 | Ortholog of A. nidulans FGSC A4 : AN12119, AN12185, A. niger CBS 513.88 : An03g05760, Neosartorya fischeri NRRL 181 : NFIA_089550 and Aspergillus wentii : Aspwe1_0035911, Aspwe1_0045304, Aspwe1_0065659 |
| Afu2g14390 | Ortholog of A. nidulans FGSC A4 : AN2405, A. oryzae RIB40 : AO090023000066, Neosartorya fischeri NRRL 181 : NFIA_089560, Aspergillus wentii : Aspwe1_0046658 and Aspergillus versicolor : Aspve1_0025636 |
| Afu2g14400 | Ortholog of A. nidulans FGSC A4 : AN1725, AN5027, A. fumigatus Af293 : Afu3g12130 and A. niger CBS 513.88 : An11g08400, An02g07940, An12g01310, An02g00780 |
| Afu2g14560 | Has domain(s) with predicted catalytic activity, lyase activity and role in metabolic process |
| Afu2g14590 | MFS monosaccharide transporter |
| Afu2g14600 | Ortholog(s) have sterol esterase activity, role in cell wall mannoprotein biosynthetic process, sterol metabolic process and integral component of membrane, plasma membrane localization |
| Afu2g14650 | Has domain(s) with predicted ATP binding, protein kinase activity and role in protein phosphorylation |
| Afu2g14720 | Sequence-specific CCAAT DNA binding transcription factor |
| Afu2g14730 | Ortholog(s) have role in mitochondrion transport along microtubule, vesicle transport along microtubule and cytoplasmic microtubule, cytoplasmic vesicle, early endosome localization |
| Afu2g14750 | Putative endo-arabinase |
| Afu2g14780 | Ortholog of A. niger CBS 513.88 : An15g03500, Neosartorya fischeri NRRL 181 : NFIA_090010, Aspergillus wentii : Aspwe1_0693082 and Aspergillus clavatus NRRL 1 : ACLA_072700 |
| Afu2g14810 | Ortholog(s) have peptidyl-proline dioxygenase activity |
| Afu2g14820 | Ortholog of A. niger CBS 513.88 : An15g03410, A. oryzae RIB40 : AO090026000793, Aspergillus wentii : Aspwe1_0693332 and Aspergillus sydowii : Aspsy1_0045989 |
| Afu2g14890 | Ortholog(s) have cytosol, nucleus localization |
| Afu2g14920 | Ortholog(s) have role in DNA damage checkpoint and nucleus localization |
| Afu2g14930 | Ortholog(s) have L-allo-threonine aldolase activity, role in carnitine biosynthetic process, glycine biosynthetic process, threonine catabolic process and cytosol, nucleus localization |
| Afu2g14940 | Ortholog of A. nidulans FGSC A4 : AN7565, A. niger CBS 513.88 : An15g03240, A. oryzae RIB40 : AO090012000409, Aspergillus wentii : Aspwe1_0693532 and Aspergillus sydowii : Aspsy1_1021602 |
| Afu2g15110 | Ortholog of A. nidulans FGSC A4 : AN7580, A. niger CBS 513.88 : An15g02960, A. oryzae RIB40 : AO090012000329, Aspergillus wentii : Aspwe1_0044605 and Aspergillus sydowii : Aspsy1_0046014 |
| Afu2g15120 | Ortholog of Aspergillus fumigatus A1163 : AFUB_030780 |
| Afu2g15350 | Ortholog of Aspergillus fumigatus A1163 : AFUB_031010 |
| Afu2g15380 | Ortholog of Aspergillus fumigatus A1163 : AFUB_031040 |
| Afu2g15390 | Has domain(s) with predicted catalytic activity, glutamate-ammonia ligase activity and role in nitrogen compound metabolic process |
| Afu2g15420 | Ortholog(s) have extracellular region localization |
| Afu2g15470 | Ortholog of A. nidulans FGSC A4 : AN3207, AN1505, A. fumigatus Af293 : Afu8g00720, Afu8g05070 and A. niger CBS 513.88 : An07g04980, An16g07680, An14g02720, An14g07130, An15g05540 |
| Afu2g15480 | Ortholog of A. nidulans FGSC A4 : AN7607, A. niger CBS 513.88 : An15g05550, An12g07620, A. oryzae RIB40 : AO090012000281, AO090020000707 and Aspergillus wentii : Aspwe1_0025841, Aspwe1_0037842, Aspwe1_0044672 |
| Afu2g15510 | Ortholog(s) have cytosol, nucleus localization |
| Afu2g15550 | Ortholog(s) have nitrate reductase (NADPH) activity and role in Mo-molybdopterin cofactor biosynthetic process |
| Afu2g15570 | Ortholog(s) have GTP binding, GTPase activity, role in hyphal growth, intracellular protein transport, retrograde transport, endosome to Golgi and Golgi membrane, cytosol, nucleus, spitzenkorper localization |
| Afu2g15590 | Putative sulphite reductase |
| Afu2g15820 | Ortholog(s) have role in Golgi to vacuole transport, vacuole fusion and Golgi trans cisterna, endoplasmic reticulum, late endosome localization |
| Afu2g15905 | Ortholog of A. nidulans FGSC A4 : AN2160, A. niger CBS 513.88 : An15g06220, A. oryzae RIB40 : AO090012000216, Aspergillus wentii : Aspwe1_0170981 and Aspergillus sydowii : Aspsy1_0084996 |
| Afu2g15910 | Ortholog(s) have alpha-1,6-mannosyltransferase activity, role in protein N-linked glycosylation and alpha-1,6-mannosyltransferase complex, endoplasmic reticulum localization |
| Afu2g15990 | Ortholog of A. nidulans FGSC A4 : AN2152, AN0341, A. fumigatus Af293 : Afu1g02260, A. niger CBS 513.88 : An01g06000 and Aspergillus wentii : Aspwe1_0025648, Aspwe1_0026895 |
| Afu2g16010 | Prolyl-tRNA synthetase |
| Afu2g16140 | Ortholog(s) have cytosol localization |
| Afu2g16210 | Ortholog(s) have fluoride transmembrane transporter activity, role in fluoride transmembrane transport and endoplasmic reticulum, plasma membrane localization |
| Afu2g16360 | Ortholog(s) have 2,5-diamino-6-ribitylamino-4(3H)-pyrimidinone 5'-phosphate deaminase activity, pseudouridine synthase activity, role in riboflavin biosynthetic process, tRNA pseudouridine synthesis and cytosol localization |
| Afu2g16420 | Ortholog of A. nidulans FGSC A4 : AN7348, A. niger CBS 513.88 : An15g06820, A. oryzae RIB40 : AO090102000221, Aspergillus wentii : Aspwe1_0108539 and Aspergillus sydowii : Aspsy1_0027034 |
| Afu2g16470 | Has domain(s) with predicted acid-amino acid ligase activity |
| Afu2g16480 | Has domain(s) with predicted zinc ion binding activity |
| Afu2g16490 | Ortholog(s) have role in filamentous growth of a population of unicellular organisms, negative regulation of filamentous growth of a population of unicellular organisms |
| Afu2g16560 | Ortholog of A. nidulans FGSC A4 : AN10483, AN2809, AN2115, AN0033, AN12460, A. fumigatus Af293 : Afu2g00940, Afu3g15340, Afu7g00460, Afu7g06360 and A. niger CBS 513.88 : An14g02830, An16g00830 |
| Afu2g16590 | Ortholog of Neosartorya fischeri NRRL 181 : NFIA_091880, Aspergillus fumigatus A1163 : AFUB_032270 and Aspergillus clavatus NRRL 1 : ACLA_075490 |
| Afu2g16730 | Ortholog(s) have ubiquitin-protein transferase activity, role in protein import into peroxisome matrix, protein monoubiquitination and integral component of peroxisomal membrane, peroxisomal importomer complex localization |
| Afu2g16750 | Putative nonsense-mediated mRNA decay protein |
| Afu2g16820 | Putative curved DNA-binding protein |
| Afu2g16830 | Ortholog(s) have extracellular region localization |
| Afu2g16900 | Has domain(s) with predicted catalytic activity |
| Afu2g16910 | Ortholog of A. nidulans FGSC A4 : AN7291, A. oryzae RIB40 : AO090102000654, Aspergillus wentii : Aspwe1_0171092, Aspergillus sydowii : Aspsy1_0027159 and Aspergillus terreus NIH2624 : ATET_10094 |
| Afu2g16950 | Transcript up-regulated in conidia exposed to neutrophils |
| Afu2g16990 | Ortholog of A. nidulans FGSC A4 : AN7273, A. oryzae RIB40 : AO090102000119, Aspergillus wentii : Aspwe1_0182487, Aspergillus sydowii : Aspsy1_0027170 and Aspergillus terreus NIH2624 : ATET_10043 |
| Afu2g17050 | Ortholog(s) have chromatin binding activity and role in DNA replication initiation, pre-replicative complex assembly involved in nuclear cell cycle DNA replication, rRNA processing, response to drug |
| Afu2g17110 | Putative cell division control protein |
| Afu2g17140 | protein of unknown function |
| Afu2g17210 | Ortholog of Aspergillus fumigatus A1163 : AFUB_032860 |
| Afu2g17270 | Has domain(s) with predicted role in transmembrane transport and integral component of membrane localization |
| Afu2g17305 | Ortholog of Aspergillus fumigatus A1163 : AFUB_032960 |
| Afu2g17320 | Ortholog of A. nidulans FGSC A4 : AN7535, A. oryzae RIB40 : AO090023000622, Neosartorya fischeri NRRL 181 : NFIA_092740, Aspergillus wentii : Aspwe1_0022988 and Aspergillus fumigatus A1163 : AFUB_032980 |
| Afu2g17420 | NB-ARC domain protein |
| Afu2g17520 | Ortholog of Neosartorya fischeri NRRL 181 : NFIA_092940, Aspergillus fumigatus A1163 : AFUB_033210, Aspergillus zonatus : Aspzo1_0135217 and Aspergillus terreus NIH2624 : ATET_09746 |
| Afu2g17530 | Laccase involved in conidial pigment biosynthesis |
| Afu2g17550 | Conidial pigment biosynthesis protein with a role in polyketide shortening |
| Afu2g17560 | 1,3,6,8-tetrahydroxynaphthalene reductase involved in conidial pigment biosynthesis |
| Afu2g17580 | Scytalone dehydratase involved in conidial pigment biosynthesis |
| Afu2g17800 | Has domain(s) with predicted RNA polymerase II transcription factor activity, sequence-specific DNA binding, zinc ion binding activity, role in regulation of transcription, DNA-templated and nucleus localization |
| Afu2g17895 | Has domain(s) with predicted DNA binding, zinc ion binding activity, role in transcription, DNA-templated and nucleus localization |
| Afu2g17900 | Ortholog of A. oryzae RIB40 : AO090003000473, Neosartorya fischeri NRRL 181 : NFIA_003780, NFIA_093330, Aspergillus niger ATCC 1015 : 194898-mRNA and Aspergillus tubingensis : Asptu1_0189799 |
| Afu2g17920 | Ortholog of A. nidulans FGSC A4 : AN8550, A. niger CBS 513.88 : An02g13300, Neosartorya fischeri NRRL 181 : NFIA_093360, Aspergillus wentii : Aspwe1_0050028 and Aspergillus versicolor : Aspve1_0187913 |
| Afu2g18040 | Putative 4-dimethylallyltryptophan synthase, involved in the production of ergot alkaloids |
| Afu2g18060 | Putative N-methyltransferase that catalyzes the N-methylation of 4-dimethylallyltryptophan in the presence of S-adenosylmethionine |
| Afu3g00100 | Has domain(s) with predicted oxidoreductase activity and role in oxidation-reduction process |
| Afu3g00270 | Cell wall glucanase |
| Afu3g00605 | protein of unknown function |
| Afu3g00610 | Has domain(s) with predicted catalytic activity, glucan 1,4-alpha-glucosidase activity, hydrolase activity, hydrolyzing O-glycosyl compounds, starch binding activity and role in polysaccharide metabolic process |
| Afu3g00670 | Ortholog of A. nidulans FGSC A4 : AN6861/phbB, AN9483, A. fumigatus Af293 : Afu5g13060, Afu8g01120, A. niger CBS 513.88 : An03g01130, An07g06330, An07g00905 and A. oryzae RIB40 : AO090120000428, AO090113000089 |
| Afu3g00810 | Putative cholestenol delta-isomerase |
| Afu3g00850 | Transcript up-regulated in conidia exposed to neutrophils |
| Afu3g00950 | Ortholog of Neosartorya fischeri NRRL 181 : NFIA_001790 and Aspergillus fumigatus A1163 : AFUB_047450 |
| Afu3g00960 | Ortholog(s) have extracellular region localization |
| Afu3g01100 | Has domain(s) with predicted nucleic acid binding activity |
| Afu3g01230 | Has domain(s) with predicted substrate-specific transmembrane transporter activity, transmembrane transporter activity, role in transmembrane transport and integral component of membrane, membrane localization |
| Afu3g01250 | Has domain(s) with predicted oxidoreductase activity and role in metabolic process |
| Afu3g01330 | Class II aldolase/adducin domain protein |
| Afu3g01530 | Putative phospholipase C |
| Afu3g01560 | Putative amino acid permease |
| Afu3g01670 | Putative major facilitator superfamily (MFS) hexose transporter |
| Afu3g01790 | Ortholog(s) have role in cellular response to oxidative stress |
| Afu3g01860 | Ortholog of A. nidulans FGSC A4 : AN8924, Neosartorya fischeri NRRL 181 : NFIA_002940, Aspergillus versicolor : Aspve1_0047531 and Aspergillus clavatus NRRL 1 : ACLA_075960 |
| Afu3g01910 | Putative cellobiohydrolase, a predicted secreted hydrolase with a fungal cellulose binding domain |
| Afu3g02040 | Has domain(s) with predicted catalytic activity |
| Afu3g02060 | Has domain(s) with predicted role in transmembrane transport and integral component of membrane localization |
| Afu3g02070 | Ortholog(s) have nucleus localization |
| Afu3g02090 | Putative secreted beta-xylosidase |
| Afu3g02230 | Ortholog of Neosartorya fischeri NRRL 181 : NFIA_003360, Aspergillus wentii : Aspwe1_0172779, Aspergillus fumigatus A1163 : AFUB_046120 and Aspergillus clavatus NRRL 1 : ACLA_062180 |
| Afu3g02253 | Ortholog of A. oryzae RIB40 : AO090012000815, Neosartorya fischeri NRRL 181 : NFIA_003390, Aspergillus clavatus NRRL 1 : ACLA_062150 and Aspergillus zonatus : Aspzo1_0135140 |
| Afu3g02390 | Ortholog(s) have role in double-strand break repair via homologous recombination, meiosis I and Smc5-Smc6 complex, cytosol, nucleus localization |
| Afu3g02530 | Protein similar to polyketide synthases (PKS-like), encoded in a predicted secondary metabolite gene cluster |
| Afu3g02540 | Ortholog of A. nidulans FGSC A4 : AN9006, A. fumigatus Af293 : Afu6g11710, Afu7g08440, Afu5g00145, A. niger CBS 513.88 : An09g05330 and A. oryzae RIB40 : AO090038000212 |
| Afu3g02570 | Putative polyketide synthase (PKS), encoded in a predicted secondary metabolite gene cluster |
| Afu3g02580 | Has domain(s) with predicted oxidoreductase activity and role in metabolic process |
| Afu3g02590 | Has domain(s) with predicted RNA polymerase II transcription factor activity, sequence-specific DNA binding, zinc ion binding activity, role in regulation of transcription, DNA-templated and nucleus localization |
| Afu3g02740 | Has domain(s) with predicted ATP binding, protein kinase activity and role in protein phosphorylation |
| Afu3g02785 | Ortholog of A. niger CBS 513.88 : An08g09080, A. oryzae RIB40 : AO090005000585, Neosartorya fischeri NRRL 181 : NFIA_004370 and Aspergillus niger ATCC 1015 : 38013-mRNA |
| Afu3g02790 | Ortholog of A. nidulans FGSC A4 : AN8593, A. fumigatus Af293 : Afu4g00460, A. niger CBS 513.88 : An11g01410 and A. oryzae RIB40 : AO090009000455, AO090138000169 |
| Afu3g02880 | Ortholog of A. nidulans FGSC A4 : AN2631, A. niger CBS 513.88 : An08g09140, An12g05700, A. oryzae RIB40 : AO090001000105 and Aspergillus wentii : Aspwe1_0137125, Aspwe1_0169838, Aspwe1_0734610 |
| Afu3g02950 | Has domain(s) with predicted oxidoreductase activity and role in metabolic process |
| Afu3g02970 | Aspergillopepsin II |
| Afu3g03040 | Ortholog of A. oryzae RIB40 : AO090010000310, Aspergillus wentii : Aspwe1_0294100, Aspwe1_0531408, Aspergillus niger ATCC 1015 : 53802-mRNA and Aspergillus terreus NIH2624 : ATET_10090 |
| Afu3g03090 | Predicted DDE1 transposon-related ORF |
| Afu3g03230 | Has domain(s) with predicted sequence-specific DNA binding, transcription factor activity, sequence-specific DNA binding activity and role in regulation of transcription, DNA-templated |
| Afu3g03280 | Putative FAD binding monooxygenase |
| Afu3g03315 | Ortholog(s) have RNA polymerase II core promoter proximal region sequence-specific DNA binding, transcriptional activator activity, RNA polymerase II core promoter proximal region sequence-specific binding activity |
| Afu3g03500 | Putative multidrug resistance protein |
| Afu3g03530 | Has domain(s) with predicted oxidoreductase activity |
| Afu3g03540 | Has domain(s) with predicted oxidoreductase activity, phosphopantetheine binding, transferase activity, transferring acyl groups other than amino-acyl groups, zinc ion binding activity and role in oxidation-reduction process |
| Afu3g03610 | Has domain(s) with predicted cellulase activity, hydrolase activity, hydrolyzing O-glycosyl compounds activity and role in polysaccharide catabolic process |
| Afu3g03620 | Ortholog of A. nidulans FGSC A4 : AN8444/celA, A. fumigatus Af293 : Afu8g00680, A. niger CBS 513.88 : An03g05740, A. oryzae RIB40 : AO090138000033, AO090701000186 and Aspergillus wentii : Aspwe1_0040565 |
| Afu3g03637 | rRNA |
| Afu3g03710 | Ortholog(s) have aminopeptidase activity and role in cellular protein catabolic process |
| Afu3g03720 | protein of unknown function |
| Afu3g03740 | Putative protein kinase |
| Afu3g03760 | protein of unknown function |
| Afu3g03815 | Ortholog of Neosartorya fischeri NRRL 181 : NFIA_006080, Aspergillus fumigatus A1163 : AFUB_044140 and Aspergillus terreus NIH2624 : ATET_02723 |
| Afu3g03910 | Ortholog of A. nidulans FGSC A4 : AN9192, A. niger CBS 513.88 : An13g01790, A. oryzae RIB40 : AO090038000138, Neosartorya fischeri NRRL 181 : NFIA_006190 and Aspergillus kawachii : Aspka1_0177086 |
| Afu3g03920 | Has domain(s) with predicted RNA polymerase II transcription factor activity, sequence-specific DNA binding, zinc ion binding activity, role in regulation of transcription, DNA-templated and nucleus localization |
| Afu3g03940 | Putative 2,3-diketo-5-methylthio-1-phosphopentane phosphatase |
| Afu3g03970 | Has domain(s) with predicted heme binding, oxidoreductase activity and role in oxidation-reduction process |
| Afu3g03990 | Ortholog of A. oryzae RIB40 : AO090124000057, Neosartorya fischeri NRRL 181 : NFIA_006280, Aspergillus clavatus NRRL 1 : ACLA_060770 and Aspergillus niger ATCC 1015 : 127791-mRNA |
| Afu3g04030 | Ortholog(s) have mitochondrion localization |
| Afu3g05580 | Ortholog of A. nidulans FGSC A4 : AN3445, A. niger CBS 513.88 : An11g11050, A. oryzae RIB40 : AO090020000030, Aspergillus wentii : Aspwe1_0173708 and Aspergillus sydowii : Aspsy1_0088185 |
| Afu3g05630 | Putative phospholipase D |
| Afu3g05660 | Has domain(s) with predicted role in transmembrane transport and integral component of membrane localization |
| Afu3g05700 | Has domain(s) with predicted ARF guanyl-nucleotide exchange factor activity and role in regulation of ARF protein signal transduction |
| Afu3g05790 | Ortholog of A. nidulans FGSC A4 : AN3430, A. niger CBS 513.88 : An11g10810, A. oryzae RIB40 : AO090020000047, Aspergillus wentii : Aspwe1_0029428, Aspwe1_0037899 and Aspergillus sydowii : Aspsy1_0056861 |
| Afu3g05990 | Ortholog of A. nidulans FGSC A4 : AN10339, Neosartorya fischeri NRRL 181 : NFIA_071260, Aspergillus wentii : Aspwe1_0042197 and Aspergillus clavatus NRRL 1 : ACLA_033940 |
| Afu3g06020 | Glyoxalase family protein |
| Afu3g06140 | Ortholog(s) have role in actin filament organization, activation of bipolar cell growth, cellular protein localization, cortical actin cytoskeleton organization, endocytosis and establishment of cell polarity, more |
| Afu3g06400 | Ortholog(s) have protein-lysine N-methyltransferase activity, role in peptidyl-lysine methylation and cytosol, nucleolus localization |
| Afu3g06520 | Ortholog of A. nidulans FGSC A4 : AN4768, A. niger CBS 513.88 : An11g09800, A. oryzae RIB40 : AO090020000350, Aspergillus wentii : Aspwe1_0029356 and Aspergillus sydowii : Aspsy1_0043240 |
| Afu3g06540 | Ortholog(s) have phosphoadenylyl-sulfate reductase (thioredoxin) activity |
| Afu3g06590 | Has domain(s) with predicted catalytic activity, deoxyribose-phosphate aldolase activity, lyase activity, role in deoxyribonucleotide catabolic process and cytoplasm localization |
| Afu3g06600 | Ortholog(s) have uroporphyrin-III C-methyltransferase activity and role in cellular response to drug, methionine biosynthetic process, siroheme biosynthetic process |
| Afu3g06610 | Proteasome regulatory particle subunit |
| Afu3g06660 | Putaive NIPSNAP family protein |
| Afu3g06670 | Putative pyridoxamine phosphate oxidase |
| Afu3g06840 | Putative cytosolic small ribosomal subunit S4 |
| Afu3g06860 | Ortholog of Aspergillus fumigatus A1163 : AFUB_042190 |
| Afu3g06901 | Ortholog(s) have P-P-bond-hydrolysis-driven protein transmembrane transporter activity, protein transporter activity, structural molecule activity |
| Afu3g07015 | septin |
| Afu3g07020 | Putative annexin-like protein |
| Afu3g07080 | Ortholog(s) have role in cellular calcium ion homeostasis, hyphal growth and Golgi medial cisterna, cis-Golgi network, endoplasmic reticulum, fungal-type vacuole membrane localization |
| Afu3g07270 | Ortholog of A. nidulans FGSC A4 : AN4834, A. niger CBS 513.88 : An02g13430, A. oryzae RIB40 : AO090020000197, Aspergillus wentii : Aspwe1_0052406 and Aspergillus sydowii : Aspsy1_0087772 |
| Afu3g07290 | protein of unknown function |
| Afu3g07440 | Has domain(s) with predicted hydrolase activity, zinc ion binding activity |
| Afu3g07460 | Ortholog of A. nidulans FGSC A4 : AN10921, A. niger CBS 513.88 : An01g14390, Neosartorya fischeri NRRL 181 : NFIA_069710 and Aspergillus niger ATCC 1015 : 119074-mRNA |
| Afu3g07520 | Ortholog of A. nidulans FGSC A4 : AN4852, A. fumigatus Af293 : Afu2g00430/exg9, A. niger CBS 513.88 : An02g13180, A. oryzae RIB40 : AO090020000144 and Aspergillus wentii : Aspwe1_0157603 |
| Afu3g07580 | Ortholog of Neosartorya fischeri NRRL 181 : NFIA_069600, Aspergillus wentii : Aspwe1_0176375, Aspergillus fumigatus A1163 : AFUB_041540 and Aspergillus clavatus NRRL 1 : ACLA_035370 |
| Afu3g07590 | Ortholog(s) have role in Golgi organization and Golgi apparatus localization |
| Afu3g07610 | Ortholog of A. oryzae RIB40 : AO090020000132, Neosartorya fischeri NRRL 181 : NFIA_069560 and Aspergillus fumigatus A1163 : AFUB_041500 |
| Afu3g07660 | Ortholog of A. nidulans FGSC A4 : AN11766, A. oryzae RIB40 : AO090020000405, Neosartorya fischeri NRRL 181 : NFIA_069490, Aspergillus wentii : Aspwe1_0173499 and Aspergillus clavatus NRRL 1 : ACLA_035620 |
| Afu3g07680 | Putative ran GTPase activating protein |
| Afu3g07690 | Ortholog(s) have copper chaperone activity, role in intracellular copper ion transport, mitochondrial respiratory chain complex IV assembly and cytosol, mitochondrial intermembrane space, nucleus localization |
| Afu3g07750 | Has domain(s) with predicted transferase activity, transferring acyl groups other than amino-acyl groups activity |
| Afu3g07890 | Ortholog of A. nidulans FGSC A4 : AN2953, A. niger CBS 513.88 : An02g11360, A. oryzae RIB40 : AO090005001486, Aspergillus wentii : Aspwe1_0041944 and Aspergillus sydowii : Aspsy1_0056308 |
| Afu3g08020 | Ortholog(s) have phosphatidylinositol binding activity and cytosol, mitochondrion, nucleus localization |
| Afu3g08030 | Ortholog(s) have role in regulation of DNA methylation and nucleus localization |
| Afu3g08180 | Ortholog(s) have copper uptake transmembrane transporter activity and role in cellular copper ion homeostasis, copper ion import, copper ion transmembrane transport, intracellular copper ion transport |
| Afu3g08210 | Ortholog of A. nidulans FGSC A4 : AN2937, A. niger CBS 513.88 : An02g11760, Aspergillus wentii : Aspwe1_0029179 and Aspergillus sydowii : Aspsy1_0042633 |
| Afu3g08260 | Ortholog(s) have role in mitotic sister chromatid cohesion and nuclear mitotic cohesin complex localization |
| Afu3g08265 | Ortholog of A. oryzae RIB40 : AO090005001451, Aspergillus wentii : Aspwe1_0041880, Aspergillus sydowii : Aspsy1_0042580 and Aspergillus terreus NIH2624 : ATET_01643 |
| Afu3g08270 | Pyruvate dehydrogenase complex component |
| Afu3g08430 | Ortholog(s) have mitochondrion localization |
| Afu3g08460 | 60S ribosomal protein L35Ae |
| Afu3g08470 | Glucose-6-phosphate 1-dehydrogenase |
| Afu3g08590 | Ortholog of A. nidulans FGSC A4 : AN2991, A. oryzae RIB40 : AO090005001413, Aspergillus wentii : Aspwe1_0029140, Aspergillus sydowii : Aspsy1_0042545 and Aspergillus terreus NIH2624 : ATET_01611 |
| Afu3g08660 | Putative isocitrate dehydrogenase |
| Afu3g08730 | Ortholog(s) have ubiquitin-specific protease activity and role in endocytosis, protein deubiquitination, protein targeting to vacuole involved in ubiquitin-dependent protein catabolic process via the multivesicular body sorting pathway |
| Afu3g08760 | Ortholog(s) have tRNA (guanine(9)-N(1))-methyltransferase activity, role in tRNA N1-guanine methylation and cytoplasm, nucleolus localization |
| Afu3g08810 | Ortholog(s) have cytosol localization |
| Afu3g08830 | Ortholog of A. nidulans FGSC A4 : AN3016, A. niger CBS 513.88 : An16g02060, A. oryzae RIB40 : AO090005001385, Aspergillus wentii : Aspwe1_0173356 and Aspergillus sydowii : Aspsy1_0029265 |
| Afu3g08890 | protein of unknown function |
| Afu3g08920 | Ortholog of A. nidulans FGSC A4 : AN3021, A. niger CBS 513.88 : An16g02420, A. oryzae RIB40 : AO090005001345, Aspergillus wentii : Aspwe1_0052288 and Aspergillus sydowii : Aspsy1_0147269 |
| Afu3g08960 | Has domain(s) with predicted catalytic activity |
| Afu3g08990 | Repeat-rich glycophosphatidylinositol (GPI)-anchored cell wall protein |
| Afu3g09020 | Ortholog(s) have cytoplasm, nuclear chromatin localization |
| Afu3g09090 | Ortholog(s) have mitochondrion localization |
| Afu3g09170 | Has domain(s) with predicted role in transmembrane transport and integral component of membrane localization |
| Afu3g09250 | Has domain(s) with predicted hydrolase activity, hydrolyzing O-glycosyl compounds activity and role in carbohydrate metabolic process |
| Afu3g09320 | Serine hydroxymethyltransferase |
| Afu3g09400 | Has domain(s) with predicted role in transmembrane transport and integral component of membrane localization |
| Afu3g09410 | protein of unknown function |
| Afu3g09450 | Ortholog of Aspergillus versicolor : Aspve1_0032283, Aspergillus fumigatus A1163 : AFUB_039720 and Aspergillus sydowii : Aspsy1_0187904 |
| Afu3g09630 | Ortholog(s) have asparagine-tRNA ligase activity, role in mitochondrial asparaginyl-tRNA aminoacylation and mitochondrion localization |
| Afu3g09710 | Ortholog(s) have Golgi apparatus, cell division site, cell tip localization |
| Afu3g09720 | Ortholog(s) have mRNA cleavage and polyadenylation specificity factor complex localization |
| Afu3g09780 | Ortholog(s) have role in cellular copper ion homeostasis, mitochondrial respiratory chain complex IV assembly and mitochondrial intermembrane space, nucleus localization |
| Afu3g09800 | Ortholog(s) have cytosol, nucleus localization |
| Afu3g09850 | Ortholog(s) have ATP binding, ATPase activity, Y-form DNA binding, double-strand/single-strand DNA junction binding, four-way junction DNA binding and guanine/thymine mispair binding, more |
| Afu3g09930 | Ortholog of A. nidulans FGSC A4 : AN4995, A. niger CBS 513.88 : An16g03610, A. oryzae RIB40 : AO090003000511, Aspergillus glaucus : Aspgl1_0123810 and Neosartorya fischeri NRRL 181 : NFIA_067370 |
| Afu3g10085 | Ortholog of Aspergillus fumigatus A1163 : AFUB_039090 |
| Afu3g10190 | Ortholog(s) have peroxisome matrix targeting signal-1 binding, peroxisome matrix targeting signal-2 binding, protein binding, bridging activity |
| Afu3g10230 | Ortholog(s) have endoplasmic reticulum localization |
| Afu3g10320 | Has domain(s) with predicted zinc ion binding activity |
| Afu3g10400 | Dolichol-phosphate mannosyltransferase with a predicted role in N- and O-linked protein glycosylation |
| Afu3g10410 | Ortholog of A. nidulans FGSC A4 : AN4946, A. niger CBS 513.88 : An16g04340, A. oryzae RIB40 : AO090003000590, Aspergillus wentii : Aspwe1_0169033 and Aspergillus sydowii : Aspsy1_0087645 |
| Afu3g10520 | Ortholog(s) have nucleus localization |
| Afu3g10670 | Ortholog of A. nidulans FGSC A4 : AN4922, A. niger CBS 513.88 : An02g06330, A. oryzae RIB40 : AO090003000612, Aspergillus wentii : Aspwe1_0360359 and Aspergillus sydowii : Aspsy1_0147179 |
| Afu3g10700 | Ortholog(s) have mRNA binding, structural molecule activity and role in Arp2/3 complex-mediated actin nucleation, actin cortical patch assembly, cellular response to drug, mitochondrion inheritance |
| Afu3g10710 | Protein of unknown function identified by mass spectrometry |
| Afu3g10720 | Ortholog(s) have microtubule motor activity, role in actin cytoskeleton organization, conidium formation, hyphal growth, microtubule-based movement, nuclear migration and dynactin complex localization |
| Afu3g10780 | Ortholog of A. nidulans FGSC A4 : AN4911, A. niger CBS 513.88 : An02g06450, Neosartorya fischeri NRRL 181 : NFIA_066470, Aspergillus wentii : Aspwe1_0362213 and Aspergillus versicolor : Aspve1_0127307 |
| Afu3g10810 | Ortholog of A. nidulans FGSC A4 : AN4907, A. niger CBS 513.88 : An03g02970, Aspergillus wentii : Aspwe1_0362245, Aspergillus sydowii : Aspsy1_0043016 and Aspergillus terreus NIH2624 : ATET_04610 |
| Afu3g10890 | Ortholog(s) have ATP-dependent RNA helicase activity |
| Afu3g10900 | Ortholog of Aspergillus fumigatus A1163 : AFUB_038230 |
| Afu3g10910 | Putative glutaminase |
| Afu3g10930 | Ortholog(s) have double-stranded DNA binding, sequence-specific DNA binding activity |
| Afu3g11160 | Ortholog(s) have cell division site, cytosol, nucleus localization |
| Afu3g11240 | protein of unknown function |
| Afu3g11290 | Ortholog of A. nidulans FGSC A4 : AN4870, A. niger CBS 513.88 : An02g07030, Aspergillus wentii : Aspwe1_0169120, Aspergillus sydowii : Aspsy1_0087725 and Aspergillus terreus NIH2624 : ATET_04661 |
| Afu3g11320 | Ortholog(s) have gamma-tubulin binding activity and role in cytoplasmic microtubule organization, establishment or maintenance of cell polarity regulating cell shape, mitotic spindle assembly |
| Afu3g11400 | Aspartic acid endopeptidase |
| Afu3g11410 | Ortholog(s) have CTD phosphatase activity and role in dephosphorylation of RNA polymerase II C-terminal domain, negative regulation of G0 to G1 transition, regulation of mitotic cytokinesis |
| Afu3g11480 | Putative enoyl-CoA hydratase |
| Afu3g11530 | Ortholog(s) have mitochondrion localization |
| Afu3g11540 | Ortholog of A. nidulans FGSC A4 : AN2893/fhdA, A. niger CBS 513.88 : An14g03210, A. oryzae RIB40 : AO090003000711, Neosartorya fischeri NRRL 181 : NFIA_065770 and Aspergillus clavatus NRRL 1 : ACLA_039420 |
| Afu3g11590 | Predicted autophagy protein with a predicted role in trafficking of autophagosomes and cytoplasm to vacuole |
| Afu3g11630 | Ortholog(s) have role in regulation of telomere maintenance via telomerase and nucleus localization |
| Afu3g11730 | Ortholog(s) have mitochondrion localization |
| Afu3g11740 | Ortholog(s) have role in anisotropic cell growth and cytoplasm, nucleus localization |
| Afu3g11750 | Ortholog(s) have oxysterol binding, phosphatidic acid binding, phosphatidylinositol-4,5-bisphosphate binding, phosphatidylinositol-4-phosphate binding, sterol transporter activity |
| Afu3g11770 | Ortholog of A. nidulans FGSC A4 : AN5876, AN2876, A. fumigatus Af293 : Afu2g11370, Aspergillus wentii : Aspwe1_0653753 and Aspergillus sydowii : Aspsy1_0146596, Aspsy1_1176317 |
| Afu3g11790 | Has domain(s) with predicted substrate-specific transmembrane transporter activity, transmembrane transporter activity, role in transmembrane transport and integral component of membrane, membrane localization |
| Afu3g11810 | Ortholog of A. nidulans FGSC A4 : AN10373, A. niger CBS 513.88 : An02g07640, A. oryzae RIB40 : AO090003000743, Aspergillus wentii : Aspwe1_0104946 and Aspergillus sydowii : Aspsy1_0778921 |
| Afu3g11820 | Ortholog(s) have 3'-5'-exoribonuclease activity |
| Afu3g11860 | Putative microtubule associated protein |
| Afu3g11920 | Has domain(s) with predicted catalytic activity, lyase activity and role in metabolic process |
| Afu3g11930 | Has domain(s) with predicted oxidoreductase activity |
| Afu3g11950 | Ortholog(s) have 3'-5' exonuclease activity, role in ascospore formation, conversion of ds siRNA to ss siRNA involved in RNA interference, fruiting body development, pre-miRNA processing and perinuclear region of cytoplasm localization |
| Afu3g11990 | Ortholog(s) have sequence-specific DNA binding activity |
| Afu3g12010 | Has domain(s) with predicted substrate-specific transmembrane transporter activity, transmembrane transporter activity, role in transmembrane transport and integral component of membrane, membrane localization |
| Afu3g12080 | Ortholog(s) have enzyme regulator activity, mRNA binding activity |
| Afu3g12100 | Putative trehalase phosphorylase with a predicted role in the glucose-1-phosphate pathway |
| Afu3g12130 | Ortholog of S. cerevisiae : YEL023C, A. nidulans FGSC A4 : AN1725, AN5027, A. fumigatus Af293 : Afu2g14400 and A. niger CBS 513.88 : An11g08400, An02g07940, An12g01310, An02g00780 |
| Afu3g12140 | Ortholog of A. nidulans FGSC A4 : AN5026, A. niger CBS 513.88 : An02g08280, A. oryzae RIB40 : AO090003000774, Aspergillus wentii : Aspwe1_0169199 and Aspergillus sydowii : Aspsy1_0087531 |
| Afu3g12150 | Has domain(s) with predicted oxidoreductase activity and role in oxidation-reduction process |
| Afu3g12350 | Ortholog(s) have role in cation transport, cellular response to drug, regulation of membrane potential and cytoplasm, plasma membrane localization |
| Afu3g12360 | Ortholog of A. nidulans FGSC A4 : AN3087, A. niger CBS 513.88 : An03g02910, A. oryzae RIB40 : AO090005000731, Aspergillus wentii : Aspwe1_0049379 and Aspergillus sydowii : Aspsy1_0056184 |
| Afu3g12600 | Has domain(s) with predicted catalytic activity, hydrolase activity, hydrolyzing O-glycosyl compounds activity and role in carbohydrate metabolic process |
| Afu3g12790 | Transcript up-regulated in conidia exposed to neutrophils |
| Afu3g12960 | Putative cytochrome P450 |
| Afu3g12970 | Ortholog(s) have plasma membrane localization |
| Afu3g13180 | Ortholog of A. niger CBS 513.88 : An02g09040, A. oryzae RIB40 : AO090012000814, Neosartorya fischeri NRRL 181 : NFIA_064160 and Aspergillus wentii : Aspwe1_0170078 |
| Afu3g13230 | Has domain(s) with predicted DNA binding activity |
| Afu3g13240 | Aldose 1-epimerase |
| Afu3g13340 | Has domain(s) with predicted GTP binding, GTPase activity, role in protein transport, signal transduction, small GTPase mediated signal transduction and intracellular, membrane localization |
| Afu3g13350 | Ortholog(s) have cytosol, nucleus localization |
| Afu3g13380 | Putative ribose-phosphate pyrophosphokinase |
| Afu3g13440 | Stomatin family protein |
| Afu3g13610 | Ortholog(s) have intracellular localization |
| Afu3g13700 | Has domain(s) with predicted catalytic activity, phosphopantetheine binding, transferase activity, transferring acyl groups other than amino-acyl groups activity and role in metabolic process |
| Afu3g13720 | Has domain(s) with predicted oxidoreductase activity, acting on paired donors, with incorporation or reduction of molecular oxygen, 2-oxoglutarate as one donor, and incorporation of one atom each of oxygen into both donors activity |
| Afu3g13750 | Ortholog of A. oryzae RIB40 : AO090010000138, Neosartorya fischeri NRRL 181 : NFIA_063520, Aspergillus wentii : Aspwe1_0044901 and Aspergillus fumigatus A1163 : AFUB_035440 |
| Afu3g13755 | protein of unknown function |
| Afu3g13950 | Has domain(s) with predicted role in cell division, chromosome segregation and MIS12/MIND type complex localization |
| Afu3g14010 | Ortholog(s) have DNA replication origin binding, chromatin binding, single-stranded DNA binding, single-stranded DNA-dependent ATP-dependent DNA helicase activity |
| Afu3g14080 | unspecified product |
| Afu3g14100 | Ortholog(s) have cytosol, nucleus localization |
| Afu3g14110 | Ortholog(s) have S-adenosylmethionine-dependent methyltransferase activity, role in chromatin silencing at rDNA, nicotinamide metabolic process and cytosol localization |
| Afu3g14180 | Ortholog of A. nidulans FGSC A4 : AN3898, A. niger CBS 513.88 : An09g04380, A. oryzae RIB40 : AO090012000661, Aspergillus wentii : Aspwe1_0038053 and Aspergillus sydowii : Aspsy1_0034796, Aspsy1_0915433 |
| Afu3g14250 | Ortholog(s) have indoleamine 2,3-dioxygenase activity and role in filamentous growth of a population of unicellular organisms in response to chemical stimulus, tryptophan catabolic process to kynurenine |
| Afu3g14260 | Ortholog of A. niger CBS 513.88 : An09g04240, A. oryzae RIB40 : AO090012000670, Neosartorya fischeri NRRL 181 : NFIA_063040 and Aspergillus wentii : Aspwe1_0038047 |
| Afu3g14280 | Ortholog(s) have cytosol localization |
| Afu3g14360 | Ortholog of Aspergillus fumigatus A1163 : AFUB_034870 |
| Afu3g14410 | Ortholog of A. nidulans FGSC A4 : AN2522, A. niger CBS 513.88 : An09g04050, A. oryzae RIB40 : AO090701000592, Aspergillus wentii : Aspwe1_0073746 and Aspergillus sydowii : Aspsy1_0146188 |
| Afu3g14430 | Ortholog(s) have role in regulation of mitotic cell cycle and cytosol, nucleus localization |
| Afu3g14445 | Ortholog of A. nidulans FGSC A4 : AN10317, A. niger CBS 513.88 : An09g03980, A. oryzae RIB40 : AO090701000586, Neosartorya fischeri NRRL 181 : NFIA_062810 and Aspergillus wentii : Aspwe1_0107543 |
| Afu3g14490 | Putative ketol acid reductoisomerase |
| Afu3g14680 | Putative secreted lysophospholipase B |
| Afu3g14740 | Ortholog of Aspergillus fumigatus A1163 : AFUB_034480 |
| Afu3g14750 | Has domain(s) with predicted DNA binding, zinc ion binding activity, role in transcription, DNA-templated and nucleus localization |
| Afu3g14760 | Ortholog(s) have role in steroid metabolic process |
| Afu3g14790 | Has domain(s) with predicted shikimate 3-dehydrogenase (NADP+) activity, role in oxidation-reduction process and cytoplasm localization |
| Afu3g14800 | Has domain(s) with predicted shikimate 3-dehydrogenase (NADP+) activity and role in oxidation-reduction process |
| Afu3g14810 | Ortholog of A. nidulans FGSC A4 : AN9274, A. niger CBS 513.88 : An02g03200, An04g09720, An04g08110, A. oryzae RIB40 : AO090103000427 and Aspergillus wentii : Aspwe1_0110739 |
| Afu3g14850 | 3-dehydroquinate dehydratase |
| Afu3g15040 | Has domain(s) with predicted monooxygenase activity, oxidoreductase activity, acting on paired donors, with incorporation or reduction of molecular oxygen activity and role in oxidation-reduction process |
| Afu3g15110 | Ortholog of A. nidulans FGSC A4 : AN2942, AN10320, AN7130 and A. fumigatus Af293 : Afu4g08715 |
| Afu3g15150 | Ortholog of A. nidulans FGSC A4 : AN3975, AN9193/llmJ, AN2891 and A. fumigatus Af293 : Afu1g10150, Afu2g04380, Afu3g15280, Afu6g03300 |
| Afu3g15170 | Has domain(s) with predicted role in Mo-molybdopterin cofactor biosynthetic process, molybdopterin cofactor biosynthetic process |
| Afu3g15190 | Putative nitrate reductase |
| Afu3g15260 | Ortholog of Aspergillus fumigatus A1163 : AFUB_033960 |
| Afu3g15270 | Putative nonribosomal peptide synthase (NRPS), encoded in a predicted secondary metabolite gene cluster |
| Afu4g00110 | TfdA family taurine dioxygenase |
| Afu4g00150 | Putative MFS maltose transporter |
| Afu4g00180 | Fatty acid 8,11-diol synthase |
| Afu4g00190 | protein of unknown function |
| Afu4g00210 | Polyketide synthase involved in endocrocin biosynthesis |
| Afu4g00225 | Anthrone oxidase involved in endocrocin biosynthesis |
| Afu4g00230 | 2-oxoglutarate-Fe(II) type oxidoreductase involved in endocrocin biosynthesis |
| Afu4g00480 | Ortholog of Aspergillus fumigatus A1163 : AFUB_101020 |
| Afu4g00550 | Has domain(s) with predicted role in transmembrane transport and integral component of membrane localization |
| Afu4g00570 | Has domain(s) with predicted role in transmembrane transport and integral component of membrane localization |
| Afu4g00630 | Has domain(s) with predicted catalytic activity, pyridoxal phosphate binding activity and role in biosynthetic process |
| Afu4g00640 | Has domain(s) with predicted carbon-carbon lyase activity, catalytic activity and role in cellular aromatic compound metabolic process |
| Afu4g00870 | Ortholog(s) have extracellular region localization |
| Afu4g01000 | Has domain(s) with predicted role in transmembrane transport and integral component of membrane localization |
| Afu4g01020 | Ortholog(s) have protein histidine kinase activity and role in peptidyl-histidine autophosphorylation |
| Afu4g01040 | Has domain(s) with predicted oxidoreductase activity and role in metabolic process |
| Afu4g01050 | Has domain(s) with predicted ATP binding, ATPase activity, ATPase activity, coupled to transmembrane movement of substances, nucleoside-triphosphatase activity, nucleotide binding activity and role in transport |
| Afu4g01090 | Has domain(s) with predicted hydrolase activity |
| Afu4g01110 | Putative 1,3-beta glucanase |
| Afu4g01115 | protein of unknown function |
| Afu4g01120 | Ortholog of A. nidulans FGSC A4 : AN11048, AN5410, A. fumigatus Af293 : Afu3g01150, A. niger CBS 513.88 : An12g10330, An11g03520, An02g14500 and A. oryzae RIB40 : AO090005000691 |
| Afu4g01290 | Glycosyl hydrolase family 75 chitosanase |
| Afu4g01340 | Has domain(s) with predicted substrate-specific transmembrane transporter activity, transmembrane transporter activity, role in transmembrane transport and integral component of membrane, membrane localization |
| Afu4g01420 | Ortholog of Neosartorya fischeri NRRL 181 : NFIA_043720, Aspergillus fumigatus A1163 : AFUB_101900 and Aspergillus clavatus NRRL 1 : ACLA_061750 |
| Afu4g01510 | Ortholog(s) have role in sporocarp development involved in sexual reproduction |
| Afu4g01530 | Has domain(s) with predicted FAD binding, oxidoreductase activity and role in metabolic process |
| Afu4g01560 | Putative major facilitator superfamily (MFS) myo-inositol transporter |
| Afu4g01590 | Ortholog of Aspergillus fumigatus A1163 : AFUB_102090 and Aspergillus niger ATCC 1015 : 126161-mRNA |
| Afu4g01600 | Ortholog of Aspergillus fumigatus A1163 : AFUB_102100 |
| Afu4g02100 | rRNA |
| Afu4g02730 | Has domain(s) with predicted electron carrier activity, heme binding, iron ion binding, oxidoreductase activity, acting on paired donors, with incorporation or reduction of molecular oxygen activity and role in oxidation-reduction process |
| Afu4g02740 | Ortholog of Aspergillus fumigatus A1163 : AFUB_100410 |
| Afu4g02760 | Ortholog of A. nidulans FGSC A4 : AN2370, AN10160, AN0323, A. fumigatus Af293 : Afu1g02440, A. niger CBS 513.88 : An01g05750, An12g09350, An13g03290 and A. oryzae RIB40 : AO090020000719, AO090005000852 |
| Afu4g02860 | Has domain(s) with predicted catalytic activity |
| Afu4g02900 | Putative sensor histidine kinase/response regulator |
| Afu4g02940 | Has domain(s) with predicted sequence-specific DNA binding, transcription factor activity, sequence-specific DNA binding activity and role in regulation of transcription, DNA-templated |
| Afu4g02960 | Has domain(s) with predicted hydrolase activity and role in nucleotide catabolic process |
| Afu4g03110 | putative monosaccharide transporter |
| Afu4g03130 | Has domain(s) with predicted hydrolase activity |
| Afu4g03140 | Ortholog(s) have RNA uridylyltransferase activity, protein serine/threonine kinase activity |
| Afu4g03150 | Ortholog(s) have O6-alkylguanine-DNA binding activity, role in DNA dealkylation involved in DNA repair and cytosol, nucleus localization |
| Afu4g03220 | Ortholog(s) have role in leucine import and Golgi apparatus, endoplasmic reticulum localization |
| Afu4g03510 | Ortholog of A. nidulans FGSC A4 : AN7150, A. niger CBS 513.88 : An14g02590, A. oryzae RIB40 : AO090011000238, Aspergillus wentii : Aspwe1_0115640 and Aspergillus sydowii : Aspsy1_0034579 |
| Afu4g03550 | Ortholog of A. fumigatus Af293 : Afu6g04370, A. niger CBS 513.88 : An01g09070, An07g06910, An12g07420, An01g10470, An09g04030 and A. oryzae RIB40 : AO090010000022, AO090005000964 |
| Afu4g03560 | Has domain(s) with predicted cellulose binding, hydrolase activity, hydrolyzing O-glycosyl compounds, serine-type peptidase activity, role in carbohydrate metabolic process, proteolysis and extracellular region localization |
| Afu4g03580 | Ortholog(s) have phosphatase activity, role in dephosphorylation and cytoplasm, nucleus localization |
| Afu4g03600 | GPI acetyltransferase |
| Afu4g03620 | Ortholog(s) have role in nucleobase-containing compound transport and plasma membrane localization |
| Afu4g03680 | Putative oxidoreductase, short-chain dehydrogenase/reductase family |
| Afu4g03790 | Putative serine peptidase, family S28 |
| Afu4g03820 | Ortholog of A. nidulans FGSC A4 : AN7101, A. niger CBS 513.88 : An14g01070, A. oryzae RIB40 : AO090011000344, Aspergillus wentii : Aspwe1_0115647 and Aspergillus sydowii : Aspsy1_0048914 |
| Afu4g03840 | Ortholog(s) have DNA-dependent ATPase activity, role in histone H2A-H2B dimer displacement, transcription-coupled nucleotide-excision repair and cytosol, nucleus localization |
| Afu4g03850 | Ortholog(s) have protein serine/threonine kinase activity, protein tyrosine kinase activity |
| Afu4g03880 | 60S ribosomal protein L7 |
| Afu4g03910 | Has domain(s) with predicted zinc ion binding activity |
| Afu4g03960 | Putative C6 transcription factor with a role in beta oxidation of long-chain fatty acids |
| Afu4g04110 | Ortholog(s) have cytosol, nucleus localization |
| Afu4g04190 | Ortholog of A. nidulans AN7033 |
| Afu4g04210 | Putative secreted aminopeptidase |
| Afu4g04220 | Ortholog of A. nidulans FGSC A4 : AN7025, A. oryzae RIB40 : AO090206000072, Aspergillus wentii : Aspwe1_0114911, Aspergillus sydowii : Aspsy1_0060635 and Aspergillus terreus NIH2624 : ATET_10114 |
| Afu4g04250 | Has domain(s) with predicted role in transmembrane transport and integral component of membrane localization |
| Afu4g04280 | Ortholog(s) have proteasome binding activity, role in cellular response to arsenic-containing substance, proteasome-mediated ubiquitin-dependent protein catabolic process and cytoplasm, nucleus localization |
| Afu4g04300 | Ortholog(s) have pyridoxal phosphate binding activity and cytosol, nucleus localization |
| Afu4g04350 | Ortholog(s) have U2-type spliceosomal complex localization |
| Afu4g04355 | Ortholog(s) have cytosol, nucleus localization |
| Afu4g04360 | Ortholog(s) have double-stranded DNA binding activity and role in nucleotide-excision repair, phosphorylation of RNA polymerase II C-terminal domain, transcription from RNA polymerase II promoter |
| Afu4g04415 | Has domain(s) with predicted zinc ion binding activity |
| Afu4g04430 | Ortholog of A. nidulans FGSC A4 : AN7006, A. niger CBS 513.88 : An14g00400, A. oryzae RIB40 : AO090206000050, Aspergillus wentii : Aspwe1_0115358 and Aspergillus sydowii : Aspsy1_0092883 |
| Afu4g04440 | Ortholog of A. nidulans FGSC A4 : AN7005, A. niger CBS 513.88 : An14g00390, A. oryzae RIB40 : AO090206000049, Aspergillus wentii : Aspwe1_0116246 and Aspergillus sydowii : Aspsy1_0158059 |
| Afu4g04450 | Ortholog of A. nidulans FGSC A4 : AN7004, A. niger CBS 513.88 : An14g00380, A. oryzae RIB40 : AO090206000048, Aspergillus wentii : Aspwe1_0043225 and Aspergillus sydowii : Aspsy1_0034669 |
| Afu4g04465 | Ortholog of A. nidulans FGSC A4 : AN7002, A. niger CBS 513.88 : An14g00360, Aspergillus wentii : Aspwe1_0053189 and Aspergillus sydowii : Aspsy1_0034671 |
| Afu4g04470 | Ortholog of A. nidulans FGSC A4 : AN7001, A. niger CBS 513.88 : An14g00350, A. oryzae RIB40 : AO090206000046, Aspergillus wentii : Aspwe1_0043221 and Aspergillus sydowii : Aspsy1_0048992 |
| Afu4g04570 | Ortholog of A. oryzae RIB40 : AO090206000032, Aspergillus flavus NRRL 3357 : AFL2T_12442, Neosartorya fischeri NRRL 181 : NFIA_028780 and Aspergillus wentii : Aspwe1_0174563 |
| Afu4g04580 | Ortholog of Aspergillus fumigatus A1163 : AFUB_098430 |
| Afu4g04590 | Ortholog(s) have mitochondrion localization |
| Afu4g04650 | Ortholog of A. nidulans FGSC A4 : AN10872, A. niger CBS 513.88 : An14g00190, A. oryzae RIB40 : AO090206000024, Aspergillus wentii : Aspwe1_0114521 and Aspergillus sydowii : Aspsy1_0158439 |
| Afu4g04680 | FGGY-family carbohydrate kinase |
| Afu4g04690 | Ortholog of A. nidulans FGSC A4 : AN6984, A. niger CBS 513.88 : An14g00150, A. oryzae RIB40 : AO090206000018, Aspergillus wentii : Aspwe1_0043196 and Aspergillus sydowii : Aspsy1_0049019 |
| Afu4g04730 | Has domain(s) with predicted 5-amino-6-(5-phosphoribosylamino)uracil reductase activity and role in oxidation-reduction process, riboflavin biosynthetic process |
| Afu4g04820 | Putative c-4 methyl sterol oxidase with a predicted role in ergosterol biosynthesis |
| Afu4g06110 | Ortholog of A. nidulans FGSC A4 : AN4319, A. niger CBS 513.88 : An04g00380, A. oryzae RIB40 : AO090023000984, Neosartorya fischeri NRRL 181 : NFIA_110030 and Aspergillus versicolor : Aspve1_0085237 |
| Afu4g06340 | Ortholog(s) have endoplasmic reticulum localization |
| Afu4g06390 | Ortholog(s) have mitochondrion localization |
| Afu4g06400 | Ortholog(s) have role in cellular response to lithium ion, cellular response to neutral pH, entry into host and filamentous growth of a population of unicellular organisms in response to pH, more |
| Afu4g06550 | Has domain(s) with predicted zinc ion binding activity |
| Afu4g06620 | Glutamate/Leucine/Phenylalanine/Valine dehydrogenase |
| Afu4g06750 | Ortholog(s) have role in actomyosin contractile ring assembly, ascospore formation, sporocarp development involved in sexual reproduction and cytosol localization |
| Afu4g06770 | Ortholog(s) have 2 iron, 2 sulfur cluster binding, ferrous iron binding, iron-sulfur transferase activity |
| Afu4g06780 | Ortholog(s) have NAD transporter activity, pyruvate secondary active transmembrane transporter activity, role in NAD transmembrane transport, mitochondrial pyruvate transport and endoplasmic reticulum, mitochondrion localization |
| Afu4g06790 | Ubiquinol-cytochrome c reductase complex 14 kDa protein with a predicted role in oxidative phosphorylation |
| Afu4g06860 | Ortholog of Aspergillus fumigatus A1163 : AFUB_063930 |
| Afu4g07100 | Ortholog(s) have protein anchor activity and role in establishment of spindle pole body localization to nuclear envelope, mitotic spindle assembly |
| Afu4g07110 | Ortholog(s) have SNAP receptor activity, role in Golgi to vacuole transport, vacuole inheritance and Golgi apparatus, endoplasmic reticulum, fungal-type vacuole membrane, late endosome localization |
| Afu4g07140 | Ortholog(s) have tRNA binding activity, role in regulation of transcription from RNA polymerase II promoter, tRNA wobble uridine modification and Elongator holoenzyme complex, cytosol, nucleus localization |
| Afu4g07160 | Ortholog(s) have role in regulation of circadian rhythm and TRAMP complex localization |
| Afu4g07300 | Predicted adhesin-like protein |
| Afu4g07330 | Ortholog(s) have endoplasmic reticulum localization |
| Afu4g07380 | Ortholog(s) have role in histone exchange and NuA4 histone acetyltransferase complex, Swr1 complex localization |
| Afu4g07390 | Ortholog(s) have Golgi apparatus, endoplasmic reticulum localization |
| Afu4g07490 | Has domain(s) with predicted ubiquitin-protein transferase activity, zinc ion binding activity, role in protein ubiquitination and ubiquitin ligase complex localization |
| Afu4g07570 | Ortholog(s) have role in maturation of 5.8S rRNA from tricistronic rRNA transcript (SSU-rRNA, 5.8S rRNA, LSU-rRNA), maturation of LSU-rRNA from tricistronic rRNA transcript (SSU-rRNA, 5.8S rRNA, LSU-rRNA), ribosomal large subunit assembly |
| Afu4g07685 | unspecified product |
| Afu4g07690 | Putative phosphoribosylaminoimidazole-carboxamide formyltransferase/IMP cyclohydrolase |
| Afu4g07710 | Putative pyruvate carboxylase |
| Afu4g07800 | Ortholog(s) have CAAX-protein geranylgeranyltransferase activity, protein farnesyltransferase activity |
| Afu4g07960 | Ortholog of Aspergillus brasiliensis : Aspbr1_0607466, Aspergillus glaucus : Aspgl1_0047581, Aspergillus kawachii : Aspka1_0172929 and Aspergillus acidus : Aspfo1_0044892 |
| Afu4g07970 | Ortholog(s) have DNA-directed DNA polymerase activity and role in DNA replication, removal of RNA primer, DNA synthesis involved in UV-damage excision repair, RNA-dependent DNA replication, premeiotic DNA replication |
| Afu4g08180 | Ortholog of A. nidulans FGSC A4 : AN3828, A. niger CBS 513.88 : An04g02620, A. oryzae RIB40 : AO090023000753, Aspergillus wentii : Aspwe1_0034490 and Aspergillus sydowii : Aspsy1_0057397 |
| Afu4g08360 | Ortholog(s) have role in cellular protein localization and cytosol, mediator complex localization |
| Afu4g08460 | Ortholog of A. nidulans FGSC A4 : AN1712, A. oryzae RIB40 : AO090023000716, Neosartorya fischeri NRRL 181 : NFIA_107710, Aspergillus wentii : Aspwe1_0102930 and Aspergillus versicolor : Aspve1_0079819 |
| Afu4g08510 | Putative RNA triphosphatase involved in mRNA 5' cap formation |
| Afu4g08520 | Has domain(s) with predicted transferase activity, transferring acyl groups other than amino-acyl groups activity |
| Afu4g08720 | Putative secreted phospholipase B |
| Afu4g08900 | Ortholog(s) have role in nucleotide-excision repair, phosphorylation of RNA polymerase II C-terminal domain, regulation of cyclin-dependent protein serine/threonine kinase activity, transcription from RNA polymerase II promoter |
| Afu4g08930 | Putative nucleolar GTPase |
| Afu4g09030 | Putative aminopeptidase |
| Afu4g09050 | Putative serine/threonine-protein kinase |
| Afu4g09160 | Ortholog of Aspergillus fumigatus A1163 : AFUB_066260 |
| Afu4g09190 | Putative S-adenosyl-methionine-sterol-C methyltransferase |
| Afu4g09230 | Ortholog of A. nidulans FGSC A4 : AN1610, A. oryzae RIB40 : AO090023000613, Neosartorya fischeri NRRL 181 : NFIA_106900, Aspergillus wentii : Aspwe1_0166893 and Aspergillus versicolor : Aspve1_0440039 |
| Afu4g09260 | Ortholog of Aspergillus fumigatus A1163 : AFUB_066390 |
| Afu4g09330 | Ortholog of A. oryzae RIB40 : AO090023000600, AO090138000106, Neosartorya fischeri NRRL 181 : NFIA_106680, Aspergillus versicolor : Aspve1_0079668 and Aspergillus clavatus NRRL 1 : ACLA_048690 |
| Afu4g09360 | ATP synthase proteolipid P2 with a predicted role in oxidative phosphorylation |
| Afu4g09420 | Putative carbonic anhydrase |
| Afu4g09440 | Putative P-type ATPase sodium transporter |
| Afu4g09450 | Ortholog of Neosartorya fischeri NRRL 181 : NFIA_106570 and Aspergillus fumigatus A1163 : AFUB_066570 |
| Afu4g09460 | Ortholog of Neosartorya fischeri NRRL 181 : NFIA_106560, Aspergillus versicolor : Aspve1_0060598, Aspergillus fumigatus A1163 : AFUB_066580 and Aspergillus sydowii : Aspsy1_0052051, Aspsy1_0148309 |
| Afu4g09840 | Has domain(s) with predicted catalytic activity, kynureninase activity, pyridoxal phosphate binding activity, role in NAD biosynthetic process, metabolic process, tryptophan catabolic process and cytoplasm localization |
| Afu4g09860 | Has domain(s) with predicted DNA binding, zinc ion binding activity, role in transcription, DNA-templated and nucleus localization |
| Afu4g09950 | Ortholog(s) have endoplasmic reticulum localization |
| Afu4g10010 | Ortholog(s) have heat shock protein binding activity, role in protein folding and cytosol, nucleus localization |
| Afu4g10020 | Ortholog(s) have role in fungal-type cell wall organization and Golgi apparatus, cytoplasmic vesicle, endosome localization |
| Afu4g10100 | Ortholog(s) have cytosol localization |
| Afu4g10130 | Ortholog(s) have alpha-amylase activity and role in carbohydrate catabolic process |
| Afu4g10190 | putative oxidoreductase |
| Afu4g10200 | putative transcription factor |
| Afu4g10330 | Ortholog(s) have protein farnesyltransferase activity, role in protein farnesylation and cytosol, nucleus, protein farnesyltransferase complex localization |
| Afu4g10360 | Ortholog(s) have nuclear localization sequence binding, ubiquitin binding activity, role in response to ethanol and cytosol localization |
| Afu4g10520 | Ortholog(s) have electron carrier activity, iron ion binding, zinc ion binding activity, role in peptidyl-diphthamide biosynthetic process from peptidyl-histidine, tRNA wobble uridine modification and cytosol, nucleus localization |
| Afu4g10700 | Ortholog of S. pombe dsc2 |
| Afu4g10750 | Ortholog(s) have role in N-glycan processing and Golgi apparatus, endoplasmic reticulum localization |
| Afu4g10800 | 40S ribosomal protein S6 |
| Afu4g10810 | Has domain(s) with predicted hydrolase activity |
| Afu4g10820 | Ortholog of A. nidulans FGSC A4 : AN1962, A. niger CBS 513.88 : An04g05820, Aspergillus wentii : Aspwe1_0145558, Aspergillus sydowii : Aspsy1_0142548 and Aspergillus terreus NIH2624 : ATET_03987 |
| Afu4g10910 | Has domain(s) with predicted transferase activity, transferring acyl groups other than amino-acyl groups, zinc ion binding activity, role in regulation of transcription, DNA-templated and nucleus localization |
| Afu4g10970 | Has domain(s) with predicted Rho GTPase binding, actin binding activity and role in actin cytoskeleton organization |
| Afu4g10990 | Ortholog(s) have inorganic cation transmembrane transporter activity, solute:proton symporter activity |
| Afu4g11060 | Has domain(s) with predicted role in transmembrane transport and integral component of membrane localization |
| Afu4g11160 | Ortholog(s) have role in cellular protein localization, mitotic spindle pole body duplication, regulation of exit from mitosis, regulation of mitotic sister chromatid segregation |
| Afu4g11180 | Has domain(s) with predicted ATP binding, DNA binding, nucleoside-triphosphatase activity, nucleotide binding activity and role in DNA repair |
| Afu4g11305 | unspecified product |
| Afu4g11320 | Ortholog of A. nidulans FGSC A4 : AN3743, AN5603, A. fumigatus Af293 : Afu7g04400, A. niger CBS 513.88 : An13g00980, An04g05280 and A. oryzae RIB40 : AO090005000127 |
| Afu4g11325 | unspecified product |
| Afu4g11330 | Ortholog(s) have ATPase activator activity, chaperone binding activity, role in cellular response to heat, positive regulation of ATPase activity, protein folding and cytosol localization |
| Afu4g11440 | Ortholog(s) have role in cellular response to light stimulus, protein ubiquitination, regulation of circadian rhythm and SCF ubiquitin ligase complex, cytosol localization |
| Afu4g11450 | Ortholog(s) have Rho guanyl-nucleotide exchange factor activity |
| Afu4g11460 | Putative aminotransferase |
| Afu4g11620 | Ortholog(s) have role in GINS complex assembly, double-strand break repair via break-induced replication, mitotic DNA replication initiation, mitotic DNA replication preinitiation complex assembly |
| Afu4g11630 | Ortholog of A. nidulans FGSC A4 : AN5573, A. niger CBS 513.88 : An04g04730, A. oryzae RIB40 : AO090003001056, Aspergillus wentii : Aspwe1_0166629 and Aspergillus sydowii : Aspsy1_0088453 |
| Afu4g11640 | Ortholog of A. nidulans FGSC A4 : AN5572, A. niger CBS 513.88 : An04g04740, Aspergillus wentii : Aspwe1_0047362, Aspergillus sydowii : Aspsy1_0057118 and Aspergillus terreus NIH2624 : ATET_03912 |
| Afu4g11650 | Putative alpha-ketoglutarate dehydrogenase complex subunit |
| Afu4g11660 | Ortholog(s) have methylated histone binding activity |
| Afu4g11680 | Ortholog of S. pombe dsc2 |
| Afu4g11700 | Ortholog of A. nidulans FGSC A4 : AN5568, A. niger CBS 513.88 : An04g04540, A. oryzae RIB40 : AO090003001049, Aspergillus wentii : Aspwe1_0033982 and Aspergillus sydowii : Aspsy1_0879955 |
| Afu4g11970 | Ortholog(s) have RNA polymerase I activity, RNA polymerase II activity, RNA polymerase III activity, RNA-directed RNA polymerase activity, zinc ion binding activity |
| Afu4g11990 | Ortholog of A. nidulans FGSC A4 : AN3635, A. niger CBS 513.88 : An01g07270, A. oryzae RIB40 : AO090003001010, Aspergillus wentii : Aspwe1_0022700 and Aspergillus sydowii : Aspsy1_0154807 |
| Afu4g12040 | Putative oxidosqualene:lanosterol cyclase (OSLC) with a role in protostadienol biosynthesis |
| Afu4g12080 | Ortholog(s) have ubiquitin conjugating enzyme activity, ubiquitin-protein transferase activity and role in free ubiquitin chain polymerization, postreplication repair, protein K63-linked ubiquitination |
| Afu4g12210 | Ortholog(s) have 5' overhang single-stranded DNA endodeoxyribonuclease activity, 5'-flap endonuclease activity, ATP-dependent DNA helicase activity and chromatin binding, more |
| Afu4g12220 | Ortholog of A. nidulans FGSC A4 : AN3655, A. niger CBS 513.88 : An01g07500, A. oryzae RIB40 : AO090009000424, Aspergillus wentii : Aspwe1_0166578 and Aspergillus sydowii : Aspsy1_0204923 |
| Afu4g12260 | Has domain(s) with predicted role in transmembrane transport and integral component of membrane localization |
| Afu4g12270 | Has domain(s) with predicted zinc ion binding activity |
| Afu4g12505 | unspecified product |
| Afu4g12510 | Ortholog of A. nidulans FGSC A4 : AN3215, AN7166, AN8609, A. fumigatus Af293 : Afu3g14210, Afu4g03360 and A. niger CBS 513.88 : An09g04200, An14g01840 |
| Afu4g12640 | Member of the RGS (regulator of G-protein signaling) family |
| Afu4g12680 | Ortholog(s) have ATPase activity, G-quadruplex DNA binding, adenylate kinase activity, double-stranded telomeric DNA binding, single-stranded telomeric DNA binding activity |
| Afu4g12690 | Ortholog(s) have protein heterodimerization activity, sequence-specific DNA binding activity |
| Afu4g13170 | G-protein complex beta subunit |
| Afu4g13180 | Tetratricopeptide (TPR) repeat protein, transcript induced by exposure to human airway epithelial cells |
| Afu4g13230 | Ortholog of A. nidulans WetA, a developmental regulatory protein involved in conidial development and activator of conidium-specific gene expression |
| Afu4g13260 | Ortholog of A. nidulans FGSC A4 : AN1934, A. niger CBS 513.88 : An01g08930, A. oryzae RIB40 : AO090009000258, Aspergillus wentii : Aspwe1_0033794 and Aspergillus sydowii : Aspsy1_0139129 |
| Afu4g13290 | Ortholog(s) have cytoplasm, nucleus localization |
| Afu4g13340 | Ortholog(s) have FAD transmembrane transporter activity, calcium channel activity |
| Afu4g13430 | Ortholog of A. nidulans FGSC A4 : AN1955, A. oryzae RIB40 : AO090009000231, Aspergillus wentii : Aspwe1_0022577, Aspergillus sydowii : Aspsy1_0026389 and Aspergillus terreus NIH2624 : ATET_03224 |
| Afu4g13450 | Ortholog(s) have rRNA (guanine-N1-)-methyltransferase activity, role in rRNA modification and endoplasmic reticulum, mitochondrion localization |
| Afu4g13520 | Has domain(s) with predicted oxidoreductase activity and role in metabolic process |
| Afu4g13660 | Ortholog(s) have drug transmembrane transporter activity, polyamine transmembrane transporter activity |
| Afu4g13710 | Ortholog of A. nidulans FGSC A4 : AN5663, A. niger CBS 513.88 : An01g09510, A. oryzae RIB40 : AO090010000145, AO090009000200, Aspergillus wentii : Aspwe1_0106727 and Aspergillus sydowii : Aspsy1_0057029 |
| Afu4g13810 | Ortholog of Aspergillus fumigatus A1163 : AFUB_070740 |
| Afu4g14010 | Ortholog of A. nidulans FGSC A4 : AN5737, AN2721, A. fumigatus Af293 : Afu1g17650, Afu6g06930, A. oryzae RIB40 : AO090005000145, AO090005001229 and Aspergillus wentii : Aspwe1_0134832, Aspwe1_0418839 |
| Afu4g14020 | Ortholog of A. nidulans FGSC A4 : AN2364, A. fumigatus Af293 : Afu8g06990, Neosartorya fischeri NRRL 181 : NFIA_099740 and Aspergillus versicolor : Aspve1_0043947 |
| Afu4g14070 | Putative glycosyl transferase |
| Afu4g14080 | Ortholog of A. nidulans FGSC A4 : AN8368, AN2952, A. fumigatus Af293 : Afu3g07900, Afu8g00900, A. niger CBS 513.88 : An02g11330, An03g05560 and A. oryzae RIB40 : AO090120000471 |
| Afu4g14085 | Ortholog of A. nidulans FGSC A4 : AN8367, A. niger CBS 513.88 : An12g10420, Neosartorya fischeri NRRL 181 : NFIA_102230 and Aspergillus zonatus : Aspzo1_0100142 |
| Afu4g14100 | Predicted glutathione S transferase |
| Afu4g14140 | Has domain(s) with predicted electron carrier activity, heme binding, iron ion binding, oxidoreductase activity, acting on paired donors, with incorporation or reduction of molecular oxygen activity and role in oxidation-reduction process |
| Afu4g14150 | Has domain(s) with predicted transferase activity, transferring acyl groups other than amino-acyl groups, transferase activity, transferring hexosyl groups activity and role in metabolic process |
| Afu4g14175 | Ortholog of Neosartorya fischeri NRRL 181 : NFIA_102130 |
| Afu4g14190 | Ortholog of Aspergillus glaucus : Aspgl1_0029891, Aspgl1_0057413, Aspergillus wentii : Aspwe1_0039585, Aspergillus fumigatus A1163 : AFUB_071340 and Aspergillus clavatus NRRL 1 : ACLA_053100 |
| Afu4g14240 | Has domain(s) with predicted O-methyltransferase activity |
| Afu4g14250 | Ortholog of Aspergillus glaucus : Aspgl1_0079685, Aspergillus flavus NRRL 3357 : AFL2T_04060, Aspergillus versicolor : Aspve1_0080228 and Aspergillus fumigatus A1163 : AFUB_071410 |
| Afu4g14300 | Has domain(s) with predicted GTP binding, GTPase activity |
| Afu4g14350 | Ortholog of A. nidulans FGSC A4 : AN3530, AN2658, A. fumigatus Af293 : Afu1g13760, Afu4g00490, A. niger CBS 513.88 : An09g01580 and A. oryzae RIB40 : AO090166000071 |
| Afu4g14360 | Putative capsular associated protein |
| Afu4g14390 | Predicted DDE1 transposon-related ORF |
| Afu4g14400 | Ortholog of A. nidulans FGSC A4 : AN3546, A. fumigatus Af293 : Afu1g16170, Afu5g15040, Afu6g09300, A. niger CBS 513.88 : An08g11160, An04g08000 and A. oryzae RIB40 : AO090166000001 |
| Afu4g14450 | Mannitol 2-dehydrogenase with a predicted role in mannitol metabolism |
| Afu4g14460 | Putative desmethylsulochrin O-methyltransferase involved in trypacidin biosynthesis, member of the tpc secondary metabolite gene cluster |
| Afu4g14470 | Putative dehydratase involved in trypacidin biosynthesis, member of the tpc secondary metabolite gene cluster |
| Afu4g14510 | Putative sulochrin O-methyltransferase involved in trypacidin biosynthesis, member of the tpc secondary metabolite gene cluster |
| Afu4g14520 | Putative NADH-dependent oxidoreductase involved in trypacidin biosynthesis, member of the tpc secondary metabolite gene cluster |
| Afu4g14540 | Putative Zn2Cys6 transcription factor involved in trypacidin biosynthesis, member of the tpc secondary metabolite gene cluster |
| Afu4g14550 | Putative transcriptional coactivator involved in trypacidin biosynthesis, member of the tpc secondary metabolite gene cluster |
| Afu4g14580 | Putative emodin O-methyltransferase involved in trypacidin biosynthesis, member of the tpc secondary metabolite gene cluster |
| Afu4g14810 | Putative cytochrome P450 monooxygenase with a predicted role in helvonic acid biosynthesis |
| Afu5g00155 | protein of unknown function |
| Afu5g00310 | Putative flavin-containing monooxygenase |
| Afu5g00330 | Has domain(s) with predicted ATP binding, catalytic activity |
| Afu5g00410 | Ortholog(s) have intracellular localization |
| Afu5g00420 | Has domain(s) with predicted role in transmembrane transport and integral component of membrane localization |
| Afu5g00490 | Ortholog of Aspergillus fumigatus A1163 : AFUB_048950 |
| Afu5g00520 | Has domain(s) with predicted DNA binding, RNA polymerase II transcription factor activity, sequence-specific DNA binding, zinc ion binding activity and role in regulation of transcription, DNA-templated, transcription, DNA-templated |
| Afu5g00660 | Ortholog of Aspergillus fumigatus A1163 : AFUB_049130 |
| Afu5g00690 | Ortholog of Aspergillus fumigatus A1163 : AFUB_049160 |
| Afu5g00720 | Has domain(s) with predicted N-acetyltransferase activity |
| Afu5g00840 | Ortholog of A. nidulans FGSC A4 : AN5639, AN2587, AN9444, AN7395, A. niger CBS 513.88 : An03g01000, A. oryzae RIB40 : AO090102000018 and Aspergillus wentii : Aspwe1_0065350, Aspwe1_0153928, Aspwe1_0178709 |
| Afu5g00870 | Ortholog of Aspergillus fumigatus A1163 : AFUB_049320 |
| Afu5g00950 | Has domain(s) with predicted RNA polymerase II transcription factor activity, sequence-specific DNA binding, transcription factor activity, sequence-specific DNA binding, zinc ion binding activity |
| Afu5g00980 | Has domain(s) with predicted role in transmembrane transport and integral component of membrane localization |
| Afu5g00990 | Ortholog of Aspergillus fumigatus A1163 : AFUB_049430 |
| Afu5g01000 | Putative oxidoreductase, 2OG-Fe(II) oxygenase family protein |
| Afu5g01010 | Ortholog of A. fumigatus Af293 : Afu8g00342, A. niger CBS 513.88 : An11g08170, Neosartorya fischeri NRRL 181 : NFIA_041160, NFIA_057730 and Aspergillus fumigatus A1163 : AFUB_049470, AFUB_086230 |
| Afu5g01040 | Ortholog(s) have versicolorin reductase activity and role in monodictyphenone biosynthetic process, sterigmatocystin biosynthetic process, xanthone-containing compound biosynthetic process |
| Afu5g01160 | Ortholog(s) have role in cellular response to drug and plasma membrane localization |
| Afu5g01170 | Ortholog(s) have role in ethanol metabolic process and mitochondrial inner membrane localization |
| Afu5g01190 | Ortholog(s) have alpha-L-fucosidase activity and role in xyloglucan metabolic process |
| Afu5g01250 | Has domain(s) with predicted coenzyme binding, oxidoreductase activity, acting on the CH-OH group of donors, NAD or NADP as acceptor, phosphogluconate dehydrogenase (decarboxylating) activity |
| Afu5g01260 | Ortholog of A. oryzae RIB40 : AO090138000080, Aspergillus flavus NRRL 3357 : AFL2T_08758, Neosartorya fischeri NRRL 181 : NFIA_040860 and Aspergillus fumigatus A1163 : AFUB_049760 |
| Afu5g01360 | Ortholog(s) have oxidoreductase activity and role in steroid metabolic process |
| Afu5g01480 | Ortholog of A. nidulans FGSC A4 : AN10795, AN6721, A. fumigatus Af293 : Afu2g13300, A. niger CBS 513.88 : An16g00890 and A. oryzae RIB40 : AO090011000070, AO090026000292 |
| Afu5g01520 | Major facilitator superfamily protein |
| Afu5g01530 | Has domain(s) with predicted oxidoreductase activity and role in metabolic process |
| Afu5g01540 | Has domain(s) with predicted role in transmembrane transport and integral component of membrane localization |
| Afu5g01680 | Ortholog(s) have endoplasmic reticulum localization |
| Afu5g01690 | Ortholog(s) have cytosol, nucleus localization |
| Afu5g01840 | protein of unknown function |
| Afu5g01860 | Ortholog(s) have role in Arp2/3 complex-mediated actin nucleation, actin cortical patch localization, cellular response to drug, endocytosis and establishment of mitochondrion localization, more |
| Afu5g01880 | Has domain(s) with predicted acid phosphatase activity |
| Afu5g01920 | Ortholog of A. niger CBS 513.88 : An16g01780, A. oryzae RIB40 : AO090003001326, Aspergillus wentii : Aspwe1_0174201, Aspergillus sydowii : Aspsy1_0029040 and Aspergillus terreus NIH2624 : ATET_09813 |
| Afu5g01930 | protein of unknown function |
| Afu5g02140 | Putative inositol polyphosphate phosphatase |
| Afu5g02250 | Ortholog of A. nidulans FGSC A4 : AN8031, A. niger CBS 513.88 : An02g10580, A. oryzae RIB40 : AO090102000288, Aspergillus wentii : Aspwe1_0042830 and Aspergillus sydowii : Aspsy1_0156829 |
| Afu5g02290 | Ortholog(s) have chloride transmembrane transporter activity, potassium uptake transmembrane transporter activity |
| Afu5g02460 | Ortholog of A. nidulans FGSC A4 : AN5465, A. niger CBS 513.88 : An02g10330, An07g05030, A. oryzae RIB40 : AO090102000359, Aspergillus wentii : Aspwe1_0114453 and Aspergillus sydowii : Aspsy1_0156722 |
| Afu5g02500 | protein of unknown function |
| Afu5g02580 | Ortholog(s) have U2-type spliceosomal complex, mitotic spindle pole body localization |
| Afu5g02590 | Ortholog(s) have role in positive regulation of mitotic metaphase/anaphase transition and anaphase-promoting complex, cytosol localization |
| Afu5g02630 | Ortholog of Neosartorya fischeri NRRL 181 : NFIA_039450, Aspergillus fumigatus A1163 : AFUB_051160, Aspergillus clavatus NRRL 1 : ACLA_002530 and Aspergillus terreus NIH2624 : ATET_09232 |
| Afu5g02910 | NAP family protein |
| Afu5g02920 | Ortholog(s) have NAD+ binding, sequence-specific DNA binding activity, role in nitrogen catabolite repression of transcription from RNA polymerase II promoter, regulation of nitrate assimilation and nucleus localization |
| Afu5g03000 | Ortholog(s) have actin monomer binding, protein kinase inhibitor activity, ribosome binding activity, role in negative regulation of protein phosphorylation and cytoplasm, nucleus, polysome, ribosome localization |
| Afu5g03110 | Has domain(s) with predicted arylformamidase activity and role in tryptophan catabolic process to kynurenine |
| Afu5g03130 | Ortholog(s) have sequence-specific DNA binding activity and role in CENP-A containing chromatin organization, kinetochore assembly, mitotic sister chromatid segregation, negative regulation of G0 to G1 transition |
| Afu5g03150 | Ortholog of A. niger CBS 513.88 : An09g05320, A. oryzae RIB40 : AO090102000518, Neosartorya fischeri NRRL 181 : NFIA_038960 and Aspergillus wentii : Aspwe1_0030094 |
| Afu5g03269 | Ortholog of A. niger CBS 513.88 : An09g05520, A. oryzae RIB40 : AO090102000529, Aspergillus wentii : Aspwe1_0043022, Aspergillus sydowii : Aspsy1_0036113 and Aspergillus terreus NIH2624 : ATET_08090 |
| Afu5g03430 | Ortholog(s) have histone demethylase activity (H3-trimethyl-K4 specific) activity |
| Afu5g03450 | Ortholog(s) have 5'-flap endonuclease activity, role in DNA-dependent DNA replication, double-strand break repair via homologous recombination, recombination within rDNA repeats and Slx1-Slx4 complex, nucleolar chromatin localization |
| Afu5g03500 | Ortholog(s) have endoplasmic reticulum localization |
| Afu5g03510 | Ortholog(s) have cytoplasm localization |
| Afu5g03670 | Ortholog(s) have adenosylmethionine decarboxylase activity, role in spermidine biosynthetic process, spermine biosynthetic process and cytosol, nucleus localization |
| Afu5g03700 | Has domain(s) with predicted protein phosphatase inhibitor activity and role in regulation of phosphoprotein phosphatase activity, regulation of signal transduction |
| Afu5g03710 | Ortholog of A. nidulans FGSC A4 : AN8235, A. niger CBS 513.88 : An09g06450, A. oryzae RIB40 : AO090102000581, Aspergillus wentii : Aspwe1_0043078 and Aspergillus sydowii : Aspsy1_0161939 |
| Afu5g03870 | Ortholog(s) have role in maturation of 5.8S rRNA from tricistronic rRNA transcript (SSU-rRNA, 5.8S rRNA, LSU-rRNA), maturation of LSU-rRNA from tricistronic rRNA transcript (SSU-rRNA, 5.8S rRNA, LSU-rRNA) |
| Afu5g03900 | Ortholog(s) have oxidoreductase activity, acting on NAD(P)H, heme protein as acceptor activity, role in heme a biosynthetic process, iron-sulfur cluster assembly, ubiquinone biosynthetic process and mitochondrial matrix localization |
| Afu5g03920 | bZIP transcription factor required for adaption to both iron depletion and excess and for transcriptional activation of the siderophore system |
| Afu5g03970 | Has domain(s) with predicted metallopeptidase activity |
| Afu5g03990 | Vacuolar aspartyl aminopeptidase |
| Afu5g04040 | Ortholog of A. nidulans FGSC A4 : AN12205, A. niger CBS 513.88 : An09g06130, A. oryzae RIB40 : AO090102000607, Aspergillus sydowii : Aspsy1_0061903 and Aspergillus terreus NIH2624 : ATET_08008 |
| Afu5g04060 | Ortholog(s) have ubiquitin-protein transferase activity |
| Afu5g04080 | Putative oxidosqualene:lanosterol cyclase (OSLC) with a role in protostadienol biosynthesis |
| Afu5g04100 | Putative G-protein coupled receptor |
| Afu5g04135 | Ortholog(s) have role in cellular response to nutrient levels, negative regulation of sexual sporulation resulting in formation of a cellular spore |
| Afu5g04170 | Heat shock protein |
| Afu5g04250 | Homocysteine synthase |
| Afu5g04255 | protein of unknown function |
| Afu5g04260 | Putative amino acid permease |
| Afu5g04360 | Ortholog(s) have phosphoenolpyruvate transmembrane transporter activity, role in phosphoenolpyruvate transmembrane import into Golgi lumen and Golgi apparatus localization |
| Afu5g05480 | Ras-related signaling protein |
| Afu5g05560 | Ortholog(s) have GTP diphosphatase activity, ITP diphosphatase activity, UTP diphosphatase activity, XTP diphosphatase activity, dATP pyrophosphohydrolase activity and dCTP diphosphatase activity, more |
| Afu5g05610 | Ortholog(s) have role in cellular bud site selection, mRNA splicing, via spliceosome and U2 snRNP, U2-type spliceosomal complex localization |
| Afu5g05700 | Ortholog(s) have 3-hydroxyacyl-[acyl-carrier-protein] dehydratase activity, role in fatty acid biosynthetic process and mitochondrion localization |
| Afu5g05830 | CorA family metal ion transporter |
| Afu5g05870 | Ortholog(s) have mitotic spindle pole body, nucleus localization |
| Afu5g05910 | Ortholog of A. nidulans FGSC A4 : AN11127, A. oryzae RIB40 : AO090009000671, Neosartorya fischeri NRRL 181 : NFIA_037220, Aspergillus wentii : Aspwe1_0179073 and Aspergillus clavatus NRRL 1 : ACLA_009170 |
| Afu5g05930 | Ortholog(s) have rRNA primary transcript binding activity |
| Afu5g06010 | Ortholog(s) have ribosomal large subunit binding activity |
| Afu5g06050 | Putative mannosyltransferase with a predicted role in glycosylphosphatidylinositol (GPI)-anchor biosynthesis |
| Afu5g06060 | Putative sulfur metabolism regulator |
| Afu5g06080 | Ortholog(s) have role in DNA recombinase assembly, gene conversion at mating-type locus, meiotic DNA repair synthesis, meiotic joint molecule formation, positive regulation of DNA binding, strand invasion |
| Afu5g06140 | Ortholog(s) have histone acetyltransferase activity, role in regulation of transcription from RNA polymerase II promoter, tRNA wobble uridine modification and Elongator holoenzyme complex, cytosol, nucleus localization |
| Afu5g06340 | Ortholog(s) have cell division site, cytosol, nucleus localization |
| Afu5g06490 | Ortholog of A. nidulans FGSC A4 : AN12336, A. niger CBS 513.88 : An17g01160, Neosartorya fischeri NRRL 181 : NFIA_036630, Aspergillus wentii : Aspwe1_0167419 and Aspergillus clavatus NRRL 1 : ACLA_009820 |
| Afu5g06760 | Ortholog(s) have role in mRNA cis splicing, via spliceosome and Prp19 complex, spliceosomal complex localization |
| Afu5g06770 | Ortholog(s) have role in cytoplasmic translation and cell tip, cytoplasm localization |
| Afu5g06780 | Putative carbamoyl-phosphate synthase |
| Afu5g06790 | Ortholog of A. nidulans FGSC A4 : AN2242, A. oryzae RIB40 : AO090701000215, Neosartorya fischeri NRRL 181 : NFIA_036330, Aspergillus wentii : Aspwe1_0034984 and Aspergillus clavatus NRRL 1 : ACLA_010180 |
| Afu5g06820 | Ortholog of A. niger CBS 513.88 : An03g00400, A. oryzae RIB40 : AO090701000306, Neosartorya fischeri NRRL 181 : NFIA_036290 and Aspergillus niger ATCC 1015 : 45807-mRNA |
| Afu5g06830 | Has domain(s) with predicted RNA binding, ribonuclease III activity and role in RNA processing |
| Afu5g06850 | Ortholog of Aspergillus fumigatus A1163 : AFUB_054410 and Aspergillus terreus NIH2624 : ATET_10159 |
| Afu5g06930 | Ortholog of Aspergillus fumigatus A1163 : AFUB_054480 |
| Afu5g06960 | Ortholog(s) have cytosol, nucleus localization |
| Afu5g07060 | Ortholog of A. nidulans FGSC A4 : AN2214, A. niger CBS 513.88 : An17g00280, A. oryzae RIB40 : AO090701000278, Neosartorya fischeri NRRL 181 : NFIA_080200 and Aspergillus versicolor : Aspve1_0080700 |
| Afu5g07140 | Ortholog(s) have role in mitochondrial genome maintenance, regulation of mitochondrial membrane potential and mitochondrion localization |
| Afu5g07160 | Has domain(s) with predicted carbon-sulfur lyase activity and role in metabolic process |
| Afu5g07170 | Ortholog of A. nidulans FGSC A4 : AN2225, A. niger CBS 513.88 : An17g00550, A. oryzae RIB40 : AO090701000254, Aspergillus wentii : Aspwe1_0048029 and Aspergillus sydowii : Aspsy1_0039724 |
| Afu5g07180 | Ortholog(s) have nucleus localization |
| Afu5g07330 | Putative carboxypeptidase S1 |
| Afu5g07370 | Ortholog(s) have metal ion binding, poly(A)-specific ribonuclease activity |
| Afu5g07550 | protein of unknown function |
| Afu5g07560 | Putative capsular associated protein |
| Afu5g07570 | Has domain(s) with predicted ATP binding, catalytic activity, ligase activity and role in metabolic process |
| Afu5g07940 | Has domain(s) with predicted chromatin binding activity |
| Afu5g08010 | Ortholog(s) have protein-N-terminal asparagine amidohydrolase activity, protein-N-terminal glutamine amidohydrolase activity and role in cellular protein modification process, protein catabolic process |
| Afu5g08140 | Ortholog of A. nidulans FGSC A4 : AN7720, A. oryzae RIB40 : AO090701000731, Aspergillus wentii : Aspwe1_0023648, Aspergillus sydowii : Aspsy1_0032289 and Aspergillus terreus NIH2624 : ATET_08313 |
| Afu5g08150 | Ortholog(s) have role in secondary metabolite biosynthetic process |
| Afu5g08170 | Ortholog(s) have Atg8 ligase activity, role in C-terminal protein lipidation, CVT pathway, autophagosome assembly, late nucleophagy, mitophagy, piecemeal microautophagy of nucleus and cytosol, nucleus localization |
| Afu5g08190 | Ortholog of A. nidulans FGSC A4 : AN7717, A. oryzae RIB40 : AO090701000736, Aspergillus wentii : Aspwe1_0101496, Aspergillus sydowii : Aspsy1_0046260 and Aspergillus terreus NIH2624 : ATET_08307 |
| Afu5g08200 | Ortholog of A. nidulans FGSC A4 : AN11006, A. niger CBS 513.88 : An03g04400, A. oryzae RIB40 : AO090701000738, Aspergillus wentii : Aspwe1_0048123 and Aspergillus sydowii : Aspsy1_0046266 |
| Afu5g08230 | Has domain(s) with predicted zinc ion binding activity |
| Afu5g08235 | Has domain(s) with predicted hydrolase activity |
| Afu5g08310 | Has domain(s) with predicted nucleic acid binding, nucleotide binding activity |
| Afu5g08580 | Putative alpha-1,6-mannosyltransferase that initiates the linkage of the N-glycan outer chain |
| Afu5g08620 | Ortholog(s) have fungal-type vacuole localization |
| Afu5g08630 | Ortholog(s) have fungal-type vacuole localization |
| Afu5g08680 | Ortholog(s) have mitochondrion localization |
| Afu5g08770 | Ortholog of Neosartorya fischeri NRRL 181 : NFIA_078530, Aspergillus kawachii : Aspka1_0176490 and Aspergillus carbonarius ITEM 5010 : Acar5010_406577 |
| Afu5g08780 | Putative cell wall glucanase |
| Afu5g08840 | Ortholog(s) have methylated histone binding, transcription factor binding activity |
| Afu5g09060 | Ortholog(s) have U4/U6 x U5 tri-snRNP complex, cytosol localization |
| Afu5g09070 | Ortholog of A. nidulans FGSC A4 : AN4671, A. niger CBS 513.88 : An07g04010, Aspergillus wentii : Aspwe1_0281750, Aspergillus sydowii : Aspsy1_0059434 and Aspergillus terreus NIH2624 : ATET_06560 |
| Afu5g09080 | Ortholog(s) have role in positive regulation of transcription from RNA polymerase II promoter and SAGA complex, cytosol localization |
| Afu5g09130 | Polysaccharide deacetylase family protein |
| Afu5g09260 | Ortholog of A. nidulans FGSC A4 : AN0243, A. niger CBS 513.88 : An07g03750, A. oryzae RIB40 : AO090020000523, Aspergillus wentii : Aspwe1_0101961 and Aspergillus sydowii : Aspsy1_0055067 |
| Afu5g09280 | Ortholog of A. nidulans FGSC A4 : AN4729, A. niger CBS 513.88 : An14g07350, An07g03730, An14g07330, A. oryzae RIB40 : AO090023000535, AO090020000531 and Aspergillus wentii : Aspwe1_0100252, Aspwe1_0111841 |
| Afu5g09290 | Has domain(s) with predicted oxidoreductase activity and role in metabolic process |
| Afu5g09460 | Has domain(s) with predicted ATP binding, ATPase activity, ATPase activity, coupled to transmembrane movement of substances, nucleoside-triphosphatase activity, nucleotide binding activity and role in transport |
| Afu5g09580 | Conidial hydrophobin |
| Afu5g09630 | Ortholog(s) have Golgi apparatus localization |
| Afu5g09640 | Ortholog(s) have role in negative regulation of o-orsellinic acid biosynthetic process |
| Afu5g09680 | Succinate dehydrogenase cytochrome b560 subunit |
| Afu5g09880 | Ortholog of Aspergillus fumigatus A1163 : AFUB_057460 |
| Afu5g09920 | Has domain(s) with predicted hydrolase activity and role in metabolic process |
| Afu5g09930 | Ortholog of A. nidulans FGSC A4 : AN9206, AN1849, A. fumigatus Af293 : Afu4g01210, Afu5g02320, A. niger CBS 513.88 : An03g02680, An12g10080, An12g02060 and A. oryzae RIB40 : AO090001000129, AO090010000537 |
| Afu5g09940 | Ortholog(s) have endoplasmic reticulum localization |
| Afu5g09960 | Putative GPI-anchored protein |
| Afu5g10140 | Has domain(s) with predicted role in transmembrane transport and integral component of membrane localization |
| Afu5g10210 | Ortholog of A. fumigatus Af293 : Afu8g05930, A. niger CBS 513.88 : An14g04490, A. oryzae RIB40 : AO090010000532 and Neosartorya fischeri NRRL 181 : NFIA_077050, NFIA_098690 |
| Afu5g10270 | Ortholog(s) have identical protein binding, mRNA binding, unfolded protein binding activity |
| Afu5g10290 | Putative fructose-bisphosphate aldolase |
| Afu5g10300 | Ortholog of A. nidulans FGSC A4 : AN11200, Neosartorya fischeri NRRL 181 : NFIA_076960, Aspergillus fumigatus A1163 : AFUB_057880 and Aspergillus sydowii : Aspsy1_0036837 |
| Afu5g10310 | Ortholog(s) have sequence-specific DNA binding, transcription factor activity, sequence-specific DNA binding activity |
| Afu5g10340 | Ortholog(s) have extracellular region localization |
| Afu5g10360 | Has domain(s) with predicted actin binding activity |
| Afu5g10440 | Ortholog of Aspergillus fumigatus A1163 : AFUB_058030 |
| Afu5g10610 | Ubiquinol-cytochrome c reductase iron-sulphur subunit precursor with a predicted role in oxidative phosphorylation |
| Afu5g10620 | Has domain(s) with predicted protein heterodimerization activity, sequence-specific DNA binding activity and intracellular localization |
| Afu5g10630 | Has domain(s) with predicted ion channel activity, voltage-gated chloride channel activity, role in chloride transport, transmembrane transport and membrane localization |
| Afu5g10730 | Ortholog of A. nidulans FGSC A4 : AN4732, A. niger CBS 513.88 : An14g03950, A. oryzae RIB40 : AO090010000468, Aspergillus wentii : Aspwe1_0439775 and Aspergillus terreus NIH2624 : ATET_07636 |
| Afu5g11250 | Ortholog of A. oryzae RIB40 : AO090026000720, Aspergillus glaucus : Aspgl1_0052593, Aspergillus flavus NRRL 3357 : AFL2T_06595 and Neosartorya fischeri NRRL 181 : NFIA_076020 |
| Afu5g11260 | GATA transcription factor that regulates iron uptake |
| Afu5g11270 | Ortholog(s) have role in vesicle-mediated transport and COPI-coated vesicle, integral component of Golgi membrane localization |
| Afu5g11340 | Ortholog(s) have enzyme regulator activity, guanyl-nucleotide exchange factor activity, translation initiation factor activity |
| Afu5g11350 | Ortholog(s) have role in attachment of mitotic spindle microtubules to kinetochore and NMS complex, Ndc80 complex, condensed nuclear chromosome kinetochore, meiotic spindle pole body, mitotic spindle pole body localization |
| Afu5g11470 | Ortholog(s) have double-stranded telomeric DNA binding activity, role in telomere maintenance and nuclear chromatin, nuclear chromosome, telomeric region localization |
| Afu5g11480 | Ortholog of Aspergillus fumigatus A1163 : AFUB_059060 |
| Afu5g11500 | Has domain(s) with predicted acid phosphatase activity |
| Afu5g11540 | Ortholog(s) have mitochondrial ribosome localization |
| Afu5g11690 | Putative protein tyrosine phosphatase |
| Afu5g11820 | Ortholog(s) have cytosol, nucleus localization |
| Afu5g12080 | Has domain(s) with predicted DNA binding, RNA polymerase II transcription factor activity, sequence-specific DNA binding, zinc ion binding activity and role in regulation of transcription, DNA-templated, transcription, DNA-templated |
| Afu5g12270 | Ortholog(s) have mRNA binding activity, role in nuclear division, rRNA processing, ribosomal large subunit biogenesis and mitotic spindle pole body, nuclear periphery, nucleolus, preribosome, large subunit precursor localization |
| Afu5g12370 | Ortholog(s) have histone methyltransferase activity (H3-K4 specific) activity |
| Afu5g12520 | Ortholog(s) have role in conidium formation, regulation of conidium formation, sexual sporulation resulting in formation of a cellular spore and plasma membrane localization |
| Afu5g12560 | Ortholog of A. nidulans FGSC A4 : AN0048, A. niger CBS 513.88 : An14g06630, A. oryzae RIB40 : AO090120000382, Aspergillus wentii : Aspwe1_0026710 and Aspergillus sydowii : Aspsy1_0097984 |
| Afu5g12570 | Ortholog(s) have RNA binding activity, role in mRNA splicing, via spliceosome and Lsm1-7-Pat1 complex, U4/U6 x U5 tri-snRNP complex, U6 snRNP, cytoplasm, nucleolus, small nucleolar ribonucleoprotein complex localization |
| Afu5g12580 | Has domain(s) with predicted GTP binding, GTPase activity |
| Afu5g12620 | Has domain(s) with predicted metal ion transmembrane transporter activity, role in metal ion transport, transmembrane transport and membrane localization |
| Afu5g12640 | Ortholog(s) have 3'-5' exonuclease activity, DNA-directed DNA polymerase activity, role in mitochondrial DNA replication and mitochondrion localization |
| Afu5g12875 | unspecified product |
| Afu5g12890 | Ortholog of A. nidulans FGSC A4 : AN6826, A. niger CBS 513.88 : An14g06450, A. oryzae RIB40 : AO090120000402, Aspergillus wentii : Aspwe1_0058891 and Aspergillus sydowii : Aspsy1_0143996 |
| Afu5g12990 | Snf1 protein kinase complex subunit |
| Afu5g13190 | Has domain(s) with predicted ATP binding, microtubule binding, microtubule motor activity, role in microtubule-based movement and microtubule associated complex localization |
| Afu5g13250 | Ortholog of A. nidulans FGSC A4 : AN6885, A. niger CBS 513.88 : An14g04640, A. oryzae RIB40 : AO090120000468, Aspergillus sydowii : Aspsy1_0054799 and Aspergillus terreus NIH2624 : ATET_06186 |
| Afu5g13280 | Ortholog of A. nidulans FGSC A4 : AN6887, A. niger CBS 513.88 : An14g04670, A. oryzae RIB40 : AO090120000470, Aspergillus wentii : Aspwe1_0039072 and Aspergillus sydowii : Aspsy1_0145002 |
| Afu5g13350 | Putative peptidyl-prolyl cis-trans isomerase |
| Afu5g13440 | protein of unknown function |
| Afu5g13450 | Putative triosephosphate isomerase |
| Afu5g13500 | Has domain(s) with predicted zinc ion binding activity |
| Afu5g13670 | Ortholog of Aspergillus fumigatus A1163 : AFUB_061380 |
| Afu5g13680 | Ortholog of Aspergillus fumigatus A1163 : AFUB_061390 |
| Afu5g13700 | protein of unknown function |
| Afu5g13800 | Has domain(s) with predicted FMN binding, oxidoreductase activity and role in oxidation-reduction process |
| Afu5g13820 | Ortholog of Neosartorya fischeri NRRL 181 : NFIA_073110, Aspergillus fumigatus A1163 : AFUB_061560 and Aspergillus clavatus NRRL 1 : ACLA_016800 |
| Afu5g13865 | Ortholog(s) have ATP-dependent RNA helicase inhibitor activity, RNA binding activity |
| Afu5g13900 | Has domain(s) with predicted nucleic acid binding activity |
| Afu5g13920 | Putative Hsp90 binding co-chaperone |
| Afu5g14010 | Ortholog(s) have role in snoRNA metabolic process and cytoplasm, nucleus localization |
| Afu5g14050 | Has domain(s) with predicted catalytic activity and membrane localization |
| Afu5g14060 | Putative Rho-type GTPase |
| Afu5g14090 | Has domain(s) with predicted catalytic activity, hydrolase activity, hydrolyzing O-glycosyl compounds activity and role in carbohydrate metabolic process |
| Afu5g14220 | Ortholog of Aspergillus fumigatus A1163 : AFUB_061920 |
| Afu5g14280 | Ortholog of Aspergillus fumigatus A1163 : AFUB_061990 |
| Afu5g14350 | C-24(28) sterol reductase |
| Afu5g14560 | Has domain(s) with predicted catalytic activity, hydrolase activity, hydrolyzing O-glycosyl compounds activity and role in carbohydrate metabolic process |
| Afu5g14582 | Has domain(s) with predicted catalytic activity and role in nucleoside metabolic process |
| Afu5g14600 | Ortholog of A. nidulans FGSC A4 : AN8144, AN1651, A. fumigatus Af293 : Afu6g00460, Afu6g13950, A. niger CBS 513.88 : An11g02490, An01g11420 and A. oryzae RIB40 : AO090001000384, AO090011000740 |
| Afu5g14610 | Putative carboxypeptidase Y |
| Afu5g14620 | Ortholog of Aspergillus fumigatus A1163 : AFUB_062290 |
| Afu5g14640 | Has domain(s) with predicted hydrolase activity, hydrolase activity, acting on acid halide bonds, in C-halide compounds activity and role in metabolic process |
| Afu5g14680 | Protein with Yap1-dependent induction in response to hydrogen peroxide |
| Afu5g14750 | Ortholog of A. nidulans FGSC A4 : AN0198, A. niger CBS 513.88 : An14g07220, Aspergillus wentii : Aspwe1_0043650, Aspwe1_0044769 and Aspergillus sydowii : Aspsy1_0040680 |
| Afu5g14760 | Ortholog of A. niger CBS 513.88 : An05g01370, A. oryzae RIB40 : AO090001000123, Neosartorya fischeri NRRL 181 : NFIA_072220 and Aspergillus wentii : Aspwe1_0724935 |
| Afu5g14770 | Has domain(s) with predicted oxidoreductase activity, pyrroline-5-carboxylate reductase activity and role in oxidation-reduction process, proline biosynthetic process |
| Afu5g14860 | Has domain(s) with predicted electron carrier activity, heme binding, iron ion binding, oxidoreductase activity, acting on paired donors, with incorporation or reduction of molecular oxygen activity and role in oxidation-reduction process |
| Afu5g14865 | protein of unknown function |
| Afu5g15140 | protein of unknown function |
| Afu6g00110 | Has domain(s) with predicted catalytic activity, lyase activity and role in metabolic process |
| Afu6g00193 | Has domain(s) with predicted metal ion binding activity |
| Afu6g00210 | Ortholog of Aspergillus fumigatus A1163 : AFUB_097310 |
| Afu6g00250 | Ortholog of Aspergillus fumigatus A1163 : AFUB_097270 |
| Afu6g00350 | Ortholog of Aspergillus fumigatus A1163 : AFUB_097170 |
| Afu6g00360 | Ortholog of A. nidulans FGSC A4 : AN5387, A. niger CBS 513.88 : An13g01480, An02g11060, An18g00050 and A. oryzae RIB40 : AO090023000455, AO090003001393, AO090001000640 |
| Afu6g00460 | Ortholog of A. nidulans FGSC A4 : AN8144, AN1651, A. fumigatus Af293 : Afu5g14600, Afu6g13950, A. niger CBS 513.88 : An11g02490, An01g11420 and A. oryzae RIB40 : AO090001000384, AO090011000740 |
| Afu6g00540 | protein of unknown function |
| Afu6g00600 | Ortholog of A. nidulans FGSC A4 : AN7786, A. oryzae RIB40 : AO090663000003, AO090113000133 and Aspergillus wentii : Aspwe1_0041313, Aspwe1_0117236 |
| Afu6g00620 | Putative GPI-anchored protein |
| Afu6g00650 | Ortholog of Aspergillus glaucus : Aspgl1_0031856, Aspergillus fumigatus A1163 : AFUB_096820, Aspergillus zonatus : Aspzo1_0058781 and Aspergillus carbonarius ITEM 5010 : Acar5010_502725 |
| Afu6g00670 | Ortholog of A. nidulans FGSC A4 : AN4815, A. niger CBS 513.88 : An02g13640, Neosartorya fischeri NRRL 181 : NFIA_047830 and Aspergillus versicolor : Aspve1_0082131 |
| Afu6g00710 | Has domain(s) with predicted role in transmembrane transport and integral component of membrane localization |
| Afu6g01800 | Putative endo-1,4 beta-glucanase, a predicted secreted hydrolase with a fungal cellulose binding domain |
| Afu6g01840 | Has domain(s) with predicted DNA binding, RNA polymerase II transcription factor activity, sequence-specific DNA binding, zinc ion binding activity and role in regulation of transcription, DNA-templated, transcription, DNA-templated |
| Afu6g01990 | Ortholog(s) have chromatin binding activity |
| Afu6g02000 | Ortholog(s) have Golgi apparatus localization |
| Afu6g02010 | Secreted protein of unknown function |
| Afu6g02270 | Ortholog(s) have ribonuclease MRP activity, ribonuclease P activity, tRNA binding activity |
| Afu6g02315 | rRNA |
| Afu6g02450 | Ortholog(s) have cytoplasmic stress granule, cytosol, nucleus localization |
| Afu6g02515 | Ortholog of A. nidulans FGSC A4 : AN8711, A. niger CBS 513.88 : An12g07890, Neosartorya fischeri NRRL 181 : NFIA_048790, Aspergillus wentii : Aspwe1_0103407 and Aspergillus versicolor : Aspve1_0044454 |
| Afu6g02620 | Has domain(s) with predicted 2-dehydropantoate 2-reductase activity, NADP binding, coenzyme binding, oxidoreductase activity, oxidoreductase activity, acting on the CH-OH group of donors, NAD or NADP as acceptor activity |
| Afu6g02630 | Ortholog(s) have ATP-dependent RNA helicase activity |
| Afu6g02670 | Putative cell-cycle regulated serine/threonine protein kinase |
| Afu6g02680 | Ortholog(s) have cytosol, ubiquitin ligase complex localization |
| Afu6g02790 | Ortholog(s) have cell division site, cytosol localization |
| Afu6g02920 | Ortholog(s) have SNAP receptor activity and role in Golgi to plasma membrane transport, endocytosis, exocytosis, vesicle fusion |
| Afu6g02960 | Ortholog(s) have mitochondrion localization |
| Afu6g03030 | Has domain(s) with predicted FAD binding, oxidoreductase activity and role in metabolic process |
| Afu6g03040 | Ortholog(s) have endoplasmic reticulum localization |
| Afu6g03130 | protein of unknown function |
| Afu6g03140 | Putative oligopeptide transporter |
| Afu6g03210 | protein of unknown function |
| Afu6g03230 | Putative cell wall glucanase |
| Afu6g03290 | Has domain(s) with predicted catalytic activity, oxidoreductase activity, acting on the CH-OH group of donors, NAD or NADP as acceptor activity and role in metabolic process, oxidation-reduction process |
| Afu6g03300 | Ortholog of A. nidulans FGSC A4 : AN3975, AN9193/llmJ, AN2891 and A. fumigatus Af293 : Afu1g10150, Afu2g04380, Afu3g15150, Afu3g15280 |
| Afu6g03310 | Ortholog of Aspergillus tubingensis : Asptu1_0036561, Aspergillus fumigatus A1163 : AFUB_094980, Aspergillus niger ATCC 1015 : 136205-mRNA and Aspergillus sydowii : Aspsy1_0038192 |
| Afu6g03390 | Predicted glutathione S transferase |
| Afu6g03440 | fructosyl amino acid oxidase, putative |
| Afu6g03450 | Ortholog of A. oryzae RIB40 : AO090001000013/wykH, AO090102000466, Aspergillus flavus NRRL 3357 : AFL2T_07266, AFL2T_09860, Neosartorya fischeri NRRL 181 : NFIA_007610 and Aspergillus versicolor : Aspve1_0401363 |
| Afu6g03570 | Has domain(s) with predicted hydrolase activity, hydrolyzing O-glycosyl compounds activity and role in carbohydrate metabolic process |
| Afu6g03580 | Ortholog(s) have ATP binding, ATPase activity, mRNA binding activity and role in poly(A)+ mRNA export from nucleus, ribosomal small subunit biogenesis |
| Afu6g03590 | Methylcitrate synthase |
| Afu6g03630 | Has domain(s) with predicted catalytic activity and role in metabolic process |
| Afu6g03685 | unspecified product |
| Afu6g03690 | Putative P-type ATPase sodium pump |
| Afu6g03740 | Ortholog(s) have role in ascospore formation |
| Afu6g03750 | Ortholog(s) have amidophosphoribosyltransferase activity, role in 'de novo' IMP biosynthetic process, adenine metabolic process and cytoplasm localization |
| Afu6g04040 | Ortholog(s) have dodecenoyl-CoA delta-isomerase activity, role in fatty acid beta-oxidation, filamentous growth and peroxisome localization |
| Afu6g04090 | Ortholog(s) have mitochondrion localization |
| Afu6g04190 | Ortholog(s) have cytosol, nucleus localization |
| Afu6g04200 | Ortholog(s) have nucleus localization |
| Afu6g04220 | Ortholog(s) have role in retrograde transport, endosome to Golgi and cytosol, late endosome, nucleus localization |
| Afu6g04230 | Ortholog(s) have cytosol, nucleus localization |
| Afu6g04240 | Putative PAP2 domain protein with a predicted role in phospholipids and triacylglycerol biosynthesis |
| Afu6g04265 | Ortholog of A. nidulans FGSC A4 : AN6613, A. niger CBS 513.88 : An15g01490, A. oryzae RIB40 : AO090701000146, Aspergillus wentii : Aspwe1_0322074 and Aspergillus sydowii : Aspsy1_0164382 |
| Afu6g04370 | Ortholog of A. fumigatus Af293 : Afu4g03550, A. niger CBS 513.88 : An01g09070, An07g06910, An12g07420, An01g10470, An09g04030 and A. oryzae RIB40 : AO090010000022, AO090005000964 |
| Afu6g04420 | Ortholog(s) have role in hyphal growth, mitochondrial genome maintenance, mitochondrial outer membrane translocase complex assembly, mitochondrion-ER tethering and phospholipid transport, more |
| Afu6g04460 | Ortholog(s) have cardiolipin synthase activity, role in cellular ion homeostasis, lipid biosynthetic process, mitochondrial genome maintenance, mitochondrial membrane organization and mitochondrion localization |
| Afu6g04465 | rRNA |
| Afu6g04470 | Ortholog of A. nidulans FGSC A4 : AN6579, A. niger CBS 513.88 : An15g01120, A. oryzae RIB40 : AO090701000109, Aspergillus wentii : Aspwe1_0179993 and Aspergillus terreus NIH2624 : ATET_07015 |
| Afu6g04530 | Ortholog(s) have role in histone acetylation and NuA4 histone acetyltransferase complex localization |
| Afu6g04540 | Calcineurin regulatory subunit |
| Afu6g04640 | Ortholog(s) have role in medium-chain fatty acid biosynthetic process |
| Afu6g04645 | protein of unknown function |
| Afu6g04710 | Has domain(s) with predicted phospholipid binding activity |
| Afu6g04770 | Ortholog of Aspergillus fumigatus A1163 : AFUB_093520 and Aspergillus niger ATCC 1015 : 127270-mRNA |
| Afu6g04790 | Ortholog(s) have role in proteasomal ubiquitin-independent protein catabolic process, proteasome-mediated ubiquitin-dependent protein catabolic process |
| Afu6g04800 | Ortholog(s) have role in cellular response to drug, response to purine-containing compound and cytoplasm, nucleus localization |
| Afu6g04920 | Putative NAD-dependent formate dehydrogenase |
| Afu6g04930 | Ortholog of A. nidulans FGSC A4 : AN6524, A. niger CBS 513.88 : An15g00400, A. oryzae RIB40 : AO090701000045, Aspergillus wentii : Aspwe1_0036161 and Aspergillus sydowii : Aspsy1_0037126 |
| Afu6g04960 | Ortholog of A. niger CBS 513.88 : An15g00360, A. oryzae RIB40 : AO090701000042, Neosartorya fischeri NRRL 181 : NFIA_051520 and Aspergillus wentii : Aspwe1_0327865 |
| Afu6g04970 | Ortholog(s) have O-phospho-L-serine:2-oxoglutarate aminotransferase activity, role in L-serine biosynthetic process, purine nucleobase biosynthetic process and cytosol localization |
| Afu6g04980 | Has domain(s) with predicted oxidoreductase activity and role in oxidation-reduction process |
| Afu6g05050 | Ortholog(s) have role in posttranslational protein targeting to membrane, protein insertion into ER membrane and TRC complex, nucleus localization |
| Afu6g05060 | Ortholog(s) have GDP binding, RNA pyrophosphohydrolase activity, enzyme regulator activity, phosphodiesterase decapping endonuclease activity |
| Afu6g05140 | Putative sterol delta 5,6-desaturase-like protein with similarity to Erg3 |
| Afu6g05150 | Ortholog(s) have histone binding activity |
| Afu6g05190 | Ortholog(s) have cytoplasm localization |
| Afu6g05200 | 60S ribosomal protein L28 |
| Afu6g05330 | Ortholog(s) have structural constituent of ribosome activity and mitochondrial small ribosomal subunit localization |
| Afu6g05350 | Putative aspartic-type endopeptidase |
| Afu6g06490 | Has domain(s) with predicted nucleic acid binding, nucleotide binding activity |
| Afu6g06520 | Ortholog(s) have role in hypotonic response, intracellular transport, syncytium formation by plasma membrane fusion and cell septum, cytoplasmic stress granule localization |
| Afu6g06580 | Ortholog(s) have phosphomannomutase activity, role in establishment or maintenance of cell polarity, protein targeting to ER and cytosol, nucleus localization |
| Afu6g06610 | Ortholog(s) have role in protein folding and endoplasmic reticulum localization |
| Afu6g06620 | Ortholog(s) have role in ER to Golgi vesicle-mediated transport and ER to Golgi transport vesicle, Golgi apparatus, endoplasmic reticulum localization |
| Afu6g06630 | Ortholog of A. nidulans FGSC A4 : AN10705, A. niger CBS 513.88 : An18g06430, A. oryzae RIB40 : AO090003000039, Neosartorya fischeri NRRL 181 : NFIA_052280 and Aspergillus wentii : Aspwe1_0170257 |
| Afu6g06920 | Ortholog(s) have methionyl-tRNA formyltransferase activity, role in conversion of methionyl-tRNA to N-formyl-methionyl-tRNA and mitochondrion localization |
| Afu6g06930 | Has domain(s) with predicted oxidoreductase activity and role in metabolic process |
| Afu6g06950 | Ortholog of A. nidulans FGSC A4 : AN5735, A. oryzae RIB40 : AO090003001560, Aspergillus wentii : Aspwe1_0152429, Aspergillus sydowii : Aspsy1_0043696 and Aspergillus terreus NIH2624 : ATET_02919 |
| Afu6g07055 | Ortholog of A. nidulans FGSC A4 : AN11468, A. niger CBS 513.88 : An07g09310, Neosartorya fischeri NRRL 181 : NFIA_052700, Aspergillus wentii : Aspwe1_0519305 and Aspergillus versicolor : Aspve1_0087640 |
| Afu6g07270 | Putative fatty acid CoA synthetase |
| Afu6g07280 | Putative ABC transporter family protein |
| Afu6g07420 | Ortholog of A. nidulans FGSC A4 : AN5209, A. niger CBS 513.88 : An07g09010, A. oryzae RIB40 : AO090005001558, Aspergillus wentii : Aspwe1_0516548 and Aspergillus sydowii : Aspsy1_0156708 |
| Afu6g07460 | Ortholog of A. nidulans FGSC A4 : AN5212, A. niger CBS 513.88 : An07g08970, Aspergillus wentii : Aspwe1_0028223, Aspergillus sydowii : Aspsy1_0033560 and Aspergillus terreus NIH2624 : ATET_03679 |
| Afu6g07490 | Ortholog(s) have role in endocytic recycling and cytosol, endosome, nucleus localization |
| Afu6g07600 | Ortholog of A. nidulans FGSC A4 : AN6023, A. niger CBS 513.88 : An19g00060, A. oryzae RIB40 : AO090003000191, AO090102000004 and Aspergillus wentii : Aspwe1_0121344 |
| Afu6g07610 | Ortholog of A. nidulans FGSC A4 : AN1910, A. oryzae RIB40 : AO090003000190, Aspergillus wentii : Aspwe1_0715572, Aspergillus sydowii : Aspsy1_0039036 and Aspergillus terreus NIH2624 : ATET_05909 |
| Afu6g07620 | GDP-mannose pyrophosphorylase A |
| Afu6g07670 | Cytochrome c oxidase assembly protein with a predicted role in oxidative phosphorylation |
| Afu6g07690 | Ortholog(s) have mRNA (guanine-N7-)-methyltransferase activity and role in 7-methylguanosine mRNA capping, RNA (guanine-N7)-methylation, cellular response to drug, mRNA methylation |
| Afu6g07710 | Has domain(s) with predicted role in transmembrane transport |
| Afu6g07720 | Putative phosphoenolpyruvate carboxykinase |
| Afu6g07855 | Ortholog of A. nidulans FGSC A4 : AN1941, A. niger CBS 513.88 : An11g02730, Neosartorya fischeri NRRL 181 : NFIA_053540 and Aspergillus versicolor : Aspve1_0025340 |
| Afu6g07940 | Putative lactoylglutathione lyase |
| Afu6g07970 | Putative calpain-like protein |
| Afu6g08010 | Ortholog of A. nidulans FGSC A4 : AN3956, A. niger CBS 513.88 : An11g02120, A. oryzae RIB40 : AO090003001561, Aspergillus wentii : Aspwe1_0055020 and Aspergillus sydowii : Aspsy1_0149662 |
| Afu6g08070 | Ortholog(s) have role in RNA metabolic process and cytoplasmic stress granule, nucleus, ribosome localization |
| Afu6g08110 | Has domain(s) with predicted DNA binding, zinc ion binding activity, role in DNA repair and intracellular localization |
| Afu6g08120 | Ortholog(s) have histone kinase activity (H2A-S121 specific) activity |
| Afu6g08170 | Ortholog(s) have rDNA binding activity, role in transcription of nuclear large rRNA transcript from RNA polymerase I promoter and cytosol, nucleolus localization |
| Afu6g08310 | Putative proteosome component |
| Afu6g08440 | Predicted oxygen-dependent protoporphyrinogen oxidase |
| Afu6g08530 | Ortholog(s) have role in attachment of telomere to nuclear envelope, maintenance of rDNA, mitotic sister chromatid segregation, nuclear envelope organization |
| Afu6g08570 | Ortholog(s) have role in error-prone translesion synthesis and mitotic spindle, nucleolus, site of double-strand break localization |
| Afu6g08580 | Putative FK506-binding protein (FKBP)-type peptidyl-prolyl cis-trans isomerase |
| Afu6g08730 | Has domain(s) with predicted NADP binding, coenzyme binding, oxidoreductase activity, acting on the CH-OH group of donors, NAD or NADP as acceptor, phosphogluconate dehydrogenase (decarboxylating) activity |
| Afu6g08800 | Ortholog(s) have nucleolus localization |
| Afu6g08880 | Ortholog(s) have cytosol, nucleus localization |
| Afu6g08930 | Ortholog(s) have endoplasmic reticulum, mitochondrial outer membrane localization |
| Afu6g09000 | Ortholog(s) have chromatin binding, methylated histone binding activity and role in negative regulation of transcription from RNA polymerase II promoter |
| Afu6g09060 | Ortholog(s) have structural constituent of ribosome activity and mitochondrial large ribosomal subunit, nucleus localization |
| Afu6g09100 | Has domain(s) with predicted catalytic activity |
| Afu6g09170 | Ortholog(s) have 3-methyl-2-oxobutanoate hydroxymethyltransferase activity, role in pantothenate biosynthetic process, respiratory chain complex IV assembly and mitochondrion localization |
| Afu6g09240 | Has domain(s) with predicted ATP binding, protein kinase activity, protein tyrosine kinase activity and role in protein phosphorylation |
| Afu6g09300 | Has domain(s) with predicted DNA binding activity and role in DNA integration, DNA recombination |
| Afu6g09350 | Ortholog of Aspergillus fumigatus A1163 : AFUB_092740 |
| Afu6g09353 | Ortholog of Aspergillus fumigatus A1163 : AFUB_092730 |
| Afu6g09360 | Ortholog of Aspergillus fumigatus A1163 : AFUB_092710 |
| Afu6g09410 | Ortholog of Aspergillus fumigatus A1163 : AFUB_092640 |
| Afu6g09450 | Ortholog of A. niger CBS 513.88 : An04g00640, Neosartorya fischeri NRRL 181 : NFIA_112440, Aspergillus wentii : Aspwe1_0731886 and Aspergillus versicolor : Aspve1_0056875, Aspve1_0755767 |
| Afu6g09500 | Ortholog of A. niger CBS 513.88 : An12g06970, An05g02130, Neosartorya fischeri NRRL 181 : NFIA_055150, Aspergillus fumigatus A1163 : AFUB_075550 and Aspergillus aculeatus ATCC16872 : Aacu16872_046735 |
| Afu6g09600 | Has domain(s) with predicted hydrolase activity and role in metabolic process |
| Afu6g09650 | Predicted membrane dipeptidase, encoded in the putative gliotoxin biosynthetic gene cluster |
| Afu6g09680 | Predicted O-methyltransferase, encoded in the putative gliotoxin biosynthetic gene cluster |
| Afu6g09690 | Glutathione S-transferase encoded in the gliotoxin biosynthetic gene cluster |
| Afu6g09700 | Protein essential for gliotoxin biosynthesis, encoded in the putative gliotoxin biosynthetic gene cluster |
| Afu6g09710 | Predicted major facilitator type glioxin transporter, encoded in the putative gliotoxin biosynthetic gene cluster |
| Afu6g09720 | Predicted methyltransferase, encoded in the putative gliotoxin biosynthetic gene cluster |
| Afu6g09730 | Predicted cytochrome P450 monooxygenase, encoded in the putative gliotoxin biosynthetic gene cluster |
| Afu6g09745 | Ortholog of Aspergillus brasiliensis : Aspbr1_0199026, Neosartorya fischeri NRRL 181 : NFIA_055440, NFIA_112700, Aspergillus fumigatus A1163 : AFUB_075800 and Aspergillus clavatus NRRL 1 : ACLA_006680 |
| Afu6g09760 | Has domain(s) with predicted electron carrier activity, heme binding, iron ion binding, oxidoreductase activity, acting on paired donors, with incorporation or reduction of molecular oxygen activity and role in oxidation-reduction process |
| Afu6g09770 | Geranylgeranyl diphosphate synthase |
| Afu6g09830 | Ortholog of A. nidulans FGSC A4 : AN7528, Neosartorya fischeri NRRL 181 : NFIA_055550, Aspergillus wentii : Aspwe1_0176933 and Aspergillus versicolor : Aspve1_0789753 |
| Afu6g09880 | Has domain(s) with predicted role in transmembrane transport and integral component of membrane localization |
| Afu6g09930 | bZIP family transcription factor |
| Afu6g10130 | Putative N,O-diacetyl muramidase |
| Afu6g10160 | Ortholog of Aspergillus fumigatus A1163 : AFUB_076220 |
| Afu6g10170 | Ortholog of Aspergillus fumigatus A1163 : AFUB_076230 |
| Afu6g10290 | Predicted GPI anchored protein |
| Afu6g10310 | Has domain(s) with predicted RNA polymerase II transcription factor activity, sequence-specific DNA binding, zinc ion binding activity, role in regulation of transcription, DNA-templated and nucleus localization |
| Afu6g10320 | Ortholog(s) have unfolded protein binding activity, role in ribosome biogenesis and cytosol, nucleolus localization |
| Afu6g10330 | Ortholog(s) have GTPase activity, role in cellular protein localization, exit from mitosis, mitotic M phase, positive regulation of barrier septum assembly, septation initiation signaling and mitotic spindle pole body localization |
| Afu6g10490 | Has domain(s) with predicted DNA binding activity, role in DNA replication and nuclear origin of replication recognition complex localization |
| Afu6g10530 | Ortholog(s) have ubiquitin-protein transferase activity and role in global genome nucleotide-excision repair, protein ubiquitination involved in ubiquitin-dependent protein catabolic process |
| Afu6g10610 | Putative ribose 5-phosphate isomerase A |
| Afu6g10760 | Ortholog(s) have cytosol, nucleus localization |
| Afu6g10780 | Ortholog of A. nidulans FGSC A4 : AN6383, A. fumigatus Af293 : Afu5g00450, A. niger CBS 513.88 : An03g05860 and A. oryzae RIB40 : AO090003001528, AO090023001010 |
| Afu6g10880 | Putative acyl-CoA dehydrogenase |
| Afu6g10890 | Ortholog(s) have role in receptor-mediated endocytosis and actin cortical patch localization |
| Afu6g10920 | Predicted DDE1 transposon-related ORF |
| Afu6g11030 | Has domain(s) with predicted nucleic acid binding, nucleotide binding activity |
| Afu6g11040 | Flavin mononucleotide adenylyltransferase (FMNAT) |
| Afu6g11060 | Ortholog(s) have nucleus localization |
| Afu6g11070 | Ortholog(s) have cytosol, nucleus localization |
| Afu6g11090 | Ortholog of Aspergillus fumigatus A1163 : AFUB_077110 |
| Afu6g11340 | Has domain(s) with predicted catalytic activity and role in metabolic process |
| Afu6g11360 | Ortholog(s) have mitochondrion localization |
| Afu6g11470 | Serine/threonine phosphatase required for cell wall integrity, biofilm formation, adhesion and virulence |
| Afu6g11500 | Putative dipeptidase |
| Afu6g11570 | Ortholog of A. nidulans FGSC A4 : AN10520, A. niger CBS 513.88 : An13g01940, A. oryzae RIB40 : AO090023000421, Aspergillus wentii : Aspwe1_0471625 and Aspergillus terreus NIH2624 : ATET_05006 |
| Afu6g11590 | Has domain(s) with predicted peroxiredoxin activity and role in oxidation-reduction process |
| Afu6g11620 | Formyltetrahydrofolate deformylase |
| Afu6g11660 | Has domain(s) with predicted hydrolase activity and role in metabolic process |
| Afu6g11780 | Ortholog of Aspergillus tubingensis : Asptu1_0169431, Aspergillus brasiliensis : Aspbr1_0051505, Aspergillus kawachii : Aspka1_0174733 and Aspergillus acidus : Aspfo1_0204257 |
| Afu6g11910 | Has domain(s) with predicted hydrolase activity, hydrolyzing O-glycosyl compounds activity and role in carbohydrate metabolic process |
| Afu6g11930 | protein of unknown function |
| Afu6g11950 | Transcript up-regulated in conidia exposed to neutrophils |
| Afu6g11960 | Has domain(s) with predicted catalytic activity and role in carbohydrate metabolic process |
| Afu6g12070 | Flavoprotein amide oxidase (FAD) |
| Afu6g12140 | Ortholog of A. nidulans FGSC A4 : AN3529, AN4144, AN8311, AN9211, AN4846 and A. fumigatus Af293 : Afu1g17260, Afu4g07950, Afu4g09380, Afu6g00560, Afu8g01900 |
| Afu6g12190 | Ortholog(s) have role in cellular response to farnesol, cellular response to heat, cellular response to hydrogen peroxide, sterigmatocystin biosynthetic process |
| Afu6g12240 | Putative glycerophosphoryl diester phosphodiesterase, phoB-regulated |
| Afu6g12270 | Ortholog(s) have ubiquitin-specific protease activity, role in protein deubiquitination, ubiquitin homeostasis, ubiquitin-dependent protein catabolic process via the multivesicular body sorting pathway and cytosol localization |
| Afu6g12280 | Ortholog(s) have role in mitochondrial respiratory chain complex I assembly and mitochondrial membrane localization |
| Afu6g12470 | Ortholog(s) have cell division site, cytosol, nucleus localization |
| Afu6g12490 | Ortholog of S. cerevisiae : AIM1, A. nidulans FGSC A4 : AN3686, A. niger CBS 513.88 : An06g01650, Aspergillus wentii : Aspwe1_0166410 and Aspergillus versicolor : Aspve1_0042056 |
| Afu6g12550 | Ortholog(s) have iron ion transmembrane transporter activity, role in RNA splicing, iron ion homeostasis, mitochondrial iron ion transport and mitochondrion, plasma membrane localization |
| Afu6g12570 | Putative Epsin2 |
| Afu6g12590 | Ortholog of Aspergillus fumigatus A1163 : AFUB_078590 |
| Afu6g12720 | 40S ribosomal protein S29 |
| Afu6g12870 | ABC iron exporter |
| Afu6g12900 | Has domain(s) with predicted role in transmembrane transport |
| Afu6g12940 | Has domain(s) with predicted hydrolase activity |
| Afu6g12980 | Ortholog(s) have microtubule plus-end binding activity |
| Afu6g13080 | Ortholog(s) have DNA helicase activity, role in DNA unwinding involved in DNA replication, reciprocal meiotic recombination, synapsis and nucleus localization |
| Afu6g13160 | serine/threonine protein kinase |
| Afu6g13170 | Ortholog(s) have APC-Cdc20 complex activity, ubiquitin conjugating enzyme activity |
| Afu6g13220 | Ortholog of A. nidulans FGSC A4 : AN5489, A. niger CBS 513.88 : An08g10180, A. oryzae RIB40 : AO090003000447, Aspergillus wentii : Aspwe1_0166336 and Aspergillus sydowii : Aspsy1_0886379 |
| Afu6g13300 | GTP-Binding nuclear protein Ran |
| Afu6g13340 | Ortholog(s) have hypoxanthine DNA N-glycosylase activity, oxanine DNA N-glycosylase activity, uracil DNA N-glycosylase activity, xanthine DNA N-glycosylase activity and nucleus localization |
| Afu6g13470 | Has domain(s) with predicted hydrolase activity |
| Afu6g13700 | Ortholog of Aspergillus fumigatus A1163 : AFUB_001040 |
| Afu6g13740 | Ortholog of A. nidulans FGSC A4 : AN0723, A. fumigatus Af293 : Afu1g13990, A. niger CBS 513.88 : An08g07150, An05g01960 and A. oryzae RIB40 : AO090103000371 |
| Afu6g13780 | Has domain(s) with predicted role in transmembrane transport and integral component of membrane localization |
| Afu6g13880 | Has domain(s) with predicted role in transmembrane transport and integral component of membrane localization |
| Afu6g13920 | Putative adenylate-forming enzyme |
| Afu6g14070 | Ortholog of Aspergillus fumigatus A1163 : AFUB_000670 |
| Afu6g14160 | Ortholog of A. nidulans FGSC A4 : AN6432, AN5348, A. niger CBS 513.88 : An09g01260, An06g01070, Aspergillus wentii : Aspwe1_0040163, Aspwe1_0166152 and Aspergillus sydowii : Aspsy1_0160977 |
| Afu6g14270 | Ortholog of Neosartorya fischeri NRRL 181 : NFIA_060150 |
| Afu6g14320 | Ortholog of A. nidulans FGSC A4 : AN10663, A. niger CBS 513.88 : An08g08150, A. oryzae RIB40 : AO090103000451, Neosartorya fischeri NRRL 181 : NFIA_060200 and Aspergillus wentii : Aspwe1_0166136 |
| Afu6g14330 | Ortholog(s) have cytosol localization |
| Afu6g14370 | AraC-like ligand binding domain protein |
| Afu6g14400 | Ortholog of A. nidulans FGSC A4 : AN5333/plyC, A. oryzae RIB40 : AO090102000072, Aspergillus flavus NRRL 3357 : AFL2T_09523 and Neosartorya fischeri NRRL 181 : NFIA_060270 |
| Afu6g14410 | Ortholog(s) have cytosol localization |
| Afu6g14510 | Has domain(s) with predicted FAD binding, oxidoreductase activity and role in metabolic process |
| Afu6g14570 | Ortholog of A. fumigatus Af293 : Afu5g00820, A. oryzae RIB40 : AO090009000539, Aspergillus wentii : Aspwe1_0037901, Aspergillus sydowii : Aspsy1_0044497 and Aspergillus terreus NIH2624 : ATET_07141 |
| Afu7g00170 | Dimethylallyl tryptophan synthase |
| Afu7g00570 | Ortholog of A. fumigatus Af293 : Afu2g17160, Afu3g13630, A. niger CBS 513.88 : An15g01210, An09g06230, An09g04210, An01g07840 and Neosartorya fischeri NRRL 181 : NFIA_095980 |
| Afu7g00580 | Putative adhesin |
| Afu7g00630 | Has domain(s) with predicted oxidoreductase activity, acting on the aldehyde or oxo group of donors, NAD or NADP as acceptor activity and role in oxidation-reduction process |
| Afu7g00770 | Has domain(s) with predicted RNA polymerase II transcription factor activity, sequence-specific DNA binding, transcription factor activity, sequence-specific DNA binding, zinc ion binding activity |
| Afu7g00780 | Has domain(s) with predicted role in transmembrane transport and integral component of membrane localization |
| Afu7g00800 | Has domain(s) with predicted acid phosphatase activity, hydrolase activity, metal ion binding activity |
| Afu7g00840 | Has domain(s) with predicted oxidoreductase activity and role in metabolic process |
| Afu7g00860 | Has domain(s) with predicted metal ion binding, oxidoreductase activity, acting on single donors with incorporation of molecular oxygen, incorporation of two atoms of oxygen activity and role in oxidation-reduction process |
| Afu7g00930 | Ortholog of A. nidulans FGSC A4 : AN6413, AN6419, AN6946, AN5312, AN5664, AN8328, AN3241, AN10369, AN1930, AN2386, AN1738, AN11159, AN8984, AN8951, AN1540, AN8971, AN9387, AN9266, AN9306, AN1317, AN10886, AN7232, AN7774, AN12202 |
| Afu7g01000 | Putative alcohol dehydrogenase involved in ethanol metabolism |
| Afu7g01020 | Ortholog of Aspergillus fumigatus A1163 : AFUB_087600 |
| Afu7g01040 | Has domain(s) with predicted hydrolase activity, zinc ion binding activity |
| Afu7g01118 | Ortholog of Neosartorya fischeri NRRL 181 : NFIA_113820 and Aspergillus fumigatus A1163 : AFUB_087700 |
| Afu7g01200 | Putative aspergillopepsin |
| Afu7g01320 | Glycoside hydrolase |
| Afu7g01340 | Putative RPEL repeat protein |
| Afu7g01430 | Putative bacterial rhodopsin Family G-protein coupled receptor (GPCR)-like protein |
| Afu7g01450 | Has domain(s) with predicted electron carrier activity, heme binding, iron ion binding, oxidoreductase activity, acting on paired donors, with incorporation or reduction of molecular oxygen activity and role in oxidation-reduction process |
| Afu7g01490 | Major facilitator superfamily (MFS) peptide transporter |
| Afu7g01640 | Ortholog(s) have sequence-specific DNA binding activity |
| Afu7g01680 | Ortholog of A. niger CBS 513.88 : An12g01030, Neosartorya fischeri NRRL 181 : NFIA_114400, Aspergillus wentii : Aspwe1_0582797 and Aspergillus versicolor : Aspve1_0057666 |
| Afu7g01720 | Has domain(s) with predicted catalytic activity |
| Afu7g01740 | Ortholog(s) have endoplasmic reticulum localization |
| Afu7g01750 | Ortholog of A. nidulans FGSC A4 : AN1239, A. niger CBS 513.88 : An12g00960, A. oryzae RIB40 : AO090120000009, Aspergillus wentii : Aspwe1_0045721 and Aspergillus sydowii : Aspsy1_0085157 |
| Afu7g01820 | Has domain(s) with predicted DNA binding, RNA polymerase II transcription factor activity, sequence-specific DNA binding, zinc ion binding activity and role in regulation of transcription, DNA-templated, transcription, DNA-templated |
| Afu7g01840 | Ortholog(s) have Golgi apparatus, cell tip, cytoplasmic vesicle, endoplasmic reticulum, plasma membrane localization |
| Afu7g01850 | Ortholog(s) have RNA polymerase III activity, role in tRNA transcription from RNA polymerase III promoter and DNA-directed RNA polymerase III complex, cytosol, mitochondrion localization |
| Afu7g01940 | Ortholog(s) have nuclear pore localization |
| Afu7g01950 | Ortholog of A. nidulans FGSC A4 : AN11176, A. oryzae RIB40 : AO090038000574, Neosartorya fischeri NRRL 181 : NFIA_114680 and Aspergillus versicolor : Aspve1_0047813 |
| Afu7g01960 | Ortholog(s) have role in filamentous growth, growth of symbiont in host |
| Afu7g02030 | Ortholog of A. nidulans FGSC A4 : AN11175, A. niger CBS 513.88 : An12g00650, Neosartorya fischeri NRRL 181 : NFIA_114750, Aspergillus wentii : Aspwe1_0112582 and Aspergillus clavatus NRRL 1 : ACLA_065500 |
| Afu7g02040 | Ortholog(s) have triglyceride lipase activity, role in triglyceride catabolic process and mitochondrion localization |
| Afu7g02060 | Ortholog of A. niger CBS 513.88 : An12g00610, A. oryzae RIB40 : AO090038000583, Neosartorya fischeri NRRL 181 : NFIA_114790 and Aspergillus wentii : Aspwe1_0579937 |
| Afu7g02140 | 40S ribosomal protein S24 |
| Afu7g02200 | Ortholog(s) have ATP binding, DNA-dependent ATPase activity, double-stranded DNA binding, recombinase activity, single-stranded DNA binding activity |
| Afu7g02230 | Ortholog(s) have mRNA binding activity, role in arginine catabolic process, positive regulation of cellular response to oxidative stress, positive regulation of nuclear-transcribed mRNA poly(A) tail shortening and cytosol localization |
| Afu7g02280 | Ortholog(s) have U5 snRNP localization |
| Afu7g02500 | Ortholog of A. nidulans FGSC A4 : AN9069, A. niger CBS 513.88 : An12g00100, A. oryzae RIB40 : AO090038000626, Aspergillus wentii : Aspwe1_0136265 and Aspergillus sydowii : Aspsy1_0061378 |
| Afu7g03640 | Ortholog(s) have nucleus localization |
| Afu7g03730 | Ortholog(s) have mitochondrial ribosome localization |
| Afu7g03770 | Transcript up-regulated in conidia exposed to neutrophils |
| Afu7g03820 | Ortholog(s) have role in nucleotide-excision repair, pyrimidine dimer repair and nucleotide-excision repair complex localization |
| Afu7g03910 | Ortholog(s) have role in conidium formation, negative regulation of conidium formation and positive regulation of sexual sporulation resulting in formation of a cellular spore, more |
| Afu7g03990 | Uridylate kinase |
| Afu7g04000 | Ortholog of A. niger CBS 513.88 : An13g00450, Neosartorya fischeri NRRL 181 : NFIA_024960, Aspergillus wentii : Aspwe1_0430380 and Aspergillus terreus NIH2624 : ATET_04281 |
| Afu7g04010 | Ortholog of A. nidulans FGSC A4 : AN4257, A. fumigatus Af293 : Afu6g12230, A. niger CBS 513.88 : An13g00460, An02g13770 and A. oryzae RIB40 : AO090026000813, AO090120000195 |
| Afu7g04090 | Has domain(s) with predicted mitochondrion localization |
| Afu7g04290 | Putative amino acid permease |
| Afu7g04350 | Ortholog(s) have mannonate dehydratase activity and role in cellular carbohydrate catabolic process |
| Afu7g04360 | Has domain(s) with predicted oxidoreductase activity and role in metabolic process |
| Afu7g04390 | Ortholog of A. nidulans FGSC A4 : AN3742, A. niger CBS 513.88 : An13g00970, A. oryzae RIB40 : AO090005000126, Aspergillus wentii : Aspwe1_0170372 and Aspergillus sydowii : Aspsy1_0149869 |
| Afu7g04420 | Ortholog(s) have mitochondrial ribosome localization |
| Afu7g04430 | Ortholog(s) have single-stranded RNA binding activity |
| Afu7g04480 | Ortholog(s) have role in gene conversion at mating-type locus, termination of copy-synthesis, maintenance of DNA repeat elements, reciprocal meiotic recombination and nucleus, site of double-strand break localization |
| Afu7g04490 | Ortholog(s) have structural constituent of ribosome activity |
| Afu7g04540 | Has domain(s) with predicted oxidoreductase activity and role in metabolic process |
| Afu7g04580 | Putative TBC domain protein with a predicted role in the regulation of plasma membrane and endosome trafficking |
| Afu7g04760 | Gamma-glutamyltranspeptidase |
| Afu7g04890 | Has domain(s) with predicted DNA binding, RNA polymerase II transcription factor activity, sequence-specific DNA binding, zinc ion binding activity and role in regulation of transcription, DNA-templated, transcription, DNA-templated |
| Afu7g04920 | protein of unknown function |
| Afu7g04930 | Putative alkaline serine protease |
| Afu7g04960 | Has domain(s) with predicted N-acetyltransferase activity |
| Afu7g05030 | Ortholog(s) have pectin lyase activity, role in pectin catabolic process and extracellular region localization |
| Afu7g05040 | Ortholog of Neosartorya fischeri NRRL 181 : NFIA_026130 and Aspergillus fumigatus A1163 : AFUB_090610 |
| Afu7g05210 | Ortholog(s) have CTP synthase activity, role in CTP biosynthetic process, negative regulation of G0 to G1 transition, phospholipid biosynthetic process, pyrimidine nucleobase biosynthetic process and cytoophidium, cytoplasm localization |
| Afu7g05250 | Ortholog(s) have methylated histone binding activity, role in histone acetylation and NuA3a histone acetyltransferase complex, NuA3b histone acetyltransferase complex, cytoplasm localization |
| Afu7g05320 | Ortholog(s) have oxidized purine nucleobase lesion DNA N-glycosylase activity and role in base-excision repair, AP site formation, error-free translesion synthesis, telomere maintenance via telomerase |
| Afu7g05550 | Ortholog(s) have dicarboxylic acid transmembrane transporter activity, role in dicarboxylic acid transport and plasma membrane localization |
| Afu7g05580 | Putative phospholipase D |
| Afu7g05590 | Ortholog(s) have cytosol, nucleus localization |
| Afu7g05710 | Ortholog of Aspergillus fumigatus A1163 : AFUB_091280 |
| Afu7g05720 | Pyruvate dehydrogenase complex, dihydrolipoamide acetyltransferase component |
| Afu7g05740 | Putative NAD-dependent malate dehydrogenase |
| Afu7g05780 | Ortholog(s) have role in establishment or maintenance of cell polarity, hyphal growth |
| Afu7g05940 | Ortholog of A. nidulans FGSC A4 : AN10851, A. niger CBS 513.88 : An07g01650, A. oryzae RIB40 : AO090005000458, Aspergillus wentii : Aspwe1_0291638 and Aspergillus sydowii : Aspsy1_0159486 |
| Afu7g05950 | Ortholog(s) have calcium ion binding activity and role in mitotic actomyosin contractile ring assembly actin filament organization, protein localization to medial cortex, regulation of cell cycle, spore germination |
| Afu7g05970 | Ortholog(s) have cytosol, nuclear envelope localization |
| Afu7g05980 | Ortholog(s) have role in mRNA splicing, via spliceosome and U1 snRNP, U2 snRNP, U2-type prespliceosome, U4/U6 x U5 tri-snRNP complex, U5 snRNP, cytosol localization |
| Afu7g06060 | Putative siderophore transporter |
| Afu7g06090 | Putative fatty-acyl coA oxidase with a predicted role in beta oxidation of fatty acids |
| Afu7g06100 | Putative acyl-coA dehydrogenase family protein with a predicted role in beta oxidation of fatty acids |
| Afu7g06110 | Has domain(s) with predicted hydrolase activity, hydrolyzing O-glycosyl compounds activity and role in carbohydrate metabolic process |
| Afu7g06140 | Putative secreted 1,4-beta-D-glucan glucanhydrolase |
| Afu7g06150 | Putative secreted cellulase |
| Afu7g06210 | Ortholog of Aspergillus fumigatus A1163 : AFUB_091780 |
| Afu7g06270 | Has domain(s) with predicted catalytic activity, metal ion binding, phosphoric diester hydrolase activity |
| Afu7g06290 | Has domain(s) with predicted catalytic activity and role in nucleoside metabolic process |
| Afu7g06410 | Ortholog(s) have hydrolase activity, acting on glycosyl bonds activity and role in carbohydrate catabolic process |
| Afu7g06480 | Ortholog of Neosartorya fischeri NRRL 181 : NFIA_027760 and Aspergillus fumigatus A1163 : AFUB_092040 |
| Afu7g06490 | Has domain(s) with predicted catalytic activity, deoxyribose-phosphate aldolase activity, lyase activity, role in deoxyribonucleotide catabolic process and cytoplasm localization |
| Afu7g06580 | Has domain(s) with predicted UDP-N-acetylmuramate dehydrogenase activity, flavin adenine dinucleotide binding, oxidoreductase activity and role in oxidation-reduction process |
| Afu7g06590 | Ortholog of A. nidulans FGSC A4 : AN7942, AN7872, AN5510, AN3420, AN2700, AN9050, AN8973, AN11222, AN10911, AN1749 and A. fumigatus Af293 : Afu1g12450, Afu2g00880, Afu2g17800, Afu2g17880, Afu5g14720 |
| Afu7g06950 | Ortholog of A. nidulans FGSC A4 : AN9032, AN10883, A. fumigatus Af293 : Afu8g02470, A. niger CBS 513.88 : An03g06330, An03g00350, An05g02580 and A. oryzae RIB40 : AO090010000660 |
| Afu7g07050 | protein of unknown function |
| Afu7g07080 | Predicted LINE transposon-related ORF |
| Afu7g07100 | Has domain(s) with predicted catalytic activity and role in nucleoside metabolic process |
| Afu7g07110 | Predicted mariner Ant1 transposon-related ORF |
| Afu7g08220 | Ortholog of Aspergillus fumigatus A1163 : AFUB_080330, AFUB_092580 |
| Afu7g08235 | protein of unknown function |
| Afu7g08260 | protein of unknown function |
| Afu7g08270 | protein of unknown function |
| Afu7g08300 | protein of unknown function |
| Afu7g08430 | protein of unknown function |
| Afu7g08480 | Has domain(s) with predicted ATP binding, DNA binding, helicase activity, nucleic acid binding, zinc ion binding activity |
| Afu7g08500 | Ortholog of A. oryzae RIB40 : AO090010000783, Aspergillus glaucus : Aspgl1_0068939, Aspgl1_0124292, Aspergillus flavus NRRL 3357 : AFL2T_00932 and Aspergillus kawachii : Aspka1_0182640 |
| Afu7g08570 | Ortholog of Neosartorya fischeri NRRL 181 : NFIA_032740, Aspergillus versicolor : Aspve1_0033447, Aspergillus niger ATCC 1015 : 45038-mRNA and Aspergillus sydowii : Aspsy1_0034803 |
| Afu7g08580 | Has domain(s) with predicted GTP binding, GTPase activity |
| Afu7g08600 | protein of unknown function |
| Afu7g08650 | Has domain(s) with predicted ATP binding activity |
| Afu8g00230 | Non-heme Fe(II) and alpha-ketoglutarate-dependent dioxygenase |
| Afu8g00290 | Ortholog of Aspergillus fumigatus A1163 : AFUB_086250 and Aspergillus niger ATCC 1015 : 178253-mRNA |
| Afu8g00400 | protein of unknown function |
| Afu8g00430 | Ortholog of A. nidulans AN1088 |
| Afu8g00530 | Putative alpha/beta hydrolase |
| Afu8g00595 | Has domain(s) with predicted hydrolase activity |
| Afu8g00660 | protein of unknown function |
| Afu8g00670 | Ortholog of A. niger CBS 513.88 : An03g02850, A. oryzae RIB40 : AO090010000618, Neosartorya fischeri NRRL 181 : NFIA_094110 and Aspergillus clavatus NRRL 1 : ACLA_044870 |
| Afu8g00750 | Has domain(s) with predicted DNA binding, RNA polymerase II transcription factor activity, sequence-specific DNA binding, nucleic acid binding, zinc ion binding activity |
| Afu8g00760 | Has domain(s) with predicted electron carrier activity, heme binding, iron ion binding, oxidoreductase activity, acting on paired donors, with incorporation or reduction of molecular oxygen activity and role in oxidation-reduction process |
| Afu8g00770 | Ortholog(s) have secondary active monocarboxylate transmembrane transporter activity, selenite:proton symporter activity and role in plasma membrane lactate transport, plasma membrane pyruvate transport, plasma membrane selenite transport |
| Afu8g00790 | Ortholog of A. oryzae RIB40 : AO090026000110 and Neosartorya fischeri NRRL 181 : NFIA_094250 |
| Afu8g00930 | Ortholog of A. fumigatus Af293 : Afu4g01290/csn, A. niger CBS 513.88 : An04g04530, A. oryzae RIB40 : AO090011000027, AO090020000697/csnA and Neosartorya fischeri NRRL 181 : NFIA_043990, NFIA_094360 |
| Afu8g00940 | Ortholog(s) have role in arginine transport, asperfuranone biosynthetic process and plasma membrane localization |
| Afu8g01030 | Ortholog of A. niger CBS 513.88 : An08g01250, An08g09900, Neosartorya fischeri NRRL 181 : NFIA_094470 and Aspergillus wentii : Aspwe1_0039768 |
| Afu8g01050 | Alkaline lipase with optimal activity at pH 8.5 |
| Afu8g01110 | Ortholog of Neosartorya fischeri NRRL 181 : NFIA_094550 and Aspergillus fumigatus A1163 : AFUB_085490 |
| Afu8g01190 | Has domain(s) with predicted UDP-N-acetylmuramate dehydrogenase activity, flavin adenine dinucleotide binding, oxidoreductase activity and role in oxidation-reduction process |
| Afu8g01240 | Ortholog(s) have role in kojic acid biosynthetic process, positive regulation of kojic acid biosynthetic process, positive regulation of secondary metabolite biosynthetic process, secondary metabolite biosynthetic process |
| Afu8g01340 | Major facilitator superfamily (MFS) sugar transporter |
| Afu8g01380 | Has domain(s) with predicted ADP binding activity |
| Afu8g01410 | Putative chitinase with a predicted role in the degradation and recycling of fungal chitin during autolysis |
| Afu8g01420 | Has domain(s) with predicted oxidoreductase activity, transferase activity, transferring acyl groups other than amino-acyl groups activity and role in oxidation-reduction process |
| Afu8g01460 | Ortholog of Aspergillus fumigatus A1163 : AFUB_085150 |
| Afu8g01465 | Has domain(s) with predicted RNA polymerase II transcription factor activity, sequence-specific DNA binding, zinc ion binding activity, role in regulation of transcription, DNA-templated and nucleus localization |
| Afu8g01485 | protein of unknown function |
| Afu8g01585 | rRNA |
| Afu8g01638 | Has domain(s) with predicted carbon-sulfur lyase activity and role in metabolic process |
| Afu8g01660 | Ortholog of Aspergillus fumigatus A1163 : AFUB_084940 |
| Afu8g01810 | Ortholog(s) have extracellular region localization |
| Afu8g01850 | Has domain(s) with predicted inorganic phosphate transmembrane transporter activity, role in phosphate ion transport and membrane localization |
| Afu8g01920 | Ortholog of A. nidulans FGSC A4 : AN12392, Neosartorya fischeri NRRL 181 : NFIA_095530, Aspergillus wentii : Aspwe1_0177490 and Aspergillus versicolor : Aspve1_0034524 |
| Afu8g01980 | Ortholog of A. nidulans FGSC A4 : AN7888, A. fumigatus Af293 : Afu4g08450, A. niger CBS 513.88 : An15g05230, A. oryzae RIB40 : AO090023000157 and Aspergillus wentii : Aspwe1_0627621, Aspwe1_0672214 |
| Afu8g02255 | rRNA |
| Afu8g02290 | Ortholog of A. nidulans FGSC A4 : AN2506, A. oryzae RIB40 : AO090026000736, Aspergillus wentii : Aspwe1_0055458, Aspwe1_0066430 and Aspergillus versicolor : Aspve1_0031627, Aspve1_0120546 |
| Afu8g02310 | Has domain(s) with predicted oxidoreductase activity, oxidoreductase activity, acting on the aldehyde or oxo group of donors, NAD or NADP as acceptor activity and role in oxidation-reduction process |
| Afu8g02315 | Ortholog(s) have endoplasmic reticulum localization |
| Afu8g02360 | Ortholog of Aspergillus fumigatus A1163 : AFUB_084230 |
| Afu8g02380 | Ortholog(s) have role in austinol biosynthetic process, dehydroaustinol biosynthetic process |
| Afu8g02390 | Ortholog of Neosartorya fischeri NRRL 181 : NFIA_096060 and Aspergillus fumigatus A1163 : AFUB_084210 |
| Afu8g02450 | Ortholog of A. nidulans FGSC A4 : AN8913, A. oryzae RIB40 : AO090010000666, Neosartorya fischeri NRRL 181 : NFIA_096150, Aspergillus wentii : Aspwe1_0177732 and Aspergillus versicolor : Aspve1_0396039 |
| Afu8g02460 | Has domain(s) with predicted role in transmembrane transport and integral component of membrane localization |
| Afu8g02470 | Ortholog of A. nidulans FGSC A4 : AN9032, AN10883, A. fumigatus Af293 : Afu7g06950, A. niger CBS 513.88 : An03g06330, An03g00350, An05g02580 and A. oryzae RIB40 : AO090010000660 |
| Afu8g02500 | Predicted glutathione S transferase |
| Afu8g02510 | Has domain(s) with predicted hydrolase activity, hydrolyzing O-glycosyl compounds activity and role in carbohydrate metabolic process |
| Afu8g02640 | Has domain(s) with predicted DNA binding, RNA polymerase II transcription factor activity, sequence-specific DNA binding, zinc ion binding activity and role in regulation of transcription, DNA-templated, transcription, DNA-templated |
| Afu8g02720 | Ortholog of A. niger CBS 513.88 : An03g06820, A. oryzae RIB40 : AO090010000763, Aspergillus wentii : Aspwe1_0177783, Aspergillus sydowii : Aspsy1_0050811 and Aspergillus terreus NIH2624 : ATET_10385 |
| Afu8g02750 | Nucleolar rRNA processing protein |
| Afu8g02770 | Ortholog(s) have cytidine deaminase activity, role in cytidine catabolic process, deoxycytidine catabolic process, pyrimidine-containing compound salvage and cytosol, nucleus localization |
| Afu8g02840 | Putative mitochondrial dynamin GTPase with a predicted role in mitochondrial fission |
| Afu8g03910 | Ortholog of A. nidulans FGSC A4 : AN9423, A. niger CBS 513.88 : An16g09290, Neosartorya fischeri NRRL 181 : NFIA_096620, Aspergillus wentii : Aspwe1_0053600 and Aspergillus versicolor : Aspve1_0422328 |
| Afu8g03950 | Ortholog of A. nidulans FGSC A4 : AN1405, A. niger CBS 513.88 : An16g09240, A. oryzae RIB40 : AO090103000008, Aspergillus wentii : Aspwe1_0043787 and Aspergillus sydowii : Aspsy1_0041996 |
| Afu8g03960 | Has domain(s) with predicted role in anaphase-promoting complex-dependent proteasomal ubiquitin-dependent protein catabolic process, regulation of mitotic metaphase/anaphase transition and anaphase-promoting complex localization |
| Afu8g04050 | Putative DNA binding transcription factor |
| Afu8g04060 | Has domain(s) with predicted hydrolase activity, hydrolyzing O-glycosyl compounds activity and role in carbohydrate metabolic process |
| Afu8g04110 | Putative DUF895 domain membrane protein |
| Afu8g04150 | Has domain(s) with predicted transporter activity, role in transmembrane transport and integral component of membrane, plasma membrane localization |
| Afu8g04190 | Ortholog of A. nidulans FGSC A4 : AN1431, A. niger CBS 513.88 : An16g08890, A. oryzae RIB40 : AO090103000034, Aspergillus wentii : Aspwe1_0043828 and Aspergillus sydowii : Aspsy1_0055863 |
| Afu8g04210 | Ortholog of A. nidulans FGSC A4 : AN1433, A. fumigatus Af293 : Afu1g01310, A. niger CBS 513.88 : An16g08870, An08g11860, An13g01880 and A. oryzae RIB40 : AO090701000542, AO090103000036 |
| Afu8g04350 | Ortholog(s) have RNA polymerase III activity, role in transcription initiation from RNA polymerase III type 2 promoter and DNA-directed RNA polymerase III complex, cytosol, nuclear chromatin localization |
| Afu8g04430 | Oligosaccharyl transferase subunit with a predicted role in N-linked protein glycosylation |
| Afu8g04490 | Transcript up-regulated in conidia exposed to neutrophils |
| Afu8g04660 | Ortholog(s) have microtubule plus-end binding activity and role in activation of bipolar cell growth, ascospore formation, cellular protein localization, microtubule polymerization or depolymerization, regulation of filamentous growth |
| Afu8g04670 | Ortholog(s) have role in ascospore formation, conidiophore development, conidium formation, regulation of vacuole fusion, non-autophagic and response to oxidative stress, more |
| Afu8g04702 | Ortholog(s) have cell cortex localization |
| Afu8g04730 | Oligopeptidase family protein |
| Afu8g04740 | Ortholog(s) have role in spliceosomal conformational changes to generate catalytic conformation and U5 snRNP, cytosol, spliceosomal complex localization |
| Afu8g04750 | Ortholog(s) have mitochondrion localization |
| Afu8g04810 | Ortholog(s) have protein kinase activity |
| Afu8g04820 | Ribonuclease P complex subunit |
| Afu8g04960 | Ortholog(s) have role in mRNA splicing, via spliceosome and U1 snRNP, U2 snRNP, U2-type prespliceosome, U4/U6 x U5 tri-snRNP complex, U5 snRNP, cytosol localization |
| Afu8g04980 | Ortholog of Aspergillus fumigatus A1163 : AFUB_082520 |
| Afu8g05060 | Ortholog of A. niger CBS 513.88 : An16g07690, A. oryzae RIB40 : AO090005000637, Neosartorya fischeri NRRL 181 : NFIA_097860 and Aspergillus wentii : Aspwe1_0053757 |
| Afu8g05080 | Ortholog of Aspergillus fumigatus A1163 : AFUB_082420 |
| Afu8g05090 | Has domain(s) with predicted role in transmembrane transport and integral component of membrane localization |
| Afu8g05130 | Ortholog of Aspergillus fumigatus A1163 : AFUB_082370 |
| Afu8g05330 | Putative methylenetetrahydrofolate dehydrogenase |
| Afu8g05360 | Ortholog(s) have cytosol, nucleus localization |
| Afu8g05370 | Ortholog(s) have role in regulation of transcription from RNA polymerase II promoter, transcription from RNA polymerase II promoter and cytosol, nucleus localization |
| Afu8g05410 | Putative glycosylphosphatidylinositol (GPI)-linked protein |
| Afu8g05420 | protein of unknown function |
| Afu8g05600 | Ortholog of A. nidulans FGSC A4 : AN1549, A. niger CBS 513.88 : An16g07080, A. oryzae RIB40 : AO090005000583, Aspergillus wentii : Aspwe1_0187131 and Aspergillus sydowii : Aspsy1_0086712 |
| Afu8g05670 | Ortholog(s) have cytoplasm localization |
| Afu8g05680 | Ortholog(s) have role in G2/M transition of mitotic cell cycle and asexual sporulation resulting in formation of a cellular spore, more |
| Afu8g05810 | DUF1295 domain protein |
| Afu8g05820 | Has domain(s) with predicted ATP binding, mismatched DNA binding activity and role in mismatch repair |
| Afu8g05850 | Has domain(s) with predicted oxidoreductase activity and role in oxidation-reduction process |
| Afu8g05870 | Ortholog of A. nidulans FGSC A4 : AN7918, A. niger CBS 513.88 : An15g04360, An09g00420, An16g04220, A. oryzae RIB40 : AO090003001435 and Aspergillus wentii : Aspwe1_0027288, Aspwe1_0048023 |
| Afu8g05950 | Ortholog of A. nidulans FGSC A4 : AN1574, AN0682, A. fumigatus Af293 : Afu1g13430, A. niger CBS 513.88 : An09g02840 and A. oryzae RIB40 : AO090012000536 |
| Afu8g05970 | Ortholog of A. nidulans FGSC A4 : AN5283, AN2708, AN1572, AN7419, A. fumigatus Af293 : Afu6g14000, A. niger CBS 513.88 : An07g02380, An08g07270 and A. oryzae RIB40 : AO090026000716, AO090005000543 |
| Afu8g06090 | Ortholog of A. nidulans FGSC A4 : AN8726, A. fumigatus Af293 : Afu7g00440, Afu8g06580 and A. niger CBS 513.88 : An02g08340, An02g09540, An03g00430 |
| Afu8g06100 | Ortholog of A. nidulans FGSC A4 : AN3303, AN3346, AN3348, AN2044, AN8727 and A. fumigatus Af293 : Afu4g10080, Afu7g00420, Afu4g01242 |
| Afu8g06132 | Ortholog of Aspergillus brasiliensis : Aspbr1_0060310, Aspergillus glaucus : Aspgl1_1496032, Neosartorya fischeri NRRL 181 : NFIA_069850 and Aspergillus fumigatus A1163 : AFUB_001950 |
| Afu8g06240 | Ortholog of Aspergillus fumigatus A1163 : AFUB_078880 |
| Afu8g06250 | Ortholog of Aspergillus fumigatus A1163 : AFUB_078890 |
| Afu8g06300 | Ortholog of Aspergillus fumigatus A1163 : AFUB_078930 |
| Afu8g06310 | Has domain(s) with predicted zinc ion binding activity and role in lipid metabolic process |
| Afu8g06370 | Has domain(s) with predicted FAD binding, oxidoreductase activity and role in metabolic process |
| Afu8g06480 | Has domain(s) with predicted hydrolase activity |
| Afu8g06520 | Has domain(s) with predicted protein-arginine deiminase activity and role in putrescine biosynthetic process |
| Afu8g06680 | Ortholog of Aspergillus fumigatus A1163 : AFUB_081100 and Aspergillus terreus NIH2624 : ATET_08599 |
| Afu8g06700 | Putative annexin |
| Afu8g06710 | Ortholog of Aspergillus fumigatus A1163 : AFUB_081070 |
| Afu8g06780 | Ortholog of Aspergillus fumigatus A1163 : AFUB_081020 |
| Afu8g06785 | Ortholog of Aspergillus clavatus NRRL 1 : ACLA_043880 |
| Afu8g06790 | protein of unknown function |
| Afu8g06960 | Ortholog of A. nidulans FGSC A4 : AN2397, Aspergillus flavus NRRL 3357 : AFL2T_05473, Neosartorya fischeri NRRL 181 : NFIA_099650 and Aspergillus versicolor : Aspve1_0026050 |
| Afu8g07140 | Aconitase family protein |
| Afu8g07235 | Ortholog of A. niger CBS 513.88 : An01g01475, A. oryzae RIB40 : AO090023000051, Neosartorya fischeri NRRL 181 : NFIA_100080, Aspergillus wentii : Aspwe1_0170039 and Aspergillus versicolor : Aspve1_0045698 |
| Afu8g07240 | Ortholog(s) have role in maltose metabolic process |
| Afu8g07260 | Has domain(s) with predicted transferase activity, transferring glycosyl groups activity and membrane localization |
| Afu8g07300 | Ortholog(s) have mitochondrion localization |

***A. flavus***

| Gene ID | Annotation |
| --- | --- |
| AFLA_000530 | conserved hypothetical protein |
| AFLA_000680 | hypothetical protein |
| AFLA_000700 | hypothetical protein |
| AFLA_000980 | conserved hypothetical protein |
| AFLA_001020 | cytochrome P450 monooxygenase, putative |
| AFLA_001030 | lanosterol synthase, putative |
| AFLA_001060 | conserved hypothetical protein |
| AFLA_001080 | glycosyltransferase, putative |
| AFLA_001090 | conserved hypothetical protein |
| AFLA_001100 | conserved hypothetical protein |
| AFLA_001260 | polyamine transporter, putative |
| AFLA_001270 | hypothetical protein |
| AFLA_001280 | isoflavone reductase family protein CipA, putative |
| AFLA_001310 | hypothetical protein |
| AFLA_001430 | conserved hypothetical protein |
| AFLA_001440 | feruloyl esterase B precursor, putative |
| AFLA_001490 | conserved hypothetical protein |
| AFLA_001500 | integral membrane protein |
| AFLA_001570 | conserved hypothetical protein |
| AFLA_001840 | conserved hypothetical protein |
| AFLA_001860 | conserved hypothetical protein |
| AFLA_001870 | MFS transporter, putative |
| AFLA_001900 | sulfatase domain protein |
| AFLA_001910 | hypothetical protein |
| AFLA_001920 | flavin-binding monooxygenase, putative |
| AFLA_001940 | amino acid transporter, putative |
| AFLA_001980 | glutamate cysteine ligase, putative |
| AFLA_002120 | conserved hypothetical protein |
| AFLA_002140 | acetyltransferase, putative |
| AFLA_002240 | hypothetical protein |
| AFLA_002340 | protein kinase regulator Ste50 |
| AFLA_002380 | conserved hypothetical protein |
| AFLA_002510 | actin monomer binding protein, putative |
| AFLA_002530 | nuclear protein SDK3, putative |
| AFLA_002730 | pre-rRNA-processing protein esf2 |
| AFLA_002750 | peroxisome biosynthesis protein (PAS10/Peroxin-12), putative |
| AFLA_002800 | conserved hypothetical protein |
| AFLA_003120 | conserved hypothetical protein |
| AFLA_003210 | conserved hypothetical protein |
| AFLA_003340 | conserved hypothetical protein |
| AFLA_003370 | hypothetical protein |
| AFLA_003380 | conserved hypothetical protein |
| AFLA_003480 | 60S ribosomal protein L32 |
| AFLA_003530 | dihydroxy acid dehydratase, putative |
| AFLA_003550 | glutamyl-tRNA synthetase |
| AFLA_003720 | sugar transporter, putative |
| AFLA_003790 | conserved hypothetical protein |
| AFLA_003960 | hypothetical protein |
| AFLA_003970 | conserved hypothetical protein |
| AFLA_004020 | conserved hypothetical protein |
| AFLA_004030 | hypothetical protein |
| AFLA_004040 | actin interacting protein 2 |
| AFLA_004070 | Mitochondrial import inner membrane translocase subunit TIM22, putative |
| AFLA_004100 | transesterase (LovD), putative |
| AFLA_004410 | conserved hypothetical protein |
| AFLA_004440 | ABC multidrug transporter, putative |
| AFLA_004450 | nonribosomal peptide synthase, putative |
| AFLA_004510 | peroxidase, putative |
| AFLA_004590 | catabolite degradation protein, putative |
| AFLA_004660 | glycogen synthase Gsy1, putative |
| AFLA_004720 | conserved hypothetical protein |
| AFLA_004810 | nuclear pore glycoprotein p62, putative |
| AFLA_004850 | hypothetical protein |
| AFLA_004920 | phosphoribosylformylglycinamidine synthase |
| AFLA_005010 | glucokinase regulator family protein, putative |
| AFLA_005030 | conserved hypothetical protein |
| AFLA_005070 | alcohol dehydrogenase, putative |
| AFLA_005080 | conserved hypothetical protein |
| AFLA_005090 | conserved hypothetical protein |
| AFLA_005130 | L-lysine 2,3-aminomutase, putative |
| AFLA_005150 | conserved hypothetical protein |
| AFLA_005200 | hypothetical protein |
| AFLA_005220 | hypothetical protein |
| AFLA_005270 | conserved hypothetical protein |
| AFLA_005400 | ankyrin repeat-containing protein, putative |
| AFLA_005420 | hypothetical protein |
| AFLA_005730 | 60S ribosomal protein L4, putative |
| AFLA_005740 | C6 transcription factor, putative |
| AFLA_005800 | WD repeat protein |
| AFLA_005820 | small nucleolar ribonucleoprotein complex subunit (Pwp2), putative |
| AFLA_005840 | conserved hypothetical protein |
| AFLA_006070 | TFIIH complex helicase Ssl2, putative |
| AFLA_006170 | polyketide synthetase PksP |
| AFLA_006230 | 1-aminocyclopropane-1-carboxylate deaminase, putative |
| AFLA_006240 | PHD transcription factor (Rum1), putative |
| AFLA_006250 | hypothetical protein |
| AFLA_006270 | tRNA-guanine transglycosylase family protein |
| AFLA_006320 | PQ loop repeat protein |
| AFLA_006350 | thioredoxin reductase, putative |
| AFLA_006380 | mitochondrial large ribosomal subunit protein L1, putative |
| AFLA_006520 | CRAL/TRIO domain protein |
| AFLA_006530 | conserved hypothetical protein |
| AFLA_006720 | bZIP transcription factor HapX |
| AFLA_006760 | integral membrane protein, putative |
| AFLA_006770 | proteasome maturation ans ribosome synthesis protein Nop10, putative |
| AFLA_006820 | conserved hypothetical protein |
| AFLA_006840 | ubiquitin conjugating enzyme (UbcH), putative |
| AFLA_006880 | PQ loop repeat protein |
| AFLA_006900 | cyclin-dependent protein kinase PhoA |
| AFLA_006990 | DNA directed RNA polymerase III subunit Rpc82, putative |
| AFLA_007000 | ubiquinol-cytochrome C reductase complex core protein 2, putative |
| AFLA_007070 | AMP-binding enzyme, putative |
| AFLA_007180 | ER to Golgi transport protein (Sly41), putative |
| AFLA_007250 | nucleolar RNAse III, putative |
| AFLA_007360 | hypothetical protein |
| AFLA_007390 | zeaxanthin epoxidase, putative |
| AFLA_007600 | oligopeptide transporter, putative |
| AFLA_007660 | conserved hypothetical protein |
| AFLA_007810 | gibberellin 20-oxidase family protein |
| AFLA_007820 | hypothetical protein |
| AFLA_007830 | conserved hypothetical protein |
| AFLA_007840 | cysteine desulfurylase, putative |
| AFLA_007930 | conserved hypothetical protein |
| AFLA_007990 | conserved hypothetical protein |
| AFLA_008120 | conserved hypothetical protein |
| AFLA_008140 | conserved hypothetical protein |
| AFLA_008150 | 5-oxoprolinase, putative |
| AFLA_008160 | conserved hypothetical protein |
| AFLA_008170 | conserved hypothetical protein |
| AFLA_008210 | NF-X1 finger and helicase domain protein, putative |
| AFLA_008220 | conserved hypothetical protein |
| AFLA_008230 | srpk, putative |
| AFLA_008250 | endosomal integral membrane protein (P24a), putative |
| AFLA_008260 | hypothetical protein |
| AFLA_008270 | U3 small nucleolar ribonucleoprotein protein LCP5, putative |
| AFLA_008280 | hypothetical protein |
| AFLA_008300 | ubiquitin conjugating enzyme (UbcB), putative |
| AFLA_008310 | acetyl-CoA-acetyltransferase, putative |
| AFLA_008330 | beta-1,4-mannosyltransferase (Alg1), putative |
| AFLA_008340 | CDF zinc transporter (Msc2), putative |
| AFLA_008380 | ubiquitin conjugating enzyme, putative |
| AFLA_008600 | conserved hypothetical protein |
| AFLA_008610 | hypothetical protein |
| AFLA_008620 | ankyrin repeat-containing protein, putative |
| AFLA_008910 | conserved hypothetical protein |
| AFLA_008960 | TPR domain protein |
| AFLA_008970 | SAM dependent methyltransferase, putative |
| AFLA_009040 | 3-isopropylmalate dehydrogenase |
| AFLA_009450 | hydroxymethylglutaryl-coenzyme A reductase family protein |
| AFLA_009570 | conserved hypothetical protein |
| AFLA_009690 | conserved hypothetical protein |
| AFLA_009740 | hypothetical protein |
| AFLA_009790 | conserved hypothetical protein |
| AFLA_009800 | conserved hypothetical protein |
| AFLA_009810 | hypothetical protein |
| AFLA_010020 | nonribosomal peptide synthase, putative |
| AFLA_010030 | oxidoreductase, 2OG-Fe(II) oxygenase family, putative |
| AFLA_010080 | ankyrin repeat-containing protein, putative |
| AFLA_010120 | extracellular dioxygenase, putative |
| AFLA_010170 | tropinone reductase, putative |
| AFLA_010200 | ankyrin, putative |
| AFLA_010310 | 2,4-dihydroxyhept-2-ene-1,7-dioic acid aldolase |
| AFLA_010480 | potassium channel, putative |
| AFLA_010490 | conserved hypothetical protein |
| AFLA_010500 | conserved hypothetical protein |
| AFLA_010680 | conserved hypothetical protein |
| AFLA_010700 | conserved hypothetical protein |
| AFLA_010710 | conserved hypothetical protein |
| AFLA_010720 | conserved hypothetical protein |
| AFLA_010950 | cytochrome P450 oxidoreductase, putative |
| AFLA_010990 | glucose-methanol-choline (gmc) oxidoreductase, putative |
| AFLA_011000 | conserved hypothetical protein |
| AFLA_011040 | conserved hypothetical protein |
| AFLA_011060 | conserved hypothetical protein |
| AFLA_011130 | conserved hypothetical protein |
| AFLA_011150 | allantoate permease, putative |
| AFLA_011170 | hypothetical protein |
| AFLA_011240 | conserved hypothetical protein |
| AFLA_011250 | conserved hypothetical protein |
| AFLA_011320 | conserved hypothetical protein |
| AFLA_011340 | hypothetical protein |
| AFLA_011350 | conserved hypothetical protein |
| AFLA_011460 | GMC oxidoreductase, putative |
| AFLA_011480 | amino acid permease (Can1), putative |
| AFLA_011530 | hypothetical protein |
| AFLA_011560 | phosphatidylglycerol specific phospholipase, putative |
| AFLA_011630 | transcriptional regulatory protein DEP1, putative |
| AFLA_011640 | RNA binding protein, putative |
| AFLA_011660 | conserved hypothetical protein |
| AFLA_011680 | conserved hypothetical protein |
| AFLA_011690 | 2-hydroxyacid dehydrogenase, putative |
| AFLA_011750 | conserved hypothetical protein |
| AFLA_011780 | hypothetical protein |
| AFLA_011900 | rRNA maturation protein (Nop14), putative |
| AFLA_011910 | triacylglycerol lipase, putative |
| AFLA_012020 | carboxypeptidase S1, putative |
| AFLA_012040 | hypothetical protein |
| AFLA_012050 | N-acetylglucosamine-6-phosphate deacetylase (NagA), putative |
| AFLA_012060 | choline oxidase (CodA), putative |
| AFLA_012090 | beta-N-acetylglucosaminidase, putative |
| AFLA_012100 | NDT80_PhoG domain protein PcaG |
| AFLA_012170 | U5 snRNP component Snu114, putative |
| AFLA_012190 | C6 finger domain protein, putative |
| AFLA_012390 | hypothetical protein |
| AFLA_012400 | oxidoreductase, short-chain dehydrogenase/reductase family |
| AFLA_012880 | pheromone response protein, putative |
| AFLA_012920 | conserved hypothetical protein |
| AFLA_012940 | sulfatase, putative |
| AFLA_013030 | conserved hypothetical protein |
| AFLA_013040 | conserved hypothetical protein |
| AFLA_013050 | conserved hypothetical protein |
| AFLA_013150 | conserved hypothetical protein |
| AFLA_013180 | williams-beuren syndrome chromosome region, putative |
| AFLA_013220 | beta-lactamase family protein |
| AFLA_013230 | steroid monooxygenase, putative |
| AFLA_013240 | C6 transcription factor, putative |
| AFLA_013270 | conserved hypothetical protein |
| AFLA_013320 | cellulase family protein |
| AFLA_013370 | sugar transporter, putative |
| AFLA_013390 | conserved hypothetical protein |
| AFLA_013490 | amino acid transporter, putative |
| AFLA_013530 | conserved hypothetical protein |
| AFLA_013590 | conserved hypothetical protein |
| AFLA_013810 | hypothetical protein |
| AFLA_013870 | 4-carboxymuconolactone decarboxylase family protein |
| AFLA_014000 | glutathione-S-transferase theta, GST, putative |
| AFLA_014020 | conserved hypothetical protein |
| AFLA_014090 | conserved hypothetical protein |
| AFLA_014110 | conserved hypothetical protein |
| AFLA_014280 | conserved hypothetical protein |
| AFLA_014290 | conserved hypothetical protein |
| AFLA_014360 | protein phosphatase 2A catalytic subunit Pph21, putative |
| AFLA_014400 | conserved hypothetical protein |
| AFLA_014470 | oxidoreductase, short-chain dehydrogenase/reductase family, putative |
| AFLA_014500 | plasma membrane channel protein (Aqy1), putative |
| AFLA_014700 | telomere-binding alpha subunit central domain protein |
| AFLA_014710 | karyopherin alpha subunit, putative |
| AFLA_014720 | nuclear cohesin complex subunit (Psc3), putative |
| AFLA_014730 | urease accessory protein UreD |
| AFLA_014810 | prolyl-tRNA synthetase |
| AFLA_014930 | cofactor for methionyl- and glutamyl-tRNA synthetase, putative |
| AFLA_014960 | mannan polymerase II complex ANP1 subunit Anp1, putative |
| AFLA_015070 | conserved hypothetical protein |
| AFLA_015080 | hypothetical protein |
| AFLA_015090 | HET-C domain protein |
| AFLA_015230 | poly(A)+ RNA transport protein (UbaA), putative |
| AFLA_015240 | gamma-tubulin complex component GCP4, putative |
| AFLA_015270 | MFS transporter, putative |
| AFLA_015290 | conserved hypothetical protein |
| AFLA_015360 | N-methyltransferase, putative |
| AFLA_015420 | sulfite reductase, putative |
| AFLA_015460 | molybdopterin biosynthesis protein, putative |
| AFLA_015530 | conserved hypothetical protein |
| AFLA_015610 | conserved hypothetical protein |
| AFLA_015660 | choline transport protein, putative |
| AFLA_015680 | conserved hypothetical protein |
| AFLA_015730 | DNA repair protein Pso2/Snm1, putative |
| AFLA_015930 | NAD dependent epimerase/dehydratase, putative |
| AFLA_016100 | glucose-methanol-choline (gmc) oxidoreductase, putative |
| AFLA_016110 | glucose dehydrogenase, putative |
| AFLA_016180 | hypothetical protein |
| AFLA_016200 | bypass of stop codon protein, putative |
| AFLA_016230 | cortactin-binding protein, putative |
| AFLA_016620 | tryptophanyl-tRNA synthetase, putative |
| AFLA_016640 | hypothetical protein |
| AFLA_016730 | alanine racemase |
| AFLA_016740 | conserved hypothetical protein |
| AFLA_016810 | Gamma-butyrobetaine hydroxylase subfamily, putative |
| AFLA_016830 | tubulin subunit TubB |
| AFLA_016930 | O-methyltransferase, putative |
| AFLA_017010 | mitochondrial processing peptidase beta subunit, putative |
| AFLA_017040 | C6 transcription factor, putative |
| AFLA_017050 | sugar transporter, putative |
| AFLA_017060 | conserved hypothetical protein |
| AFLA_017160 | integral membrane protein Pth11-like, putative |
| AFLA_017210 | C2H2 finger domain protein, putative |
| AFLA_017260 | solid-state culture specific ATP-grasp domain protein |
| AFLA_017530 | DNA-directed RNA polymerase I subunit (Rpa190), putative |
| AFLA_017580 | conserved hypothetical protein |
| AFLA_017600 | MFS multidrug transporter, putative |
| AFLA_017670 | hypothetical protein |
| AFLA_017680 | pentafunctional polypeptide (AroM), putative |
| AFLA_017770 | D-isomer specific 2-hydroxyacid dehydrogenase family protein |
| AFLA_017830 | small nuclear ribonucleoprotein complex protein Nhp2, putative |
| AFLA_017840 | NRPS-like enzyme, putative |
| AFLA_017910 | transketolase TktA |
| AFLA_017940 | hypothetical protein |
| AFLA_017950 | ubiquinol-cytochrome C reductase complex subunit UcrQ, putative |
| AFLA_017960 | prohibitin complex subunit Phb1, putative |
| AFLA_018200 | conserved hypothetical protein |
| AFLA_018230 | short-chain dehydrogenase, putative |
| AFLA_018240 | uridine permease Fui1, putative |
| AFLA_018250 | conserved hypothetical protein |
| AFLA_018300 | cell division control protein 14 |
| AFLA_018310 | geranylgeranyl diphosphate synthase |
| AFLA_018370 | nucleoporin (Nup184), putative |
| AFLA_018380 | anthranilate synthase multifunctional protein TrpC, putative |
| AFLA_018410 | C2H2 transcription factor, putative |
| AFLA_018460 | conserved hypothetical protein |
| AFLA_018470 | conserved hypothetical protein |
| AFLA_018830 | conserved hypothetical protein |
| AFLA_018860 | Exocyst complex component Sec5, putative |
| AFLA_018880 | conserved hypothetical protein |
| AFLA_018890 | factor for adipocyte differentiation, putative |
| AFLA_019000 | conserved hypothetical protein |
| AFLA_019050 | conserved hypothetical protein |
| AFLA_019060 | ABC transporter, putative |
| AFLA_019100 | F-box domain protein |
| AFLA_019110 | conserved hypothetical protein |
| AFLA_019120 | conserved hypothetical protein |
| AFLA_019130 | metacaspase CasB |
| AFLA_019580 | sugar transporter, putative |
| AFLA_019610 | carboxypeptidase S1, putative |
| AFLA_019710 | conserved hypothetical protein |
| AFLA_019740 | multidrug resistance protein 1, 2, putative |
| AFLA_019750 | hypothetical protein |
| AFLA_019920 | hypothetical protein |
| AFLA_020020 | conserved hypothetical protein |
| AFLA_020030 | conserved hypothetical protein |
| AFLA_020190 | hypothetical protein |
| AFLA_020250 | vacuolar segregation protein pep7, putative |
| AFLA_020300 | mitochondrial inner membrane protease subunit Imp2, putative |
| AFLA_020380 | stomatin family protein |
| AFLA_020400 | conserved hypothetical protein |
| AFLA_020470 | ribose-phosphate pyrophosphokinase |
| AFLA_020510 | 40S ribosomal protein S0 |
| AFLA_020520 | ribosomal protein S15, putative |
| AFLA_020550 | DEAD/DEAH box helicase (Sbp4), putative |
| AFLA_020840 | ubiquitin conjugating enzyme (UbcB), putative |
| AFLA_020880 | transcriptional corepressor of histone genes (Hir3), putative |
| AFLA_020960 | copper resistance-associated P-type ATPase, putative |
| AFLA_020970 | transcription factor TFIIIB complex subunit Brf1, putative |
| AFLA_020980 | conserved hypothetical protein |
| AFLA_020990 | DUF250 domain membrane protein |
| AFLA_021240 | HLH transcription factor (GlcD gamma), putative |
| AFLA_021350 | transulfuration enzyme family protein, putative |
| AFLA_021360 | transcriptional regulator, putative |
| AFLA_021420 | bypass of stop codon protein, putative |
| AFLA_021480 | actin-interacting protein (Bud6/Aip3), putative |
| AFLA_021660 | GPI anchored serine-threonine rich protein |
| AFLA_021740 | conserved hypothetical protein |
| AFLA_021780 | efflux pump antibiotic resistance protein, putative |
| AFLA_021820 | nuclear movement protein NudC |
| AFLA_021870 | cellobiohydrolase celD |
| AFLA_021880 | alpha/beta hydrolase family protein, putative |
| AFLA_021930 | C6 transcription factor RosA |
| AFLA_021940 | conserved hypothetical protein |
| AFLA_022050 | armadillo repeat protein |
| AFLA_022060 | cyclin-dependent protein kinase regulator Pho80 |
| AFLA_022130 | RING finger domain protein (Rnf10), putative |
| AFLA_022290 | hypothetical protein |
| AFLA_022310 | cytochrome P450, putative |
| AFLA_022320 | RNA12 protein, putative |
| AFLA_022480 | 26S proteasome regulatory particle subunit Rpn8, putative |
| AFLA_022530 | cell polarity protein (Alp11), putative |
| AFLA_022650 | MFS monosaccharide transporter, putative |
| AFLA_022660 | mitochondrial carrier protein, putative |
| AFLA_022670 | transcription elongation complex subunit (Cdc68) |
| AFLA_022690 | LEM3/CDC50 family protein |
| AFLA_022800 | conserved hypothetical protein |
| AFLA_022880 | oligopeptide transporter, putative |
| AFLA_022990 | conserved hypothetical protein |
| AFLA_023040 | C6 transcription factor, putative |
| AFLA_023080 | integral membrane protein TmpA |
| AFLA_023090 | conserved hypothetical protein |
| AFLA_023140 | conserved hypothetical protein |
| AFLA_023160 | ankyrin repeat-rich membrane-spanning protein, putative |
| AFLA_023170 | MFS transporter, putative |
| AFLA_023180 | GNAT family N-acetyltransferase, putative |
| AFLA_023210 | FAD dependent oxidoreductase, putative |
| AFLA_023500 | extracellular conserved serine-rich protein |
| AFLA_023510 | UDP-galactose 4-epimerase, putative |
| AFLA_023560 | periplasmic nitrate reductase, putative |
| AFLA_023610 | conserved hypothetical protein |
| AFLA_023630 | hypothetical protein |
| AFLA_023640 | conserved hypothetical protein |
| AFLA_023680 | conserved hypothetical protein |
| AFLA_023710 | cytochrome B5, putative |
| AFLA_023720 | phosphocarrier protein HPr, putative |
| AFLA_023740 | glutathione S-transferase, putative |
| AFLA_023840 | conserved hypothetical protein |
| AFLA_023910 | conserved hypothetical protein |
| AFLA_023930 | conserved hypothetical protein |
| AFLA_023970 | C6 transcription factor, putative |
| AFLA_024210 | cytochrome P450, putative |
| AFLA_024220 | conserved hypothetical protein |
| AFLA_024320 | conserved hypothetical protein |
| AFLA_024330 | phosphate transporter, putative |
| AFLA_024370 | MFS pantothenate transporter, putative |
| AFLA_024390 | pantothenate transporter, putative |
| AFLA_024410 | hypothetical protein |
| AFLA_024490 | IMP-specific 5'-nucleotidase (Isn1), putative |
| AFLA_024500 | conserved hypothetical protein |
| AFLA_024590 | plasma membrane zinc ion transporter, putative |
| AFLA_024600 | short chain oxidoreductase (CsgA), putative |
| AFLA_024610 | NADP-dependent alcohol dehydrogenase |
| AFLA_024630 | conserved hypothetical protein |
| AFLA_024680 | hypothetical protein |
| AFLA_024700 | short-chain alcohol dehydrogenase, putative |
| AFLA_024810 | MFS multidrug transporter, putative |
| AFLA_024890 | cell differentiation and development protein Fsr1/Pro11 |
| AFLA_024990 | conserved hypothetical protein |
| AFLA_025140 | dephospho-CoA kinase, putative |
| AFLA_025310 | hypothetical protein |
| AFLA_025330 | hypothetical protein |
| AFLA_025360 | epoxide hydrolase, putative |
| AFLA_025370 | conserved hypothetical protein |
| AFLA_025420 | conserved hypothetical protein |
| AFLA_025830 | histone acetyltransferase subunit (Yaf9), putative |
| AFLA_025910 | conserved hypothetical protein |
| AFLA_026080 | hypothetical protein |
| AFLA_026180 | conserved hypothetical protein |
| AFLA_026250 | homeobox transcription factor (RfeB), putative |
| AFLA_026390 | RING and UBP finger domain protein, putative |
| AFLA_026770 | alpha-1,6-mannosyltransferase subunit, putative |
| AFLA_026800 | conserved hypothetical protein |
| AFLA_026880 | nicotinamide riboside kinase, putative |
| AFLA_026970 | conserved hypothetical protein |
| AFLA_026980 | transporter protein smf2 |
| AFLA_027140 | ADAM family of metalloprotease ADM-B |
| AFLA_027240 | carbonic anhydrase Nce103, putative |
| AFLA_027350 | saccharopine dehydrogenase Lys9, putative |
| AFLA_027430 | Rho guanyl nucleotide exchange factor, putative |
| AFLA_027500 | mannose-1-phosphate guanylyltransferase |
| AFLA_027590 | conserved hypothetical protein |
| AFLA_027630 | conserved hypothetical protein |
| AFLA_027650 | PHD finger domain protein (Ing1), putative |
| AFLA_027700 | hypothetical protein |
| AFLA_027760 | conserved hypothetical protein |
| AFLA_027830 | ATP binding protein, putative |
| AFLA_027870 | hypothetical protein |
| AFLA_027890 | tubulin-specific chaperone, putative |
| AFLA_027910 | serine/threonine protein kinase, putative |
| AFLA_027940 | caffeine-induced death protein Cid2, putative |
| AFLA_028070 | conserved hypothetical protein |
| AFLA_028180 | RWD domain protein (Gir2), putative |
| AFLA_028190 | TPR domain protein |
| AFLA_028260 | exo-beta-1,3-glucanase (Exg1), putative |
| AFLA_028290 | conserved hypothetical protein |
| AFLA_028330 | hypothetical protein |
| AFLA_028340 | GPI anchored serine-threonine rich protein |
| AFLA_028400 | conserved hypothetical protein |
| AFLA_028430 | DUF726 domain protein |
| AFLA_028510 | oligosaccharyl transferase subunit Dad1, putative |
| AFLA_028760 | C2H2 transcription factor, putative |
| AFLA_028770 | conserved hypothetical protein |
| AFLA_028800 | Eukaryotic translation initiation factor eIF-5A |
| AFLA_028810 | hypothetical protein |
| AFLA_028920 | IQ and HECT domain protein |
| AFLA_029030 | Ser/Thr protein phosphatase family protein |
| AFLA_029130 | proline utilization protein PrnX-like, putative |
| AFLA_029160 | endoglucanase, putative |
| AFLA_029180 | PP-loop ATPase superfamily protein, putative |
| AFLA_029210 | HIT finger domain protein, putative |
| AFLA_029230 | hypothetical protein |
| AFLA_029330 | RING finger domain protein, putative |
| AFLA_029340 | transcriptional regulator Ngg1, putative |
| AFLA_029360 | conserved hypothetical protein |
| AFLA_029380 | urease Ure |
| AFLA_029520 | conserved hypothetical protein |
| AFLA_029530 | conserved hypothetical protein |
| AFLA_029570 | general stress response phosphoprotein phosphatase Psr1/2, putative |
| AFLA_029590 | conserved hypothetical protein |
| AFLA_029610 | hypothetical protein |
| AFLA_029640 | dynein light chain type 1, putative |
| AFLA_029660 | urea transporter (Dur3), putative |
| AFLA_029730 | calmodulin-binding protein Sha1, putative |
| AFLA_029770 | Leucine Rich Repeat domain protein |
| AFLA_029780 | Patatin-like serine hydrolase, putative |
| AFLA_029960 | Golgi complex component (Vps8), putative |
| AFLA_029970 | conserved hypothetical protein |
| AFLA_030330 | hypothetical protein |
| AFLA_030340 | RING finger domain protein, putative |
| AFLA_030350 | MFS sugar transporter, putative |
| AFLA_030360 | zinc binding dehydrogenase, putative |
| AFLA_030680 | microtubule associated protein EB1, putative |
| AFLA_030750 | hypothetical protein |
| AFLA_030760 | hypothetical protein |
| AFLA_030800 | conserved hypothetical protein |
| AFLA_030860 | RNAPII degradation factor Def1, putative |
| AFLA_030870 | conserved hypothetical protein |
| AFLA_030880 | conserved hypothetical protein |
| AFLA_030900 | ubiquitin C-terminal hydrolase, putative |
| AFLA_030970 | homoserine dehydrogenase |
| AFLA_031070 | Hsp40 co-chaperone Jid1, putative |
| AFLA_031150 | methylglutaconyl-CoA hydratase, mitochondrial precursor, putative |
| AFLA_031250 | aspartic endopeptidase Pep2 |
| AFLA_031270 | ARID/BRIGHT domain protein (SWI1), putative |
| AFLA_031280 | mitochondrial large ribosomal subunit L49, putative |
| AFLA_031460 | zinc metalloproteinase, putative |
| AFLA_031470 | hypothetical protein |
| AFLA_031870 | conserved hypothetical protein |
| AFLA_031880 | RTA1 domain protein, putative |
| AFLA_032040 | GNAT family acetyltransferase, putative |
| AFLA_032050 | glutamate carboxypeptidase Tre2, putative |
| AFLA_032120 | conserved hypothetical protein |
| AFLA_032140 | conserved hypothetical protein |
| AFLA_032150 | C6 finger domain protein, putative |
| AFLA_032160 | 3-oxoacyl-(acyl-carrier-protein) reductase, putative |
| AFLA_032280 | conserved hypothetical protein |
| AFLA_032330 | conserved hypothetical protein |
| AFLA_032350 | non-muscle caldesmon, putative |
| AFLA_032360 | hypothetical protein |
| AFLA_032490 | elongation of fatty acids protein, putative |
| AFLA_032650 | peroxisomal membrane anchor protein (Pex14), putative |
| AFLA_032660 | HEC/Ndc80p family protein |
| AFLA_032800 | conserved hypothetical protein |
| AFLA_032840 | forkhead domain protein |
| AFLA_032850 | hypothetical protein |
| AFLA_032890 | conserved hypothetical protein |
| AFLA_033020 | conserved hypothetical protein |
| AFLA_033150 | hypothetical protein |
| AFLA_033160 | C2H2 transcription factor (Sfp1), putative |
| AFLA_033280 | WD repeat protein |
| AFLA_033300 | hypothetical protein |
| AFLA_033310 | hypothetical protein |
| AFLA_033360 | transcription initiation factor TFIID, 31kD subunit, putative |
| AFLA_033650 | GTP-binding nuclear protein Ran, putative |
| AFLA_033660 | SWI/SNF family DNA-dependent ATPase Ris1, putative |
| AFLA_033700 | nitrilase family protein (Nit3), putative |
| AFLA_033710 | conserved hypothetical protein |
| AFLA_033720 | conserved hypothetical protein |
| AFLA_033760 | ubiquitin conjugating enzyme (UbcK), putative |
| AFLA_033770 | serine/threonine protein kinase, putative |
| AFLA_033800 | transcription initiation factor TFIIE, beta subunit, putative |
| AFLA_033840 | conserved hypothetical protein |
| AFLA_034040 | metallo-beta-lactamase domain protein, putative |
| AFLA_034110 | conserved hypothetical protein |
| AFLA_034340 | 40S ribosomal protein S10b |
| AFLA_034350 | conserved hypothetical protein |
| AFLA_034360 | conserved hypothetical protein |
| AFLA_034610 | C6 transcription factor, putative |
| AFLA_034620 | copper amine oxidase |
| AFLA_034880 | anaphase-promoting complex subunit ApcB, putative |
| AFLA_034890 | MFS monosaccharide transporter, putative |
| AFLA_034900 | hypothetical protein |
| AFLA_034910 | ER membrane protein (Pkr1), putative |
| AFLA_034990 | GTP binding protein, putative |
| AFLA_035200 | hypothetical protein |
| AFLA_035410 | conserved hypothetical protein |
| AFLA_035420 | hypothetical protein |
| AFLA_035460 | ketopantoate reductase family protein, putative |
| AFLA_035570 | conserved hypothetical protein |
| AFLA_035580 | alcohol dehydrogenase, putative |
| AFLA_035620 | Hsp70 chaperone BiP/Kar2, putative |
| AFLA_035650 | cytochrome P450, putative |
| AFLA_035660 | conserved hypothetical protein |
| AFLA_035680 | hydrolase, putative |
| AFLA_035960 | conserved hypothetical protein |
| AFLA_035990 | conserved hypothetical protein |
| AFLA_036000 | salicylate hydroxylase, putative |
| AFLA_036080 | fumarylacetoacetate hydrolase FahA |
| AFLA_036160 | hypothetical protein |
| AFLA_036170 | t-complex protein 1, epsilon subunit, putative |
| AFLA_036340 | mRNA cap methyltransferase |
| AFLA_036400 | conserved hypothetical protein |
| AFLA_036480 | chromatin remodeling complex subunit (Rsc9), putative |
| AFLA_036510 | methionine-R-sulfoxide reductase SelR, putative |
| AFLA_036530 | conserved hypothetical protein |
| AFLA_036570 | hypothetical protein |
| AFLA_036590 | mitochondrial GTPase (Miro-2), putative |
| AFLA_036600 | DUF500 and SH3 domain protein |
| AFLA_036640 | PH domain protein |
| AFLA_036670 | fungal specific transcription factor, putative |
| AFLA_036680 | fungal specific transcription factor, putative |
| AFLA_036830 | RNP domain protein |
| AFLA_036840 | 6-phosphogluconate dehydrogenase Gnd1, putative |
| AFLA_036850 | conserved hypothetical protein |
| AFLA_036920 | ankyrin repeat-containing protein, putative |
| AFLA_036960 | O-methyltransferase, putative |
| AFLA_036970 | pyroglutamyl peptidase type I, putative |
| AFLA_037020 | GINS DNA replication complex subunit Sld5, putative |
| AFLA_037060 | CwfJ domain protein |
| AFLA_037070 | conserved hypothetical protein |
| AFLA_037200 | conserved hypothetical protein |
| AFLA_037350 | casein kinase I, putative |
| AFLA_037540 | hypothetical protein |
| AFLA_037560 | conserved hypothetical protein |
| AFLA_037710 | phosphomannomutase (Sec53), putative |
| AFLA_037720 | mitochondrial protein FMP32, putative |
| AFLA_037750 | proteasome component (Ecm29), putative |
| AFLA_037800 | conserved hypothetical protein |
| AFLA_037850 | proteasome component Prs3, putative |
| AFLA_037860 | rRNA processing protein (Rrp20), putative |
| AFLA_037890 | conserved hypothetical protein |
| AFLA_037900 | methyltransferase, putative |
| AFLA_038250 | hypothetical protein |
| AFLA_038360 | conserved hypothetical protein |
| AFLA_038370 | alternative oxidase AlxA, putative |
| AFLA_038380 | allantoate permease, putative |
| AFLA_038440 | hypothetical protein |
| AFLA_038500 | conserved hypothetical protein |
| AFLA_038540 | ABC transporter, putative |
| AFLA_038580 | RTA1 domain protein |
| AFLA_038670 | MFS transporter, putative |
| AFLA_038680 | conserved hypothetical protein |
| AFLA_038700 | conserved hypothetical protein |
| AFLA_038780 | amino acid transporter, putative |
| AFLA_038830 | conserved hypothetical protein |
| AFLA_038840 | conserved hypothetical protein |
| AFLA_038850 | copper homeostasis protein, putative |
| AFLA_038970 | conserved hypothetical protein |
| AFLA_039020 | acetylcholinesterase, putative |
| AFLA_039070 | serine protein kinase Sky1, putative |
| AFLA_039100 | conserved hypothetical protein |
| AFLA_039130 | sensor histidine kinase/response regulator TcsB/Sln1, putative |
| AFLA_039180 | MFS gliotoxin efflux transporter GliA |
| AFLA_039190 | conserved hypothetical protein |
| AFLA_039460 | conserved hypothetical protein |
| AFLA_039500 | conserved hypothetical protein |
| AFLA_039570 | conserved hypothetical protein |
| AFLA_039580 | conserved hypothetical protein |
| AFLA_039600 | conserved hypothetical protein |
| AFLA_039700 | 2,4-dihydroxyhept-2-ene-1,7-dioic acid aldolase, putative |
| AFLA_039740 | conserved hypothetical protein |
| AFLA_039780 | PKS-like enzyme, putative |
| AFLA_039800 | integral membrane protein |
| AFLA_039900 | conserved hypothetical protein |
| AFLA_039920 | hypothetical protein |
| AFLA_039950 | ankyrin repeat-containing protein, putative |
| AFLA_040060 | TPR domain protein |
| AFLA_040120 | flavohemoprotein |
| AFLA_040140 | aquaporin |
| AFLA_040330 | chitin binding domain protein Peritrophin-A, putative |
| AFLA_040450 | amino acid permease family protein, putative |
| AFLA_040480 | hypothetical protein |
| AFLA_040520 | hypothetical protein |
| AFLA_040530 | bcs1 AAA-type ATPase, putative |
| AFLA_040580 | conserved hypothetical protein |
| AFLA_040690 | hypothetical protein |
| AFLA_040780 | hypothetical protein |
| AFLA_040830 | conserved hypothetical protein |
| AFLA_040880 | vegetative specific protein H5, putative |
| AFLA_040890 | dienelactone hydrolase, putative |
| AFLA_040910 | conserved hypothetical protein |
| AFLA_041010 | conserved hypothetical protein |
| AFLA_041030 | aldo-keto reductase, putative |
| AFLA_041110 | hypothetical protein |
| AFLA_041120 | hypothetical protein |
| AFLA_041160 | oxidoreductase, 2OG-Fe(II) oxygenase family |
| AFLA_041250 | mitochondrial complex I protein Fmp36, putative |
| AFLA_041350 | sulfonylurea receptor/ ABC transporter, putative |
| AFLA_041360 | hypothetical protein |
| AFLA_041460 | conserved hypothetical protein |
| AFLA_041470 | hypothetical protein |
| AFLA_041490 | conserved hypothetical protein |
| AFLA_041520 | chromosome segregation protein (Pcs1), putative |
| AFLA_041550 | cystathionine beta-lyase MetG |
| AFLA_041700 | ferric-chelate reductase, putative |
| AFLA_041740 | protein kinase Yak1, putative |
| AFLA_041750 | DNA repair protein Rhp26/Rad26, putative |
| AFLA_041790 | cytochrome P450 alkane hydroxylase, putative |
| AFLA_041800 | serine peptidase, family S28, putative |
| AFLA_041810 | hypothetical protein |
| AFLA_042130 | conserved hypothetical protein |
| AFLA_042260 | conserved hypothetical protein |
| AFLA_042370 | conserved hypothetical protein |
| AFLA_042660 | cytochrome P450, putative |
| AFLA_042670 | C2 and Extensin domain protein |
| AFLA_042690 | conserved hypothetical protein |
| AFLA_042740 | hypothetical protein |
| AFLA_042760 | AMP-binding enzyme, putative |
| AFLA_042980 | signal recognition particle 14kD protein, putative |
| AFLA_043040 | peptidyl-prolyl cis-trans isomerase Cpr7, putative |
| AFLA_043050 | aminoacyl-tRNA hydrolase, putative |
| AFLA_043060 | CCR4-NOT transcription complex, subunit 3 |
| AFLA_043070 | C2H2 finger domain protein, putative |
| AFLA_043120 | phosphoglucomutase, putative |
| AFLA_043150 | 40S ribosomal protein S10a |
| AFLA_043190 | conserved hypothetical protein |
| AFLA_043200 | hypothetical protein |
| AFLA_043360 | conserved hypothetical protein |
| AFLA_043370 | conserved hypothetical protein |
| AFLA_043390 | Hsp70 chaperone (BiP), putative |
| AFLA_043410 | MYB DNA-binding domain protein |
| AFLA_043470 | ankyrin repeat-containing protein, putative |
| AFLA_043570 | cytoplasm to vacuole targeting Vps64, putative |
| AFLA_043580 | hypothetical protein |
| AFLA_043590 | mitochondrial genome maintenance protein Mgr2, putative |
| AFLA_043830 | thiamin biosynthesis protein (Thi-4), putative |
| AFLA_043900 | conserved hypothetical protein |
| AFLA_043920 | alpha-tubulin suppressor protein Aats1, putative |
| AFLA_043940 | hypothetical protein |
| AFLA_043970 | enoyl-CoA hydratase |
| AFLA_044030 | conserved hypothetical protein |
| AFLA_044150 | conserved hypothetical protein |
| AFLA_044290 | pre-mRNA-splicing factor cwc25, putative |
| AFLA_044770 | mandelate racemase/muconate lactonizing enzyme family protein |
| AFLA_044950 | hypothetical protein |
| AFLA_045100 | conserved hypothetical protein |
| AFLA_045120 | conserved hypothetical protein |
| AFLA_045150 | conserved hypothetical protein |
| AFLA_045340 | universal stress protein family domain protein |
| AFLA_045350 | conserved hypothetical protein |
| AFLA_045390 | N-alkane-inducible cytochrome P450, putative |
| AFLA_045440 | D-amino acid oxidase, putative |
| AFLA_045570 | acetyl xylan esterase, putative |
| AFLA_045580 | hypothetical protein |
| AFLA_045650 | hypothetical protein |
| AFLA_045660 | multicopper oxidase/laccase, putative |
| AFLA_045760 | conserved hypothetical protein |
| AFLA_046000 | conserved hypothetical protein |
| AFLA_046010 | eukaryotic translation initiation factor 5, putative |
| AFLA_046030 | conserved hypothetical protein |
| AFLA_046210 | conserved hypothetical protein |
| AFLA_046260 | SET domain protein |
| AFLA_046270 | RING finger domain protein, putative |
| AFLA_046300 | integral membrane protein, putative |
| AFLA_046330 | conserved hypothetical protein |
| AFLA_046360 | acetyl-CoA carboxylase, putative |
| AFLA_046480 | DNA directed RNA polymerase II 15 kDa subunit, putative |
| AFLA_046510 | ATP-dependent RNA helicase Mrh4, putative |
| AFLA_046520 | GTP binding protein (Bud4), putative |
| AFLA_046660 | mitochondrial carrier protein, putative |
| AFLA_046680 | hypothetical protein |
| AFLA_046780 | hypothetical protein |
| AFLA_046980 | diphosphonucleoside phosphohydrolase, putative |
| AFLA_047040 | conserved hypothetical protein |
| AFLA_047200 | palmitoyltransferase SidR |
| AFLA_047410 | actin-bundling protein Sac6, putative |
| AFLA_047450 | gamma-glutamyl phosphate reductase |
| AFLA_047460 | COP9 subunit 3, putative |
| AFLA_048050 | nucleosome assembly protein Nap1, putative |
| AFLA_048070 | non-canonical purine NTP pyrophosphatase, rdgB/HAM1 family |
| AFLA_048230 | Golgi GDP-mannose transporter |
| AFLA_048240 | NTP binding protein, putative |
| AFLA_048370 | glycyl-tRNA synthetase |
| AFLA_048480 | SET and WW domain protein |
| AFLA_048560 | integral membrane protein (Ytp1), putative |
| AFLA_048630 | conserved hypothetical protein |
| AFLA_048640 | conserved hypothetical protein |
| AFLA_048720 | 5-aminolevulinic acid synthase HemA |
| AFLA_048810 | 60S ribosomal protein L8, putative |
| AFLA_048820 | conserved hypothetical protein |
| AFLA_048900 | ubiquitin C-terminal hydrolase family protein |
| AFLA_048930 | hypothetical protein |
| AFLA_048940 | conserved hypothetical protein |
| AFLA_048950 | cytochrome P450, putative |
| AFLA_049100 | aminotransferase, putative |
| AFLA_049290 | citrate synthase Cit1, putative |
| AFLA_049300 | mRNA-nucleus export ATPase (Elf1), putative |
| AFLA_049330 | conserved hypothetical protein |
| AFLA_049360 | O-methyltransferase, putative |
| AFLA_049370 | short-chain dehydrogenase/reductase family protein, putative |
| AFLA_049420 | beta-glucosidase, putative |
| AFLA_049520 | conserved hypothetical protein |
| AFLA_049560 | hypothetical protein |
| AFLA_049570 | allantoate permease, putative |
| AFLA_049630 | conserved hypothetical protein |
| AFLA_049640 | conserved hypothetical protein |
| AFLA_049650 | conserved hypothetical protein |
| AFLA_049670 | short-chain dehydrogenase, putative |
| AFLA_049690 | conserved hypothetical protein |
| AFLA_049730 | conserved hypothetical protein |
| AFLA_049770 | hypothetical protein |
| AFLA_049810 | conserved hypothetical protein |
| AFLA_049990 | AMP-binding domain protein, putative |
| AFLA_050350 | conserved hypothetical protein |
| AFLA_050390 | conserved hypothetical protein |
| AFLA_050400 | conserved hypothetical protein |
| AFLA_050450 | toxin biosynthesis protein, putative |
| AFLA_050470 | DNA repair protein Rtt107, putative |
| AFLA_050480 | ESCRT-II complex component (Vps25), putative |
| AFLA_050540 | conserved hypothetical protein |
| AFLA_050730 | transcription factor SipA3, putative |
| AFLA_050780 | dfg10 protein, putative |
| AFLA_050790 | histone deacetylase complex subunit (Hos4), putative |
| AFLA_050830 | uridine nucleosidase Urh1, putative |
| AFLA_051000 | nuclear-mitochondrial protein Rmp1, putative |
| AFLA_051020 | conserved hypothetical protein |
| AFLA_051050 | conserved hypothetical protein |
| AFLA_051100 | C6 transcription factor, putative |
| AFLA_051110 | conserved hypothetical protein |
| AFLA_051220 | conserved hypothetical protein |
| AFLA_051230 | protein transport protein Sec39, putative |
| AFLA_051350 | C6 transcription factor, putative |
| AFLA_051370 | phosphoribosyl-aminoimidazole carboxylase |
| AFLA_051380 | conserved hypothetical protein |
| AFLA_051450 | hypothetical protein |
| AFLA_051520 | conserved hypothetical protein |
| AFLA_051570 | 50S ribosomal protein L36, putative |
| AFLA_051600 | ubiquitination network signaling protein, putative |
| AFLA_051720 | ubiquinone biosynthesis monooxgenase (Coq6), putative |
| AFLA_051730 | conserved hypothetical protein |
| AFLA_051840 | tubulin beta subunit |
| AFLA_051910 | hypothetical protein |
| AFLA_051960 | DUF159 domain protein |
| AFLA_051990 | TPR repeat protein |
| AFLA_052030 | developmental regulatory protein WetA |
| AFLA_052100 | HAD superfamily hydrolase, putative |
| AFLA_052400 | isocitrate lyase AcuD |
| AFLA_052460 | PE repeat family protein |
| AFLA_052470 | gamma-glutamyltranspeptidase |
| AFLA_052480 | conserved hypothetical protein |
| AFLA_052510 | WSC domain protein, putative |
| AFLA_052520 | hypothetical protein |
| AFLA_052580 | conserved hypothetical protein |
| AFLA_052600 | conserved hypothetical protein |
| AFLA_052630 | NADH-ubiquinone oxidoreductase 213 kDa subunit |
| AFLA_052730 | muramidase, putative |
| AFLA_052800 | 1,3-beta-glucan synthase catalytic subunit FksP |
| AFLA_052810 | 1,3-beta-glucanosyltransferase, putative |
| AFLA_052820 | conserved hypothetical protein |
| AFLA_052840 | Ran-interacting protein Mog1, putative |
| AFLA_052850 | conserved hypothetical protein |
| AFLA_052860 | chaperone/heat shock protein Hsp12, putative |
| AFLA_052870 | conserved hypothetical protein |
| AFLA_053380 | hypothetical protein |
| AFLA_053550 | conserved hypothetical protein |
| AFLA_053560 | conserved hypothetical protein |
| AFLA_053610 | conserved hypothetical protein |
| AFLA_053620 | geranylgeranyl pyrophosphate synthase, putative |
| AFLA_053660 | nuclear transport factor 2 domain protein |
| AFLA_053750 | hypothetical protein |
| AFLA_053760 | hypothetical protein |
| AFLA_053770 | PKS-like enzyme, putative |
| AFLA_053820 | FAD dependent oxidoreductase, putative |
| AFLA_053870 | polyketide synthase, putative |
| AFLA_053880 | hypothetical protein |
| AFLA_053910 | pantothenate transporter, putative |
| AFLA_053920 | hypothetical protein |
| AFLA_053930 | multicopper oxidase, putative |
| AFLA_054000 | hypothetical protein |
| AFLA_054010 | siderochrome-iron transporter, putative |
| AFLA_054160 | hypothetical protein |
| AFLA_054180 | conserved hypothetical protein |
| AFLA_054220 | conserved hypothetical protein |
| AFLA_054230 | conserved hypothetical protein |
| AFLA_054530 | conserved hypothetical protein |
| AFLA_054540 | conserved hypothetical protein |
| AFLA_054660 | aspartic-type endopeptidase (OpsB), putative |
| AFLA_054680 | purine nucleoside phosphorylase I, inosine and guanosine-specific |
| AFLA_054740 | BET3 family protein |
| AFLA_054770 | conserved hypothetical protein |
| AFLA_054810 | transcriptional repressor TupA/RocA, putative |
| AFLA_055010 | homoaconitase LysF |
| AFLA_055050 | conserved hypothetical protein |
| AFLA_055080 | sucrase/ferredoxin-like family protein Fmi1, putative |
| AFLA_055260 | leucine rich repeat and nacht domain-containing protein, putative |
| AFLA_055290 | conserved hypothetical protein |
| AFLA_055330 | ER-associated proteolytic system protein Der1, putative |
| AFLA_055370 | conserved hypothetical protein |
| AFLA_055380 | alpha/beta hydrolase domain containing protein 1,3, putative |
| AFLA_055480 | calcineurin Ca2+-binding regulatory subunit CnaB |
| AFLA_055490 | histone acetyltransferase complex component Epl1, putative |
| AFLA_055610 | mitochondrial translation optimization protein (Mto1), putative |
| AFLA_055690 | ABC drug exporter AtrF |
| AFLA_055760 | mRNA binding protein Pumilio 2, putative |
| AFLA_055780 | phosphoethanolamine N-methyltransferase, putative |
| AFLA_055790 | GTPase activating protein (Tsc2), putative |
| AFLA_055800 | chromosome segregation protein Cse1, putative |
| AFLA_055830 | protein PET100, mitochondrial precursor, putative |
| AFLA_055840 | peroxisomal D3,D2-enoyl-CoA isomerase |
| AFLA_056180 | conserved hypothetical protein |
| AFLA_056200 | tetratricopeptide repeat protein |
| AFLA_056370 | high-affinity glucose transporter, putative |
| AFLA_056380 | conserved hypothetical protein |
| AFLA_056420 | hypothetical protein |
| AFLA_056440 | conserved hypothetical protein |
| AFLA_056470 | conserved hypothetical protein |
| AFLA_056530 | conserved hypothetical protein |
| AFLA_056540 | MFS multidrug transporter, putative |
| AFLA_056700 | DUF89 domain protein |
| AFLA_056730 | cell cycle control protein (Cwf2), putative |
| AFLA_056740 | GTP binding protein (Gtp1), putative |
| AFLA_056820 | geranylgeranyl pyrophosphate synthase, putative |
| AFLA_056890 | conserved hypothetical protein |
| AFLA_056950 | homoserine O-acetyltransferase, putative |
| AFLA_056960 | conserved hypothetical protein |
| AFLA_056990 | conserved hypothetical protein |
| AFLA_057130 | THO complex component (Rlr1), putative |
| AFLA_057190 | conserved hypothetical protein |
| AFLA_057200 | conserved hypothetical protein |
| AFLA_057300 | conserved hypothetical protein |
| AFLA_057330 | conserved hypothetical protein |
| AFLA_057530 | S-adenosylmethionine synthetase, putative |
| AFLA_057540 | neuroligin, putative |
| AFLA_057550 | conserved hypothetical protein |
| AFLA_057620 | CCR4-NOT core complex subunit Caf1, putative |
| AFLA_057630 | NADH-ubiquinone oxidoreductase B12 subunit, putative |
| AFLA_057640 | 60S ribosome biogenesis protein Sqt1, putative |
| AFLA_057710 | carboxyphosphonoenolpyruvate phosphonomutase, putative |
| AFLA_057730 | DUF1275 domain protein |
| AFLA_057820 | siderophore esterase IroE-like, putative |
| AFLA_057830 | ABC multidrug transporter, putative |
| AFLA_057980 | conserved hypothetical protein |
| AFLA_057990 | purine nucleoside permease, putative |
| AFLA_058140 | hypothetical protein |
| AFLA_058150 | myo-inositol-phosphate synthase, putative |
| AFLA_058410 | GPI anchored protein, putative |
| AFLA_058610 | HLH transcription factor, putative |
| AFLA_058620 | ferric-chelate reductase FREA |
| AFLA_058690 | sugar transporter, putative |
| AFLA_058700 | short-chain dehydrogenase, putative |
| AFLA_058710 | alcohol dehydrogenase, putative |
| AFLA_058730 | conserved hypothetical protein |
| AFLA_058790 | exosome complex subunit Rrp46, putative |
| AFLA_058810 | arginase, putative |
| AFLA_058820 | GABA permease, putative |
| AFLA_058870 | xenobiotic compound monooxygenase, DszA family |
| AFLA_059150 | conserved hypothetical protein |
| AFLA_059180 | short-chain dehydrogenase, putative |
| AFLA_059380 | integral membrane protein |
| AFLA_059590 | copper amine oxidase, putative |
| AFLA_059670 | conserved hypothetical protein |
| AFLA_059740 | conserved hypothetical protein |
| AFLA_059810 | conserved hypothetical protein |
| AFLA_060140 | carboxylesterase, putative |
| AFLA_060160 | oxidoreductase, putative |
| AFLA_060170 | conserved hypothetical protein |
| AFLA_060220 | conserved hypothetical protein |
| AFLA_060290 | isochorismatase family protein family |
| AFLA_060300 | Ketol-acid reductoisomerase |
| AFLA_060450 | O-methyltransferase, putative |
| AFLA_060550 | conserved hypothetical protein |
| AFLA_060560 | bromodomain associated domain protein |
| AFLA_060660 | AMP-binding enzyme, putative |
| AFLA_060680 | dimethylallyl tryptophan synthase, putative |
| AFLA_060700 | phenylalanine ammonia-lyase, putative |
| AFLA_060860 | conserved hypothetical protein |
| AFLA_060990 | conserved hypothetical protein |
| AFLA_061000 | hypothetical protein |
| AFLA_061020 | CAIB/BAIF family enzyme |
| AFLA_061110 | mono- and diacylglycerol lipase precursor, putative |
| AFLA_061140 | cytochrome P450, putative |
| AFLA_061150 | alcohol dehydrogenase, putative |
| AFLA_061180 | GTPase activating protein (Gyp2), putative |
| AFLA_061190 | run and tbc1 domain containing 3, plant, putative |
| AFLA_061200 | conserved hypothetical protein |
| AFLA_061280 | alcohol dehydrogenase, putative |
| AFLA_061310 | methylmalonyl-CoA decarboxylase, alpha subunit, putative |
| AFLA_061420 | nuclear migration protein (ApsA), putative |
| AFLA_061430 | CCCH and RING finger protein (Znf183), putative |
| AFLA_061590 | conserved hypothetical protein |
| AFLA_062220 | high osmolarity signaling protein Sho1, putative |
| AFLA_062230 | conserved hypothetical protein |
| AFLA_062330 | C6 transcription factor, putative |
| AFLA_062360 | FHA domain protein |
| AFLA_062430 | conserved hypothetical protein |
| AFLA_062540 | integral peroxisomal membrane peroxin, putative |
| AFLA_062570 | phosphatidylserine decarboxylase, putative |
| AFLA_062590 | endoplasmic reticulum mannosyl-oligosaccharide 1,2-alpha-mannosidase, putative |
| AFLA_062690 | alpha-1,6 mannosyltransferase subunit (Mnn9), putative |
| AFLA_062720 | conserved hypothetical protein |
| AFLA_062970 | copper/topaquinone oxidase, putative |
| AFLA_062990 | 1-aminocyclopropane-1-carboxylate oxidase, putative |
| AFLA_063060 | conserved hypothetical protein |
| AFLA_063070 | conserved hypothetical protein |
| AFLA_063110 | hypothetical protein |
| AFLA_063120 | isoamyl alcohol oxidase, putative |
| AFLA_063130 | conserved hypothetical protein |
| AFLA_063140 | hypothetical protein |
| AFLA_063210 | amino acid permease, putative |
| AFLA_063220 | conserved hypothetical protein |
| AFLA_063320 | conserved hypothetical protein |
| AFLA_063330 | protein phosphatase 1 regulatory inhibitor subunit 16A, putative |
| AFLA_063390 | conserved hypothetical protein |
| AFLA_063400 | hypothetical protein |
| AFLA_063410 | conserved hypothetical protein |
| AFLA_063550 | conserved hypothetical protein |
| AFLA_063730 | conserved hypothetical protein |
| AFLA_063740 | vitamin H transporter, putative |
| AFLA_064210 | hypothetical protein |
| AFLA_064290 | O-methyltransferase, putative |
| AFLA_064310 | hypothetical protein |
| AFLA_064320 | oligopeptide transporter, putative |
| AFLA_064480 | conserved hypothetical protein |
| AFLA_064500 | conserved hypothetical protein |
| AFLA_064510 | thioredoxin reductase GliT-like, putative |
| AFLA_064520 | O-methyltransferase GliM-like, putative |
| AFLA_064530 | glutathione S-transferase GliG-like, putative |
| AFLA_064700 | hypothetical protein |
| AFLA_064710 | pantothenate transporter, putative |
| AFLA_064780 | conserved hypothetical protein |
| AFLA_064790 | conserved hypothetical protein |
| AFLA_064800 | MFS monosaccharide transporter, putative |
| AFLA_064840 | conserved hypothetical protein |
| AFLA_064870 | NAD dependent epimerase/dehydratase, putative |
| AFLA_064880 | hypothetical protein |
| AFLA_064990 | conserved hypothetical protein |
| AFLA_065000 | phenylacrylic acid decarboxylase, putative |
| AFLA_065330 | malate dehydrogenase, putative |
| AFLA_065390 | 3-isopropymalate dehydrogenase, putative |
| AFLA_065400 | conserved hypothetical protein |
| AFLA_065410 | conserved hypothetical protein |
| AFLA_065590 | estradiol 17 beta-dehydrogenase, putative |
| AFLA_065870 | conserved hypothetical protein |
| AFLA_065930 | pantothenate transporter, putative |
| AFLA_066010 | hypothetical protein |
| AFLA_066100 | conserved hypothetical protein |
| AFLA_066490 | WD repeat protein |
| AFLA_066520 | hypothetical protein |
| AFLA_066550 | alcohol dehydrogenase, putative |
| AFLA_066640 | short chain dehydrogenase family protein |
| AFLA_066670 | conserved hypothetical protein |
| AFLA_066740 | MSF multidrug transporter, putative |
| AFLA_066760 | conserved hypothetical protein |
| AFLA_066780 | geranylgeranyl pyrophosphate synthase, putative |
| AFLA_066920 | alcohol dehydrogenase, putative |
| AFLA_067010 | carnitine acetyl transferase |
| AFLA_067060 | NADH-ubiquinone oxidoreductase 19 kDa subunit, putative |
| AFLA_067070 | nucleoporin, putative |
| AFLA_067190 | pla2g4b, putative |
| AFLA_067200 | ABC multidrug transporter, putative |
| AFLA_067210 | conserved hypothetical protein |
| AFLA_067330 | conserved hypothetical protein |
| AFLA_067360 | translation elongation factor EF-Tu, putative |
| AFLA_067380 | nucleoside diphosphatase Gda1 |
| AFLA_067420 | NADH-ubiquinone oxidoreductase 14 kDa subunit, putative |
| AFLA_067450 | glycine cleavage system H protein |
| AFLA_067460 | 37S ribosomal protein Mrp17 |
| AFLA_067470 | acyl-CoA thioesterase |
| AFLA_067580 | hypothetical protein |
| AFLA_067710 | protein kinase, putative |
| AFLA_067720 | conserved hypothetical protein |
| AFLA_067730 | SH3 domain protein, putative |
| AFLA_068130 | quinate 5-dehydrogenase QutB, putative |
| AFLA_068140 | MFS quinate transporter QutD |
| AFLA_068180 | ethanolamine kinase, putative |
| AFLA_068190 | CORD and CS domain protein |
| AFLA_068200 | conserved hypothetical protein |
| AFLA_068220 | conserved hypothetical protein |
| AFLA_068230 | hypothetical protein |
| AFLA_068280 | cop-coated vesicle membrane protein P24, putative |
| AFLA_068290 | conserved hypothetical protein |
| AFLA_068300 | 1,3-beta-glucanosyltransferase Bgt1 |
| AFLA_068310 | myosin class II heavy chain (MHC), putative |
| AFLA_068320 | conserved hypothetical protein |
| AFLA_068330 | RNA export mediator Gle1, putative |
| AFLA_068340 | cell wall biogenesis protein phosphatase Ssd1, putative |
| AFLA_068400 | 3-ketosphinganine reductase (Tsc10), putative |
| AFLA_068410 | Sterol desaturase family |
| AFLA_068460 | conserved hypothetical protein |
| AFLA_068470 | histone-lysine N-methyltransferase Clr4 |
| AFLA_068490 | G-patch domain protein, putative |
| AFLA_068510 | hypothetical protein |
| AFLA_068530 | ribosomal RNA processing protein, putative |
| AFLA_068630 | nuclear mRNA splicing factor-associated protein, putative |
| AFLA_068640 | hypothetical protein |
| AFLA_068680 | P-type calcium ATPase, putative |
| AFLA_068700 | conserved hypothetical protein |
| AFLA_068820 | hypothetical protein |
| AFLA_068850 | neutral amino acid permease, putative |
| AFLA_068910 | conserved hypothetical protein |
| AFLA_068940 | proteasome regulatory particle subunit (Nas6), putative |
| AFLA_069030 | conserved hypothetical protein |
| AFLA_069090 | GPI anchored protein, putative |
| AFLA_069220 | conserved hypothetical protein |
| AFLA_069250 | hypothetical protein |
| AFLA_069380 | integral membrane protein, Mpv17/PMP22 family, putative |
| AFLA_069440 | pyridoxal reductase (AKR8), putative |
| AFLA_069560 | conserved hypothetical protein |
| AFLA_069680 | 3-oxoacyl-(acyl-carrier-protein) reductase |
| AFLA_069730 | HMG box protein, putative |
| AFLA_069920 | GTP-binding protein YchF |
| AFLA_070070 | conserved hypothetical protein |
| AFLA_070170 | lysosomal alpha-glucosidase, putative |
| AFLA_070190 | hypothetical protein |
| AFLA_070230 | hypothetical protein |
| AFLA_070250 | conserved hypothetical protein |
| AFLA_070270 | siderophore iron transporter, putative |
| AFLA_070280 | conserved hypothetical protein |
| AFLA_070380 | conserved hypothetical protein |
| AFLA_070390 | conserved hypothetical protein |
| AFLA_070400 | conserved hypothetical protein |
| AFLA_070610 | conserved hypothetical protein |
| AFLA_070680 | molybdopterin synthase small subunit CnxG |
| AFLA_070820 | 3-hydroxymethyl-3-methylglutaryl-Coenzyme A lyase, putative |
| AFLA_070930 | sugar transporter, putative |
| AFLA_070970 | C6 transcription factor, putative |
| AFLA_071030 | PHD finger domain protein, putative |
| AFLA_071040 | nicotinate phosphoribosyltransferase |
| AFLA_071380 | conserved hypothetical protein |
| AFLA_071390 | mRNA binding post-transcriptional regulator (Csx1), putative |
| AFLA_071400 | conserved hypothetical protein |
| AFLA_071420 | TRAPP complex component Bet3, putative |
| AFLA_071430 | U5 snRNP complex subunit, putative |
| AFLA_071440 | conserved hypothetical protein |
| AFLA_071510 | C2 domain protein |
| AFLA_071630 | polysaccharide synthase Cps1, putative |
| AFLA_071780 | Dyp-type peroxidase family protein |
| AFLA_071790 | galactose oxidase, putative |
| AFLA_072280 | hypothetical protein |
| AFLA_072320 | sugar transporter, putative |
| AFLA_072330 | disulfide oxidoreductase, putative |
| AFLA_072550 | dimethylaniline monooxygenase, putative |
| AFLA_072570 | conserved hypothetical protein |
| AFLA_072580 | hypothetical protein |
| AFLA_072620 | proline-specific permease, putative |
| AFLA_072710 | hypothetical protein |
| AFLA_072760 | conserved hypothetical protein |
| AFLA_072820 | metallophosphoesterase domain-containing protein, putative |
| AFLA_072830 | sugar transporter, putative |
| AFLA_072870 | conserved hypothetical protein |
| AFLA_072880 | conserved hypothetical protein |
| AFLA_072920 | amino acid transporter, putative |
| AFLA_073180 | MFS monosaccharide transporter, putative |
| AFLA_073330 | conserved hypothetical protein |
| AFLA_073450 | copper amine oxidase, putative |
| AFLA_073470 | conserved hypothetical protein |
| AFLA_073580 | cell division control protein Cdc6, putative |
| AFLA_073590 | UBX domain protein (Ubx5), putative |
| AFLA_073850 | TBC domain protein, putative |
| AFLA_073870 | C6 transcription factor RegA, putative |
| AFLA_073940 | Ulp1 protease family protein |
| AFLA_073950 | Rho guanyl nucleotide exchange factor, putative |
| AFLA_073970 | conserved hypothetical protein |
| AFLA_074190 | hypothetical protein |
| AFLA_074270 | sugar transporter, putative |
| AFLA_074300 | hypothetical protein |
| AFLA_074460 | hypothetical protein |
| AFLA_074490 | decarboxylase DEC1, putative |
| AFLA_074560 | cytochrome P450, putative |
| AFLA_074570 | efflux pump antibiotic resistance protein, putative |
| AFLA_074630 | MFS nicotinic acid transporter Tna1, putative |
| AFLA_074730 | hypothetical protein |
| AFLA_074740 | conserved hypothetical protein |
| AFLA_074850 | hypothetical protein |
| AFLA_074870 | hypothetical protein |
| AFLA_074880 | conserved hypothetical protein |
| AFLA_074910 | conserved hypothetical protein |
| AFLA_074990 | DNA N-glycosylase, putative |
| AFLA_075060 | transformer-SR ribonucleoprotein, putative |
| AFLA_075300 | salicylate hydroxylase, putative |
| AFLA_075320 | C6 transcription factor, putative |
| AFLA_075330 | conserved hypothetical protein |
| AFLA_075400 | quinone oxidoreductase, putative |
| AFLA_075510 | general amidase, putative |
| AFLA_075520 | conserved hypothetical protein |
| AFLA_075580 | conserved hypothetical protein |
| AFLA_075640 | pigment biosynthesis protein Ayg1 |
| AFLA_075650 | hypothetical protein |
| AFLA_075680 | conserved hypothetical protein |
| AFLA_075770 | dienelactone hydrolase family protein |
| AFLA_075890 | conserved hypothetical protein |
| AFLA_075900 | conserved hypothetical protein |
| AFLA_075960 | conserved hypothetical protein |
| AFLA_075980 | choline monooxygenase, putative |
| AFLA_076010 | zinc finger protein, putative |
| AFLA_076090 | mitochondrial carrier protein, putative |
| AFLA_076200 | HAD superfamily hydrolase, putative |
| AFLA_076290 | capsule associated protein, putative |
| AFLA_076370 | ankyrin repeat-containing protein, putative |
| AFLA_076520 | GPI maturation protein (Bst1), putative |
| AFLA_076570 | conserved hypothetical protein |
| AFLA_076620 | siroheme synthase Met8, putative |
| AFLA_076630 | ribose phosphate diphosphokinase Prs1, putative |
| AFLA_076760 | vacuolar morphogenesis protein AvaB, putative |
| AFLA_076780 | ribonucleoprotein, putative |
| AFLA_076910 | cytokinesis EF-hand protein Cdc4, putative |
| AFLA_077030 | siderochrome-iron transporter Sit1, putative |
| AFLA_077040 | pectate lyase, putative |
| AFLA_077110 | hypothetical protein |
| AFLA_077200 | conserved hypothetical protein |
| AFLA_077220 | NADPH flavin oxidoreductase, putative |
| AFLA_077230 | hypothetical protein |
| AFLA_077280 | acyl-CoA dehydrogenase, putative |
| AFLA_077300 | sugar transporter, putative |
| AFLA_077380 | sugar transporter, putative |
| AFLA_077390 | conserved hypothetical protein |
| AFLA_077580 | ribonuclease p/mrp subunit, putative |
| AFLA_077800 | conserved hypothetical protein |
| AFLA_077910 | alpha-1,3-glucanase, putative |
| AFLA_077930 | conserved hypothetical protein |
| AFLA_077950 | toxin biosynthesis protein (Tri7), putative |
| AFLA_078030 | conserved hypothetical protein |
| AFLA_078040 | (S)-2-haloacid dehalogenase IVA, putative |
| AFLA_078050 | cytochrome P450 monooxygenase, putative |
| AFLA_078080 | conserved hypothetical protein |
| AFLA_078120 | cell wall glucanase/allergen F16-like protein |
| AFLA_078140 | acetyltransferase, GNAT family |
| AFLA_078240 | conserved hypothetical protein |
| AFLA_078370 | oxidoreductase, short chain dehydrogenase/reductase family |
| AFLA_078480 | cysteine protease, putative |
| AFLA_078540 | conserved hypothetical protein |
| AFLA_078600 | DNA repair/transcription protein, putative |
| AFLA_078610 | conserved hypothetical protein |
| AFLA_078640 | methylenetetrahydrofolate dehydrogenase |
| AFLA_078760 | cysteine synthase, putative |
| AFLA_078770 | Myb-like DNA-binding domain protein |
| AFLA_078780 | autophagy protein Apg6, putative |
| AFLA_078800 | peroxisomal membrane protein (Pex13), putative |
| AFLA_079060 | conserved hypothetical protein |
| AFLA_079170 | peroxisomal membrane protein (PmpP24), putative |
| AFLA_079190 | acetoacetyl-CoA synthase |
| AFLA_079200 | N-ethylmaleimide reductase, putative |
| AFLA_079390 | hypothetical protein |
| AFLA_079560 | conserved hypothetical protein |
| AFLA_079580 | cytochrome b561, putative |
| AFLA_079610 | conserved hypothetical protein |
| AFLA_080020 | ATP synthase gamma chain, mitochondrial precursor, putative |
| AFLA_080320 | conserved hypothetical protein |
| AFLA_080430 | hypothetical protein |
| AFLA_080550 | conserved hypothetical protein |
| AFLA_080570 | F-actin capping protein beta subunit |
| AFLA_080630 | NADH-quinone oxidoreductase Pst2, putative |
| AFLA_080770 | 2-nitropropane dioxygenase precursor, putative |
| AFLA_080780 | hypothetical protein |
| AFLA_080920 | tubulin alpha-1 subunit |
| AFLA_080980 | conserved hypothetical protein |
| AFLA_080990 | MFS sugar transporter, putative |
| AFLA_081220 | conserved hypothetical protein |
| AFLA_081240 | conserved hypothetical protein |
| AFLA_081260 | 60S ribosome biogenesis protein Brx1, putative |
| AFLA_081280 | Ras GTPase Rab11, putative |
| AFLA_081290 | hypothetical protein |
| AFLA_081390 | pachytene checkpoint component Pch2, putative |
| AFLA_081410 | secretion pathway protein Sls2/Rcy1, putative |
| AFLA_081460 | DNA mismatch repair protein Msh4, putative |
| AFLA_081510 | conserved hypothetical protein |
| AFLA_081520 | conserved hypothetical protein |
| AFLA_081660 | hypothetical protein |
| AFLA_081730 | integral membrane protein |
| AFLA_081880 | branched-chain amino acid aminotransferase |
| AFLA_081890 | conserved hypothetical protein |
| AFLA_082040 | DUF614 domain protein |
| AFLA_082070 | pantothenate transporter, putative |
| AFLA_082210 | conserved hypothetical protein |
| AFLA_082280 | glutathione S-transferase, putative |
| AFLA_082360 | conserved hypothetical protein |
| AFLA_082370 | conserved hypothetical protein |
| AFLA_082620 | conserved hypothetical protein |
| AFLA_082740 | AMP dependent ligase, putative |
| AFLA_082750 | hypothetical protein |
| AFLA_082900 | Swr1p complex component (Swc5), putative |
| AFLA_082920 | conserved hypothetical protein |
| AFLA_083110 | conidiation-specific protein (Con-10), putative |
| AFLA_083150 | hypothetical protein |
| AFLA_083160 | molybdopterin cofactor biosynthetic protein |
| AFLA_083280 | hypothetical protein |
| AFLA_083330 | C6 transcription factor, putative |
| AFLA_083340 | threonyl-tRNA synthetase |
| AFLA_083350 | conserved hypothetical protein |
| AFLA_083360 | extracellular cell wall glucanase Crf1/allergen Asp F9 |
| AFLA_083400 | Auxin Efflux Carrier superfamily |
| AFLA_083500 | diaminopropionate ammonia-lyase, putative |
| AFLA_083620 | protein phosphatase 2C, putative |
| AFLA_083640 | 3-isopropylmalate dehydrogenase Leu2A |
| AFLA_083740 | 40S ribosomal protein S22 |
| AFLA_083920 | adenylosuccinate synthetase AdB |
| AFLA_083990 | JmjC domain protein, putative |
| AFLA_084020 | homeobox and C2H2 transcription factor, putative |
| AFLA_084070 | CUE domain protein, putative |
| AFLA_084080 | dimethylallyl tryptophan synthase, putative |
| AFLA_084090 | C6 transcription factor, putative |
| AFLA_084100 | 2-oxoglutarate dehydrogenase, putative |
| AFLA_084110 | 2-oxoglutarate dehydrogenase, putative |
| AFLA_084150 | peroxisome biosynthesis protein (Peroxine-7), putative |
| AFLA_084590 | Hsp70 family chaperone Lhs1/Orp150, putative |
| AFLA_084640 | conserved hypothetical protein |
| AFLA_084710 | sugar 1,4-lactone oxidase, putative |
| AFLA_084940 | conserved hypothetical protein |
| AFLA_084970 | C2H2 transcription factor, putative |
| AFLA_084980 | hypothetical protein |
| AFLA_084990 | conserved hypothetical protein |
| AFLA_085270 | t-complex protein 1, zeta subunit, putative |
| AFLA_085280 | pre-rRNA processing nucleolar protein Sik1, putative |
| AFLA_085350 | AMMECR1 family protein |
| AFLA_085360 | regulatory factor Sgt1, putative |
| AFLA_085370 | spindle-pole body protein (Pcp1), putative |
| AFLA_085400 | phosphoglycerate mutase, 2,3-bisphosphoglycerate-independent |
| AFLA_085510 | trytophan synthase alpha subunit, putative |
| AFLA_085520 | glycan biosynthesis protein (PigL), putative |
| AFLA_085550 | hypothetical protein |
| AFLA_085640 | hypothetical protein |
| AFLA_085650 | conserved hypothetical protein |
| AFLA_085660 | arsenic resistance protein ArsH |
| AFLA_085740 | C6 transcription factor, putative |
| AFLA_085780 | RING finger domain protein |
| AFLA_085790 | conserved hypothetical protein |
| AFLA_085870 | conserved hypothetical protein |
| AFLA_085950 | alcohol dehydrogenase, putative |
| AFLA_085960 | cell surface protein, putative |
| AFLA_085970 | DNA primase subunit Pri1, putative |
| AFLA_086130 | NUDIX domain, putative |
| AFLA_086150 | conserved hypothetical protein |
| AFLA_086160 | tripeptidyl peptidase SED3 |
| AFLA_086190 | 5-carboxymethyl-2-hydroxymuconate semialdehyde dehydrogenase, putative |
| AFLA_086200 | betaine-aldehyde dehydrogenase, putative |
| AFLA_086210 | epoxide hydrolase, putative |
| AFLA_086520 | conserved hypothetical protein |
| AFLA_086550 | conserved hypothetical protein |
| AFLA_086560 | short-chain dehydrogenase/reductase family protein, putative |
| AFLA_086660 | mitochondrial phosphate carrier protein, putative |
| AFLA_086670 | cystathionine beta-synthase (beta-thionase), putative |
| AFLA_086740 | 4-nitrophenylphosphatase |
| AFLA_086800 | epoxide hydrolase, putative |
| AFLA_086870 | MRS7 family protein |
| AFLA_087100 | benzoate 4-monooxygenase cytochrome P450, putative |
| AFLA_087130 | conserved hypothetical protein |
| AFLA_087450 | conserved hypothetical protein |
| AFLA_087470 | plasma membrane low affinity zinc ion transporter, putative |
| AFLA_087680 | NADH:flavin oxidoreductase/NADH oxidase family protein |
| AFLA_087710 | GPR/FUN34 family protein |
| AFLA_087820 | cell wall integrity signaling protein Lsp1/Pil1, putative |
| AFLA_087840 | WD repeat protein |
| AFLA_087860 | cellulose-binding GDSL lipase/acylhydrolase, putative |
| AFLA_087940 | conserved hypothetical protein |
| AFLA_087950 | isocitrate dehydrogenase LysB |
| AFLA_088250 | transcription initiation factor TFIID subunit 13, putative |
| AFLA_088270 | nucleolus protein required for cell viability, putative |
| AFLA_088280 | DUF6 domain protein, putative |
| AFLA_088300 | cholinephosphate cytidylyltransferase |
| AFLA_088350 | hydrolase, alpha/beta fold family protein |
| AFLA_088460 | ribosome biogenesis protein Erb1, putative |
| AFLA_088570 | conserved hypothetical protein |
| AFLA_088580 | conserved hypothetical protein |
| AFLA_088670 | pheromone processing carboxypeptidase Kex1 |
| AFLA_089090 | actin cytoskeleton organization and biogenesis protein, putative |
| AFLA_089120 | inositol monophosphatase, putative |
| AFLA_089150 | transcription initiation factor IIF subunit alpha, putative |
| AFLA_089240 | glutamyl-tRNA amidotransferase, A subunit, putative |
| AFLA_089290 | conserved hypothetical protein |
| AFLA_089440 | conserved hypothetical protein |
| AFLA_089450 | GABA permease, putative |
| AFLA_089470 | glycine-rich protein, putative |
| AFLA_089570 | 2,3-diketo-5-methylthio-1-phosphopentane phosphatase, putative |
| AFLA_089770 | alpha-N-arabinofuranosidase A precursor, putative |
| AFLA_089800 | conserved hypothetical protein |
| AFLA_089810 | sodium/solute symporter, putative |
| AFLA_089860 | lipase 1 precursor, putative |
| AFLA_089930 | rhamnogalacturonase A precursor, putative |
| AFLA_089950 | conserved hypothetical protein |
| AFLA_089960 | conserved hypothetical protein |
| AFLA_090010 | conserved hypothetical protein |
| AFLA_090030 | aryl-alcohol dehydrogenase, putative |
| AFLA_090180 | alpha/beta hydrolase, putative |
| AFLA_090190 | dimethylallyl tryptophan synthase, putative |
| AFLA_090200 | nonribosomal peptide synthase, putative |
| AFLA_090330 | U2 auxiliary factor small subunit, putative |
| AFLA_090470 | srpk, putative |
| AFLA_090630 | cytochrome P450, putative |
| AFLA_090680 | cytochrome P450, putative |
| AFLA_090890 | ATP synthase subunit E, putative |
| AFLA_090940 | peptidyl prolyl cis-trans isomerase (CypC), putative |
| AFLA_091000 | cytochrome P450 monooxygenase, putative |
| AFLA_091040 | ribonuclease P complex subunit Pop1, putative |
| AFLA_091050 | hypothetical protein |
| AFLA_091220 | WD repeat protein |
| AFLA_091240 | conserved hypothetical protein |
| AFLA_091250 | 60S ribosomal protein L24a |
| AFLA_091270 | fumarate hydratase, putative |
| AFLA_091280 | hypothetical protein |
| AFLA_091330 | conserved hypothetical protein |
| AFLA_091340 | actin associated protein Wsp1, putative |
| AFLA_091350 | magnesium ion transporter (Mrs2), putative |
| AFLA_091400 | 2-dehydropantoate 2-reductase, putative |
| AFLA_091430 | conserved hypothetical protein |
| AFLA_091440 | conserved hypothetical protein |
| AFLA_091450 | G2-specific protein kinase NimA, putative |
| AFLA_091460 | ankyrin repeat and BTB/POZ domain protein |
| AFLA_091540 | conserved hypothetical protein |
| AFLA_091560 | FMN dependent dehydrogenase, putative |
| AFLA_091570 | MFS transporter, putative |
| AFLA_091750 | conserved hypothetical protein |
| AFLA_091760 | C-3 sterol dehydrogenase/C-4 decarboxylase family protein |
| AFLA_091770 | integral membrane protein |
| AFLA_091920 | alpha-1,6-mannosyltransferase subunit (Och1), putative |
| AFLA_092090 | oxidoreductase, short-chain dehydrogenase/reductase family |
| AFLA_092100 | IMP dehydrogenase, putative |
| AFLA_092110 | NUDIX family hydrolase, putative |
| AFLA_092160 | two-component osmosensing histidine kinase (Bos1), putative |
| AFLA_092190 | conserved hypothetical protein |
| AFLA_092200 | hypothetical protein |
| AFLA_092220 | hypothetical protein |
| AFLA_092230 | sugar transporter, putative |
| AFLA_092250 | conserved hypothetical protein |
| AFLA_092300 | hypothetical protein |
| AFLA_092380 | conserved hypothetical protein |
| AFLA_092460 | bZIP transcription factor, putative |
| AFLA_092470 | glycosyl hydrolase, putative |
| AFLA_092530 | hypothetical protein |
| AFLA_092550 | hypothetical protein |
| AFLA_092560 | conserved hypothetical protein |
| AFLA_092570 | conserved hypothetical protein |
| AFLA_092640 | alkaline phosphatase Pho8 |
| AFLA_092690 | Leucine carboxyl methyltransferase family |
| AFLA_092700 | protein tyrosine phosphatase Pps1, putative |
| AFLA_092710 | DNA mismatch repair protein Mlh1, putative |
| AFLA_092720 | conserved hypothetical protein |
| AFLA_092730 | 26S proteasome regulatory subunit Mts4, putative |
| AFLA_092880 | protein kinase, putative |
| AFLA_093310 | conserved hypothetical protein |
| AFLA_093320 | pseudouridine synthase |
| AFLA_093350 | WD repeat protein |
| AFLA_093630 | conserved hypothetical protein |
| AFLA_093640 | U6 small nuclear ribonucleoprotein (Lsm3), putative |
| AFLA_093680 | conserved hypothetical protein |
| AFLA_093740 | UPF0052 domain protein |
| AFLA_093800 | small nuclear ribonucleoprotein SmD2, putative |
| AFLA_093940 | mitochondrial large ribosomal subunit YmL35, putative |
| AFLA_093960 | mitochondrial 3-hydroxyisobutyryl-CoA hydrolase, putative |
| AFLA_094030 | Snf1 protein kinase complex subunit Snf4, putative |
| AFLA_094040 | CRAL/TRIO domain protein |
| AFLA_094070 | translation initiation factor eif-2b delta subunit |
| AFLA_094080 | kinesin family protein |
| AFLA_094090 | conserved hypothetical protein |
| AFLA_094100 | DUF1275 domain protein |
| AFLA_094340 | kinesin family protein |
| AFLA_094500 | peptidyl-prolyl cis-trans isomerase, putative |
| AFLA_094540 | transmembrane GTPase Fzo1, putative |
| AFLA_094600 | conserved hypothetical protein |
| AFLA_094730 | exosome complex subunit Csl4, putative |
| AFLA_094760 | conserved hypothetical protein |
| AFLA_094770 | conserved hypothetical protein |
| AFLA_094900 | vacuolar armadillo repeat protein Vac8, putative |
| AFLA_094920 | hypothetical protein |
| AFLA_094930 | hypothetical protein |
| AFLA_095280 | conserved hypothetical protein |
| AFLA_095340 | cellobiose dehydrogenase, putative |
| AFLA_095350 | conserved hypothetical protein |
| AFLA_095380 | siderophore iron transporter, putative |
| AFLA_095460 | conserved hypothetical protein |
| AFLA_095550 | conserved hypothetical protein |
| AFLA_095560 | conserved hypothetical protein |
| AFLA_095580 | SNF7 family protein |
| AFLA_095590 | Hsp90 binding co-chaperone (Sba1), putative |
| AFLA_095840 | hypothetical protein |
| AFLA_096010 | conserved hypothetical protein |
| AFLA_096060 | conserved hypothetical protein |
| AFLA_096070 | sugar transporter, putative |
| AFLA_096140 | conserved hypothetical protein |
| AFLA_096150 | MFS alpha-glucoside transporter, putative |
| AFLA_096280 | conserved hypothetical protein |
| AFLA_096320 | C6 and C2H2 transcription factor |
| AFLA_096330 | C6 and C2H2 transcription factor |
| AFLA_096560 | conserved hypothetical protein |
| AFLA_096760 | monocarboxylate transporter, putative |
| AFLA_096780 | conserved hypothetical protein |
| AFLA_097130 | conserved hypothetical protein |
| AFLA_097140 | conserved hypothetical protein |
| AFLA_097150 | conserved hypothetical protein |
| AFLA_097210 | hypothetical protein |
| AFLA_097300 | conserved hypothetical protein |
| AFLA_097310 | conserved hypothetical protein |
| AFLA_097350 | amino acid transporter, putative |
| AFLA_097360 | MFS peptide transporter, putative |
| AFLA_097440 | conserved hypothetical protein |
| AFLA_097450 | conserved hypothetical protein |
| AFLA_097510 | cytochrome P450 monooxygenase, putative |
| AFLA_097610 | gluconolactonase precursor, putative |
| AFLA_097670 | conserved hypothetical protein |
| AFLA_097680 | C6 transcription factor, putative |
| AFLA_097690 | cysteine dioxygenase Cdo1, putative |
| AFLA_097750 | conserved hypothetical protein |
| AFLA_097800 | hypothetical protein |
| AFLA_097890 | Glycosyl hydrolases family 32 superfamily |
| AFLA_098110 | Indoleamine 2,3-dioxygenase subfamily |
| AFLA_098210 | conserved hypothetical protein |
| AFLA_098250 | cation efflux family protein, putative |
| AFLA_098290 | succinate dehydrogenase cytochrome b560 subunit |
| AFLA_098300 | transaldolase, putative |
| AFLA_098400 | hypothetical protein |
| AFLA_098420 | WW domain protein |
| AFLA_098500 | conserved hypothetical protein |
| AFLA_098520 | conserved hypothetical protein |
| AFLA_098670 | clock controlled protein (Ccg-8), putative |
| AFLA_098690 | conserved hypothetical protein |
| AFLA_098720 | conserved hypothetical protein |
| AFLA_098740 | hypothetical protein |
| AFLA_098760 | conserved hypothetical protein |
| AFLA_098770 | Phospholipase/Carboxylesterase superfamily |
| AFLA_098790 | phosphatidylethanolamine-binding protein, putative |
| AFLA_098840 | L-amino acid oxidase, putative |
| AFLA_098920 | conserved hypothetical protein |
| AFLA_098930 | DUF1479 domain protein |
| AFLA_099000 | Cu,Zn superoxide dismutase SOD1 |
| AFLA_099050 | conserved hypothetical protein |
| AFLA_099090 | hypothetical protein |
| AFLA_099160 | hypothetical protein |
| AFLA_099210 | conserved hypothetical protein |
| AFLA_099300 | triglyceride lipase-cholesterol esterase, putative |
| AFLA_099340 | conserved hypothetical protein |
| AFLA_099540 | conserved hypothetical protein |
| AFLA_099560 | oxidoreductase, short-chain dehydrogenase/reductase family |
| AFLA_099570 | conserved hypothetical protein |
| AFLA_099630 | conserved hypothetical protein |
| AFLA_099640 | transcriptional regulator (Cti6), putative |
| AFLA_099700 | L-kynurenine/alpha-aminoadipate aminotransferase, putative |
| AFLA_099780 | cell wall glucanase, putative |
| AFLA_099810 | zinc finger protein DHHC domain containing protein, putative |
| AFLA_099900 | integral membrane protein |
| AFLA_099910 | hypothetical protein |
| AFLA_099950 | conserved hypothetical protein |
| AFLA_100060 | ran GTPase activating protein 1 (RNA1 protein) |
| AFLA_100110 | plasma membrane H+-ATPase Pma1 |
| AFLA_100120 | hypothetical protein |
| AFLA_100180 | phosphoribosyl-aminoimidazole-succinocarboxamide synthase |
| AFLA_100200 | conserved hypothetical protein |
| AFLA_100220 | conserved hypothetical protein |
| AFLA_100320 | hypothetical protein |
| AFLA_100380 | hypothetical protein |
| AFLA_100540 | oxidoreductase, putative |
| AFLA_100560 | conserved hypothetical protein |
| AFLA_100590 | cytochrome P450, putative |
| AFLA_100660 | conserved hypothetical protein |
| AFLA_100690 | 3'-phosphoadenosine-5'-phosphosulfate reductase Met16, putative |
| AFLA_100700 | phosphoserine phosphatase |
| AFLA_100760 | conserved hypothetical protein |
| AFLA_100860 | Rho GTPase Rho3 |
| AFLA_100870 | COP9 signalosome subunit 2 (CsnB), putative |
| AFLA_100970 | RNA-directed RNA polymerase (Sad-1), putative |
| AFLA_101130 | C2H2 zinc finger domain protein, putative |
| AFLA_101150 | 60S ribosomal protein L21, putative |
| AFLA_101160 | 40S ribosomal protein S9 |
| AFLA_101180 | conserved hypothetical protein |
| AFLA_101220 | annexin ANXC4 |
| AFLA_101230 | glutaminase GtaA |
| AFLA_101320 | UPF0016 domain protein, putative |
| AFLA_101340 | MFS multidrug transporter, putative |
| AFLA_101370 | conserved hypothetical protein |
| AFLA_101550 | thioredoxin reductase, putative |
| AFLA_101700 | NRPS-like enzyme, putative |
| AFLA_101710 | NmrA-like family protein |
| AFLA_101760 | molybdenum cofactor biosynthesis protein Gephyrin, putative |
| AFLA_101770 | conserved hypothetical protein |
| AFLA_101790 | hypothetical protein |
| AFLA_101910 | sensor histidine kinase/response regulator, putative |
| AFLA_101920 | extracellular developmental signal biosynthesis protein FluG |
| AFLA_102000 | hypothetical protein |
| AFLA_102180 | SD08430p |
| AFLA_102190 | conserved hypothetical protein |
| AFLA_102200 | conserved hypothetical protein |
| AFLA_102350 | conserved hypothetical protein |
| AFLA_102370 | conserved hypothetical protein |
| AFLA_102500 | 9-cis-epoxycarotenoid dioxygenase, putative |
| AFLA_102520 | phytoene dehydrogenase, putative |
| AFLA_102540 | hypothetical protein |
| AFLA_102710 | DUF1275 domain protein |
| AFLA_102730 | conserved hypothetical protein |
| AFLA_102750 | conserved hypothetical protein |
| AFLA_102910 | RNA polymerase II mediator complex component Srb8, putative |
| AFLA_102980 | SET domain-containing protein, putative |
| AFLA_102990 | Coenzyme Q (ubiquinone) biosynthesis protein Coq4, putative |
| AFLA_103000 | ubiquinone biosynthesis protein, putative |
| AFLA_103070 | hypothetical protein |
| AFLA_103090 | conserved hypothetical protein |
| AFLA_103100 | iron-sulfur cluster biosynthesis protein Isd11, putative |
| AFLA_103110 | conserved hypothetical protein |
| AFLA_103240 | hexaprenyl pyrophosphate synthetase Coq1, putative |
| AFLA_103250 | proteasome regulatory particle subunit (RpnC), putative |
| AFLA_103260 | conserved hypothetical protein |
| AFLA_103310 | Flap endonuclease Rad27, putative |
| AFLA_103570 | ZZ type zinc finger domain protein |
| AFLA_103580 | serum paraoxonase/arylesterase family protein |
| AFLA_103650 | aldose 1-epimerase, putative |
| AFLA_103850 | condensin complex component cnd1 |
| AFLA_103970 | mitochondrial translation release factor (RF-1), putative |
| AFLA_103980 | DnaJ and TPR domain protein |
| AFLA_104430 | subtilisin, putative |
| AFLA_104450 | conserved hypothetical protein |
| AFLA_104460 | carboxylesterase, type B, putative |
| AFLA_104640 | Asp hemolysin-like protein |
| AFLA_104660 | sensory transduction histidine kinase, putative |
| AFLA_104710 | conserved hypothetical protein |
| AFLA_104830 | integral membrane protein Pth11-like, putative |
| AFLA_104840 | tannase, putative |
| AFLA_104890 | hydrolase, putative |
| AFLA_104970 | conserved hypothetical protein |
| AFLA_105000 | DUF636 domain protein |
| AFLA_105010 | methyltransferase, putative |
| AFLA_105050 | geranylgeranyl pyrophosphate synthetase AtmG, putative |
| AFLA_105170 | O-methyltransferase, putative |
| AFLA_105190 | NRPS-like enzyme, putative |
| AFLA_105300 | metallo-beta-lactamase domain protein, putative |
| AFLA_105320 | phosphoserine aminotransferase |
| AFLA_105480 | DUF636 domain protein |
| AFLA_105530 | C6 transcription factor, putative |
| AFLA_105600 | conserved hypothetical protein |
| AFLA_105620 | protein kinase, putative |
| AFLA_105720 | hypothetical protein |
| AFLA_105740 | sulfide quinone reductase, putative |
| AFLA_105750 | metallo-beta-lactamase domain protein, putative |
| AFLA_105760 | N,N-dimethylglycine oxidase, putative |
| AFLA_105870 | conserved hypothetical protein |
| AFLA_105940 | cysteinyl-tRNA synthetase |
| AFLA_106190 | cytochrome P450, putative |
| AFLA_106230 | carboxylesterase, putative |
| AFLA_106250 | HAD superfamily hydrolase, putative |
| AFLA_106270 | conserved hypothetical protein |
| AFLA_106280 | nucleoporin NUP49/NSP49, putative |
| AFLA_106320 | cleavage and polyadenylylation specificity factor subunit Fip1 |
| AFLA_106340 | E3 ubiquitin-protein ligase CCNB1IP1, putative |
| AFLA_106460 | DNA polymerase delta subunit 4, putative |
| AFLA_106470 | arrestin domain protein |
| AFLA_106540 | conserved hypothetical protein |
| AFLA_106710 | conserved hypothetical protein |
| AFLA_106750 | sugar transporter, putative |
| AFLA_106780 | CRAL/TRIO domain protein |
| AFLA_106790 | C6 transcription factor, putative |
| AFLA_106800 | guanine deaminase, putative |
| AFLA_106870 | alkaline serine protease AorO, putative |
| AFLA_107110 | conserved hypothetical protein |
| AFLA_107140 | conserved hypothetical protein |
| AFLA_107170 | conserved hypothetical protein |
| AFLA_107230 | small oligopeptide transporter, OPT family |
| AFLA_107240 | fructosyl amine: oxygen oxidoreductase |
| AFLA_107250 | PAP2 domain protein |
| AFLA_107260 | RNA polymerase II mediator complex protein (Med7), putative |
| AFLA_107540 | transketolase, putative |
| AFLA_107550 | alcohol dehydrogenase, putative |
| AFLA_107620 | conserved hypothetical protein |
| AFLA_107720 | fumarylacetoacetate hydrolase, putative |
| AFLA_107730 | conserved hypothetical protein |
| AFLA_108070 | conserved hypothetical protein |
| AFLA_108220 | conserved hypothetical protein |
| AFLA_108350 | alpha/beta hydrolase, putative |
| AFLA_108360 | integral membrane protein, putative |
| AFLA_108440 | phenol 2-monooxygenase, putative |
| AFLA_108460 | nonsense-mediated mRNA decay protein Upf3, putative |
| AFLA_108740 | hypothetical protein |
| AFLA_108790 | aldehyde dehydrogenase AldA, putative |
| AFLA_108830 | meiotic recombination protein Mre11 |
| AFLA_108840 | F-box and WD domain protein |
| AFLA_108930 | hypothetical protein |
| AFLA_108960 | bifunctional pyrimidine biosynthesis protein (PyrABCN), putative |
| AFLA_108980 | integral membrane channel protein, putative |
| AFLA_109080 | high-affinity glucose transporter, putative |
| AFLA_109130 | conserved hypothetical protein |
| AFLA_109450 | apoptosis inducing factor, putative |
| AFLA_109860 | translation elongation factor eEF-1B gamma subunit, putative |
| AFLA_110060 | hypothetical protein |
| AFLA_110150 | conserved hypothetical protein |
| AFLA_110200 | hypothetical protein |
| AFLA_110380 | hypothetical protein |
| AFLA_110400 | hypothetical protein |
| AFLA_110410 | phosphate transporter |
| AFLA_110450 | GTPase activating protein for Arf, putative |
| AFLA_110520 | conserved hypothetical protein |
| AFLA_110540 | conserved hypothetical protein |
| AFLA_110550 | nucleolar GTPase, putative |
| AFLA_110560 | protein kinase, putative |
| AFLA_110600 | aminopeptidase |
| AFLA_110640 | ATP binding L-PSP endoribonuclease family protein, putative |
| AFLA_110840 | RING finger protein, putative |
| AFLA_110900 | conserved hypothetical protein |
| AFLA_110970 | conserved hypothetical protein |
| AFLA_111050 | conserved hypothetical protein |
| AFLA_111060 | calcium homeostasis protein Regucalcin, putative |
| AFLA_111150 | acyltransferase, putative |
| AFLA_111160 | mRNA capping nucleoside-triphosphatase, putative |
| AFLA_111170 | transcription initiation protein spt5 |
| AFLA_111180 | conserved hypothetical protein |
| AFLA_111200 | nuclear migration protein NudF |
| AFLA_111210 | 26S proteasome regulatory subunit Rpn2, putative |
| AFLA_111230 | conserved hypothetical protein |
| AFLA_111270 | conserved hypothetical protein |
| AFLA_111290 | conserved hypothetical protein |
| AFLA_111410 | YeeE/YedE family integral membrane protein |
| AFLA_111440 | hypothetical protein |
| AFLA_111470 | conserved hypothetical protein |
| AFLA_111500 | conserved hypothetical protein |
| AFLA_111590 | aldo-keto reductase, putative |
| AFLA_111710 | conserved hypothetical protein |
| AFLA_111840 | 2-hydroxychromene-2-carboxylate isomerase, putative |
| AFLA_111880 | conserved hypothetical protein |
| AFLA_111980 | 60S ribosomal protein L38, putative |
| AFLA_111990 | nuclear transport factor 2 domain protein, putative |
| AFLA_112000 | methionyl-tRNA synthetase, putative |
| AFLA_112080 | small nuclear ribonucleoprotein SmB, putative |
| AFLA_112120 | pyruvate carboxylase, putative |
| AFLA_112150 | phosphoribosylaminoimidazolecarboxamide formyltransferase/IMP |
| AFLA_112270 | translation initiation factor EF-2 gamma subunit, putative |
| AFLA_112280 | 60S ribosome biogenesis protein Mak11, putative |
| AFLA_112290 | MYB and HSA domain protein |
| AFLA_112420 | epoxide hydrolase, putative |
| AFLA_112450 | DNA methyltransferase 1-associated protein DMAP1 |
| AFLA_112690 | ATP dependent RNA helicase (Dob1), putative |
| AFLA_112710 | killer toxin sensitivity protein (IKI3), putative |
| AFLA_112900 | epoxide hydrolase, putative |
| AFLA_113020 | methionine aminopeptidase, type II, putative |
| AFLA_113030 | DnaJ domain protein |
| AFLA_113040 | outer mitochondrial membrane protein porin |
| AFLA_113070 | G-patch DNA repair protein (Drt111), putative |
| AFLA_113080 | coenzyme A synthetase, putative |
| AFLA_113130 | DNA-3-methyladenine glycosylase, putative |
| AFLA_113160 | conserved hypothetical protein |
| AFLA_113180 | ubiquinone biosynthesis protein, putative |
| AFLA_113280 | MFS monosaccharide transporter, putative |
| AFLA_113300 | monoxygenase, putative |
| AFLA_113320 | Glutamate/Leucine/Phenylalanine/Valine dehydrogenase, putative |
| AFLA_113370 | RNA polymerase II mediator complex subunit Nut1, putative |
| AFLA_113450 | bZIP transcription factor (MetR), putative |
| AFLA_113470 | RING finger domain protein (Znf1), putative |
| AFLA_113480 | DUF974 domain protein |
| AFLA_113500 | pentafunctional AROM polypeptide, putative |
| AFLA_113610 | ribosomal biogenesis protein Gar2 |
| AFLA_113660 | conserved hypothetical protein |
| AFLA_113760 | JmjC domain protein, putative |
| AFLA_113870 | conserved hypothetical protein |
| AFLA_113950 | hypothetical protein |
| AFLA_113960 | conserved hypothetical protein |
| AFLA_114090 | NADH-ubiquinone oxidoreductase 21 kDa subunit, putative |
| AFLA_114200 | hypothetical protein |
| AFLA_114210 | hypothetical protein |
| AFLA_114220 | hypothetical protein |
| AFLA_114230 | hypothetical protein |
| AFLA_114240 | hypothetical protein |
| AFLA_114250 | reverse transcriptase, putative |
| AFLA_114270 | conserved hypothetical protein |
| AFLA_114280 | pyrroline-5-carboxylate reductase, putative |
| AFLA_114450 | mucin family signaling protein Msb2, putative |
| AFLA_114750 | glutamyl-tRNA(Gln) amidotransferase subunit A, putative |
| AFLA_114790 | myo-inositol-1(or 4)-monophosphatase |
| AFLA_114800 | hypothetical protein |
| AFLA_114820 | polyketide synthase, putative |
| AFLA_114880 | MFS transporter, putative |
| AFLA_114950 | AP-2 adaptor complex subunit alpha, putative |
| AFLA_115100 | conserved hypothetical protein |
| AFLA_115140 | conserved hypothetical protein |
| AFLA_115220 | hypothetical protein |
| AFLA_115230 | conserved hypothetical protein |
| AFLA_115250 | conserved hypothetical protein |
| AFLA_115260 | ubiquinone biosynthesis protein, putative |
| AFLA_115270 | cytochrome c heme lyase, putative |
| AFLA_115330 | hypothetical protein |
| AFLA_115440 | NADH-ubiquinone oxidoreductase 21 kDa subunit, putative |
| AFLA_115460 | hypothetical protein |
| AFLA_115470 | hypothetical protein |
| AFLA_115770 | glycosyltransferase, putative |
| AFLA_115950 | conserved hypothetical protein |
| AFLA_115960 | conserved hypothetical protein |
| AFLA_116020 | conserved hypothetical protein |
| AFLA_116050 | conserved hypothetical protein |
| AFLA_116060 | conserved hypothetical protein |
| AFLA_116070 | conserved hypothetical protein |
| AFLA_116110 | C6 transcription factor, putative |
| AFLA_116170 | hypothetical protein |
| AFLA_116180 | hypothetical protein |
| AFLA_116190 | conserved hypothetical protein |
| AFLA_116200 | conserved hypothetical protein |
| AFLA_116220 | polyketide synthase, putative |
| AFLA_116240 | hypothetical protein |
| AFLA_116260 | N-hydroxyarylamine O-acetyltransferase, putative |
| AFLA_116270 | paraoxonase, putative |
| AFLA_116390 | amino acid transporter, putative |
| AFLA_116490 | conserved hypothetical protein |
| AFLA_116510 | conserved hypothetical protein |
| AFLA_116640 | conserved hypothetical protein |
| AFLA_116650 | pectate lyase, putative |
| AFLA_116660 | conserved hypothetical protein |
| AFLA_116670 | 4-hydroxy-2-oxovalerate aldolase, putative |
| AFLA_116780 | homocysteine S-methyltransferase, putative |
| AFLA_116930 | conserved hypothetical protein |
| AFLA_116970 | conserved hypothetical protein |
| AFLA_117000 | RNA exonuclease, putative |
| AFLA_117030 | conserved hypothetical protein |
| AFLA_117050 | RAC GTPase, putative |
| AFLA_117060 | conserved hypothetical protein |
| AFLA_117110 | conserved hypothetical protein |
| AFLA_117120 | hypothetical protein |
| AFLA_117240 | conserved hypothetical protein |
| AFLA_117260 | N-acetyltransferase, GNAT family, putative |
| AFLA_117270 | conserved hypothetical protein |
| AFLA_117440 | MFS sugar transporter, putative |
| AFLA_117450 | conserved hypothetical protein |
| AFLA_117460 | hypothetical protein |
| AFLA_117470 | hypothetical protein |
| AFLA_117550 | hypothetical protein |
| AFLA_117730 | DNA-directed polymerase kappa, putative |
| AFLA_118000 | integral membrane protein, Mpv17/PMP22 family, putative |
| AFLA_118050 | MFS peptide transporter Ptr2, putative |
| AFLA_118140 | SNARE domain protein |
| AFLA_118160 | AMP-binding enzyme, putative |
| AFLA_118350 | glutamate decarboxylase, putative |
| AFLA_118540 | conserved hypothetical protein |
| AFLA_118570 | short-chain dehydrogenases/reductase, putative |
| AFLA_118660 | conserved hypothetical protein |
| AFLA_118670 | penicillin-binding protein, putative |
| AFLA_118710 | NAD binding Rossmann fold oxidoreductase, putative |
| AFLA_118720 | flavin-containing monooxygenase, putative |
| AFLA_118760 | hypothetical protein |
| AFLA_118900 | conserved hypothetical protein |
| AFLA_118990 | efflux pump antibiotic resistance protein, putative |
| AFLA_119050 | conserved hypothetical protein |
| AFLA_119060 | conserved hypothetical protein |
| AFLA_119070 | conserved hypothetical protein |
| AFLA_119120 | beta-lactamase family protein |
| AFLA_119140 | short-chain dehydrogenases/reductase, putative |
| AFLA_119350 | monooxygenase, putative |
| AFLA_119420 | mitochondrial ATP-dependent RNA helicase Suv3, putative |
| AFLA_119430 | Sec1 family superfamily |
| AFLA_119460 | UDP-glucose 4-epimerase |
| AFLA_119480 | alpha-1,2-mannosyltransferase (Kre2), putative |
| AFLA_119500 | 20S proteasome maturation protein Ump1, putative |
| AFLA_119590 | ubiquinol-cytochrome c reductase iron-sulfur subunit precursor |
| AFLA_119600 | mitochondrion biogenesis protein (She9), putative |
| AFLA_119640 | cofilin |
| AFLA_120010 | amino acid transporter, putative |
| AFLA_120190 | 67 kDa myosin-cross-reactive antigen family protein |
| AFLA_120210 | conserved hypothetical protein |
| AFLA_120230 | short-chain dehydrogenase, putative |
| AFLA_120250 | peptidase, putative |
| AFLA_120420 | alanyl-tRNA synthetase, putative |
| AFLA_120430 | amino acid transporter, putative |
| AFLA_120470 | conserved hypothetical protein |
| AFLA_120550 | allantoate permease, putative |
| AFLA_120560 | conserved hypothetical protein |
| AFLA_120570 | short-chain dehydrogenase, putative |
| AFLA_120580 | hypothetical protein |
| AFLA_120590 | conserved hypothetical protein |
| AFLA_120760 | fatty acid oxygenase, putative |
| AFLA_120800 | ATP-binding cassette transporter, putative |
| AFLA_120810 | conserved hypothetical protein |
| AFLA_120820 | pyruvate carboxylase, putative |
| AFLA_120900 | integral membrane protein |
| AFLA_120960 | hypothetical protein |
| AFLA_120970 | alcohol dehydrogenase, putative |
| AFLA_121000 | conserved hypothetical protein |
| AFLA_121010 | integral membrane protein |
| AFLA_121020 | lipase 8 precursor, putative |
| AFLA_121070 | glycosyl hydrolase, putative |
| AFLA_121110 | conserved hypothetical protein |
| AFLA_121130 | conserved hypothetical protein |
| AFLA_121140 | conserved hypothetical protein |
| AFLA_121180 | conserved hypothetical protein |
| AFLA_121190 | zinc-binding oxidoreductase, putative |
| AFLA_121240 | phosphate-repressible phosphate permease, putative |
| AFLA_121280 | conserved hypothetical protein |
| AFLA_121320 | conserved hypothetical protein |
| AFLA_121360 | conserved hypothetical protein |
| AFLA_121460 | hypothetical protein |
| AFLA_121470 | hscarg dehydrogenase, putative |
| AFLA_121550 | conserved hypothetical protein |
| AFLA_121680 | cytochrome P450, putative |
| AFLA_122090 | conserved hypothetical protein |
| AFLA_122210 | oxidoreductase, putative |
| AFLA_122320 | NAD dependent epimerase/dehydratase, putative |
| AFLA_122440 | lipase/thioesterase family protein |
| AFLA_122510 | ABC multidrug transporter, putative |
| AFLA_122610 | rRNA processing protein CgrA |
| AFLA_122630 | cytidine deaminase, putative |
| AFLA_122680 | ELMO/CED-12 family protein |
| AFLA_122840 | conserved hypothetical protein |
| AFLA_123000 | hypothetical protein |
| AFLA_123240 | conserved hypothetical protein |
| AFLA_123260 | peroxisomal membrane protein 2, pxmp2, putative |
| AFLA_123280 | conserved hypothetical protein |
| AFLA_123300 | hypothetical protein |
| AFLA_123310 | conserved hypothetical protein |
| AFLA_123320 | carboxylesterase, putative |
| AFLA_123330 | conserved hypothetical protein |
| AFLA_123410 | GPI anchored cell wall protein, putative |
| AFLA_123510 | iron-sulfur domain containing protein, putative |
| AFLA_123520 | amino acid transporter, putative |
| AFLA_123530 | hypothetical protein |
| AFLA_123540 | conserved hypothetical protein |
| AFLA_123550 | fructosyl amino acid oxidase, putative |
| AFLA_123560 | conserved hypothetical protein |
| AFLA_123570 | amino acid transporter, putative |
| AFLA_123590 | glutathione S transferase, putative |
| AFLA_123650 | glutathione synthetase, putative |
| AFLA_123660 | conserved hypothetical protein |
| AFLA_123800 | conserved hypothetical protein |
| AFLA_123820 | short-chain dehydrogenase, putative |
| AFLA_123890 | conserved hypothetical protein |
| AFLA_123900 | RTA1 domain protein, putative |
| AFLA_123910 | protein RTA1, putative |
| AFLA_123920 | hypothetical protein |
| AFLA_123940 | peroxisomal copper amine oxidase |
| AFLA_124080 | amino acid transporter, putative |
| AFLA_124110 | galactose-proton symport, putative |
| AFLA_124120 | hypothetical protein |
| AFLA_124130 | MFS transporter, putative |
| AFLA_124140 | beta lactamase domain, putative |
| AFLA_124180 | monocarboxylate transporter, putative |
| AFLA_124190 | glycosyl hydrolase, putative |
| AFLA_124240 | hypothetical protein |
| AFLA_124250 | aminotriazole resistance protein, putative |
| AFLA_124350 | conserved hypothetical protein |
| AFLA_124370 | hypothetical protein |
| AFLA_124490 | guanine deaminase, putative |
| AFLA_124560 | conserved hypothetical protein |
| AFLA_124640 | Pfs, NACHT and Ankyrin domain protein |
| AFLA_124680 | myotrophin, putative |
| AFLA_124690 | vegetative incompatibility WD repeat protein, putative |
| AFLA_124700 | conserved hypothetical protein |
| AFLA_124720 | ankyrin repeat-containing protein, putative |
| AFLA_124760 | conserved hypothetical protein |
| AFLA_124770 | hypothetical protein |
| AFLA_124780 | efflux pump antibiotic resistance protein, putative |
| AFLA_124890 | hypothetical protein |
| AFLA_125040 | conserved hypothetical protein |
| AFLA_125050 | conserved hypothetical protein |
| AFLA_125130 | conserved hypothetical protein |
| AFLA_125140 | C6 transcription factor, putative |
| AFLA_125150 | MFS monocarboxylate transporter, putative |
| AFLA_125170 | oxidoreductase, putative |
| AFLA_125210 | conserved hypothetical protein |
| AFLA_125250 | 3-beta hydroxysteroid dehydrogenase/isomerase family protein |
| AFLA_125270 | tyrosinase |
| AFLA_125280 | hypothetical protein |
| AFLA_125460 | conserved hypothetical protein |
| AFLA_125550 | hypothetical protein |
| AFLA_125770 | LysR family regulatory protein, putative |
| AFLA_125940 | alcohol dehydrogenase, putative |
| AFLA_125960 | conserved hypothetical protein |
| AFLA_126140 | conserved hypothetical protein |
| AFLA_126210 | conserved hypothetical protein |
| AFLA_126550 | ornithine decarboxylase, putative |
| AFLA_126560 | conserved hypothetical protein |
| AFLA_126570 | conserved hypothetical protein |
| AFLA_126580 | sarcosine oxidase, putative |
| AFLA_126680 | WD repeat-containing protein, putative |
| AFLA_126690 | conserved hypothetical protein |
| AFLA_126700 | hypothetical protein |
| AFLA_126780 | beta-glucosidase 2 precursor, putative |
| AFLA_126820 | conserved hypothetical protein |
| AFLA_126830 | conserved hypothetical protein |
| AFLA_126840 | conserved hypothetical protein |
| AFLA_126850 | vacuolar protease A, putative |
| AFLA_126860 | cell wall cysteine-rich protein |
| AFLA_126920 | hypothetical protein |
| AFLA_126940 | hypothetical protein |
| AFLA_126990 | conserved hypothetical protein |
| AFLA_127120 | hypothetical protein |
| AFLA_127210 | pathogenesis associated protein Cap20, putative |
| AFLA_127310 | CAIB/BAIF family enzyme |
| AFLA_127400 | conserved hypothetical protein |
| AFLA_127470 | conserved hypothetical protein |
| AFLA_127550 | conserved hypothetical protein |
| AFLA_127670 | zinc-binding oxidoreductase, putative |
| AFLA_127680 | actin filament organization protein App1-like, putative |
| AFLA_127780 | conserved hypothetical protein |
| AFLA_127790 | Leucine Rich Repeat domain protein |
| AFLA_127800 | 40S ribosomal protein S26 |
| AFLA_127830 | Fe-containing alcohol dehydrogenase, putative |
| AFLA_127870 | vacuolar membrane PQ loop repeat protein |
| AFLA_127890 | protein phophatase 2C family protein |
| AFLA_127960 | conserved hypothetical protein |
| AFLA_128010 | nuclear polyadenylated RNA-binding protein Nab2, putative |
| AFLA_128070 | conserved hypothetical protein |
| AFLA_128100 | glycerol kinase, putative |
| AFLA_128110 | aquaglyceroporin, putative |
| AFLA_128140 | sister chromatid separation protein (Src1), putative |
| AFLA_128170 | NRPS-like enzyme, putative |
| AFLA_128440 | fumarylacetoacetate hydrolase, putative |
| AFLA_128470 | efflux pump antibiotic resistance protein, putative |
| AFLA_128580 | NADH-ubiquinone oxidoreductase 304 kDa subunit precursor |
| AFLA_128600 | 2-oxoisovalerate dehydrogenase complex alpha subunit, putative |
| AFLA_128610 | beta-mannosidase |
| AFLA_128620 | UDP-glucuronate 5-epimerase, putative |
| AFLA_128630 | ubiquinone biosynthesis methlytransferase Coq5, putative |
| AFLA_128640 | sugar isomerase, KpsF/GutQ |
| AFLA_128680 | flavin-binding monooxygenase, putative |
| AFLA_128690 | conserved hypothetical protein |
| AFLA_128710 | hypothetical protein |
| AFLA_128730 | ATP dependent RNA helicase, putative |
| AFLA_128740 | tRNA methyltransferase, putative |
| AFLA_128800 | proteasome component Pre9, putative |
| AFLA_128810 | conserved hypothetical protein |
| AFLA_128850 | conserved hypothetical protein |
| AFLA_128870 | feruloyl esterase, putative |
| AFLA_128920 | MFS multidrug transporter, putative |
| AFLA_129030 | conserved hypothetical protein |
| AFLA_129190 | lipase/esterase family protein |
| AFLA_129320 | glutaminase, putative |
| AFLA_129370 | cytochrome B5, putative |
| AFLA_129390 | conserved hypothetical protein |
| AFLA_129620 | sister chromatid cohesion protein Mis4, putative |
| AFLA_129690 | cell division control protein (Cdc5), putative |
| AFLA_129710 | exonuclease, putative |
| AFLA_129780 | hypothetical protein |
| AFLA_129850 | conserved hypothetical protein |
| AFLA_129870 | zinc binding dehydrogenase, putative |
| AFLA_129880 | hypothetical protein |
| AFLA_129960 | oligosaccharyl transferase subunit (alpha), putative |
| AFLA_130010 | hypothetical protein |
| AFLA_130080 | small nucleolar ribonucleoprotein complex subunit, putative |
| AFLA_130110 | hypothetical protein |
| AFLA_130120 | PHD finger and JmjC domain protein, putative |
| AFLA_130160 | mitochondrial respiratory complex I chaperone (Cia84), putative |
| AFLA_130200 | mRNA splicing factor (Prp1/Zer1), putative |
| AFLA_130250 | conserved hypothetical protein |
| AFLA_130260 | chromatin remodeling and histone acetyltransferase complexes subunit |
| AFLA_130360 | yippee family protein |
| AFLA_130430 | conserved hypothetical protein |
| AFLA_130440 | hypothetical protein |
| AFLA_130450 | nuclear division Rft1 protein, putative |
| AFLA_130480 | replication protein A 70 kDa DNA-binding subunit |
| AFLA_131170 | phosphatidylinositol 4-kinase (STT4), putative |
| AFLA_131220 | hypothetical protein |
| AFLA_131230 | conserved hypothetical protein |
| AFLA_131320 | hypothetical protein |
| AFLA_131390 | thioesterase family protein |
| AFLA_131400 | conserved hypothetical protein |
| AFLA_131570 | endo-arabinase, putative |
| AFLA_131590 | Type I phosphodiesterase / nucleotide pyrophosphatase family protein |
| AFLA_131600 | lysine-rich arabinogalactan protein 18 precursor, putative |
| AFLA_131640 | HLH transcription factor (Hpa3), putative |
| AFLA_131650 | hypothetical protein |
| AFLA_131690 | hypothetical protein |
| AFLA_131740 | aromatic ring-opening dioxygenase LigB subunit, putative |
| AFLA_131750 | NADH-ubiquinone oxidoreductase 105 kDa subunit |
| AFLA_131780 | conserved hypothetical protein |
| AFLA_131850 | G-protein beta WD-40 repeats containing protein, putative |
| AFLA_131860 | conserved hypothetical protein |
| AFLA_131880 | catecholamine-O-methyltransferase, putative |
| AFLA_131950 | carnitinyl-CoA dehydratase, putative |
| AFLA_132060 | conserved hypothetical protein |
| AFLA_132180 | aldehyde dehydrogenase ALDH |
| AFLA_132250 | MFS multidrug transporter, putative |
| AFLA_132260 | conserved hypothetical protein |
| AFLA_132300 | PAXNEB protein superfamily |
| AFLA_132410 | hypothetical protein |
| AFLA_132420 | PTH11-like integral membrane protein |
| AFLA_132460 | COPI-coated vesicle protein, putative |
| AFLA_132570 | translation initiation factor eIF-2B alpha subunit, putative |
| AFLA_132780 | conserved hypothetical protein |
| AFLA_132900 | protein methyltransferase RmtC |
| AFLA_132910 | pre-mRNA splicing factor RNA helicase (Prp43), putative |
| AFLA_132920 | conserved hypothetical protein |
| AFLA_132930 | secretory pathway protein Ssp120, putative |
| AFLA_133010 | hypothetical protein |
| AFLA_133020 | MYB DNA-binding domain protein |
| AFLA_133060 | conserved hypothetical protein |
| AFLA_133070 | protein-ER retention receptor, putative |
| AFLA_133090 | translation factor pelota, putative |
| AFLA_133100 | aldehyde reductase, putative |
| AFLA_133110 | conserved hypothetical protein |
| AFLA_133160 | tRNA splicing 2' phosphotransferase 1 |
| AFLA_133170 | conserved hypothetical protein |
| AFLA_133250 | conserved hypothetical protein |
| AFLA_133460 | Ku family DNA helicase, putative |
| AFLA_133470 | SNARE complex subunit (Vam7), putative |
| AFLA_133490 | aspartyl-tRNA synthetase Dps1, putative |
| AFLA_133510 | F-box domain protein |
| AFLA_133700 | conserved hypothetical protein |
| AFLA_133760 | aminotransferase, putative |
| AFLA_133860 | hypothetical protein |
| AFLA_133890 | DNA replication complex GINS protein (Psf2), putative |
| AFLA_133900 | glucosyltransferase (Die2), putative |
| AFLA_133970 | NOT2 family protein |
| AFLA_133980 | 50S ribosomal protein L4 |
| AFLA_134050 | PHD finger and SET domain protein, putative |
| AFLA_134070 | UDP-N-acetyl-glucosamine-1-P transferase Alg7, putative |
| AFLA_134080 | aryl-alcohol dehydrogenase Aad14, putative |
| AFLA_134370 | acetylglutamate synthase |
| AFLA_134380 | hypothetical protein |
| AFLA_134570 | Mob1 family protein |
| AFLA_134690 | conserved hypothetical protein |
| AFLA_134720 | conserved hypothetical protein |
| AFLA_134740 | 60S ribosomal protein L3 |
| AFLA_134750 | conserved hypothetical protein |
| AFLA_134860 | pentatricopeptide repeat protein |
| AFLA_134890 | 3-hydroxyacyl-CoA dehyrogenase, putative |
| AFLA_134920 | C6 transcription factor, putative |
| AFLA_134950 | YagE family protein |
| AFLA_134980 | voltage-gated chloride channel, putative |
| AFLA_134990 | midasin, putative |
| AFLA_135090 | cytochrome c oxidase assembly protein Cox11, putative |
| AFLA_135120 | kinetochore protein fta7, putative |
| AFLA_135360 | hydroxymethylglutaryl-CoA lyase |
| AFLA_135380 | conserved hypothetical protein |
| AFLA_135470 | ornithine decarboxylase, putative |
| AFLA_135490 | nonribosomal peptide synthase, putative |
| AFLA_135580 | WD repeat protein |
| AFLA_135700 | allergenic cerato-platanin Asp F13 |
| AFLA_135720 | conserved hypothetical protein |
| AFLA_135760 | hypothetical protein |
| AFLA_135850 | conserved hypothetical protein |
| AFLA_135940 | RNA polymerase II subunit 3 |
| AFLA_135970 | calcineurin binding protein, putative |
| AFLA_136010 | pyrimidine 5'-nucleotidase, putative |
| AFLA_136030 | chitin synthase ChsE |
| AFLA_136070 | DNA mismatch repair protein pms1, putative |
| AFLA_136120 | iron ABC transporter, putative |
| AFLA_136160 | SNF2 family helicase/ATPase, putative |
| AFLA_136280 | RNA polymerase II transcription mediator complex subunit Srb7 |
| AFLA_136310 | mitochondrial membrane fission protein (Fis1), putative |
| AFLA_136440 | universal stress protein family domain protein |
| AFLA_136460 | DRAP deaminase, putative |
| AFLA_136510 | conserved hypothetical protein |
| AFLA_136530 | hypothetical protein |
| AFLA_136550 | 3' exoribonuclease family protein (Rrp42), putative |
| AFLA_136860 | conserved hypothetical protein |
| AFLA_136930 | conserved hypothetical protein |
| AFLA_137010 | NADH-ubiquinone oxidoreductase 49 kDa subunit, putative |
| AFLA_137020 | DNA-directed RNA polymerase I and III subunit Rpc40, putative |
| AFLA_137060 | plasma membrane SNARE protein (Sec9), putative |
| AFLA_137080 | conserved hypothetical protein |
| AFLA_137110 | extracellular serine-rich protein, putative |
| AFLA_137120 | integral membrane protein |
| AFLA_137140 | 3-hexulose-6-phosphate synthase, putative |
| AFLA_137170 | hypothetical protein |
| AFLA_137370 | conserved hypothetical protein |
| AFLA_137390 | malate/L-lactate dehydrogenase, putative |
| AFLA_137580 | hypothetical protein |
| AFLA_137640 | conserved hypothetical protein |
| AFLA_137710 | hypothetical protein |
| AFLA_137720 | hypothetical protein |
| AFLA_137730 | conserved hypothetical protein |
| AFLA_137740 | conserved hypothetical protein |
| AFLA_138000 | chromosome segregation protein SudA, putative |
| AFLA_138010 | extragenic suppressor of the bimD6 mutation |
| AFLA_138200 | conserved hypothetical protein |
| AFLA_138370 | hypothetical protein |
| AFLA_138400 | NAD dependent epimerase/dehydratase, putative |
| AFLA_138450 | conserved hypothetical protein |
| AFLA_138700 | conserved hypothetical protein |
| AFLA_138710 | choline transport protein, putative |
| AFLA_138780 | conserved hypothetical protein |
| AFLA_138800 | conserved hypothetical protein |
| AFLA_138810 | hypothetical protein |
| AFLA_139090 | sugar transporter, putative |
| AFLA_139100 | aflYe/ orf/ Ser -Thr protein phosphatase family protein |
| AFLA_139230 | aflI/ avfA/ cytochrome P450 monooxygenase |
| AFLA_139240 | aflLa/ hypB/ hypothetical protein |
| AFLA_139310 | aflE/ norA/ aad/ adh-2/ NOR reductase/ dehydrogenase |
| AFLA_139340 | aflS/ pathway regulator |
| AFLA_139360 | aflR / apa-2 / afl-2 / transcription activator |
| AFLA_139470 | FAD dependent oxidoreductase, putative |
| AFLA_139540 | cytochrome P450, putative |
| AFLA_139660 | hypothetical protein |
| AFLA_139670 | nonribosomal peptide synthase, putative |
